# Conservation of an *Agrobacterium* cT-DNA insert in *Camellia* section *Thea* reveals the ancient origin of tea plants from a genetically modified ancestor

**DOI:** 10.3389/fpls.2022.997762

**Published:** 2022-12-06

**Authors:** Ke Chen, Peter Zhurbenko, Lavrentii Danilov, Tatiana Matveeva, Léon Otten

**Affiliations:** ^1^ Shanghai Key Laboratory of Plant Functional Genomics and Resources, Shanghai Chenshan Botanical Garden, Shanghai, China; ^2^ Department of Genetics and Biotechnology, Saint-Petersburg State University, Saint Petersburg, Russia; ^3^ Komarov Botanical Institute of the Russian Academy of Sciences, Saint Petersburg, Russia; ^4^ Institute of Plant Molecular Biology, Centre National de Recherche Scientifique (C.N.R.S.), Strasbourg, France

**Keywords:** natural genetically transformed organisms, cT-DNA, allele phasing, Camellia sinensis, Thea section, tea plant evolution

## Abstract

**Introduction:**

Many higher plants contain cellular T-DNA (cT-DNA) sequences from *Agrobacterium* and have been called “natural genetically modified organisms” (nGMOs). Among these natural transformants, the tea plant *Camellia sinensis* var. *sinensis* cv. Shuchazao contains a single 5.5 kb T-DNA fragment (CaTA) with three inactive T-DNA genes, with a 1 kb inverted repeat at the ends. *Camellia* plants are allogamous, so that each individual may contain two different CaTA alleles.

**Methods:**

142 *Camellia* accessions, belonging to 10 of 11 species of the section *Thea*, were investigated for the presence of CaTA alleles.

**Results discussion:**

All accessions were found to contain the CaTA insert, showing that section *Thea* derives from a single transformed ancestor. Allele phasing showed that 82 accessions each contained two different CaTA alleles, 60 others had a unique allele. A phylogenetic tree of these 225 alleles showed two separate groups, A and B, further divided into subgroups. Indel distribution corresponded in most cases with these groups. The alleles of the different *Camellia* species were distributed over groups A and B, and different species showed very similar CaTA alleles. This indicates that the species boundaries for section *Thea* may not be precise and require revision. The nucleotide divergence of the indirect CaTA repeats indicates that the cT-DNA insertion took place about 15 Mio years ago, before the emergence of section *Thea*. The CaTA structure of a *C. fangchengensis* accession has an exceptional structure. We present a working model for the origin and evolution of nGMO plants derived from allogamous transformants.

## Introduction

The genus *Camellia* belongs to the Theaceae family and contains several economically and culturally important species. *C. sinensis* consists of two main varieties: var. *sinensis* (generally used for green tea) and var. *assamica* (mainly used for black tea). *Camellia* systematics is still uncertain (Hoi et al., 2020). Species numbers range from 120 (Min and Bartholomew, 2007, based on Sealy, 1958) to 280 (Vijayan et al., 2009). Min and Bartholomew (2007) propose a Theaceae classification based on morphological criteria. In their system, subfamily Theoideae contains six genera. One of these, *Camellia*, is divided into subgenera *Thea* and *Camellia*. Subgenus *Thea* is further divided into six sections, among which section *Thea*. The tea plant, *C. sinensis* (L.) O. Kuntze, and the wild teas, belong to section *Thea*, which contains eleven species (according to Min and Bartholomew, 2007). *C. sinensis* is further divided in varieties *sinensis*, *assamica*, *pubilimba* and *dehungensis*.

Although the name “Thea” has been used to designate the genus *Camellia*, it is now only used for the subgenus *Thea* (which belongs to the genus *Camellia*) and for the section *Thea* (which belongs to the subgenus *Camellia*, itself part of the genus *Camellia*). Because of the economical and cultural importance of the genus *Camellia*, many efforts have been made to reconstruct its molecular evolution. The complex genetic relations between cultivated tea plants and their wild ancestors have been studied extensively (Prince and Parks, 2001; Wachira et al., 2001; Chen and Yamaguchi, 2002; Xiao and Parks, 2003; Sharma et al., 2009; Vijayan et al., 2009; Sharma et al., 2010; Liu et al., 2012; Yao et al., 2012; Yang et al., 2013; Huang et al., 2014; Zhang et al., 2014; Xu et al., 2015; Meegahakumbura et al., 2016; Yang et al., 2016; Wang et al., 2020; Xia et al., 2020; Zhang et al., 2020b; Wu et al., 2022); Zhang et al., 2020b.

Nuclear DNA-based *Camellia* phylogenies have been proposed on the basis of ribosomal DNA (Vijayan et al., 2009; Xu et al., 2015; Zhang M. et al., 2021), single-nucleotide polymorphisms (SNPs) and restriction-site associated (RAD) markers (Yang et al., 2016), transposons (Yao et al., 2017) and transcript sequences (Zhang et al., 2022; Wu et al., 2022). Recently, whole genome sequencing has provided new opportunities for phylogenetic tree construction (Wei et al., 2018; Wang et al., 2020; Xia et al., 2020; Zhang et al., 2020b; Zhang et al., 2020b; Zhang X. et al., 2021). In Next Generation Sequencing (NGS) data, DNA sequences newly acquired as a result of horizontal gene transfer can be found. They can also be used as a tool to study phylogeny (Matveeva et al., 2011). Thus, many dicotyledonous species have been found to harbor one or several *Agrobacterium* DNA fragments in their genomes. These sequences are called cellular T-DNAs or cT-DNAs, and the resulting plants have been called natural genetically modified organisms (nGMOs) (Matveeva and Otten, 2019; Matveeva, 2021a).

*Agrobacterium* is a phytopathogenic bacterium with a unique mechanism to transfer well-defined DNA fragments (T-DNAs) into the nuclear genome of a large variety of dicotyledonous plant species (Chilton et al., 1977; Zhu et al., 2000; Nester, 2015; Gelvin, 2017; Lacroix and Citovsky, 2019; Matveeva and Otten, 2019). Normally, the transfers result in Crown gall tumors (in the case of *Agrobacterium tumefaciens* or *A. vitis*) or hairy roots (in the case of *A. rhizogenes*, also called *Rhizobium rhizogenes*). In both cases, expression of T-DNA genes leads to abnormal growth and to the synthesis of small molecules, called opines. Opines can be degraded by the transforming Agrobacteria; they constitute the “raison d’être” of the transformation process (Petit and Tempé, 1985; Dessaux et al., 1998). It has been experimentally demonstrated that hairy roots can regenerate into fertile plants (Tepfer, 1990; Christey, 2001; Lütken et al., 2012).

After the initial discovery of cT-DNA sequences in *N. glauca* and other *Nicotiana* species (White et al., 1983; Furner et al., 1986; Suzuki et al., 2002), further nGMOs were discovered: *Linaria* (Matveeva et al., 2012), *Ipomoea* (Kyndt et al., 2015) and *Cuscuta* (Zhang Y. et al., 2020). Detailed evolutionary studies on the cT-DNAs of *Nicotiana* (Chen et al., 2014; Chen et al., 2018) revealed 4 different cT-DNA sequences (called TA to TD) in the ancestor of *N. tabacum* (tobacco), *N. tomentosiformis*. They occur in different combinations among *Nicotiana* species of section *Tomentosa*, and result from independent transformation events during the evolution of section *Tomentosa*. Additional cT-DNAs were found outside section *Tomentosa*, showing the widespread occurrence of natural transformation in this genus (Otten, 2020). Interestingly, some cultivars of the nGMO *N. tabacum* (Chen et al., 2016) and *Cuscuta* (Zhang Y. et al., 2020) have been shown to produce opines, but the biological significance of this is not yet clear. The frequent presence of inverted repeats in cT-DNAs provides relative dates for their insertion (Chen et al., 2014; Chen et al., 2018; Otten, 2020). Evolutionary data are also available for *Linaria* (Matveeva, 2018) and *Ipomoea* (Quispe-Huamanquispe et al., 2019). The number of nGMOs was considerably enlarged when it was discovered that 7-10% of dicotyledonous plants carry cT-DNA sequences. Among these, *Camellia sinensis* carries a 5.5 kb cT-DNA (Matveeva and Otten, 2019).

In this paper we compare a large number of phased alleles of the cT-DNA sequence CaTA obtained from different *Camellia* species of section *Thea*. The results show that CaTA sequences from different *Thea* section species do not cluster as expected, and therefore require further investigation of the *Thea* section phylogeny.

## Materials and methods

### Recovery and assembly of cT-DNA reads

*Camellia* sequences of the SRR/SRX type (as of June 2022) were blasted to the *Camellia sinensis* var. *sinensis* G240 cultivar CaTA query sequence, using the discontiguous megablast (Altschul et al., 1990; O'Leary et al., 2016). Recovered reads were assembled and alleles separated using the allele separation function of CodonCode Aligner (version 10.0.2). (CodonCode Corporation). Apart from Default settings, a value of 100% of Minimum Percent Identity was used, in order to separate alleles with sequences as similar as possible (down to about 10 nt differences per 5500 nt). Some descriptions of *Camellia* sequences investigated here use species names not recognized by Min and Bartholomew (2007), but mentioned by them as synonyms. These are *quinquelocularis* and *tetracocca* (synonyms of *tachangensis*), *atrothea* and *makuanica* (*crassicolumna*), and *pubilimba* and *angustifolia* (*sinensis*).

### Sequence alignments and phylogenetic analysis

After allele phasing, full-length CaTA sequences were aligned using MUSCLE (Edgar, 2004) with subsequent manual indel deletion in MEGA v.11 (Tamura et al., 2021). The phylogenetic analysis was carried out by IQ-TREE v.1.6.1 (Nguyen et al., 2015) using the maximum likelihood method with 1000 bootstrap replicates and *C. fangchengensis* CaTA as outgroup. The tree was visualized using iTOL (Letunic and Bork, 2021).

## Results

### Structure of the *C. sinensis* cT-DNA

Matveeva and Otten (2019) reported a cT-DNA in *C. sinensis* var. *sinensis* commercial variety Shuchazao (accession SDRB01002054.1, gene coordinates 1834755-1838742). In the present paper, we used the 98% identical cT-DNA sequence from *C. sinensis* var. *sinensis* G240 (Zhang et al., 2020a) as the model sequence. We will call this cT-DNA “CaTA” (for *Camellia* TA sequence). CaTA is located on contig JACBKZ010000009.1 (chromosome 9), coordinates 78660407-78665693. Here we investigate this cT-DNA sequence and its surrounding 200 nt flanking plant sequences (together 5687 nt) in various *Camellia* accessions, using G240-CaTA coordinates as a reference.

**Figure 1:**
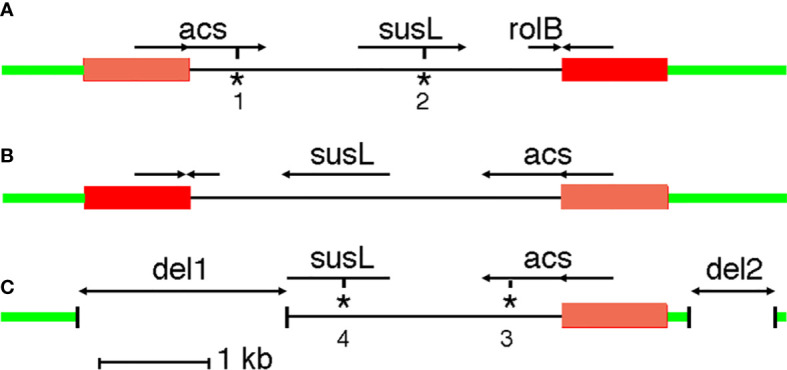
CaTA maps. **(A)** CaTA map common to all accessions, except *C. fangchengensis*. **(B)** First putative step in origin of *C. fangchengensis* CaTA. Inversion of the central part. **(C)** Second putative step in origin of *C. fangchengensis* CaTA: two deletions (del1 and del2). The plant DNA surrounding the CaTA insert is marked in green, the CaTA inverted repeats are marked in orange (left repeat) and red (right repeat). *acs*: agrocinopine synthase gene, *susL*: L,L-succinamopine synthase gene, *rolB*: root locus B gene. *1: stop codon at 1556, *2: 32 nt deletion at 3182 and frameshift, *3: stop codon at 1538, *4: 1 nt deletion at 3147 and frameshift.

G240-CaTA carries an inverted repeat on the left (TGCTCGCGTG….GTCGCTTGGC, 201-1148) and right (GCCAAGCGAC….CGCTCGAGCA, 4528-5487). The 948 nt and 960 nt repeats are 90% identical. The 5287 nt G240-CaTA sequence is flanked by a moderately repeated plant sequence on the left, and a highly repeated plant sequence on the right. G240-CaTA (**Figure 1A**) shows regions with homology to agrocinopine synthase (*acs*) genes (CaTA-*acs*, 642-1840), starting in the left repeat, an L,L-succinamopine synthase (*susL*) gene (CaTA-*susL*, 2677-3657) in the central unique part, and a truncated root locus B (*rolB*) sequence (CaTA-*rolB*, 4218-4532) on the right, interrupted by the right repeat. Thus, CaTA is derived from a 4327 nt partial T-DNA fragment, followed by a second fragment similar to its left end, but linked to it in an inverted orientation (right arm). We assume the repeats were originally identical.

The closest *Agrobacterium* relative of CaTA-*acs* is found in *A. tumefaciens* strain Kerr14, but the corresponding proteins are only 31% identical. *R. rhizogenes* strains also carry *acs* genes (Otten, 2021), these are even less similar. However, the nGMO *Parasponia andersonii* (Matveeva and Otten, 2019) potentially encodes much more similar Acs sequences (PON34801, PON34802, PON34803, about 75% identity). The same difference is found for CaTA-SusL: 37% identity for SusL from *R. rhizogenes* strain A4, but 52% for *P. andersonii* PON40029.1 SusL. The protein fragment deduced from the partial *rolB* gene has 46% identity to *R. rhizogenes* K599 RolB. CaTA-*acs* and CaTA-*susL* are interrupted by premature stop codons. The gene order *acs-susL-rolB* has not been found earlier in *Agrobacterium* and represents a new type of T-DNA.

### CaTA insertion site

**Figure 2:**
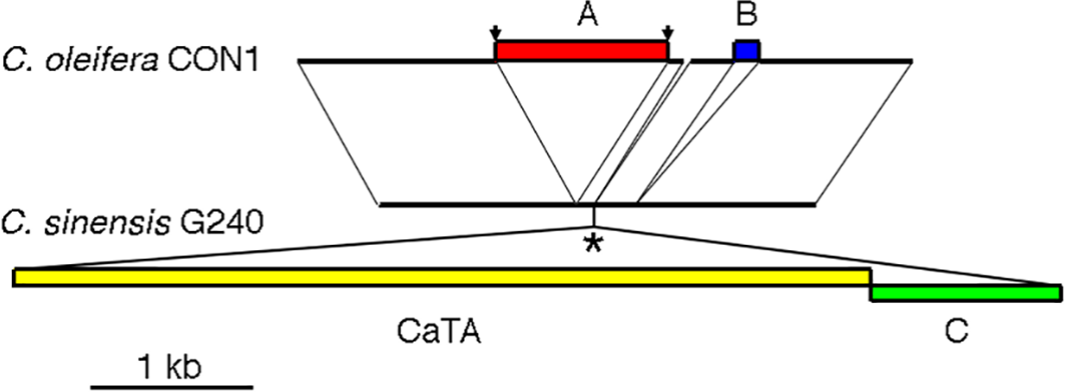
Sequence of the CaTA-less species *C. oleifera* isolate CON1 (top) at the CaTA insertion site of *C. sinensis* G240 (bottom). **(A)** insert in *C. oleifera* CON1, flanked by inverted repeats TGCAACTAAG at its ends, and a direct repeat of the original target sequence GTCCAGCT (arrows). **(B)** insert in *C. oleifera*. *: putative site of insertion of the *C. sinensis* G240 CaTA insert ligated to an adjacent plant sequence (indicated by **(C)**.

The original, unmodified CaTA insertion site might be preserved in *Camellia* species without CaTA. As the CaTA insert is surrounded by repeated sequences, no short SRR/SRX sequences from other *Camellia* species could be used to reconstruct this site. We therefore used the assembled genome from *C. oleifera* isolate CON1 (JAKJMZ), which does not carry CaTA, to search for the insertion site. A 45 kb fragment of G240 containing G240-CaTA and surrounding sequences (JACKBKZ010000009.1:78640000-78685000) was used as a query sequence. This detected a colinear region on *C. oleifera* chromosome 5 (JAKJMZ010000005.1:112492914-112526157), with an expected gap at the position of the CaTA DNA. The alignment of a 3.7 kb *C. oleifera* CON1 region with the corresponding G240 region is shown in **Figure 2**. From left to right the aligment shows a 1219 nt common region, followed by a 1042 nt insert in CON1 (insert A). Insert A has an 10 nt inverted repeat TGCAACTAAG at its ends, and a direct repeat of the original target sequence GTCCAGCT. Insert A is absent in two other CaTA-less accessions, *C. oleifera* SRX10143503, and *C. sasanqua* SRX8770629. It is followed by a small 88 nt region, also found in G240. After this, G240 shows the 5286 nt CaTA region, and to the right of it, a 872 nt plant sequence (insert B). A sequence similar to insert B (94% identity) is found in chromosome 3 of G240 (located at JACBKZ010000003.1:79203545-79204449) and could have been ligated to the CaTA fragment before insertion. A 73 nt CON1 fragment (112515620-112515692) seems to have been lost in G240. Insert B is followed by a 250 nt sequence common to G240 and CON1, a 162 nt CON1-specific sequence (marked in blue), and a 930 nt common sequence.

### CaTAs in different *Camellia* accessions of section *Thea*

Since the *Agrobacterium*-mediated insertion of T-DNA sequences in plant DNA is random, every insert is unique and marks the origin of a new clade (Matveeva et al., 2011; Chen and Otten, 2017). In other words, the presence and distribution of a particular cT-DNA insert among related nGMO species reflects their origin from a single transformant, and can be used to reconstruct the evolution of such a group. We therefore investigated the occurrence of CaTA sequences in *C. sinensis* and other species of section *Thea*. For this, we not only used whole genome sequences (as in Matveeva and Otten, 2019), but also investigated short (150 nt) sequence read runs (SRR) from hundreds of *Camellia* accessions. This considerably enlarged the search for cT-DNA sequences, but required assembly of individual reads into full CaTA sequences. SRR reads homologous to G240-CaTA were recovered from accessions belonging to 9 *Thea* section species. CaTA alleles of each accession were phased, with manual correction when necessary (Materials and Methods). After selection of 142 accessions with sufficient coverage for phasing (**Supplementary Table 1**), 60 showed unique alleles, 82 had two different alleles. In all, 225 alleles were identified (**Supplementary Table 2**, Materials and Methods, see below). The most diverse sequences showed 7.3% divergence. The identity values for the left and right repeat of each CaTA sequence were remarkably similar: 90.3 (SD: +/-0.6%). This suggests the absence of gene conversion between the repeats, and provided a good starting point to calculate the age of the CaTA insertion (see below).

The CaTA sequence of *C. fangchengensis* (SRX10143457, yielding two alleles with 98% identity) is inserted in the same site as the other CaTAs, but its structure is unusual. It is best explained as a derivative of the more common CaTA form, generated by an inversion of the unique region between the two repeats (**Figure 1B**), followed by two deletions (**Figure 1C**). The inversion of the central region could result from recombination between the flanking inverted repeats. One of the alleles carries a plant insert (ins2824, **Table 1**). Overall identity values of the two *C. fangchengensis* CaTA alleles with other CaTAs are relatively low, the best matches, sin8233452_2, pubi8770663_1 and assam8233492_2 show only 92.7% identity. Thus, *C. fangchengensis* CaTA separated at an early stage from the other CaTAs. Both *fangchengensis* alleles carry an additional 32 nt sequence after CaTA3182 (within *susL*). This sequence is found in all *Agrobacterium susL* sequences (Otten, 2021), suggesting that the 32 nt fragment was lost in the ancestor of section *Thea*, but retained in *C. fangchengensis* CaTA.

**Table 1:** Summary of indels in different CaTA alleles.

	type	sequence change	direct repeat	x
239	del	TGCG=A		10
**255**	**ins**	**Plant repeat**	**GCCTT**	**1**
258	del	CTC		3
438	del	AATTTTG		1
440	del	TTTTG		1
441	del	TTTGTAG (also in G240)		134
442	del	TTGTAGA		3
444	del	GTAGAA		1
446	ins	AGA		1
449a	ins	AGCCCGTAGAATTTTGTAGA		1
449b	ins	AGCCCGTAGAATTTTGTAGAAGCCCGTAGAATTTTGTAGA		1
449c	Ins	AGAGGG		3
**626**	**ins**	**Plant repeat**	**GATTTAG**	**1**
640	del	CCTTGGCCG		1
1012	del	AAGAGCGAGTTCAC		1
1277	ins	CGCTAATGCC=GCTCTGGTAGTTCCC		2
1508	del	AGA		7
1712	del	AGCCTG		1
1790	del	AAAGCCGTGG=CGATCT		1
1928	del	GGAGTTT		2
**1946**	**ins**	**Plant repeat**	**AATCGC**	**17**
2001	del	CTT (=TCT)		1
2146a	del	TGCCTGGCCCG		2
2146b	del	TGCCTGGCC		1
2152	del	GGCCC		15
2337	dupl	ATCATGACATCC/GG		3
2553	del	AAACTGGACCTTTATTACTGTC		1
2644	del	AGCGTTCGTGAATCTGCCAGGC		1
2744	ins	CACC		1
**2748**	**ins**	**Plant repeat**	**CCTGAATTCA**	**8**
2767	del	TCAGAGGCTTT		1
2768	del	AGAGGCTTTTG		1
2769a	del	GAGGCTTT		1
2769b	del	GAGGCTTTTGG/GCTTTTGGGAG		3
2770	ins	AAGGCTTTTGG		1
2772	del	GCTTTTGGGAG		3
2774	del	TTTTGGGAGT		1
2779a	del	GGAGTCTTTTG/C(T)TTTTGGGAGT (also in G240)		55
2779b	del	GGAGT		1
2781a	del	AGTCTTTTTGC		2
2781b	del	AGTCTTTTTGCC		1
**2824**	**ins**	**Plant repeat**	**GATCGAGATC**	**1**
3085	del	TCT/CTT		3
3182	ins	GCGGTGGAAAGTCTAATCACGAGTTGGTCGAG		2
3383	ins	TCT		1
3471	ins	TTC		1
3525	ins	AGATTG		1
3574	del	TTT		1
3664	del	CAAAACCCTCTTAGGATTGCGCTCTTCAGAAGGTGCGCCTCCCCAGTCA		1
3687	del	CTTCAGAAGGTGCGCC		3
3736	del	GCTCAAG		1
3789	del	TAA/AAT		2
3905	ins	GATCGG		1
3962a	del	TTCGGAAGC		40
3962b	del	TCGGAAGT		5
3962c	del	TTCGGAA=GC		1
3962d	ins	TTCGGAAGC=GACGCAGGAGCTT		1
3962e	ins	TTCGGAAGC=GACGCA		1
3962f	ins	TTCGGAAGC=GACGCAGCA		1
3969	del	AGC		1
4183	del	TCACCGGC		4
4440	del	TGC/TAC		14
5238	del	CTACAAA		1
**5347**	**ins**	**Plant repeat**	**AGCATGTG**	**1**

Positions are from G240-CaTA, and indicate last nucleotide before modification. Plant repeats are indicated in bold. The direct repeats represent the duplication of the CaTA sequence upon insertion of the plant sequence. X: number of alleles with this particular modification. For presence and location of indels in different alleles, see **Supplementary Table 1**.

Indels in CaTA. Numbers refer to last nucleotide before change in G240-CaTA. x: numbers of alleles with this indel.

For *C. costata*, only one accession was available (SRR17596358) with 19 CaTA reads. *C. danzaiensis* (SRR19266665, 16 reads) and *C. kwangtungensis* (SRR19266666, 10 reads) are both considered to be synonyms for *C. costata* (Min and Bartholomew, 2007). The altogether 45 reads are insufficient for allele phasing, but show the presence of a CaTA in *C. costata*. No sequences were available for *C. grandibracteata*. Thus, our data show that at least 10 of the 11 *Camellia* species of section *Thea* contain the CaTA insert.

None of the 225 CaTA alleles has an open reading frame. All *acs* sequences have a TGA at CaTA position 1556 (ATCGCGAAATGA), except *C. fangchengensis* CaTA. However, the latter has a TAA at 1538 (GATTCAACTTAA), where all others have TCA. All *susL* sequences, except the one from *C. fangchengensis*, have a 32 nt deletion at 3182 (see above), leading to a frame shift. *C. fangchengensis susL* has a deletion at the 3’ end, due to CaTA rearrangements (**Figure 1C**), and an insertion of a G (TCATGGTGC**G**) at 3147, leading to a frame shift.

### CaTA polymorphism

**Figure 3:**
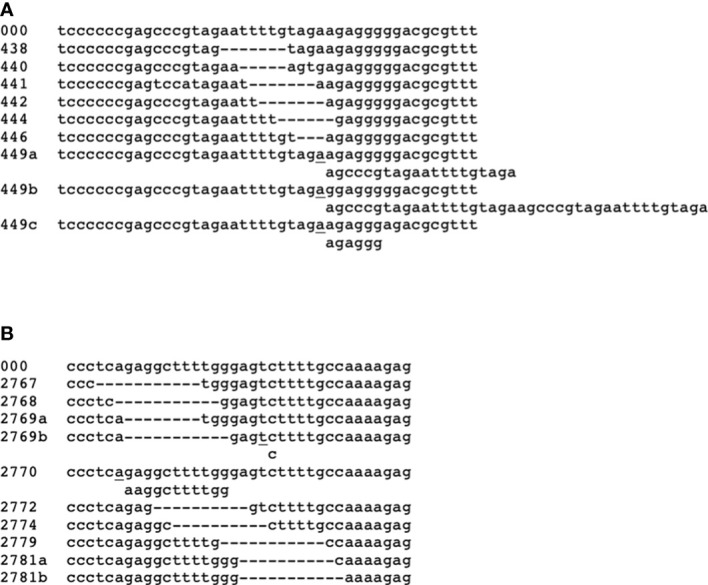
Structures of two variable CaTA regions. **(A)** Region around G240-CaTA coordinates 438-449, showing 6 different types of deletion, the region starting from 449 shows three types of insertion. **(B)** Region around 2767-2781. 10 different deletions and one insertion were found in this region. Coordinates on the left indicate the positions after which the sequence diverges from the standard sequence, the latter indicated by 000. Insertions are shown below the normal sequence and start behind the underlined nucleotide. See also **Table 1** and **Supplementary Table 1**.

Indels provide interesting markers for evolution. We investigated the occurrence and distribution of indels longer than 2 nucleotides in the 225 CaTA alleles (overview in **Table 1**, details in **Supplementary Table 3**). The location of each modification is indicated by the coordinate of the G240-CaTA sequence after which the sequence starts to be different. Six different plant inserts were found at positions 255, 626, 1946, 2748, 2824, and 5347. Those at 1946 and 2748 were found in 15 and 8 accessions respectively, the others are unique. All are flanked by short direct repeats at the insertion site and could be related to transposons. These inserts are highly repeated in the plant genome, and therefore could only partially be reconstructed in the CaTA assembly process. Further reconstruction will require longer reads. Two locations (438-442d and 2767-2781b) are highly polymorphic (**Figure 3**).

### Distribution of CaTA variants among different accessions

**Figure 4:**
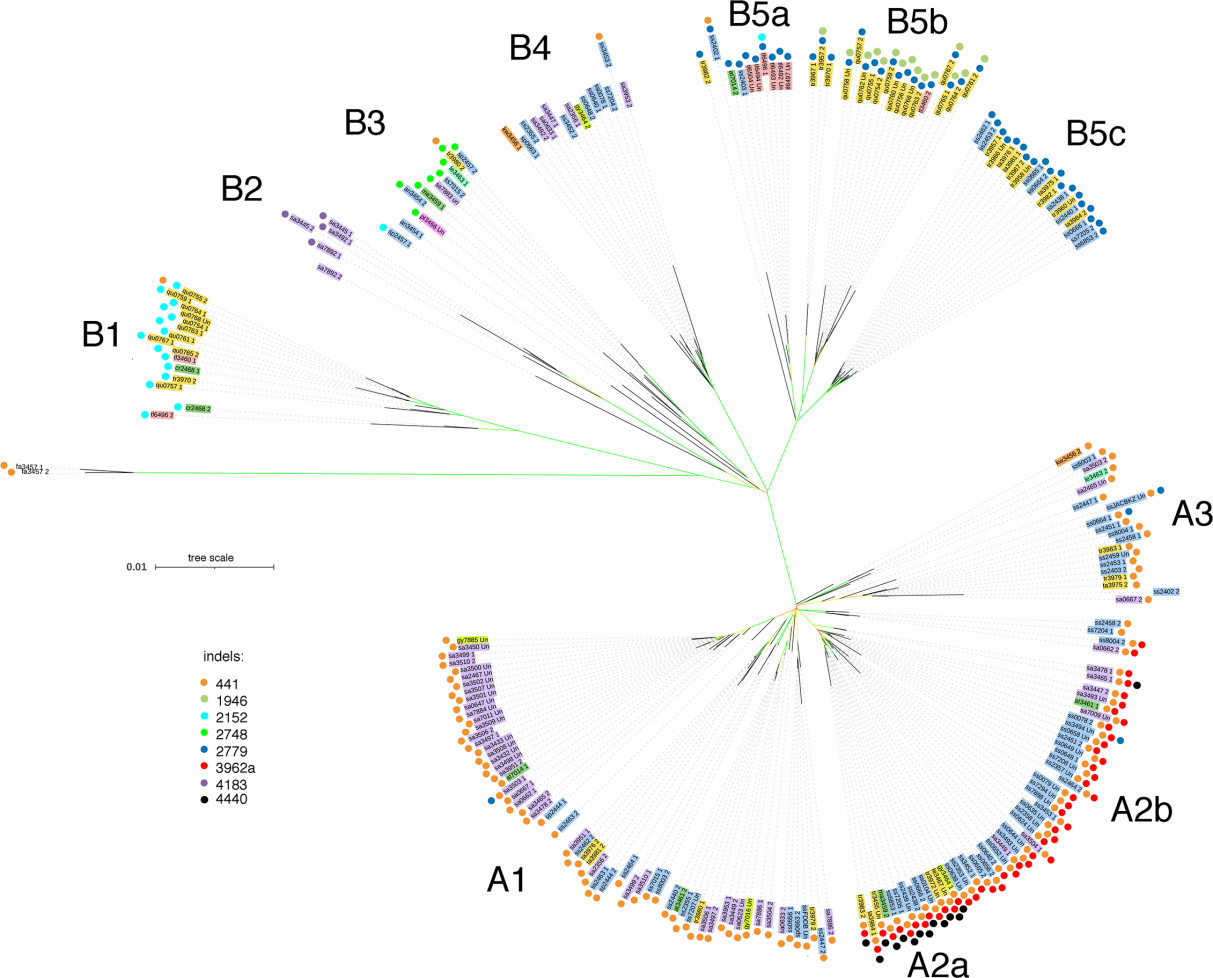
Phylogenetic tree of 225 CaTA alleles of 9 *Camellia* species of section *Thea*. CaTA from *C. fangchengensis* was used as outgroup. Accessions are marked by abbreviations corresponding to their full names given in **Supplementary Table 1** and the last 4 digits of the SRX number. Color codes indicate the different species (according to Min and Bartholomew, 2007). *C. sinensis* var. *assamica* belongs to *C. sinensis*, but was given a special color code (purple). *C. sinensis*: blue (these comprise *C. sinensis* var. *sinensis*, *C. pubilimba*, *C. angustifolia*). *C. tachangensis* (*C. tachangensis* var. *tachangensis*, *C. tachangensis* var. *remotiserrata*, *C. quinquelocularis*, *C. tetracocca*): dark yellow, *C. crassicolumna* (*C. crassicolumna*, *C. atrothea*, *C. makuanica*): green, *C. gymnogyna*: yellow, *C. taliensis*: pink, *C. leptophylla*: dark green, *C. kwangsiensis*: orange, *C. ptilophylla*: red, *C. fangchengensis*: no color. On the outside, the indels are marked with colored dots. Orange: deletion at G240-CaTA coordinate 441. Dark green: insertion at 1946. Light blue: insertion at 2152. Light green: insertion at 2748. Dark blue: deletion at 2779. Red: deletion at 3962a. Purple: deletion at 4183. Black: deletion at 4440. The various groups cluster into 2 main groups, A and B. A can be further subdivided into A1-A3, A2 into A2a and 2b. Group B is subdivided into B1-B5, B5 into B5a-c. In general, indel distribution corresponds to overall tree topology, but exceptions occur. Group B3 contains CaTAs of 5 different species, all have the 2748 plant sequence insert. B**ootstrap values are color-coded**: green 85-100%, yellow around 50-85%, red: less than 20%.

The phased CaTA sequences (**Supplementary Table 2**) were used to construct a phylogenetic tree (see Materials and Methods). *Camellia* species (according to Min and Bartholomew, 2007) were color-coded in order to visualize their distribution. Information on various indels was added to the tree (colored dots) in order to investigate whether indel distribution corresponded to tree topology. Two main groups can be distinguished (**Figure 4**), called A (mainly with del441) and B (mainly without del441). Group A is subdivided in A1 (mainly with *C. sinensis* var. *assamica*, sa), A2 with del441 and del3962a (mainly with *C. sinensis* var. *sinensis*, ss), A2a also has del4440, A2b does not. A3 is a mixed group. Group B can be divided in B1, with del2152 (mainly *C. quinquelocularis*, qu), B2: with del4183 (all sa), B3: ins2748 (mixed group), B4: mixed group, B5: del2779, with B5a (mainly *C. taliensis*, tl), B5b (qu), and B5c (*C. tachangensis* var. *tachangensis* (ta), *C. tachangensis* var. *remotiserrata* (tr), and ss). A few indels occur in unexpected branches: del441 in B1, B3, B4, and B5a, and del2779 in A1, A2b, and A3.

CaTA sequences of different *Thea* section species are interspersed. A striking example is B3 which contains sequences from *sinensis* (var. *pubilimba*, *angustifolia*, *assamica*), *makuanica*, *tachangensis* var. *remotiserrata*, *leptophylla*, and *ptilophylla*. These sequences also share the same plant insert (ins2748, **Table 1**), confirming their close relationship.

**Figure 5:**
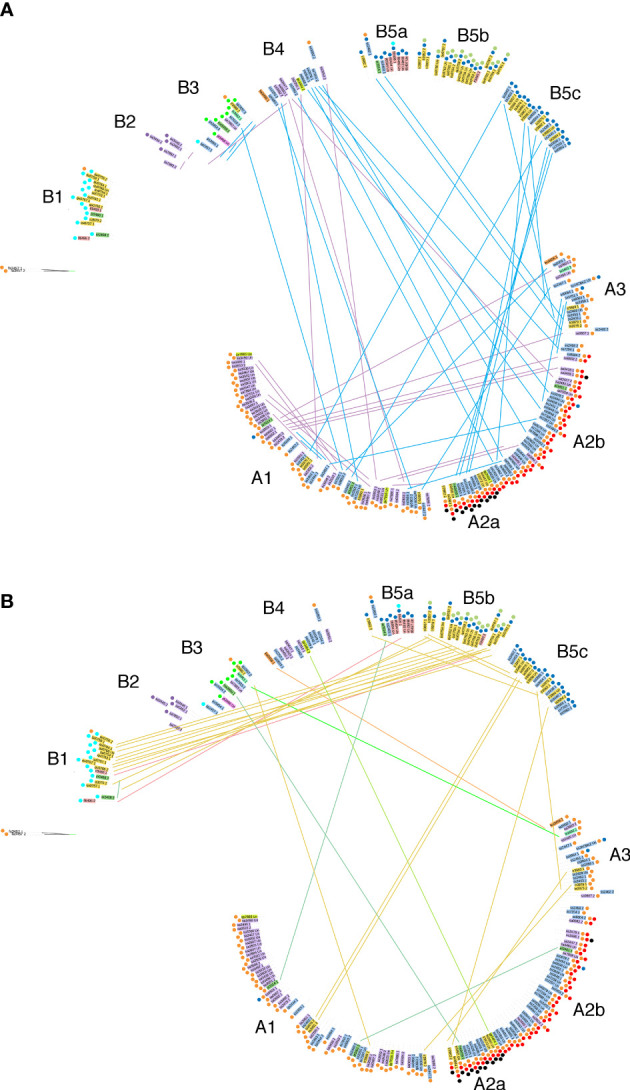
Localisation of paired alleles on the phylogenetic tree (same as in **Figure 4**) Paired alleles are connected by lines, with the same species color code as in **Figure 4**. Several pairs have one member in group **(A)**, the other one in group **(B)**.

We also investigated the distribution of allele pairs on the tree. Many pairs have one member on branch A, the other on branch B. An overview for the cultivated tea varieties *C. sinensis* var. *sinensis* and *assamica* is shown in **Figure 5A**, for the other *Thea* section species in **Figure 5B**. Interestingly, the nine *quinquelocularis* accessions with two dissimilar alleles (plus one *C. taliensis* accession) each have one allele on branch B1, the other on B5b. The distribution of these alleles suggests that the *quinquelocularis* group constitutes a *bona fide* subgroup derived from a founder plant with two different CaTA alleles, each of which diverged separately.

## Discussion

### Structure of CaTA cT-DNA and its occurrence in section *Thea*

*Camellia sinensis* var. *sinensis* cultivar Shuchazao was earlier found to contain a 5.5 kb cT-DNA insert (Matveeva and Otten, 2019). At the time it was unknown whether this represented an exceptional case or not. The results reported here show that at least 10 out of 11 *Camellia* species of section *Thea* (according to Min and Bartholomew, 2007) contain this insert, CaTA. The exception is *C. grandibracteata*, for which no sequences are available, this species needs further study. The original CaTA insertion site could be reconstructed using an assembled *C. oleifera* sequence, which lacks CaTA. The CaTA *acs-susL-rolB* structure is unusually small for a T-DNA, and has only low similarity to T-DNAs from *Agrobacterium* (Otten, 2021). The closest relatives are two cT-DNAs from *P. andersonii*, Pa3 and Pa6 (Matveeva and Otten, 2019). Interestingly, the *C. fangchengensis* CaTA structure is different from the other CaTAs, and its overall sequence is more diverged. This seems to set *C. fangchengensis* apart from other *Thea* section species. A recent study also found *C. fangchengensis* at the base of section *Thea* (Zhang X. et al., 2021). (Note that in this study *C. fangchengensis*, named after the city of Fangcheng in the province of Guangxi,was indicated as *C. fengchengensis*). The phylogenetic position of *C. fangchengensis* merits further analysis, using additional *fangchengensis* accessions and further nuclear sequences.

### Allele separation of CaTA sequences

About 70% of Angiosperms species are significantly heterozygous (Wright et al., 2013). The use of unphased mosaic sequences in molecular phylogeny leads to imprecise trees. So far, no attempts have been made to separate cT-DNA alleles of heterozygous nGMO species. In the case of *Nicotiana*, this was not necessary because *Nicotiana* is largely autogamous and homozygous. However, published cT-DNAs sequences from heterozygous nGMO species like *Linaria* (Matveeva et al., 2012) and *Ipomoea* (Kyndt et al., 2015; Quispe-Huamanquispe et al., 2019) are unphased. Allele separation algorithms are now commercially available, but still require manual adjustments, and a sufficiently high coverage. In the present study, we were able to isolate and phase 225 CaTA alleles from SRR/SRX data. Other *Camellia* sequences are available, but coverage values were insufficient for phasing. The 225 CaTA alleles provide a detailed view of CaTA variability and evolution. They can therefore be used as preliminary indicators for the taxonomic position of *Camellia* species of section *Thea*.

### CaTA alleles and phylogeny of *Camellia*

The phylogeny of *Camellia* might be difficult to unravel, as different *Camellia* species have been found to hybridize (Wight, 1962; Takeda, 1990; Sharma et al., 2010; Wang et al., 2020; Zhang et al., 2020a). *C. taliensis* wild type plants have been cultivated in tea gardens or were recently domesticated, as they are considered to be interesting sources for tea plant improvement. Having a 5-locule ovary, they are easily differentiated from the 3-locule ovary *sinensis*/*assamica* group (Zhao et al., 2014). Possibly, some 5-locule species considered as wild tea species, might in fact carry sequences from cultivated species, due to man-made crosses. This has been shown for *C. taliensis* var. *bangwei* (Yang et al., 2016), but other cases may go unnoticed if the genetic contribution from the cultivated species is small. It has also been suggested that some wild tea trees could in fact be of feral origin (Zhang et al., 2020a).

A study based on restriction-site-associated DNA (RAD) sequencing (Yang et al., 2016) found a cluster for the cultivated species *C. sinensis* var. *sinensis* (6 accessions), and one for *C. sinensis* var. *assamica* (3). Non-cultivated species *C. tachangensis* (1), *C. taliensis* (3) and *C. crassicolumna* (4) clustered together, separately from the cultivated species. Another study (Vijayan et al., 2009) showed clear separation between *taliensis* and *kwangsiensis* on the one hand, and *assamica*, *leptophylla*, *angustifolia*, *atrothea*, *ptilophylla* and *sinensis* on the other hand. These two studies did not use allele phasing. Our study yielded a tree composed of two different parts, A and B, with subgroups A1-A3 and B1-B5. Although the tree shows a concentration of *C. sinensis* var. *sinensis* accessions in A2 and A3, of *C. sinensis* var. *assamica* in A1, and of wild species in B1 and B5, there are several exceptions. Most importantly, when the distribution of paired alleles is taken into account, many accessions can be seen to have one allele in group A and one in group B. Thus, there is no strict separation of CaTA sequences between the different accessions and species. The A and B group may result from expansion of two dissimilar alleles. The origin of this combination may be due to a unique hybridization event between members of two already diverged populations. The distribution of the *C. quinquelocularis* allele pairs over group B1 and B5b indicates the presence of another founding ancestor plant with a specific combination of alleles. A recent study based on transcript analysis (Wu et al., 2022) obtained a subtree for 9 species of the *Thea* section (according to Min and Bartholomew, 2007). Different species were interspersed with *C. sinensis*, and *C. taliensis* (a single accession) was found at the base of section *Thea*. This study did not attempt to separate the alleles. The impact of this is difficult to estimate, also because the authors used other accessions as we did.

### Comparison to phylogenies based on assembled *Camellia* genomes

Recent papers report assembled *Camellia* sequences and their use in phylogenetic tree construction. Comparison between an unphased CSA and CSS genome yielded a sequence identity of 92,4% and a 0.38-1.54 Mya divergence time (Wei et al., 2018). However, our comparison of several CaTA alleles from CSA and CSS shows no clear separation, as CSA alleles occur in A1, A2a, A2b, A3, B2, B3, B4, and CSS alleles in A1, A2a, A2b, A3, B3, B4, B5a, B5c (**Figure 4**).

Another study, from BioProject PRJNA597714 (Xia et al., 2020) reported the unphased sequence of the heterozygous CSS Shuchazao genome. SNP’s were used to construct a phylogenetic tree showing clustering of CSA, CSS and wild tea plants, the latter including ancient Laos tea plants. CaTA sequences from CSA and CSS sequences recovered from PRJNA597714 do not cluster separately, and the CaTA sequences from Laos tea plants sa3445-1, sa3445-2, sa3447-1, sa3447-2, sa3492-1, sa3492-2, and sa3493-un are found in B2, B2, B4, A2b, B2, B4, and A2b respectively.

A further study reported the unphased sequence of an ancient wild tea tree, called DASZ (Zhang et al., 2020a). The CaTA alleles from DASZ1 are found in B5a (ss2402-1 and ss2403-1) and A3 (ss2402-2 and ss2403-2). The same authors also obtained unphased RNA sequences from 217 accessions (BioProject PRJNA595795) and used them for tree construction. They found no distinct separation between ancient trees and cultivars, nor between plants with *sinensis* and *assamica* characteristics. This fits well with our findings. Interestingly, they labeled all their accessions as “*C. sinensis*”. The same group (Zhang et al., 2020b) also obtained a phased genome for CSS cultivar Fudingdabai, using sperm cells. Using this sequence, they established a kinship analysis with a subset of the 217 accessions. Unfortunately, the PRJNA595795 RNA sequences contain very few CaTA reads, preventing assembly and phasing, and further comparison with our CaTA data.

A recent paper (Zhang X. et al., 2021) reported the haplotype-resolved genome of the highly heterozygous CSS cultivar Tieguanyin (TGY). SNP-based trees of 190 accessions were constructed, in which 8 C*. sinensis* accessions clustered in one group, whereas other *Thea* section species clustered in a separate group. When comparing their results with ours (obtained with the same sequence data), we noted important differences. The authors found that *C. tetracocca* and *C. gymnogyna* were very closely related, whereas *C. leptophylla*, *C. angustifolia* and *C. kwangsiensis* formed another highly related group. Based on CaTA comparisons from the same sequences, we found that CaTA from te3467-un, gy3464-1, and gy3464-2 were in groups A2a, A2a and B4 respectively, whereas le3463-1, le3463-2, an3454-1, an3454-2, kw3456-1, kw3456-2 were in B3, A3, B3, B3, B4 and A3. The *C. taliensis* CaTA sequences tl3460-1 and tl-3460-2 were in two different groups, B1 and B5b. Finally, the unique *C. ptilophylla* CaTA allele pt3466-un was found in B3, which also contains sequences from CSS and CSA. These differences in clustering may be due to the use of unphased versus phased sequences and requires further analysis.

### Different mechanisms to generate groups of plants from a single transformant

Our results show that the *Thea* section species are derived from a single *Agrobacterium*-transformed ancestor. The CaTA transformation event seems to predate the origin of section *Thea* because the divergence values for the inverted CaTA repeat (9.7% divergence since insertion in the ancestor) and the maximal divergence values for the two CaTA alleles (7.3%) differ. Using the 9.7% divergence of the CaTA repeat and an estimated universal substitution rate of 6.5x10-9 mutations per site per year (Gaut et al., 1996), the transformation event would have happened 15 Mio years ago. This estimate places the ancestor of the CaTA-carrying plants well before the origin of section *Thea*, 6.7 Mio years ago, and close to the origin of the *Camellia* group, 14.3 Mio years ago (Wu et al., 2022). Interestingly, in the CaTA-less *oleifera* and *sasanqua* species, the sequences around the CaTA insertion site seem to be intact, suggesting that these species split off at an early phase, before the transformation event.

The precise sequence of events leading from the initial transformant to the CaTA-carrying plants is still unknown. A likely scenario is the following. An *R. rhizogenes* bacterium transformed a single cell of a *Camellia*-like ancestor plant, introducing the CaTA insert into chromosome 9. This cell spontaneously regenerated into a fertile R0 plant with one CaTA copy. In order to explain how a single transformed individual gave rise to a large group of descendants, we (Chen et al., 2018; Matveeva, 2021b) and others (Martin-Tanguy et al., 1996) have proposed that strong phenotypic effects of the cT-DNA genes may lead to reproductive isolation, thus generating a new species. Based on experimental evidence, Martin-Tanguy et al. suggested (Martin-Tanguy et al., 1996) that cT-DNA expression could have deleterious effects in the 1-copy state, but lose these in the 2-copy state by silencing. This could lead to rapid selection for phenotypically normal 2-copy plants, and would prevent back-crosses with untransformed plants, as this would restore the one-copy situation, thus favoring speciation. We have proposed an alternative speciation model in which growth effects of the cT-DNA genes would be sufficient to prevent back-crosses to the ancestor plants, but would still allow survival of the transformants. This model was based on the study of the TE-*6b* genes from a *N. otophora* cT-DNA, which cause considerable growth changes in *Arabidopsis* and *N. tabacum* with a delay in flowering time, but do not prevent growth and reproduction of the transformants (Chen et al., 2018; Potuschak et al., 2020).

The CaTA cT-DNA does not carry growth modification genes which could interfere with reproduction. We therefore prefer an third model for this cT-DNA, in which the *acs* and *susL* genes provided initially a selective advantage, which led to fixation of the CaTA sequence. Later the selective advantage was lost (possibly by external changes, like changes in climate or environment), and both genes acquired premature stop codons. Interestingly, the premature stop codon and frameshift mutations in the CaTA *acs* and *susL* genes are common to all *Thea* section sequences, except those from *C. fangchengensis*, which show other types of mutations. We conclude that the CaTA genes were still active at the time of emergence of section *Thea*, but lost their function after the separation of *C. fangchengensis* from the other species, in both groups by independent processes.

The mechanisms by which groups of nGMOs, like *Nicotiana* plants from section *Tomentosa* or *Camellia* plants of section *Thea*, developed from a single transformed cell, will to a large extent determine their overall genetic make-up. On the one extreme, selfing of R0 plants in the case of autogamous species, and rapid selection of 2-copy plants could lead to a strong reduction in genetic diversity. On the other extreme, continued hybridization of transformed plants with untransformed plants, as in the case of allogamous species, would maintain a much larger diversity. Analysis of the variability of other, carefully phased nuclear alleles of the *Camellia* accessions investigated here, might shed more light on these processes.

### Natural transformation and genetic engineering of tea

Our results may be of interest for genetic engineering of tea plants as they demonstrate the natural capacity of *R. rhizogenes* for *Camellia* transformation. Transformation of tea plants with disarmed *A. tumefaciens* vectors has been reported (Mondal et al., 2001; Singh et al., 2015; Lü et al., 2018). *R. rhizogenes* has been used to induce Hairy Roots, but no plants were obtained (Zhang et al., 2006; Rana et al., 2016; Alagarsamy et al., 2018), contrary to what has been reported for other plant species (Tepfer, 1990; Christey, 2001; Lütken et al., 2012). Thus, results are still quite limited, in spite of the potential of genetic engineering for the genetic improvement of tea plants. It would be worthwhile to further optimize HR induction, for example by using special gene constructs allowing inactivation of the HR-inducing genes after induction of transformed hairy roots.

### cT-DNAs and plant phylogeny

Based on the present study, we believe that cT-DNAs have several advantages to explore the origin and evolution of nGMO species. First, cT-DNAs are well-defined, highly specific and recognizable DNA fragments, unrelated to plant sequences. They do not occur in non-transformed ancestors, and their integration at a particular chromosomal site constitutes a founder event which creates a clean start for a new clade. Second, unlike transposable elements, cT-DNA inserts do not amplify and disseminate in the genome after insertion. This avoids the generation of multiple paralogs, which is a significant advantage over classical nuclear markers. Third, cT-DNAs can be sufficiently large and old to generate a range of variants, thereby allowing the construction of detailed trees. Finally, repeat sequences in cT-DNAs can be used to estimate time since transformation, or as relative time markers. Whatever the precise course of events that led to the present-day section *Thea*, it is interesting to realize that the tea plant *Camellia sinensis* and its close relatives are all descendants of a single cell, transformed by the natural engineer *Agrobacterium*.

## Conclusion

The various species of the *Thea* section of *Camellia* are derived from a single plant, transformed by *Agrobacterium*, about 15 Mio years ago, well before the origin of section *Thea*. The descendants of the transformant show various modifications of the introduced cT-DNA, among which insertions of plant sequences and structural rearrangements. The cT-DNA genes are inactive. The distribution of the CaTA sequences among 143 different accessions belonging to 10 species does not correlate with the proposed separation of section *Thea* into different species, but shows that different species share very similar CaTA alleles.

## Data availability statement

The original contributions presented in this study are included in the article/**Supplementary Material**, further inquiries can be directed to the corresponding author.

## Author contributions

LO and TM designed the study. KC provided sequence data. KC, PZ, and LD contributed to data analysis. LO wrote the first draft of the manuscript. LO, TM, KC, PZ, and LD contributed to the interpretation of the results and revised the manuscript. All authors contributed to the article and approved the submitted version.

## Supplementary material

Supplementary Table 1 List of accessions and their indels, indicated by positions of the G240-CaTA. First column: composite numbers referring to species (as provided in the sequence description) and SRX accession number. Other columns: positions of indels according to G240-CaTA sequence, the position is the last nucleotide before the modification. For type of modifications, see **Table 1**.

Supplementary Table 2 Phased alleles of Camellia accessions from section Thea.

Supplementary Table 3 Details for indels.
