# Supplementary material for: Conservation of an Agrobacterium cT-DNA insert in Camellia section Thea reveals the ancient origin of tea plants from a genetically modified ancestor
Source: Front Plant Sci. 2022 Dec 6;13:997762. doi: 10.3389/fpls.2022.997762 (PMC9763466; doi:10.3389/fpls.2022.997762)
Supplement: Supplementary Table 2 — Phased alleles of Camellia accessions from section Thea. [file Table_2.docx]

>ssJACBKZ_Un

GTAACCCCAGATTCTTATCAGCTAAATCTCTTTGCGGGCAGCTCTCTGTGCTTCCAGGAA

TTTTAGCATATCTTATGATGGTTGACGTCCAGCTACAAGCCTCTCCAAATTTCCCTCACC

TTGAAGTATCAATTTGATCTAGAAGCCAATCCAAACTCATTGAAAGGCCTGTCATCAACC

GGTCAACACGGTTCAATGTTTGCTCGCGTGGCGCAGCAATGGTGTGCTAGGCGTTAGGTG

CGCCTGGCTTGGATGGCCCTCAAAGCATTTGAGGCAGTTCGGACCTCGGCAGAGATTTTA

TATTATTTTTTTGTTGTAATCGCGCTTGGCCCACATGCTGCACACTCCGATAGACACGGC

ATTGGCTAAACCCGCTAATTGCGTGGTGATGCGTCCACGTGCTGCGCACTTTCATGCGGA

GTCCCCCCGAGTCCATAGAATAAGAGGGGGACGCGTTTCCTTTTTTCCATCAGCAAACAA

CACCAACTCACTGTGCTAGTATAGTTCATCTGAGAACAGACACACTGCTGAGCAAGCTCT

CCTTCTCCAACTAGCTTTTTCTTTGCTGACATTTCCTACAGAATGGCAGCAAAACAGATT

CCGTTAACACAACTACTGCTCAGGTTGATTTAGAGGCCGCCTTGGCCGAAGCGATCGGGT

CTACATGGAGATTTGCATCAGACTTTCCTCTTAAAATGCAATCGCGAAACACTACCTTGC

CAATTGGCTTATCGCTCGAAAGACTTCAAGCAACCACCAATTGATAGTTCACCGTGAATT

CCATGGGCCACACCTGCCGGAAAACAGCGTAGGGGCAGTCTGCAAATCTCTCAGACTTTT

TAATATGGTCGAGATCAACGTAGTACCGACCAAAGATAAAGATTTCATAGTCGTCCACGA

CTTCAACACAGAAAGAACGACGCAGTTAGGCAATTGAAATTGGTTTGATATGTCGACGAA

AGAGGCTGTTGGCACCCCCCTGGTAGTTCCCATAATGGGCGGTGACTCCCTAAAGAGCGA

GTTCACACCCACCGATGAGAAAGTTGTCAAGCAAGAAACCCTTCTCAAGAATGCCTTTGA

AACAAACAAACAGGCAACCATACGGATTGACGCTCACGAGAGGCAGGCCGCTCCCCTCGT

CGCTTGGCTGAGCCATAGGCCGGCCTATCACGACAAGACAATCGTTATCTTTCATACATA

TGGATACAACAATGGACACGAGTTCGCCTTGGCGGTCGATCAAGCCGGCGCTACAAAAAA

TTGGCGCGAAACTGTAGCGCTAATGCCAAACATTCTGCCGAATGAACTCTCTAGGCTCGC

GAAGCAATATCCACCAAATTGCACGTCGCATTTTTACGACCTCGTTGAGGCCAGCAAGAA

TTGGTTCGCATCTGTCTTCTCCCAGAATATGATGGTGGTCGTCACTTTCCTCAAGATTGC

GGGCACTGGCAAAGGACAGACTAACAAAGTCGTTGATCCAAATGGAAATGAGGTTCTGCA

TCCTAAAGAGAAGGAGGAATATCTCCGCGATTCAGCTTCAATCGAAATCGCGAAATGAAT

CTGCGCCACCCATCCCGGAATCGAGATTGGAGGCGTCACACGAACTTATGCTTATAAGAG

TCGAGGACATCGCTACAAAATCAATCTTTGGACGGGTCAACCGAAGCGGTGGCCGACCCA

CGCGACGAAGTACATCTGGGAAGATTTCGCCAAGCCTGGAAATGCGCTCAGATGGGGTCA

AACGATTATCTCTGATCGCATTGGTGACGACCTTGCAGCTTGTGTGCTTCAAAGCCGTGG

AGAAAATTTTGTCAGCAGTCATGAATATCCTGCTGTTCATGTCGATTAGATGGGCGATCT

GTGTCTTAGTCTAAGCTTCCGCTTAACGCATCTTTGCTACTTTAATGTATTTTTTTAATG

ATATTGTTGGAGTTTTGAGCACATCTTAATCGCAAGTAGATATTTTTTTTGAATCGGATT

CGACTTCAAGCAGATGTACTTCTTTGTCTCTCGATAAATCTAATGACCCTGCGAGCCAGC

CTCTCGCCTTAAACTAATTTCTGAGAGTTAGGTCGGCGCCTCTCGTGGTTTCCATGTTCT

ATGGAAACGGCCGCTCGGGACTTGCCTTCCCGCAACTCATAGAGCTTGCCTGGCCCGCTC

AGGTGGTGGCACAAGCCATCGTTAAATCGTTCGTAATCCTCTACTCTCGCATAAATGAGA

AGGGGAATCCAAAGATGATCACTTTTCAGCTAATTGGAATCTGCAATCCCAAAAGAGGCA

AGCACTATGTGGGGTCCACAACTTATAGAATTTTGGCCTAACCATTCTGCATAATGGATC

ATGACATAGGCCATTTATGGCCGCAAAAACTGGCACAAATTCCAACCTATTCAGCACAAT

GAACAAGTTCTTCCAATTGGAATAAAGGAGGTTAAAATTATTCAAGTAGACATGACGTGC

TGGCACGCCACTAGCCTCGCATTGATGCCACTCTTTCTTGCGCACACTTATATAATGCTG

AGCTAATAATAGAGATAAAAATAGTCAGCTTGTAAACTGGACCTTTATTACTGTCCCTAA

TCTCAAGATCAAAGCGACATATACCTCCTCTCTTTCAAGTCTTTTTGCAACTTGTTCCAA

AGCAAGCGTTCGTGAATCTGCCAGGCAGCCTTCACGAAAATGGCTGATCAACTACGTGAG

GGGCGTTGGTCCGAAGCCAATCTATCATTCGTTGGGATATCCATCACCCCTGAATTCATC

GATGAGGCCCTCAGAGGCTTTTGCCAAAAGAGCCGTTGGCTACAAAGAGGTTTACGTTCT

GGTTGATCGAGATCCTAAAGTTGCCTGGGAAGGAGCGCTCAATGGTTACCTCGGCAAATA

TTCTGGCGTCAAAAATGTTCATTTTGCGCCGAACAACGCCGTGATTGGAAAGCCCCCACG

CCATATCGACATTTTGCTTCGGAAAAAATTTAGCGGCGAATTTCTTGCGACGCTCGAAGG

TGTGGCAATTTCAAACGGTGCAACTGGTTAGGCTTGCGCTCGCATGCTGTAAACCTTCTC

TTGAAGGCAAGACAAGATATTGATGTCTTCTTGTTTGGGGCAGGGAAAAGTCGCCGAGGC

TGTGATTCTTGCCCTCAATCATGGTGCGGCTGCAAAAATCAAAACCATGGCAGTGCTCAG

CCAAGCTCCAATTGAAGGTGGGGTTCCAGCTCGATGCCGTCATTGATCGGAAGGTTATAC

CAAAAGCCAAACTCGTCATAACAGCAACAAATTCCGAGGAGCTCGGTACTCGAGGCCGAC

GAAATTGCGCCAAATGCAGTAACCACATCCCTCGGAAAAGATGAATTGCCCGCCGCCTAC

TTCGATCGCCTTTTGAACGCAGAGGGTCTGATTATTGGCGACGATCTGGATGTGATCGAA

TCGCGCAATGTCAATTCCCTGGTGCTTTACTACTCGAAACGTGATTTGAAACTGACAGAA

CATGGAAGGGATCATTGGATAAAGAACTACGCCAATGTCCTTGCTGATCCAGCTCTCATG

GAGGAACTTAAGACATGAGAGGGGCCAGCCAACTTTTCATCGGTTGGCCTTGCCAGCCTA

GACTTGGCGATGGCCGGCCGACTCTACGAAACTCTTACTGCGAAACTCTCCCACCCCCAG

TAGACAAAACCCTCTTAGGATTGCGCTCTTCAGAAGGTGCGCCTCCCCAGTCACAACCGG

AAAGTCACTTAACTTGGCTCAAGTACCCTACTTAGTTTGTCTCTCTGTCGTGTTTAGTAT

CAGCAACAATAACAAATATTGTAAACTAACATATACTTATATGGTAATTATATGGCATGT

AAGCGTGACAACGATTATGTTTTCCATAGTTTGATAAACTCAACACCCGAAAAAGCCGGT

GACCAAAGGGTTGGGACCTCTTTGCACGAACTCCTATGGCTGCCAGACTATCTACAGAGC

TTTTTCGGAAGCTGACGCAACATCTGATCGCCTACACAATCCTCGCGGACGTGGATGGCG

TGGCTACATGCAAGCAGTTTGACTGAGAGTTTTCGAGTGACTATTTTAAAAGGACAATAT

TTCGTCCCAAGCGATAAAACTCGCTCACATGACTAAGTCGTTGCTTTAATTCAACTGCGT

GAAAAGATTTAAAGGGCAGAACTGTTTGTGTTTCGTATCCAGTTCACCGGCAATTATTGG

CTACGCATGAAGCTCATTATCGTCGATTCTTTACCCAAACACTGATGCCATTCCAAATGA

GTTGGGCCAAAGATGTACTCATGTCGGACAGGCACGATTTGGACTTCGTGATCCTCAAGT

CTATCGCAGTGCTGCTTTATTACAGGCTATGCTTCCACCCTCCATTATTTGGTCAACTGG

ACCTAGCTTTTGACAAGAATAGGCCAGCGTTCATCTATCTATCTTTCAAAAACATGCAGT

TGCTGCTGCGTAAAGGATGTAACATCGGATTTGGTGTGGAGACGGTGCTCGCTACGACTA

TACTGCCTTACTGTAGTGATCTGTCCCGCCAAGCGACGAGGGGAGCGGCCTTGCCTCTCG

TGAGCGTCAATCCATACGGTTGCATGTTCGTTTGTTTCAAAGGCATTCCCAAGAAGGGTT

TCCAGCTTGACAACTTTCTCATCGGTGGGTGTGAACTCGCCGGTTAGTGAGTCACCGCCC

ATTTTGGGAACTACCAGGGGGGTGCCAACGACCTCCTCCGTCGACATCTCAGACCAATTT

CAATTGCCCAACTGCACCGTTCTTTCTATGTTGAAGTCATGGGCGACTATGAAATCTCCA

TCTTTGGTCGGTACTACCTCGATCTCGACCATATTGAAAAGCCTGAGAGATTTGCAAACT

GCCCCTACACTGTTTTCCGGCAAGTGTGGCCCATGGAATCCACGGTGAACTATCAATTGG

CGGTCGCTTGAAGTCTTTCGAGCGATAAGCCAATTGGCGAGGCAGTGTTTCGCGATCGCA

TCTTTAAGAGGAAAGTCCGATGCAAATCTCCATGTAGACCCGATCGTTTCGGCCAAGGCG

GCCTCTAAATCAACTTGAGCAGCAGTTGTGTTAACGGAATCTGTTTTGACTGCCATTCTG

TAAGAAATGTCAGCAAAGGAAAAGCTAGTTAGAGAAGGAGAGCTTGCTCAATAGTGTGTC

TATTCTCTAATGAACTATACTAGCGCAATGAGCTAGGTGTTGTTCGCTGATGGGAAAAAG

GAAATGCATCCCCCTCTTCTACAAAATTCTGTGGGCCCGGAGGGACTCCGTATGAAAGTG

CACAGCATGTGGACGCATCACCATGCAATTAGCGAGCTTAGCCAATGTCGTGTCTGTCAG

AGTGTGCAGCATGTGAGCCAAGCGCAATTACAATAAAAAAATAATATAAAAACTCTGCCT

GAGGTCTGAACTGTCTCAAATGCTTTGAGGGCCATCCAAGCCAAGCGCACCTAACGCCTA

GCACACCATTGTTGCGCCGCTCGAGCATTAATGGTCGGGACCTGGTGTCATCTGGAAACC

TATGAAGCATGGACACGGCAAAATCAGCGCCGTGGCGGTGTCAGACACGAGGGACACGTC

GGGGACACGTACAGGACACGTCATCTGCCGTGTCCCTTTAATTTAATTGTTTTTCTTGTA

GGGTACATGCATGTCCAGATCTGGACACGGCGTGAGACACGGCAAGG

>ss0652_Un

TCACCTTGAAGTATCAATTTGATCTAGAAGCCAATCCAAACTCATTGAAAGGCCTGTCAT

CAACCGGTCAACACGGTTCAATGTTTGCTTGCGTGGCGTAGCAATGGTGTGCTAGGCGTT

AGGTGCKCCTGGCTTGGATGGCCCTCAAAGCATTTGAGGCAGTTCGGACCTCGGCAGAGA

TTTTATATTATTTTTTTGTTGTAATCGCGCTTGGCCCACATGCTGCACACTCCGATAGAC

ACGGCATTGGCTAAACCCGCTAATTGCGTGGTGATGCGTCCACGTGCTGCGCACTTTCAT

GCGGAGTCCCCCCGAGTCCATAGAATAAGAGGGGGACGCGTTTCCTTTTTCCCATCAGCA

AACAACACCAACTCACTGTGCTAGTATAGTTCATCTGAGAACAGACACACTGCTGAGCAA

GCTCTCCTTCTCCAACTAGCTTTTTCTTTGCTGACATTTCCTACAGAATGGCAGCAAAAC

AGATTCCGTTAACACAACTACTGCTCAGGTTGATTTAGAGGCCGCCTTGGCCGAAGCGAT

CGAGTCTACATGGAGATTTGCATCAGACTTTCCTCTTAAAAATGCAATCGCGAAACATTG

CCTCGCCAATTGGCTTATCGCTCGAAAGACTTCAAGCAACCACCAATTGATAGTTCACCG

TGAATTCCATGGGCCACACCTGCCGGAAAACAGCGTAGGGGCAGTCTGCAAATCTCTCAG

ACTTTTTAATATGGTCGAGATCAACGTAGTACCGACCAAAGATAAAGATTTCATAGTCGT

CCACGACTTCAACACAGAAAGAACGACGCAGTTAGGCAATTGAAATTGGTTTGATATGTC

GACGAAAGAGGCTGTTGGCACCCCCTTGGTAGTTCCCATAATGGGCGGTGACTCCCTAAC

CAGCGAGTTCACACCCACCGATGAGAAAGTTGTCAAGCAAGAAACCCTTCTCAAGAATGC

CTTTGAAACAAACAAACAGGCAACCATACGGATTGACGCTCACGAGAGGCAGGCCGCTCC

CCTCGTCGCTTGGCTGAGCCATAGGCCGGCCTATCACGACAAGACAATCGTTATCTTTCA

TACATATGGATACAAAAATGGACACGAGTTCGCCTTGGCGGTCAATCAAGCCAGCGCTAC

AAAAAATTGGCGCGAAACTGTAGCGCTAATGCCAAACATTCTGCCGAATGAACTCTCTAG

GCTCGCGAAGCAATATCCACCAAATTGCACGTCGCATTTTTACGACCTCGTTGAGGCCAG

CAAGAATTGGTTCGCATCTGTCTTCTCCCAGAATATGATGGTGGTCGTCACTTTCCTCAA

GATTGCGGGCACTGGCGAAGGACAGACTAACAAAGTCGTTGATCCAAATGGAAATGAGGT

TCTGCATCCTAAAGAGAAGGAGGAATATCTCCGCGATTCAGCTTCAATCGAAATCGCGAA

ATGAATCTGCGCCACCCATCCCGGAATCGAGATTAGAGGCGTCACACGAACTTATGCTTA

TAAGAGTCGAGGACATCGCTACAAAATCAATCTTTGGACGGGTCAACCGAAGCGGTGGCC

GACCCACGCGACGAAGTACATCTGGGAAGATTTCGCCAAGCCTGGAAATGCGCTCAGATG

GGGTCAAACGATTATCTCTGATCGCATTGGTGACGACCTTGCAGCTTGTGTGCTTCAAAG

CCGTGGAGAAAATTTTGTCAGCAGTCATGAATATCCTGCTGTTCATGTCGATTAGATGGG

CGATCTGTGTCTTAGTCTAAGCTTCCGCTTAACGCATCTTTGCTACTTTAATGTATTTTT

TTAATGATATTGTTGGAGTTTTGAGCACATCTTAATCGCAAGTAGATATTTTTTTTGAAT

CGGATTCGACTTCAAGCAGATGTACTTCTTTGTCTCTCGATAAATCTAATGACCCTGCGA

GCCGGCCTCTCACCTTAAACTAATTTCTGAGAGTTAGGTCGGCGCCTCTCGTGGTTTCCA

TGTTCTATGGAAACGGCCGCTCGGGACTTGCCTTCCCGCAACTCATCGAGCTCTCGGGTG

GTGGCACAAGCCATCGTTAAATCGTTCGTAATCCTCTACTCTCGCATAAATGAGAGGGGA

ATCCAAAGATGATCACTTTTCAGCTAATTGGAATCTGCAATCCCAAAAGAGGCAAGCACT

ATGTGGGGTCCACAACTTATAGAATTTTGGCCTAACCATTCTGCATAATGGATCATGACA

TGGGCCATTTATGGCCACAAAAACTGGCACAAATTCCAACCTATTCAGCACAATGAACAA

GTTCTTCCAATTGGAATAAAGGAGGTTAAAATTATTCGAGTAGACATGACGTGCTGGCAC

GCCACTAGCCTCGCATTGATGCCACTCTTTCTTGCGCACACTTATATAATGCTGAGCTAA

TAATAGAGATAAAAATAGTCAGCTTGTAAACTGGACCTTTATTATTGCCCCTAATCTCAA

GATCAAAGCGACATATACCTCTTCTCTTTCAAGTCTTTTTGCAACTTGTTCCAAAGCAAG

CGTTTGTGAATCTGCCAGGCAGCCTTCACGAAAATGGCTGATCAACTGTGTGAGGGGCGT

TGGTCCGAAGCCAATCTATCATTCGTTGGGATATCCATCACCCCTGAATTCATCGATGAG

GCCCTCRGAGKTTTTTGGGAGTCTTTTGCCAAAAGAGCCGTTGGCTACAAAGAGGTTTAC

GTTCAGGTTGATCGAGATCCTAAAGTTGCCTGGGAAGGAGCGCTCAATGGTTACCTCGGC

AAATATTCTGGCGTAAAAAATGTTCATTTTGCGCCGAACAACGCCACGATTGGAAAGCCC

CCACGCCATATCGACATTTTGCTTCGGAAAAAATTTAGCGGCGAACTTCTTGCAACGCTC

GAAGGTGTGGCAATTTCAAACGGTGCAACTGGTTGGCTTGCGCTCRCATGTGTAAACCTT

CTCTTGAAGGCAAGACAAGATATTGATGTCTTCTTGTTTGGGGCAGGAAAAGTCACCGAG

GCTGTGATTCTTGCCCTCAATCATGGTGCGGCTGCAAAAATCAAAACCATGGCAGTGCTC

AGCCAAGCTCCAATTGAAGGTGGGGTTCCAGCTCGATGCCATCATTGATCGGAAGGTTAT

ACCAAAAGCCAAACTCGTCATAATAGCAACAAATTCTGAGGAGCTCGGTACTCGAGGCCG

ACGAAATTGCGCCAAATGCAGTAACCATATCCCTCGGAAAAGATGAATTGCCCGCCGCCT

ACTTCGATCGCCTTTTGAACGCAGAGGGTCTGATTATTGGCGACGATCTGGATGCGATCG

AATCGCGCAATGTCAATTCCCTGGTGCTTTACTACTCGAAACGTGATTTGAAGCTGACAG

AACATGGACGGGATCATTGGATAAAGAACTACGCCAATGTCCTTGCTGATCCAGCTCTCA

TGGAGGAACTTAAGACATGAGAGGGGCCAGCCAACTTTTCATCGGTTGGCCTTGCCAGCC

TAGACTTGGCGATGGCCGGCCGACTCTACGAAACTCTTACTGCGAAACTCTCCCACCCCC

AGTAGACAAAACCCTCTTAGGATTGCGCTCTTCAGAAGGTGCGCCTCCCCAGTCACAACC

GGAAAGTCACTCAACTTGGCTCAAGTACCCTACTTAGTTTGTCTCTCTGTTGTGTTTAGT

ATCAGCAACAATAACAAATATTGTAAATAACATATATTTATATGGTAATTATATGGCATG

TAAGCGTGACAACAATTATGTTTTCCATAGTTTGATAAACTCAACACCCGAAAAAGCCGG

TGACCAAAGGGTTGGGACCTCTTTGCACGAACTCCTATGGCTGCCAAACTATCTACAGAG

CTTTGACGCAGGAGCTTTGACGCAGCATCTGATCGCCACACAATCCTCGCGGACGTRGAT

GGCGTGGCTACATGCAAGCAGTTTGACTGAGAGTTTTCGAGTGACTATTTTAAAAGGACA

ATATTTCGTCCCAAGCGATAAAACTCGCTCACATGACTAAGTCGTTGCTTTAATTCAACT

GCGTGAAAAGATTTAAAGGGCAGAACTGTTTGTGTTTCGTATCCAGTTCACCGGCAATTA

TTGGCTACGCATGAAGCTCATTATCGTCGATTCTTTACCCAAACACTGATGCCATTCCAA

ATGAGTTGGGCCAAAGATGTACTCATGTCGGACAGGCGCGATTTGGACTTCGTGATCCTC

AAGTCTATCGCAGTGCTGCTTTATTACAGGCTATGCTTCCACCCTCCATTATTTGGTCAA

CTGGACCTAGCTTTTGACAAGAATAGGCCAGCGTTCATCTATCTATCTTTCAAAAACATG

CAGTTGCTGCTGCGTAAAGGATGTAACATCGGATTTGGTGTGGAGACGGTGCTCGCTACA

ACTATACTGCCTTACTGTAGTGATCTGTCCCGCCAAGCGATGAGGGGAGCGGCCTGCCTC

TCGTGAGCGTCAATCCATACGGTTGCATGTTTGTTTGTTTCAAAGGCATTCCCAAGAAGG

GTTTCCAGCTTGACAACTTTCTCATCGGTGGGTGTGAACTCGCCGGTTAGTGAGTCACCG

CCCATTTTGGGAACTACCAGGGGGGTGCCAACGACTTCCTCCGTCGACATCTCAGACCAA

TTTCAATTGCCCAACTGCACCGTTCTTTCTATGTTGAAGTCATGGGCGACTATGAAATCT

CCATCTTTGGTCGGTACTACCTCGATCTCGACCATATTGAAAAGCCTGAAAGATTTGCAA

ACTGCCCCTACACTGTTTTCCGGCAAGTGTGGCCCATGGAATCCACGGTGAACTATCAAT

TGGCGGTCGCTTGAAGTCTTTCGAGCGATAAGCCAATTGGCGAGGCAGTGTTTCGCGATC

GCATCTTTAAGAGGAAAGTCCGATGCAAATCTCCATGTAGACCCAGTCGTTTCGGCCAAG

GCGGCCTCTAAATCAACTTGAGCAGCAGTTGTGTTAACGGAATCTGTTTTGACTGCCATT

CTGTAAGAAATGTCAGCAAAGGAAAAGCTAGTTAGAGAAGGAGAGCTTGCTCAATAGTGT

GTCTATTCTCAAATGAACTATACTAGCGCAATGAGTTGGTGTTGTTCGCTGATGGAAAAA

AGGAAACGCATCCCCCTCTTCTACAAAATTCTGTGGGCCCGGAGGGACTCCGTATGAAAG

TGCACAGCATGTGGACGCATCACCATGCAATTAGCAAGCTTAGCCAATGTCGTGTCTGTC

AGAGTGTGCAGCATGTGAGCCAAGCGCAATTACAATAAAAAAATAATATAAAAACTCTGC

CGAGGTCCGAACCGTCTCAAATGCTTTGAGGGCCATCCAAGCCAAGCGCACCTAACGCCT

AGCACACCATTGTTGCGCCGCTCGAGCATTAATGGTTCGGGACCTGGTGTCATCTGGAAA

CCTATGAAGCATGGACACGGCAAAATCAGCGTCGTGGCGGTGTCAGACACGAGGACACGT

C

>ss0648_2

ttcccTCACCTTGAAGTATCAATTTGATCTAGAAGCCAATCCAAACTCATTGAAAGGCCT

GTCATCAACCTGTCAACACGGTTCAATGTTTGCTCGAGTGGCGCAGCAATGGTGTGCTAG

GCGTTAGGTGCGCCTGGCTTGGATAGCCCTCAAAGCATTTGAGACAGTTCGGACCTCGGC

AGAGATTTTATATTATTTTTTTGTTGTAATCGTGCTTGGCCCACATGCTGCACACTCCGA

CAGACACGGCATTGACTAAACCCGCTAATTGCGTGGTGATGCGTCCACGTGCTGCGCACT

TTCATGCGGAGTCCCCCCGAGCCCGTAGAATTTTGTAGAAGAGGGGGACGCGTTTTCTTT

TTCCCATCAGCAAACAACACCAACTCACTGTGCAAGTATAGTTCATCTGAGAACAGACAC

ACTGCTGAGCAAGCTCTCCTTCTCCAACTAGCTTTTTCTTTGCTGACATTTCCTACAGAA

TGGCAGCAAAACAGATTCCGTTAACACAACTGCTGCTCAGGTTGATTTAGAGGCCGCCTT

GGCCGAAGCGACCGGGTCTACATGGAGATTTGCATCAGACTTTCCTCTTAAAGATGCGAT

CGCGAAACACTGCCTCGCCAATTGGCTTATCGCTCGAAAGACTTCAAGCAACCACCAATT

GATAGTTCACCGTGAATTTCATGGGCCACACCTGCCGGAAAACAGCGTAGGGGCAGTCTG

CAAATCTCTCAGACTTTTTAATATGGTCGAGATCAACGTAGTACCGACCAAAGATAGAGA

TTTCATAGTCGTCCACGACTTCAACACAGAAAGAACGACGCAGTTAGGCAATTGAAATTG

GTCTGATATGTCGACGAAAGAGGCCGTTGGCACCCCCCTGGTAGTTCCCATAATGGGCGG

TGACTCCCTAACTAGCGAGTTCACACCCACCGATGAGAAAGTTGTCAAGCAAGAAACCCT

TCTCAGGAATGCCTTTGAAACAAACAAACAGGCAACCATACGGATTGACGCTCACGAGAG

GCAGGCCGCTCCCCTCGTCGCTTGGCTGAGCCATAGGCCGGCCTATCACGACAAGACAAT

CGTTATCTTTCATACATATAGATACAACAATGGACACGAGTTCGCCTTGGCGGTCGATCA

AGCCGGCGCTACAGAAAATTGGCACGAAACTGTAGCGCTAATGCCAAGCATTCTGCCGAA

TGAACTCTCTAGGCTCGCGAAGCAATATCCACCAAATCGCACGTCGCATTTTTACGACCT

CGTTGAGGCCGGCAAGAATTGGTTCGCGTCTGTCTTCTCCCAGAATATGATGGTGGTCGT

CACTTTCCTCAAGATTGCGGGCACTGGCGAAGGACAGACTAACAAAGTCGTTGATCCAAa

tggaaatgaggttctgcatcctaaagagaaggaggaatatcTCCACGATTCAGCTTCAAT

CAAAATCGTGAAATGAATCTGCGCCACCCATCCCGGAATCGAGATTGGAGGCGTCACACG

AACTTATGCTTATAAGAGTCGAGGACATCGCTACAAAATCGATCTTTGGACGGGTCAACC

GAAGCGGTGGCCGACCCACGCGACGAAGTATATCTGGGAAGATTTCGCCAAGCCTGGAAA

TGCGCTCAGATGGGGTCAAACGATTATCTCTGATCGCATTGGCGACGACCTTGCAGCTTG

TGTGCTTCAAAGCCGTTGAGAAAATTTTGTCAGCAGTCATGAATATCCCGCTGTTCATGT

CGATTAGATAGGCGATCTGTGTCTTAGTCTAAGCTTCCGTTTAACGCATCTTTGCTACTT

TAATGTATTTTTTTAATGATATTGTTGGAGTTTTCAGCACATCTTAATCGCAACTAGATA

TTTTTTTTTGAATCGGATTCGACTTCAAGTAGATGTACTTCTTTGTCTCTCGATAAATCT

AATGACCCTGCGAGCCGGCCTCTCGCCTTAAACTAATTTCTGAGAGTTAGGTCGGCGCCT

CTCGTGGTTTCCATGTTCTATGGAAACGGCCTCTCGGGACTTGCCTTCCCGCAACTCATC

GAGCTTGCCTGGCCCGCTTGGGTGGTGGCGCAAGCCATCGTTAAATCGTTCGTAATCCTC

TACTCTCGCATAAATGAGAGGGGAATCCAAAGATGATCACTTTTCAGCTAATTGGAATCT

GTAATCCCAAAAGAGGCAAGCACTATGTGGGGTCCACAACTTATAGAATTTTGGCCTAAC

CATTCTGCATAATGGATCATGACATGGGCCATTTATGGCCGCAAAAACTGGCACAAATTC

CAACCTGTTCAGCACAATGAACAAGTTCTTCCAATTGGAATAAAGGAGGTTAAAATTATT

CGAGTAGACATGACGTGCCGGCACGCCACTAGCCTCGCATTGATGCCACCCTTTCTTGCG

CACACTTATATAATGCTGAGCTAATAATAGAGATAAAAATAGTCAGCTTGTAAACTGGAC

CTTTACTACTGCCCCTAATCTCAAGATCAAAGCGACATATACCTCCTCTCTTTCAAGTCT

TTTTGCAACTTGTTCCAAAGCAAGCGTTCATGAATCTGCTAGGCAACCTTCACGAAAATG

GCTGATCAACTATGTGAGGGGCGTTGGTCCGAAGCCGATCTATCATTCATTGGGATATCC

ATCACCCCTGAATTCATCGATGAGGCCCTCAGAGGCTTTTGGGAGTCTTTTGCCAAAAGA

GCCGTTGGCTACAAAGAGGTTTACGTTCAGGTTGATCGAGATCCTAAAGTTGTCTGGGAA

GGAGCGCTCAATGGTTACCTCGGCAAATATTCTGGCGTCAAAAATGTTCATTTTGCGCCG

AACAACGCCGCGATTGGAAAGCCCCCACGCCATATCGACATTTTGCTTCGGAAAAAATTT

AGCAGCGAACTTCTTGCGACGCTCGAAGGTGTGGCAATTTCAAACGGTGCAACTGGTTGG

CTTGCGCTCGCATGCGTAAACCTTCTCTTGAAGGCAAGACAAGATATTGATGTCTTCTTG

TTTGGGGCGGGAAAAGTCGCCGAGGCTGTGATTCTTGCCCTCAATCATGGTGTGGCTGCA

AAAATCAAAACCATGGCAGTGCTCAGCCAAGCTCCAATTGAAGGTGGGGTTCCAGCTCGA

TGTCGTCATTGATCAGAAGGTTATACCAAAAGCCAAACTCGTCATAACAGCAACAAATTC

CGAGGAGCTCGGTACTTGAGGCCGACGAAATTGCGCCAAATGCAGTAACCATATCCCTCG

GAAAAGATGAATTGCCCGTCGCCTACTTCGATCGCCTTTTGAACGCAGAGGGTCTGATTA

TCGGCGACGATCTGGATGCGATCGAATCATGCAATGCCAATTCCCTGGTGCTTTACTACT

CGAAACGTGATTTGAAGCTGACAGAACATAGAAGGGATCATTGGATAAAGAACTACGCCA

ATGTCCTTGCTGATCCAGCTCTCATGGAGGAACTTAAGACATGGGAGGGGCCAGCCAACT

TTTCATCGGTTGGCCTTGCCAGCCTAGACTTGGCGATGGCCGGCCGACTCTACGAAACTC

TTACTGCGAAACTCTCCCACCCCCAGTAGACAAGACCCTCTTAGGATTGCGCTCTTCAGA

AGGTGCGCCTCCCCAGTCACAACCGGAAAGTCACTCAACTTGGCTCAAGTACCCTACTTA

GTTTGTCTCTCTGTCGTGTTTAGTATCAGCAACAATAACAAATATTGTAAATAACATATA

TTTATATGGTAATTATATGGCATGTAAGCGTGACAACGATTATGTTTTCCATAGTTTGAT

AAACTCAACACCCGAAAAAGCCGGTGACCAAAGGGTTAGGACCTCTTTGCACGAACTCCT

ATGGCTGCCAGACTATCTACAGAGCTTTTCGGAAGCTGACGCAGCATCTGATCGCCACAC

AATCCTCGCGGACGTGGATGGCGTGGCTACACGCAAGCAGTTTGACTGAGAGTTTTTGAG

TGACTATTTTAAAAGGACAATATTTCGTCCCAAGCGATAAAACTTGCTCACATGACTAAG

TCGTTGCTTTAATTCAGCTGCGTGAAAAGATTTAAAGGGCAGAACTGTTTGTGTTTCGTA

TCCAGTTCACCTGCAATTATTGGCTACGCATGAAGCTCATTATCGTCGATTCTTTACCAA

AACACTGATGCCATTCCAAATGAGTTGGGCCAAAGATGTACTCATGTCGGACAGGTGCGA

TTTGGACTTCGTGATCCTCAAGTCTATCGCAGTGCTGCTTtattacaggctatgcttcca

ccctccattatttggtcaactggacctagcttttgacaagaataggccagcgttcatcta

tctatctttcaaaaacatgcagttgctgctgcgtaaaggatgtaacatcGGATTTGGTGT

GGAGACGGTGCTCACTACGACTATACTGCCTTACTGTAGTGATCTGTCCCGCCAAGCGAC

GAGGGGAGCGGCCTGCCTCTCGTGAGCGTCAATCCATATGGTTGCATGTTCGTTTGTTTC

AAAGGCATTCCCAAGAAGGGTTTCTAGCTTGACAACTTTCTCATCGGTAGGTGTAAACTC

GCCGGTTAGTGAGTCACTGCCCATTTTGGGAACTACCAAGGGGGTGCCAACGACCTCCTC

CGTCGACATCTCAGACCAATTTCAATTGCCCAACTGCGCCGTTCTTTCTATGTTGAAGTC

ATGGGCGACTATGAAATCTCCATCTTTGGTCAGTACTACCTCGATCTCAACTATATTGAA

AAGCCTGAGAGATTTGCAAACTGCCCTACACTGTTTTCCGGCAAGTGTGGCCCATGGAAT

CCACGGTGAACTATCAATTGGCGGTCGCTTGAAGTCTTTCGAGCGATAAGCCAATTGGCG

AGGCAGTGTTTCGCGATCGCATCTTTAAGAGGAAAGTCCGATGCAAATCTCCATGTAGAC

CCGGTCGCTTCGGCCAAGACGGCCTCTAAATCAACTTGAGCAGAAGTTGTGTTAAGGGAA

TCTGTTTTgactgccattctgtaagaaatgtcagcaaaggaaaagctagttagaGAATGA

GAGCTTGCTCAATAGTGTGTCTATTCTCAGATGAACTATACTAGCGCAATGAGTTGGTGT

TGTTCGCTGATGGGAAAAAGGAAACGCATCCCCCTCTTCTACAAAATTCTATGGGCCCGG

AGGGACTCTGTATGAAAGTGCACAGCATGTGGACGCATTACCAtgcaattagcaagctta

gccaatgtcgtgtctgtcagagtgtgcAGCATGTGGGCCAAGCGCAATTACAATAAAAAA

ATAATATAAAAACTTTGCCAAGGTCCGAACCGTCTCAAATGCTTTGAGGGCCATCCAAGC

CAAGCGCACCTAACGCCTAGCACACCATTGTTGCGCCGCTCGAGCATTAATGGTCCGGGA

CCTGGTGTCatctggaaacctatgaagcatggacacggcaaaatcag

>ss0648_1

cacctTGAAGTATCAATTTGATCTAGAAGCCAATCCAAACTCATTGAAAGGCCTGTCATC

AACCGGTCAACACGGTTCAATGTTTGCTCGCGTGGCGCAGCAATGGTGTGCTAGGCGTTA

GGTGCGCCTGGCTTGGATGGCCCTCAAAGCATTTGAGGCAGTTCGGACCTCGGCAGAGAT

TTTATATTATTTTTTTGTTGTAATCGCGCTTGGCCCACATGCTGCACACTCCGATAGACA

CGGCATTGGCTAAACCCGCTAATTGCGTGGTGATGCGTCCACGTGCTGCGCACTTTCATG

CGGAGTCCCCCCGAGTCCATAGAATAAGAGGGGGACGCGTTTCCTTTTTCCCATCAGCAA

ACAACACCAACTCACTGTGCTAGTATAGTTCATCTGAGAACAGACACACTGCTGAGCAAG

CTCTCTTTCTCCAACTAGCTTTTTCTTTGCTGACATTTCCTACAGAATGGCAGCAAAACA

GATTCCGTTAACACAACTACTGCTCAGGTTGATTTAGAGGCCGCCTTGGCCGAAGCGATC

GAGTCTACATGGAGATTTGCATCAGACTTTCCTCTTAAAAATGCAATCGCGAAACACTGC

CTCGCCAATTGGCTTATCGCTCGAAAGACTTCAAGCAACCACCAATTGATAGTTCACCGT

GAATTCCATGGGCCACACCTGCCGGAAAACAGCGTAGGGGCAGTCTGCAAATCTCTCAGA

CTTTTTAATATGGTCGAGATCAACGTAGTACCGACCAAAGATAAAGATTTCATAGTCGTC

CACGACTTCAACACAGAAAGAACGACGCAGTTAGGCAATTGAAATTGGTTTGATATGTCG

ACGAAAGAGGCTGTTGGCACCCCCTTGGTAGTTCCCATAATGGGCGGTGACTCCCTAACC

AGCGAGTTCACACCCACCGATGAGAAAGTTGTCAAGCAAGAAACCCTTCTCAAGAATGCC

TTTGAAACAAACAAACAGGCAACCATACGGATTGACGCTCACGAGAGGCAGGCCGCTCCC

CTCGTCGCTTGGCTGAGCCATAGGCCGGCCTATCACGACAAGACAATCGTTATCTTTCAT

ACATATGGATACAAAAATGGACACGAGTTCGCCTTGGCGGTCAATCAAGCCGGCGCTACA

AAAAATTGGCGCGAAACTGTAGCGCTAATGCCAAACATTCTGCCGAATGAACTCTCTAGG

CTCGCGAAGCAATATCCACCAAATTGCACGTCGCATTTTTACGACCTCGTTGAGGCCAGC

AAGAATTGGTTCGCATCTGTCTTCTCCCAGAATATGATGGTGGTCGTCACTTTCCTCAAG

ATTGCGGGCACTGGCGAAGGACAGACTAACAAAGTCGTTGATCCAAATGGAAATGAGGTT

CTGCATCCTAAAGAGAAGGAGGAATATCTCCGCGATTCAGCTTCAATCGAAATCGCGAAA

TGAATCTGCACCACCCATCCCGGAATCGAGATTGGAGGCGTCACACGAACTTATGCTTAT

AAGAGTCGAGGACATCGCTACAAAATCAATCTTTGGACGGGTCAACCGAAGCGGTGGCCG

ACCCACGCGACGAAGTACATCTGGGAAGATTTCGCCAAGCCTGGAAATGCGCTCAGATGG

GGTCAAACGATTATCTCTGATCGCATTGGTGACGACCTTGCAGCTTGTGTGCTTCAAAGC

CGTGGAGAAAATTTTGTCAGCAGTCATGAATATCCTGCTGTTCATGTCGATTAGATGGGC

GATCTGTGTCTTAGTCTAAGCTTCCGCTTAACGCATCTTTGCTACTTTAATGTATTTTTT

TAATGATATTGTTGGAGTTTTGAGCACATCTTAATCGCAAGTAGATATTTTTTTTGAATC

GGATTCGACTTCAAGCAGATGTACTTCTTTGTCTCTCGATAAATCTAATGACCCTGCGAG

CCGGCCTCTCGCCTTAAACTAATTTCTGAGAGTTAGGTCGGCGCCTCTCGTGGTTTCCAT

GTTCTATGGAAACGGCCGCTCGGGACTTGCCTTCCCGCAACTCATCGAGCTTGCCTGGCC

CGCTCGGGTGGTGGCACAAGCCATCGTTAAATCGTTCGTAATCCTCTACTCTCGCATAAA

TGAGAGGGGAATCCAAAGATGATCACTTTTCAGCTAATTGGAATCTGCAATCCCAAAAGA

GGCAAGCACTATGTGGGGTCCACAACTTATAGAATTTTGGCCTAACCATTCTGCATAATG

GATCATGACATGGGCCATTTATGGCCACAAAAACTGGCACAAATTCCAACCTATTCAGCA

CAATGAACAAGTTCTTCCAATTGGAATAAAGGAGGTTAAAATTATTCGAGTAGACATGAC

GTGCTGGCACGCCACTAGCCTCGCATTGATGCCACTCTTTCTTGCGCACACTTATATAAT

GCTGAGCTAATAATAGAGATAAAAATAGTCAGCTTGTAAACTGGACCTTTATTACTGCCC

CTAATCTCAAGATCAAAGCGACATATACCTCTTCTCTTTCAAGTCTTTTTGCAACTTGTT

CCAAAGCAAGCGTTTGTGAATCTGCCAGGCAGCCTTCACGAAAATGGCTGATCAACTGTG

TGAGGGGCGTTGGTCCGAAGCCAATCTATCATTCGTTGGGATATCCATCACCCCTGAATT

CATCGATGAGGCCCTCAGAGGTTTTTGGGAGTCTTTTGCCAAAAGAGCCGTTGGCTACAA

AGAGGTTTACGTTCAGGTTGATCGAGATCCTAAAGTTGCCTGGGAAGGAGCGCTCAATGG

TTACCTCGGCAAATATTCTGGCGTAAAAAATGTTCATTTTGCGCCGAACAACGCCACGAT

TGGAAAGCCCCCACGCCATATCGACATTTTGCTTCGGAAAAAATTTAGCGGCGAACTTCT

TGCAACGCTCGAAGGTGTGGCAATTTCAAACGGTGCAACTGGTTGGCTTGCGCTCGCATG

TGTAAACCTTCTCTTGAAGGCAAGACAAGATATTGATGTCTTCTTGTTTGGGGCAGGAAA

AGTCACCGAGGCTGTGATTCTTGCCCTCAATCATGGTGCGGCTGCAAAAATCAAAACCAT

GGCAGTGCTCAACCAAGCTCCAATTGAAGGTGGGGTTCCAGCTCGATGCCATCATTGATC

GGAAGGTTATACCAAAAGCCAAACTCGTCATAATAGCAACAAATTCTGAGGAGCTCGGTA

CTCGAGGCCGACGAAATTGCGCCAAATGCAGTAACCATATCCCTCGGAAAAGATGAATTG

CCCGCCGCCTACTTCGATCGCCTTTTGAACGCAGAGGGTCTGATTATTGGCGACGATCTG

GATGCGATCGAATCGCGCAATGTCAATTCCCTGGTGCTTTACTACTCAAAACGTGATTTG

AAGCTGACAGAACATGGAAGGGATCATTGGATAAAGAACTACGCcAATGTCCTTgctgat

cCagctctcaTGGAGGAACTTAAGACATGAGAGGGGCCAGCCAACTTTTCATCGGTTGGC

CTTGCCAGCCTAGACTTGGCGATGGCCGGCCGACTCTACGAAACTCTTACTGCGAAACTC

TCCCACCCCCAGTAGACAAAACCCTCTTAGGATTGCGCTCTTCAGAAGGTGCGCCTCCCC

AGTCACAACCGGAAAGTCACTCAACTTGGCTCAAGTACCCTACTTAGTTTGTCTCTCTGT

TGTGTTTAGTATCAGCAACAATAACAAATATTGTAAATAACATATATTTATATGGTAATT

ATATGGCATGTAAGCGTGACAACGATTATGTTTTCCATAGTTTGATAAACTCAACACCCG

AAAAAGCCGGTGACCAAAGGGTTGGGACCTCTTTGCACGAACTCCTATGGCTGCCAAACT

ATCTACAGAGCTTTGACGCAGCATCTGATCGCCACACAATCCTCGCGGACGTGGATGGCG

TGGCTACATGCAAGCAGTTTGACTGAGAGTTTTCGAGTGACTATTTTAAAAGGacaatat

ttcgtcccaagcgataaaacttgctcacatgactaagtcgttgctttaattcagctgcgt

gaaaagatttaaagggcagaactgtttgtgtttcgtatccagttcacctgcaattattgg

ctacgcatgaAGCTCATTATCGTCGATTCTTTACCCAAACACTGATGCCATTCCAAATGA

GTTGGGCCAAAGATGTACTCATGTCGGACAGGCGCGATTTGGACTTCGTGATCCTCAAGT

CTATCGCAGTGCTGCTTTATTACAGGCTATGCTTCCACCCTCCATTATTTGGTCAACTGG

ACCTAGCTTTTGACAAGAATAGGCCAGCGTTCATCTATCTATCTTTCAAAAACATGCAGT

TGCTGCTGCGTAAAGGATGTAACATCGGATTTGGTGTGGAGACGGTGCTCGCTACGACTA

TACTGCCTTACTGTAGTGATCTGTCCCGCCAAGCGATGAGGGGAGCGGCCTGCCTCTCGT

GAGCGTCAATCCATACGGTTGCATGTTCGTTTGTTTCAAAGGCATTCCCAAGAAGGGTTT

CCAGCTTGACAACTTTCTCATCGGTGGGTGTGAACTCGCCGGTTAGTGAGTCACCGCCCA

TTTTGGGAACTACCAGGGGGGTGCCAACGACTTCCTCCGTCGACATCTCAGACCAATTTC

AATTGCCCAACTGCACCGTTCTTTCTATGTTGAAGTCATGGGCGACTATGAAATCTCCAT

CTTTGGTCGGTACTACCTCGATCTCGACCATATTGAAAAGCCTGAAAGATTTGCAAACTG

CCCCTACACTGTTTTCCGGCAAGTGTGGCCCATGGAATCCACGGTGAACTATCAATTGGC

GGTCGCTTGAAGTCTTTCGAGCGATAAGCCAATTGGCGAGGCAGTGTTTCGCGATCGCAT

CTTTAAGAGGAAAGTCCGATGCAAATCTCCATGTAGACCCAGTCGTTTCGGCCAAGGCGG

CCTCTAAATCAACTTGAGCAGCAGTTGTGTTAACGGAATCTGTTTTGACTGCCATTCTGT

AAGAAATGTCAGCAAAGGAAAAGCTAGTTAGAGAAGGAGAGCTTGCTCAATAGTGTGTCT

ATTCTCAAATGAACTATACTAGCGCAATGAGTTGGTGTTGTTCGCTGATGGAAAAAAGGA

AACGCATCCCCCTCTTCTACAAAATTCTGTGGGCCCGGAGGGACTCCGTATGAAAGTGCA

CAGCATGTGGACGCATCACCATGCAATTAGCAAGCTTAGCCAATGTCGTGTCTGTCAGAG

TGTGCAGCATGTGAGCCAAGCGCAATTACAATAAAAAAATAATATAAAAACTCTGCCGAG

GTCCGAACCGTCTCAAATGCTTTGAGGGCCATCCAAGCCAAGCGCACCTAACGCCTAGCA

CACCATTGTTGCGCCGCTCGAGCATTAATGGTTCGGGACCTGGTGTCATCTGGAAACCTA

TGAAGCATGGACACGGCAAAATCAGCGCCGTGGCGGTGTCAGACACGAGGACACGTcggg

gacacgtacaggacac

>ss0644_Un

CTCTCCAAATTTCCCTCACCTTGAAGTATCAATTTGATCTAGAAGCCAATCCAAACTCAT

TGAAAGGCCTGTCATCAACCGGTCAACACGGTTCAATGTTTGCTTGCGTGGCGCAGCAAT

GGTGTGCTAGGCGTTAGGTGCGCCTGGCTTGGATGGCCCTCAAAGCATTTGAGGCAGTTC

GGACCTCGGCAGAGATTTTATATTATTTTTTTGTTGTAATCGCGCTTGGCCCACATGCTG

CACACTCCGATAGACACGGCATTGGCTAAACCCGCTAATTGCGTGGTGATGCGTCCACGT

GCTGCGCACTTTCATGCGGAGTCCCCCCGAGTCCATAGAATAAGAGGGGGACGCGTTTCC

TTTTTCCCATCAGCAAACAACACCAACTCACTGTGCTAGTATAGTTCATCTGAGAACAGA

CACACTGCTGAGCAAGCTCTCCTTCTCCAACTAGCTTTTTCTTTGCTGACATTTCCTACA

GAATGGCAGCAAAACAGATTCCGTTAACACAACTACTGCTCAGGTTGATTTAGAGGCCGC

CTTGGCCGAAGCGATCGAGTCTACATGGAGATTTGCATCAGACTTTCCTCTTAAAAATGC

MATCGCGAAACACTGCCTCGCCAATTGGCTTATCGCTCGAAAGACTTCAAGCAACCACCA

ATTGATAGTTCACCGTGAATTCCATGGGCCACACCTGCCGGAAAACAGCGTAGGGGCAGT

CTGCAAATCTCTCAGACTTTTTAATATGGTCGAGATCAACGTAGTACCGACCAAAGATAA

AGATTTCATAGTCGTCCACGACTTCAACACAGAAAGAACGACGCAGTTAGGCAATTGAAA

TTGGTTTGATATGTCGACGAAAGAGGCTGTTGGCACCCCCTTGGTAGTTCCCATAATGGG

CGGTGACTCCCTAACCAGCGAGTTCACACCCACCGATGAGAAAGTTGTCAAGCAAGAAAC

CCTTCTCAAGAATGCCTTTGAAACAAACAAACAGGCAACCATACGGATTGACGCTCACGA

GAGGCAGGCCGCTCCCCTCGTCGCTTGGCTGAGCCATAGGCCGGCCTATCACGACAAGAC

AATCGTTATCTTTCATACATATGGATACAAAAATGGACACGAGTTCGCCTTGGCGGTCAA

TCAAGCCGGCGCTACAAAAAATTGGCGCGAAACTGTAGCGCTAATGCCAAACATTCTGCC

GAATGAACTCTCTAGGCTCGCGAAGCAATATCCACCAAATTGCACGTCGCATTTTTACGA

CCTCGTTGAGGCCAGCAAGAATTGGTTCGCATCTGTCTTCTCCCAGAATATGATGGTGGT

CGTCACTTTCCTCAAGATTGCGGGCACTGGCGAAGGACAGACTAACAAAGTCGTTGATCC

AAATGGAAATGAGGTTCTGCATCCTAAAGAGAAGGAGGAATATCTCCGCGATTCAGCTTC

AATCGAAATCGCGAAATGAATCTGCGCCACCCATCCCGGAATCGAGATTGGAGGCGTCAC

ACGAACTTATGCTTATAAGAGTCGAGGACATCGCTACAAAATCAATCTTTGGACGGGTCA

ACCGAAGCGGTGGCCGACCCACGCGACGAAGTACATCTGGGAAGATTTCGCCAAGCCTGG

AAATGCGCTCAGATGGGGTCAAACGATTATCTCTGATCGCATTGGTGACGACCTTGCAGC

TTGTGTGCTTCAAAGCCGTGGAGAAAATTTTGTCAGCAGTCATGAATATCCTGCTGTTCA

TGTCGATTAGATGGGCGATCTGTGTCTTAGTCTAAGCTTCCGCTTAACGCATCTTTGCTA

CTTTAATGTATTTTTTTAATGATATTGTTGGAGTTTTGAGCACATCTTAATCGCAAGTAG

ATATTTTTTTTGAATCGGATTCGACTTCAAGCAGATGTACTTCTTTGTCTCTCGATAAAT

CTAATGACCCTGCGAGCCGGCCTCTCACCTTAAACTAATTTCTGAGAGTTAGGTCGGCGC

CTCTCGTGGTTTCCATGTTCTATGGAAACGGCCGCTCGGGACTTGCCTTCCCGCAACTCA

TCGAGCTTGCCTGGCCCGCTCGGGTGGTGGCACAAGCCATCGTTAAATCGTTCGTAATCC

TCTACTCTCGCATAAATGAGAGGGGAATCCAAAGATGATCACTTTTCAGCTAATTGGAAT

CTGCAATCCCAAAAGAGGCAAGCACTATGTGGGGTCCACAACTTATAGAATTTTGGCCTA

ACCATTCTGCATAATGGATCATGACATGGGCCATTTATGGCCACAAAAACTGGCACAAAT

TCCAACCTATTCAGCACAATGAACAAGTTCTTCCAATTGGAATAAAGGAGGTTAAAATTA

TTCGAGTAGACATGACGTGCTGGCACGCCACTAGCCTCGCATTGATGCCACTCTTTCTTG

CGCACACTTATATAATGCTGAGCTAATAATAGAGATAAAAATAGTCAGCTTGTAAACTGG

ACCTTTATTACTGCCCCTAATCTCAAGATCAAAGCGACATATACCTCTTCTCTTTCAAGT

CTTTTTGCAACTTGTTCCAAAGCAAGCGTTTGTGAATCTGCCAGGCAGCCTTCACGAAAA

TGGCTGATCAACTGTGTGAGGGGCGTTGGTCCGAAGCCAATCTATCATTCGTTGGGATAT

CCATCACCCCTGAATTCATCGATGAGGCCCTCAGAGGTTTTTGGGAGTCTTTTGCCAAAA

GAGCCGTTGGCTACAAAGAGGTTTACGTTCAGGTTGATCGAGATCCTAAAGTTGCCTGGG

AAGGAGCGCTCAATGGTTACCTCGGCAAATATTCTGGCGTAAAAAATGTTCATTTTGCGC

TGAACAACGCCACGATTGGAAAGCCCCCACGCCATATCGACATTTTGCTTCGGAAAAAAT

TTAGCGGCGAACTTCTTGCAACGCTCGAAGGTGTGGCAATTTCAAACGGTGCAACTGGTT

GGCTTGCGCTCGCATGTGTAAACCTTCTCTTGAAGGCAAGACAAGATATTGATGTCTTCT

TGTTTGGGGCAGGAAAAGTCACCGAGGCTGTGATTCTTGCCCTCAATCATGGTGCGGCTG

CAAAAATCAAAACCATGGCAGTGCTCAGCCAAGCTCCAATTGAAGGTGGGGTTCCAGCTC

GATGCCATCATTGATCTGAAGGTTATACCAAAAGCCAAACTCGTCATAATAGCAACAAAT

TCTGAGGAGCTCGGTACTAGAGGCCGACGAAATTGCGCCAAATGCAGTAACCATATCCCT

CGGAAAAGATGAATTGCCCGCCGCCTACTTCGATCGCCTTTTGAACGCAGAGGGTCTGAT

TATTGGCGACGATCTGGATGCGATCGAATCGCGCAATGTCAATTCCCTGGTGCTTTACTA

CTCGAAACGTGATTTGAAGCTGACAGAACATGGACGGGATCATTGGATAAAGAACTACGC

CAATGTCCTTGCTGATCCAGCTCTCATGGAGGAACTTAAGACATGAGAGGGGCCAGCCAA

CTTTTCATCGGTTGGCCTTGCCAGCCTAGACTTGGCGATGGCCGGCCGACTCTACGAAAC

TCTTACTGCGAAACTCTCCCACCCCCAGTAGACAAAACCCTCTTAGGATTGCGCTCTTCA

GAAGGTGCGCCTCCCCAGTCACAACCGGAAAGTCACTCAACTTGGCTCAAGTACCCTACT

TAGTTTGTCTCTCTGTTGTGTTTAGTATCAGCAACAATAACAAATATTGTAAATAACATA

TATTTATATGGTAATTATATGGCATGTAAGCGTGACAACGATTATGTTTTCCATAGTTTG

ATAAACTCAACACCCGAAAAAGCCGGTGACCAAAGGGTTGGGACCTCTTTGCACGAACTC

CTATGGCTGCCAAACTATCTACAGAGCTTTGACGCAGCATCTGATCGCCACACAATCCTC

GCGGACGTGGATGGCGTGGCTACATGCAAGCAGTTTGACTGAGAGTTTTCGAGTGACTAT

TTTAAAAGGACAATATTTCGTCCCAAGCGATAAAACTCGCTCACATGACTAAGTCGTTGC

TTTAATTCAACTGCGTGAAAAGATTTGAAGGGCAGAACTGTTTGTGTTTCGTATCCAGTT

CACCGGCAATTATTGGCTACGCATGAAGCTCATTATCGTCGATTCTTTACCCAAACACTG

ATGCCATTCCAAATGAGTTGGGCCAAAGATGTACTCATGTCGGACAGGCGCGATTTGGAC

TTCGTGATCCTCAAGTCTATCGCAGTGCTGCTTTATTACAGGCTATGCTTCCACCCTCCA

TTATTTGGTCAACTGGACCTAGCTTTTGACAAGAATAGGCCAGCGTTCATCTATCTATCT

TTCAAAAACATGCAGTTGCTGTTGCGTAAAGGATGTAACATCGGATTTGGTGTGGAGACG

GTGCTCGCTACAACTATACTGCCTTACTGTAGTGATCTGTCCCGCCAAGCGATGAGGGGA

GCGGCCTGCCTCTCGTGAGCGTCAATCCATACGGTTGCATGTTCGTTTGTTTCAAAGGCA

TTCCCAAGAAGGGTTTCCAGCTTGACAACTTTCTCATCGGTGGGTGTGAACTCGCCGGTT

AGTGAGTCACCGCCCATTTTGGGAACTACCAGGGGGGTGCCAACGACTTCCTCCGTCGAC

ATCTCAGACCAATTTCAATTGCCCAACTGCACCGTTCTTTCTATGTTGAAGTCATGGGCG

ACTATGAAATCTCCATCTTTGGTCGGTACTACCTCGATCTCGACCATATTGAAAAGCCTG

AAAGATTTGCAAACTGCCCCTACACTGTTTTCCGGCAAGTGTGGCCCATGGAATCCACGG

TGAACTATCAATTGGCGGTCGCTTGAAGTCTTTCGAGCGATAAGCCAATTGGCGAGGCAG

TGTTTCGCGATCGCATCTTTAAGAGGAAAGTTCGATGCAAATCTCCATGTAGACCCAGTC

GTTTCGGCCAAGGCGGCCTCTAAATCAACTTGAGCAGCAGTTGTGTTAACGGAATCTGTT

TTGACTGCCATTCTGTAAGAAATGTCAGCAAAGGAAAAGCTAGTTAGAGAAGGAGAGCTT

GCTCAATAGTGTGTCTATTCTCAAATGAACTATACTAGCGCAATGAGTTGGTGTTGTTCG

CTGATGGAAAAAAGGAAACGCATCCCCCTCTTCTACAAAATTCTGTGGGCCCGGAGGGAC

TCCGTATGAAAGTGCACAGCATGTGGACGCATCACCATGCAATTAGCAAGCTTAGCCAAT

GTCGTGTCTGTCAGAGTGTGCAGCATGTGAGCCAAGCGCAATTACAATAAAAAAATAATA

TAAAAACTCTGCCGAGGTCCGAACCGTCTCAAATGCTTTGAGGGCCATCCAAGCCAAGCG

CACCTAACGCCTAGCACACCATTGTTGCGCCGCTCGAGCATTAATGGTTCGGGACCTGGT

GTCATCTGGAAACCTATGAAGCATGGACACGGCAAAATCAGCGTCGKGGCGGTGTCAGAC

ACGAGGACACGTCGGGGACACGTACAGGAC

>ss0635_Un

GAAGTATCAATTTGATCTAGAAGCCAATCCAAACTCATTGAAAGGCCTGTCATCAACCGG

TCAACACGGTTCAATGTTTGCTTGCGTGGCGCAGCAATGGTGTGCTAGGCGTTAGGTGCG

CCTGGCTTGGATGGCCCTCAAAGCATTTGAGGCAGTTCGGACCTCGGCAGAGATTTTATA

TTATTTTTTTGTTGTAATCGCGCTTGGCCCACATGCTGCACACTCCGATAGACACGGCAT

TGGCTAAACCCGCTAATTGCGTGGTGATGCGTCCACGTGCTGCGCACTTTCATGCGGAGT

CCCCCCGAGTCCATAGAATAAGAGGGGGACGCGTTTCCTTTTTCCCATCAGCAAACAACA

CCAACTCACTGTGCTAGTATAGTTCATCTGAGAACAGACACACTGCTGAGCAAGCTCTCC

TTCTCCAACTAGCTTTTTCTTTGCTGACATTTCCTACAGAATGGCAGCAAAACAGATTCC

GTTAACACAACTACTGCTCAGGTTGATTTAGAGRCCGCCTTGGCCGAAGCGATCGAGTCT

ACATGGAGATTTGCATCAGACTTTCCTCTTAAAAATGCAATCGCGAAACACTGCCTCGCC

AATTGGCTTATCGCTCGAAAGACTTCAAGCAACCACCAATTGATAGTTYACCGTGAATTC

CATGGGCCACACCTGCCGGAAAACAGCGTAGGGGCAGTCTRCAAATCTCTCAGACTTTTT

AATATGGTCGAGATCAACGTAGTACCGACCAAAGATAAAGATTTCATAGTCGTCCACGAC

TTCAACACAGAAAGAACGACGCAGTTAGGCAATTGAAATTGGTTTGATATGTCGACGAAA

GAGGCTGTTGGCACCCCCTTGGTAGTTCCCATAATGGGCGGTGACTCCCTAACCAGCGAG

TTCACACCCACCGATGAGAAAGTTGTCAAGCAAGAAACCCTTCTCAAGAATGCCTTTGAA

ACAAMCAAACAGGCAACCATACGGATTGACGCTCACGAGAGGCAGGCCGCTCCCCTCGTC

GCTTGGCTGAGCCATAGGCCGGCCTATCACGACAAGACAATCGTTATCTTTCATACATAT

GGATACAAAAATGGACACGAGTTCGCCTTGGCGGTCAATCAAGCCGGCGCTACAAAAAAT

TGGCGCGAAACTGTAGCRCTAATGCCAAACATTCTGCCGAATGAACTCTCTAGGCTCGCG

AAGCAATATCCACCAAATTGCACGTCGCATTTTTACGACCTCGTTGAGGCCAGCAAGAAT

TGGTTCGCATCTGTCTTCTCCCAGAATATGATGGTGGTCGTCACTTTCCTCAAGATTGCG

GGCACTGGCGAAGGACAGACTAACAAAGTCGTTGATCCAAATGGAAATGAGGTTCTGCAT

CCTAAAGAGAAGGAGGAATATCTCCGCGATTCAGCTTCAATCGAAATCGCGAAATGAATC

TGCGCCACCCATCCYGGAATCGAGATTGGAGGCGTCACACGAACTTATGCTTATAAGAGT

CGAGGACATCGCTACAAAATCAATCTTTGGACGGGTCAACCGAAGCGGTGGCCGACCCAC

GCGACGAAGTACATCTGGGAAGATTTCGCCAAGCCTGGAAATGCGCTCAGATGGGATCAA

ACGATTATCTCTGATCGCATTGGTGACGACCTTGCAGCTTGTGTGCTTCAAAGCCGTGGA

GAAAATTTTGTCAGCAGTCATGAATATCCTGCTGTTCATGTCGATTAGATGGGCGATCTG

TGTCTTAGTCTAAGCTTCCGCTTAACGCATCTTTGCTACTTTAATGTATTTTTTTAATGA

TATTGTTGGAGTTTTGAGCACATCTTAATCGCAAGTAGATATTTTTTTTGAATCGGATTC

GACTTCAAGCAGATGTACTTCTTTGTCTCTCGATAAATCTAATGACCCTGCGAGCCGGCC

TCTCACCTTAAACTAATTTCTGAGAGTTAGGTCGGCGCCTCTCGTGGTTTCCATGTTCTA

TGGAAACGGCCGCTCGGGACTTGCCTTCCCGCAACTCATCGAGCTTGCCTGGCCCGCTCG

GGTGGTGGCACAAGCCATCGTTAAATCGTTCGTAATCCTCTACTCTCGCATAAATGAGAG

GGGAATCCAAAGATGATCACTTTTCAGCTAATTGGAATCTGCAATCCCAAAAGAGGCAAG

CACTATGTGGGGTCCACAACTTATAGAATTTTGGCCTAACCATTCTGCATAATGGATCAT

GACATGGGCCATTTATGGCCACAAAAACTGGCACAAATTCCAACCTATTCAGCACAATGA

ACAAGTTCTTCCAATTGGAATAAAGGAGGTTAAAATTATTCGAGTAGACATGACGTGCTG

GCACGCCACTAGCCTCGCATTGATGCCACTCTTTCTTGCGCACACTTATATAATGCTGAG

CTAATAATAGAGATAAAAATAGTCAGCTTGTAAACTGGACCTTTATTACTGCCCCTAATC

TCAAGATCAAAGCGACATATACCTCTTCTCTTTCAAGTCTTTTTGCAACTTGTTCCAAAG

CAAGCGTTTGTGAATCTGCCAGGCAGCCTTCACGAAAATGGCTGATCAACTGTGTGAGGG

GCGTTGGTCCGAAGCCAATCTATCATTCGTTGGGATATCCATCACCCCTGAATTCATCGA

TGAGGCCCTCAGAGGTTTTTGGGAGTCTTTTGCCAAAAGAGCCGTTGGCTACAAAGAGGT

TTACGTTCAGGTTGATCGAGATCCTAAAGTTGCCTGGGAAGGAGCGCTCAATGGTTACCT

CGGCAAATATTCTGGCGTAAAAAATGTTCATTTTGCGCCGAACAACGCCACGATTGGAAA

GCCCCCACGCCATATCGACATTTTGCTTCGGAAAAAATTTAGCGGCGAACTTCTTGCAAC

GCTCGAAGGTGTGGCAATTTCAAACGGTGCAACTGGTTGGCTTGCKCTCGCMTGTGTAAA

CCTTCTCTTGAAGGCAAGACAAGATATTGATGTCTTCTTGTTTGGGGCAGGAAAAGTCAC

CGAGGCTGTGATTCTTGCCCTCAATCATGGTGCGGCTGCAAAAATCAAAACCATGGCAGT

GCTCAGCCAAGCTCCAATTGAAGGTGGGGTTCCAGCTCGATGCCATCATTGATCGGAAGG

TTATACCAAAAGCCAAACTCGTCATAATAGCAACAAATTCTGAGGAGCTCGGTACTCGAG

GCCGACGAAATTGCGCCAAATGCAGTAACCATATCCCTCGGAAAAGATGAATTGCCCGCC

GCCTACTTCGATCGCCTTTTGAACGCAGAGGGTCTGATTATTGGCGACGATCTGGATGCG

ATCGAATCGCGCAATGTCAATTCCCTGGTGCTTTACTACTCGAAACGTGATTTGAAGCTG

ACAGAACATGGAMGGGATCATTGGATAAAGAACTACGCCAATGTCCTTGCTGATCCAGCT

CTCATGGAGGAACTTAAGACATGAGAGGGGCCAGCCAACTTTTCATCGGTTGGCCTTGCC

AGCCTAGACTTGGCGATGGCCGGCCGACTCTACGAAACTCTTACTGCGAAACTCTCCCAC

CCCCAGTAGACAAAACCCTCTTAGGATTGCGCTCTTCAGAAGGTGCGCCTCCCCAGTCAC

AACCGGAAAGTCACTCAACTTGGCTCAAGTACCCTACTTAGTTTGTCTCTCTGTTGTGTT

TAGTATCAGCAACAATAACAAATATTGTAAATAACATATATTTATATGGTAATTATATGG

CATGTAAGCGTGACAACGATTATGTTTTCCATAGTTTGATAAACTCAACACCCGAAAAAG

CCGGTGACCAAAGGGTTGGGACCTCTTTGCACGAACTCCTATGGCTGCCAAACTATCTAC

AGAGCTTTTTGACGCAGCATCTGATCGCCACACAATCCTCGCGGACGTGGATGGCGTGGC

TACATGCAAGCAGTTTGACTGAGAGTTTTCGAGTGACTATTTTAAAAGGACAATATTTCG

TCCCAAGCGATAAAACTCGCTCACATGACTAAGTCGTTGCTTTAATTCAACTGCGTGAAA

AGATTTAAAGGGCAGAACTGTTTGTGTTTCGTATCCAGTTCACCGGCAATTATTRGCTAC

GCATGAAGCTCATTATCGTCGATTCTTTACCCAAACACTGATGCCATTCCAAATGAGTTG

GGCCAAAGATGTACTCATGTCGGACAGGCGCGATTTGGACTTCGTGATCCTCAAGTCTAT

CGCAGTGCTGCTTTATTACAGGCTATGCTTCCACCCTCCATTATTTGGTCAACTGGACCT

AGCTTTTGACAAGAATAGGCCAGCGTTCATCTATCTATCTTTCAAAAACATGCAGTTGCT

GCTGCGTAAAGGATGTAACATCGGATTTGGTGTGGAGACGGTGCTCGCTACAACTATACT

GCCTTACTGTAGTGATCTGTCCCGCCAAGCGATGAGGGGAGCGGCCTGCCTCTCGTGAGC

GTCAATCCATACGGTTGCATGTTCGTTTGTTTCAAAGGCATTCCCAAGAAGGGTTTCCAG

CTTGACAACTTTCTCATCGGTGGGTGTGAACTCGCCGGTTAGTGAGTCACCGCCCATTTT

GGGAACTACCAGGGGGGTGCCAACGACTTCCTCCGTCGACATCTCAGACCAATTTCAATT

GCCCAACTGCACCGTTCTTTCTATGTTGAAGTCATGGGCGACTATGAAATCTCCATCTTT

GGTCGGTACTACCTCGATCTCGACCATATTGAAAAGCCTGAAAGATTTGCAAACTGCCCC

TACACTGTTTTCCGGCAAGTGTGGCCCATGGAATCCACGGTGAACTATCAATTGGCGGTC

GCTTGAAGTCTTTCGAGCGATAAGCCAATTGGCGAGGCAGTGTTTCGCGATCGCATCTTT

AAGAGGAAAGTCCGATGCAAATCTCCATGTAGACCCAGTCGTTTCGGCCAAGGCGGCCTC

TAAATCAACTTGAGCAGCAGTTGTGTTAACGGAATCTGTTTTGACTGCCATTCTGTAAGA

AATGTCAGCAAAGGAAAAGCTAGTTAGAGAAGGAGAGCTTGCWCAATAGTGTGTCTATTC

TCAAATGAACTATACTAGCGCAATGAGTTGGTGTTGTTCGCTGATGGAAAAAAGGAAACG

CATCCCCCTCTTCTACAAAATTCTGTGGGCCCGGAGGGACTCCGTATGAAAGTGCAYAGC

ATGTGGACGCATCACCATGCAATTAGCAAGCTTAGCCAATGTCGTGTCTGTCAGAGTGTG

CAGCATGTGAGCCAAGCGCAATTACAATAAAAAAATAATATAAAAACTCTGCCGAGGTCC

GAACCGTCTCAAATGCTTTGAGGGCCATCCAAGCCAAGCGCACCTAACGCCTAGCACACC

ATTGTTGCGCCGCTCGAGCATTAATGGTTYGGGACCTGGTGTCATCTGGAAACCTATGAA

GCATGGACACGGCAAAATCAGCGTYGTGGCGGTGTCAGACACGAGGACACGTCGGGGACA

CGTACAGGACAC

>ss0664_1

CTCTCCAAATTTCCCTCACCTTGAAGTATCAATTTGATCTAGAAGCCAATCCAAACTCAT

TGAAAGGCCTGTCATCAACCGGTCAACACGGTTCAATGTTTGCTCGCGTGGCGCAGCAAT

GGTGTGCTAGGCGTTAGGTGCGCCTGGCTTGGATGGCCCTCAAAGCATTTGAGGCAGTTC

GGACCTCGGCAGAGATTTTATATTATTTTTTTGTTGTAATCGCGCTTGGCCCACATGCTG

CACACTCCGATAGACACGGCATTGGCTAAACCCGCTAATTGCGTGGTGATGCGTCCACGT

GCTGCGCACTTTCATGCGGAGTCCCCCCGAGTCCATAGAATAAGAGGGGGACGCGTTTCC

TTTTTTCCATCAGCAAACAACACCAACTCACTGTGCTAGTATAGTTCATCTGAGAACAGA

CACACTGCTGAGCAAGCTCTCCTTCTCCAACTAGCTTTTTCTTTGCTGACATTTCCTACA

GAATGGCAGCAAAACAGATTCCGTTAACACAACTACTGCTCAGGTTGATTTAGAGGCCGC

CTTGGCCGAAGCGATCGGGTCTACATGGAGATTTGCATCAGACTTTCCTCTTAAAAATGC

AATCGCGAAACACTACCTTGCCAATTGGCTTATCGCTCGAAAGACTTCAAGCAACCACCA

ATTGATAGTTCACCGTGAATTCCATGGGCCACACCTGCCGGAAAACAGCGTAGGGGCAGT

CTGCAAATCTCTCAGACTTTTTAATATGGTCGAGATCAACGTAGTACCGACCAAAGATAA

AGATTTCATAGTCGTCCACGACTTCAACACAGAAAGAACGACGCAGTTAGGCAATTGAAA

TTGGTTTGATATGTCGACGAAAGAGGCTGTTGGCACCCCCCTGGTAGTTCCCATAATGGG

CGGTGACTCCCTAAAGAGCGAGTTCACACCCACCGATGAGAAAGTTGTCAAGCAAGAAAC

CCTTCTCAAGAATGCCTTTGAAACAAACAAACAGGCAACCATACGGATTGACGCTCACGA

GAGGCAGGCCGCTCCCCTCGTCGCTTGGCTGAGCCATAGGCCGGCCTATCACGACAAGAC

AATCGTTATCTTTCATACATATGGATACAACAATGGACACGAGTTCGCCTTGGCGGTCGA

TCAAGCCGGCGCTACAAAAAATTGGCGCGAAACTGTAGCGCTAATGCCAAACATTCTGCC

GAATGAACTCTCTAGGCTCGCGAAGCAATATCCACCAAATTGCACGTCGCATTTTTACGA

CCTCGTTGAGGCCAGCAAGAATTGGTTCGCATCTGTCTTCTCCCAGAATATGATGGTGGT

CGTCACTTTCCTCAAGATTGCGGGCACTGGCAAAGGACAGACTAACAAAgTCGTTGATCC

AAATGGAAATGAGGTTCTGCATCCTAAAGAGAAGGAGGAATATCTCCGCGATTCAGCTTC

AATCGAAATCGCGAAATGAATCTGCGCCACCCATCCCGGAATCGAGATTGGAGGCGTCAC

ACGAACTTATGCTTATAAGAGTCGAGGACATCGCTACAAAATCAATCTTTGGACGGGTCA

ACCGAAGCGGTGGCCGACCCACGCGACGAAGTACATCTGGGAAGATTTCGCCAAGCCTGG

AAATGCGCTCAGATGGGGTCAAACGATTATCTCTGATCGCATTGGTGACGACCTTGCAGC

TTGTGTGCTTCAAAGCCGTGGAGAAAATTTTGTCAGCAGTCATGAATATCCTGCTGTTCA

TGTCGATTAGATGGGCGATCTGTGTCTTAGTCTAAGCTTCCGCTTAACGCATCTTTGCTA

CTTTAATGTATTTTTTTAATGATATTGTTGGAGTTTTGAGCACATCTTAATCGCAAGTAG

ATATTTTTTTTGAATCGGATTCGACTTCAAGCAGATGTACTTCTTTGTCTCTCGATAAAT

CTAATGACCCTGCGAGCCAGCCTCTCGCCTTAAACTAATTTCTGAGAGTTAGGTCGGCGC

CTCTCGTGGTTTCCATGTTCTATGGAAACGGCCGCTCGGGACTTGCCTTCCCGCAACTCA

TCGAGCTTGCCTGGCCCGCTCAGGTGGTGGCACAAGCCATCGTTAAATCGTTCGTAATCC

TCTACTCTCGCATAAATGAGAAGGGAATCCAAAGATGATCACTTTTCAGCTAATTGGAAT

CTGCAATCCCAAAAGAGGCAAGCACTATGTGGGGTCCACAACTTATAGAATTTTGGCCTA

ACCATTCTGCATAATGGATCATGACATAGGCCATTTATGGCCGCAAAAACTGGCACAAAT

TCCAACCTATTCAGCACAATGAACAAGTTCTTCCAATTGGAATAAAGGAGGTTAAAATTA

TTCAAGTAGACATGACGTGCTGGCACGCCACTAGCCTCGCATTGATGCCACTCTTTCTTG

CGCACACTTATATAATGCTGAGCTAATAATAGAGATAAAAATAGTCAGCTTGTAAACTGG

ACCTTTATTACTGCCCCTAATCTCAAGATCAAAGCAACATATACCTCTTCTCTTTCAAGT

CTTTTTGCAACTTGTTCTAAAGCAAGCGTTTGTGAATCTGCCAggcagccttcacgaaaa

tggctgatcaactatgtgaggggcgttggtccgaagccaatctatcattcgttgggatat

ccatcacccctgaattcatcgatgaggccctcagaggcttttgccaAAAGAGCCGTTGGC

TACAAAGAGGTTTACGTTCAGGTTGATCGAGATCCTAAAGTTGCCTGGGAAGGAGCGCTC

AATGGTTACCTCGGCAAATATTCTGGCGTAAAAAATGTTCATTTTGCGCCGAACAACGCC

ACGATTGGAAAGCCCCCACGCCATATTGACATTTTGCTTCGGAAAAAATTTAGCGGCGAA

CTTCTTGCGATGCTCGAAGGTGTGGCAATTTCAAACGGTGCAACTGGTTGGCTTGCGCTC

GCATGTGTAAACCTTCTCTTGAAGGCAAGACAAGATATTGATGTCTTCTTGTTTGGGGCA

GGAAAAGTCGCCGAGGCTGTGATTCTTGCCCTCAATCATGGTGCGGCTGCAAAAATCAAA

ACCATGGCAGTGCTCAGCCAAGCTCCAATTGAAGGTGGGGTTCCAGCTCGATGCCGTCAT

TGATCGGAAGGTTATACCAAAAGCCAAACTCGTCATAACAGCAACAAATTCCGAGGAGCT

CGGTACTCGAGGCCGACGAAATTGCGCCAaATGCAGTAACCACaTCCCTCGGAAAAGATG

AATTGCCCGCCGCCTACTTCGATCGCCTTTTGAACGCAGAGGGTCTGATTATTGGCGACG

ATCTGGATGTGATCGAATCGCGCAATGTCAATTCCCTGGTGCTTTACTACTCGAAACGTG

ATTTGAAACTGACAGAACATGGAAGGGATCATTGGATAAAGAACTACGCCAATGTCCTTG

CTGATCCAGCTCTCATGGAGGAACTTAAGACATGAGAGGGGCCAGCCAACTTTTCATCGG

TTGGCCTTGCCAGCCTAGACTTGGCGATGGCCGGCCGACTCTACGAAACTCTTACTGCGA

AACTCTCCCACCCCCAGTAGACAAAACCCTCTTAGGATTGCGCTCTTCAGAAGGTGCGCC

TCCCCAGTCACAACCGGAAAGTCACTCAACTTGGCTCAAGTACCCTACTTAGTTTGTCTC

TCTGTCGTGTTTAGTATCAGCAACAATAACAAATATTGTAAATAACATATATTTATATGG

TAATTATATGGCATGTAAGCGTGACAACGATTATGTTTTCCATAGTTTGATAAACTCAAC

ACCCGAAAAAGCCGGTGACCAAAGGGTTGGGACCTCTTTGCACGAACTCCTATGGCTGCC

AGACTATCTACAGAGCTTTTTCGGAAGCTGACGCAACATCTGATCGCCACACAATCCTCG

CGGACGTGGATGGCGTGGCTACATGCAAGCAGTTTGACTGAGAGTTTTCGAGTGACTATT

TTAAAAGGACAATATTTCGTCCCAAGCGATAAAACTcgctcacatgactaaGTCGTTGCT

TTAATTCAACTGCGTGAAAAGATTTAAAGGGCAGAACTGTTTGTGTTTCGTATCCAGTTC

ACCGGCAATTATTGGCTACGCATGAAGCTCATTATCGTCGATTCTTTACCCAAACACTGA

TGCCATTCCAAATGAGTTGGGCCAAAGATGTACTCATGTCGGACAGGCACGATTTGGACT

TCGTGATCCTCAAGTCTATCGCAGTGCTGCTTTATTACAGGCTATGCTTCCACCCTCCAT

TATttggtcaACTGGACCTAGCTTTTGACAAGAATAGGCCAGCGTTCATCTATCTATCTT

TCAAAAACATGcagttgctgctGCGTAAAGGATGTAACATCGGATTTGGTGTGGAGACGG

TGCTCGCTACGACTATACTGCCTTACTGTAGTGATCTGTCCCGCCAAGCGATGAGGGGAG

CGGCCTGCCTCTCGTGAGCGTCAATCCATACGGTTGCATGTTCGTTTGTTTCAAAGGCAT

TCCCAAGAAGGGTTTCCAGCTTGACAACTTTCTCATCGGTGGGTGTGAACTCGCCGGTTA

GTGAGTCACCGCCCATTTTGGGAACTACCAGGGGGGTGCCAACGACCTCCTCCGTCGACA

TCTCAGACCAATTTCAATTGCCCAACTGCACCGTTCTTTCTATGTTGAAGTCATGGGCGA

CTATGAAATCTCCATCTTTGGTCGGTACTACCCCGATCTCGACCATATTGAAAAGCCTGA

AAGATTTGCAAACTGCCCCTACACTGTTTTCCGGCAAGTGTGGCCCATGGAATCCACGGT

GAACTATCAATTGGCGGTCGCTTGAAGTCTTTCGAGCGATAAGCCAATTGGCGAGGCAGT

GTTTCGCGATCGCATCTTTAAGAGGAAAGTCCGATGCAAATCTCCATGTAAACCCAGTCG

TTTCGGCCAAGGCGGCCTCTAAATCAACTTGAGCAGCAGTTGTGTTAACGGAATCTGTTT

TGACTGCCATTCTGTAAGAAATGTCAGCAAAGGAAAAGCTAGTTAGAGAAGGAGAGCTTG

CTCAATAGTGTGTCTATTCTCAAATGAACTATACTAGCGCAATGAGTTGGTGTTGTTCGC

TGATGGGAAAAAGGAAACGCATCCCCCTCTTCTACAAAATTCTGTGGGCCCGGAGGGACT

CCGTATGAAAGTGCACAGCATGTGGACGCATCACCATGCAATTAGCAAGCTTAGCCAATG

TCGTGTCTGTCAGAGTGTGCAGCATGTGAGCCAAGCGCAATTACAATAAAAAAATAATAT

AAAAACTCTGCTGAGGTCCGAACCGTCTCAAATGCTTTGAGGGCCATCCAAGCCAAGCGC

ACCTAACGCCTAGCACACCATTGTTGCGCCGTTCGAGCATTAATGGTTCGGGACCTGGTG

TCATCTGGAAACCTATGAAGCATGGACACGGCAAAATCAGCGCCGTGGCGGTGTCAGACA

CGAgGgacacgt

>ss0664_2

cctcaccttgaagtatcaatttgatcTAGAAGCCAATCCAAACTCATTGAAAGGCCTGTC

TTCAACCTGTCAACACGGTTCAATGTTTGCTCGAGTGGTGCAGCAATGGTGTGCTAGGCG

TTAGGTGCGCCTGGCTGGGATGGCCCTCAAAGCATTTGAGACAGTTCGGACCTCGGCAGA

GATTTTATATTATTTTTTTGTTGTAATCGCGTTTGGCCCACATGCTGCACACTCCGACAG

ACACGGCATTGGCTAAACCCGCTAATTGCGTGGTGATGCGTCCACATGCTGCGCACTTTC

ATGCGGAGTCCCCCCGAGCCCGTAGAATTTTGTAGAAGAGGGGGACGCGTTTCCTTTTTC

CCATCAGCAAACAACACCAACTCACTGTGCTAGTATAGTTCATCTGAGAACAGACACACT

GCTGAGCAAGCTCTCCTTCTCCAACTAGCTTTTTCTTTGCTGACATTTCCTACAAAATGG

CAGCAAAACAGATTCCGTTAACACAACTGCTGCTCAGGTTGATTTAGAGGCCGCCTTGGC

CGAAGCGACTGGGTCTACATGGAGATTTGCATCAAACTTTCCTCTTAAAGATGCGATCGC

AAAACACTGCCTCGCCAATTGGCTTATCGCTTGAAAGACTTCAAGCAACCACCAATTGAT

AGTTCACCGTGAATTCCATGGGCCACACCTGCCGGAAAACAGCATAGGGGCAGTCTGCAA

ATCTCTCAGACTTTTTAATATGGTCGAGATCAACGTAGTACCGACCAAAGATAGAGATTT

CATAGTCGTCCACGACTTCAACACAGAAAGAACGACGCAGTTAGGCAATTGAAATTGGTC

TGATATGTCGATGAAAGAGGCCGTTGGCACCCCCCTAGTAGTTCCCATAATGGGCGGTGA

CTCCCTAACCAGCGAGTTCACACCCACCGATGAGAAAGTTGTCAAGCAAGAAACCCTTCT

CAGGAATGCCTTTGAAACAAACAAACAGGCAACCATACGGATTGACGCTCACGAGAGGCA

GGCCGCTCCCCTCGTCGCTTGGCTGAGCCATAGGCCGGCCTATCACGACAAGACAATCGT

TATCTTTCATACATATGGATACAACAATGGACACAAGTTCGCCTTGGCGGTCGATCAAGC

CGACGCTACAGAAAATTGGCGCGAAACTGTAGCGCTAATGCCAAACATTCTGCCAAATGA

ACTCTCTAGGCTCGCGAAGCAATATCCACCAAATATCACGTCGCATTTTTACGACCTCGT

TGAGGCCGGCAAGAATTGGTTCGCGTCTGTCTTCTCCCAGAATATGATGGTGGACGTCAC

TTTCCTCAAGATTGCGGGCACTGGCGAAGGACAGACTAACAAAGTCGTTGATCCAAATGG

AAATGAGGTTCTGCATCCTAAAGAGAAGGAGGAATATCTCCGCGATTCAGCTTCAATTGA

AATCGCGAAATGAATCTGCGCCACCCATCCCGGAATCGAGATTGGAGGCGTCACACGAAC

TTATGCTTATAAGAGTCGAGGACATCGCCACAAAATCGATCTTTGGACGGGTCAACCGAA

GCGGTGGCCGACCCACGCGACGAAGTATATCTGGGAAGATTTCGCCAAGCCTGGAAATGC

GCTCAGATGGGGTCAAACGATTATCTCTGATCGCATTGGCGACGACCTTGCAGCTTGTGT

GCTTCAAAGCCGTGGAGAAAATTTTGTCAGCAGTCATGAATATCCCGCTGTTCATGTCGA

TTAGATGGGCGATCTGTGTCTTAGTCTAAGCTTCCGCTTAACGCATCTTTGCTACTTTAA

TGTATTTTTTTAATGATATTGTTGGAGTTTTCAGCACATCTTAATCGCAAGTAGATATTT

TTTTTGAATCGGATTCGACTTCAAGCAGATGTACTTCTTTGTCTCTCGATAAATCTAATG

ACCCTGCGAGCCGGCCTTTCGCCTTAAACTAATTTCTGAGAGTTAGGTCGGCGCCTCTCG

TGGTTTCCATGTTCTATGGAAACGGCCTCTCGGGACTTGCCTTCCCGCAACTCATCGAGC

TTGCCTGGCCCGCTCGGGTGGTGGCGCAAGCCATCGTTAAATCGTTCGTAATCCTCTACT

CTCGCATAAATGAGAGGGGAATCCAAAGATGATCACTTTTCAGCTAATTGGAATCTGCAA

TCCTAAAAGAGGCAAGCACTATGTGGGGTCCACAACTTATAGAATTTTGGCCTAACCATT

CTGCATAATGGATCATGACATGGGCCATTTATGGCCGCAAAAACTGGCACAAATTCCAAC

CTGTTCAACACAATGAACAAGTTCTTCCAATTGGAATAAAGGAGGTTAAAATTATTCGAG

TAGACATGATGTGCCGGCACGCCACTAGCCTCGCATTGATGCCACCCTTTCTTGCGCACA

CTTATATAATGCTGAGCTAATAATAGAGATAAAAATAGTCAGCTTGTAAACTGGACCTTT

ACTACTGCCCCTAATCTCAAGATCAAAGCGACATATACCTCCTCTCTTTCAAGTCTTTTT

GCAACTTGTTCCAAAGCAAGCGTTCGTGAATCTGCCAGGCAGCCTTCACGAAAATGGCTG

ATCAACTATGTGAGGGGCGTTGGTCCGAAGCCAATCTATCATTCGTTGGGATATCCATCA

CCCCTGAATTCATCGATGAGGCCCTCAGAGGCTTTTGCCAAAAGAGCCGTTGGCTACAAA

GAGGTTTACGTTCGGGTTGATCGAGATCCTAAAGTTGCCTGGGAAGGAGCGCTCAATGGT

TACCTCGGCAAATATTCTGGCGTCAAAAATGTTCATTTTGCGCCGAACAACGCCGCGATT

GGAAAGCCCCCATGCCATATCGACATTTTGCTTCGGAAAAAATTTAGCGGCGAATTTCTT

GTGACGCTCGAAGGTGTGGCAATTTCAAACGGTGCAACTGGTTGGCTTGCGCTCGCGTGC

GTAAACCTTCTCTTGAAGGCAAGACAAGATATTGATGTCTTCTTGTTTGGGGCGGGAAAA

GTCGCCGAGGCTGTGATTCTTGCCCTCAATCACGGTGCGGCTGCAAAAATCAAACCATGG

CAGTGCTCAGCCAAGCTCCAATCGAAGGTGGGGTTCCAGCTCGATGTCGTCATTGATCGG

AAGGTTATACCAAAAGCCAAACTCGTCATAACAGCAACAAATTCCAAGGAGCTCGCACTC

GAGGCCGACGAAATTGCGCCAAATGCAGTAACCATATCCCTCGGAAAAGATGAATTGCTC

GCCGCCTACTTCGATCGCCTTTTGAACGCAGAGGGTCTGATTATCGGCGACGATCTGGAT

GCGATCGAATCGCGCAATGTCAATTCCCTGGTGCTTTACTACTCGAAACGTGATTTGAAG

CTGACAGAACATGGAAGGGATCATTGGATAAAAAACTACGCCAATGTCCTTGTTGATCCA

GCTCTCATGGAGGAACTTAAGACATGGGAGGGGCCAGCCAACTTTTCATCGGTTGGCCTT

GCCAGCCTAGACTTGGCGATGGTCGGCCGACTCTACGAAACTCTTACTGCGAAACTCTCC

CACCCCCAGTAGACAAGACCCTCTTAGGATTGCGCTCTTCAGAAGGTGCGCCTCCCCAGT

CACAACCGGAAAGTCACTCAACTTGGCTCAAGTACCCTACTTAGTTTGTCTCTCTGTCGT

GTTTAGTATCAACAACAATAACAAATATTGTAAACAACATATATTTATATGGTAATTATA

TGGCATGTAAGCGTGACAACGATTATGTTTTCCATAGTTTGATAAACTCAACACCCGAAA

AAGCCGGTGACCAAAGGGTTGGGACCTCTTTGCACGAACTCCTATGGCTGCCAGACTATC

TACAGAGCTTTTTCGAAAGCTGACGCAGCATCTGATCGCTACACAATCCTCGCGGACGTG

GATGGCGTGGCTACACGCAAGCAGTTTGACTGAGAGTTTTCGAGTGACTATTTTAAAAGG

ACGATATTTCGTCCCAAGCAATAAAACTCGCTCACATGACTAAGTCGTTGCTTTAATTCA

GCTGCGTGAAAAGATTTAAAGGGCAGAACTGTTTGTGTTTCGTATCCAGTTCACCGGCAA

TTATTGGCTACGCATGAAGCTCATTATCGTCGATTCTTTACCCAAACACTGATGTCATTC

CAAATGAGTTGGGCCAAAGATGTACTCATGTCGGATAGGCGCGATTTGGACTTCGTGATC

CTCAAGTCTATCGCAGTGCTGCTTTATTACAGGCTATGCTTCCACCCTCCATTATTTGGT

CAACTGGACCTGGCTTTTGACAAGAATAGGCCAGCGTTCATCTATCTATCTTTCAAAAAC

ATGCAGTTGCTGCTGCGTAAAGGATGTAACATCGGATTTGGTGTGGAGACGGTGCTTGCT

ACGACTATACTGCCTTACTGTAGTGATCTGTCCTACCAAGCGACGAGGGGAGCAGCCTGC

CTCTCGTGAGCGTCAATCCATACAGTTGCATGTTCGTTTGTTTCAAAGGCATTCCCAAGA

AGGGTTTCCAGCTTGACAACTTTCTCATCGGTGGGTGTGAACTAGCCGGTTAGTGAGTCA

CCGCCCATTTTGGGAACTACCAGGGGGGTGCCAACGACCTCCTCCGTCGACATCTCAGAC

CAATTTCAATTGCTCAACTGCGCCGTTCTTTCTATGTTGAAGTCATGGGCGACTATGAAA

TCTCCATCTTTGGTCGGTACTACCTCGATCTCGACCATATTGAAAAGCCTGAGAGATTTG

CAAATTGCCCCTACACTGTTTTCCGGCAAGTGTGGCCCATGGAATCCACGGTGAACTATC

AATTGGCGGTCGCTTGAAGTCTTTCGAGCGATAAGCCAATTGGCGAGGCAGTGTTTCGCG

ATCGCATCTTTAAGAGGAAAGTCCGATGCAAATCTCCATGTAGACCCGGTTGCTTCGGCC

AAGGCGGCCTCTAAACCAACTTGAGCAGCAGTTGTGTTAACGGAATCTATTTTGACTACC

ATTTTGTAAGAAATGTCAGCAAAGGAAAAGCTAGTTAGAGAAGGAGAGCTTGCTCAATAG

TGTGTCTATTCTCATATGAACTATACTAGCGCAATGAGTTAGTGTTGTTCGCTGATGGGA

AAAAGGAAATGCATCCCCCTCTTCTACAAAATTCTGTGGGCCCGGAGGGACTCCGTATGA

AAGTGCACAGCATGTGGACGCATCACCATGCAATTAGCGAGCTTAGCCAATGCCGTGTCT

GTCAGAGTGTGCAGCATGTGGGCCAAGCGCAATTACAATAAAAAAATAATATAAAAACTC

TGCCGAGGTCTGAACCGTCTCAAATGCTTTGAGGGCCATCCAAGCCAAGCGCACCTAACG

CCTAGCACACCATTGTTGCGCCGCTCGAGCATTAATGGTTCCGG

>ss0649_Un

GCCTCTCCAAATTTCCCTCACCTTGAAGTATCAATTTGATCTAGAAGCCAATCCAAACKC

AYTGAAAGSCCYGTCATCAMCCGSTCAACACSGTTCMAYGYTTGCTCGCGTGGCGCAGCA

ATGGTGTGCTAGGCGTTAGGTGCGCCTGGCTTGGATGGCCCTCAAAGCATTTGAGGCAGT

TCGGACCTCGGCAGAGATTTTATATTATTTTTTTGTTGTAATCGCGCTTGGCCCACMTGC

TGCACACTCCGATAGACACGGCATTGGCTAAACCCGCTAATTGCGTGGTGATGCGTCCAC

GTGCTGCGCACTTTCATGCGGAGTCCCCCCGAGTCCATAGAATAAGAGGGGGACGCGTTT

CCTTTTTCCCATCAGCAAACAACACCAACTCACTGTGCTAGTATAGTTCATCTGAGAACA

GACACACTGCTGAGCAAGCTCTCTTTCTCCAACTAGCTTTTTCTTTGCTGACATTTCCTA

CAGAATGGCAGCAAAACAGATTCCGTTAACACAACTACTGCTCAGGTTGATTTAGAGGCC

GCCTTGGCCGAAGCGATCGAGTCTACATGGAGATTTGCATCAGACTTTCCTCTTAAAAAT

GCAATCGCGAAACACTGCCTCGCCAATTGGCTTATCGCTCGAAAGACTTCAAGCAACCAC

CAATTGATAGTTCACCGTGAATTCCATGGGCCACACCTGCCGGAAAACAGCGTAGGGGCA

GTCTGCAAATCTCTCAGACTTTTTAATATGGTCGAGATCAACGTAGTACCGACCAAAGAT

AAAGATTTCATAGTCGTCCACGACTTCAACACAGAAAGAACGACGCAGTTAGGCAATTGA

AATTGGTTTGATATGTCGACGAAAGAGGCTGTTGGCACCCCCTTGGTAGTTCCCATAATG

GGCGGTGACTCCCTAACCAGCGAGTTCACACCCACCGATGAGAAAGTTGTCAAGCAAGAA

ACCCTTCTCAAGAATGCCTTTGAAACAAACAAACAGGCAACCATACGGATTGACGCTCAC

GAGAGGCAGGCCGCTCCCCTCGTCGCTTGGCTGAGCCATAGGCCGGCCTATCACGACAAG

ACAATCGTTATCTTTCATACATATGGATACAAAAATGGACACGAGTTCGCCTTGGCGGTC

AATCAAGCCGGCGCTACAAAAAATTGGCGCGAAACTGTAGCGCTAATGCCAAACATTCTG

CCGAATGAACTCTCTAGGCTCGCGAAGCAATATCCACCAAATTGCACGTCGCATTTTTAC

GACCTCGTTGAGGCCAGCAAGAATTGGTTCGCATCTGTCTTCTCCCAGAATATGATGGWG

GTCGTCACTTTCCTCAAGATTGCGGGCACTGGCGAAGGACAGACTAACAAAGTCGTTGAT

CCAAATGGAAATGAGGTTCTGCATCCTAAAGAGAAGGAGGAATATCTCCGCGATTCAGCT

TCAATCGAAATCGCGAAATGAATCTGCACCACCCATCCCGGAATCGAGATTGGAGGCGTC

ACACGAACTTATGCTTATAAGAGTCGAGGACATCGCTACAAAATCAATCTTTGGACGGGT

CAACCGAAGCGGTGGCCGACCCACGCGACGAAGTACATCTGGGAAGATTTCGCCAAGCCT

GGAAATGCGCTCAGATGGGGTCAAACGATTATCTCTGATCGCATTGGTGACGACCTTGCA

GCTTGTGTGCTTCAAAGCCGTGGAGAAAATTTTGTCAGCAGTCATGAATATCCTGCTGTT

CATGTCGATTAGATGGGCGATCTGTGTCTTAGTCTAAGCTTCCGCTTAACGCATCTTTGC

TACTTTAATGTATTTTTTTAATGATATTGTTGGAGTTTTGAGCACATCTTAATCGCAAGT

AGATATTTTTTTTGAATCGGATTCGACTTCAAGCAGATGTACTTCTTTGTCTCTCGATAA

ATCTAATGACCCTGCGAGCCGGCCTCTCGCCTTAAACTAATTTCTGAGAGTTAGGTCGGC

GCCTCTCGTGGTTTCCATGTTCTATGGAAACGGCCGCTCGGGACTTGCCTTCCCGCAACT

CATCGAGCTTGCCTGGCCCGCTCGGGTGGTGGCACAAGCCATCGTTAAATCGTTCGTAAT

CCTCTACTCTCGCATAAATGAGAGGGGAATCCAAAGATGATCACTTTTCAGCTAATTGGA

ATCTGCAATCCCAAAAGAGGCAAGCACTATGTGGGGTCCACAACTTATAGAATTTTGGCC

TAACCATTCTGCATAATGGATCATGACATGGGCCATTTATGGCCACAAAAACTGGCACAA

ATTCCAACCTATTCAGCACAATGAACAAGTTCTTCCAATTGGAATAAAGGAGGTTAAAAT

TATTCGAGTAGACATGACGTGCTGGCACGCCACTAGCCTCGCATTGATGCCACTCTTTCT

TGCGCACACTTATATAATGCTGAGCTAATAATAGAGATAAAAATAGTCAGCTTGTAAACT

GGACCTTTATTACTGCCCCTAATCTCAAGATCAAAGCGACATATACCTCTTCTCTTTCAA

GTCTTTTTGCAACTTGTTCCAAAGCAAGCGTTTGTGAATCTGCCAGGCAGCCTTCACGAA

AATGGCTGATCAACTGTGTGAGGGGCGTTGGTCCGAAGCCAATCTATCATTCGTTGGGAT

ATCCATCACCCCTGAATTCATCGATGAGGCCCTCAGAGGTTTTTGGGAGTCTTTTGCCAA

AAGAGCCGTTGGCTACAAAGAGGTTTACGTTCAGGTTGATCGAGATCCTAAAGTTGCCTG

GGAAGGAGCGCTCAATGGTTACCTCGGCAAATATTCTGGCGTAAAAAATGTTCATTTTGC

GCCGAACAACGCCACGATTGGAAAGCCCCCACGCCATATCGACATTTTGCTTCGGAAAAA

ATTTAGCGGCGAACTTCTTGCAACGCTCGAAGGTGTGGCAATTTCAAACGGTGCAACTGG

TTGGCTTGCGCTCGCATGTGTAAACCTTCTCTTGAAGGCAAGACAAGATATTGATGTCTT

CTTGTTTGGGGCAGGAAAAGTCACCGAGGCTGTGATTCTTGCCCTCAATCATGGTGCGGC

TGCAAAAATCAAAACCATGGCAGTGCTCAACCAAGCTCCAATTGAAGGTGGGGTTCCAGC

TCGATGCCATCATTGATCGGAAGGTTATACCAAAAGCCAAACTCGTCATAATAGCAACAA

ATTCTGAGGAGCTCGGTACTCGAGGCCGACGAAATTGCGCCAAATGCAGTAACCATATCC

CTCGGAAAAGATGAATTGCCCGCCGCCTACTTCGATCGCCTTTTGAACGCAGAGGGTCTG

ATTATTGGCGACGATCTGGATGCGATCGAATCGCGCAATGTCAATTCCCTGGTGCTTTAC

TACTCAAAACGTGATTTGAAGCTGACAGAACATGGAAGGGATCATTGGATAAAGAACTAC

GCCAATGTCCTTGCTGATCCAGCTCTCATGGAGGAACTTAAGACATGAGAGGGGCCAGCC

AACTTTTCATCGGTTGGCCTTGCCAGCCTAGACTTGGCGATGGCCGGCCGACTCTACGAA

ACTCTTACTGCGAAACTCTCCCACCCCCAGTAGACAAAACCCTCTTAGGATTGCGCTCTT

CAGAAGGTGCGCCTCCCCAGTCACAACCGGAAAGTCACTCAACTTGGCTCAAGTACCCTA

CTTAGTTTGTCTCTCTGTTGTGTTTAGTATCAGCAACAATAACAAATATTGTAAATAACA

TATATTTATATGGTAATTATATGGCATGTAAGCGTGACAACGATTATGTTTTCCATAGTT

TGATAAACTCAACACCCGAAAAAGCCGGTGACCAAAGGGTTGGGACCTCTTTGCACGAAC

TCCTATGGCTGCCAAACTATCTACAGAGCTTTGACGCAGCATCTGATCGCCACACAATCC

TCGCGGACGTGGATGGCGTGGCTACATGCAAGCAGTTTGACTGAGAGTTTTCGAGTGACT

ATTTTAAAAGGACAATATTTCGTCCCAAGCGATAAAACTCGCTCACATGACTAAGTCGTT

GCTTTAATTCAACTGCGTGAAAAGATTTAAAGGGCAGAACTGTTTGTGTTTCGTATCCAG

TTCACCGGCAATTATTGGCTACGCATGAAGCTCATTATCGTCGATTCTTTACCCAAACAC

TGATGCCATTCCAAATGAGTTGGGCCAAAGATGTACTCATGTCGGACAGGCGCGATTTGG

ACTTCGTGATCCTCAAGTCTATCGCAGTGCTGCTTTATTACAGGCTATGCTTCCACCCTC

CATTATTTGGTCAACTGGACCTAGCTTTTGACAAGAATAGGCCAGCGTTCATCTATCTAT

CTTTCAAAAACATGCAGTTGCTGCTGCGTAAAGGATGTAACATCGGATTTGGTGTGGAGA

CGGTGCTCGCTACGACTATACTGCCTTACTGTAGTGATCTGTCCCGCCAAGCGATGAGGG

GAGCGGCCTGCCTCTCGTGAGCGTCAATCCATACGGTTGCATGTTCGTTTGTTTCAAAGG

CATTCCCAAGAAGGGTTTCCAGCTTGACAACTTTCTCATCGGTGGGTGTGAACTCGCCGG

TTAGTGAGTCACCGCCCATTTTGGGAACTACCAGGGGGGTGCCAACGACTTCCTCCGTCG

ACATCTCAGACCAATTTCAATTGCCCAACTGCACCGTTCTTTCTATGTTGAAGTCATGGG

CGACTATGAAATCTCCATCTTTGGTCGGTACTACCTCGATCTCGACCATATTGAAAAGCC

TGAAAGATTTGCAAACTGCCCCTACACTGTTTTCCGGCAAGTGTGGCCCATGGAATCCAC

GGTGAACTATCAATTGGCGGTCGCTTGAAGTCTTTCGAGCGATAAGCCAATTGGCGAGGC

AGTGTTTCGCGATCGCATCTTTAAGAGGAAAGTCCGATGCAAATCTCCATGTAGACCCAG

TCGTTTCGGCCAAGGCGGCCTCTAAATCAACTTGAGCAGCAGTTGTGTTAACGGAATCTG

TTTTGACTGCCATTCTGTAAGAAATGTCAGCAAAGGAAAAGCTAGTTAGAGAAGGAGAGC

TTGCTCAATAGTGTGTCTATTCTCAAATGAACTATACTAGCGCAATGAGTTGGTGTTGTT

CGCTGATGGAAAAAAGGAAACGCATCCCCCTCTTCTACAAAATTCTGTGGGCCCGGAGGG

ACTCCGTATGAAAGTGCACAGCATGTGGACGCATCACCATGCAATTAGCAAGCTTAGCCA

ATGTCGTGTCTGTCAGAGTGTGCAGCATGTGAGCCAAGCGCAATTACAATAAAAAAATAA

TATAAAAACTCTGCCGAGGTCCGAACCGTCTCAAATGCTTTGAGGGCCATCCAAGCCAAG

CGCACCTAACGCCTAGCACACCATTGTTGCGCCGCTCGAGCATTAATGGTTCGGGACCTG

GTGTCATCTGGAAACCTATGAAGCATGGACACGGCAAAATCAGCGCCGTGGCGGTGTCAG

ACACGAGGACACGTCGGGGACACGTACAGGACACGT

>ss2439_Un

CCAAATTTCCCTCACCTTGMAGTATCAATTTGATCTAGAAGCCAMTCCAAACTCATTGAA

AGGCCTGTCATCAACCGGTCAACACGGTTCAATGTTTGCTTGCGTGGCGCAGCAATGGTG

TGCTAGGCGTTAGGTGCGCCTGGCTTGGATGGCCCTCAAAGCATTTGAGGCAGTTCGGAC

CTCGGCAGAGATTTTATATTATTTTTTTGTTGTAATCGCGCTTGGCCCACATGCTGCACA

CTCCGATAGACACGGCATTGGCTAAACCCGCTAATTGCGTGGTGATGCGTCCACGTGCTG

CGCACTTTCATGCGGAGTCCCCCCGAGTCCATAGAATAAGAGGGGGACGCGTTTCCTTTT

TCCCATCAGCAAACAACACCAACTCACTGTGCTAGTATAGTTCATCTGAGAACAGACACA

CTGCTGAGCAAGCTCTCCTTCTCCAACTAGCTTTTTCTTTGCTGACATTTCCTACAGAAT

GGCAGCAAAACAGATTCCGTTAACACAACTACTGCTCAGGTTGATTTAGAGGCCGCCTTG

GCCGAAGCGATCGAGTCTACATGGAGATTTGCATCAGACTTTCCTCTTAAAAATGCAATC

GCGAAACACTGCCTCGCCAATTGGCTTATCGCTCGAAAGACTTCAAGCAACCACCAATTG

ATAGTTCACCGTGAATTCCATGGGCCACACCTGCCGGAAAACAGCGTAGGGGCAGTCTGC

AAATCTCTCAGACTTTTTAATATGGTCGAGATCAACGTAGTACCGACCAAAGATAAAGAT

TTCATAGTCGTCCACGACTTCAACACAGAAAGAACGACGCAGTTAGGCAATTGAAATTGG

TTTGATATGTCGACGAAAGAGGCTGTTGGCACCCCCTTGGTAGTTCCCATAATGGGCGGT

GACTCCCTAACCAGCGAGTTCACACCCACCGATGAGAAAGTTGTCAAGCAAGAAACCCTT

CTCAAGAATGCCTTTGAAACAAACAAACAGGCAACCATACGGATTGACGCTCACGAGAGG

CAGGCCGCTCCCCTCGTCGCTTGGCTGAGCCATAGGCCGGCCTATCACGACAAGACAATC

GTTATCTTTCATACATATGGATACAAAAATGGACACGAGTTCGCCTTGGCGGTCAATCAA

GCCGGCGCTACAAAAAATTGGCGCGAAACTGTAGCGCTAATGCCAAACATTCTGCCGAAT

GAACTCTCTAGGCTCGCGAAGCAATATCCACCAAATTGCACGTCGCATTTTTACGACCTC

GTTGAGGCCAGCAAGAATTGGTTCGCATCTGTCTTCTCCCAGAATATGATGGTGGTCGTC

ACTTTCCTCAAGATTGCGGGCACTGGCGAAGGACAGACTAACAAAGTCGTTGATCCAAAT

GGAAATGAGGTTCTGCATCCTAAAGAGGAGGAGGAATATCTCCGCGATTCAGCTTCAATC

GAAATCGCGAAATGAATCTGCGCCACCCATCCCGGAATCGAGATTGGAGGCGTCACACGA

ACTTATGCTTATAAGAGTCGAGGACATCGCTACAAAATCAATCTTTGGACGGGTCAACCG

AAGCGGTGGCCGACCCACGCGACGAAGTACATCTGGGAAGATTTCGCCAAGCCTGGAAAT

GCGCTCAGATGGGGTCAAACGATTATCTCTGATCGCATTGGTGACGACCTTGCAGCTTGT

GTGCTTCAAAGCCGTGGAGAAAATTTTGTCAGCAGTCATGAATATCCTGCTGTTCATGTC

GATTAGATGGGCGATCTGTGTCTTAGTCTAAGCTTCCGCTTAACGCATCTTTGCTACTTT

AATGTATTTTTTTAATGATATTGTTGGAGTTTTGAGCACATCTTAATCGCAAGTAGATAT

TTTTTTTGAATCGGATTCGACTTCAAGCAGATGTACTTCTTTGTCTCTCGATAAATCTAA

TGACCCTGCGAGCCGGCCTCTCACCTTAAACTAATTTCTGAGAGTTAGGTCGGCGCCTCT

CGTGGTTTCCATGTTCTATGGAAACGGCCGCTCGGGACTTGCCTTCCCGCAACTCATCGA

GCTTGCCTGGCCCGCTCGGGTGGTGGCACAAGCCATCGTTAAATCGTTCGTAATCCTCTA

CTCTCGCATAAATGAGAGGGGAATCCAAAGATGATCACTTTTCAGCTAATTGGAATCTGC

AATCCCAAAAGAGGCAAGCACTATGTGGGGTCCACAACTTATAGAATTTTGGCCTAACCA

TTCTGCATAATGGATCATGACATGGGCCATTTATGGCCACAAAAACTGGCACAAATTCCA

ACCTATTCAGCACAATGAACAAGTTCTTCCAATTGGAATAAAGGAGGTTAAAATTATTCG

AGTAGACATGACGTGCTGGCACGCCACTAGCCTCGCATTGATGCCACTCTTTCTTGCGCA

CACTTATATAATGCTGAGCTAATAATAGAGATAAAAATAGTCAGCTTGTAAACTGGACCT

TTATTACTGCCCCTAATCTCAAGATCAAAGCGACATATACCTCTTCTCTTTCAAGTCTTT

TTGCAACTTGTTCCAAAGCAAGCGTTTGTGAATCTGCCAGGCAGCCTTCACGAAAATGGC

TGATCAACTGTGTGAGGGGCGTTGGTCCGAAGCCAATCTATCATTCGTTGGGATATCCAT

CACCCCTGAATTCATCGATGAGGCCCTCAGAGGTTTTTGGGAGTCTTTTGCCAAAAGAGC

CGTTGGCTACAAAGAGGTTTACGTTCAGGTTGATYGAGATCCTAAAGTTGCCTGGGAAGG

AGCGCTCAATGGTTACCTCGGCAAATATTCTGGCGTAAAAAATGTTCATTTTGCGCCGAA

CAACGCCACGATTGGAAAGCCCCCACGCCATATCGACATTTTGCTTCGGAAAAAATTTAG

CGGCGAACTTCTTGCAACGCTCGAAGGTGTGGCAATTTCAAACGGTGCAACTGGTTGGCT

TGCGCTCGCATGTGTAAACCTTCTCTTGAAGGCAAGACAAGATATTGATGTCTTCTTGTT

TGGGGCAGGAAAAGTCACCGAGGCTGTGATTCTTGCCCTCAATCATGGTGCGGCTGCAAA

AATCAAAACCATGGCAGTGCTCAGCCAAGCTCCAATTGAAGGTGGGGTTCCAGCTCGATG

CCATCATTGATCGGAAGGTTATACCAAAAGCCAAACTCGTCATAATAGCAACAAATTCTG

AGGAGCTCGGTACTCGAGGCCGACGAAATTGCGCCAAATGCAGTAACCATATCCCTCGGA

AAAGATGAATTGCCCGCCGCCTACTTCGATCGCCTTTTGAACGCAGAGGGTCTGATTATT

GGCGACGATCTGGATGCGATCGAATCGCGCAATGTCAATTCCCTGGTGCTTTACTACTCG

AAACGTGATTTGAAGCTGACAGAACATGGAACGGATCATTGGATAAAGAACTACGCCAAT

GTCCTTGCTGATCCAGCTCTCATGGAGGAACTTAAGACATGAGAGGGGCCAGCCAACTTT

TCATCGGTTGGCCTTGCCAGCCTAGACTTGGCGATGGCCGGCCGACTCTACGAAACTCTT

ACTGCGAAACTCTCCCACCCCCAGTAGACAAAACCCTCTTAGGATTGCGCTCTTCAGAAG

GTGCGCCTCCCCAGTCACAACCGGAAAGTCACTCAACTTGGCTCAAGTACCCTACTTAGT

TTGTCTCTCTGTTGTGTTTAGTATCAGCAACAATAACAAATATTGTAAATAACATATATT

TATATGGTAATTATATGGCATGTAAGCGTGACAACGATTATGTTTTCCATAGTTTGATAA

ACTCAACACCCGAAAAAGCCGGTGACCAAAGGGTTGGGACCTCTTTGCACGAACTCCTAT

GGCTGCCAAACTATCTACAGAGCTTTGACGCAGCATCTGATCGCCACACAATCATCGCGG

ACGTGGATGGCGTGGCTACATGCAAGCAGTTTGACTGAGAGTTTTCGAGTGACTATTTTA

AAAGGACAATATTTCGTCCCAAGCGATAAAACTCGCTCACATGACTAAGTCGTTGCTTTA

ATTCAACTGCGTGAAAAGATTTAAAGGGCAGAACTGTTTGTGTTTCGTATCCAGTTCACC

GGCAATTATTGGCTACGCATGAAGCTCATTATCGTCGATTCTTTACCCAAACACTGATGC

CATTCCAAATGAGTTGGGCCAAAGATGTACTCATGTCGGACAGGCGCGATTTGGACTTCG

TGATCCTCAAGTCTATCGCAGTGCTGCTTTATTACAGGCTATGCTTCCACCCTCCATTAT

TTGGTCAACTGGACCTAGCTTTTGACAAGAATAGGCCAGCGTTCATCTATCTATCTTTCA

AAAACATGCAGTTGCTGCGTAAAGGATGTAACATCGGATTTGGTGTGGAGACGGTGCTCG

CTACAACTATACTGCCTTACTGTAGTGATCTGTCCCGCCAAGCGATGAGGGGAGCGGCCT

GCCTCTCGTGAGCGTCAATCCATACGGTTGCATGTTCGTTTGTTTCAAAGGCATTCCCAA

GAAGGGTTTCCAGCTTGACAACTTTCTCATCGGTGGGTGTGAACTCGCCGGTTAGTGAGT

CACCGCCCATTTTGGGAACTACCAGGGGGGTGCCAACGACTTCCTCCGTCGACATCTCAG

ACCAATTTCAATTGCCCAACTGCACCGTTCTTTCTATGTTGAAGTCATGGGCGACTATGA

AATCTCCATCTTTGGTCGGTACTACCTCGATCTCGACCATATTGAAAAGCCTGAAAGATT

TGCAAACTGCCCCTACACTGTTTTCCGGCAAGTGTGGCCCATGGAATCCACGGTGAACTA

TCAATTGGCGGTCGCTTGAAGTCTTTCGAGCGATAAGCCAATTGGCGAGGCAGTGTTTCG

CGATCGCATCTTTAAGAGGAAAGTCCGATGCAAATCTCCATGTAGACCCAGTCGTTTCGG

CCAAGGCGGCCTCTAAATCAACTTRAGCAGCAGTTGTGTTAACGGAATCTGTTTTGACTG

CCATTCTGTAAGAAATGTCAGCAAAGGAAAAGCTAGTTAGAGAAGGAGAGCTTGCACAAT

AGTGTGTCTATTCTCAAATGAACTATACTAGCGCAATGAGTTGGTGTTGTTCGCTGATGG

AAAAAAGGAAACGCATCCCCCTCTTCTACAAAATTATGTGGGCCCGGAGGGACTCCGTAT

GAAAGTGCACAGCATGTGGACGCATCACCATGCAATTAGCAAGCTTAGCCAATGTCGTGT

CTGTCAGAGTGTGCAGCATGKGAGCCAAGCGCAATTACAATAAAAAAATAATATAAAAAC

TCTGCCGAGGTCCGAACCGTCTCAAATGCTTTGAGGGCCATCCAAGCCAAGCGCACCTAA

CGCCTAGCACACCATTATTGCGCCGCTCGAGCATTAATGGTTTGGGACTTGGTGTCATCT

GGAAACCTATGAAGCATGGACACGGCAAAATCAGCGTTGTGGCGGTGTCAGACACGAGGA

CACGTC

>ss0638_Un

CTTGAAGTATCAATTTGATCTAGAAGCCAATCCAAACTCATTGAAAGGCCTGTCATCAAC

CGGTCAACACGGTTCAATGTTTGCTTGCGTGGCGCAGCAATGGTGTGCTAGGCGTTAGGT

GCGCCTGGCTTGGATGGCCCTCAAAGCATTTGAGGCAGTTCGGACCTCGGCAGAGATTTT

ATATTATTTTTTTGTTGTAATCGCGCTTGGCCCACATGCTGCACACTCCGATAGACACGG

CATTGGCTAAACCCGCTAATTGCGTGGTGATGCGTCCACGTGCTGCGCACTTTCATGCGG

AGTCCCCCCGAGTCCATAGAATAAGAGGGGGACGCGTTTCCTTTTTCCCATCAGCAAACA

ACACCAACTCACTGTGCTAGTATAGTTCATCTGAGAACAGACACACTGCTGAGCAAGCTC

TCCTTCTCCAACTAGCTTTTTCTTTGCTGACATTTCCTACAGAATGGCAGCAAAACAGAT

TCCGTTAACACAACTACTGCTCAGGTTGATTTAGAGGCCGCCTTGGCCGAAGCGATCGAG

TCTACATGGAGATTTGCATCAGACTTTCCTCTTAAAAATGCAATCRCGAAACACTGCCTC

GCCAATTGGCTTATCGCTCGAAAGACTTCAAGCAACCACCAATTGATAGTTCACCGTGAA

TTCCATGGGCCACACCTGCCGGAAAACAGCGTAGGGGCAGTCTGCAAATCTCTCAGACTT

TTTAATATGGTCGAGATCAACGTAGTACCGACCAAAGATAAAGATTTCATAGTCGTCCAC

GACTTCAACACAGAAAGAACGACGCAGTTAGGCAATTGAAATTGGTTTGATATGTCGACG

AAAGAGGCTGTTGGCACCCCCTTGGTAGTTCCCATAATGGGCGGTGACTCCCTAACCAGC

GAGTTCACACCCACCGATGAGAAAGTTGTCAAGCAAGAAACCCTTCTCAAGAATGCCTTT

GAAACAAACAAACAGGCAACCATACGGATTGACGCTCACGAGAGGCAGGCCGCTCCCCTC

GTCGCTTGGCTGAGCCATAGGCCGGCCTATCACGACAAGACAATCGTTATCTTTCATACA

TATGGATACAAAAATGGACACGAGTTCGCCTTGGCGGTCAATCAAGCCGGCGCTACAAAA

AATTGGCGCGAAACTGTAGCGCTAATGCCAAACATTCTGCCGAATGAACTCTCTAGGCTC

GCGAAGCAATATCCACCAAATTGCACGTCGCATTTTTACGACCTCGTTGAGGCCAGCAAG

AATTGGTTCGCATCTGTCTTCTCCCAGAATATGATGGTGGTCGTCACTTTCCTCAAGATT

GCGGGCACTGGCGAAGGACAGACTAACAAAGTCGTTGATCCAAATGGAAATGAGGTTCTG

CATCCTAAAGAGGAGGAGGAATATCTCCGCGATTCAGCTTCAATCGAAATCGCGAAATGA

ATCTGCGCCACCCATCCCGGAATCGAGATTGGAGGCGTCACACGAACTTATGCTTATAAG

AGTCGAGGACATCGCTACAAAATCAATCTTTGGACGGGTCAACCGAAGCGGTGGCCGACC

CACGCGACGAAGTACATCTGGGAAGATTTCGCCAAGCCTGGAAATGCGCTCAGATGGGGT

CAAACGATTATCTCTGATCGCATTGGTGACGACCTTGCAGCTTGTGTGCTTCAAAGCCGT

GGAGAAAATTTTGTCAGCAGTCATGAATATCCTGCTGTTCATGTCGATTAGATGGGCGAT

CTGTGTCTTAGTCTAAGCTTCCGCTTAACGCATCTTTGCTACTTTAATGTATTTTTTTAA

TGATATTGTTGGAGTTTTGAGCACATCTTAATCGCAAGTAGATATTTTTTTTGAATCGGA

TTCGACTTCAAGCAGATGTACTTCTTTGTCTCTCGATAAATCTAATGACCCTGCGAGCCG

GCCTCTCACCTTAAACTAATTTCTGAGAGTTAGGTCGGCGCCTCTCGTGGTTTCCATGTT

CTATGGAAACGGCCGCTCGGGACTTGCCTTCCCGCAACTCATCGAGCTTGCCTGGCCCGC

TCGGGTGGTGGCACAAGCCATCGTTAAATCGTTCGTAATCCTCTACTCTCGCATAAATGA

GAGGGGAATCCAAAGATGATCACTTTTCAGCTAATTGGAATCTGCAATCCCAAAAGAGGC

AAGCACTATGTGGGGTCCACAACTTATAGAATTTTGGCCTAACCATTCTGCATAATGGAT

CATGACATGGGCCATTTATGGCCACAAAAACTGGCACAAATTCCAACCTATTCAGCACAA

TGAACAAGTTCTTCCAATTGGAATAAAGGAGGTTAAAATTATTCGAGTAGACATGACGTG

CTGGCACGCCACTAGCCTCGCATTGATGCCACTCTTTCTTGCGCACACTTATATAATGCT

GAGCTAATAATAGAGATAAAAATAGTCAGCTTGTAAACTGGACCTTTATTACTGCCCCTA

ATCTCAAGATCAAAGCGACATATACCTCTTCTCTTTCAAGTCTTTTTGCAACTTGTTCCA

AAGCAAGCGTTTGTGAATCTGCCAGGCAGCCTTCACGAAAATGGCTGATCAACTGTGTGA

GGGGCGTTGGTCCGAAGCCAATCTATCATTCGTTGGGATATCCATCACCCCTGAATTCAT

CGATGAGGCCCTCAGAGGTTTTTGGGAGTCTTTTGCCAAAAGAGCCGTTGGCTACAAAGA

GGTTTACGTTCAGGTTGATCGAGATCCTAAAGTTGCCTGGGAAGGAGCGCTCAATGGTTA

CCTCGRCAAATATTCTGGCGTAAAAAATGTTCATTTTGCGCCGAACAACGCCACGATTGG

AAAGCCCCCACGCCATATCGACATTTTGCTTCGGAAAAAATTTAGCGGCGAACTTCTTGC

AACGCTCGAAGGTGTGGCAATTTCAAACGGTGCAACTGGTTGGCTTGCGCTCGCATGTGT

AAACCTTCTCTTGAAGGCAAGACAAGATATTGATGTCTTCTTGTTTGGGGCAGGRAAAAG

TCACCGAGGCTGTGATTCTTGCCCTCAATCATGGTGCGGCTGCAAAAATCAAAACCATGG

CAGTGCTCAGCCAAGCTCCAATTGAAGGTGGGGTTCCAGCTCGATGCCATCATTGATCGG

AAGGTTATACCAAAAGCCAAACTCGTCATAATAGCAACAAATTCTGAGGAGYTCGGTACT

CGAGGCCGACGAAATTGCGCCAAATGCAGTAACCATATCCCTCGGAAAAGATGAATTGCC

CGCCGCCTACTTCGATCGCCTTTTGAACGCAGAGGGTCTGATTATTGGCGACGATCTGGA

TGCGATCGAATCGCGCAATGTCAATTCCCTGGTGCTTTACTACTCGAAACGTGATTTGAA

GCTGACAGAACATGGAASGGATCATTGGATAAAGAACTACGCCAATGTCCTTGCTGATCC

AGCTCTCATGGAGGAACTTAAGACATGAGAGGGGCCAGCCAACTTTTCATCGGTTGGCCT

TGCCAGCCTAGACTTGGCGATGGCCGGCCGACTCTACGAAACTCTTACTGCGAAACTCTC

CCACCCCCAGTAGACAAAACCCTCTTAGGATTGCGCTCTTCAGAAGGTGCGCCTCCCCAG

TCACAACCGGAAAGTCACTCAACTTGGCTCAAGTACCCTACTTAGTTTGTCTCTCTGTTG

TGTTTAGTATCAGCAACAATAACAAATATTGTAAATAACATATATTTATATGGTAATTAT

ATGGCATGTAAGCGTGACAACGATTATGTTTTCCATAGTTTGATAAACTCAACACCCGAA

AAAGCCGGTGACCAAAGGGTTGGGACCTCTTTGCACGAACTCCTATGGCTGCCAAACTAT

CTACAGAGCTTTGACGCAGCATGACGCAGCATCTGATCGCCACACAATCMTCGCGGACGT

GGATGGCGTGGCTACATGCAAGCAGTTTGACTGAGAGTTTTCGAGTGACTATTTTAAAAG

GACAATATTTCGTCCCAAGCGATAAAACTCGCTCACATGACTAAGTCGTTGCTTTAATTC

AACTGCGTGAAAAGATTTAAAGGGCAGAACTGTTTGTGTTTCGTATCCAGTTCACCGGCA

ATTATTGGCTACGCATGAAGCTCATTATCGTCGATTCTTTACCCAAACACTGATGCCATT

CCAAATGAGTTGGGCCAAAGATGTACTCATGTCGGACAGGCGCGATTTGGACTTCGTGAT

CCTCAAGTCTATCGCAGTGCTGCTTTATTACAGGCTATGCTTCCACCCTCCATTATTTGG

TCAACTGGACCTAGCTTTTGACAAGAATAGGCCAGCGTTCATCTATCTATCTTTCAAAAA

CATGCAGTTGCTGCTGCGTAAAGGATGTAACATCGGATTTGGTGTGGAGACGGTGCTCGC

TACAACTATACTGCCTTACTGTAGTGATCTGTCCCGCCAAGCGATGAGGGGAGCGGCCTG

CCTCTCGTGAGCGTCAATCCATACGGTTGCATGTTCGTTTGTTTCAAAGGCATTCCCAAG

AAGGGTTTCCAGCTTGACAACTTTCTCATCGGTGGGTGTGAACTCGCCGGTTAGTGAGTC

ACCGCCCATTTTGGGAACTACCAGGGGGGTGCCAACGACTTCCTCCGTCGACATCTCAGA

CCAATTTCAATTGCCCAACTGCACCGTTCTTTCTATGTTGAAGTCATGGGCGACTATGAA

ATCTCCATCTTTGGTCGGTACTACCTCGATCTCGACCATATTGAAAAGCCTGAAAGATTT

GCAAACTGCCCCTACACTGTTTTCCGGCAAGTGTGGCCCATGGAATCCACGGTGAACTAT

CAATTGGCGGTCGCTTGAAGTCTGTCGAGCGATAAGCCAATTGGCGAGGCAGTGTTTCGC

GATCGCATCTTTAAGAGGAAAGTCCGATGCAAATCTCCATGTAGACCCAGTCGTTTCGGC

CAAGGCGGCCTCTAAATCAACTTGAGCAGCAGTTGTGTTAACGGAATCTGTTTTGACTGC

CATTCTGTAAGAAATGTCAGCAAAGGAAAAGCTAGTTAGAGAAGGAGAGCTTGCACAATA

GTGTGTCTATTCTCAAATGAACTATACTAGCGCAATGAGTTGGTGTTGTTCGCTGATGGA

AAAAAGGAAACGCATCCCCCTCTTCTACAAAATTMTGTGGGCCCGGAGGGACTCCGTATG

AAAGTGCACAGCATGTGGACGCATCACCATGCAATTAGCAAGCTTAGCCAATGTCGTGTC

TGTCAGAGTGTGCAGCATGTGAGCCAAGCGCAATTACAATAAAAAAATAATATAAAAACT

CTGCCGAGGTCCGAACCGTCTCAAATGCTTTGAGGGCCATCCAAGCCAAGCGCACCTAAC

GCCTAGCACACCATTRTTGCGCCGCTCGAGCATTAATGGTTTGGGACTTGGTGTCATCTG

GAAACCTATGAAGCATGGACACGGCAAAATCAGCGTTGTGGCGGTGTCAGACACGAGGAC

ACGTCGGGGACA

>ss2464_2

attgaaaggcctgtcatcaaccggtcaacacggttcaatGTTTGCTCGCGTGGCGCAGCA

ATGGTGTGCTAGGCGTTAGGTGCGCCTGGCTTGGATGGCCCTCAAAGCATTTGAGGCAGT

TCGGACCTCGGCAGAGATTTTATATTATTTTTTTGTTGTAATCGCGCTTGGCCCACATGC

TGCACACTCCGATAGACACGGCATTGGCTAAACCCGCTAATTGCGTGGTGATGCGTCCAC

GTGCTGCGCACTTTCATGCGGAGTCCCCCCGAGTCCATAGAATAAGAGGGGGACGCGTTT

CCTTTTTCCCATCAGCAAACAACACCAACTCACTGTGCTAGTATAGTTCATCTGAGAACA

GACACACTGCTGAGCAAGCTCTCTTTCTCCAACTAGCTTTTTCTTTGCTGACATTTCCTA

CAGAATGGCAGCAAAACAGATTCCGTTAACACAACTACTGCTCAGGTTGATTTAGAGGCC

GCCTTGGCCGAAGCGATCGAGTCTACATGGAGATTTGCATCAGACTTTCCTCTTAAAAAT

GCAATCGCGAAACACTGCCTCGCCAATTGGCTTATCGCTCGAAAGACTTCAAGCAACCAC

CAATTGATAGTTCACCGTGAATTCCATGGGCCACACCTGCCGGAAAACAGCGTAGGGGCA

GTCTGCAAATCTCTCAGACTTTTTAATATGGTCGAGATCAACGTAGTACCGACCAAAGAT

AAAGATTTCATAGTCGTCCACGACTTCAACACAGAAAGAACGACGCAGTTAGGCAATTGA

AATTGGTTTGATATGTCGACGAAAGAGGCTGTTGGCACCCCCTTGGTAGTTCCCATAATG

GGCGGTGACTCCCTAACCAGCGAGTTCACACCCACCGATGAGAAAGTTGTCAAGCAAGAA

ACCCTTCTCAAGAATGCCTTTGAAACAAACAAACAGGCAACcatacggattgatgctcac

gagaggcaggccgctcccctcgtcgcttggctgagccataggccggccTATCACGACAAG

ACAATCGTTATCTTTCATACATATGGATACAACAATGGACACGAGTTCGCCTTGGCGGTC

GATCAAGCCGGCGCTACAAAAAATTGGCGCGAAACTGTAGCGCTAATGCCAAACATTCTG

CCGAATGAACTCTCTAGGCTCGCGAAGCAATatccaccaaATTGCACGTCGCATTTTTAC

GACCTCGTTGAGGCCAGCAAGAATTGGTTCGCATCTGTCTTCTCCCAGAATATGATGGTG

GTCGTCACTTTCCTCAAGATTGCGGGCAMTGGCGAAGGACAGACTAACAAAGTCGTTGAT

CCAAATGGAAATGAGGTTTTgCATcctaaagagaaggaggaatatctccgcgattcagct

tcaatcgaaatcgcgaaatgaATCTGCGCCACCCATCCCGGAATCGAGATTGGAGGCGTC

ACACGAACTTATGCTTATAAGAGTCGAGGACATCGCTACAAAATCAATCTTTGGACGGGT

CAACCGAAGCGGTGGCCGACCCACGCGACAAAGTACATCTGGGAAGATTTCgccaagcct

ggaaatgcgctcagatggggtcaaacgattatccctgatcgcattggtgacgaccttgca

gcttgtgtgcttcaaagccgtggagaaaatttTGTCAGCAGTCATGAATATCTTGCTGTT

CATGTCGATTAGATGGGCGATCTGTGTCTTAGTCTAAGCTTCCGCTTAACGCATCTTTGC

TACTTTAATGTATTTTTTTAATGATATTGTTGGAGTTTDGAGCACATCTTAATCGCAAGT

AGATATTTTTTTTGAATCGGATTTGACTTCAAGCAGATGTACTTCTTTGTCTCTCGATAA

ATCtaatgaccctgcgaccggcctctcgccttaaactaatttctgagagttaggtcgGCG

CCTCTCGTGGTTTCCATGTTTTATGGAAACGGCCGCTCGGGACTTGCCTTCCCGCAACTC

ATCGAGCTTGCCTGGCCCGCTCGGGTGGTGGCACAAGCCATCGTTAAATCGTTCGTAATC

CTCTACTCTCGCATAAATGAGAGGGGAATCCAAAGATGATCACTTTTCAGCTAATTGGAA

TCTGCAATCCCAAAAGAGGCAAGCACTATGTGGGGTCCACAACTTATAGAATTTTGGCCT

AACCATTCTGCATAATGGATCATGACATGGGCCATTTATGGCCGCAAAAACTGGCACAAA

TTCCAACCTATTCAGCACAATGAACAAGTTCTTCCAATTGGAATAAAGGAGGTTAAAATT

ATTCGAGTAGACATGACGTGCTGGCACGCCACTAGCCTCGCATTGATGCCACTCTTTCTT

GCGCACACTTATATAATGCTGAGCTAATAATAGAGATAAAAATAGTCCGCTTGTAAACTG

GACCTTTATTACTGCCCCTAATCTCAAGATCAAAGCGACATATACCTCTTCTCTTTCAAG

TCTTTTTGCAACTTGTTCCAAAGCAAGCGTTTGTGAATCTGCCAGGCAGCCTTCACGAAA

ATGGCTGATCAACTGTGTGAGGGGCGTTGGTCCGAAGCCAATCTATCATTCGTTGGGATA

TCTATCACCCCTGAATTCATCGATGAGGCCCTCAGAGGCTTTTGGGAGTCTTTTGCCAAA

AGAGCCGTTGGCTACAAAGAGGTTTACGTTCAGGTTGATCGAGATCCTAAAGTTGCCTGG

GAAGGAGCGCTCAATGGTTACCTCGGCAAATATTCTGGCGTAAAAAATGTTCATTTTGCG

CCGAACAACGCCACGATTGGAAAGCCCCCACGCCATATCGACATTTTGCTTCGGAAAAAA

TTTAGCGGCGAACKTCTTGCGACGCTCGAAGGTGTGGCAATTTCAAACGGTGCAACTGGT

TGGCTTGCGCTCGCATGTGTAAACCTTCTCTTGAAGGCAAGACAAGATATTGATGTCTTC

TTGTTTGGGGCAGGAAAAGTCACCGAGGCTGTGATTCTTGCCCTCAATCATGGTGCGGCT

GCAAAAATCAAAACCATGGCAGTGCTCAACCAAGCTCCAATTGAAGGTGGGGTTCCAGCT

CGATGCCATCATTGATCGGAAGGTTATACCAAAAGCCAAACTCGTCATAATAGCAACAAA

TTCTGAGGAGCTCGGTACTCGAGGCCGACGAAATTGCGCCAAATGCAGTAACCATATCCC

TCGGAAAAGATGAATTGCCCGCCGCCTACTTCGATCGCCTTTTGAACGCAGAGGGTCTGA

TTATTGGCGACGATCTGGATGCGATCGAATCGCGCAATGTCAATTCCCTGGTGCTTTACT

ACTCAAAACGTGATTTGAAGCTGACAGAACATGGAAGGGATCATTGGATAAAGAACTACG

CCAATGTCCTTGCTGATCCAGCTCTCATGGAGGAACTTAAGACATGAGAGGGGCCAGCCA

ACTTTTCATCGGTTGGCCTTGCCAGCCTAGACTTGGCGATGGCCGGCCGACTCTACGAAA

CTCTTACTGCGAAACTCTCCCACCCCCAGTAGACAAAACCCTCTTAGGATtgcgctcttc

agaaggtgcgcctccccagtcacaaccgaaaagtcactcaacttggctcaagtaccctac

ttagtTTGTCTCTCTGTCGTGTTTAGTATCAGCAACAATAACAAATATTGTAAATAACAT

ATATTTATATGGTAATTATATGGCATGTAAGCGTGACAACGATTATGTTTTCCATAGTTT

GATAAACTCAACACCCGAAAAAGCCGGTGACCAAAGGGTTGGGACCTCTTTGCACGAACT

CCTATGGCTGCCAAACTATCTACAGAGCTTTGACGCAGCATCTGATCGCCACACAATCCT

CGCGGACGTGGATGGCGTGGCTACATGCAAGCAGTTTGACTGAGAGTTTTCGAGTGACTA

TTTTAAAAGGACAATATTTCGTCCCaagcgataaaactcgctcaCATGACTAAGTCATTG

CTTTAATTCAACTGCGTGAAAAGATTTAACGGGCAGAACTGTTTGTGTTTCGTATCCAGT

TCACCGGCAATTATTGGCTACGCATGAAGCTCATTATCGTCGATTCTTTACCCAAACACT

GATGCCATTCCAAATGAGTTGGGCCAAAGATGTACTCATGTCGGACAGGCGCGATTTGGA

CTTCGTGATCCTCAAGTCTATCGCAGTGCTGCTTTATTACAGGCTATGCTTCCACCCTCC

ATTATTTGGTCAACTGGACCTAGCTTTTGACAAGAATAGGCCAGCGTTCATCTATCTATC

TTTCAAAAACATGCAGTTGCTGCTGCGTAAAGGATGTAACATCGGATTTGGTGTGGAGAC

GGTGCTCGCTACGACTATACTGCCTTACTGTAGTGATCTGTCCCGCCAAGCGATGAGGGG

AGCGGCCTGCCTCTCGTGAGCGTCAATCCATACGGTTGCATGTTCGTTTGTTTCAAAGGC

ATTCCCAAGAAGGGTTTCCAGCTTGACAACTTTCTCATCGGTGGGTGTGAACTCGCCGGT

TAGTGAGTCACCGCCCATTTTGGGAACTACCAGGGGGGTGCCAACGACYTCCTCCGTCGA

CATCTCAGACCAATTTCAATTGCCCAACTGCACCGTTCTTTCTATGTTGAAGTCATGGGC

GACTATGAAATCTCCATCTTTGGTCGGTACTACCTCGATCTCGACCATATTGAAAAGCCT

GAAAGATTTGCAAACTGCCCCTACACTGTTTTCCGGCAAGTGTGGCCCATGGAATCCACG

GTGAACTATCAATTGGCGGTCGCTTGAAGTCTTTCGAGCGATAAGCCAATTGGCGAGGCA

GTGTTTCGCGATCGCATCTTTAAGAGGAAAGTCCGATGCAAATCTCCATGTAGACCCAGT

CGTTTCGGCCAAGGCGGCCTCTAAATCAACTTGAGCAGCAGTTGTGTTAACGGAATCTGT

TTTGACTGCCATTCTGTAAGAAATGTCAGCAAAGGAAAAGCTAGTTAGAGAAGGAGAGCT

TGCTCAATAGTGTGTCTATTCTCAAATGAACTATACTAGCGCAATGAGTTGGTGTTGTTC

GCTGATGGAAAAAAGGAAACGCATCCCCCTCTTCTACAAAATTCTGTGGGCCCGGAGGGA

CTCCGTATGAAAGTGCACAGCATGTGGACGCATCACCATGCAATTAGCAAGCTTAGCCAA

TGTCGTGTCTGTCAGAGTGTGCAGCATGTGAGCCAAGCGCAATTACAATAAAAAAATAAT

ATAAAAACTCTGCCGAGGTCCGAACCGTCTCAAATGCTTTGAGGGCCATCCAAGCCAAGC

GCACCTAACGCCTAGCACACCATTGTTGCGCCGCTCGAGCATTAATGGTTCG

>ss2464_1

cggacctcggcagagattttatattatttttttgttgtaatcgcgcttggcccacatgct

gcacactccgatagacacggcattggctaaacccgctaattgcgtggtgatgcgtccacg

tgctGCGCACTTTCATGCGGAGTCcCCCCGagtccatagaatacgagggggacgcatttc

ctttttcccatcagcaaacaacaccaactcactgtgctagtatagttcatctgagaACAG

ACACACTGCtGAGCAAGCTCTCCTTCTCCAaCTAgctttttctttgctgaCATTtCCgAC

AGAATGGCAGCAAAACAGATTCCgTTAAcACAACTAcTGCTcAGGTTGATTTAGAGGCCG

TCTTGGCCGAAGCGATCGGGTCTACATGGAGATTTGCATCAGACTTTCCTCTTAAAAATG

CAATCGCGAAACACTGCCTCGCCAATTGGCTTATCGCTCGAAAGACTTCAAGCAACCACC

AATTGATAGTTCACCGTGAATTCCATGGGCCACACCTGCCCGAAAACAGCGTAGGGGCAG

TCTGCAAATCTCTCAGACTTTTTAATATGGTCGAGATCAACGTAGTACCGACCAAAGATA

AAGATTTCATAGTCGTCCACGACTTCAACACAGAAAGAACGACGCAGTTAGGCAATTGAA

ATTGGTTTGATATGTCGACGAAAGAGGCTGTTGGCACCCCCCTGGTAGTTCCCATAATGG

GCGGTGACTCCCTAACCAGCGAGTTCACACCCACCGATGAGAAAGTTGTCAAGCAAGAAA

CCCTTCTCAAGAATGCCTTTGAAACAAACAAACAGGCAACCATACGGATTGACGCTCACG

AGAGGCAGGCCGCTCCCCTCGTCGCTTGGCTGAGCCATAGGCCGGCCTATCACGACAAGA

CAATCGTTATCTTTCATACATATGGATACAACAATGGACACGAGTTCGCCTTGGCGGTCA

ATCAAGCCGGCGCTACAAAAAATTGGCGCGAAACTGTAGCGCTAATGCCAAACATTCTGC

CGAATGAACTCTCTAGGCTCGCGAAGCAATATCCACCAAATTGCACGTCGCATTTTTACG

ACCTCGTTGAGGCCAGCAAGAATTGGTTCGCATCTGTCTTCTCCCAGAATATGATGGTGG

TCGTCACTTTCCTCAAGATTGCGGGCACTGGCGAAGGACAGACTAACAAAGTCGTTGATC

CAAATGGAAATGAGGTTCTGCATCCTAAAGAGAAGGAGGAATATCTCCGCGATTCAGCTT

CAATCGAAATCGCGAAATGAATCTGCACCACCCATCCCGGAATCGAGATTGGAGGCGTCA

CACGAACTTATGCTTATAAGAGTCGAGGACATCGCTACAAAATCAATCTTTGGACGGGTC

AACCGAAGCGGTGGCCGACCCACGCGACGAAGTACATCTGGGAAGATTTCGCCAAGCCTG

GAAATGCGCTCAGATGGGGTCAAACGATTATCCCTGATCGCATTGGTGACGACCTTGCAG

CTTGTGTGCTTCAAAGCCGTGGAGAAAATTTTGTCAGCAGTCATGAATATCCTGCTGTTC

ATGTCGATTAGATGGGCGATCTGTGTCTTAGTCTAAGCTTCCGCTTAACGCATCTTTGCT

ACTTTAATGTATTTTTTTAATGATATTGTTGGAGTTTTGAGCACATCTTAATCGCAAGTA

GATATTTTTTTTGAATCGGATTCGACTTCAAGCAGATGTACTTCTTTGTCTCTCGATAAA

TCTAATGACCCTGCGAGCCGGCCTCTCGCCTTAAACTAATTTCTGAGAGTTAGGTCGGCG

CCTCTCGTGGTTTCCATGTTCTATGGAAACGGCCGCTCGGGACTTGCCTTCCCGCAACTC

ATCGAGCTTGCCTGGCCCGCTCGGGTGGTGGCACAAGCCATCGTTAAATCGTTCGTAATC

CTCTACTCTCGCATAAATGAGAGGGGAATCCAAAGATGATCACTTTTCAGCTAATTGGAA

TCTGCAATCCCAAAAGAGGCAAGCACTATGTGGGGTCCACAACTTATAGAATTTTGGCCT

AACCATTCTGCATAATGGATCATGACATGGGCCATTTATGGCCRYAAAAACTGGCACAAA

TTCCAACCTATTCAGCACAATGAACAAGTTCTTCCAATTGGAATAAAGGAGGTTAAAATT

ATTCGAGTAGACATGACGTGCTGGCACGCCACTAGCCTCGCATTGATGCCACTCTTTCTT

GCGCACACTTATATAATGCTGAGCTAATAATAGAGATAAAAATAGTCAGCTTGTAAACTG

GACCTTTATTACTGCCCCTAATCTCAAGATCAAAGCGACATATACCTCTTCTCTTTCAAG

TCTTTTTGCAACTTGTTCCAAAGCAAGCGTTTGTGAATCTGCCAGGCAGCCTTCACGAAA

ATGGCTGATCAACTGTGTGAGGGGCGTTGGTCCGAAGCCAATCTATCATTCGTTGGGATA

TCCATCACCCTGAATTCATCGATGAGGCCCTCAGAGGTTTTTGGGAGTCTTTTGCCAAAA

GAGCCGTTGGCTACAAAGAGGTTTACGTTCAGGTTGATCGAGATCCTAAAGTTGCCTGGG

AAGGAGCGCTCAATGGTTACCTCGGCAAATATTCTGGCGTAAAAAATGTTCATTTTGCGC

CGAACAACGCCACGATTGGAAAGCCCCCACGCCATATCGACATTTTGCTTCGGAAAAAAT

TTAGCGGCGAACTTCTTGCGACGCTCGAAGGTGTGGCAATTTCAAACGGTGCAACTAGTT

GGCTTGCGCTCGCATGTGTAAACCTTCTCTTGAAGGCAAGACAAGAAATTGATGTCTTCT

TGTTTGGGGCAGGAAAAGTCGCGGAGGCTGTGATTCTTGCCCTTAATCATGGTGCGGCTG

CAAAAaTCAAAACCATGGCAGTGCTCAGCCAAGCTCCAATTGAAGGTGGGGTTCCAGCTC

GATGCCGTCATTGATCGGAAGGTTATACCAAAAGCCAAACTCGTCATAACAGCAACAAAT

TCCGAGGAGCTCGGTACTCGAGGCCGACGAAATTGCGCCAAATGCAGTAACCATATCCCT

CGGAAAAGATGAATTGCCCGCCGCCTACTTCGATCGCCTTTTGAACGCAGAGGGTCTGAT

TATTGGCGACGATCTGGATGCGAYCGAATCGCGCAATGTCAATTCCCTGGTGCTTTACTA

CTCGAAACGTGATTTGAAGCTGACAGAACATGGAAGGGATCATTGGATAAAGAACTACGC

CAATGTCCTTGCTGATCCAGCTCTCATGGAGGAACTTAAGACATGAGAGGGGCCAGCCAA

CTTTTCATCGGTTGGCCTTGCCAGCCTAGACTTGGCGATGGCCGGCCGACTCTACGAAAC

TCTTACTGCGAAACTCTCCCACCCCCAGTAGACAAAACCCTCTTAGGATTGCGCTCTTCA

GAAGGTGCGCCTCCCCAGTCACAACCGGAAAGTCACTCAACTTGGCTCAAGTACCCTACT

TAGTTTGTCTCTCTGTCGTGTTTAGTATCAGCAACAATAACAAATATTGTAAATAACATA

TATTTATATGGTRATTATATGGCATGTAAGCGTGACAACGATTATGTTTTCCATAGTTTG

ATAAACTCAACACCCGAAAAAGCCGGTGACCAAAGGGTTGGGACCTCTTTGCACGAACTC

CTATGGCTGCCAGACTATCTACAGAGCTTTTTCGGAAGCTGACGCAGCATCTGATCGCCA

CACAATCCTCGCGGACGTGGATGGCGTGGCTACATGCAAGCAGTTTGACTGAGAGTTTTC

GAGTGACTATTTTAAAAGGACAATATTTCGTCCCAAGCGATAAAACTCGCTCACATGACT

AAGTCGTTGCTTTAATTCAACTGCGTGAAAAGATTTAAAGGGCAGAACTGTTTGTGTTTC

GTATCCAGTTCACCGGCAATTATTGGCTACGCATGAAGCTCATTATCGTCGATTCTTTAC

CCAAACACTGATGCCATTCCAAATGAGTTGGGCCAAAGATGTACTCATGTCGGACAGGCG

CAATTTGGACTTCGTGAtcctcaagtctatcgcagtgctgctttattaCAgGCTATGCTT

CCACCCTCCATTATTTGGTCAACTGGACCTAGCTTTTGACAAGAATAGGCCAGCGTTCAT

CTATCTATCTTTCAAAAACATGCAGTTGCTGCTGCgtaaaggatgtaacatcggatttgg

tgtggagacggtgctcgctacgactatactgccttactgtagtgatctgtcccgccaagc

gatgaggggagcggcctgcctctcgtgagcgtcaatccatacggttgcatgttcgtttgt

ttcaaaagcattcccaagaagggtttccagcttgacaactttctcatcggtgggtgtgaa

ctcgccggttagtgagtcaccgcccattttgggaactaccaggggggtgccaacgacttc

ctccgtcgacatctcagaccaatttcaattgcccaactgcaccgttctttctatgttgaa

gtcatgggtgactatgaaatctccatctTTGGTCGGTACTAtCTCGATCTCGACCATATT

GAAAAGCCTGAAAGATTTGCAAACTGCCCCTACACTGTTTTCTGGCAAGTGTGGCCCATG

GAATCCACGGTGAGCTATCAATTGGCGGTCGCTTGAAGTCTTTCGAGCGATAAGCCAAtt

ggcgaggcagtgtttcgcgatCGCATCTTTAAGAGGaaagtccgatgcaaatctccatgt

agacccagtcgttTCGGCCAAGGCGGCCTCTAAATCAACTTGAGTAGCAGTTGTGTTAAC

GGAATCTGTTTTGACTGCCATTCTGTAAGAAATGTCAGCAAAGGAAAAGCTAGTTAGAGA

AGGAGAGCTTGCTCAATAGTGTGTCTATTCTCAAATGAACTATACTAGCGCAATGAGTTG

GTGTTGTTCGCTGATGGGAAAAAGGAAACGCATCCCCCTCTTCTACAAAATTCTGTGGGC

CCGGAGGGACTCCGTATGAAAGTGCACAGCATGTGGACGCATCACCATGCAATTAGCAAG

CTTAGCCAATGTCGTGTCTGTCAGAGTGTGCAGCATGTGAGCCAAGCGCAATTACAATAA

AAAAATAATATAAAAACTCTGCCGAGGTCCGAACCGTCTCAAATGCTTTGAGGGCCATCC

AAGCCAAGCGCACCTAACGCCTAGCACACCATTGTTGCGCCGCTCGAGCATTAATGGTTC

GGGACCTGGTGTCATCTGGAAACCTATGAAGCATGGACACGGCAAAATCAGCGCCGTGGC

GGTGTCAGACACGAGGACACGTCGGggacacgtacaggacacgt

>ss2463_1

gaAGTATCAATTTGATCTAGAAGCCAATCCAAACTCATTGAAAGGCCTGTCATCAACCGG

TCAACACGGTTCAATGTTTGCTCGCGTGGCGCAGCAATGGTGTGCTAGGCATTAGGTGCG

CCTGGCTTGGATGGCCCTCAAAGCATTTGAGGCAGTTCGGACCTCGGCAGAGATTTTATA

TTATTTTTTTGTTGTAATCGCGCTTGGCCCACATGCTGCACACTCCGATAGACACGGCAT

TGGCTAAACCCGCTAATTGCGTGGTGATGCGTCCACGTGCTGCGCACTTTCAGGCGGAGT

CCCCCCGATTCCATAGAATAAGAGGGGGACGCGTTTCCTTTTTCCCATCAGCAAACAACA

CCAACTCACTGTGCTAGTATAGTTCATCTGAGAACAGACACACTGCTGAGCAAGATCTCC

TTCTCCAACTAGCTTTTTCTTTGCTGACATTTCCTACAGAATGGCAGCAAAACAGATTCC

GTTAACACAACTACTGCTCAGGTTGATTTAGAGGCCGTCTTGGCCGAAGCGATCGGGTCT

ACATGGAGATTTGCATCAGACTTTCCTCTTAAAAATGCAATCGCGAAACACTGCCTCGCC

AATTGGCTTATCGCTCGAAAGACTTCAAGCAACCACCAATTGATAGTTCACCGTGAATTC

CATGGGCCACACCTGCCGGAAAACAGCGTAGGGGCAGTCTGCAAATCTCTCAGACTTTTT

AATATGGTCGAGATCAACGTAGTACCGACCAAAGATAAAGATTTCATAGTCGTCCACGAC

TTCAACACAGAAAGAACGACGCAGTTAGGCAATTGAAATTGGTTTGATATGTCGACGAAA

GAGGCTGTTGGCACCCCCCTGGTAGTTCCCATAATGGGCGGTGACTCCCTAACCAGCGAG

TTCACACCCACCGATGAGAAAGTTGTCAAGCAAGAAACCCTTCTCAAGAATGCCTTTGAA

ACAAACAAACAGGCAACCATACGGATTGATGCTCACGAGAGGCAGGCCGCTCCCCTCGTC

GCTTGGCTGAGCCATAGGCCGGCCTATCACGACAAGACAATCGTTATCTTTCATACATAT

GGATACAACAATGGACACGAGTTCGCCTTGGCGGTCGATCAAGCCAGCGCTACAAAAAAT

TGGCNCGAAACTGTAGCGCTAATGCCAAACATTCTGCCGAGTGAACTCTCTAGGCTCGCG

AAGCAATATCCACCAAATTGCACGTCGCATTTTTACGACCTCGTTGAGGCCAGCAAGAAT

TGGTTCGCATCTGTCTTCTCCCAGAATATGATGGTGGTCGTCACTTTCCTCAAGATTGCG

GGCACTAGCGAAGGACAGACTAACAAAGTCGTTGATCCAAATGGAAATGAGGTTYTGCAT

CCTAAAGAGAAGGAGGAATATCTCCGCGATTCAGCTTCAATCGAAATCGCGAAATGAATC

TGCGCCACCCATCCCGGAATCGAGATTGGAGGCGTCACACGAACTTATGCTTATAAGAGT

CGAGGACATCGCTACAAAATCAATCTTTGGACGGGTCAACCGAAGCGGTGGCCGACCCAC

GCGACGAAGTACATCTAGGAAGATTTCGCCAAGCCTGGAAATGCGCTCAGATGGGGTCAA

ACGATTATCTCTGATCGCATTGGTGACGACCTTGCAACTTGTGTGCTTCAAAGCCGTGGA

GAAAATTTTGTCAGCAGTCATGAATATCCTGCTGTTCATGTCGATTAGATGGGCGATCTG

TGTCTTAGTCTAAGCTTCCGCTTAACACATCTTTGCTACTTTAATGTATTTTTTTAATGA

TATTGTTGGAGTTTTGAGCACATCTTAATCGCAAGTAGATATTTTTTTTGAATCGGATTC

GACTTCAAGCAGATGTACTTCTTTGTCTCTCGATAAATCTAATGACCCTGCGAGCCGGCC

TCTCGCCTTAAACTAATTTCTGAGAGTTAGGTCGGCGCCTCTCGTGGTTTCCATGTTCTA

TGGAAACGGCCGCTCGGGACTTGCCTTCCCGCAACTCATCGAGCTTGCCTGGCCCGCTCG

GGTGGTGGCACAAGCCATCGTTAAATCGTTCGTAATCCTCTACTCTCGCATAAATGAGAG

GGGAATCCAAAGATGATCACTTTTCAGCTAATTGGAATCTGCAATCCCAAAAGAGGCAAG

CACTATGTGGGGTCCACAACTTATAGAATTTTGGCCTAACCATTCTGCATAATGGATCAT

GAGATGGGCCATTTATGGCCGCAAAAACTGGCACAAATTCCAACCTATTCAGCACAATGA

ACAAGTTCTTCCAATTGGTATAAAGGAGGTTAAAATTATTCGAGTAGACATGACGTGCTG

GCACGCCACTAGCCTCGCATTGATGCCACTCTTTCTTGCGCACACTTATATAATGctgag

ctaatAATAGAGATAAAAATAGTCCGCTTGTAAACTGGACCTTTATTACTGCCCCTAATC

TCAAGATCAAAGCGACATATACCTCTTCTCTTTCAAGTCTTTTTGCAACTTGTTCCAAAG

CAAGCGTTTGTGAATCTGCCAGGCAGCCTTCACGAAAATGGCTGATCAACTGTGTGAGGG

GCGTTGGTCCGAAGCCAATCTATCATTCGTTGGGATATCCATCACCCCTGAATTCATCGA

TGAGGCCCTCAGAGGCTTTTGGGAGTCTTTTGCCAAAAGAGCCGTTGGCTACAAAGAGGT

TTACGTTCAGGTTGATCGAGATCCTAAAGTTGCCTGGGAAGGAGCGCTCAATGGTTACCT

CGGCAAATATTCTGGCGTAAAAAATGTTCATTTTGCGCCGAACAACGCCACGATTGGAAA

GCCCCCACGCCATATCGACATTTTGCTTCGGAAAAAATTTAGCGGCGAACTTCTTGCGAC

GCTCGAAGGTGTGGCAATTTCAAACGGTGCAACTAGTTGGCTTGCGCTCGCATGTGTAAA

CCTTCTCTTGAAGGCAAGACAAGAAATTGATGTCTTCTTGTTTGGGGCAGGAAAAGTCGC

GGAGGCTGTGATTCTTGCCCTTAATCATGGTGCGGCTGCAAAAATCAAAACCATGGCAGT

GCTCAGCCAAGCTCCAATTGAAGGTGGGGTTCCAGCTCGATGCCGTCATTGATCGGAAGG

TTATACCAAAAGCCAAACTCGTCATAACAGCAACAAATTCCGAGGAGCTCGGTACTCGAG

GCCGACGAAATTGCGCCAAATGCAGTAACCATATCCCTCGGAAAAGATGAATTGCCCGCC

GCCTACTTCGATCGCCTTTTGAACGCAGAGGGTCTGATTATTGGCGACGATCTGGATGCG

ATCGAATCGCGCAATGTCAATTCCCTGGTGCTTTACTACTCGAAACGTGATTTGAAGCTG

ACAGAACATGGAAGGGATCATTGGATAAAGAACTACGCCAATGTCCTTGCTGATCCAgct

ctcatggaggaacttaagacatgagaggggccagccaacttttcatcggtTGGCCTTGCC

AGCCTAGACttGGCAATGGCCggccgactctacgaaactcttactgcgaagctctccccc

ccccagtagacaaaaccctcttaggattgcgctcttCAGAAGGTGCGCCTCCCCAGTCAC

AACCGAAAAGTCACTCAACTTGGCTCAAGTACCCTACTTAGTTTGTCTCTCTGTCGTGTT

TAGTATCAGCAACAATAACAAATATTGTAAATAACATATATTTATATGGTGATTATATGG

CATGAAAGCGTGACAACGAATATGTTTTCCATAGTTTGATAAACTCAACACCCGAAAAAG

CCGGTGACCAAAGGGTTGGGACCTCTTTGCACGAACTCCTATGGCTGCCAGACTATCTAC

AGAGCTTTTTCGGAAGCTGACGCAGCATCTGATCGCCACACAATCCTCGCGGACGTGGAT

GGCGTGGCTACATGCAAGCAGTTTGACTGAGAGTTTTCGAGTGACTATTTTAAAAGGACA

ATATTTCGTCCCAAGCGATAAAGCTCGCTCACATGACTAAGTCGTTGCTTTAATTCAACT

GCGTGAAAAGATTTAAAGGGCAGAACTGTTTGTGTTTCGTATCCAGTTCACCAGCAATTA

TTGGCTACGCATAAAGCTCATTATCGTCGATTCTTTACCCAAACACTGATGCCATTCCAA

ATGAGTTAGGCCAAAGATGTACTCATGTCGGACAGGCGCGATTTGGACTTCGTGATCCTC

AAGTCTATCGCAGTGCTGCTTTATTACAAGCTATGCTTCCACCCTCCATTATTTGGTCAA

CTGGACCTAGCTTTTGACAAGAATAGGCCAGCGTTCATCTATCTATCTTTCAAAAACATG

CAGTTGCTGCTGCGTAAAGGATGTAACATCGGATTTGGTGTGGAGACGGTGCTCGCTACG

ACTATACTGCCTTACTGTAGTGATCTGTCCCGCCAAGCGATGAGGGGAGCGGCCTGCCTC

TCGTGAGCGTCAATCCATACGGTTGCATGTTCGTTTGTTTCAAAGGCATTCCCAAGAAGG

GTTTCCAGCTTGACAACTTTCTCATCAGTGGGTGTGAACTCGCCGGTTAGTGAGTCACCG

CCCATTTTGGGAACTACCAGGGGGGTGCCAACGACCTCCTCCGTCGACATCTCAGACCAA

TTTCAATTGCCCAACTGCACCGTTCTTTCTATGTTGAAGTCATGGGTGACTATGAAATCT

CCATCTTTGGTCGGTACTATCTCGATCTCGACCATATTGAAAAGCCTGAAAGATTTGCAA

ACTGCCCATACACTGTTTTCCGGCAAGTGTGGCCCATGGAATCCACGGTGAACTATCAAT

TGGCGGTTGCTTGAAGTCTTTCGAGCGATAAGCCAATTGGCGAGGCAGTGTTTCGCGATC

GCATCTTTAAGAGGAAAGTCCGATGCAAATCTCCATGTAGACCCAGTCGTTTCGGCCAAG

GCGGCCTCTAAATCAACTTGAGCAGCAGTTGTGTTAACGGAATCTGTTTTGACTGCCATT

CTGTAAGAAATGTCAGCAAAGGAAAAGCTAGTTAGAGAAGGAGAGCTTGCTCAATAGTGT

GTCTATTCTCAAATGAACTATACTAGCGCAATGAGTTGGTGTTGTTCGCTGATGGGAAAA

AGGAAACGCATCCCCCTCTTCTACAAAATTCtgtgggcccggagggactccgtatgaaag

tgcacagcatgtggacgcatcacCATGCAATTAGCAAGCTTAGCCAATGTCGTGTCTGTC

AGAGTGTGCAGCATGTGAGCCAAGCGCAATTACAATAAAAAAATAATATAAAAACTCTGC

CGAGGTCCGAACCGTCTCAAATGCTTTGAGGGCCATCCAAGCCAAGCGCACCTAACGCCT

AGCACACCATTGTTGCGCCGCTCGAGCATTAATGGTTCGGGACCTGGTGTCATCTGGAAA

CCTATGAAGCATGGACACGG

>ss2463_2

tccaaattTCCCTCACCTTGAAGTATCAATTTGATCTAGAAGCCAATCCAAACTCATTGA

AAGGCCTGTCATCAACCGGTCAACACGGTTCAATGTTTGCTCGCGTGGCGCAGCAATGGT

GTGCTAGGCGTTAGGTGCACCTGGCTTGGATGGCCCTCAAAGCATTTGAGGCAGTTCGGA

CCTCGGCAGAGATTTTATATTATTTTTTTGTTGTAATCGCGCTTGGCCCACATGCTGCAC

ACTCCGATAGACACGGCATTGGCTAAACCCGCTAATTGCGTGGTGATGCGTCCACGTGCT

GCGCACTTTCATGCGGAGTCCCCCCGAGTCCATAGAATAAGAGGGGGACGCGTTTCCTTT

TTCCCATCAGCAAACAACACCAACTCACTGTGCTAGTATAGTTCATCTGAGAACAGACAC

ACTGCTGAGCAAGCTCTCCTTCTCCAACTAGCTTTTTCTTTGCTGACATTTCCTACAGAA

TGGCAGCAAAACAGATTCCGTTAACACAACTACTGCTCAGGTTGATTTAGAGGCCGCCTT

GGCCGAAGCGATCGGGTCTACATGGAGATTTGCATCAGACTTTCCTCTTAAAAATGCAAT

CGCGAAACACTGCCTCGCCAATTGGCTTATCGCTCGAAAGACTTCAAGCAACCACCAATT

GATAGTTCACCGTGAATTCCATGGGCCACACCTGCCCGAAAACAGCGTAGGGGCAGTCTG

CAAATCTCTCAGACTTTTTAATATGGTCGAGATCAACGTAGTACCGACCAAAGATAAAGA

TTTCATAGTCGTCCACGACTTCAACACAGAAAGAACGACGCAGTTAGGCAATTGAAATTG

GTTTGATATGTCGACGAAAGAGGCTGTTGGCACCCCCCTGGTAGTTCCCATAATGGGCGG

TGACTCCCTAACCAGCGAGTTCACACCCACCGATGAGAAAGTTGTCAAGCAAGAAACCCT

TCTCAAGAATGCCTTTGAAACAAACAAACAGGCAACCATACGGATTGACGCTCACGAGAG

GCAGGCCGCTCCCCTCGTCGCTTGGCTGAGCCATAGGCCGGCCTATCACGACAAGACAAT

CGTTATCTTTCATACATATGGATACAACAATGGACACGAGTTCGCCTTGGCGGTCAATCA

AGCCGGCGCTACAAAAAATTGGCGCGAAACTGTAGCGCTAATGCCAAACATTCTGCCGAA

TGAACTCTCTAGGCTCGCGAAGCAATATCCACCAAATTGCACGTCGCATTTTTACGACCT

CGTTGAGGCCAGCAAGAATTGGTTCGCATCTGTCTTCTCCCAGAATATGATGGTGGTCGT

CACTTTCCTCAAGATTGCGGGCAATGGCGAAGGACAGACTAACAAAGTCGTTGATCCAAA

TGGAAATGAGGTTCTGCATCCTAAAGAGAAGGAGGAATATCTCCGCGATTCAGCTTCAAT

CGAAATCGCGAAATGAATCTGCGCCACCCATCCCGGAATCGAGATTGGAGGCGTCACACG

AACTTATGCTTATAAGAGTCGAGGACATCGCTACAAAATCAATCTTTGGACGGGTCAACC

GAAGCGGTGGCCGACCCACGCGACGAAGTACATCTGGGAAGATTTCGCCAAGCCTGGAAA

TGCGCTCAGATGGGGTCAAACGATTATCTCTGATCGCATTGGTGACGACCTTGCAGCTTG

TGTGCTTCAAAGCCGTGGAGAAAATTTTGTCAGCAGTCATGAATATCCTGCTGTTCATGT

CGATTAGATGGGCGATCTGTGTCTTAGTCTAAGCTTCTGCTTAACGCATCTTTGCTACTT

TAATGTATTTTTTTAATGATATTGTTGGAGTTTTGAGCACATCTTAATCGCAAGTAGATA

TTTTTTTTGAATCGGATTCGACTTCAAGCAGATGTACTTCTTTGTCTCTCGATAAATCTA

ATGACCCTGCGAgCCGGCCTCTCGCCTTAAACTAATTTCTGAGAGTTAGGTCGCCGCCTC

TCGTGGTTTCCATGTTCTATGGAAACGGCCGCTCGGGACTTGCCTTCCCGCAACTCATCG

AGCTTGCCTGGCCCGCTCGGGTGGTGGCACAAGCCATCGTTAAATCGTTCGTAATCCTCT

ACTCTCGCATAAATGAGAGGGGAATCCAAAGATGATCACTTTTCAGCTAATTGGAATCTG

CAATCCCAAAAGAGGCAAGCACTATGTGGGGTCCACAACTTATAGAATTTTGGCCTAACC

ATTCTGCATAATGGATCATGACATGGGCCATTTATGGCCGTAAAAACTGGCACAAATTCC

AACCTATTCAGCACAATGAACAAGTTCTTCCAATTGGAATAAAGGAGGTTAAAATTATTC

GAGTAGACATGACGTGCTGGCACGCCACTAGCCTCGCATTGATGCCACTCTTTCTTGCGC

ACACTTATATAATGCTGAGCTAATAATAGAGATAAAAATAGTCAGCTTGTAAACTGGACC

TTTATTACTGCCCCTAATCTCAAGATCAAAGCGACATATACCTCTTCTCTTTCAAGTCTT

TTTGCAACTTGTTCCAAAGCAAGCGTTTGTGAATCTGCCAGGCAGCCTTCACGAAAATGG

CTGATCAACTGTGTGAGGGGCGTTGGTCCGAAGCCAATCTATCATTCGTTGGGATATCCA

TCACCCCTGAATTCATTGATGAGGCCCTCAAAGGCTTTTGGGAGTCTTTTGCCAAAAGAG

CCGTTGGCTACAAAGAGGTTTACGTTCAGGTTGATCGAGATCCTAAAGTTGCCTGGGAAG

GAGCGCTCAATGGTTACCTCGGCAAATATTCTGGCGTAAAAAATGTTCATTTTGCGCCGA

ACAACGCCACGATTGGAAAGCCCCCACGCCATATCGACATTTTGCTTCGGAAAAAATTTA

GCGGCGAACTTCTTGCGACGCTCGAAGGTGTGGCAATTTCAAACGGTGCAACTGGTTGGC

TTGCGCTCGCATGTGTAAACCTTCTCTTGAAGGCAAGACAAGATATTGATGTCTTCTTGT

TTGGGGCAGGAAAAGTCGCCGAGGCTGTGATTCTTGCCCTCAATCATGGTGCGGCTGCAA

AAATCAAAACCATGGCAGTGCTCAGCCAAGCTCCAATTGAAGGTGGGGTTCTAGCTCGAT

GCCGTCATTGATTAGAAGGTTATACCAAAAGCCAAACTCGTCATAACAGCAACAAATTCC

GAGGAGCTCGGTACTCAAGGCCGACGAAATTGCGCCAAATGCAGTAACCATATCCCTCGG

AAAAGATGAATTGCCCGCCGCCTACTTCGATCGCCTTTTGAACGCAGAGGGTCTGATTAT

TGGCGACGATCTGGATGCGACCGAATCGCGCAATGTCAATTCCCTGGTGCTTTACTACTC

GAAACGTGATTTGAAGCTGACAGAACATGGAAGGGATCATTAGATAAAGAACTACGCCAA

TGTCCTTGCTGATCCAGCTCTCATGGAGGAACTTAAGACATGAGAGGGGCCAGCCAACTT

TTCATCGGTTGGCCTTGCCAGCCTAGACTTGGCGATGGCCGGCCGACTCTACGAAACTCT

TACTGCGAAACTCTCCCACCCCCAGTAGACAAAACCCTCTTAGGATTGCGCTCTTCAGAA

GGTGCGCCTCCCCAGTCACAACCGGAAAGTCACTCAACTTGGCTCAAGTACCCTACTTAG

TTTGTCTCTCTGTCGTGTTTAGTATCAGCAACAATAACAAATATTGTAAATAACATATAT

TTATATAGTAATTATATGGCATGTAAGCGTGACAACGATTATGTTTTCCATAGTTTGATA

AACTTAACACCCGAAAAAAGCCGGTGACCAAAGGGTTGGGACCTCTTTGCACGAACTCCT

ATGGCTGCCAGACTATCTACAGAGCTTTTTCGGAAGCTGACGCAGCATCTGATCGCCACA

CAATCCTCGCGGACGTGGATGGCGTGGCTACATGCAAGCAGTTTGACTGAGAGTTTTCGA

GTGACTGTTTTAAAAGGACAATATTTCGTCCCAAGCGATAAAACTCGCTCACATGACTAA

GTCGTTGCTTTAATTCAACTGCGTGAAAAGATTTAAAGGGCAGAACTGTTTGTGTTTCGT

ATCCAGTTCACCGGCAATTATTGGCTACGCATAAAGCTCATTATCGTCGATTCTTTACCC

AAACACTGATGCCATTCCAAATGCGTTGGGCCAAAGATGTACTCATGTCGGACAGGCGCA

ATTTGGACTTCGTGATCCTCAAGTCTATCGCAGTGCTGCTTTATTACAGGCTATGCTTCC

ACCCTCCATTATTTGGTCAACTGGACCTAGCTTTTGACAAGAATAGGCCAGCGTTCATCT

ATCTATCTTTCAAAAACATGCAGTTGCTGCTGTGTAAAGGATGTAACATCGGATTTGGTG

TGGAGACGGTGCTCGCTACGACTATACTGCCTTACTGTAGTGATCTGTCCCGCCAAGCGA

TGAGGGGAGCGGCCTGCCTCTCGTGAGCGTCAATCCATACGGTTGCATGTTCGTTTGTTT

CAAAAGCATTCCCAAGAAGGGTTTCCAGCTTGACAACTTTCTCATCGGTGGGTGTGAACT

CGCCGGTTAGTGAGTCACCGCCCATTTTGGGAACTACCAGGGGGGTGCCAACGACCTCCT

CCGTCGACATCTCAGACCAATTTCAAttGcccAACTGCACCGTtctttctatgttgaagt

catgggcgACTATGAAATCTCCATCTTTGGTCGGTACTACCTCGATCTCGACCATATTGA

AAAGCCTGAAAGATTTGCAAACTGCCCCTACACTGTTTTCTGGCAAGTGTGGCCCATGGA

ATCCACGGTGAGCTATCAATTGGCGGTCGCTTGAAGTCTTTCGAGCGATAAGCCAATTGG

CGAGGCAGTGTTTCGCGATCGCATCTTTAAGAGGAAAGTCCGATGCAAATCTCCATGTAG

ACCCAGTCGTTTCGGCCAAGGCGGCCTCTAAATCAACTTGAGTAGCAGTTGTGTTAACGG

AATCTGTTTTGACTGCCATTCTGTAAGAAATGTCAGCAAAGGAAAAGCTAGTTAGAGAAG

GAGAGCTTGCTCAATAGTGTGTCTATTCTCAAATGAACTATACTAGCGCAATGAGTTGGT

GTTGTTCGCTGATGGGAAAAAGGAAACGCATCCCCCTCTTCTACAAAATTCtgtgggccc

ggagggactccgtatgaaagtgcacagcatgtggacgcatcacCATGCAATTAGCAAGCT

TAGCCAATGTCGTGTCTGTCAGAGTGTGCAGCATGTGAGCCAAGCGCAATTACAATAAAA

AAATAATATAAAAACTCTGCCGAGGTCCGAACCGTCTCAAATGCTTTGAGGGCCATCCAA

GCCAAGCGCACCTAACGCCTAGCACACCATTGTTGCGCCGCTCGAGCATTAATGGTTCGG

GACCTGGTGTCATCTGGAAACCTATGAAGCATGGACACGG

>ss7208_Un

ACAAGCCTCTCCAAATTTCCCTCACCTTGAAGTATCAATTTGATCTAGAAGCCAATCCAA

ACTCATTGAAAGGCCTGTCATCAACCGGTCAACACGGTTCAATGTTTGCTCGCGTGGCGC

AGCAATGGTGTGCTAGGCGTTAGGTGCGCCTGGCTTGGATGGCCCTCAAAGCATTTGAGG

CAGTTCGGACCTCGGCAGAGATTTTATATTATTTTTTTGTTGTAATCGCGCTTGGCCCAC

ATGCTGCACACTCCGATAGACACGGCATTGGCTAAACCCGCTAATTGCGTGGTGATGCGT

CCACGTGCTGCGCACTTTCATGCGGAGTCCCCCCGAGTCCATAGAATAAGAGGGGGACGC

GTTTCCTTTTTCCCATCAGCAAACAACACCAACTCACTGTGCTAGTATAGTTCATCTGAG

AACAGACACACTGCTGAGCAAGCTCTCTTTCTCCAACTAGCTTTTTCTTTGCTGACATTT

CCTACAGAATGGCAGCAAAACAGATTCCGTTAACACAACTACTGCTCAGGTTGATTTAGA

GGCCGCCTTGGCCGAAGCGATCGAGTCTACATGGAGATTTGCATCAGACTTTCCTCTTAA

AAATGCAATCGCGAAACACTGCCTCGCCAATTGGCTTATCGCTCGAAAGACTTCAAGCAA

CCACCAATTGATAGTTCACCGTGAATTCCATGGGCCACACCTGCCGGAAAACAGCGTAGG

GGCAGTCTGCAAATCTCTCAGACTTTTTAATATGGTCGAGATCAACGTAGTACCGACCAA

AGATAAAGATTTCATAGTCGTCCACGACTTCAACACAGAAAGAACGACGCAGTTAGGCAA

TTGAAATTGGTTTGATATGTCGACGAAAGAGGCTGTTGGCACCCCCTTGGTAGTTCCCAT

AATGGGCGGTGACTCCCTAACCAGCGAGTTCACACCCACCGATGAGAAAGTTGTCAAGCA

AGAAACCCTTCTCAAGAATGCCTTTGAAACAAACAAACAGGCAACCATACGGATTGACGC

TCACGAGAGGCAGGCCGCTCCCCTCGTCGCTTGGCTGAGCCATAGGCCGGCCTATCACGA

CAAGACAATCGTTATCTTTCATACATATGGATACAAAAATGGACACGAGTTCGCCTTGGC

GGTCAATCAAGCCGGCGCTACAAAAAATTGGCGCGAAACTGTAGCGCTAATGCCAAACAT

TCTGCCGAATGAACTCTCTAGGCTCGCGAAGCAATATCCACCAAATTGCACGTCGCATTT

TTACGACCTCGTTGAGGCCAGCAAGAATTGGTTCGCATCTGTCTTCTCCCAGAATATGAT

GGTGGTCGTCACTTTCCTCAAGATTGCGGGCACTGGCGAAGGACAGACTAACAAAGTCGT

TGATCCAAATGGAAATGAGGTTCTGCATCCTAAAGAGAAGGAGGAATATCTCCGCGATTC

AGCTTCAATCGAAATCGCGAAATGAATCTGCACCACCCATCCCGGAATCGAGATTGGAGG

CGTCACACGAACTTATGCTTATAAGAGTCGAGGACATCGCTACAAAATCAATCTTTGGAC

GGGTCAACCGAAGCGGTGGCCGACCCACGCGACGAAGTACATCTGGGAAGATTTCGCCAA

GCCTGGAAATGCGCTCAGATGGGGTCAAACGATTATCYCTGATCGCATTGGTGACGACCT

TGCAGCTTGTGTGCTTCAAAGCCGTGGAGAAAATTTTGTCAGCAGTCATGAATATCCTGC

TGTTCATGTCGATTAGATGGGCGATCTGTGTCTTAGTCTAAGCTTCCGCTTAACGCATCT

TTGCTACTTTAATGTATTTTTTTAATGATATTGTTGGAGTTTTGAGCACATCTTAATCGC

AAGTAGATATTTTTTTTGAATCGGATTCGACTTCAAGCAGATGTACTTCTTTGTCTCTCG

ATAAATCTAATGACCCTGCGAGCCGGCCTCTCGCCTTAAACTAATTTCTGAGAGTTAGGT

CGGCGCCTCTCGTGGTTTCCATGTTCTATGGAAACGGCCGCTCGGGACTTGCCTTCCCGC

AACTCATCGAGCTTGCCTGGCCCGCTCGGGTGGTGGCACAAGCCATCGTTAAATCGTTCG

TAATCCTCTACTCTCGCATAAATGAGARGGGGAATCCAAAGATGATCACTTTTCAGCTAA

TTGGAATCTGCAATCCCAAAAGAGGCAAGCACTATGTGGGGTCCACAACTTATAGAATTT

TGGCCTAACCATTCTGCATAATGGATCATGACATGGGCCATTTATGGCCACAAAAACTGG

CACAAATTCCAACCTATTCAGCACAATGAACAAGTTCTTCCAATTGGAATAAAGGAGGTT

AAAATTATTCGAGTAGACATGACGTGCTGGCACGCCACTAGCCTCGCATTGATGCCACTC

TTTCTTGCGCACACTTATATAATGCTGAGCTAATAATAGAGATAAAAATAGTCAGCTTGT

AAACTGGACCTTTATTACTGCCCCTAATCTCAAGATCAAAGCGACATATACCTCTTCTCT

TTCAAGTCTTTTTGCAACTTGTTCCAAAGCAAGCGTTTGTGAATCTGCCAGGCAGCCTTC

ACGAAAATGGCTGATCAACTGTGTGAGGGGCGTTGGTCCGAAGCCAATCTATCATTCGTT

GGGATATCCATCACCCCTGAATTCATCGATGAGGCCCTCAGAGGTTTTTGGGAGTCTTTT

GCCAAAAGAGCCGTTGGCTACAAAGAGGTTTACGTTCAGGTTGATCGAGATCCTAAAGTT

GCCTGGGAAGGAGCGCTCAATGGTTACCTCGGCAAATATTCTGGCGTAAAAAATGTTCAT

TTTGCGCCGAACAACGCCACGATTGGAAAGCCCCCACGCCATATCGACATTTTGCTTCGG

AAAAAATTTAGCGGCGAACTTCTTGCAACGCTCGAAGGTGTGGCAATTTCAAACGGTGCA

ACTGGTTGGCTTGCGCTCGCATGTGTAAACCTTCTCTTGAAGGCAAGACAAGATATTGAT

GTCTTCTTGTTTGGGGCAGGAAAAGTCACCGAGGCTGTGATTCTTGCCCTCAATCATGGT

GCGGCTGCAAAAATCAAAACCATGGCAGTGCTCAACCAAGCTCCAATTGAAGGTGGGGTT

CCAGCTCGATGCCATCATTGATCGGAAGGTTATACCAAAAGCCAAACTCGTCATAATAGC

AACAAATTCTGAGGAGCTCGGTACTCGAGGCCGACGAAATTGCGCCAAATGCAGTAACCA

TATCCCTCGGAAAAGATGAATTGCCCGCCGCCTACTTCGATCGCCTTTTGAACGCAGAGG

GTCTGATTATTGGCGACGATCTGGATGCGATCGAATCGCGCAATGTCAATTCCCTGGTGC

TTTACTACTCAAAACGTGATTTGAAGCTGACAGAACATGGAAGGGATCATTGGATAAAGA

ACTACGCCAATGTCCTTGCTGATCCAGCTCTCATGGAGGAACTTAAGACATGAGAGGGGC

CAGCCAACTTTTCATCGGTTGGCCTTGCCAGCCTAGACTTGGCGATGGCCGGCCGACTCT

ACGAAACTCTTACTGCGAAACTCTCCCACCCCCAGTAGACAAAACCCTCTTAGGATTGCG

CTCTTCAGAAGGTGCGCCTCCCCAGTCACAACCGGAAAGTCACTCAACTTGGCTCAAGTA

CCCTACTTAGTTTGTCTCTCTGTTGTGTTTAGTATCAGCAACAATAACAAATATTGTAAA

TAACATATATTTATATGGTAATTATATGGCATGTAAGCGTGACAACGATTATGTTTTCCA

TAGTTTGATAAACTCAACACCCGAAAAAGCCGGTGACCAAAGGGTTGGGACCTCTTTGCA

CGAACTCCTATGGCTGCCAAACTATCTACAGAGCTTTGACGCAGCATCTGATCGCCACAC

AATCCTCGCGGACGTGGATGGCGTGGCTACATGCAAGCAGTTTGACTGAGAGTTTTCGAG

TGACTATTTTAAAAGGACAATATTTCGTCCCAAGCGATAAAACTCGCTCACATGACTAAG

TCGTTGCTTTAATTCAACTGCGTGAAAAGATTTAAAGGGCAGAACTGTTTGTGTTTCGTA

TCCAGTTCACCGGCAATTATTGGCTACGCATGAAGCTCATTATCGTCGATTCTTTACCCA

AACACTGATGCCATTCCAAATGAGTTGGGCCAAAGATGTACTCATGTCGGACAGGCGCGA

TTTGGACTTCGTGATCCTCAAGTCTATCGCAGTGCTGCTTTATTACAGGCTATGCTTCCA

CCCTCCATTATTTGGTCAACTGGACCTAGCTTTTGACAAGAATAGGCCAGCGTTCATCTA

TCTATCTTTCAAAAACATGCAGTTGCTGCTGCGTAAAGGATGTAACATCGGATTTGGTGT

GGAGACGGTGCTCGCTACGACTATACTGCCTTACTGTAGTGATCTGTCCCGCCAAGCGAT

GAGGGGAGCGGCCTGCCTCTCGTGAGCGTCAATCCATACGGTTGCATGTTCGTTTGTTTC

AAAGGCATTCCCAAGAAGGGTTTCCAGCTTGACAACTTTCTCATCGGTGGGTGTGAACTC

GCCGGTTAGTGAGTCACCGCCCATTTTGGGAACTACCAGGGGGGTGCCAACGACTTCCTC

CGTCGACATCTCAGACCAATTTCAATTGCCCAACTGCACCGTTCTTTCTATGTTGAAGTC

ATGGGCGACTATGAAATCTCCATCTTTGGTCGGTACTACCTCGATCTCGACCATATTGAA

AAGCCTGAAAGATTTGCAAACTGCCCCTACACTGTTTTCCGGCAAGTGTGGCCCATGGAA

TCCACGGTGAACTATCAATTGGCGGTCGCTTGAAGTCTTTCGAGCGATAAGCCAATTGGC

GAGGCAGTGTTTCGCGATCGCATCTTTAAGAGGAAAGTCCGATGCAAATCTCCATGTAGA

CCCAGTCGTTTCGGCCAAGGCGGCCTCTAAATCAACTTGAGCAGCAGTTGTGTTAACGGA

ATCTGTTTTGACTGCCATTCTGTAAGAAATGTCAGCAAAGGAAAAGCTAGTTAGAGAAGG

AGAGCTTGCTCAATAGTGTGTCTATTCTCAAATGAACTATACTAGCGCAATGAGTTGGTG

TTGTTCGCTGATGGAAAAAAGGAAACGCATCCCCCTCTTCTACAAAATTCTGTGGGCCCG

GAGGGACTCCGTATGAAAGTGCACAGCATGTGGACGCATCACCATGCAATTAGCAAGCTT

AGCCAATGTCGTGTCTGTCAGAGTGTGCAGCATGTGAGCCAAGCGCAATTACAATAAAAA

AATAATATAAAAACTCTGCCGAGGTCCGAACCGTCTCAAATGCTTTGAGGGCCATCCAAG

CCAAGCGCACCTAACGCCTAGCACACCATTGTTGCGCCGCTCGAGCATTAATGGTTCGGG

ACCTGGTGTCATCTGGAAACCTATGAAGCATGGACACGGCAAAATCAGCGCCGTGGCGGT

GTCAGAC

>ss7207_Un

TCTAGAAGCCAAKCCAAACTCATTGAAAGGCCTGTCATCAACCGGTCAACACGGTTCAAT

GTTTGCTCGCGTGGCACAGCAATGGTGTGCTAGGCGTTAGGTGCGCCTGGCTTGGATGGC

CCTCAAAGCATTTGAGGCAGTTCGGACCTCGGCAGAGATTTTATATTATTTTTTTGTTGT

AATCGCGCTTGGCCCACATGCTGCACACTCCGATAGACACGGCATTGGCTAAACCCGCTA

ATTGCGTGGTGATGCGTCCACGTGCTGCGCACTTTCAGGCGGAGTCCCCCCGATTCCATA

GAATAAGAGGGGGACGCGTTTCCTTTTTCCCATCAGCAAACAACACCAACTCACTGTGCT

AGTATAGTTCATCTGAGAACAGACACACTGCTGAGCAAGATCTCCTTCTCCAACTAGCTT

TTTCTTTGCTGACATTTCCTACAGAATGGCAGCAAAACAGATTCCGTTAACACAACTACT

GCTCAGGTTGATTTAGAGGCCGCCTTGGCCGAAGCGATCGGGTCTACATGGAGATTTGCA

TCAGACTTTCCTCTTAAAAATGCAATCGCGAAACACTGCCTCGCCAATTGGCTTATCGCT

CGAAAGACTTCAAGCAACCACCAATTGATAGTTCACCGTGAATTCCATGGGCCACACCTG

CCGGAAAACAGCGTAGGGGCAGTCTGCAAATCTCTCAGACTTTTTAATATGGTCGAGATC

AACGTAGTACCGACCAAAGATAAAGATTTCATAGTCGTCCACGACTTCAACACAGAAAGA

ACGACGCAGTTAGGCAATTGAAATTGGTTTGATATGTCGACGAAAGAGGCTGTTGGCACC

CCCCTGGTAGTTCCCATAATGGGCGGTGACTCCCTAACCAGCGAGTTCACACCCACCGAT

GAGAAAGTTGTCAAGCAAGAAACCCTTCTCAAGAATGCCTTTGAAACAAACAAACAGGCA

ACCATACGGATTGACGCTCACGAGAGGCAGGCCGCTCCCCTCGTCGCTTGGCTGAGCCAT

AGGCCGGCCTATCAYGACAAGACAATCGTTATCTTTCATACATATGGATACAACAATGGA

CACGAGTTCGCCTTGGCGGTCAATCAAGCCGGCGCTACAAAAAATTGGCGCGAAACTGTA

GCGCTAATGCCAAACATTCTGCCGAATGAACTCTCTAGGCTCGCGAAGCAATATCCACCA

AATTGCACGTCGCATTTTTACGACCTCGTTGAGGCCAGCAAGAATTGGTTCGCATCTGTC

TTCTCCCAGAATATGATGGTGGTCGTCACTTTCCTCAAGATTGCGGGCAATGGCGAAGGA

CARACTAACAAAGTCGTTGATCCAAATGGAAATGAGGTTCTGCATCCTAAAGAGAAGGAG

GAATATCTCCGCGATTCAGCTTCAATCGAAATCGCGAAATGAATCTGCGCCACCCATCCC

GGAATCGAGATTGGAGGCGTCACACGAACTTATGCTTATAAGAGTCGAGGACATCGCTAC

AAAATCAATCTTTGGACGGGTCAACCGAAGCGGTGGCCGACCCACGCGACAAAGTACATC

TGGGAAGATTTCGCCAAGCCTGGAAATGCGCTCAGATGGGGTCAAACGATTATCTCTGAT

CGCATTGGTGACGACCTTGCAGCTTGTGTGCTTCAAAGCTGTGGAGAAAATTTTGTCAGC

AGTCATGAATATCYTGCTGTTCATGTCGATTAGATGGGCGATCTGTGTCTTAGTCTAAGC

TTCCGCTTAACGCATCTTTGCTACTTTAATGTATTTTTTTAATGATATTGTTGGAGTTTT

GAGCACATCTTAATCGCAAGTAGATATTTTTTTTGAATCGGATTTGACTTCAAGCAGATG

TACTTCTTTGTCTCTCGATAAATCTAATGACCCTGCGACCGGCCTCTCGCCTTAAACTAA

TTTCTGAGAGTTAGGTCGCCGCCTCTCGTGGTTTCCATGTTCTATGGAAACGGCCGCTCG

GGACTTGCCTTCCCGCAACTCATCGAGCTTGCCTGGCCCGCTCGGGTGGTGGCACAAGCC

ATCGTTAAATCGTTCGTAATCCTCTACTCTCGCATAAATGAGAGGGGAATCCAAAGATGA

TCACTTTTCAGCTAATTGGAATCTGCAATCCCAAAAGAGGCAAGCACTATGTGGGGTCCA

CAACTTATAGAATTTTGGCCTAACCATTCTGCATAATGGATCATGACATGGGCCATTTAT

GGCCGTAAAAACTGGCACAAATTCCAACCTATTCAGCACAATGAACAAGTTCTTCCAATT

GGAATAAAGGAGGTTAAAATTATTCGAGTAGACATGACGTGCTGGCACGCCACTAGCCTC

GCATTGATGCCACTCTTTCTTGCGCACACTTATATAATGCTGAGCTAATAATAGAGATAA

AAATAGTCAGCTTGTAAACTGGACCTTTATTACTGCCCCTAATCTCAAGATCAAAGCGAC

ATATACCTCTTCTCTTTCAAGTCTTTTTGCAACTTGTTCCAAAGCAAGCGTTTGTGAATC

TGCCAGGCAGCCTTCACGAAAATGGCTGATCAACTGTGTGAGGGGCGTTGCTCCGAAGCC

AATCTATCATTCGTTGGGATATCCATCACCCCTGAATTCATCGATGAGGCCCTCAGAGGC

TTTTGGGAGTCTTTTGCCAAAAGAGCCGTTGGCTACAAAGAGGTTTACGTTCAGGTTGAT

CGAGATCCTAAAGTTGCCTGGGAAGGAGCGCTCAATGGTTACCTCGGCAAATATTCTGGC

GTAAAAAATGTTCATTTTGCGCCGAACAACGCCACGATTGGAAAGCCCCCACGCCATATC

GACATTTTGCTTCGGAAAAAATTTAGCGGCGAACGTCTTGCGACGCTCGAAGGTGTGGCA

ATTTCAAACGGTGCAACTGGTTGGCTTGCGCTCGCATGTGTAAACCTTCTCTTGAAGGCA

AGACAAGATATTGATGTCTTCTTGTTTGGGGCAGGAAAAGTTGCCGAGGCTGTGATTCTT

GCCCTCAATCATGGTGCGGCTGCAAAAATCAAAACCATGGCAGTGCTCAGCCAAGCTCCA

ATTGAAGGTGGGGTTCCAGCTCGATGCCATCATTGATCGGAAGGTTATACCAAAAGCCAA

ACTCGTCATAACAGCAACAAATTCCGAGGAGCTCGGTACTCGAGGCCGACGAAATTGCGC

CAAATGCAGTAACCATATCCCTCGGAAAAGATGAATTGCCCGCCGCCTACTTCGATCGCC

TTTTGAACGCAGAGGGTCTGATTATTGGCGACGATCTGGATGCGATCGAATCGCACAATG

TCAATTCCCTGGTGCTTTACTACTCGAAACGTGATTTGAAGCTGACAGAACATGGAAGGG

ATCATTGGATAAAGAACTACGCCAATGTCCTTGCTGATCCAGCTCTCATGGAGGAACTTA

AGACATGAGAGGGGCCAGCCAACTTTTCATCGGTTGGCCTTGCCAGCCTAGACTTGGCGA

TGGCCGGCCGACTCTACGAAACTCTTACTGCGAAACTCTCCCACCCCCAGTAGACAAAAC

CCTCTTAGGATTGCGCTCTTCAGAAGGTGCGCCTCCCCAGTCACAACCGGAAAGTCACTC

AACTTGGCTCAAGTACCCTACTTAGTTTGTCTCTCTGTCGTGTTTAGTATCAGCAACAAT

AACAAATATTGTAAATAACATATATTTATATGGTAATTATATGGCATGTAAGCGTGACAA

CGATTATGTTTTCCATAGTTTGATAAACTCAACACCCGAAAAAGCCGGTGACCAAAGGGT

TGGGACCTCTTTGCACGAACTCCTATGGCTGCCAGACTATCTACAGAGCTTTTTCGGAAG

CTGACGCAGCATCTGATCGCCACACAATCCTCGCGGACGTGGATGGCGTGGCTACATGCA

AGCAGTTTGACTGAGAGTTTTCGAGTGACTGTTTTAAAAGGACAATATTTCGTCCCAAGC

GATAAAACTCGCTCACATGACTAAGTCGTTGCTTTAATTCAACTGCGTGAAAAGATTTAA

AGGGCAGAACTGTTTGTGTTTCGTATCCAGTTCACCGGCAATTATTGGCTACGCATAAAG

CTCATTATCGTCGATTCTTTACCCAAACACTGATGCCATTCCAAATGCGTTGGGCCAAAG

ATGTACTCATGTCGGACAGGCGCAATTTGGACTTCGTGATCCTCAAGTCTATCGCAGTGC

TGCTTTATTACAGGCTATGCTTCCACCCTCCATTATTTGGTCAACTGGACCTAGCTTTTG

ACAAGAATAGGCCAGCGTTCATCTATCTATCTTTCAAAAACATGCAGTTGCTGCTGCGTA

AAGGATGTAACATCGGATTTGGTGTGGAGACGGTGCTCGCTACGACTATACTGCCTTACT

GTAGTGATCTGTCCCGCCAAGCGATGAGGGGAGCGGCCTGCCTCTCGTGAGCGTCAATCC

ATACGGTTGCATGTTCGTTTGTTTCAAAAGCATTCCCAAGAAGGGTTTCCAGCTTGACAA

CTTTCTCATCGGTGGGTGTGAACTCGCCGGTTAGTGAGTCACCGCCCATTTTGGGAACTA

CCAGGGGGGTGCCAACGACCTCCTCCGTCGACATCTCAGACCAATTTCAATTGCCCAACT

GCACCGTTCTTTCTATGTTGAAGTCATGGGCGACTATGAAAYCTCCATCTTTGGTCGGTA

CTACCTCGATCTCGACCATATTGAAAAGCCTGAAAGATTTGCAAACTGCCCCTACACTGT

TTTCCGGCAAGTGTGGCCCATGGAATCCACGGTGAACTATCAATTGGCGGTCGCTTGWAG

TCTTTCGAGCGATAAGCCAATTGGCGAGGCAGTGTTTCGCGATCGCATCTTTAAGAGGAA

AGTCCGATGCAAATCTCCATGTAGACCCAGTCGTTTCGGCCAAGGCGGCCTCTAAATCAA

CTTGAGCAGCAGTTGTGTTAACGGAATCTGTTTTGACTGCCATTCTGTAAGAAATGTCAG

CAAAGGAAAAGCTAGTTAGAGAAGGAGAGCTTGCTCAATAGTGTGTCTATTCTCAAATGA

ACTATACTAGCGCAATGAGTTGGTGTTGTTCGCTGATGGGAAAAAGGAAACGCATCCCCC

TCTTCTACAAAATTCTGTGGGCCCGGAGGGACTCCGTATGAAAGTGCACAGCATGTGGAC

GCATCACCATGCAATTAGCAAGCTTAGCCAATGTCGTGTCTGTCAGAGTGTGCAGCATGT

GAGCCAAGCGCAATTACAATAAAAAAATAATATAAAAACTCTGCCGAGGTCCGAACCGTC

TCAAATGCTTTGAGGGCCATCCAAGCCAAGCGCACCTAACGCCTAGCACACCATTGTTGC

GCCGCTCGAGCATTAATGGTTYGGGACCTGGTGTCATCTGGAAACCTATGAAGCATAGAC

ACGGCAAAATCAGCRCCGTGGCGGTGTCAGACACGAGGACACGTCGGGGAC

>sa0667_2

tgaagtatcAATTTGATCTAGAAGCCAATCCAAACTCATTGAAAGGCCTGTCATCAACCG

GTCAACACGGTTCAATGTTTGCTCGCGTGGCGCAGCAATGGTGCGCTAGGCGTTAGGTGC

GCCTGGCTTGGATGGCCCTCAAAGCATTTGAGGCAGTTCGGACCTCGGCAGAGATTTTAT

ATTATTTTTTTGTTGTAATCGCGCTTGGCCCACATGCTGCACACTCCGATAGACACGGCA

TTGGCTAAACCCGCTAATTGCGTGGTGATGCGTCCACGTGCTGCGCACTTTCATGCGGAG

TCCCCCCGAGTCCATAGAATAAGAGGGGGACGCATTTCCTTTTTCCCATCAGCAAACAAC

ACCAACTCACTGTGCTAGTATAGTTCATCTGAGAACAGACACACTGCTGAGCAAGCTCTC

CTTCTCTAACTAGCTTTTTCTTTGCTGACATTTCCTACAGAATGGCAGCAAAACAGATTC

CGTTAACACAACTACTGCTCAGGTTGATTTAGAGGCCGCCTTGGCCGAAGCGaTCGggtc

tACATGGAGAtttgcatcagactttcctcttAAAATGCAATCGCGAAACACTGCCTCGCC

AATTGGCTTATCGCTCGAAAGACTTCAAGCAACCACCAATTGATAGTTCACCGTGAATTC

CATGGGCCACACCTGCCGGAAAACAGCGTAGGGGCAGTCTGCAAATCTCTCAGACTTTTT

AATATGGTCGAGATCAACGTAGTACCGACCAAAGATAAAGATTTCATAGTCGTCCACGAC

TTCAACACAGAAAGAATGACGCAGTTAGGCAATTGAAATTGGTTTGACATGTCGACGAAA

GAGGCTGTTGGCACCCCCCTGGTAGTTCCCATAATGGGCGGTGACTCCCTAACCAGCGAG

TTCACACCCACCGATGAGAAAGTTGTCAAGCAAGAAACCCTTCTCAAGAATGCCTTTGAA

ACAAACAAACAGGCAACCATACGGATTGATGCTCACGAGAGGCAGGCCGCTCCCCTCGTC

GCTTGGCTGAGCCATAGGCCGGCCTATCACGACAAGACAATCGTTATCTTTCATACATAT

GGATACAACAATGGACACGAGTTCGCCTTGGCGGTCGATCAAGCCGGCGCTACAAAAAAT

TGGCGCGAAACTGTAGCGCTAATGCCAAACATTCTGCCGAATGAACTCTCTAGGCTCGCG

AAGCAATATCCACCAAATTGCACGTCGCATTTTTACGACCTCGTTGAGGCCAGCAAGAAT

TGGTTCGCATCTGTCTTCTCCCAGAATATGATGGTGGTCGTCACTTTCCTCAAGATTGCG

GGCACTGGCAAAGGACAGACTAACAAAGTCGTTGATCCAAATGGAAATGAGGTTCTGCAT

CCTAAAGAGAAGGAGGAATATCTCCGCGATTCAGCTTCAATCGAAATCGCGAAATGAATC

TGCGCCACCCATCCCGGAATCGAGATTGGAGGCGTCACACGAACTTATGCTTATAAGAGT

CGAGGACATCGCTACAAAATCAATCTTTGGACGGGTCAACCGAAGCGGTGGCCGACCCAC

GCGACGAAGTACATCTGGGAAGATTTCGCCAAGCCTGGAAATGCGCTCAGATGGGGTCAA

ACGATTATCTCTGATCGCATTGGTGACGACCTTGCAGCTTGTGTGCTTCAAAGCCGTGGA

GAAAATTTTGTCAGCAGTCATGAATATCCTGCTGTTCATGTCGATTAGATGGGCGATCTG

TGTCTTAGTCTAAGCTTCCGCTTAACGCATCTTTGCTACTTTAATGTATTTTTTTAATGA

TATTGTTGGAGTTTTGAGCACATCTTAATCGCAAGTAGATATTTTTTTTGAATCGGATTC

GACTTCAAGCAGATGTACTTCTTTGTCTCTCGATAAATCTAATGACCCTGCGAGCCAGCC

TCTCGCCTTAAACTAATTTCTGAGAGTTAGGTCGGCGCCTCTCGTGGTTTCCATGTTCTA

TGGAAACGGCCGCTCGGGACTTGCCTTCCCGCAACTCATCGAGCTTGCCTGGCCCGCTCA

GGTGGTGGCACAAGCCATCGTTAAATCGTTCGTAATCCTCTACTCTCGCATAAATGAGAA

GGGAATCCAAAGATGATCACTTTTCAGCTAATTGGAATCTGCAATCCCAAAAGAGGCAAG

CACTATGTGGGGTCCACAACTTATAGAATTTTGGCCTAACCATTCTGCATAATGGATCAT

GACATAGGCCATTTATGGCCGCAAAAACTGGCACAAATTCCAACCTATTCAGCACAATGA

ACAAGTTCTTCCAATTGGAATAAAGGAGGTTAAAATTATTCAAGTAGACATGACGTGCTG

GCACGCCACTAGCCTCGCATTGATGCCACTCTTTCTTGCGCACACTTATATAATGCTGAG

CTAATAATAGAGATAAAAATAGTCAGCTTGTAAACTGGACCTTTATTACTGCCCCTAATC

TCAAGATCAAAGCAACATATACCTCTTCTCTTTCAAGTCTTTTTGCAACTTGTTCTAAAG

CAAGCGTTTGTGAATCTGCCAAGCAGCCTTCACGAAAATGGCTGATCAACGGTGTGAGGG

GCGTTGGTCCGAAGCCAATCTACCATTCGTTGGGATATCCATCACCCCTGAATTCATCGA

TGAGGCCCTCAGAGGCTTTTGGGAGTCTTTTGCCAAAAGAGCCGTTGGCTACAAAGAGGT

TTACGTTCAGGTTGATCGAGATCCTAAAGTTGCCTGGGAAGGAGCGCTCAATGGTTACCT

CGGCAAATATTCTGGCGTAAAAAATGTTCATTTTGCGCCGAACAACGCCACGATTGGAAA

GCCCCCACGCCATATTGACATTTTGCTTCGGAAAAAATTTAGCGGCGAACTTCTTGCGAT

GCTCGAAGGTGTGGCAATTTCAAACGGTGCAACTGGTTGGCTTGCGCTCGCATGTGTAAA

CCTTCTCTTGAAGGCAAGACAAGATATTGATGTCTTCTTGTTTGGGGCAGGAAAAGTCGC

CGAGGCTGTGATTCTTGCCCTCAATCATGGTGCGGCTGCAAAAATCAAAACCATGGCAGT

GCTCAGCCAAGCTCCAATTGAAGGTGGGGTTCCAGCTCGATGcCGTCATTgatcggaagg

ttataccaaaagccaaactcgtcataacagcaacaaattccgaggagctcggtactcgag

gccgacgaaattgcgccaaatgcagtaaccatatccctcggaaaagatgaattgcccgcc

gcctacttcgatcgccttttgaacgcagagggtctgattattggcgacgatctgGATGTG

ATCGAATCGCGCAATGTCAATTCCCTGGTGCTTTACTACTCGAAACGTGATTTGAAACTG

ACAGAACATGGAAGGGATCATTGGATAAAGAACTACGCCAATGTCCTTGCTGATCCAGCT

CTCATGGAGGAACTTAAGACATGAGAGGGGCCAGCCAACTTTTCATCGGTTGGCCTTGCC

AGCCTAGACTTGGCGATGGCCGGCCGACTCTACGAAACTCTTACTGCGAAACTCTCCCAC

CCCCAGTAGACAAAACCCTCTTAGGATTGCGCTCTTCAGAAGGTGCGCCTCCCCAGTCAC

AACCGGAAAGTCACTCAACTTGGCTCAAGTACCCTACTTAGTTTGTCTCTCTGTCGTGTT

TAGTATCAGCAACAATAACAAATATTGTAAAttaacatatatttatatagtAATTATATG

GCATGTAAGCGTGACAACGATTATGTTTTCCATAGTTTGATAAACTCAACACCCGAAAAA

GCCGGTGACCAAAGGGTTGGGACCTCTTTGCACGAACTCCTATGGCTGCCAGACTATCTA

CAGAGCTTTTTCGGAAGCTGACGCAACATCTGATCGCCACACAATCCTCGCGGACGTGGA

TGGCGTGGCTACATGCAAGCAGTTTGACTGAGAGTTTTCGAGTGACTATTTTAAAAGGAC

AATATTTCGTCCCAAGCGATAAAACTCGCTCACATGACTAAGTCGTTGCTTTAATTCAAC

TGCGTGAAAAGATTTAAAGGGCAGAACTGTTTGTGTTTCGTATCCAGTTCACCGGCAATT

ATTGGCTACGCATGAAGCTCATTATCGTCGATTCTTTACCCAAACACTGATGCCATTCCA

AATGAGTTGGGCCAAAGATGTACTCATGTCGGACAGGCACGATTTGGACTTCGTGATCCT

CAAGTCTATCGCAGTGCTGCTTTATTACAGGCTATGCTTCCACCCTCCATTATTTGGTCA

ACTGGACCTAGCTTTTGACAAGAATAGGCCAGCGTTCATCTATCTATCTTTCAAAAACAT

GCAGTTGCTGCTGCGTAAAGGATGTAACATCGGATTTGGTGTGGAGACGGTGCTCGCTAC

GACTATACTGCCTTACTGTAGTGATCTGTCCCGCCAAGCGATGAGGGGAGCGGCCTGCCT

CTCGTGAGCGTCAATCCATACGGTTGCATGTTCGTTTGTTTCAAAGGCATTCCCAAGAAG

GGTTTCCAGCTTGACAACTTTCTCATCGGTGGGTGTGAACTCGCCGGTTAGTGAGTCACC

GCCCATTTTGGGAACTACCAGGGGGGTGCCAACGACCTCCTCCGTCGACATCTCAGACCA

ATTTCAATTGCCCAACTGCACCGTTCTTTCTATGTTGAAGTCATGGGCGACTATGAAATC

TCCATCTTTGGTCGGTACTACCCCGATCTCGACCATATTGAAAAGCCTGAAAGATTTGCA

AACTGCCCCTACACTGTTTTCCGGCAAGTGTGGCCCATGGAATCCACGGTGAACTATCAA

TTGGCGGTCGCTTGAAGTCTTTCGAGCGATAAGCCAATTGGCGAGGCAGTGTTTCGCGAT

CGCATCTTTAAGAGGAAAGTCCGATGCAAATCTCCATGTAAACCCAGTCGTTTCGGCCAA

GGCGGCCTCTAAATCAACTTGAGCAGCAGTTGTGTTAACGGAATCTGTTTTGACTGCCAT

TCTGTAAGAAATGTCAGCAAAGGAAAAGCTAGTTAGAGAAGGAGAGCTTGCTCAATAGTG

TGTCTATTCTCAAATGAACTATACTAGCGCAATGAGTTGGTGTTGTTCGCTGATGGGAAA

AAGGAAACGCATCCCCCTCTTCTACAAAATTCTGTGGGCCCGGAGGGACTCCGTATGAAA

GTGCACAGCATGTGGACGCATCACCATGCAATTAGCAAGCTTAGCCAATGTCGTGTCTGT

CAGAGTGTGCAGCATGTGAGCCAAGCGCAATTACAATAAAAAAATAATATAAAAACTCTG

CTGAGGTCCGAACCGTCTCAAATGCTTTGAGGGCCATCCAAGCCAAGCGCACCTAACGCC

TAGCACACCATTGTTGCGCCGTTCGAGCATTAATGGTTCGGGACCTGGTGTCATCTGGAA

ACCTATGAAGCATGGACAcggcaaaatc

>sa0667_1

agtatcaatttgatctagaagccAATCCAAACTCATTGAAAGGCCTGTCATCAACCGGTC

AACACGGTTCAATGTTTGCTCGCGTGGCGCAGCAATGGTGTGCTAGGCGTTAGGTGCGCC

TGGCTTGGATGGCCCTCAAAGCATTTGAGGCAGTTCGGACCTCGGCAGAGATTTTATATT

ATTTTTTTGTTGTAATCGCGCTTGGCCCACATGCTGCACACTCCGATAGACACGGCATTG

GCTAAACCCGCTAATTGCGTGGTGATGCGTCCACGTGCTGCGCACTTTCATGCGGAGTCC

CCCCGAGTCCATAGAATAAGAGGGGGACGCGTTTCCTTTTTTCCATCAGCAAACAACACC

AACTCACTGTGCTAGTATAGTTCATCTGAGAACAGACACACTGCTGAGCAAGCTCTCCTT

CTCCAACTAGCTTTTTCTTTGCTGACATTTCCTACAGAATGGCAGCAAAACAGATTCCGT

TAACACAACTACTGCTCAGGTTGATTTAGAGGCCGCCTTGGCCGAAGCGATCGGGTCTAC

ATGGAGATTTGCATCAGACTTTCCTCTTAAAAATGCAATCGCGAAACACTACCTTGCCAA

TTGGCTTATCGCTCGAAAGACTTCAAGCAACCACCAATTGATAGTTCACCGTGAATTCCA

TGGGCCACACCTGCCGGAAAACAGCGTAGGGGCAGTCTGCAAATCTCTCAGACTTTTTAA

TATGGTCGAGATCAACGTAGTACCGACCAAAGATAAAGATTTCATAGTCGTCCACGACTT

CAACACAGAAAGAACGACGCAGTTAGGCAATTGAAATTGGTTTGATATGTCGACGAAAGA

GGCTGTTGGCACCCCCCTGGTAGTTCCCATAATGGGCGGTGACTCCCTAAAGAGCGAGTT

CACACCCACCGATGAGAAAGTTGTCAAGCAAGAAACCCTTCTCAAGAATGCCTTTGAAAC

AAACAAACAGGCAACCATACGGATTGACGCTCACGAGAGGCAGGCCGCTCCCCTCGTCGC

TTGGCTGAGCCATAGGCCGGCCTATCACGACAAGACAATCGTTATCTTTCATACATATGG

ATACAACAATGGACACGAGTTCGCCTTGGCGGTCGATCAAGCCGGCGCTACAAAAAATTG

GCGCGAAACTGTAGCGCTAATGCCAAACATTCTGCCGAATGAACTCTCTAGGCTCGCGAA

GCAATATCCACCAAATTGCACGTCGCATTTTTACGACCTCGTTGAGGCCAGCAAGAATTG

GTTCGCATCTGTCTTCTCCCAGAATATGATGGTGGTCGTCACTTTCCTCAAGATTGCGGG

CACTGGCGAAGGACAGACTAACAAAGTCGTTGATCCAAATGGAAATGAGGTTTTGCATCC

TAAAGAGAAGGAGGAATATCTCMGCGATTCAGCTTCAATCGAAATCGCGAAATGAATCTG

CGCCACCCATCCCGGAATCGAGATTGGAGGCGTCACACGAACTTATGCTTATAAGAGTCG

AGGACATCGCTACAAAATCAATCTTTGGACGGGTCAACCGAAGCGGTGGCCGACCCACGC

GACGAAGTACATCTAGGAAGATTTCGCCAAGCCTGGAAATGCGCTCAGATGGGGTCAAAC

GATTATCTCTGATCGCATTGGTGACGACCTTGCAACTTGTGTGCTTCAAAGCCGTGGAGA

AAATTTTGTCAGCAGTCATGAATATCCTGCTGTTCATGTCGATTAGATGGGCGATCTGTG

TCTTAGTCTAAGCTTCCGCTTAACACATCTTTGCTACTTTAATGTATTTTTTTAATGATA

TTGTTGGAGTTTTGAGCACATCTTAATCGCAAGTAGATATTTTTTTTGAATCGGATTCGA

CTTCAAGCAGATGTACTTCTTTGTCTCTCGATAAATCTAATGACCCTGCGAGCCGGCCTC

TCACCTTAAACTAATTTCTGAGAGTTAGGTCGGCGCCTCTCGTGGTTTCCATGTTCTATG

GAAACGGCCGCTCGGGACTTGCCTTCCCGCAACTCATCGAGCTTGCCTGGCCCGCTCGGG

TGGTGGCACAAGCCATCGTTAAATCGTTCGTAATCCTCTACTCTCGCATAAATGAGAGGG

GAATCCAAAGaTGATCACTTTTCAGCTAATTGGAATCTGCaatCCCAAAAGAGGCAAGCA

CTATGTGGGGTCCACAACTTATAGAATTTTGGCCTAACCATTCTGCATAATGGATCATGA

GATGGGCCATTTATGGCCGCAAAAACTGGCACAAATTCCAACCTATTCAGCACAATGAAC

AAGTTCTTCCAATTGGTATAAAGGAGGTTAAAATTATTCGAGTAGACATGACGTGCTGGC

ACGCCACTAGCCTCGCATTGATGCCACTCTTTCTTGCGCACACTTATATAATGCTGAGCT

AATAATAGAGATAAAAATAGTCAGCTTGTAAACTGGACCTTTATTACTGCCCCTAATCTC

AAGATCAAAGCGACATATACCTCTTCTCTTTCAAGTCTTTTTGCAACTTGTTCCAAAGCA

AGCGTTTGTGAATCTGCCAGGCAGCCTTCACGAAAATGGCTGATCAACTGTGTGAGGGGC

GTTGGTCCAAAGCCAATCTATCATTCGTTGGGATATCCATCACCCCTGAATTCATTGATG

AGGCCCTCAAAGGCTTTTGGGAGTCTTTTGCCAAAAGAGCCGTTGGCTACAAAGAGGTTT

ACGTTCAGGTTGATCGagatcctaaagttgcctgggaaggagcgctcaatggttacctcg

gcaaatattctggcgtaAAAAATGTTCATTTTGCGCCGAACAACGCCACGaTTGGAAAGC

CCCCACGCCATATCGACATTTTGCtTCGGAAAAAATTTAGCGGCGAACTTCTTGCGACgc

tcgaaggtgtggcaaTTTCAAACGGTGCAACTGGTTGGCTTGCGCTTGCATGTGTAAACC

TTCTCTTGAAGGCAAGACAAGATATTGATGTCTTCTTGTTTGGGGCAGGAAAAGTCGCCG

AGGCTGTGATTCTTGCCCTCAATCATGGTGCGGCTGCAAAAATCAAAACCATGGCAGTGC

TCAGCCAAGCTCCAATTGAAGGTGGGGTTCTAGCTCGATGCCGTCattgatcggaaggtt

ataCCAAAAGCCAAACTCGTCATAACAGCAACAAATTCCGAGGAGCTCGGTACTCGAGGC

CGACGAAATTGCGCCAAATGCAGTAACCATATCCCTCGGAAAAGATGAATTGCCCGCCGC

CTACTTCGATCGCCTTTTGAACGCAGAGGGTCTGATTATTGGCGACGATCTGGATGCGAC

CGAATCGCGCAATGTCAATTCCCTGGTGCTTTACTACTCGAAACGTGATTTGAAGCTGAC

AGAACATGGAAGGGATCATTAGATAAAGAACTACGCCAATGTCCTTGCTGATCCAGCTCT

CATGGAGGAACTTAAGACATGAGAGGGGCCAGCCAACTTTTCATCGGTTGGCCTTGCCAG

CCTAGACTTGGCGATGGCCGGCCGACTCTACGAAACTCTTACTGCGAAACTCTCCCACCC

ccagtagacaaaaccctcttaggattgcgctcttcagaaggtgcgcctCcccagtcacaa

ccggaAAGtCACTCAACTTGGCTCAAGTACCCTACTTAGTTTGTCTCTCTGTCGTGTTTA

GTATCAGCAACAATAACAAATATTGTAAATAACATATATTTATATAGTAATTATATGGCA

TGTAAGCGTGACAACGATTATGTTTTCCATAGTTTGATAAACTTAACACCCGAAAAAAGC

CGGTGACCAAAGGGTTGGGACCTCTTTGCACGAACTCCTATGGCTGCCAGACTATCTACA

GAGCTTTTTCGGAAGCTGACGCAGCATCTGATCGCCACACAATCCTCGCGGACGTGGATG

GCGTGGCTACATGCAAGCAGTTTGATTGAGAGTTTTCGAGTGACTATTTTAAAAGGACAA

TATTTCGTCCCAAGCGATAAAACTCGCTCACATGACTAAGTCATTGCTTTAATTCAACTG

CGTGAAAAGATTTAACGGGCAGAACTGTTTGTGTTTCGTATCCAGTTCACCGGCAATTAT

TGGCTACGCATGAAGCTCATTATCGTCGATTCTTTACCCAAACACTGATGCCATTCCAAA

TGAGTTGGGCCAAAGATGTACTCATGTCGGACAGGCGCGATTTGGACTTCGTGATCCTCA

AGTCTATCGCAGTGCTGCTTTATTACAAGCTATGCTTCCACCCTCCATTATTTGGTCAAC

TGGACCTAGCTTTTGACAAGAATAGGCCAGCGTTCATCTATCTATCTTTCAAAAACATGC

AGTTGCTGCTGTGTAAAGGATGTAACATCGGATTTGGTGTGGAGACGGTGCTCGCTACGA

CTATACTGCCTTACTATAGTGATCTGTCCCGCCAAGCGATGAGGGGAGCGGCCTGCCTCT

CATGAGCGTCAATCCATACGGTTGCATGTTCGTTTGTTTCAAAGGCATTCCCAAGAAGGG

TTTCCAGCTTGACAACTTTCTCATCGGTGGGTGTGAACTCGCCGGTTAGTGAGTCACCGC

CCATTTTGGGAACTACCAGGGGGGTGCCAACGACCTCCTCCGTCGACATCTCAGACCAAT

TTCAATTGCCCAACTGCACCGTTCTTTCTATGTTGAAGTCATGGGTGACTATGAAATCTC

CATCTTTGGTCGGTACTATCTCGATCTCGACCATATTGAAAAGCCTGAAAGATTTGCAAA

CTGCCCCTACACTGTTTTCCGGCAAGTGTGGCCCATGGAATCCACGGTGAACTATCAATT

GGCGGTCGCTTGAAGTCTTTCGAGCGATAAGCCAATTGGCGAGGCAGTGTTTCGCGATCG

CATCTTTAAGAGGAAAGTCCGATGCAAATCTCCATGTAGACCCAGTCGTTTCGGCCAAGG

CGGCCTCTAAATCAACTTGAGCAGCAGTTGTGTTAACGGAATCTGTTTTGACTGCCATTC

TGTAAGAAATGTCAGCAAAGGAAAAGCTAGTTAGAGAAGGAGAGCTTGCTCAATAGTGTG

TCTATTCTCAAATGAACTATACTAGCGCAATGAGTTGGTGTTGTTCGCTGATGGGAAAAA

GGAAATGCATCCCCCTCTTCTACAAAATTCTGTGGGCCCGGAGGGACTCCGTATGAAAGT

GCACAGCATGTGGACGCATCACCATGCAATtagcaagcttagccaatgtcgtgtctgtca

gAGTGTGCAGCATGTGAGCCAAGCGCAATTACAATAAAAAAATAATATAAAAACTCTGCC

GAGGTCCGAACCGTCTCAAATGCTTTGAAGGCCATCCAAGCCAAGCGCACCTAACGCCTA

GCACACCATTGTTGCGCCGCTCGAGCATTAATGGTTCGGGACCTGGTGTCATCTGGAAAC

CTATGAAGCATGGACACGGCAAAATCAGCGTCGTGGCGGkgtcagac

>sa0647_Un

TATCAATTTGATCTAGAAGCCAATCCAAACTCATTGAAAGGCCTGTCATCAACCGGTCAA

CACGGTTCAATGTTTGCTCGCGTGGCGCAGCAATGGTGCGCTAGGCGTTAGGTGCGCCTG

GCTTGGATGGCCCTCAAAGCATTTGAGGCAGTTCGGACCTCGGCAGAGATTTTATATTAT

TTTTTTGTTGTAATCGCGCTTGGCCCACATGCTGCACACTCCGATAGACACGGCATTGGC

TAAACCCGCTAATTGCGTGGTGATGCGTCCACGTGCTGCGCACTTTCATGCGGAGTCCCC

CCGAGTCCATAGAATAAGAGGGGGACGCATTTCCTTTTTCCCATCAGCAAACAACACCAA

CTCACTGTGCTAGTATAGTTCATCTGAGAACAGACACACTGCTGAGCAAGCTCTCCTTCT

CTAACTAGCTTTTTCTTTGCTGACATTTCCTACAGAATGGCAGCAAAACAGATTCCGTTA

ACACAACTACTGCTCAGGTTGATTTAGAGGCCGCCTTGGCCGAAGCGATCGGGTCTACAT

GGAGATTTGCATCAGACTTTCCTCTTAAAAATGCAATCGCGAAACACTGCCTCGCCAATT

GGCTTATCGCTCGAAAGACTTCAAGCAACCACCAATTGATAGTTCACCGTGAATTCCATG

GGCCACACCTGCCGGAAAACAGCGTAGGGGCAGTCTGCAAATCTCTCAGACTTTTTGATA

TGGTCGAGATCAACGTAGTACCGACCAAAGATAAAGATTTCATAGTCGTCCACGACTTCA

ACACAGAAAGAATGACGCAGTTAAGCAATTGAAATTGGTTTGATATGTCGACGAAAGAGG

CTGTTGGCACCCCCCTGGTAGTTCCCATAATGGGCGGTGACTCCCTAACCAGCGAGTTCA

CACCCACCGATGAGAAAGTTGTCAAGCAAGAAACCCTTCTCAAGAATGCCTTTGAAACAA

ACAAACAGGCAACCATACGGATTGATGCTCACGAGAGGCAGGCCGCTCCCCTCGTCGCTT

GGCTGAGCCATAGGCCGGCCTATCACGACAAGACAATCGTTATCTTTCATACATATGGAT

ACAACAATGGACACGAGTTCGCCTTGGCGGTCGATCAAGCCGGCGCTACAAAAAATTGGC

GCGAAACTGTAGCGCTAATGCCAAACATTCTGCCGAATGAACTCTCTAGGCTCGCGAAGC

AATATCCACCAAATTGCACGTCGCATTTTTACGACCTCGTTGAGGCCAGCAAGAATTGGT

TCGCATCTGTCTTCTCCCAGAATATGATGGTGGTCGTCACTTTCCTCAAGATTGCGGGCA

CTGGCGAAGGACAGACTAACAAAGTCGTTGATCCAAATGGAAATGAGGTTTTGCATCCTA

AAGAGAAGGAGGAATATCTCCGCGATTCAGCTTCAATCGAAATCGCGAAATGAATCTGCG

CCACCCATCCCGGAATCGAGATTGGAGGCGTCACACGAACTTATGCTTATAAGAGTCGAG

GACATCGCTACAAAATCAATCTTTGGACGGGTCAACCGAAGCGGTGGCCGACCCACGCGA

CGAAGTACATCTAGGAAGATTTCGCCAAGCCTGGAAATGCGCTCAGATGGGGTCAAACGA

TTATCTCTGATCGCATTGGTGACGACCTTGCAACTTGTGTGCTTCAAAGCCGTGGAGAAA

ATTTTGTCAGCAGTCATGAATATCCTGCTGTTCATGTCGATTAGATGGGCGATCTGTGTC

TTAGTCTAAGCTTCCGCTTAACACATCTTTGCTACTTTAATGTATTTTTTTAATGATATT

GTTGGAGTTTTGAGCACATCTTAATCGCAAGTAGATATTTTTTTTGAATCGGATTCGACT

TCAAGCAGATGTACTTCTTTGTCTCTCGATAAATCTAATGACCCTGCGAGCCGGCCTCTC

ACCTTAAACTAATTTCTGAGAGTTAGGTCGGCGCCTCTCGTGGTTTCCATGTTCTATGGA

AACGGCCGCTCGGGACTTGCCTTCCCGCAACTCATCGAGCTTGCCTGGCCCGCTCGGGTG

GTGGCACAAGCCATCGTTAAATCGTTCGTAATCCTCTACTCTCGCATAAATGAGAGGGGA

ATCCAAAGATGATCACTTTTCAGCTAATTGGAATCTGCAATCCCAAAAGAGGCAAGCACT

ATGTGGGGTCCACAACTTATAGAATTTTGGCCTAACCATTCTGCATAATGGATCATGAGA

TGGGCCATTTATGGCCGCAAAAACTGGCACAAATTCCAACCTATTCAGCACAATGAACAA

GTTCTTCCAATTGGTATAAAGGAGGTTAAAATTATTCGAGTAGACATGACGTGCTGGCAC

GCCACTAGCCTCGCATTGATGCCACTCTTTCTTGCGCACACTTATATAATGCTGAGCTAA

TAATAGAGATAAAAATAGTCAGCTTGTAAACTGGACCTTTATTACTGCCCCTAATCTCAA

GATCAAAGCGACATATACCTCTTCTCTTTCAAGTCTTTTTGCAACTTGTTCCAAAGCAAG

CGTTTGTGAATCTGCCAGGCAGCCTTCACGAAAATGGCTGATCAACTGTGTGAGGGGCGT

TGGTCCAAAGCCAATCTATCATTCGTTGGGATATCCATCACCCCTGAATTCATTGATGAG

GCCCTCAAAGGCTTTTGGGAGTCTTTTGCCAAAAGAGCCGTTGGCTACAAAGAGGTTTAC

GTTCAGGTTGATCGAGATCCTAAAGTTGCCTGGGAAGGAGCGCTCAATGGTTACCTCGGC

AAATATTCTGGTGTAAAAAATGTTCATTTTGCGCCGAACAACGCCACGATTGGAAAGCCC

CCACGCCATATCGACATTTTGCTTCGGAAAAAATTTAGCGGCGAACTTCTTGCGACGCTC

GAAGGTGTGGCAATTTCAAACGGTGCAACTGGTTGGCTTGCGCTCGCATGTGTAAACCTT

CTCTTGAAGGCAAGACAAGATATTGATGTCTTCTTGTTTGGGGCAGGAAAAGTCGCCGAG

GCTGTGATTCTTGCCCTCAATCATGGTGCGGCTGCAAAAATCAAAACCATGGCAGTGCTC

AGCCAAGCTCCAATTGAAGGTGGGGTTCTAGCTCGATGCCGTCATTGATTGGAAGGTTAT

ACCAAAAGCCAAACTCGTCATAACAGCAACAAATTCCGAGGAGCTCGGTACTCGAGGCCG

ACGAAATTGCGCCAAATGCAGTAACCATATCCCTCGGAAAAGATGAATTGCCCGCCGCCT

ACTTCGATCGCCTTTTGAACGCAGAGGGTCTGATTATTGGCGACGATCTGGATGCGACCG

AATCGCGCAATGTCAATTCCCTGGTGCTTTACTACTCGAAACGTGATTTGAAGCTGACAG

AACATGGAAGGGATCATTAGATAAAGAACTACGCCAATGTCCTTGCTGATCCAGCTCTCA

TGGAGGAACTTAAGACATGAGAGGGGCCAGCCAACTTTTCATCGGTTGGCCTTGCCAGCC

TAGACTTGGCGATGGCCGGCCGACTCTACGAAACTCTTACTGCGAAACTCTCCCACCCCC

AGTAGACAAAACCCTCTTAGGATTGCGCTCTTCAGAAGGTGCGCCTCCCCAGTCACAACC

GGAAAGTCACTCAACTTGGCTCAAGTACCCTACTTAGTTTGTCTCTCTGTCGTGTTTAGT

ATCAGCAACAATAACAAATATTGTAAATAACATATATTTATATAGTAATTATATGGCATG

TAAGCGTGACAACGATTATGTTTTCCATAGTTTGATAAACTTAACACCCGAAAAAAGCCG

GTGACCAAAGGGTTGGGACCTCTTTGCACGAACTCCTATGGCTGCCAGACTATCTACAGA

GCTTTTTCGGAAGCTGACGCAGCATCTGATCGCCACACAATCCTCGCGGACGTGGATGGC

GTGGCTACATGCAAGCAGTTTGATTGAGAGTTTTCGAGTGACTATTTTAAAAGGACAATA

TTTCGTCCCAAGCGATAAAACTCGCTCACATGACTAAGTCATTGCTTTAATTCAACTGCG

TGAAAAGATTTAACGGGCAGAACTGTTTGTGTTTCGTATCCAGTTCACCGGCAATTATTG

GCTACGCATGAAGCTCATTATCGTCGATTCTTTACCCAAACACTGATGCCATTCCAAATG

AGTTGGGCCAAAGATGTACTCATGTCGGACAGGCGCGATTTGGACTTCGTGATCCTCAAG

TCTATCGCAGTGCTGCTTTATTACAAGCTATGCTTCCACCCTMYATTATTTGGTCAACTG

GACCTAGCTTTTGACAAGAATAGGCCAGCGTTCATCTATCTATCTTTCAAAAACATGCAG

TTGCTGCTGTGTAAAGGATGTAACATCGGATTTGGTGTGGAGACGGTGCTCGCTACGACT

ATACTGCCTTACTGTAGTGATCTGTCCCGCCAAGCGATGAGGGGAGCGGCCTGCCTCTCA

TGAGCGTCAATCCATACGGTTGCATGTTCGTTTGTTTCAAAGGCATTCCCAAGMAGGGTT

TCCAGCTTGACAACTTTCTCATCGGTGGGTGTGAACTCGCCGGTTAGTGAGTCACCGCCC

ATTTTGGGAACTACCAGGGGGGTGCCAACGACCTCCTCCGTCGACATCTCAGACCAATTT

CAATTGCCCAACTGCACCGTTCTTTCTATGTTGAAGTCATGGGTGACTATGAAATCTCCA

TCTTTGGTCGGTACTATCTCGATCTCGACCATATTGAAAAGCCTGAAAGATTTGCAAACT

GCCCCTACACTGTTTTCCGGCAAGTGTGGCCCATGGAATCCACGGTGAACTATCAATTGG

CGGTCGCTTGAAGTCTTTCGAGCGATAAGCCAATTGGCGAGGCAGTGTTTCGCGATCGCA

TCTTTAAGAGGAAAGTCCGATGCAAATCTCCATGTAGACCVAGTCGTTTCGGCCAAGGCG

GCCTCTAAATCAACTTGAGCAGCAGTTGTGTTAACGGAATCTGTTTTGACTGCCATTCTG

TAAGAAATGTCAGCAAAGGAAAAGCTAGTTAGAGAAGGAGAGCTTGCTCAATAGTGTGTC

TATTCTCAAATGAACTATACTAGCGCAATGAGTTGGTGTTGTTCGCTGATGGGAAAAAGG

AAATGCATCCCCCTCTTCTACAAAATTCTGTGGGCCCGGAGGGACTCCGTATGAAAGTGC

ACAGCATGTGGACGCATCACCATGCAATTAGCAAGCTTAGCCAATGTCGTGTCTGTCAGA

GTGTGCAGCATGTGAGCCAAGCGCAATTACAATAAAAAAATAATATAAAAACTCTGCCGA

GGTCCGAACCGTCTCAAATGCTTTGAAGGCCATCCAAGCCAAGCGCACCTAACGCCTAGC

ACACCATTGTTGCGCCGCTCGAGCATTAATGGTTCGGGACCTGGTGTCATCTGGAAACCT

ATGAAGCATGGACACGGCAAAATCAGCGCCGTGGCGGTGTCAGACACGAGGACACGTCGG

>sa0623_Un

CAAATTTCCCTCACCTTGAAGTATCAATTTGATCTAGAAGCCAATCCAAACTCATTGAAA

GGCCTGTCATCAACCGGTCAACACGGTTCAATGTTTGCTCGCGTGGCACAGCAATGGTGT

GCTAGGCGTTAGGTGCGCCTGGCTTGGATGGCCCTCAAAGCATTTGAGGCAGTTCGGACC

TCGGCAGAGATTTTATATTATTTTTTTGTTGTAATCACGCTTGGCCCACATGCTGCACAC

TCCGATAGACACGGCATTGGCTAAACCCGCTAATTGCGTGGTGATGCGTCCACGTGCTGC

GCACTTTCAGGCGGAGTCCCCCCGATTCCATAGAATAAGAGGGGGACGCGTTTCCTTTTT

CCCATCAGCAAACAACACCAACTCACTGTGCTAGTATAGTTCATCTGAGAACAGACACAC

TGCTGAGCAAGCTCTCCTTCTCCAACTAGCTTTTTCTTTGCTGACATTTCCTACAGAATG

GCAGCAAAACAGATTCCGTTAACACAACTACTGCTCAGGTTGATTTAGAGGCCGCCTTGG

CCGAAGCGATCGGGTCTACATGGAGATTTGCATMAGACTTTCCTCTTAAAAATGCAATCG

CGAAACACTGCCTCGCCAATTGGCTTATCGCTCGAAAGACTTCAAGCAACCACCAATTGA

TAGTTCACCGTGAATTCCATGGGCCACACCTGCCGGAAAACAGCGTAGGGGCAGTCTGCA

AATCTCTCAGACTTTTTAATATGGTCGAGATCAACGTAGTACCGACCAAAGATAAAGATT

TCATAGTCGTCCACGACTTCAACACAGAAAGAACGACGCAATTAGGCAATTGAAATTGGT

TTGATATGTCGACGAAAGAGGCTGTTGGCACCCCCCTGGTAGTTCCCATAATGGGCGGTG

ACTCCCTAACCAGCGAGTTCACACCCACCGATGAGAAAGTTGTCAAGCAAGAAACCCTTC

TCAAGAATGCCTTTGAAACAAACAAACAGGCAACCATACGGATTGACGCTCACGAGAGGC

AGGCCGMTCCCCTCGTCGCTTGGCTGAGCCATAGGCCGGCCTATCACGACAAGACAATCG

TTATCTTTCATACATATGGATACAACAATGGACACGAGTTCGCCTTGGCGGTCAATCAAG

CCGGCGCTACAAAAAATTGGCGCGAAACTGTAGCGCTAATGCCAAACATTCTGCCGAATG

AACTCTCTAGGCTCGCGAAGCAATATCCACCAAATTGCACGTCGCATTTTTACGACCTCG

TTGAGGCCAGCAAGAATTGGTTCGCATCTGTCTTCTCCCAGAATATGATGGTGGTCGTCA

CTTTCCTCAAGATTGCGGGCACTGGCGAAGGACAGACTAACAAAGTCGTTGATCCAAATG

GAAATGAGGTTCTGCATCCTAAAGAGAAGGAGGAATATCTCCGCGATTCAGCTTCAATCG

AAATCGCGAAATGAATCTGCGCCACCCATCCCGGAATCGAGATTGGAGGCGTCACACGAA

CTTATGCTTATAAGAGTCGAGGACATCGCTACAAAATCAATCTTTGGACGGGTCAACCGA

AGCKGTGGCCGACCCACGCGACGAAGTACATCTGGGAAGATTTCGCCAAGCCTGGAAATG

CGCTCAGATGGGGTCAAACGATTATCTCTGATCGCATTGGTGACGACCTTGCAGCTTGTG

TGCTTCAAAGCTGTGGAGAAAATTTTGTCAGCAGTCATGAATATCCTGCTGTTCATGTCG

ATTAGATGGGCGATCTGTGTCTTAGTCTAAGCTTCCGCTTAACGCATCTTTGCTACTTTA

ATGTATTTTTTTAATGATATTGTTGGAGTTTTGAGCACATCTTAATCGCAAGTAGATATT

TTTTTTGAATCGGATTCGACTTCAAGCAGATGTACTTCTTTGTCTCTCGATAAATCTAAT

GACCCTGCGACCGGCCTCTCGCCTTAAACTAATTTCTGAGAGTTAGGTCGGCGCCTCTCG

TGGTTTCCATGTTCTATGGAAACGGCCGCTCGRGACTTGCCTTCCCGCAACTCATCGAGC

TTGCCTGGCCCGCTCGGGTGGTGGCACAAGCCATCGTTAAATCGTTCGTAATCCTCTACT

CTCGCATAAATGAGAGGGGAATCCAAAGATGATCACTTTTCAGCTAATTGGAATCTGCAA

TCCCAAAAGAGGCAAGCACTATGTGGGGTCCACAACTTATAGAATTTTGGCCTAACCATT

CTGCATAATGGATCATGACATGGGCCATTTATGGCCGTAAAAACTGGCACAAATTCCAAC

CTATTCAGCACAATGAACAAGTTCTTCCAATTGGAATAAAGGAGGTTAAAATTATTCGAG

TAGACATGACGTGCTGGCATGCCACTAGCCTCGCATTGATGCCACTCTTTCTTGCGCACA

CTTATATAATGCTGAGCTAATAATAGAGATAAAAATAGTCAGCTTGTAAACTGGACCTTT

ATTACTGCCCCTAATCTCAAGATCAAAGCGACATATACCTCTTCTCTTTCAAGTCTTTTT

GCAACTTGTTCCAAAGCAAGCGTTTGTGAATCTGCCAGGCAGCCTTCACGAAAATGGCTG

ATCAACTGTGTGAGGGGCGTTGCTCCGAAGCCAATCTATCATTCGTTGGGATATCCATCA

CCCCTGAATTCATCGATGAGGCCCTCAGAGGCTTTTGGGAGTCTTTTGCCAAAARAGCCG

TTGGCTACAAAGAGGTTTACGTTCAGGTTGATCGAGATCCTAAAGTTGCCTGGGAAGGAG

CGCTCAATGGTTACCTCGGCAAATATTCTGGCGTAAAAAATGTTCATTTTGCGCCGAACA

ACGCCACGATTGGAAAGCCCCCACGCCATATCGACATTTTGCTTCGGAAAAAATTTAGCG

GCGAACGTCTTGCGACGCTCGAAGGTGTGGCAATTTCAAACGGTGCAACTGGTTGGCTTG

CGCTYGCATGTGTAAACCTTCTCTTGAAGGCAAGACAAGATATTGATGTCTTCTTGTTTG

GGGCAGGAAAAGTTGCCGAGGCTGTGATTCTTGCCCTCAATCATGGTGCGGCTGCAAAAA

TCAAAACCATGGCAGTGCTCAGCCAAGCTCCAATTGAAGGTGGGGTTCCAGCTTGATGCC

GTCATTGATCGGAAGGTTATACCAAAAGCCAAACTCGTCATAACAGCAACAAATTCCGAG

GAGCTCGGTACTCGAGGCCGACGAAATTGCGCCAAATGCAGTAACCATATCCCTCGGAAA

AGATGAATTGCCCGCCGCCTACTTCGATCGCCTTTTGAACGCAGAGGGTCTGATTATTGG

CGACGATCTGGATGCGATCGAATCGCACAATGTCAATTCCCTGGTGCTTTACTACTCGAA

ACGTGATTTGAAGCTGACAGAACATGGAAGGGATCATTGGATAAAGAACTACGCCAATGT

CCTTGCTGATCCAGCTCTCATGGAGGAACTTAAGACATGAGAGGGGCCAGCCAACTTTTC

ATCGGTTGGCCTTGCCAGCCTAGACTTGGCGATGGCCGGCCGACTCTACGAAACTCTTAC

TGCGAAACTCTCCCACCCCCAGTAGACAAAACCCTCTTAGGATTGCGCTCTTCAGAAGGT

GCGCCTCCCCAGTCACAACCGGAAAGTCACTCAACTTGGCTCAAGTACCCTACTTAGTTT

GTCTCTCTGTCGTGTTTAGTATCAGCAACAATAACAAATATTGTAAATAACATATATTTA

TATGGTAATTATATGGCATGTAAGCGTGACAACGATTATGTTTTCCATAGTTTGATAAAC

TCAACACCCGAAAAAGCCGGTGACCAAAGGGTTGGGACCTCTTTGCACGAACTCCTATGG

CTGCCAGACTATCTACAGAGCTTTTTCGGAAGCTGACGCAGCATCTGATCGCCACACAAT

CCTCGCGGACGTGGATGGCGTGGCTACATGCAAGCAGTTTGACTGAGAGTTTTCGAGTGA

CTGTTTTAAAAGGACAATATTTCGTCCCAAGCGATAAAACTCGCTCACATGACTAAGTCG

TTGCTTTAATTCAACTGCGTGAAAAGATTTAAAGGGCAGAACTGTTTGTGTTTCGTATCC

AGTTCACCGGCAATTATTGGCTACGCATAAAGCTCATTATCGTCGATTCTTTACCCAAAC

ACTGATGCCATTCCAAATGCGTTGGGCCAAAGATGTACTCATGTCGGACAGGCGCAATTT

GGACTTCGTGATCCTCAAGTCTATCGCAGTGCTGCTTTATTACAGGCTATGCTTCCACCC

TCCATTATTTGGTCAACTGGACCTAGCTTTTGACAAGAATAGGCCAGCGTTCATCTATCT

ATCTTTCAAAAACATGCAGTTGCTGCTGCGTAAAGGATGTAACATCGGATTTGGTGTGGA

GACGGTGCTCGCTACGACTATACTGCCTTACTGTAGTGATCTGTCCYGCCAAGCGATGAG

GGAAGCGGCCTGCCTCTCGTGAGCGTCAATCCATACGGTTGCATGTTCGTTTGTTTCAAA

AGCATTCCCAAGAAGGGTTTCCAGCTTGACAACTTTCTCATCGGTGGGTGTGAACTCGCC

GGTTAGTGAGTCACCGCCCATTTTGGGAACTACCAGGGGGGTGCCAACGACCTCCTCCGT

CGACATCTCAGACCAATTTCAATTGCCCAACTGCACCGTTCTTTCTATGTTGAAGTCATG

GGCGACTATGAAATCTCCATCTTTGGTCGGTACTACCTCGATCTCGACCATATTGAAAAG

CCTRAAAGATTTGCAAACTGCCCCTACACTGTTTTCCGGCAAGTGTGGCCCATGGAATCC

ACGGTGAACTATCAATTGGCGGTCGCTTGAAGTCTTTCGAGCGATAAGCCAATTGGCGAG

GCAGTGTTTCGCGATCGCATCTTTAAGAGGAAAGTCCGATGCAAATCTCCATGTAGACCC

AGTCGTTTCGGCCAAGGCGGCCTCTAAATCAACTTGAGCAGCAGTTGTGTTAACGGAATC

TGTTTTGACTGCCATTCTGTAAGAAATGTCAGCAAAGGAAAAGCTAGTTAGAGAAGGAGA

GCTTGCTCAATAGTGTGTCTATTCTCAAATGAACTATACTAGCGCAATGAGTTGGTGTTG

TTCGCTGATGGGAAAAAGGAAACGCATCCCCCTCTTCTACAAAATTCTGTGGGCCCGGAG

GGACTCCGTATGAAAGTGCACAGCATGTGGACGCATCACCATGCAATTAGCAAGCTTAGC

CAATGTCGTGTCTGTCAGAGTGTGCAGCATGTGAGCCAAGCGCAATTACAATAAAAAAAT

AATATAAAAACTCTGCCGAGGTCCGAACCGTCTCAAATGCTTTGAGGGCCATCCAAGCCA

AGCGCACCTAACGCCTAGCACACCATTGTTGCGCCGCTCGAGCATTAATGGTTCGGGACC

TGGTGTCATCTGGAAACCTATGAAGCATAGACACGGCAAAATCAGCGCCGTGGMGGTGTC

AGACACGAGGACACGTCGGGGACAC

>sa2467_Un

CTCCAAATTTCCCTCACCTTGAAGTATCAATTTGATCTAGAAGCCAATCCAAACTCATTG

AAAGGCCTGTCATCAACCGGTCAACACGGTTCAATGTTTGCTCGCGTGGCGCAGCAATGG

TGCGCTAGGCGTTAGGTGCGCCTGGCTTGGATGGCCCTCAAAGCATTTGAGGCAGTTCGG

ACCTCGGCAGAGATTTTATATTATTTTTTTGTTGTAATCGCGCTTGGCCCACATGCTGCA

CACTCCGATAGACACGGCATTGGCTAAACCCGCTAATTGCGTGGTGATGCGTCCACGTGC

TGCGCACTTTCATGCGGAGTCCCCCCGAGTCCATAGAATAAGAGGGGGACGCATTTCCTT

TTTCCCATCAGCAAACAACACCAACTCACTGTGCTAGTATAGTTCATCTGAGAACAGACA

CACTGCTGAGCAAGCTCTCCTTCTCTAACTAGCTTTTTCTTTGCTGACATTTCCTACAGA

ATGGCAGCAAAACAGATTCCGTTAACACAACTACTGCTCAGGTTGATTTAGAGGCCGCCT

TGGCCGAAGCGATCGGGTCTACATGGAGATTTGCATCARACTTTCCTCTTAAAAATGCAA

TCGCGAAACACTGCCTCGCCAATTGGCTTATCGCTCGAAAGACTTCAAGCAACCACCAAT

TGATAGTTCACCGTGAATTCCATGGGCCACACCTGCCGGAAAACAGCGTAGGGGCAGTCT

GCAAATCTCTCAGACTTTTTAATATGGTCGAGATCAACGTAGTACCGACCAAAGATAAAG

ATTTCATAGTCRTCCACGACTTCAACACAGAAAGAATGACGCAGTTAGGCAATTGAAATT

GGTTTGATATGTCGACGAAAGAGGCTGTTGGCACCCCCCTGGTAGTTCCCATAATGGGCG

GTGACTCCCTAACCAGCGAGTTCACACCCACCGATGAGAAAGTTGTCAAGCAAGAAACCC

TTCTCAAGAATGCCTTTGAAACAAACAAACAGGCAACCATACGRATTGATGCTCACGAGA

GGCAGGCCGCTCCCCTCGTCGCTTGGCTGAGCCATAGGCCGGCCTATCACGACAAGACAA

TCGTTATCTTTCATACATATGGATACAACAATGGACACGAGTTCGCCTTGGCGGTCGATC

AAGCCGGCGCTACAAAAAATTGGCGCGAAACTGTAGCGCTAATGCCAAACATTCTGCCGA

ATGAACTCTCTAGGCTCGCGAAGCAATATCCACCAAATTGCACGTCGCATTTTTACGACC

TCGTTGAGGCCAGCAAGAATTGGTTCGCATCTGTCTTCTCCCAGAATATGATGGTGGTCG

TCACTTTCCTCAAGATTGCGGGCACTGGCGAAGGACAGACTAACAAAGTCGTTGATCCAA

ATGGAAATGAGGTTTTGCATCCTAAAGAGAAGGAGGAATATCTCCGCGATTCAGCTTCAA

TCGAAATCGCGAAATGAATCTGCGCCACCCATCCCGGAATCGAGATTGGAGGCGTCACAC

GAACTTATGCTTATAAGAGTCGAGGACATCGCTACAAAATCAATCTTTGGACGGGTCAAC

CGAAGCGGTGGCCGACCCACGCGACGAAGTACATCTAGGAAGATTTCGCCAAGCCTGGAA

ATGCGCTCAGATGGGGTCAAACGATTATCTCTGATCGCATTGGTGACGACCTTGCAACTT

GTGTGCTTCAAAGCCGTGGAGAAAATTTTGTCAGCAGTCATGAATATCCTGCTGTTCATG

TCGATTAGATGGGCGATCTGTGTCTTAGTCTAAGCTTCCGCTTAACACATCTTTGCTACT

TTAATGTATTTTTTTAATGATATTGTTGGAGTTTTGAGCACATCTTAATCGCAAGTAGAT

ATTTTTTTTGAATCGGATTCGACTTCAAGCAGATGTACTTCTTTGTCTCTCGATAAATCT

AATGACCCTGCGAGCCGGCCTCTCACCTTAAACTAATTTCTGAGAGTTAGGTCGGCGCCT

CTCGTGGTTTCCATGTTCTATGGAAACGGCCGCTCGGGACTTGCCTTCCCGCAACTCATC

GAGCTTGCCTGGCCCGCTCGGGTGGTGGCACAAGCCATCGTTAAATCGTTCGTAATCCTC

TACTCTCGCATAAATGAGAGGGGAATCCAAAGATGATCACTTTTCAGCTAATTGGAATCT

GCAATCCCAAAAGAGGCAAGCACTATGTGGGGTCCACAACTTATAGAATTTTGGCCTAAC

CATTCTGCATAATGGATCATGAGATGGGCCATTTATGGCCGCAAAAACTGGCACAAATTC

CAACCTATTCAGCACAATGAACAAGTTCTTCCAATTGGTATAAAGGAGGTTAAAATTATT

CGAGTAGACATGACGTGCTGGCACGCCACTAGCCTCGCATTGATGCCACTCTTTCTTGCG

CACACTTATATAATGCTGAGCTAATAATAGAGATAAAAATAGTCAGCTTGTAAACTGGAC

CTTTATTACTGCCCCTAATCTCAAGATCAAAGCGACATATACCTCTTCTCTTTCAAGTCT

TTTTGCAACTTGTTCCAAAGCAAGCGTTTGTGAATCTGCCAGGCAGCCTTCACGAAAATG

GCTGATCAACTGTGTGAGGGGCGTTGGTCCAAAGCCAATCTATCATTCGTTGGGATATCC

ATCACCCCTGAATTCATTGATGAGGCCCTCAAAGGCTTTTGGGAGTCTTTTGCCAAAAGA

GCCGTTGGCTACAAAGAGGTTTACGTTCAGGTTGATCGAGATCCTAAAGTTGCCTGGGAA

GGAGCGCTCAATGGTTACCTCGGCAAATATTCTGGTGTAAAAAATGTTCATTTTGCGCCG

AACAACGCCACGATTGGAAAGCCCCCACGCCATATCGACATTTTGCTTCGGAAAAAATTT

AGCGGCGAACTTCTTGCGACGCTCGAAGGTGTGGCAATTTCAAACGGTGCAACTGGTTGG

CTTGCGCTCGCATGTTGTAAACCTTCTCTTGAAGGCAAGACAAGATATTGATGTCTTCTT

GTTTGGGGCAGGAAAAGTCGCCGAGGCTGTGATTCTTGCCCTCAATCATGGTGCGGCTGC

AAAAATCAAAACCATGGCAGTGCTCAGCCAAGCTCCAATTGAAGGTGGGGTTCTAGCTCG

ATGCCGTCATTGATTGGAAGGTTATACCAAAAGCCAAACTCGTCATAACAGCAACAAATT

CCGAGGAGCTCGGTACTCGAGGCCGACGAAATTGCGCCAAATGCAGTAACCATATCCCTC

GGAAAAGATGAATTGCCCGCCGCCTACTTCGATCGCCTTTTGAACGCAGAGGGTCTGATT

ATTGGCGACGATCTGGATGCGACCGAATCGCGCAATGTCAATTCCCTGGTGCTTTACTAC

TCGAAACGTGATTTGAAGCTGACAGAACATGGAAGGGATCATTAGATAAAGAACTACGCC

AATGTCCTTGCTGATCCAGCTCTCATGGAGGAACTTAAGACATGAGAGGGGCCAGCCAAC

TTTTCATCGGTTGGCCTTGCCAGCCTAGACTTGGCGATGGCCGGCCGACTCTACGAAACT

CTTACTGCGAAACTCTCCCACCCCCAGTAGACAAAACCCTCTTAGGATTGCGCTCTTCAG

AAGGTGCGCCTCCCCAGTCACAACCGGAAAGTCACTCAACTTGGCTCAAGTACCCTACTT

AGTTTGTCTCTCTGTCGTGTTTAGTATCAGCAACAATAACAAATATTGTAAATAACATAT

ATTTATATAGTAATTATATGGCATGTAAGCGTGACAACGATTATGTTTTCCATAGTTTGA

TAAACTTAACACCCGAAAAAAGCCGGTGACCAAAGGGTTGGGACCTCTTTGCACGAACTC

CTATGGCTGCCAGACTATCTACAGAGCTTTTTCGGAAGCTGACGCAGCATCTGATCGCCA

CACAATCCTCGCGGACGTGGATGGCGTGGCTACATGCAAGCAGTTTGATTGAGAGTTTTC

GAGTGACTATTTTAAAAGGACAATATTTCGTCCCAAGCGATAAAACTCGCTCACATGACT

AAGTCATTGCTTTAATTCAACTGCGTGAAAAGATTTAACGGGCAGAACTGTTTGTGTTTC

GTATCCAGTTCACCGGCAATTATTGGCTACGCATGAAGCTCATTATCGTCGATTCTTTAC

CCAAACACTGATGCCATTCCAAATGAGTTGGGCCAAAGATGTACTCATGTCGGACAGGCG

CGATTTGGACTTCGTGATCCTCAAGTCTATCGCAGTGCTGCTTTATTACARGCTATGCTT

CCACCCTCCATTATTTGGTCAACTGGACCTAGCTTTTGACAAGAATAGGCCAGCGTTCAT

CTATCTATCTTTCAAAAACATGCAGTTGCTGCTGTGTAAAGGATGTAACATCGGATTTGG

TGTGGAGACGGTGCTCGCTACGACTATACTGCCTTACTGTAGTGATCTGTCCCGCCAAGC

GATGAGGGGAGCGGCCTGCCTCTCATGAGCGTCAATCCATACGGTTGCATGTTCGTTTGT

TTCAAAGGCATTCCCAAGAAGGGTTTCCAGCTTGACAACTTTCTCATCGGTGGGTGTGAA

CTCGCCGRTTAGTGAGTCACCGCCCATTTTGGGAACTACCAGGGGGGTGCCAACGACCTC

CTCCGTCGACATCTCAGACCAATTTCAATTGCCCAACTGCACCGTTCTTTCTATGTTGAA

GTCATGGGTGACTATGAAATCTCCATCTTTGGTCGGTACTATCTCGATCTCGACCATATT

GAAAAGCCTGAAAGATTTGCAAACTGCCCCTACACTGTTTTCCGGCAAGTGTGGCCCATG

GAATCCACGGTGAACTATCAATTGGCGGTCGCTTGAAGTCTTTCGAGCGATAAGCCAATT

GGCGAGGCAGTGTTTCGCGATCGCATCTTTAAGAGGAAAGTCCRATGCAAATCTCCATGT

AGACCCAGTCGTTTCGGCCAAGGCGGCCTCTAAATCAACTTGAGCAGCAGTTGTGTTAAC

GGAATCTGTTTTGACTGCCATTCTGTAAGAAATGTCAGCAAAGGAAAAGCTAGTTAGAGA

AGGAGAGCTTGCTCAATAGTGTGTCTATTCTCAAATGAACTATACTAGCGCAATGAGTTG

GTGTTGTTCGCTGATGGGAAAAAGGAAATGCATCCCCCTCTTCTACAAAATTCTGTGGGC

CCGGAGGGACTCCGTATGAAAGTGCACAGCATGTGGACGCATCACCATGCAATTAGCAAG

CTTAGCCAATGTCGTGTCTGTCAGAGTGTGCAGCATGTGAGCCAAGCGCAATTACAATAA

AAAAATAATATAAAAACTCTGCCGAGGTCCGAACCGTCTCAAATGCTTTGAAGGCCATCC

AAGCCAAGCGCACCTAACGCCTAGCACACCATTGTTGCGCCGCTCGAGCATTAATGGTTC

GGGACCTGGTGTCATCTGGAAACCTATGAAGCATGGACACGGCAAAATCAGCGCCGTGGC

GGTGTCAGACACGAGGACACGTCGGGGACACGTACAGG

>sa2465_Un

CCTCTCCAAATTTCCCTCACCTTGAAGTATTAATTTGATCTAGAAGCCAATCCAAACTCA

TTGAAAGGCCTGTCATCAACCGGTCAACACGGTTCAATGTTTGCTCGCGTGGCGCAGCAA

TGGTGTGCTAGGCGTTAGGTGCGCCTGGCTTGGATGGCCCTCAAAGCATTTGAGGCAGTT

CGGACCTCGGCAGAGATTTTATATTATTTTTTTGTTGTAATCGCGCTTGGCCCACATGCT

GCACACTCCGATAGACACGGCATTGGCTAAACCCGCTAATTGCGTGGTGATGCGTCCACG

TGCTGTGCACTTTCATGCGGAGTCCCCCCGAGTCCATAGAATAAGAGGGGGACGCGTTTC

CTTTTTCCCATCAGCAAACAACACCAACTCACTGTGCTAGTATAGTTCATCTGAGAACAG

ACACACTGCTGAGCAAGCTCTCCTTCTCCAACTAGCTTTTTCTTTGCTGACATTTCCTAC

AGAATGGCAGCAAAACAGATTCCGTTAACACAACTACTGCTCAGGTTGATTTAGAGGCCG

CCTTGGCCGAAGCGATCGGGTCTACATGGAGATTTGCATCAGACTTTCCTCTTAAAAATG

CAATCGCGAAACACTGCCTTGCCAATTGGCTTATCGCTCGAAAGACTTCAAGCAACCACC

AATTGATAGTTCACCGTGAATTCCATGGGCCACACCTGCCGGAAAACAGCGTAGGGGCAG

TCTGCAAATCTCTCAGACTTTTTAATATGGTCGAGATCAACGTAGTACCGACCAAAGATA

AAGATTTCATAGTCGTCCACGACTTCAACACAGAAAGAACGACGCAGTTAGGCAATTGAA

ATTGGTTTGATATGTCGACGAAAGAGGCTGTTGGCACCCCCCTGGTAGTTCCCATAATGG

GCGGTGACTCCCTAACCAGCGAGTTCACACCCACCAATGAGAAAGTTGTCAAGCAAGAAA

CCCTTCTCAAGAATGCCTTTGAAACAAACAAACAGGCAACCATACGGATTGATGCTCACG

AGAGGCAGGCCGCTCCCCTCGTCGCTTGGCTGAGCCATAGGCCGGCCTATCACGACAAGA

CAATCGTTATCTTTCATACATATGGATACAACAATGGACACGAGTTCGCCTTGGCGGTCG

ATCAAGCCGGCGCTACAAAAAATTGGCGCGAAACTGTAGCGCTAATGCCAAACATTCTGC

CGAATGAACTCTCTAGGCTCGCGAAGCAATATCCACCAAATTGCACGTCGCATTTTTACG

ACCTCGTTGAGGCCAGCAAGAATTGGTTCGCATCTGTCTTCTCCCAGAATATGATGGTGG

TCGTCACTTTCCTCAAGATTACGGGCACTGGCGAAGGACAGACTAACAAAGTCGTTGATC

CAAATGGAAATGAGGTTCTGCATCCTAAAGAGAAGGAGGAATATCTCCGCGATTCAGCTT

CAATCGAAATCGCGAAATGAATCTGCGCCACCCATCCCGGAATCGAGATTGGAGGCGTCA

CACGAACTTATGCTTATAAGAGTCGAGGACATAGCTACAAAATCAATCTTTGGACGAGTC

AACCGAAGCGGTGGCCGACCCACGCGACGAAGTACATCTAGGAAGATTTCGCCAAGCCTG

GAAATGCGCTCAGATGGGGTCAAAYGATTATCTCTGATCGCATTGGTGACGACCTTGCAG

CTTGTGTGCTTCAAAGCCGTGGAGAAAATTTTGTCAACAGTCATGAATATCCTGCTGTTC

ATGTCGATTAGATGGGCGATCTGTGTCTTAGTCTAAGCTTCCGCTTAACGCATCTTTGCT

ACTTTAATGTATTTTTTTAATGATATTGTTGGAGTTTTGAGCACATCTTAATCGCAAGTA

GATATTTTTTTTGAATCGGATTCGACTTCAAGCAGATGTACTTCTTTGTCTCTYGATAAA

TCTAATGACCCTGTGAGCCGGCCTCTCACCTTAAACTAATTTCTGAGAGTTAGGTCGGCG

CCTCTCGTGGTTTCCATGTTCTATGGAAACGGCCGCTCGGGACTTGCCTTCCCGCAACTC

ATCGAGCTTGCCTGGCCCGCTCGGGTGGTGGCACAAGCCATCGTTAAATCGTTCGTAATC

CTCTACTCTCGCATAAATGAGAGGGGAATCCAAAGATGATCACTTTTCAGCTAATTGGAA

TCTGCAATCCCAAAAGAGGCAAGCAGTATGTGGGGTCCACAACTTATAGAATTTTGGCCT

AACCATTCTGCATAATGGATCATGACATGGGCCATTTATGGCCGCAAAAACTGGCACAAA

TTCCAACCTATTCAGCACAATGAACAAGTTCTTCCAATTGGAATAAAGGAGGTTAAAATT

ATTCGAGTAGACATGACGTGCTGGCACGCCACTAGCCTCGCATTGATGCCACTCTTTCTT

GCGCACACTTATATAATGCTGAGCTAATAATAGAGATAAAAATAGTCAGCTTGTAAACTG

GACCTTTATTACTGCCCCTAATCTCAAGATCAAAGCGACATATACCTCTTCTCTTTCAAG

TCTTTTTGCAACTTGTTCCAAAGCAGGCGTTTGTGAATCTGCCAGGCAGCCTTCACGAAA

ATGGCTGATCAACTGTGTGAGGGGCGTTGGTCCGAAGCCAATCTATCATTCGTYGGGATA

TCCATCACCCCTGAATTCATCGATGAGGCCCTCAGAGGCTTTTGGGAGTCTTTTGCCAAA

AGAGCCGTTGGCTACAAAGAGGTTTACGTTCAGGTTGATCGAGATCCTAAAGTTGCTTGG

GAAGGAGCGCTCAATGGTTACCTCGGCAAATATTCTGGCGTAAAAAATGTTCATTTTGCG

CCGAACAACGCCACGATTGGAAAGCCCCCACGCCATATCGACATTTTGCTTCGGAAAAAA

TTTAGCGGCGAACTTCTTGCGACGCTCGAAGGTGTGGCAATTTCAAACGGTGCAACTGGT

TGGCTTGCGCTCGCATGTGTAAACCTTCTCTTGAAGGCAAGACAAGATATTGATGTCTTC

TTGTTTGGGGCAGGAAAAGTCGCCGAGGCTGTGATTCTTGCCCTCAATCATGGTGCGGCT

GCAAAAATCAAAACCATGGCAGTGCTCAGCCAAGCTCCAATTGAAGGTGGGGTTCCAGCT

CGATGCTGTCATTGATCGGAAGGTTATACCAAAAGCCAAACTCGTCATAACAGCAACAAA

TTCTGAGGAGCTCGGTACTCGAGGCCGACGAAATTGCGCCAAATGCAGTAACCATATCCC

TCGGAAAAGATGAATTGCCCGCCGCCTACTTCGATCGCCTTTTGAACGCAGAGGGTCTGA

TTATTGGCGACGATCTGGATGCGATCGAATCGCGCAATGTCAATTCCCTGGTGCTTTACT

ACTCGAAACGTGATTTGAAGCTGACAGAACATGGAAGGGATCATTGGATAAAGAACTACG

CCAATGTCCTTGCTGATCCAGCTCTCATGGAGGAACTTAAGACATGAGAGGGGCCAGCCA

ACTTTTCATCGGTTGGCCTTGCCAGCCTAGACTTGGCGATGGCCGGCCGACTCTACGAAA

CTCTTACTACGAAACTCTCCCACCCCCAGTAGACAAAACCCTCTTAGGATTGCGCTCTTC

AGAAGGTGCGCCTCCCCAGTCACAACCAGAAAGTCACTCAACTTGGCTCAAGTACCCTAC

TTAGTTTGTCTCTCTGTCGTGTTTAGTATCAGCAACAATAACAAATATTGTAAATAACAT

ATATTTATATGGTAATTATATGGCATGTAAGCGTGACAACGATTATGTTTTCCATAGTTT

GATAAACTCAACACCCGAAAAAGCCGGTGACCAAAGGGTTGGGACCTCTTTGCACGAACT

CCTATGGCTGCCAGACTATCTACAGAGCTTTTTCGGAAGCTGACGCAGCATCTGATCGCC

ACACAATCCTCGCGGACGTGGATGGCGTGGCTACATGCAAGCAGTTTGACTGAGAGTTTT

CGAGTGACTATTTTAAAAGGACAATATTTCGTCCCAAGCGATAAAACTCGCTCACATGAC

TAAGTCATTGCTTTAATTCAACTGCGTGAAAAGATTTAAAGGGCAGAACTGTTTGTGTTT

CGTATCCAGTTCACCGGCAATTATTGGCTACGCATGAAGCTCATTATCGTCGATTCTTTA

CCCAAACACTGATGCCATTCCAAATGAGTTGGGCCAAAGATGTACTCATGTCGGACAGGC

GCGATTTGGACTTCGTGATCCTCAAGTCTATCGCAGTGCTGCTTTATTACAGGCTATGCT

TCCACCCTCCATTATTTGGTCAACTGGACCTAGCTTTTGACAAGAATAGGCCAGCGTTCA

TCTACCTATCTTTCAAAAACATGCAGTTGCTGCTGCGTAAAGGATGTAACATCGGATTTG

GTGTGGAGACAGTGCTCGCTACGACTATACTGCCTTACTGTAGTGATCTGTCCCGCCAAG

CGATGAGGGGAGCGGCCTGCCTCACGTGAGCGTCAATCCATACGGTTGCATGTTCGTTTG

TTTCAAAGGCATTCCCAAGAAGGGTTTCCAGCTTGACAACTTTCTCATCGGTGGGTGTGA

ACTCGCCGGTTAGTGAGTCACCGCCCATTTTGGGAACTACCAGGGGGTGCCAACGACCTC

CTCCGTCGACATCTCAGACCAATTTCAATTGCCCAACTGCACCGTTCTTTCTATGTTGAA

GTCATGGGCGACTATGAAATCTCCATCTTTGGTCGGTACTACCTCGATCTCGACCATATT

GAAAAGCCTGAAAGATTTGCAAACTGCCCCTACACTGTTTTCCGGCCAGTGTGGCCCATG

GAATCCACGGTGAACTATCAATTGGCGGTCGGTTGAAGTCTTTCGAGCGATAAGCCAATT

GGCGAGGCAGTGTTTCGCGATCGCATCTTTAAGAGGAAAGTCCGATGCAAATCTCCATGT

AGACCCAGTCGTTTCGGCCAAGGCGGCCTCTAAATCAACTTGAGCAGTAGWTGTGTTAAC

GGAATCTGTTTTGACTGCCATTCTGTAAGAAAYGTCAGCAAAGGAAAAGCTAGTTAGAGA

AGGAGAGCTTGCTCAATAGTGTGTCTATTCTCAAATGAACTATACTAGCGCAATGAGTTG

GTGTTGTTCGCTGATGGGAAAAAGGAAACGCATCCCCCTCTTCTACAAAATTCTGTGGGC

CCGGAGGGACTCCGTATGAAAGTGCACAGCATGTGGACGCATCACCATGCAATTAGCAAG

CTTAGCCAATGTCGTGTCTGTCAGAGTGTGCAGCATGTGAGCCAAGCGCAATTACAATAA

AAAAATAATATAAAAACTCTGCCGAGGTCCGAACCGTCTCAAATGCTTTGAGGGCCATCC

AAGCCAAGCGCACCTAACGCCTAGCACACCATTGTTGCGCCGCTCGAGCATTAATGGTTC

GGGACCTGGTGTCATCTGGAAACCTATGAAGCATGGACACGGCAAAATCAGCGCCGTGGC

GGTGTCAGACACGAGGGACACGTC

>sa3953_2

tctccaaatttccctcaccttgaaGTATCAATTTGATCTAGAAGCCAATCCAAACTCATT

GAAAGGCCTGTCATCAACCTGTCAACACGGTTCAATGTTTGCTCGAGTGGCGCAGCAATG

GTGTGCTAGGCGTTAGGTGCGCCTGGCTTGGATAGCCCTCAAAGCATTTGAGACAGTTCG

GACCTCGGCAGAGATTTTATATTATTTTTTTGTTGTAATCGTGCTTGGCCCACATGCTGC

ACACTCCGACAGACACGGCATTGACTAAACCCGCTAATTGCGTGGTGATGCGTCCACGTG

CTGCGCACTTTCATGCGGAGTCCCCCCGAGCCCGTAGAATTTTGTAGAAGAGGGGGACGC

GTTTTCTTTTTCCCATCAGCAAACAACACCAACTCACTGTGCAAGTATAGTTCATCTGAG

AACAGACACACTGCTGAGCAAGCTCTCCTTCTCCAACTAGCTTTTTCTTTGCTGACATTT

CCTACAGAATGGCAGCAAAACAGATTCCGTTAACACAACTGCTGCTCAGGTTGATTTAGA

GGCCGCCTTGGCCGAAGTGACCGGGTCTACATGGAGATTTGCATCAGACTTTCCTCTTAA

AGATGCGATCGCGAAACACTGCCTCGCCAATTGGCTTATCGCTCGAAAGACTTCAAGCAA

CCACCAATTGATAGTTCACCGTGAATTTCATGGGCCACACCTGCCGGAAAACAGCGTAGG

GGCAGTCTGCAAATCTCTCAGACTTTTTAATATGGTCGAGATCAACGTAGTACCGACCAA

AGATAGAGATTTCATAGTCGTCCACGACTTCAACACAGAAAGAACGACGCAGTTAGGCAA

TTGAAATTGGTCTGATATGTCGACGAAAGAGGCCGTTGGCACCCCCCTGGTAGTTCCCAT

AATGGGCGGTGACTCCCTAACTAGCGAGTTCACACCCACCGATGAGAAAGTTGTCAAGCA

AGAAACCCTTCTCAGGAATGCCTTTGAAACAAACAAACAGGCAACCATACGGATTGACGC

TCACGAGAGGCAGGCCGCTCCCCTCGTCGCTTAGCTGAGCCATAGGCCGGCCTATCACGA

CAAGACAATCGTTATCTTTCATACATATAGATACAACAATGGACACGAGTTCGCCTTGGC

GGTCGATCAAGCCGGCGCTACAGAAAATTGGCACGAAACTGTAGCGCTAATGCCAAACAT

TCTGCCGAATGAACTCTCTAGGCTCGCGAAGCAATATCCACCAAATCGCACGTCGCATTT

TTACGACCTCGTTGAGGCCGGCAAGAATTGGTTCGCGTCTGTCTTCTCCCAGAATATGAT

GGTGGTCGTCACTTTCCTCAAGATTGCGGGCACTGGCGAAGGACAGACTAACAAAGTCGT

TGATCCAAATGGAAATGAGGTTCTGCATCCTAAAGAGGAGGAGGAATATCTCCACGATTC

AGCTTCAATCAAAATCGTGAAATGAATCTGCGCCACCCATCCCGGAATCGAGATTGGAGG

CGTCACACGAACTTATGCTTATAAGAGTCGAGGACATCGCTACAAAATCGATCTTTGGAC

GGGTCAACCGAAGCGGTGGCCGACCCACGCGACGAAGTATATCTGGGAAGATTTCGCCAA

GCCTGGAAATGCGCTCAGATGGGGTCAAACGATTATCTCTGATCGCATTGGCGACGACCT

TGCAGCTTGTGTGCTTCAAAGCCGTTGAGAAAATTTTGTCAGCAATCATGAATATCCCGC

TGTTCATGTCGATTAGATAGGCGATCTGTGTCTTAGTCTAAGCTTCCGTTTAACGCATCT

TTGCTACTTTAATGTATTTTTTTAATGATATTGTTGGAGTTTTCAGCACATCTTAATCGC

AACTAGATATTTTTTTTTGAATCGGATTCGACTTCAAGTAGATGTACTTCTTTGTCTCTC

GATAAATCTAATGACCCTGCGAGCCGGCCTCTCgcctTAAACTAATTTCTGAGAGTTAGG

TCGGCGCCTCTCGTGGTTTCCATGTTCTATGGAAACGGCCTCTCGGGACTTGCCTTCCCG

CAACTCATCGAGCTTGCCTGGCCCGCTCGGGTGGTGGCGCAAGCCATCGTTAAATCGTTC

GTAATCCTCTACTCTCGCATAAATGAGAGGGGAATCCAAAGATGATCACTTTTCAGCTAA

TTGGAATCTGTAATCCCAAAAGAGGCAAGCACTATGTGGGGTCCACAACTTATAGAATTT

TGGCCTAACCATTCTGCATAATGGATCATGACATGGGCCATTTATGGCCGCAAAAACTGG

CACAAATTCCAACCTGTTCAGCACAATGAACAAGTTCTTCCAATTGGAATAAAGGAGGTT

AAAATTATTCGAGTAGACATGACGTGCCAGCACGCCACTAGCCTCGCATTGATGCCACCC

TTTCTTGCGCACACTTATATAATGCTGAGCTAATAATAGAGATAAAAATAGTCAGCTTGT

AAACTGGACCTTTACTACTGCCCCTAATCTCAAGATCAAAGCGACATATACCTCCTCTCT

TTCAAGTCTTTTTGCAACTTGTTCCAAAGCAAGCGTTCATGAATCTGCTAGGCAACCTTC

ACGAAAATGGCTGATCAACTATGTGAGGGGCGTTGGTCCGAAGCCGATCTATCATTCATT

GGGATATCCATCACCCCTGAATTCATCGATGAGGCCCTCAGAGGCTTTTGGGAGTCTTTT

GCCAAAAGAGCCGTTGGCTACAAAGAGGTTTACGTTCAGGTTGATCGAGATCCTAAAGTT

GTCTGGGAAGGAGCGCTCAATGGTTACCTCGGCAAATATTCTGGCGTCAAAAATGTTCAT

TTTGCGCCGAACAACGCCGCGATTGGAAAGCCCCCACGCCATATCGACATTTTGCTTCGG

AAAAAATTTAGCAGCGAACTTCTTGCGACGCTCGAAGGTGTGGCAATTTCAAACGGTGCA

ACTGGTTGGCTTGCGCTCGCATGCGTAAACCTTCTCTTGAAGGCAAGACAAGATATTGAT

GTCTTCTTGTTTGGGGCGGGAAAAGTCGCCGAGGCTGTGATTCTTGCCCTCAATCATGGT

GCGGCTGCAAAAATCAAAACCATGGCAGTGCTCAGCCAAGCTCCAATTGAAGGTGGGGTT

CCAGCTCGATGTCGTCATTGATCAGAAGGTTATACCAAAAGCCAAACTCGTCATAACAGC

AACAAATTCCGAGGAGCTCGGTACTTGAGGCCGACGAAATTGTGCCAAATGCAGTAACCA

TATCCCTCGGAAAAGATGAATTGCCCGTCGCCTACTTCGATCGCCTTTTGAACGCAGAGG

GTCTGATTATCGGCGACGATCTGGATGCGATCGAATCATGCAATGCCAATTCCCTGGTGC

TTTACTACTCGAAATGTGATTTGAAGCTGACAGAACATAGAAGGGATCATTGGATAAAGA

ACTACGCCAATGTCCTTGCTGATCCAGCTCTCATGGAGGAACTTAAGACATGGGAGGGGC

CAGCCAACTTTTCATCGGTTGGCCTTGCCAGCCTAGACTTGGCGATGGCCGGCCGACTCT

ACGAAACTCTTACTGCGAAACTCTCCCACCCCCAGTAGACAAGACCCTCTTAGGATTGCG

CTCTTCAGAAGGTGCGCCTCCCCAGTCACAACCGGAAAGTCACTCAACTTGGCTCAAGTA

CCCTACTTAGTTTGTCTCTCTGTCGTGTTTAGTATCAGCAACAATAACAAATATTGTAAA

TAACATATATTTATATGGTAATTATATGGCATGTAAGCGTGACAACGATTATGTTTTCCA

TAGTTTGATAAACTCAACACCCGAAAAAGCCGGTGACCAAAGGGTTGGGACCTCTTTGCA

CGAACTCCTATGGCTGCCAGACTATCTACAGAGCTTTTCGGAAGCTGACGCAGCATCTGA

TCGCCACACAATCCTCGCGGACGTGGATGGCGTGGCTACACGCAAGCAGTTTGACTGAGA

GTTTTTGAGTGACTAttttaaaaggacaatatttcgtcccaagcgataaaactcgctcac

atgactaagtcgttgctttaattcaactgcgtgaaaagatttaaagggcagaactgtttg

TGTTTCGTATCCAGTTCACCTGCAATTATTGGCTACGCATGAAGCTCATTATCGTCGATT

CTTTACCAAAACACTGATGCCATTCCAAATGAGTTGGGCCAAAGATGTACTCATGTCGGA

CAGGTGCGATTTGGACTTCGTGATCCTCAAGTCTATCGCAGTGCTGCTTTATTACAGGCT

ATGCTTCCACCCTCCATTATTTGGTCAATTGGACCTGGCTTTTGACAAGAATAGGCCAGC

GTTCATCTATCTATCTTTCAAAAACATGCAGTTACTGCTGCGTAAAGGATGTAACATCGG

ATTTGGTGTGGAGACGGTGCTCACTACGACTATACTGCCTTACTGTAGTGATCTGTCCCG

CCAAGCGACGAGGGGAGCGGCCTGCCTCTCGTGAGCGTCAATCCATATGGTTGCATGTTC

GTTTGTTTCAAAGGCATTCCCAAGAAGGGTTTCCAGCTTGACAACTTTCTCATCGGTAGG

TGTAAACTCGCCGGTTAGTGAGTCACTGCCCATTTTGGGAACTACTAAGGGGGTGCCAAC

GACCTCCTCCGTCGACATCTCAGACCAATTTCAATTGCCCAACTGCGCCGTTCTTTCTAT

GTTGAAGTCATGGGCGACTATGAAATCTCCATCTTTGGTCAGTACTACCTCGATCTCAAC

TATATTGAAAAGCCTGAGAGATTTGCAAACTGCCCTACACTGTTTTCCGGCAAGTGTGGC

CCATGGAATCCACGGTGAACTATCAATTGGCGGTCGCTTGAAGTCTTTCGAGCGATAAGC

CAATTGGCGAGGTAGTGTTTCGCGATCGCATCTTTAAGAGGAAAGTCCGATGCAAATCTC

CATGTAGACCCGGTCGCTTCGGCCAAGACGGCCTCTAAATCAACTTGAGCAGAAGTTGTG

TTAAGGGAATCTGTTTTGACTGCCATTCTGTAAGAAATGTCAGCAAAGGAAAAGCTAGTT

GGAGAAGGAGAGCTTGCTCAATAGTGTGTCTATTCTCAGATGAACTATACTAGCGCAATG

AGTTGGTGTTGTTCGCTGATGGGAAAAAGGAAACGCATCCCCCTCTTCTACAAAATTCTA

TGGGCCCGGAGGGACTCTGTATGAAAGTGCACAGCATGTGGACGCATTACCATGCAATTA

GCGAGCTTAGCCAATGCCGTGTCTGTCAGAGTGTGCAGCATGTGGGCCAAGCGCAATTAC

AATAAAAAAATAATATAAAAACTTTGCCAAGGTCCGAACCGTCTCAAATGCTTTGAGGGC

CATCCAAGCCAAGCGCACCTAACGCCTAGCACACCATTGTTGCGCCGCTCGAGCATTAAT

GGTCCGGGACCTGGTGTCATCTGGAAACCTATGAAGCATGGACACGGCAAAAtCAGCgcc

gtggc

>sa3953_1

TATCAATTTGATCTAGAAGCCAATCCAAACTCATTGAAAGGCCTGTCATCAACCGGTCAA

CACGGTTCAATGTTTGCTCGCGTGGCACAGCAATGGTGTGCTAGGCGTTAGGTGCGCCTG

GCTTGGATGGCCCTCAAAGCATTTGAGGCAGTTCGGACCTCGGCAGAGATTTTATATTAT

TTTTTTGTTGTAATCACGCTTGGCCCACATGCTGCACACTCCGATAGACACGGCATTGGC

TAAACCCGCTAATTGCGTGGTGATGCGTCCACGTGCTGCGCACTTTCAGGCGGAGTCCCC

CCGATTCCATAGAATAAGAGGGGGACGCGTTTCCTTTTTCCCATCAGCAAACAACACCAA

CTCACTGTGCTAGTATAGTTCATCTGAGAACAGACACACTGCTGAGCAAGCTCTCCTTCT

CCAACTAGCTTTTTCTTTGCTGACATTTCCTACAGAATGGCAGCAAAACAGATTCCGTTA

ACACAACTACTGCTCAGGTTGATTTAGAGGCCGCCTTGGCCGAAGCGATCGGGTCTACAT

GGAGATTTGCATCAGACTTTCCTCTTAAAAATGCAATCGCGAAACACTGCCTCGCCAATT

GGCTTATCGCTCGAAAGACTTCAAGCAACCACCAATTGATAGTTCACCGTGAATTCCATG

GGCCACACCTGCCGGAAAACAGCGTAGGGGCAGTCTGCAAATCTCTCAGACTTTTTAATA

TGGTCGAGATCAACGTAGTACCGACCAAAGATAAAGATTTCATAGTCGTCCACGACTTCA

ACACAGAAAGAACGACGCAATTAGGCAATTGAAATTGGTTTGATATGTCGACGAAAGAGG

CTGTTGGCACCCCCCTGGTAGTTCCCATAATGGGCGGTGACTCCCTAACCAGCGAGTTCA

CACCCACCGATGAGAAAGTTGTCAAGCAAGAAACCCTTCTCAAGAATGCCTTTGAAACAA

ACAAACAGGCAACCATACGGATTGACGCTCACGAGAGGCAGGCCGCTCCCCTCGTCGCTT

GGCTGAGCCATAGGCCggcctatCACGACAAGACAATCGTTATCTTTCATACATATGGAT

ACAACAATGGACACGAGTTCGCCTTGGCGGTCAATCAAGCCGGCGCTACAAAAAATTGGC

GCGAAACTGTAGCGCTAATGCCAAACATTCTGCCGAATGAACTCTCTAGGCTCGCGAAGC

AATATCCACCAAATTGCACGTCGCATTTTTACGACCTCGTTGAGGCCAGCAAGAATTGGT

TCGCATCTGTCTTCTCCCAGAATATGATGGTGGTCGTCACTTTCCTCAAGATTGCGGGCA

CTGGCGAAGGACAGACTAACAAAGTCGTTGATCCAAATGGAAATGAGGTTCTGCATCCTA

AAGAGAAGGAGGAATATCTCCGCGATTCAGCTTCAATCGAAATCGCGAAATGAATCTGCG

CCACCCATCCCGGAATCGAGATTGGAGGCGTCACACGAACTTATGCTTATAAGAGTCGAG

GACATCGCTACAAAATCAATCTTTGGACGGGTCAACCGAAGCGGTGGCCGACCCACGCGA

CGAAGTACATCTGGGAAGATTTCGCCAAGCCTGGAAATGCGCTCAGATGGGGTCAAACGA

TTATCTCTGATCGCATTGGTGACGACCTTGCAGCTTGTGTGCTTCAAAGCTGTGGAGAAA

ATTTTGTCAGCAGTCATGAATATCCTGCTGTTCATGTCGATTAGATGGGCGATCTGTGTC

TTAGTCTAAGCTTCCGCTTAACGCATCTTTGCTACTTTAATGTATTTTTTTAATGATATT

GTTGGAGTTTTGAGCACATCTTAATCGCAAGTAGATATTTTTTTTGAATCGGATTCGACT

DCAAGCAGATGTACTTCTTTGTCTCTCGATAAATCTAATGACCCTGCGACCGGCCTCTCG

CCTTAAACTAATTTCTGAGAGTTAGGTCGGCGCCTCTCGTGGTTTCCATGTTCTATGGAA

ACGGCCGCTCGGGACTTGCCTTCCCGCAACTCATCGAGCTTGCCTGGCCCGCTCGGGTGG

TGGCACAAGCCATCGTTAAATCGTTCGTAATCCTCTACTCTCGCATAAATGAGAGGGGAA

TCCAAAGATGATCACTTTTCAGCTAATTGGAATCTGCAATCCCAAAAGAGGCAAGCACTA

TGTGGGGTCCACAACTTATAGAATTTTGGCCTAACCATTCTGCATAATGGATCATGACAT

GGGCCATTTATGGCCGTAAAAACTGGCACAAATTCCAACCTATTCAGCACAATGAACAAG

TTCTTCCAATTGGAATAAAGGAGGTTAAAATTATTCGAGTAGACATGACGTGCTGGCATG

CCACTAGCCTCGCATTGATGCCACTCTTTCTTGCGCACACTTATATAATGCTGAGCTAAT

AATAGAGATAAAAATAGTCAGCTTGTAAACTGGACCTTTATTACTGCCCCTAATCTCAAG

ATCAAAGCGACATATACCTCTTCTCTTTCAAGTCTTTTTGCAACTTGTTCCAAAGCAAGC

GTTTGTGAATCTGCCAGGCAGCCTTCACGAAAATGGCTGATCAACTGTGTGAGGGGCGTT

GCTCCGAAGCCAATCTATCATTCGTTGGGATATCCATCACCCCTGAATTCATCGATGAGG

CCCTCAGAGGCTTTTGGGAGTCTTTTGCCAAAAGAGCCGTTGGCTACAAAGAGGTTTACG

TTCAGGTTGATCGAGATCCTAAAGTTGCCTGGGAAGGAGCGCTCAATGGTTACCTCGGCA

AATATTCTGGCGTAAAAAATGTTCATTTTGCGCCGAACAACGCCACGATTGGAAAGCCCC

CACGCCATATCGACATTTTGCTTCGGAAAAAATTTAGCGGCGAACGTCTTGCGACGCTCG

AAGGTGTGGCAATTTCAAACGGTGCAACTGGTTGGCTTGCGCTTGCATGTGTAAACCTTC

TCTTGAAGGCAAGACAAGATATTGATGTCTTCTTGTTTGGGGCAGGAAAAGTTGCCGAGG

CTGTGATTCTTGCCCTCAATCATGGTGCGGCTGCAAAAATCAAAACCATGGCAGTGCTCA

GCCAAGCTCCAATTGAAGGTGGGGTTCCAGCTTGATGCCGTCATTGATCGGAAGGTTATA

CCAAAAGCCAAACTCGTCATAACAGCAACAAATTCCGAGGAGCTCGGTACTCGAGGCCGA

CGAAATTGCGCCAAATGCAGTAACCATATCCCTCGGAAAAGATGAATTGCCCGCCGCCTA

CTTCGATCGCCTTTTGAACGCAGAGGGTCTGATTATTGGCGACGATCTGGATGCGATCGA

ATCGCACAATGTCAATTCCCTGGTGCTTTACTACTCGAAACGTGATTTGAAGCTGACAGA

ACATGGAAGGGATCATTGGATAAAGAACTACGCCAATGTCCTTGCTGATCCAGCTCTCAT

GGAGGAACTTAAGACATGAGAGGGGCCAGCCAACTTTTCATCGGTTGGCCTTGCCAGCCT

AGACTTGGCGATGGCCGGCCGACTCTACGAAACTCTTACTGCGAAACTCTCCCACCCCCA

GTAGACAAAACCCTCTTAGGATTGCGCTCTTCAGAAGGTGCGCCTCCCCAGTCACAACCG

GAAAGTCACTCAACTTGGCTCAAGTACCCTACTTAGTTTGTCTCTCTGTCGTGTTTAGTA

TCAGCAACAATAACAAATATTGTAAATAACATATATTTATATGGTAATTATATGGCATGT

AAGCGTGACAACGATTATGTTTTCCATAGTTTGATAAACTCAACACCCGAAAAAGCCGGT

GACCAAAGGGTTGGGACCTCTTTGCACGAACTCCTATGGCTGCCAGACTATCTACAGAGC

TTTTTCGGAAGCTGACGCAGCATCTGATCGCCACACAATCCTCGCGGACGTGGATGGCGT

GGCTACATGCAAGCAGTTTGACTGAGAGTTTTCGAGTGACTGTTTTAAAAGGACAATATT

TCGTCCCAAGCGATAAAACTCGCTCACATGACTAAGTCGTTGCTTTAATTCAACTGCGTG

AAAAGATTTAAAGGGCAGAACTGTTTGTGTTTCGTATCCAGTTCACCGGCAATTATTGGC

TACGCATAAAGCTCATTATCGTCGATTCTTTACCCAAACACTGATGCCATTCCAAATGCG

TTGGGCCAAAGATGTACTCATGTCGGACAGGCGCAATTTGGACTTCGTGATCCTCAAGTC

TATCGCAGTGCTGCTTTATTACAGGCTATGCTTCCACCCTCCATTATTTGGTCAACTGGA

CCTAGCTTTTGACAAGAATAGGCCAGCGTTCATCTATCTATCTTTCAAAAACATGCAGTT

GCTGCTGCGTAAAGGATGTAACATCGGATTTGGTGTGGAGACGGTGCTCGCTACGACTAT

ACTGCCTTACTGTAGTGATCTGTCCCGCCAAGCGATGAGGGAAGCGGCCTGCCTCTCGTG

AGCGTCAATCCATACGGTTGCATGTTCGTTTGTTTCAAAAGCATTCCCAAGAAGGGTTTC

CAGCTTGACAACTTTCTCATCGGTGGGTGTGAACTCGCCGGTTAGTGAGTCACCGCCCAT

TTTGGGAACTACCAGGGGGGTGCCAACGACCTCCTCCGTCGACATCTCAGACCAATTTCA

ATTGCCCAACTGCACCGTTCTTTCTATGTTGAAGTCATGGGCGACTATGAAATCTCCATC

TTTGGTCGGTACTACCTCGATCTCGACCATATTGAAAAGCCTAAAAGATTTGCAAACTGC

CCCTACACTGTTTTCCGGCAAGTGTGGCCCATGGAATCCACGGTGAACTATCAATTGGCG

GTCGCTTGAAGTCTTTCGAGCGATAAGCCAATTGGCGAGGCAGTGTTTCGCGATCGCATC

TTTAAGAGGAAAGTCCGATGCAAATCTCCATGTAGACCCAGTCGTTTCGGCCAAGGCGGC

CTCTAAATCAACTTGAGCAGCAGTTGTGTTAACGGAATCTGTTTTGACTGCCATTCTGTA

AGAAATGTCAGCAAAGGAAAAGCTAGTTAGAGAAGGAGAGCTTGCTCAATAGTGTGTCTA

TTCTCAAATGAACTATACTAGCGCAATGAGTTGGTGTTGTTCGCTGATGGGAAAAAGGAA

ACGCATCCCCCTCTTCTACAAAATTCTGTGGGCCCGGAGGGACTCCGTATGAAAGTGCAC

AGCATGTGGACGCATCACCATGCAATTAGCAAGCTTAGCCAATGTCGTGTCTGTCAGAGT

GTGCAGCATGTGAGCCAAGCGCAATTACAATAAAAAAATAATATAAAAACTCTGCCGAGG

TCCGAACCGTCTCAAATGCTTTGAGGGCCATCCAAGCCAAGCGCACCTAACGCCTAGCAC

ACCATTGTTGCGCCGCTCGAGCATTAATGGTTCGGGACCTGGTGTCATCTGGAAACCTAT

GAAGCATAGACACGGCAAAATCAGCGCCGTGGCGGTGTCAGACACGaggacacgtcgggg

acacgtaca

>sa3951_1

agccaATCCAAACTCATTGAAAGGCCTGTCATCAACCGGTCAACACGGTTCAATGTTTGC

TCGCGTGGCGCAGCAATGGTGTGCTAGGCATTAGGTGCGCCTGGCTTGGATGGCCCTCAA

AGCATTTGAGGCAGTTCGGACCTCGGCAGAGATTTTATATTATTTTTTTGTTGTAATCGC

GCTTGGCCCACATGCTGCACACTCCGATAGACACGGCATTGGCTAAACCCGCTAATTGCG

TGGTGATGCGTCCACGTGCTGCGCACtTTCATGCGGAGTCCCCCCGAGTCCATAGAATAA

GAGGGGGACGCGTTTCCTTTTTCCCATCAGCAAACAACACCAACTCACTGTGCTAGTATA

GTTCATCTGAGAACAGACACACTGCTGAGCAAGCTCTCCTTCTCCAACTAGCTTTTTCTT

TGCTGACATTTCCTACAGAATGGCAGCAAAACAGATTCCGTTAACACAACTACTGCTCAG

GTTGATTTAGAGGCCGCCTTGGCCGAAGCGATCGGGTCTACATGGAGATTTGCATCAGAC

TTTCCTCTTAAAAATGCAATCGCGAAACACTGCCTCGCCAATTGGCTTATCGCTCGAAAG

ACTTCAAGCAACCACCAATTGATAGTTCACCGTGAATTCCATGGGCCACACCTGCCCGAA

AACAGCGTAGGGGCAGTCTGCAAATCTCTCAGACTTTTTAATATGGTCGAGATCAACGTA

GTACCGACCAAAGATAAAGATTTCATAGTCGTCCACGACTTCAACACAGAAAGAACGACG

CAGTTAGGCAATTGAAATTGGTTTGATATGTCGACGAAAGAGGCTGTTGGCACCCCCCTG

GTAGTTCCCATAATGGGCGGTGACTCCCTAACCAGCGAGTTCACACCCACCGATGAGAAA

GTTGTCAAGCAAGAAACCCTTCTCAAGAATGCCTTTGAAACAAACAAACAGGCAACCATA

CGGATTGACGCTCACGAGAGGCAGGCCGCTCCCCTCGTCGCTTGGCTGAGCCATAGGCCG

GCCTATCACGACAAGACAATCGTTATCTTTCATACATATGGATACAACAATGGACACGAG

TTCGCCTTGGCGGTCAATCAAGCCGGCGCTACAAAAAATTGGCGCGAAACTGTAGCGCTA

ATGCCAAACATTCTGCCGAATGAACTCTCTAGGCTCGCGAAGCAATATCCACCAAATTGC

ACGTCGCATTTTTACGACCTCGTTGAGGCCAGCAAGAATTGGTTCGCATCTGTCTTCTCC

CAGAATATGATGGTGGTCGTCACTTTCCTCAAGATTGCGGGCACTAGCGAAGGACAGACT

AACAAAGTCGTTGATCCAAATGGAAATGAGGTTCTGCATCCTAAAGAGAAGGAGGAATAT

CTCCGCGATTCCGCTTCAATCGAAATCGCGAAATGAATCTGCGCCACCCATCCCGGAATC

GAGATTGGAGGCGTCACACGAACTTATGCTTATAAGAGTCGAGGACATCGCTACAAAATC

AATCTTTGGACGGGTCAACCGAAGCGGTGGCCGACCCACGCGACGAAGTACATCTGGGAA

GATTTCGCCAAGCCTGGAAATGCGCTCAGATGGGGTCAAACGATTATCTCTGATCGCATT

GGTGACGACCTTGCAGCTTGTGTGCTTCAAAGCCGTGGAGAAAATTTTGTCAGCAGTCAT

GAATATCCTGCTGTTCATGTCGATTAGATGGGCGATCTGTGTCTTAGTCTAAGCTTCTGC

TTAACGCATCTTTGCTACTTTAATGTATTTTTTTAATGATATTGTTGGAGTTTTGAGCAC

ATCTTAATCGCAAGTAGATATTTTTTTTGAATCGGATTCGACTTCAAGCAGATGTACTTC

TTTGTCTCTCGATAAATCTAATGACCCTGCGAGCCGGCCTCTCACCTTAAACTAATTTCT

GAGAGTTAGGTCGGCGCCTCTCGTGGTTTCCATGTTTTATGGAAACGGCCGCTCGGGACT

TGCCTTCCCgcaactcatcgagcttgcctggcccgctcgggtggtggcaCAAGCCATCGT

TAAATCGTTCGTAATCCTctactctcGCATAAATGAGAGGGGAATCCAAAGATGATCACT

TTTCAGCTAATTGGAATCTGCAATCCCAAAAGAGGCAAGCACTATGTGGGGTCCACAACT

TATAGAATTTTGGCCTAACCATTCTGCATAATGGATCATGACATGGGCCATTTATGGCCG

CAAAAACTGGCACAAATTCCAACCTATTCAGCACAATGAACAAGTTCTTCCAATTGGAAT

AAAGGAGGTTAAAATTATTCGAGTAGACATGACGTGCTGGCACGCCACTAGCCTCGCATT

GATGCCACTCTTTCTTGCGCACACTTATATAATGCTGAGCTAATAATAGAGATAAAAATA

GTCCGCTTGTAAACTGGACCTTTATTACTGCCCCTAATCTCAAGATCAAAGCGACATATA

CCTCTTCTCTTTCAAGTCTTTTTGCAACTTGTTCCAAAGCAAGCGTTTGTGAATCTGCCA

GGCAGCCTTCACGAAAATGGCTGATCAACTGTGTGAGGGGCGTTGGTCCGAAGCCAATCT

ATCATTCGTTGGGATATCTATCACCCCTGAATTCATCGATGAGGCCCTCAGAGGCTTTTG

GGAGTCTTTTGCCAAAAGAGCCGTTGGCTACAAAGAGGTTTACGTTCAGGttGATCGAGA

TCCTAAAGTTGCCTGGGAAGGAGCGCTCAATGGTTACCTCGGCAAATATTCTGGCGTAAA

AAATTTTCATTTTGCGCCGAACAACGCCACGATTGGAAAGCCCCCACGCCATATCGACAT

TTTGCTTCGGAAAAAATTTAGCGGCGAACTTCTTGCGACGCTCGAAGGTGTGGCAATTTC

AAACGGTGCAACTAGTTGGCTTGCGCTCGCATGTGTAAACCTTCTCTTGAAGGCAAGACA

AGAAATTGATGTCTTCTTGTTTGGGGCAGGAAAAGTCGCGGAGGCTGTGATTCTTGCCCT

TAATCATGGTGCGGCTGCAAAAATCAAAACCATGGCAGTGCTCAGCCAAGCTCCAATTGA

AGGTGGGGTTCCAGCTCGATGCCGTCATTGATCGGAAGGTTATACCAAAAGCCAAACTCG

TCATAACAGCAACAAATTCCGAGGAGCTCGGTACTCGAGGCCGACGAAATTGCGCCAAAT

GCAGTAACCATATCCCTCGGAAAAGATGAATTGCCCGCCGCCTACTTCGATCGCCTTTTG

AACGCAGAGGGTCTGATTATTGGCGACGATCTGGATGCGATCGAATCGCGCAATGTCAAT

TCCCTGGTGCTTTACTACTCGAAACGTGATTTGAAGCTGACAGAACATGGAAGGGATCAT

TGGATAAAGAACTACGCCAATGTCCTTGCTGATCCAGCTCTCATGGAGGAACTTAAGACA

TGAGAGGGGCCAGCCAACTTTTCATCGGTTGGCCTTGCCAGCCTAGACTTGGCAATGGCC

GGCCGACTCTACGAAACTCTTACTGCGAAACTCTCCCACCCCCAGTAGACAAAACCCTct

taggattgcGCTCTTCAGAAGGTGCGCCTCCCCAGTCACAACCGAAAAGTCACTCAACTT

GGCTCAAGTACCCTACTTAGTTTGTCTCTCTGTCGTGTTTAGTATCAGCAACAATAACAA

ATATTGTAAATAACATATATTTATATGGTGATTATATGGCATGAAAGCGTGACAACGAAT

ATGTTTTCCATAGTTTGATAAACTCAACACCCGAAAAAGCCGGTGACCAAAGGGTTGGGA

CCTCTTTGCACGAACTCCTATGGCTGCCAGACTATCTACAGAGCTTTTTCGGAAGCTGAC

GCAGCATCTGATCGCCACACAATCCTCGCGGACGTGGATGGCGTGGCTACATGCAAGCAG

TTTGACTGAGAGTTTTCGAGTGACTATTTTAAAAGGACAATATTTCGTCCCAAGCGATAA

AGCTCGCTCACATGACTAAGTCGTTGCTTTAATTCAACTGCGTGAAAAGATTTAAAGGGC

AGAACTGTTTGTGTTTCGTATCCAGTTCACCAGCAATTATTGGCTACGCATAAAGCTCAT

TATCGTCGATTCTTTACCCAAACACTGATGCCATTCCAAATGAGTTAGGCCAAAGATGTA

CTCATGTCGGACAGGCGCGATTTGGACTTCGTGATCCTCAAGTCTATCGCAGTGCTGCTT

TATTACAAGCTATGCTTCCACCCTCCATTATTTGGTCAACTGGACCTAGCTTTTGACAAG

AATAGGCCAGCGTTCATCTATCTATCTTTCAAAAACATGCAGTTGCTGCTGCGTAAAGGA

TGTAACATCGGATTTGGTGTGGAGACGGTGCTCGCTACGACTATACTGCCTTACTGTAGT

GATCTGTCCCGCCAAGCGATGAGGGGAGCGGCCTGCCTCTCGTGAGCGTCAATCCATACG

GTTGCATGTTCGTTTGTTTCAAAGGCATTCCCAAGAAGGGTTTCCAGCTTGACAACTTTC

TCATCAGTGGGTGTGAACTCGCCGGTTAGTGAGTCACCGCCCATTTTGGGAACTACCAGG

GGGGTGCCAACGACCTCCTCCGTCGACATCTCAGACCAATTTCAATTGCCCAACTGCACC

GTTCTTTCTATGTTGAAGTCATGGGCGACTATGAAATCTCCATCTTTGGTCGGTACTACC

TCGATCTCGACCATATTGAAAAGCCTGAAAGATTTGCAAACTGCCCCTACACTGTTTTCT

GGCAAGTGTGGCCCATGGAATCCACGGTGAGCTATCAATTGGCGGTCGCTTGAAGTCTTT

CGAGCGATAAGCCAATTGGCGAGGCAGTGTTTCGCGATCGCATCTTTAAGAGGAAAGTCC

GATGCAAATCTCCATGTAGACCCAGTCGTTTCGGCCAAGGCGGCCTCTAAATCAACTTGA

GTAGCAGTTGTGTTAACGGAATCTGTTTTGACTGCCATTCTGTAAGAAATGTCAGCAAAG

GAAAAGCTAGTTAGAGAAGGAGAGCTTGCTCAATAGTGTGTCTATTCTCAAATGAACTAT

ACTAGCGCAATGAGTTGGTGTTGTTCGCTGATGGGAAAAAGGAAACGCATCCCCCTCTTC

TACAAAATTCTGTGGGCCCGGAGGGACTCCGTATGAAAGTGCACAGCATGTGGACGCATC

ACCATGCAATTAGCAAGCTTAGCCAATGTCGTGTCTGTCAGAGTGTGCAGCATGTGAGCC

AAGCGCAATTACAATAAAAAAATAATATAAAAACTCTGCCGAGGTCCGAACCGTCTCAAA

TGCTTTGAGGGCCATCCAAGCCAAGCGCACCTAACGCCTAGCACACCATTGTTGCGCCGC

TCGAGCATTAATGGTTCGGGACCTGGTGTCATCTGGAAACCTATGAAGCATGGACACGGC

AAAATCAGCGCCGTGGCTGTGTCAGACACAAGGACACGTCGGGGACACGTAcaggacacg

t

>sa3951_2

aagccaatccaaactcattgaaaggCCTGTCATCAACCGGTCAACACGGTTCAATGTTTG

CTCGCGTGGCGCAGCAATGGTGCGCTAGGCGTTAGGTGCGCCTGGCTTGGATGGCCCTCA

AAGCATTTGAGGCAGTTCGGACCTCGGCAGAGATTTTATATTATTTTTTTGTTGTAATCG

CGCTTGGCCCACATGCTGCACACTCCGATAGACACGGCATTGGCTAAACCCGCTAATTGC

GTGGTGATGCGTCCACGTGCTGCGCACTTTCATGCGGAGTCCCCCCGAGTCCATAGAATA

AGAGGGGGACGCATTTCCTTTTTCCCATCAGCAAACAACACCAACTCACTGTGCTAGTAT

AGTTCATCTGAGAACAGACACACTgCTGAGcaaGCTCTCCTTCTCTAACTAGCTTTTTCT

TTGCTGACATTTCCTACAGAATGGCAGCAAAACAGATTCCGTTAACACAACTACTGCTCA

GGTTGATTTAGAGGCCGCCTTGGCCGAAGCGATCGGGTCTACATGGAGATTTGCATCAGA

CTTTCCTCTTAAAAATGCAATCGCGAAACACTGCCTCGCCAATTGGCTTATCGCTCGAAA

GACTTCAAGCAACCACCAATTGATAGTTCACCGTGAATTCCATGGGCCACACCTGCCGGA

AAACAGCGTAGGGGCAGTCTGCAAATCTCTCAGACTTTTTAATATGGTCGAGATCAACGT

AGTACCGACCAAAGATAAAGATTTCATAGTCGTCCACGACTTCAACACAGAAAGAATGAC

GCAGTTAGGCAATTGAAATTGGTTTGATATGTCGACGAAAGAGGCTGTTGGCACCCCCCT

GGTAGTTCCCATAATGGGCGGTGACTCCCTAACCAGCGAGTTCACACCCACCGATGAGAA

AGTTGTCAAGCAAGAAACCCTTCTCAAGAATGCCTTTGAAACAAACAAACAGGCAACCAT

ACGGATTGATGCTCACGAGAGGCAGGCCGCTCCCCTCGTCGCTTGGCTGAGCCATAGGCC

GGCCTATCACGACAAGACAATCGTTATCTTTCATACATATGGATACAACAATGGACACGA

GTTCGCCTTGGCGGTCGATCAAGCCGGCGCTACAAAAAATTGGCGCGAAACTGTAGCGCT

AATGCCAAACATTCTGCCGAATGAACTCTCTAGGCTCGCGAAGCAATATCCACCAAATTG

CACGTCGCATTTTTACGACCTCGTTGAGGCCAGCAAGAATTGGTTCGcatctgtcttctC

CCAGAATATGATGGTGGTCGTCACTTTCCTCAAGATTGCGGGCACTGGCGAAGGACAGAC

TAACAAAGTCGTTGATCCAAATGGAAATGAGGTTTTGCATCCTAAAGAGAAGGAGGAATA

TCTCCGCGATTCAGCTTCAATCGAAATCGCGAAATGAATCTGCGCCACCCATCCCGGAAT

CGAGATTGGAGGCGTCACACGAACTTATGCTTATAAGAGTCGAGGACATCGCTACAAAAT

CAATCTTTGGACGGGTCAACCGAAGCGGTGGCCGACCCACGCGACGAAGTACATCTAGGA

AGATTTCGCCAAGCCTGGAAATGCGCTCAGATGGGGTCAAACGATTATCTCTGATCGCAT

TGGTGACGACCTTGCAACTTGTGTGCTTCAAAGCCGTGGAGAAAATTTTGTCAGCAGTCA

TGAATATCCTGCTGTTCATGTCGATTAGATGGGCGATCTGTGTCTTAGTCTAAGCTTCCG

CTTAACACATCTTTGCTACTTTAATGTATTTTTTTAATGATATTGTTGGAGTTTTGAGCA

CATCTTAATCGCAAGTAGATATTTTTTTTGAATCGGATTCGACTTCAAGCAGATGTACTT

CTTTGTCTCTCGATAAATCTAATGACCCTGCGAGCCGGCCTCTCACCTTAAACTAATTTC

TGAGAGTTAGGTCGGCGCCTCTCGTGGTTTCCATGTTCTATGGAAACGGCCGCTCGGGAC

TTGCCTTCCCGCAACTCATCGAGCTTGCCTGGCCCGCTCGGGTGGTGGCACAAGCCATCG

TtaaatcgttCGTAATCCTCTACTCTCGCATAAATGAGAGGGGAATCCAAAGATGATCAC

TTTTCAGCTAATTGGAATCTGCAATCCCAAAAGAGGCAAGCACTATGTGGGGTCCACAAC

TTATAGAATTTTGGTCTAACCATTCTGCATAATGGATCATgacatgggccatttatggcc

gcaaaaactggcacaaattccaacctattcagcacaatgaacaagttcttccaattggaa

taaaggaggttaaaattattcgagTAGACATGACGTGCTGGCACGCCACTAGCCTCGCAT

TGATGCCACTCTTTCTTGCGCACACTTATATAATGCTGAGCTAATAATAGAGATAAAAAT

AGTCAGCTTGTAAACTGGACCTTTATTACTGCCCCTAATCTCAAGATCAAAGCGACATAT

ACCTCTTCTCTTTCAAGTCTTTTTGCAACTTGTTCCAAAGCAAGCGTTTGTGAATCTGCC

AGGCAGCCTTCACGAAAATGGCTGATCAACTGTGTGAGGGGCGTTGGTCCAAAGCCAATC

TATCATTCGTTGGGATATCCATCACCCCTGAATTCATTGATGAGGCCCTCAAAGGCTTTT

GGGAGTCTTTTGCCAAAAGAGCCGTTGGCTACAAAGAGGTTTACGTTCAGGTTGATCGAG

ATCCTAAAGTTGCCTGGGAAGGAGCGCTCAATGGTTACCTCGGCAAATATTCTGGTGTAA

AAAATGTTCATTTTGCGCCGAACAACGCCACGATTGGAAAGCCCCCACGCCATATCGACA

TTTTGCTTCGGAAAAAATTTAGCGGCGAACTTCTTGCGACGCTCGAAGGTGTGGCAATTT

CAAACGGTGCAACTGGTTGGCTTGCGCTCGCATGTGTAAACCTTCTCTTGAAGGCAAGAC

AAGATATTGATGTCTTCTTGTTTGGGGCAGGAAAAGTCGCCGAGGCTGTGATTCTTGCCC

TCAATCATGGTGCGGCTGCAAAAATCAAAACCATGGCAGTGCTCAGCCAAGCTCCAATTG

AAGGTGGGGTTCTAGCTCGATGCCGTCATTGATTGGAAGGTTATACCAAAAGCCAAACTC

GTCATAACAGCAACAAATTCCGAGGAGCTCGGTACTCGAGGCCGACGAAATTGCGCCAAA

TGCAGTAACCATATCCCTCGGAAAAGATGAATTGCCCGCCGCCTACTTCGATCGCCTTTT

GAACGCAGAGGGTCTGATTATTGGCGACGATCTGGATGCGACCGAATCGCGCAATGTCAA

TTCCCTGGTGCTTTACTACTCGAAACGTGATTTGAAGCTGACAGAACATGGAAGGGATCA

TTAGATAAAGAACTACGCCAATGTCCTTGCTGATCCAGCTCTCATGGAGGAACTTAAGAC

ATGAGAGGGGCCAGCCAACTTTTCATCGGTTGGCCTTGCCAGCCTAGACTTGGCGATGGC

CGGCCGACTCTACGAAACTCTTACTGCGAAACTCTCCCACCCCCAGTAGACAAAACCCTC

TTAGGATTGCGCTCTTCAGAAGGTGCGCCTCCCCAGTCACAACCGGAAAGTCACTCAACT

TGGCTCAAGTACCCTACTTAGTTTGTCTCTCTGTCGTGTTTAGTATCAGCAACAATAACA

AATATTGTAAATAACATATATTTATATAGTAATTATATGGCATGTAAGCGTGACAACGAT

TATGTTTTCCATAGTTTGATAAACTTAACACCCGAAAAAAGCCGGTGACCAAAGGGTTGG

GACCTCTTTGCACGAACTCCTATGGCTGCCAGACTATCTACAGAGCTTTTTCGGAAGCTG

ACGCAGCATCTGATCGCCACACAATCCTCGCGGACGTGGATGGCGTGGCTACATGCAAGC

AGTTTGATTGAGAGTTTTCGAGTGACTATTTTAAAAGGACAATATTTCGTCCCAAGCGAT

AAAACTCGCTCACATGACTAAGTCATTGCTTTAATTCAACTGCGTGAAAAGATTTAACGG

GCAGAACTGTTTGTGTTTCGTATCCAGTTCACCGGCAATTATTGGCTACGCATGAAGCTC

ATTATCGTCGATTCTTTACCCAAACACTGATGCCATTCCAAATGAGTTGGGCCAAAGATG

TACTCATGTCGGACAGGCGCGATTTGGACTTCGTGATCCTCAAGTCTATCGCAGTGCTGC

TTTATTACAAGCTATGCTTCCACCCTCCATTATTTGGTCAACTGGACCTAGCTTTTGACA

AGAATAGGCCAGCGTTCATCTATCTATCTTTCAAAAACATGCAGTTGCTGCTGTGTAAAG

GATGTAACATCGGATTTGGTGTGGAGACGGTGCTCGCTACGACTATACTGCCTTACTGTA

GTGATCTGTCCCGCCAAGCGATGAGGGGAGCGGCCTGCCTCTCATGAGCGTCAATCCATA

CGGTTGCATGTTCGTTTGTTTCAAAGGCATTCCCAAGAAGGGTTTCCAGCTTGACAACTT

TCTCATCGGTGGGTGTGAACTCGCCGGTTAGTGAGTCACCGCCCATTTTGGGAACTACCA

GGGGGGTGCCAACGACCTCCTCCGTCGACATCTCAGACCAATTTCAATTGCCCAACTGCA

CCGTTCTTTCTATGTTGAAGTCATGGGTGACTATGAAATCTCCATCTTTGGTCGGTACTA

TCTCGATCTCGACCATATTGAAAAGCCTGAAAGATTTGCAAACTGCCCCTACACTGTTTT

CCGGCAAGTGTGGCCCATGGAATCCACGGTGAACTATCAATTGGCGGTCGCTTGAAGTCT

TTCGAGCGATAAGCCAATTGGCGAGGCAGTGTTTCGCGATCGCATCTTTAAGAGGAAAGT

CCGATGCAAATCTCCATGTAGACCCAGTCGTTTCGGCCAAGGCGGCCTCTAAATCAACTT

GAGCAGCAGTTGTGTTAACGGAATCTGTTTTGACTGCCATTCTGTAAGAAATGTCAGCAA

AGGAAAAGCTAGTTAGAGAAGGAGAGCTTGCTCAATAGTGTGTCTATTCTCAAATGAACT

ATACTAGCGCAATGAGTTGGTGTTGTTCGCTGATGGGAAAAAGGAAATGCATCCCCCTCT

TCTACAAAATTCTGTGGGCCCGGAGGGACTCCGTATGAAAGTGCACAGCATGTGGACGCA

TCACCATGCAATTAGCAAGCTTAGCCAATGTCGTGTCTGTCAGAGTGTGCAGCATGTGAG

CCAAGCGCAATTACAATAAAAAAATAATATAAAAACTCTGCCGAGGTCCGAACCGTCTCA

AATGCTTTGAAGGCCATCCAAGCCAAGCGCACCTAACGCCTAGCACACCATTGTTGCGCC

GCTCGAGCATTAATGGTTCGGGACCTGGTGTCATCTGGAAACCTATGAAGCATGGACACG

GCAAAATCAGCGTCGTGGCGGTGTCAGACACGAGGACACGTCGGGGACACGtacag

>sa6856_2

ccctcaCCTTGAAGTATCAATTTGATCTAGAAGCCAATCCAAACTCATTGAAAGGCCTGT

CATCAACCGGTCAACACGGTTCAATGTTTGCTCGCGTGGCACAGCAATGGTGTGCTAGGC

GTTAGGTGCGCCTGGCTTGGATGGCCCTCAAAGCATTTGAGGCAGTTCGGACCTCGGCAG

AGATTTTATATTATTTTTTTGTTGTAATCACGCTTGGCCCACATGCTGCACACTCCGATA

GACACGGCATTGGCTAAACCCGCTAATTGCGTGGTGATGCGTCCACGTGCTGCGCACTTT

CAGGCGGAGTCCCCCCGATTCCATAGAATAAGAGGGGGACGCGTTTCCTTTTTCCCATCA

GCAAACAACACCAACTCACTGTGCTAGTATAGTTCATCTGAGAACAGACACACTGCTGAG

CAAGCTCTCCTTCTCCAACTAGCTTTTTCTTTGCTGACATTTCCTACAGAATGGCAGCAA

AACAGATTCCGTTAACACAACTACTGCTCAGGTTGATTTAGAGGCCGCCTTGGCCGAAGC

GATCGGGTCTACATGGAGATTTGCATCAGACTTTCCTCTTAAAAATGCAATCGCGAAACA

CTGCCTCGCCAATTGGCTTATCGCTCGAAAGACTTCAAGCAACCACCAATTGATAGTTCA

CCGTGAATTCCATGGGCCACACCTGCCGGAAAACAGCGTAGGGGCAGTCTGCAAATCTCT

CAGACTTTTTAATATGGTCGAGATCAACGTAGTACCGACCAAAGATAAAGATTTCATAGT

CGTCCACGACTTCAACACAGAAAGAACGACGCAATTAGGCAATTGAAATTGGTTTGATAT

GTCGACGAAAGAGGCTGTTGGCACCCCCTGGTAGTTCCCATAATGGGCGGTGACTCCCTA

ACCAGCGAGTTCACACCCACCGATGAGAAAGTTGTCAAGCAAGAAACCCTTCTCAAGAAT

GCCTTTGAAACAAACAAACAGGCAACCATACGGATTGACGCTCACGAGAGGCAGGCCGCT

CCCCTCGTCGCTTGGCTGAGCCATAGGCCGGCCTATCACGACAAGACAATCGTTATCTTT

CATACATATGGATACAACAATGGACACGAGTTCGCCTTGGCGGTCAATCAAGCCGGCGCT

ACAAAAAATTGGCGCGAAACTGTAGCGCTAATGCCAAACATTCTGCCGAATGAACTCTCT

AGGCTCGCGAAGCAATATCCACCAAATTGCACGTCGCATTTTTACGACCTCGTTGAGGCC

AGCAAGAATTGGTTCGCATCTGTCTTCTCCCAGAATATGATGGTGGTCGTCACTTTCCTC

AAGATTGCGGGCACTGGCGAAGGACAGACTAACAAAGTCGTTGATCCAAATGGAAATGAG

GTTCTGCATCCTAAAGAGAAGGAGGAATATCTCCGCGATTCAGCTTCAATCGAAATCGCG

AAATGAATCTGCGCCACCCATCCCGGAATCGAGATTGGAGGCGTCACACGAACTTATGCT

TATAAGAGTCGAGGACATCGCTACAAAATCAATCTTTGGACGGGTCAACCGAAGCGGTGG

CCGACCCACGCGACGAAGTACATCTGGGAAGATTTCGCCAAGCCTGGAAATGCGCTCAGA

TGGGGTCAAACGATTATCTCTGATCGCATTGGTGACGACCTTGCAGCTTGTGTGCTTCAA

AGCTGTGGAGAAAATTTTGTCAGCAGTCATGAATATCCTGCTGTTCATGTCGATTAGATG

GGCGATCTGTGTCTTAGTCTAAGCTTCCGCTTAACGCATCTTTGCTACTTTAATGTATTT

TTTTAATGATATTGTTGGAGTTTTGAGCACATCTTAATCGCAAGTAGATATTTTTTTTGA

ATCGGATTCGACTTCAAGCAGATGTACTTCTTTGTCTCTCGATAAATCTAATGAcCCTGC

GACCGGCCTCTCGCCTTAAACTAATTTCTGAGAGTTAGGTCGGCGCCTCTCGTGGTTTCC

ATGTTCTATGGAAACGGCCGCTCGGGACTTGCCTTCCCGCAACTCATCGAGCTTGCCTGG

CCCGCTCGGGTGGTGGCACAAGCCATCGTTAAATCGTTCGTAATCCTCTACTCTCGCATA

AATGAGAGGGGAATCCAAAGATGATCACTTTTCAGCTAATTGGAATCTGCAATCCCAAAA

GAGGCAAGCACTATGTGGGGTCCACAACTTATAGAATTTTGGCCTAACCATTCTGCATAA

TGGATCATGACATGGGCCATTTATGGCCGTAAAAACTGGCACAAATTCCAACCTATTCAG

CACAATGAACAAGTTCTTCCAATTGGAATAAAGGAGGTTAAAATTATTCGAGTAGACATG

ACGTGCTGGCATGCCACTAGCCTCGCATTGATGCCACTCTttcttgcgcacacTTATATA

ATGCTGAGCTAATAATAGAGATAAAAATAGTCAGCTTGTAAACTGGACCTTTATTACTGC

CCCTAATCTCAAGATCAAAGCGACATATACCTCTTCTCTTTCAAGTCTTTTTGCAACTTG

TTCCAAAGCAAGCGTTTGTGAATCTGCCAGGCAGCCTTCACGAAAATGGCTGATCAACTG

TGTGAGGGGCGTTGCTCCGAAGCCAATCTATCATTCGTTGGGATATCCATCACCCCTGAA

TTCATCGATGAGGCCCTCAGAGGCTTTTGCCAAAAGAGCCGTTGGCTACAAAGAGGTTTA

CGTTCAGGTTGATCGAGATCCTAAAGTTGYCTGGGAAGGAGCGCTCAATGGTTACCTCGG

CAAATATTCTGGCGTCAAAAATGTTCATTTTGCGCCGAACAACGCCGCGATTGGAAAGCC

CCCACGCCATATCGACATTTTGCTTCGGAAAAAATTTAGCAGCGAACTTCTTGCGACGCT

CGAAGGTGTGGCAATTTCAAACGGTGCAACTGGTTGGCTTGCGCTCGCATGCGTAAACCT

TCTCTTGAAGGCAAGACAAGATATTGATGTCTTCTTGTTTGGGGCGGGAAAAGTCGCCGA

GGCTGTGATTCTTGCCCTCAATCATGGTGCGGCTGCAAAAATCAAAACCATGGCAGTGCT

CAGCCAAGCTCCAATTGAAGGTGGGGTTCCAGCTCGATGTCGTCATTGATCAGAAGGTTA

TACCAAAAGCCAAACTCGTCATAACAGCAACAAATTCCGAGGAGCTCGGTACTTGAGGCC

GACGAAATTGCGCCAAATGCAGTAACCATATCCCTCGGAAAAGATGAATTGCCCGTCGCC

TACTTCGATCGCCTTTTGAACGCAGAGGGTCTGATTATCGGCGACGATCTGGATGCGATC

GAATCATGCAATGCCAATTCCCTGGTGCTTTACTACTCGAAATGTGATTTGAAGCTGACA

GAACATAGAAGGGATCATTGGATAAAGAACTACGCCAATGTCCTTGCTGATCCAGCTCTC

ATGGAGGAACTTAAGACATGGGAGGGGCCAGCCAACTTTTCATCGGTTGGCCTTGCCAGC

CTAGACTTGGCGATGGCCGGCCGACTCTACGAAACTCTTACTGCGAAACTCTCCCACCCC

CAGTAGACAAGACCCTCTTAGGATTGCGCTCTTCAGAAGGTGCGCCTCCCCAGTCACAAC

CGGAAAGTCACTCAACCTGGCTCAAGTACCCTACTTAGTTTGTCTCTCTGTCGTGTTTAG

TATCAGCAACAATAACAAATATTGTAAATAACATATATTTATATGGTAATTATATGGCAT

GTAAGCGTGACAACGATTATGTTTTCCATAGTTTGATAAACTCAACACCCGAAAAAGCCG

GTGACCAAAGGGTTGGGACCTCTTTGCACGAACTCCTAtggCTGCCAGACTATCTACAGA

GCTTTTCGGAAGCTGACGCAGCATCTGATCGCCACACAATCCTCGCGGACGTGGATGGCG

TGGCTACACGCAAGCAGTTTGACTGAGAGTTTTTGAGTGACTATTTTAAAAGGACAATAT

TTCGTCCCAAGCGATAAAACTTGCTCACATGACTAAGtcgttgctttaattcaacTGCGT

GAAAAGATTTAAAGGGCAGAACTGTTTGTGTTTCGTATCCAGTTCACCTGCAATTATTGG

CTACGCATGAAGCTCATTATCGTCGATTCTTTACCAAAACACTGATGCCATTCCAAATGA

GTTGGGCCAAAGATGTACTCATGTCGGACAGGTGCGATTTGGACTTCGTGATCCTCAAGT

CTATCGCAGTGCTGCTTTATTACAGGCTATGCTTCCACCCTCCATTATTTGGTCAACTGG

ACCTGGCTTTTGACAAGAATAGGCCAGCGTTCATCTATCTATCTTTCAAAAACATGAAGT

TACTGCTGCGTAAAGGATGTAACATCGGATTTGGTGTGGAGACGGTGCTCACTACGACTA

TACTGCCTTACTGTAGTGATCTGTCCCGCCAAGCGACGAGGGGAGCGGCCTGCCTCTCGT

GAGCGTCAATCCATATGGTTGCATGTTCGTTTGTTTCAAAGGCATTCCCAAGAAGGGTTT

CCAGCTTGACAACTTTCTCATCGGTAGGTGTAAACTCGCCGGTTAGTGAGTCACTGCCCA

TTTTGGGAACTACCAGGGGGGTGCCAACGACCTCCTCCGTCGACATCTCAGACCAATTTC

AATTGCCCAACTGCGCCGTTCTTTCTATGTTGAAGTCATGGGCGACTATGAAATCTCCAT

CTTTGGTCAGTACTACCTCGATCTCAACTATATTGAAAAGCCTGAGAGATTTGCAAACTG

CCCTACACTGTTTTCCGGCAAGTGTGGCCCATGGAATCCACGGTGAACTATCAATTGGCG

GTCGCTTGAAGTCTTTCGAGCGATAAGCCAATTGGCGAGGCAGTGTTTCGCGATCGCATC

TTTAAGAGGAAAGTCCGATGCAAATCTCCATGTAGACCCGGTCGCTTCGGCCAAGACGGC

CTCTAAATCAACTTGAGCAGAAGTTGTGTTAAGGGAATCTGTTTTGACTGCCATTCTGTA

AGAAATGTCAGCAAAGGAAAAGCTAGTTGGAGAAGGAGAGCTTGCTCAATAGTGTGTCTA

TTCTCAGATGAACTATACTAGCGCAATGAGTTGGTGTTGTTCGCTGATGGGAAAAAGGAA

ACGCATCCCCCTCTTCTACAAAATTCTATGGGCCCGGAGGGACTCTGTATGAAAGTGCAC

AGCATGTGGACGCATTACCATGCAATTAGCGAGCTTAGCCAATGCCGTGTCTGTCAGAGT

GTGCAGCATGTGGGCCAAGCGCAATTACAATAAAAAAATAATATAAAAACTTTGCCAAGG

TCCGAACCGTCTCAAATGCTTTGAGGGCCATCCAAGCCAAGCGCACCTAACGCCTAGCAC

ACCATTGTTGCGCCGCTCGAGCATTAATGGTCCGGGACCTGGTGTCATCTGGAAACCTAT

GAAGCATGGACACGGCAAAATCAGCGCCGTGGCGGTGTCAGACACGAGGACACGTCGGGG

ACACGTACA

>sa6856_1

CCTTGAAGTATCAATTTGATCTAGAAGCCAATCCAAACTCATTGAAAGGCCTGTCATCAA

CCTGTCAACACGGTTCAATGTTTGCTCGAGTGGCGCAGCAATGGTGTGCTAGGCGTTAGG

TGCGCCTGGCTTGGATAGCCTTCAAAGCATttgaggcagttcggacctcggcagagattt

tatattatttttttgttgtaatcacgcttggcccacatgctgcacactccgatagacacg

gcattggctaaacccgctaattgcgtggtgatgcgtccacgtgctgcgcactttcaggcg

gagtccccccgattccataGaAtAAGAGGGGGACGCGTTTTCTTTTTCCCATCAGCAAAC

AACACCAACTCACTGTGCAAGTATAGTTCATCTGAGAACAGACACACTGCTGAGCAAGCT

CTCCTTCTCCAACTAGCTTTTTCTTTGCTGACATTTCCTACAGAATGGCAGCAAAACAGA

TTCCGTTAACACAACTGCTGCTCAAGTTGATTTAGAGGCCGCCTTGGCCGAAGTGACCGG

GTCTACATGGAGATTTGCATCAGACTTTCCTCTTAAAGATGCGATCGCGAAACACTGCCT

CGCCAATTGGCTTATCGCTCGAAAGACTTCAAGCAACCACCAATTGATAGTTCACCGTGA

ATTTCATGGGCCACACCTGCCGGAAAACAGCGTAGGGGCAGTCTGCAAATCTCTCAGACT

TTTTAATATGGTCGAGATCAACGTAGTACCGACCAAAGATAGAGATTTCATAGTCGTCCA

CGACTTCAACACAGAAAGAACGACGCAGTTAGGCAATTGAAATTGGTCTGATATGTCGAC

GAAAGAGGCTGTTGGCACCCCCCTGGTAGTTCCCATAATGGGCGGTGACTCCCTAACTAG

CGAGTTCACACCCACCGATGAGAAAGTTGTCAAGCAAGAAACCCTTCTCAGGAATGCCTT

TGAAACAAACAAACAGGCAACCATACGGATTGACGCTCACGAGAGGCAGGCCGCTCCCCT

CGTCGCTTAGCTGAGCCATAGGCCGGCCTATCACGACAAGACAATCGTTATCTTTCATAC

ATATAGATACAACAATGGACACGAGTTCGCCTTGGCGGTCGATCAAGCCGGCGCTACAGA

AAATTGGCACGAAACTGTAGCGCTAATGCCAAACATTCTGCCGAATGAACTCTCTAGGCT

CGCGAAGCAATATCCACCAAATCGCACGTCGCATTTTTACGACCTCGTTGAGGCCGGCAA

GAATTGGTTCGCGTCTGTCTTCTCCCAGAATATGATGGTGGTCGTCACTTTCCTCAAGAT

TGCGGGCACTGGCGAAGGACAGACTAACAAAGTCGTTGATCCAAATGGAAATGAGGTTCT

GCATCCTAAAGAGGAGGAGGAATATCTCCACGATTCAGCTTCAATCAAAATCGTGAAATG

AATCTGCGCCACCCATCCCGGAATCGAGATTGGAGGCGTCACACGAACTTATGcttataa

gagtcgaggacatcgctacaaaatcGATCTTTGGACGGGTCAACCGAAGCGGTGGCCGAC

CCACGCGACGAAGTATATCTGGGAAGATTTCGCCAAGCCTGGAAATGCGCTCAGATGGGG

TCAAACGATTATCTCTGATCGCATTGGCGACGACCTTGCAGCTTGTGTGCTTCAAAGCCG

TTGAGAAAATTTTGTCAGCAGTCATGAATATCCCGCTGTTCATGTCGATTAGATAGGCGA

TCTGTGTCTTAGTCTAAGCTTCCGTTTAACGCATCTTTGCTACTTTAATGTATTTTTTTA

ATGATATTGTTGGAGTTTTCAGCACATCTTAATCGCAACTAGATATTTTTTTTTGAATCG

GATTCGACTTCAAGTAGATGTACTTCTTTGTCTCTCGATAAATCTAATGACCCTGCGAGC

CGGCCTCTCGCCTTAAACTAATTTCTGAGAGTTAGGTCGGCGCCTCTCGTGGTTTCCATG

TTCTATGGAAACGGCCTCTCGGGACTTGCCTTCCCGCAACTCATCGAGCTTGCCTGGCCC

GCTCGGGTGGTGGCGCAAGCCATCGTTAAATCGTTCGTAATCCTCTACTCTCGCATAAAT

GAGAGGGGAATCCAAAGATGATCACTTTTCAGCTAATTGGAATCTGTAATCCCAAAAGAG

GCAAGCACTATGTGGGGTCCACAACTTATAGAATTTTGGCCTAACCATTCTGCATAATGG

ATCATGACATGGGCCATTTATGGCCGCAAAAACTGGCACAAATTCCAACCTGTTCAGCAC

AATGAACAAGTTCTTCCAATTGGAATAAAGGAGGTTAAAATTATTCGAGTAGACATGACG

TGCCAGCACGCCACTAGCCTCGCATTGATGCCACCCTTTCTTGCGCACACTTATATAATG

CTGAGCTAATAATAGAGATAAAAATAGTCAGCTTGTAAACTGGACCTTTACTACTGCCCC

TAATCTCAAGATCAAAGCGACATATACCTCCTCTCTTTCAAGTCTTTTTGCAACTTGTTC

CAAAGCAAGCGTTCATGAATCTGCTAGGCAACCTTCACGAAAATGGCTGATCAACTATGT

GAGGGGCGTTGGTCCGAAGCCGATCTATCATTCRTTGGGATATCCATCACCCCTGAATTC

ATCGATGAGGCCCTCAGAGGCTTTTGGGAGTCTTTTGCCAAAAGAGCCGTTGGCTACAAA

GAGGTTTACGTTCAGGTTGATCGAGATCCTAAAGTTGCCTGGGAAGGAGCGCTCAATGGT

TACCTCGGCAAATATTCTGGCGTAAAAAATGTTCATTTTGCGCCGAACAACGCCACGATT

GGAAAGCCCCCACGCCATATCGACATTTTGCTTCGGAAAAAATTTAGCGGCGAACGTCTT

GCGACGCTCGAAGGTGTGGCAATTTCAAACGGTGCAACTGGTTGGCTTGCGCTCGCATGT

GTAAACCTTCTCTTGAAGGCAAGACAAGATATTGATGTCTTCTTGTTTGGGGCAGGAAAA

GTTGCCGAGGCTGTGATTCTTGCCCTCAATCATGGTGCGGCTGCAAAAATCAAAACCATG

GCAGTGCTCAGCCAAGCTCCAATTGAAGGTGGGGTTCCAACTCGATGCCGTCATTGATCG

GAAGGTTATACCAAAAGCCAAACTCGTCATAACAGCAACAAATTCCGAGGAGCTCGGTAC

TCGAGGCCGACGAAATTGCGCCAAATGCAGTAACCATATCCCTCGGAAAAGATGAATTGC

CCGCCGCCTACTTCGATCGCCTTTTGAACGCAGAGGGTCTGATTATTGGCGACGATCTGG

ATGCGATCGAATCGCACAATGTCAATTCCCTGGTGCTTTACTACTCGAAACGTGATTTGA

AGCTGACAGAACATGGAAGGGATCATTGGATAAAGAACTACGCCAATGTCCTTGCTGATC

CAGCTCTCATGGAGGAACTTAAGACATGAGAGGGGCCAGCCAACTTTTCATCGGTTGGCC

TTGCCAGCCTAGACTTGGCGATGGCCGGCCGACTCTACGAAACTCTTACTGCGAAACTCT

CCCACCCCCAGTAGACAAAACCCTCTTAGGATTGCGCTCTTCAGAAGGTGCGCCTCCCCA

GTCACAACCGGAAAGTCACTCAACTTGGCTCAAGTACCCTACTTAGTTTGTCTCTCTGTC

GTGTTTAGTATCAGCAACAATAACAAATATTGTAAAtaacatatatttatatggtaatta

tatggcatgtaagcgtgaCAACGATTATGTTTTCCATAGTTTGATAAACTCAACACCCGA

AAAATCCGGTGACCAAAGGGTTGGGACCTCTTTGCACGAACTCCTATGGCTGCCAGACTA

TCTACAGAGCTTTTTCGGAAGCTGACGCAGCATCTGATCGCCACACAATCCTCGCGGACG

TGGATGGCGTGGCTACATGCAAGCAGTTTGACTGAGAGTTTTCGAGTGACTGTTTTAAAA

GGACAATATTTCGTCCCAAGCGATAAAACTCGCTCACATGACTAAGTCGTTGCTTTAATT

CAACTGCGTGAAAAGATTTAAAGGGCAGAACTGTTTGTGTTTCGTATCCAGTTCACCGGC

AATTATTGGCTACGCATAAAGCTCATTATCGTCGATTCTTTACCCAAACACTGATGCCAT

TCCAAATGCGTTGGGCCAAAGATGTACTCATGTCGGACAGGCGCAATTTGGACTTCGTGA

TCCTCAAGTCTATCGCAGTGCTGCTTTATTACAGGCTATGCTTCCACCCTCCATTATTTG

GTCAACTGGACCTAGCTTTTGACAAGAATAGGCCAGCGTTCATCTATCTATCTTTCAAAA

ACATGCAGTTGCTGCTGCGTAAAGGATGTAACATCGGATTTGGTGTGGAGACGGTGCTCG

CTACGACTATACTGCCTTACTGTAGTGATCTGTCCTGCCAAGCGATGAGGGAAGCGGCCT

GCCTCTCGTGAGCGTCAATCCATACGGTTGCATGTTCGTTTGTTTCAAAAGCATTCCCAA

GAAGGGTTTCCAGCTTGACAACTTTCTCATCGGTGGGTGTGAACTCGCCGGTTAGTGAGT

CACCGCCCATTTTGGGAACTACCAGGGGGGTGCCAACGACCTCCTCCGTCGACATCTCAG

ACCAATTTCAATTGCCCAACTGCACCGTTCTTTCTATGTTGAAGTCATGGGCGACTATGA

AATCTCCATCTTTGGTCGGTACTACCTCGATCTCGACCATATTGAAAAGCCTGAAAGATT

TGCAAACTGCCCCTACACTGTTTTCCGGCAAGTGTGGCCCATGGAATCCACGGTGAACTA

TCAATTGGCGGTCGCTTGAAGTCTTTCGAGCGATAAGCCAATTGGCGAGGCAGTGTTTCG

CGATCGCATCTTTAAGAGGAAAGTCCGATGCAAATCTCCATGTAGACCCAGTCGTTTCGG

CCAAGGCGGCCTCTAAATCAACTTGAGCAGCAGTTGTGTTAACGGAATCTGTTTTGACTG

CCATTCTGTAAGAAATGTCAGCAAAGGAAAAGCTAGTTAGAGAAGGAGAGCTTGCTCAAT

AGTGTGTCTATTCTCAAATGAACTATACTAGCGCAATGAGTTGGTGTTGTTCGCTGATGG

GAAAAAGGAAACGCATCCCCCTCTTCTACAAAATTCTGTGGGCCCGGAGGGACTCCGTAT

GAAAGTGCACAGCATGTGGACGCATCACCATGCAATTAGCAAGCTTAGCCAATGTCGTGT

CTGTCAGAGTGTGCAGCATGTGAGCCAAGCGCAATTACAATAAAAAAATAATATAAAAAC

TCTGCCGAGGTCCGAACCGTCTCAAATGCTTTGAGGGCCATCCAAGCCAAGCGCACCTAA

CGCCTAGCACACCATTGTTGCGCCGCTCGAGCATTAATGGTTCAGGACCTGGTGTCATCT

GGAAACCTATGAAGCATAGACACGGCAAAATCAGCGCCGTGGCGGTGTCAGACACGAGGA

CACGTCggggacacgtacag

>sa7011_Un

TTTGATCTAGAAGCCAATCCAAACTCATTGAAAGGCCTGTCATCAACCGGTCAACACGGT

TCAATGTTTGCTCGCGTGGCGCAGCAATGGTGCGCTAGGCGTTAGGTGCGCCTGGCTTGG

ATGGCCCTCAAAGCATTTGAGGCAGTTCGGACCTCGGCAGAGATTTTATATTATTTTTTT

GTTGTAATCGCGCTTGGCCCACATGCTGCACACTCCGATAGMCACGGCATTGGCTAAACC

CGCTAATTGCGTGGTGATGCGTCCACGTGCTGCGCACTTTCATGCGGAGTCCCCCCGAGT

CCATAGAATAAGAGGGGGACGCATTTCCTTTTTCCCATCAGCAAACAACACCAACTCACT

GTGCTAGTATAGTTCATCTGAGAACAGACACACTGCTGAGCAAGCTCTCCTTCTCTAACT

AGCTTTTTCTTTGCTGACATTTCCTACAGAATGGCAGCAAAACAGATTCCGTTAACACAA

CTACTGCTCAGGTTGATTTAGAGGCCGCCTTGGCCGAAGCGATCGGGTCTACATGGAGAT

TTGCATCAGACTTTCCTCTTAAAAATGCAATCGCGAAACACTGCCTCGCCAATTGGCTTA

TCGCTCGAAAGACTTCAAGCAACCACCAATTGATAGTTCACCGTGAATTCCATGGGCCAC

ACCTGCCGGAAAACAGCGTAGGGGCAGTCTGCAAATCTCTCAGACTTTTTAATATGGTCG

AGATCAACGTAGTACCGACCAAAGATAAAGATTTCATAGTCRTCCACGACTTCAACACAG

AAAGAATGACGCAGTTAGGCAATTGAAATTGGTTTGATATGTCGACGAAAGAGGCTGTTG

GCACCCCCCTGGTAGTTCCCATAATGGGCGGTGACTCCCTAACCAGCGAGTTCACACCCA

CCGATGAGAAAGTTGTCAAGCAAGAAACCCTTCTCAAGAATGCCTTTGAAACAAACAAAC

AGGCAACCATACGGATTGATGCTCACGAGAGGCAGGCCGCTCCCCTCGTCGCTTGGCTGA

GCCATAGGCCGGCCTATCACGACAAGACAATCGTTATCTTTCATACATATGGATACAACA

ATGGACACGAGTTCGCCTTGGCGGTCGATCAAGCCGGCGCTACAAAAAATTGGCGCGAAA

CTGTAGCGCTAATGCCAAACATTCTGCCGAATGAACTCTCTAGGCTCGCGAAGCAATATC

CACCAAATTGCACGTCGCATTTTTACGACCTCGTTGAGGCCAGCAAGAATTGGTTCGCAT

CTGTCTTCTCCCAGAATATGATGGTGGTCGTCACTTTCCTCAAGATTGCGGGCACTGGCG

AAGGACAGACTAACAAAGTCGTTGATCCAAATGGAAATGAGGTTTTGCATCCTAAAGAGA

AGGAGGAATATCTCCGCGATTCAGCTTCAATCGAAATCGCGAAATGAATCTGCGCCACCC

ATCCCGGAATCGAGATTGGAGGCGTCACACGAACTTATGCTTATAAGAGTCGAGGACATC

GCTACAAAATCAATCTTTGGACGGGTCAACCGAAGCGGTGGCCGACCCACGCGACGAAGT

ACATCTAGGAAGATTTCGCCAAGCCTGGAAATGCGCTCAGATGGGGTCAAACGATTATCT

CTGATCGCATTGGTGACGACCTTGCAACTTGTGTGCTTCAAAGCCGTGGAGAAAATTTTG

TCAGCAGTCATGAATATCCTGCTGTTCATGTCGATTAGATGGGCGATCTGTGTCTTAGTC

TAAGCTTCCGCTTAACACATCTTTGCTACTTTAATGTATTTTTTTAATGATATTGTTGGA

GTTTTGAGCACATCTTAATCGCAAGTAGATATTTTTTTTGAATCGGATTCGACTTCAAGC

AGATGTACTTCTTTGTCTCTCGATAAATCTAATGACCCTGCGAGCCGGCCTCTCACCTTA

AACTAATTTCTGAGAGTTAGGTCGGCGCCTCTCGTGGTTTCCATGTTCTATGGAAACGGC

CGCTCGGGACTTGCCTTCCCGCAACTCATCGAGCTTGCCTGGCCCGCTCGGGTGGTGGCA

CAAGCCATCGTTAAATCGTTCGTAATCCTCTACTCTCGCATAAATGAGAGGGGAATCCAA

AGATGATCACTTTTCAGCTAATTGGAATCTGCAATCCCAAAAGAGGCAAGCACTATGTGG

GGTCCACAACTTATAGAATTTTGGCCTAACCATTCTGCATAATGGATCATGAGATGGGCC

ATTTATGGCCGCAAAAACTGGCACAAATTCCAACCTATTCAGCACAATGAACAAGTTCTT

CCAATTGGTATAAAGGAGGTTAAAATTATTCGAGTAGACATGACGTGCTGGCACGCCACT

AGCCTCGCATTGATGCCACTCTTTCTTGCGCACACTTATATAATGCTGAGCTAATAATAG

AGATAAAAATAGTCAGCTTGTAAACTGGACCTTTATTACTGCCCCTAATCTCAAGATCAA

AGCGACATATACCTCTTCTCTTTCAAGTCTTTTTGCAACTTGTTCCAAAGCAAGCGTTTG

TGAATCTGCCAGGCAGCCTTCACGAAAATGGCTGATCAACTGTGTGAGGGGCGTTGGTCC

AAAGCCAATCTATCATTCGTTGGGATATCCATCACCCCTGAATTCATTGATGAGGCCCTC

AAAGGCTTTTGGGAGTCTTTTGCCAAAAGAGCCGTTGGCTACAAAGAGGTTTACGTTCAG

GTTGATCGAGATCCTAAAGTTGCCTGGGAAGGAGCGCTCAATGGTTACCTCGGCAAATAT

TCTGGTGTAAAAAATGTTCATTTTGCGCCGAACAACGCCACGATTGGAAAGCCCCCACGC

CATATCGACATTTTGCTTCGGAAAAAATTTAGCGGCGAACTTCTTGCGACGCTCGAAGGT

GTGGCAATTTCAAACGGTGCAACTGGTTGGCTTGCGCTCGCATGTGTAAACCTTCTCTTG

AAGGCAAGACAAGATATTGATGTCTTCTTGTTTGGGGCAGGAAAAGTCGCCGAGGCTGTG

ATTCTTGCCCTCAATCATGGTGCGGCTGCAAAAATCAAAACCATGGCAGTGCTCAGCCAA

GCTCCAATTGAAGGTGGGGTTCTAGCTCGATGCCGTCATTGATTGGAAGGTTATACCAAA

AGCCAAACTCGTCATAACAGCAACAAATTCCGAGGAGCTCGGTACTCGAGGCCGACGAAA

TTGCGCCAAATGCAGTAACCATATCCCTCGGAAAAGATGAATTGCCCGCCGCCTACTTCG

ATCGCCTTTTGAACGCAGAGGGTCTGATTATTGGCGACGATCTGGATGCGACCGAATCGC

GCAATGTCAATTCCCTGGTGCTTTACTACTCGAAACGTGATTTGAAGCTGACAGAACATG

GAAGGGATCATTAGATAAAGAACTACGCCAATGTCCTTGCTGATCCAGCTCTCATGGAGG

AACTTAAGACATGAGAGGGGCCAGCCAACTTTTCATCGGTTGGCCTTGCCAGCCTAGACT

TGGCGATGGCCGGCCGACTCTACGAAACTCTTACTGCGAAMCTCTCCCACCCCCAGTAGA

CAAAACCCTCTTAGGATTGCGCTCTTCAGAAGGTGCGCCTCCCCAGTCACAACCGGAAAG

TCACTCAACTTGGCTCAAGTACCCTACTTAGTTTGTCTCTCTGTCGTGTTTAGTATCAGC

AACAATAAYAAATATTGTAAATAACATATATTTATATAGTAATTATATGGCATGTAAGCG

TGACAACGATTATGTTTTCCATAGTTTGATAAACTTAACACCCGAAAAAAGCCGGTGACC

AAAGGGTTGGGACCTCTTTGCACGAACTCCTATGGCTGCCAGACTATCTACAGAGCTTTT

TCGGAAGCTGACGCAGCATCTGATCGCCACACAATCCTCGCGGACGTGGATGGCGTGGCT

ACATGCAAGCAGTTTGATTGAGAGTTTTCGAGTGACTATTTTAAAAGGACAATATTTCGT

CCCAAGCGATAAAACTCGCTCACATGACTAAGTCATTGCTTTAATTCAACTGCGTGAAAA

GATTTAACGGGCAGAACTGTTTGTGTTTCGTATCCAGTTCACCGGCAATTATTGGCTACG

CATGAAGCTCATTATCGTCGATTCTTTACCCAAACACTGATGCCATTCCAAATGAGTTGG

GCCAAAGATGTACTCATGTCGGACAGGCGCGATTTGGACTTCGTGATCCTCAAGTCTATC

GCAGTGCTGCTTTATTACAAGCTATGCTTCCACCCTCCATTATTTGGTCAACTGGACCTA

GCTTTTGACAAGAATAGGCCAGCGTTCATCTATCTATCTTTCAAAAACATGCAGTTGCTG

CTGTGTAAAGGATGTAACATCGGATTTGGTGTGGAGACGGTGCTCGCTACGACTATACTG

CCTTACTGTAGTGATCTGTCCCGCCAAGCGATGAGGGGAGCGGCCTGCCTCTCATGAGCG

TCAATCCATACGGTTGCATGTTCGTTTGTTTCAAAGGCATTCCCAAGAAGGGTTTCCAGC

TTGACAACTTTCTCATCGGTGGGTGTGAACTCGCCGGTTAGTGAGTCACCGCCCATTTTG

GGAACTACCAGGGGGGTGCCAACGACCTCCTCCGTCGACATCTCAGACCAATTTCAATTG

CCCAACTGCACCGTTCTTTCTATGTTGAAGTCATGGGTGACTATGAAATCTCCATCTTTG

GTCGGTACTATCTCGATCTCGACCATATTGAAAAGCCTGAAAGATTTGCAAACTGCCCCT

ACACTGTTTTCCGGCAAGTGTGGCCCATGGAATCCACGGTGAACTATCAATTGGCGGTCG

CTTGAAGTCTTTCGAGCGATAAGCCAATTGGCGAGGCAGTGTTTCGCGATCGCATCTTTA

AGAGGAAAGTCCGATGCAAATCTCCATGTAGACCCAGTCGTTTCGGCCAAGGCGGCCTCT

AAATCAACTTGAGCAGCAGTTGTGTTAACGGAATCTGTTTTGACTGCCATTCTGTAAGAA

ATGTCAGCAAAGGAAAAGCTAGTTAGAGAAGGAGAGCTTGCTCAATAGTGTGTCTATTCT

CAAATGAACTATACTAGCGCAATGAGTTGGTGTTGTTCGCTGATGGGAAAAAGGAAATGC

ATCCCCCTCTTCTACAAAATTCTGTGGGCCCGGAGGGACTCCGTATGAAAGTGCACAGCA

TGTGGACGCATCACCATGCAATTAGCAAGCTTAGCCAATGTCGTGTCTGTCAGAGTGTGC

AGCATGTGAGCCAAGCGCAATTACAATAAAAAAATAATATAAAAACTCTGCCGAGGTCCG

AACCGTCTCAAATGCTTTGAAGGCCATCCAAGCCAAGCGCACCTAACGCCTAGCACACCA

TTGTTGCGCCGCTCGAGCATTAATGGTTCGGGACCTGGTGTCATCTGGAAACCTATGAAG

CATGGACACGGCAAAATCAGCGCCGTGGCGGTGTCAGACACGAGGGACACGTCGGGGACA

CG

>sa7009_Un

ACAAGCCTCTCCAAATTTCCCTCACCTYGAAGTATCAATTTGATCTAGAAGCCMATCCAA

ACTCATTGAAAGGCCTGTCATCMACCGGTCAACACGGTCAATGTTTGCTCGCGTGGCGCA

GCAATGGTGTGCTAGGCGTTAGGTGCGCCTGGCTTGGATGGCCCTCAAAGCATTTGAGGC

AGTTCGGACCTCGGCAGAGATTTTATATTATTTTTTTGTTGTAATCGCACTTGGCCCACA

TGCTGCACACTCCGATAGACACGGCATTGGCTAAACCCGCTAATTGCGTGGTGATGCGTC

CACGTGCTGCGCACTTTCATGCGGAGTCCCCCCGAGTCCATAGAATAAGAGGGGGACGCG

TTTCCTTTTTCCCATCAGCAAACAACACCAACTCACTGTGCTAGTATAGTTCATCTGAGA

ACAGACACACTGCTGAGCAAGCTCTCCTTCTCCAACTAGCTTTTTCTTTGCTGACATTTC

CTATAGAATGGCAGCAAAACAGATTCCGTTAACACAACTACTGCTCAGGTTGATTTAGAG

GCCGCCTTGGCCGAAGCGATCAAGTCTACATGGAGATTTGCATCAGACTTTCCTCTTAAA

AATGCAATCGCGAAACACTGCCTCGCCAATTGGCTTGTCGCTCGAAAGACTTCAAGCAAC

CACCAATTGATAGTTCACCGTGAATTCCATGGGCCACACCTGCCGGAAAACAGCGTAGGG

GCAGTCTGCAAATCTCTCAGACTTTTTAATATGGTCGAGATCAACGTAGTACCGACCAAA

GATAAAGATTTCATAGTCGTCCACGACTTCAACACAGAAAGAACGACGCAGTTAGGCAAT

TGAAATTGGTTTGATATGTCGACGAAAGAGGCTGTTGGCACCCCCTTGGTAGTTCCCATA

ATGGGCGGTGACTCCCTAACCAGCGAGTTCACACCCACCGATGAGAAAGTTGTCAAGCAA

GAAACCCTTCTCAAGAATGCCTTTGAAACAAACAAACAGGCAACCATACGGATTGACGCT

CACGAGAGGCAGGCCGCTCCCCTCGTCGCTTGGCTGAGCCATAGGCCGGCCTATCACGAC

AAGACAATCGTTATCTTTCATACATATGGATACAAAAAATGGACACGAGTTCGCCTTGGC

GGTCAATCAAGCCGGCGCTACAAAAAATTGGCGCGAAACTGTAGCGCTAATGCCAAACAT

TCTGCCGAATGAACTCTCTAGGCTCGCGAAGCAATATCCACCAAATTGCACGTCGCATTT

TTACGACCTCGTTGAGGCCAGCAAGAATTGGTTCGCATCTGTCTTCTCCCAGAATATGAT

GGTGGTCGTCACTTTCCTCAAGATTGCGGGCACTGGCGAAGGACAGACTAACAAAGTCGT

TGATCCAAATGGAAATGAGGTTCTGCATCCTAAAGGGAAGGAGGAATATCTCCGCGATTC

AGCTTCAATCGAAATCGCGAAATGAATCTGCGCCACCCATCCCGGAATCGAGATTGGAGG

CGTCACACGAACTTATGCTTATAAGAGTCGAGGACATCGCTACAAAATCAATCTTTGGAC

GGGTCAACCGAAGCGGTGGCCGACCCACGCGACGAAGTACATCTGGGAAGATTTCGCCAA

GCCTGGAAATGCGCTCAGATGGGGTCAAACGATTATCTCTGATCGCATTGGTGACGACCT

TGCAGCTTGTGTGCTTCAAAGCCGTGGAGAAAATTTTGTCAGCAGTCATGAATATCCTGC

TGTTCATGTCGATTAGATGGGCGATCTGTGTCTTAGTCTAAGCTTCCGCTTAACGCATCT

TTGCTACTTTAATGTATTTTTTTAATGATATTGTTGGAGTTTTGAGCACATCTTAATCGC

AAGTAGATATTTTTTTTGAATCGGATTCGACTTCAAGCAGATGTACTTCTTTGTCTCTCG

ATAAATCTAATGACCCTGCGAGCCGGCCTCTCGCCTTAAACTAATTTCTGAGAGTTAGGT

CGGCGCCTCTCGTGGTTTCCATGTTCTATGGAAACGGCCGCTCGGGACTTGCCTTCCCGC

AACTCATCGAGCTTGCCTGGCCCGCTCGGGTGGTGGCACAAGCCATCGTTAAATCGTTCG

TAATCCTCTACTCTCGCATAAATGAGARGGGGAATCCAAAGATGATCACTTTTCAGCTAA

TTGGAATCTGCAATCCCAAAAGAGGCAAGCACTATGTGGGGTCCACAACTTATAGAATTT

TGGCCTAACCATTCTGCATAATGGATCATGACATGGGCCATTTATGGCCACAAAAACTGG

CACAAATTCCAACCTATTCAGCACAATGAACAAGTTCTTCCAATTGGAATAAAGGAGGTT

AAAATTATTCGAGTAGACATGACGTGCTGGCACGCCACTAGCCTCGCATTGATGCCACTC

TTTCTTGCGCACACTTATATAATGCTGAGCTAATAATAGAGATAAAAATAGTCAGCTTGT

AAACTGGACCTTTATTACTGCCCCTAATCTCAAGATCAAAGCGACATATACCTCTTCTCT

TTCAAGTCTTTTTGCAACTTGTTCCAAAGCAAGCGTTTGTGAATCTGCCAGGCAGCCTTC

ACGAAAATGGCTGATCAACTGTGTGAGGGGCGTTGGTCCGAAGCCAATCTATCATTCGTT

GGGATATCCATCACCCCTGAATTCATTGATGAGGCCCTCAGAGGTTTTTGGGAGTCTTTT

GCCAAAAGAGCCGTTGGCTACAAAGAGGTTTACGTTCAGGTTGATCGAGATCCTAAAGTT

GCCTGGGAAGGAGCGCTCAATGGTTACCTCGACAAATATTCTGGCGTAAAAAATGTTCAT

TTTGCGCCGAACAACGCCACGATTGGAAAGCCCCCACGCCATATCGACATTTTGCTTCGG

AAAAAATTTAGCGGCGAACTTCTTGCAACACTCGAAGGTGTGGCAATTTCAAACGGTGCA

ACTGGTTGGCTTGCGCTCGCATGTGTAAACCTTCTCTTGAAGGCAAGACAAGATATTGAT

GTCTTCTTGTTTGGGGCAGGAAAAGTCACCGAGGCTGTGATTCTTGCCCTCAATCATGGT

GCGGCTGCAAAAATCAAAACCATGGCAGTGCTCAGCCAAGCTCCAATTGAAGGTGGGGTT

CCAGCTCGATGCCATCATTGATCGGAAGGTTATACCAAAAGCCAAACTCGTCATAATAGC

AACAAATTCTGAGGAGCTCGGTACTCGAGGCCGACGAAATTGCGCCAAATGCAGTAACCA

TATCCCTCGGAAAAGATGAATTGCCCGCCGCCTACTTCAATCGCCTTTTGAACGCAGAGG

GTCTGATTATTGGCGACGATCTGGATGCGATCGAATCGCGCAATGTCAATTCCCTGGTGC

TTTACTACTCGAAACGTGATTTGAAGCTGACAGAACATGGAAGGGATCATTGGATAAAGA

ACTACGCCAATGTCCTTGCTGATCCAGCTCTCATGGAGGAACTTAAGACATGAGAGGGGC

CAGCCAACTTTTCATCGGTTGGCCTTGCCAGCCTAGACTTGGCGATGGCCGACCGACTCT

ACGAAACCCTTACTGCGAAACTCTCCCACCCCCAGTAGACAAAACCCTCTTAGGATTGCG

CTCTTCAGAAGGTGCGCCTCCCCAGTCACAACCGGAAAGTCACTCAACTTGGCTCAAGTA

CCCTACTTAGTTTGTCTCTCTATTGTGTTTAGTATCAGCAACAATAACAAATATTGTAAA

TAACATATATTTATATGGTAATTATATGGCATGTAAGCGTGACAACGATTATGTTTTCCA

TAGTTTGATAAACTCAACACCCGAAAAAGCCGGTGACCAAAGGGTTGGGACCTCTTTGCA

CGAACTCCTATGGCTGCCAAACTATCTACAGAGCTTTGCGCTGACGCAGCATCTGATCGC

CACACAATCCTCGCGGACGTGGATGGCATGGCTACATGCAAGCAGTTTGACTAAGAGTTT

TCGAGTGACTATTTTAAAAGGACAATATTTCGTCCCAAGCGATAAAACTCGCTCACATGA

CTAAGTCGTTGCTTTAATTCAACTGCGTGAAAAGATTTAAAGGGCAGAACTGTTTGTGTT

TCGTATCCAGTTCACCGGCAATTATTGGCTACGCATGAAGCTCATTATCGTCGATTCTTT

ACCCAAACACTGATGCCATTCCAAATGAGTTGGGCCAAAGATGTACTCATGTCGGACAGG

CGCGATTTGGACTTCGTGATCCTCAAGTCTATCGCAGTGCTGCTTTATTACAGGCTATGC

TTCCACCCTCCATTATTTGGTCAACTGGACCTAGCTTTTGACAAGAATAGGCCAGCGTTC

ATCTATCTATCTTTCAAAAACATGCAGTTGCTGCTGCGTAAAGGATGTAACATCGGATTT

GGTGTGGAGACGGTGCTCGCTACGACTATACTGCCTTACTGTAGTGATCTGTCCCGCCAA

GCGATGAGGGGAGCGGCCTGCCTCTCGTGAGCGTCAATCCATACGGTTGCATGTTCGTTT

GTTTCAAAGGCATTACCAAGAAGGGTTTCCAGCTTGACAACTTTCTCATCGGTGGGTGTG

AACTCGCCGGTTAGTGAGTCACCGCCCATTTTGGGAACTACCAGGGGGGTGCCAACGACT

TCCTCCGTCGACATCTCAGACCAATTTCAATTGCCCAACTGCACCGTTCTTTCTATGTTG

AAGTCATGGGCGACTATGAAATCTCCATCTTTGGTCGGTACTACCTCGATCTCGACCATA

TTGAAAAGCCTGAAAGATTTGCAAACTGCCCCTACACTGTTTTCCGGCAAGTGTGGCCCA

TGGAATCCACGGTGAACTATCAATTGGCGGTCGCTTGAAGTCTTTCGAGCGATAAGCCAA

TTGGCGAGGCAGTGTTTCGCGATCGCATCTTTAAGAGGAAAGTCCGATGCAAATCTCCAT

GTAGACCCAGTCGTTTCGGCCAAGGCGGCCTCTAAATCAACTTGAGCAGCAGTTGTGTTA

ACGGAATCTGTTTTGACTGCCATTCTGTAAGAAATGTCAGCAAAGGAAAAGCTAGTTAGA

GAAGGAGAGCTTGCTCAATAGTGTGTCTATTCTCAAATGAACTATACTAGCGCAATGAGT

TGGTGTTGTTCGCTGATGGAAAAAAGGAAACGCATCCCCCTCTTCTACAAAATTCTGTGG

GCCCGGAGGGACTCCGTATGAAAGTGCACAGCATGTGGACGCATCACCATGCAATTAGCA

AGCTTAGCCAATGTCGTGTCTGTCAGAGTGTGCAGCATGTGAGCCAAGCGCAATTACAAT

AAAAAAATAATATAAAAACTCTGCCGAGGTCCGAACCGTCTCAAATGCTTTGAGGGCCAT

CCAAGCCAAGCGCACATAACGCCTAGCACACCATTGTTGCGCCGCTCGAGCATTAATGGT

TCGGGACCTGGTGTCATCTGGAAACCTATGAAGCATGGACACGGCAAAATCAGCGCCGTG

GCGGTGTCAGACACGAGGACACGTCGGGGACACGTACAG

>ss0666_1

tcaccttgaagtatcaatttgatctagaagccaatccaaactcattgaaaggcctgtcat

caaccggtcaacacggttcaatgtttgcttGAGTGGCGCAGCAATGGTGTGCTAGGCGTT

AGGTGGGCCTGGCTGGGATGGCCCTCAAAGCATTTGAGACAGTTCGGACCTCGGCAGAGA

TTTTATATTATTTTTTTGTTGTAATCGCGTTTGGCCCACATGCTGCACACTCCGACAGAC

ACGGCATTGGCTAAACCCGCTAATTGCGTGGTGATGCGTCCACGTGCTGCGCACTTTCAT

GCGGAGTCCCCCCGAGCCCGTAGAATTTTGTAGAAGAGGGGGACGCGTTTCCTTTTTCCC

ATCAGCAAACAACACCAACTCACTGTGCTAGTATAGTTCATCTGAGAACAGACACACTGC

TGAGCAAGCTCTCCTTCTCCAACTAGCTTTTTCTTTGCTGACATTTCCTACAAAATGGCA

GCAAAACAGATTCCGTTAACACAACTGCTGCTCAGGTTGATTTAGAGGCCGCCTTGGCCG

AAGCGACCGGGTCTACATGGAGATTTGCATCAAACTTTCCTCTTAAAGATGCGATCGCAA

AACACTGCCTCGCCAATTGGCTTATCGCTCGAAAGACTTCAAGCAACCACCAATTGATAG

TTCACCGTGAATTCCATGGGCCACACCTGCCGGAAAACAGCGTAGGGGCAGTCTGCAAAT

CTCTCAGACTTTTTAATATGGTCGAGATCAACGTAGTACCGACCAAAGATAGAGATTTCA

TAGTCGTCCACGACTTCAACACAAAAAGAACGACGCAGTTAGGCAATTGAAATTGGTCTG

ATATGTCGACGAAAGAGGCCGTTGGCACCCCCCTAGTAGTTCCCATAATGGGCGGTGACT

CCCTAACCAGCGAGTTCACACCCACCGATGAGAAAGTTCTCAAGCAAGAAACCCTTCTCA

GGAATGCCTTTGAAACAAACAAACAGGCAACCATACGGATTGACGCTCACGAGAGGCAGG

CCGCTCCCCTCGTCGCTTGGCTGAGCCATAGGCCGGCCTATCACGACAAGACAATCGTTA

TCTTTCATACATATGGATACAACAATGGACACAAGTTCGCCTTGGCGGTCGATCAAGCCG

ACGCTACAGAAAATTGGCGCGAAACTGTAGCGCTAATGCCAAACATTCTGCCAAATTAAC

TCTCTAGGCTCGCGAAGCAATATCCACCAAATATCACGTCGCATTTTTACGACCTCGTTG

AGGCCGGCAAGAATTGGTTCGCGTCTGTCTTCTCCCAGAATATGATGGTGGACGTCACTT

TCCTCAAGATTGCGAGCACTGGCGAAGGACAGACTAACAAAGTCGTTGATCCAAATGGAA

ATGAGGTTCTGCATCCTAAAGAGAAGAAGGAATATCTCCGCGATTCAGCTTCAATTGAAA

TCGCGAAATGAATCTGCGCCACCCATCCCGGAATCGAGATTGGAGGCGTCACACGAACTT

ATGCTTATAAGAGTCGAGGACATCGCCACAAAATCGATCTTTGGACGGGTCAACcgaagc

ggtggccgacccacgcgacgaagtacatctgggaagatttcGCCAAGCCTGGAAATGCGC

TCAGATGGGGTCAAACGATTATCTCTGATCGCATTGGCGACGACCTTGCAGCTTGTGTGC

TTCAAAGCCGTGGAGAAAATTTTGTCAGCAGTCATGAATATCCCGCTGTTCATGTCGATT

AGATGGGCGATCTGTGTCTTAGTCTAAGCTTCTGCTTAACGCATCTTTGCTACTTTAATG

TATTTTTTTAATGATATTGTTGGAGTTTTCAGCACATCTTAATCGCAAGTAGATATTTTT

TTTGAATCGGATTCGACTTCAAGCAGATGTACTTCTTTGTCTCTCGATAAATCTAATGAC

CCTGCGAGCCGGCCTTTCGCCTTAAACTAATTTCTGAGAGTTAGGTCGGCGCCTCTCGTG

GTTTCCATGTTCTATGGAAACGGCCTCTCGGGACTTGCCTTCCCGCAACTCATCGAGCTT

GCCTGGCCCGCTCGGGTGGTGGCGCAAGCCATCGTTAAATCGTTCGTAATCCTCTACTCT

CGCATAAATGAGAGGGGAATCCAAAGATGATCACTTTTCAGCTAATTGGAATCTGCAATC

CTAAAAGAGGCAAGCACTATGTGGGGTCCACAACTTATAGAATTTTGGCCTAACCATTCT

GCATAATGGATCATGACATGGGCCATTTATGGCCGCAAAAACTGGCACAAATTCCAACCT

GTTCAGCACAATGAACAAGTTCTTCCAATTGGAATAAAGGAGGTTAAAATTATTCGAGTA

GACATGATGTGCCGGCACGCCACTAGCCTCGCATTGATGCCACCCTTTCTTGCGCACACT

TATATAATGCTGAGCTAATAATAGAGATAAAAATAGTCAGCTTGTAAACTGGACCTTTAC

TACTGCCCCTAATCTCAAGATCAAAGCGACATATACCTCCTCTCTTTCAAGTCTTTTTGC

AACTTGTTCCAAAGCAAGCGTTCGTGAATCTGCCAGGCAGCCTTCACGAAAATGGCTGAT

CAACTATGTGAGGGGCGTTGGTCCGAAGCCAATCTATCATTCGTTGGGATATCCATCACC

CCTGAATTCATCGATGAGGCCCTCAGAGGCTTTTGCCAAAAGAGCCGTTGGCTACAAAGA

GGTTTACGTTCGGGTTGATCGAGATCCTAAAGTTGCCTGGGAAGGAGCGCTCAATGGTTA

CCTCGGCAAATATTCTGGCGTCaaaaATGTTCATTTTGCGCCGAACAACGCCGCGATTGG

AAAGCCCCCATGCCATATCGACATTTTGCTTCGGAAAAAATTTAGCGGCGAATTTCTTGT

GACGCTCGAAGGTGTGGCAATTTCAAACGGTGCAACTAGTTGGCTTGCGCTCGCGTGCGT

AAACCTTCTCTTGAAGGCAAGACAAGATATTGATGTCTTCTTGTTTGGGGCGGGAAAAGT

CGCCGAGGCTGTGATTCTTGCCCTCAATCACGGTGCGGCTGCAAAAATCAAAACCATGGC

AGTGCTCAGCCAAGCTCCAATCGAAGGTGGGGTTCCAGCTCGATGTCGTCATTGATCGGA

AGGTTATACCAAAAGCCAAACTCGTCATAACAGCAACAAATTCCGAGGAGCTCGCACTCG

AGGCCGACGAAATTGCGCCAAATGCAGTaacCATATCCCTCGGAAAAGATGAATTGCTCG

CCGCCTACTTCGATCGCCTTTTGAACGCAGAGGGTCTGATTATCGGCGACGATCTGGATG

CGATCGAATCGCGCAATGTCAATTCCCTGGTGCTTTACTACTCGAAACGTGATTTGAAGC

TGACAGAACATGGAAGGGATCATTGGATAAAAAACTACGCCAATGTCCTTGTTGATCCAG

CTCTCATGGAGGAACTTAAGACATGGGAGGGGCCAGCCAACTTTTCATCGGTTGGCCTTG

CCAGCCTAGACTTGGCGATGGCCGGCCGACTCTACGAAACTCTTACTGCGAAACTCTCCC

ACCCCCAGTAGACAAGACCCTCTTAGGATTGCGCTCTTCAGAAGGTGCGCCTCCCCAGTC

ACAACCGGAAAGTCACTCAACTTGGCTCAAGTACCCTACTTAGTTTGTCTCTCTGTCGTG

TTTAGTATCAACAACAATAACAAATATTGTAAACAACATATATTTATATGGTAATTATAT

GGCATGTAAGCGTGACAACGATTATGTTTTCCATAGTTTGATAAACTCAACACCCGAAAA

AGCCGGTGACCAAAGGGCTGGGACCTCTTTGCACGAACTCCTATGGCTGCCAGACTATCT

ACAGAGCTTTTTCGAAAGCTGACGCAGCATCTGATCGCTACACAATCCTCGCGGACGTGG

ATGGCGTGGCTACACGCAAGCAGTTTGACTGAGAGTTTTCGAGTGACTATTTTAAAAGGA

CGATATTTCGTCCCAAGCAATAAAACTCGCTCACATGACTAAGTCGTTGCTTTAATTCAG

CTGCGTGAAAAGATTTAAAGGGCAGAACTGTTTGTGTTTCGTATCCAGTTCACCGGCAAT

TATTGGCTACGCATGAAGCTCATTATCGTCGATTCTTTACCCAAACACTGATGTCATTCC

AAATGAGTTGGGCCAAAGATGTACTCATGTCGGACAGGCGCGATTTGGACTTCGTGATCC

TCAAGTCTATCGCAGTGCTGCTTTATTACAGGCTATGCTTCCACCCTCCATTATTTGGTC

AACTGGACCTGGCTTTTGACAAGAATAGGCCAGCGTTCATCTATCTATCTTTCAAAAACA

TGCAGTTGCTGTTGCGTAAAGGATGTAACATCGGATTTGGTGTGGAGACGGTGCTTGCTA

CGACTATACTGCCTTACTGTAGTGATCTGTCCTGCCAAGCGACGAGGGGAGCAGCCTGCC

TCTCGTGAGCGTCAATCCATACAGTTGCATGTTCGTTTGTTTCAAAGGCATTCCCAAGAA

GGGTTTCCAGCTTGACAACTTTCTCATCGGTGGGTGTGAACTAGCCGGTTAGTGAGTCAC

CGCCCATTTTGGGAACTACCAGGGGGTGCCAACGACCTCCTCCGTCGACATCTCAGACCA

ATTTCAATTGCTCAACTGCGCCGTTCTTTCTATGTTGAAGTCATGGGCGACTATGAAATC

TCCATCTTTGGTCGGTACTACCTCGATCTCGACCATATTGAAAAGCCTGAGAGATTTGCA

AATTGCCCCTACACTGTTTTCCGGCAAGTGTGGCCCATGGAATCCACGGTGAACTATCAA

TTGGCGGTCGCTTGAAGTCTTTCGAGCGATAAGCCAATTGGCGAGGCAGTGTTTCGCGAT

CGCATCTTTAAGAGGAAAGTCCGATGCAAATCTCCATGTAGACCCGGTTGCTTCGGCCAA

GGCGGCCTCTAAACCAACTTGAGCAGCAGTTGTGTTAACGGAATCTATTTTGACTACCAT

TTTGTAAGAAATGTCAGCAAAGGAAAAGCTAGTTAGAGAAGGAGAGCTTGCTCAATAGTG

TGTCTATTCTCATATGAACTATACTAGCGCAATGAGTTAGTGTTGTTCGCTGATGGGAAA

AAGGAAATGCATCCCCCTCTTCTTCAAAATTCTGTGGGCCCGGAGGGACTCCGTATGAAA

GTGCACAGCATGTGGACGCATCACCATGCAATTAGCGAGCTTAGCCAATGCCGTGTCTGT

CAGAGTGTGCAGCATGTGGGCCAAGCGCAATTACAATAAAAAAATAATATAAAAACTCTG

CCGAGGTCTGAACCGTCTCAAATGCTTTGAGGGCCATCCAAGCCAAGCGCACCTAACGCC

TAGCACACCATTGTTGCGCCGCTCGAGCATTAATGGTCCGGGACCTGGTGTCATCTGGAA

ACCTAtgaagcatggacacggcaaaatcagcgccgtggcggtgtcagacaCggggacacg

tcggggacacgtacgg

>ss0666_2

AAGTATCAATTTGATCTAGAAGCCAATCCAAACTCATTGAAAGGCCTGTCATCAACCGGT

CAACACGGTTCAATGTTTGCTTGCGTGGCGCAGCAATGGTGTGCTAGGCGTTAGGTGCGC

CTGGCTTGGATGGCCCTCAAAGCATTTGAGGCAGTTCGGACCTCGGCAGAGATTTTATAT

TATTTTTTTGTTGTAATCGCGCTTGGCCCACATGCTGCACACTCCGATAGACACGGCATT

GGCTAAACCCGCTAATTGCGTGGTGATGCGTCCACGTGCTGCGCACTTTCATGCGGAGTC

CCCCCGAGTCCATAGAATAAGAGGGGGACGCGTTTCCTTTTTCCCATCAGCAAACAACAC

CAACTCACTGTGCTAGTATAGTTCATCTGAGAACAGACACACTGCTGAGCAAGCTCTCCT

TCTCCAACTAGCTTTTTCTTTGCTGACATTTCCTACAGAATGGCAGCAAAACAGATTCCG

TTAACACAACTACTGCTCAGGTTGATTTAGAGGCCGCCTTGGCCGAAGCGATCGAGTCTA

CATGGAGATTTGCATCAGACTTTCCTCTTAAAAATGCAATCGCGAAACACTGCCTCGCCA

ATTGGCTTATCGCTCGAAAGACTTCAAGCAACCACCAATTGATAGTTCACCGTGAATTCC

ATGGGCCACACCTGCCGGAAAACAGCGTAGGGGCAGTCTGCAAATCTCTCAGACTTTTTA

ATATGGTCGAGATCAACGTAGTACCGACCAAAGATAAAGATTTCATAGTCGTCCACGACT

TCAACACAGAAAGAACGACGCAGTTAGGCAATTGAAATTGGTTTGATATGTCGACGAAAG

AGGCTGTTGGCACCCCCTTGGTAGTTCCCATAATGGGCGGTGACTCCCTAACCAGCGAGT

TCACACCCACCGATGAGAAAGTTGTCAAGCAAGAAACCCTTCTCAAGAATGCCTTTGAAA

CAAACAAACAGGCAACCATACGGATTGACGCTCACGAGAGGCAGGCCGCTCCCCTCGTCG

CTTGGCTGAGCCATAGGCCGGCCTATCACGACAAGACAATCGTTATCTTTCATACATATG

GATACAAAAATGGACACGAGTTCGCCTTGGCGGTCAATCAAGCCGGCGCTACAAAAAATT

GGCGCGAAACTGTAGCGCTAATGCCAAACATTCTGCCGAATGAACTCTCTAGGCTCGCGA

AGCAATATCCACCAAATTGCACGTCGCATTTTTACGACCTCGTTGAGGCCAGCAAGAATT

GGTTCGCATCTGTCTTCTCCCAGAATATGATGGTGGTCGTCACTTTCCTCAAGATTGCGG

GCACTGGCGAAGGACAGACTAACAAAGTCGTTGATCCAAATGGAAATGAGGTTCTGCATC

CTAAAGAGGAGGAGGAATATCTCCGCGATTCAGCTTCAATCGAAATCGCGAAATGAATCT

GCGCCACCCATCCCGGAATCGAGATTGGAGGCGTCACACGAACTTATGCTTATAAGAGTC

GAGGACATCGCTACAAAATCAATCTTTGGACGGGTCAACCGAAGCGGTGGCCGACCCACG

CGACGAAGTACATCTGGGAAGATTTCGCCAAGCCTGGAAATGCGCTCAGATGGGGTCAAA

CGATTATCTCTGATCGCATTGGTGACGACCTTGCAGCTTGTGTGCTTCAAAGCCGTGGAG

AAAATTTTGTCAGCAGTCATGAATATCCTGCTGTTCATGTCGATTAGATGGGCGATCTGT

GTCTTAGTCTAAGCTTCCGCTTAACGCATCTTTGCTACTTTAATGTATTTTTTTAATGAT

ATTGTTGGAGTTTTGAGCACATCTTAATCGCAAGTAGATATTTTTTTTGAATCGGATTCG

ACTTCAAGCAGATGTACTTCTTTGTCTCTCGATAAATCTAATGACCCTGCGAGCCGGCCT

CTCACCTTAAACTAATTTCTGAGAGTTAGGTCGGCGCCTCTCGTGGTTTCCATGTTCTAT

GGAAACGGCCGCTCGGGACTTGCCTTCCCGCAACTCATCGAGCTTGCCTGGCCCGCTCGG

GTGGTGGCACAAGCCATCGTTAAATCGTTCGTAATCCTCTACTCTCGCATAAATGAGAGG

GGAATCCAAAGATGATCACTTTTCAGCTAATTGGAATCTGCAATCCCAAAAGAGGCAAGC

ACTATGTGGGGTCCACAACTTATAGAATTTTGGCCTAACCATTCTGCATAATGGATCATG

ACATGGGCCATTTATGGCCACAAAAACTGGCACAAATTCCAACCTATTCAGCACAATGAA

CAAGTTCTTCCAATTGGAATAAAGGAGGTTAAAATTATTCGAGTAGACATGACGTGCTGG

CACGCCACTAGCCTCGCATTGATGCCACTCTTTCTTGCGCACACTTATATAATGCTGAGC

TAATAATAGAGATAAAAATAGTCAGCTTGTAAACTGGACCTTTATTACTGCCCCTAATCT

CAAGATCAAAGCGACATATACCTCTTCTCTTTCAAGTCTTTTTGCAACTTGTTCCAAAGC

AAGCGTTTGTGAATCTGCCAGGCAGCCTTCACGAAAATGGCTGATCAACTGTGTGAGGGG

CGTTGGTCCGAAGCCAATCTATCATTCGTTGGGATATCCATCACCCCTGAATTCATCGAT

GAGGCCCTCAGAGGTTTTTGGGAGTCTTTTGCCAAAAGAGCCGTTGGCTACAAAGAGGTT

TACGTTCAGGTTGATCGAGATCCTAAAGTTGCCTGGGAAGGAGCGCTCAATGGTTACCTC

GGCAAATATTCTGGCGTAAAAAATGTTCATTTTGCGCCGAACAACGCCACGATTGGAAAG

CCCCCACGCCATATCGACATTTTGCTTCGGAAAAAATTTAGCGGCGAACTTCTTGCAACG

CTCGAAGGTGTGGCAATTTCAAACGGTGCAACTGGTTGGCTTGCGCTCGCATGTGTAAAC

CTTCTCTTGAAGGCAAGACAAGATATTGATGTCTTCTTGTTTGGGGCAGGAAAAGTCACC

GAGGCTGTGATTCTTGCCCTCAATCATGGTGCGGCTGCAAAAATCAAAACCATGGCAGTG

CTCAGCCAAGCTCCAATTGAAGGTGGGGTTCCAGCTCGATGCCATCATTGATCGGAAGGT

TATACCAAAAGCCAAACTCGTCATAATAGCAACAAATTCTGAGGAGCTCGGTACTCGAGG

CCGACGAAATTGCGCCAAATGCAGTAACCATATCCCTCGGAAAAGATGAATTGCCCGCCG

CCTACTTCGATCGCCTTTTGAACGCAGAGGGTCTGATTATTGGCGACGATCTGGATGCGA

TCGAATCGCGCAATGTCAATTCCCTGGTGCTTTACTACTCGAAACGTGATTTGAAGCTGA

CAGAACATGGAACGGATCATTGGATAAAGAACTACGCCAATGTCCTTGCTGATCCAGCTC

TCATGGAGGAACTTAAGACATGAGAGGGGCCAGCCAACTTTTCATCGGTTGgccttgcca

gcctagacttggcGATGGCCGGCCGACTCTACGAAACTCTTACTGCGAAACTCTCCCACC

CCCAGTAGACAAAACCCTCTTAGGATTGCGCTCTTCAGAAGGTGCGCCTCCCCAGTCACA

ACCGGAAAGTCACTCAACTTGGCTCAAGTACCCTACTTAGTTTGTCTCTCTGTTGTGTTT

AGTATCAGCAACAATAACAAATATTGTAAATAACATATATTTATATGGTAATTATATGGC

ATGTAAGCGTGACAACGATTATGTTTTCCATAGTTTGATAAACTCAACACCCGAAAAAGC

CGGTGACCAAAGGGTTGGGACCTCTTTGCACGAACTCCTATGGCTGCCAAACTATCTACA

GAGCTTTGACGCAGCATCTGATCGCCACACAATCATCGCGGACGTGGATGGCGTGGCTAC

ATGCAagCAGTTTGACTGaGAGTTTTCGAGTgactattttaaaaGGACAATATTTCGTCC

CAAGCGATAAAACTCGCTCACATGACTAAGTCGTTGCTTTAATTCAACTGCGTGAAAAGA

TTTAAAGGGCAGAACTGTTTGTGTTTCGTATCCAGTTCACCGGCAATTATTGGCTACGCA

TGAAGCTCATTATCGTCGATTCTTTACCCAAACACTGATGCCATTCCAAATGAGTTGGGC

CAAAGAYGTACTCATGTCGGACAGGCGCGATTTGGACTTCGTGATCCTCAAGTCTATCGC

AGTGCTGCTTTATTACAGGCTATGCTTCCACCCTCCATTATTTGGTCAACTGGACCTAGC

TTTTGACAAGAATAGGCCAGCGTTCATCTATCTATCTTTCAAAAACATGCAGTTGCTGCG

TAAAGGATGTAACATCGGATTTGGTGTGGAGACGGTGCTCGCTACAACTATACTGCCTTA

CTGTAGTGATCTGTCCCGCCAAGCGATGAGGGGAGCGGCCTGCCTCTCGTGAGCGTCAAT

CCATACGGTTGCATGTTCGTTTGTTTCAAAGGCATTCCCAAGAAGGGTTTCCAGCTTGAC

AACTTTCTCATCGGTGGGTGTGAACTCGCCGGTTAGTGAGTCACCGCCCATTTTGGGAAC

TACCAGGGGGGTGCCAACGACTTCCTCCGTCGACATCTCAGACCAATTTCAATTGCCCAA

CTGCACCGTTCTTTCTATGTTGAAGTCATGGGCGACTATGAAATCTCCATCTTTGGTCGG

TACTACCTCGATCTCGACCATATTGAAAAGCCTGAAAGATTTGCAAACTGCCCCTACACT

GTTTTCCGGCAAGTGTGGCCCATGGAATCCACGGTGAACTATCAATTGGCGGTCGCTTGA

AGTCTTTCGAGCGATAAGCCAATTGGCGAGGCAGTGTTTCGCGATCGCATCTTTAAGAGG

AAAGTCCGATGCAAATCTCCATGTAGACCCAGTCGTTTCGGCCAAGGCGGCCTCTAAATC

AACTTGAGCAGCAGTTGTGTTAACGGAATCTGTTTTGACTGCCATTCTGTAAGAAATGTC

AGCAAAGGAAAAGCTAGTTAGAGAAGGAGAGCTTGCACAATAGTGTGTCTATTCTCAAAT

GAACTATACTAGCGCAATGAGTTGGTGTTGTTCGCTGATGGAAAAAAGGAAACGCATCCC

CCTCTTCTACAAAATTATGTGGGCCCGGAGGGACTCCGTATGAAAGTGCACAGCATGTGG

ACGCATCACCATGCAATTAGCAAGCTTAGCCAATGTCGTGTCTGTCAGAGTGTGCAGCAT

GTGAGCCAAGCGCAATTACAATAAAAAAATAATATAAAAACTCTGCCGAGGTCCGAACCG

TCTCAAATGCTTTGAGGGCCATCCAAGCCAAGCGCACCTAACGCCTAGCACACCATTATT

GCGCCGCTCGAGCATTAATGGTTTGGGACTTGGTGTCATCTGGaaacctatgaagcatgg

acacggca

>ss0665_1

aagCCTCTCCAAATTTCCCTCACCTTGAAGTATCAATTTGATCTAGAAGCCAATCCAAAC

TCATTGAAAGGCCTGTCATCAACCTGTCAACACGGTTCAATGTTTGCTCGAGTGGCGCAG

CAATGGTGTGCTAGGCGTTAGGTGCGCCTGGCTGGGATGGCCCTCAAAGCATTTGAGACA

GTTTGGACCTCGGCAGAGATTTCATATTATTTTTTTGTTGTAATCGCGTTTGGCCCACAT

GCTGCACACTCCGACAGACACGGCATTGGCTAAACCCGCTAATTGCGTGGTGATGCGTCC

ACGTGCTGCGCACTTTCATGCGGAGTCCCCCCGAGCCCGTAGAATTTTGTAGAAGAGGGG

GACGCGTTTCCTTTTTTCCATCAGCAAACAACACCAACTCACTATGCTAGTATAGTTCAT

CTGAGAACAGACACACTGCTGAGCAAGCTCTCCTTCTCCAACTAGCTTTTTCTTTGCTGA

CATTTCCTACAAAATGGCAGCAAAACAGATTCCGTTAACACAACTGCTGCTCAGGTTGAT

TTAGAGGCCACCTTGGCCGAAGCGACCGGGTCTACATGGAGATTTGCATCAAACTTTCCT

CTTAAAGATGCGATCGCAAAACACTGCCTCGCCAATTGGCTTATCGCTCGAAAGACTTCA

AGCAACCACCAATTGATAGTTCACCGTGAATTCCATGGGCCACACCTGCCGGAAAACAGC

GTAGGGGCAGTCTGCAAATCTCTCAGACTTTTTAATATGGTCGAGATCAACGTAGTACCG

ACCAAAGATAGAGATTTCATAGTCGTCCACGACTTCAACACAGAAAGAACGACGCAGTTA

GGCAATTGAAATTGGTCTGATATGTCGACGAAAGAGGCCGTTGGCACCCCCCTAGTAGTT

CCCATAATGGGCGGTGACTCCCTAACCAGCGAGTTCACACCCACCGATGAGAAAGTTGTC

AAGCAAGAAACCCTTCTCAGGAATGCCTTTGAAACAAACAAACAGGCAACCATACGGATT

GACGCTCACGAGAGGCAGGCCGCTCCCCTCGTCGCTTGGCTGAGCCATAGGCCGGCCTAT

CACGACAAGACAATCGTTATCTTTCATACATATGGATACAACAATGGACACAAGTTCGCC

TTGGCGATCGATCAAGCCGACGCTACAGAAAATTGGCGCGAAACTGTAGCGCTAATGCCA

AACATTCTGCCAAATGAACTCTCTAGGCTCGCGAAGCAATATCCACCAAATATCACGTCG

CATTTTTACGACCTCGTTGAGGCCGGCAAGAATTGGTTCGCATCTGTCTTCTCCCAGAAT

ATGATGGTGGACGTCACTTTCCTCAAGATTGCGGGCACTGGCGAAGGACAGACTAACAAA

GTCGTTGATCCAAATGGAAATGAGGTTCTGCATCCTAAAGAGAAGGAGGAATATCTCCGC

GATTCAGCTTCAATTGAAATCGCGAAATGAATCTGCGCCACCCATCCCGGAATCGAGATT

GGAGGCGTCACACGAACTTATGCTTATAAGAGTCGAGGACATCGCCACAAAATCGATCTT

TGGACGGGTCAACCGAAGCGGTGGCCGACCCACGCGACGAAGTATATCTGGGAAGATTTC

GCCAAGCCTGGAAATGCGCTCAGATGGGGTCAAACGATTATCTCTGATCGCATTGGCGAC

GACCTTGCAGCTTGTGTGCTTCAAAGCCGTGGAGAAAATTTTGTCAGCAGTCATGAATAT

CCCGCTGTTCATGTCGATTAGATGGGCGATCTGTGTCTTAGTCTAAGCTTCCGCTTAACG

CATCTTTGCTACTTTAATGTATTTTTTTAATGATATTGTTGGAGTTTTCAGCACATCTTA

ATCGCAAGTAGATATTTTTTTTGAATCGGATTCGACTTCAAGCAGATGTACTTCTTTGTC

TCTCGATAAATCTAATGACCCTGCGAGCCGGCCTTTCGCCTTAAACTAATTTCTGAGAGT

TAGGTCGGCGCCTCTCGTGGTTTCCATGTTCTATGGAAACGGCCTCTCGGGACTTGCCTT

CCCGCAACTCATCGAGCTTGCCTGGCCCGCTCGGGTGGTGGCGCAAGCCATCGTTAAATC

GTTCGTAATCCTCTACTCTCGCATAAATGAGAGGGGAATCCAAAGATGATCACTTTTCAG

CTAATTGGAATCTGCAATCCTAAAAGAGGCAAGCACTATGTGGGGTCCACAACTTATAGA

ATTTTGGCCTAACCATTCTGCATAATGGATCATGACATGGGCCATTTATGGCCGCAAAAA

CTGGCACAAATTCCAACCTGTTCAGCACAATGAACAAGTTCTTCCAATTGGAATAAAGGA

GGTTAAAATTATTCGAGTAGACATGATGTGCCGGCACGCCACTAGCCTCGCATTGATGCC

ACCCTTTCTTGCGCACACTTATATAATGCTGAGCTAATAATAGAGATAAAAATAGTCAGC

TTGTAAACTGGACCTTTACTACTGCCCCTAATCTCAAGATCAAAGCGACATATACCTCCT

CTCTTTCAAGTCTTTTTGCAACTTGTTCCAAAGCAAGCGTTCGTGAATCTGCCAGGCAGC

CTTCACGAAAATGGCTGATCAACTATGTGAGGGGCGTTGGTCCGAAGCCAATCTATCATT

CGTTGGGATATCCATCACCCCTGAATTCATCGATGAGGCCCTCAAAGGCTTTTGCCAAAA

GAGCCGTTGGCTACAAAGAGGTTTACGTTCGGGTTGATCGAGATCCTAAAGTTGCCTGGG

AAGGAGCGCTCAATGGTTACCTCGGCAAATATTCTGGCGTCAAAAATGTTCATTTTGCGC

CGAACAACGCCGCGATTGGAAAGCCCCCATGCCATATCGACATTTTGCTTCGGAAAAAAT

TTAGCGGCGAATTTCTTGTGACGCTCGAAGGTGTGGCAATTTCAAACGGTGCAACTGGTT

GGCTTGCGCTCGCGTGCGTAAACCTTCTCTTGAAGGCAAGACAAGATATTGATGTCTTCT

TGTTTGGGGCGGGAAAAGTCGCCGAGGCTGTGATTCTTGCCCTCAATCACGGTGCGGCTG

CAAAAATCAAACCATGGCAGTGCTCAGCCAAGCTCCAATCGAAGGTGGGGTTCCAGCTCG

ATGTCGTCATTGATCGGAAGGTTATACCAAAAGCCAAACTCGTCATAACAGCAACAAATT

CCGAGGAGCTCGCACTCGAGGCCGACGAAATTGCGCCAAATGCAGTAACCATATCCCTCG

GAAAAGATGAATTGCTTGCCGCCTACTTCGATCGCCTTTTGAACGCAGAGGGTCTGATTA

TCGGCGACGATCTGGATGCGATCGAATCGCGCAATGTCAATTCCCTGGTGCTTTACTACT

CGAAACGTGATTTGAAGCTGACAGAACATGGAAGGGATCATTGGATAAAAAACTACGCCA

ATGTCCTTGTTGATCCAGCTCTCATGGAGGAACTTAAGACATGGGAGGGGCCAGCCAACT

TTTCATCGGTTGGCCTTGCCAGCCTAGACTTGGCGATGGCCGGCCGACTCTACGAAACTC

TTACTGCGAAACTCTCCCACCCCCAGTAGACAAGACCCTCTTAGGATTGCGCTCTTCAGA

AGGTGCGCCTCCCCAGTCACAACCGGAAAGTCACTCAACTTGGCTCAAGTACCCTACTTA

GTTTGTCTCTCTGTCGTGTTTAGTATCAACAACAATAACAAATATTGTAAACAACATATA

TTTATATGGTAATTATATGGCATGTAAGCGTGACAACGATTATGTTTTCCATAGTTTGAT

AAACTCAACACCCGAAAAAGCTGGTGACCAAAGGGTTGGGACCTCTTTGCACGAACTCCT

ATGGCTGCCAGACTATCTACAGAGCTTTTTCGAAAGCTGACGCAGCATCTGATCGCTACA

CAATCCTCGCGGACGTGGATGGCGTGGCTACACGCAAGCAGTTTGACTGAGAGTTTTCGA

GTGACTATTTTAAAAGGACGATATTTCGTCCCAAGCAATAAAACTCGCTCACATGACTAA

GTCGTTGCTTTAATTCAGCTGCGTGAAAAGATTTAAAGGGCAGAACTGTTTGTGTTTCGT

ATCCAGTTCACCGGCAATTATTGGCTACGCATGAAGCTCATTATCGTCGATTCTTTACCC

AAACACTGATGTCATTCCAAATGAGTTGGGCCAAAGATGTACTCATGTCGGACAGGCGCG

ATTTGGACTTCGTGATCCTCAAGTCTATCGCAGTGCTGCTTTATTACAGGCTATGCTTCC

ACCCTCCATTATTTGGTCAACTGGACCTGGCTTTTGACAAGAATAGGCCAGCGTTCATCT

ATCTATCTTTCAAAAACATGCAGTTGCTGCTGCGTAAAGGATGTAACATCGGATTTGGTG

TGGAGACGGTGCTTGCTACGACTATACTGCCTTACTATAGTGATCTGTCCTGCCAAGCGA

CGAGGGGAGCAGCCTGCCTCTCGTGAGCGTCAATCCATACAGTTGCATGTTCGTTTGTTT

CAAAGGCATTCCCAAGAAGGGTTTCCAGCTTGACAACTTTCTCATCGGTGGGTGTGAACT

AGCCGGTTAGTGAGTCACCGCCCATTTTGGGAACTACCAGGGGGGTGCCAACGACCTCCT

CCGTCGACATCTCAGACCAATTTCAATTGCTCAACTGCGCCGTTCTTTCTATGTTGAAGT

CATGGGCGACTATGAAATCTCCATCTTTGGTCGGTACTACCTCGATCTCGACCATATTGA

AAAGCCTGAGAGATTTGCAAATTGCCCCTAAACTGTTTTCCGGCAAGTGTGGCCCATGGA

ATCCACGGTGAACTATCAATTGGCGGTCGCTTGAAGTCTTTCGAGCGATAAGCCAATTGG

CGAGGCAGTGTTTCGCGATCGCATCTTTAAGAGGAAAGTCCGATGCAAATCTCCATGTAG

ACCCGGTTGCTTCGGCCAAGGCGGCCTCTAAACCAACTTGAGCAGCAGTTGTGTTAACGG

AATCTATTTTGACTACCATTTTGTAAGAAATGTCAGCAAAGGAAAAGCTAGTTAGAGAAG

GAGAGCTTGCTCAATAGTGTGTCTATTCTCATATGAACTATACTAGCGTAATGAGTTAGT

GTTGTTCGCTGATGGGAAAAAGGAAATGCATCCCCCTCTTCTACAAAATTCTGTGGGCCC

GGAGGGACTCCGTATGAAAGTGCACAGCATGTGGACGCATCACCATGCAATTAGCGAGCT

TAGCCAATGCCGTGTCTGTCAGAGTGTGCAGCATGTGGGCCAAGCGCAATTACAATAAAA

AAATAATATAAAAACTCTGCCGAGGTCTGAACCGTCTCAAATGCTTTGAGGGCCATCCAA

GCCAAGCGCACCTAACGCCTAGCACACCATTGTTGCGCCGCTCGAGCATTAATGGTCCGG

GACCTGGTGTCATCTGGAAACCTATGAAGCATGGACACGGCAAAATCAGCGttgtggcgg

tgtcagacacggggacacgtacgggacacgtcatcta

>ss0665_2

tatcaatttgatctagAAGCCAATCCAAACTCATTGAAAGGCCTGTCATCAACCGGTCAA

CACGGTTCAATGTTTGCTTGCGTGGCGCAGCAATGGTGTGCTAGGCGTTAGGTGCGCCTG

GCTTGGATGGCCCTCAAAGCATTTGAGGCAGTTCGGACCTCGGCAGAGATTTTATATTAT

TTTTTTGTTGTAATCGCGCTTGGCCCACATGCTGCACACTCCGATAGACACGGCATTGGC

TAAACCCGCTAATTGCGTGGTGATGCGTCCACGTGCTGCGCACTTTCATGCGGAGTCCCC

CCGAGTCCATAGAATAAGAGGGGGACGCGTTTCCTTTTTCCCATCAGCAAACAACACCAA

CTCACTGTGCTAGTATAGTTCATCTGAGAACAGACACACTGCTGAGCAAGCTCTCCTTCT

CCAACTAGCTTTTTCTTTGCTGACATTTCCTACAGAATGGCAGCAAAACAGATTCCGTTA

ACACAACTACTGCTCAGGTTGATTTAGAGGCCGCCTTGGCCGAAGCGATCGAGTCTACAT

GGAGATTTGCATCAGACTTTCCTCTTAAAAATGCAATCGCGAAACACTGCCTCGCCAATT

GGCTTATCGCTCGAAAGACTTCAAGCAACCACCAATTGATAGTTCACCGTGAATTCCATG

GGCCACACCTGCCGGAAAACAGCGTAGGGGCAGTCTACAAATCTCTCAGACTTTTTAATA

TGGTCGAGATCAACGTAGTACTGACCAAAGATAAAGATTTCATAGTCGTCCACGACTTCA

ACACAGAAAGAACGACGCAGTTAGGCAATTGAAATTGGTTTGATATGTCGACGAAAGAGG

CTGTTGGCACCCCCTTGGTAGTTCCCATAATGGGCGGTGACTCCCTAACCAGCGAGTTCA

CACCCACCGATGAGAAAGTTGTCAAGCAAGAAACCCTTCTCAAGAATGCCTTTGAAACAA

ACAAACAGGCAACCATACGGATTGACGCTCACGAGAGGCAGGCCGCTCCCCTCGTCGCTT

GGCTGAGCCATAGGCCGGCCTATCACGACAAGACAATCGTTATCTTTCATACATATGGAT

ACAAAAATGGACACGAGTTCGCCTTGGCGGTCAATCAAGCCGGCGCTACAAAAAATTGGC

GCGAAACTGTAGCGCTAATGCCAAACATTCTGCCGAATGAACTCTCTAGGCTCGCGAAGC

AATATCCACCAAATTGCACGTCGCATTTTTACGACCTCGTTGAGGCCAGCAAGAATTGGT

TCGCATCTGTCTTCTCCCAGAATATGATGGTGGTCGTCACTTTCCTCAAGATTGCGGGCA

CTGGCGAAGGACAGACTAACAAAGTCGTTGATCCAAATGGAAATGAGGTTCTGCATCCTA

AAGAGAAGGAGGAATATCTCCGCGATTCAGCTTCAATCGAAATCGCGAAATGAATCTGCG

CCACCCATCCTGGAATCGAGATTGGAGGCGTCACACGAACTTATGCTTATAAGAGTCGAG

GACATCGCTACAAAATCAATCTTTGGACGGGTCAACCGAAGCGGTGGCCGACCCACGCGA

CGAAGTACATCTGGGAAGATTTCGCCAAGCCTGGAAATGCACTCAGATGGGGTCAAACGA

TTATCTCTGATCGCATTGGTGACGACCTTGCAGCTTGTGTGCTTCAAAGCCGTGGAGAAA

ATTTTGTCAGCAGTCATGAATATCCTGCTGTTCATGTCGATTAGATGGGCGATCTGTGTC

TTAGTCTAAGCTTCCGCTTAACGCATCTTTGCTACTTTAATGTATTTTTTTAATGATATT

GTTGGAGTTTTGAGCACATCTTAATCGCAAGTAGATATTTTTTTTGAATCGGATTCGACT

TCAAGCAGATGTACTTCTTTGTCTCTCGATAAATCTAATGACCCTGCGAGCCGGCCTCTC

ACCTTAAACTAATTTCTGAGAGTTAGGTCGGCGCCTCTCGTGGTTTCCATGTTCTATGGA

AACGGCCGCTCGGGACTTGCCTTCCCGCAACTCATCGAGCTTGCCTGGCCCGCTCGGGTG

GTGGCACAAGCCATCGTTAAATCGTTCGTAATCCTCTACTCTCGCATAAATGAGAGGGGA

ATCCAAAGATGATCACTTTTCAGCTAATTGGAATCTGCAATCCCAAAAGAGGCAAGCACT

ATGTGGGGTCCACAACTTATAGAATTTTGGCCTAACCATTCTGCATAATGGATCATGACA

TGGGCCATTTATGGCCACAAAAACTGGCACAAATTCCAACCTATTCAGCACAATGAACAA

GTTCTTCCAATTGGAATAAAGGAGGTAAAAATTATTCGAGTAGACATGACGTGCTGGCAC

GCCACTAGCCTCGCATTGATGCCACTCTTTCTTGCGCACACTTATATAATGCTGAGCTAA

TAATAGAGATAAAAATAGTCAGCTTGTAAACTGGACCTTTATTACTGCCCCTAATCTCAA

GATCAAAGCGACATATACCTCTTCTCTTTCAAGTCTTTTTGCAACTTGTTCCAAAGCAAG

CGTTTGTGAATCTGCCAGGCAGCCTTCACGAAAATGGCTGATCAACTGTGTGAGGGGCGT

TGGTCCGAAGCCAATCTATCATTCGTTGGGATATCCATCACCCCTGAATTCATCGATGAG

GCCCTCAGAGGTTTTTGGGAGTCTTTTGCCAAAAGAGCCGTTGGCTACAAAGAGGTTTAC

GTTCAGGTTGATCGAGATCCTAAAGTTGCCTGGGAAGGAGCGCTCAATGGTTACCTCGGC

AAATATTCTGGCGTAAAAAATGTTCATTTTgcgccGAACAACGCCACGATTGGAAAGCCC

CCACGCCATATCGACATTTTGCTTCGGAAAAAATTTAGCGGCGAACTTCTTGCAACGCTC

GAAGGTGTGGCAATTTCAAACGGTGCAACTGGTTAGCTTGCGCTCGCATGTGTAAACCTT

CTCTTGAAGGCAAGACAAGATATTGATGTCTTCTTGTTTGGGGCAGGAAAAGTCACCGAG

GCTGTGATTCTTGCCCTCAATCATGGTGCGGCTGCAAAAATCAAAACCATGGCAGTGCTC

AGCCAAGCTCCAATTGAAGGTGGGGTTCCAGCTCGATGCCATCATTGATCGGAAGGTTAT

ACCAAAAGCCAAACTCGTCATAATAGCAACAAATTCTGAGGAGCTCGGCACTCGAGGCCG

ACGAAATTGCGCCAAATGCAGTAACCATATCCCTCGGAAAAGATGAATTGCCCGCCGCCT

ACTTCGATCGCCTTTTGAACGCAGAGGGTCTGATTATTGGCGACGATCTGGATGCGATCG

AATCGCGCAATGTCAATTCCCTGGTGCTTTACTACTCGAAACGTGATTTGAAGCTGACAG

AACATGGAAGGGATCATTGGATAAAGAACTACGCCAATGTCCTTGCTGATCCAGCTCTCA

TGGAGGAACTTAAGACATGAGAGGGGCCAACCAACTTTTCATCGGTTGGCCTTGCCAGCC

TAGACTTGGCGATGGCCGGCCGACTCTACGAAACTCTTACTGCGAAACTCTCCCACCCCC

AGTAGACAAAACCCTCTTAGGATTGCGCTCTTCAGAAGGTGCGCCTCCCCAGTCACAACC

GGAAAGTCACTCAACTTGGCTCAAGTACCCTACTTAGTTTGTCTCTCTGTTGTGTTTAGT

ATCAGCAACAATAACAAATATTGTAAATAACATATATTTATATGGTAATTATATGGCATG

TAAGCGTGACAACGATTATGTTTTCCATAGTTTGATAAACTCAACACCCGAAAAAGCCGG

TGACCAAAGGGTTGGGACCTCTTTGCACGAACTCCTATGGCTGCCAAACTATCTACAGAG

CTTTGACGCAACATCTGATCGCCACACAATCCTCGCGGACGTGGATGGCGTGGCTACATG

CAAGCAGTTTGACTGAGAGTTTTCGAGTGACTATTTTAAAAGGACAATATTTCGTCCCAA

GCGATAAAACTCGCTCACATGACTAAGTCGTTGCTTTAATTCAACTGCGTGAAAAGATTT

AAAGGGCAGAACTGTTTGTGTTTCGTATCCAGTTCACCGGCAATTATTGGCTACGCATGA

AGCTCATTATCGTCGATTCTTTACCCAAACACTGATGCCATTCCAAATGAGTTGGGCCAA

AGATGTACTCATGTCGGACAGGCGCGATTTGGACTTCGTGATCCTCAAGTCTATCGCAGT

GCTGCTTTATTACAGGCTATGCTTCCACCCTCCATTATTTGGTCAACTGGACCTAGCTTT

TGACAAGAATAGGCCAGCGTTCATCTATCTATCTTTCAAAAACATGCAGTTGCTGCTGCG

TAAAGGATGTAACATCGGATTTGGTGTGGAGACGGTGCTCGCTACAACTATACTGCCTTA

CTGTAGTGATCTGTCCCGCCAAGCGATGAGGGGAGCGGCCTGCCTCTCGTGAGCGTCAAT

CCATACGGTTGCATGTTCGTTTGTTTCAAAGGCATTCCCAAGAAGGGTTTCCAGCTTGAC

AACTTTCTCATCGGTGGGTGTGAACTCGCCGGTTAGTGAGTCACCGCCCATTTTGGGAAC

TACCAGGGGGGTGCCAACGACTTCCTCCGTCGACATCTCAGACCAATTTCAATTGCCCAA

CTGCACCGTTCTTTCTATGTTGAAGTCATGGGCGACTATGAAATCTCCATCTTTGGTCGG

TACTACCTCGATCTCGACCATATTGAAAAGCCTGAAAGATTTGCAAACTGCCCCTACACT

GTTTTCCGGCAAGTGTGGCCCATGGAATCCACGGTGAACTATCAATTGGCGGTCGCTTGA

AGTCTTTCGAGCGATAAGCCAATTGGCGAGGCAGTGTTTCGCGATCGCATCTTTAAGAGG

AAAGTCCGATGCAAATCTCCATGTAGACCCAGTCGTTTCGGCCAAGGCGGCCTCTAAATC

AACTTGAGCAGCAGTTGTGTTAACGGAATCTGTTTTGACTGCCATTCTGTAAGAAATGTC

AGCAAAGGAAAAGCTAGTTAGAGAAGGAGAGCTTGCACAATAGTGTGTCTATTCTCAAAT

GAACTATACTAGCGCAATGAGTTGGTGTTGTTCGCTGATGGAAAAAAGGAAACGCATCCC

CCTCTTCTACAAAATTCTGTGGGCCCGGAGGGACTCCGTATGAAAGTGCATAGCATGTGG

ACGCATCACCATGCAATTAGCAAGCTTAGCCAATGTCGTGTCTGTCAGAGTGTGCAGCAT

GTGAGCCAAGCGCAATTACAATAAAAAAATAATATAAAAACTCTGCCGAGGTCCGAACCG

TCTCAAATGCTTTGAGGGCCATCCAAGCCAAGCGCACCTAACGCCTAGCACACCATTGTT

GCGCCGCTCGAGCATTAATGGTTCGGGACCTGGTGTCATCTGGAAACCTATGAAGCATGG

ACACGGCAAAATCAGCGTTGTGGCGGt

>ss0658_Un

TACAAGCCTCTCCAAATTTCCCTCACCTTGAAGTATCAATTTGATCTAGAAGCMAATCCA

AACTCATTGAAAGGCCTGTCATCAACCGGTCAACACGGTTCAATGTTTGCTCGCGTGGCG

CAGCAATGGTGTGCTAGGCGTTAGGTGCGCCTGGCTTGGATGGCCCTCAAAGCATTTGAG

GCAGTTCGGACCTCGGCAGAGATTTTATATTATTTTTTTGTTGTAATCGCGCTTGGCCCA

CATGCTGCACACTCCGATAGACACGGCATTGGCTAAACCCGCTAATTGCGTGGTGATGCG

TCCACGTGCTGCGCACTTTCATGCGGAGTCCCCCCGAGTCCATAGAATAAGAGGGGGACG

CGTTTCCTTTTTCCCATCAGCAAACAACACCAACTCACTGTGCTAGTATAGTTCATCTGA

GAACAGACACACTGCTGAGCAAGCTCTCTTTCTCCAACTAGCTTTTTCTTTGCTGACATT

TCCTACAGAATGGCAGCAAAACAGATTCCGTTAACACAACTACTGCTCAGGTTGATTTAG

AGGCCGCCTTGGCCGAAGCGATCGAGTCTACATGGAGATTTGCATCAGACTTTCCTCTTA

AAAATGCAATCGCGAAACACTGCCTCGCCAATTGGCTTATCGCTCGAAAGACTTCAAGCA

ACCACCAATTGATAGTTCACCGTGAATTCCATGGGCCACACCTGCCGGAAAACAGCGTAG

GGGCAGTCTGCAAATCTCTCAGACTTTTTAATATGGTCGAGATCAACGTAGTACCGACCA

AAGATAAAGATTTCATAGTCGTCCACGACTTCAACACAGAAAGAACGACGCAGTTAGGCA

ATTGAAATTGGTTTGATATGTCGACGAAAGAGGCTGTTGGCACCCCCTTGGTAGTTCCCA

TAATGGGCGGTGACTCCCTAACCAGCGAGTTCACACCCACCGATGAGAAAGTTGTCAAGC

AAGAAACCCTTCTCAAGAATGCCTTTGAAACAAACAAACAGGCAACCATACGGATTGACG

CTCACGAGAGGCAGGCCGCTCCCCTCGTCGCTTGGCTGAGCCATAGGCCGGCCTATCACG

ACAAGACAATCGTTATCTTTCATACATATGGATACAAAAATGGACACGAGTTCGCCTTGG

CGGTCAATCAAGCCGGCGCTACAAAAAATTGGCGCGAAACTGTAGCGCTAATGCCAAACA

TTCTGCCGAATGAACTCTCTAGGCTCGCGAAGCAATATCCACCAAATTGCACGTCGCATT

TTTACGACCTCGTTGAGGCCAGCAAGAATTGGTTCGCATCTGTCTTCTCCCAGAATATGA

TGGTGGTCGTCACTTTCCTCAAGATTGCGGGCACTGGCGAAGGACAGACTAACAAAGTCG

TTGATCCAAATGGAAATGAGGTTCTGCATCCTAAAGAGAAGGAGGAATATCTCCGCGATT

CAGCTTCAATCGAAATCGCGAAATGAATCTGCACCACCCATCCCGGAATCGAGATTGGAG

GCGTCACACGAACTTATGCTTATAAGAGTCGAGGACATCGCTACAAAATCAATCTTTGGA

CGGGTCAACCGAAGCGGTGGCCGACCCACGCGACGAAGTACATCTGGGAAGATTTCGCCA

AGCCTGGAAATGCGCTCAGATGGGGTCAAACGATTATCTCTGATCGCATTGGTGACGACC

TTGCAGCTTGTGTGCTTCAAAGCCGTGGAGAAAATTTTGTCAGCAGTCATGAATATCCTG

CTGTTCATGTCGATTAGATGGGCGATCTGTGTCTTAGTCTAAGCTTCCGCTTAACGCATC

TTTGCTACTTTAATGTATTTTTTTAATGATATTGTTGGAGTTTTGAGCACATCTTAATCG

CAAGTAGATATTTTTTTTGAATCGGATTCGACTTCAAGCAGATGTACTTCTTTGTCTCTC

GATAAATCTAATGACCCTGCGAGCCGGCCTCTCGCCTTAAACTAATTTCTGAGAGTTAGG

TCGGCGCCTCTCGTGGTTTCCATGTTCTATGGAAACGGCCGCTCGGGACTTGCCTTCCCG

CAACTCATCGAGCTTGCCTGGCCCGCTCGGGTGGTGGCACAAGCCATCGTTAAATCGTTC

GTAATCCTCTACTCTCGCATAAATGAGAGGGGAATCCAAAGATGATCACTTTTCAGCTAA

TTGGAATCTGCAATCCCAAAAGAGGCAAGCACTATGTGGGGTCCACAACTTATAGAATTT

TGGCCTAACCATTCTGCATAATGGATCATGACATGGGCCATTTATGGCCACAAAAACTGG

CACAAATTCCAACCTATTCAGCACAATGAACAAGTTCTTCCAATTGGAATAAAGGAGGTT

AAAATTATTCGAGTAGACATGACGTGCTGGCACGCCACTAGCCTCGCATTGATGCCACTC

TTTCTTGCGCACACTTATATAATGCTGAGCTAATAATAGAGATAAAAATAGTCAGCTTGT

AAACTGGACCTTTATTACTGCCCCTAATCTCAAGATCAAAGCGACATATACCTCTTCTCT

TTCAAGTCTTTTTGCAACTTGTTCCAAAGCAAGCGTTTGTGAATCTGCCAGGCAGCCTTC

ACGAAAATGGCTGATCAACTGTGTGAGGGGCGTTGGTCCGAAGCCAATCTATCATTCGTT

GGGATATCCATCACCCCTGAATTCATCGATGAGGCCCTCAGAGGTTTTTGGGAGTCTTTT

GCCAAAAGAGCCGTTGGCTACAAAGAGGTTTACGTTCAGGTTGATCGAGATCCTAAAGTT

GCCTGGGAAGGAGCGCTCAATGGTTACCTCGGCAAATATTCTGGCGTAAAAAATGTTCAT

TTTGCGCCGAACAACGCCACGATTGGAAAGCCCCCACGCCATATCGACATTTTGCTTCGG

AAAAAATTTAGCGGCGAACTTCTTGCAACGCTCGAAGGTGTGGCAATTTCAAACGGTGCA

ACTGGTTGGCTTGCGCTCGCATGTGTAAACCTTCTCTTGAAGGCAAGACAAGATATTGAT

GTCTTCTTGTTTGGGGCAGGAAAAGTCACCGAGGCTGTGATTCTTGCCCTCAATCATGGT

GCGGCTGCAAAAATCAAAACCATGGCAGTGCTCAACCAAGCTCCAATTGAAGGTGGGGTT

CCAGCTCGATGCCATCATTGATCGGAAGGTTATACCAAAAGCCAAACTCGTCATAATAGC

AACAAATTCTGAGGAGCTCGGTACTCGAGGCCGACGAAATTGCGCCAAATGCAGTAACCA

TATCCCTCGGAAAAGATGAATTGCCCGCCGCCTACTTCGATCGCCTTTTGAACGCAGAGG

GTCTGATTATTGGCGACGATCTGGATGCGATCGAATCGCGCAATGTCAATTCCCTGGTGC

TTTACTACTCAAAACGTGATTTGAAGCTGACAGAACATGGAAGGGATCATTGGATAAAGA

ACTACGCCAATGTCCTTGCTGATCCAGCTCTCATGGAGGAACTTAAGACATGAGAGGGGC

CAGCCAACTTTTCATCGGTTGGCCTTGCCAGCCTAGACTTGGCGATGGCCGGCCGACTCT

ACGAAACTCTTACTGCGAAACTCTCCCACCCCCAGTAGACAAAACCCTCTTAGGATTGCG

CTCTTCAGAAGGTGCGCCTCCCCAGTCACAACCGGAAAGTCACTCAACTTGGCTCAAGTA

CCCTACTTAGTTTGTCTCTCTGTTGTGTTTAGTATCAGCAACAATAACAAATATTGTAAA

TAACATATATTTATATGGTAATTATATGGCATGTAAGCGTGACAACGATTATGTTTTCCA

TAGTTTGATAAACTCAACACCCGAAAAAGCCGGTGACCAAAGGGTTGGGACCTCTTTGCA

CGAACTCCTATGGCTGCCAAACTATCTACAGAGCTTTGTGACGCAGCATCTGATCGCCAC

ACAATCCTCGCGGACGTGGATGGCGTGGCTACATGCAAGCAGTTTGACTGAGAGTTTTCG

AGTGACTATTTTAAAAGGACAATATTTCGTCCCAAGCGATAAAACTCGCTCACATGACTA

AGTCGTTGCTTTAATTCAACTGCGTGAAAAGATTTAAAGGGCAGAACTGTTTGTGTTTCG

TATCCAGTTCACCGGCAATTATTGGCTACGCATGAAGCTCATTATCGTCGATTCTTTACC

CAAACACTGATGCCATTCCAAATGAGTTGGGCCAAAGATGTACTCATGTCGGACAGGCGC

GATTTGGACTTCGTGATCCTCAAGTCTATCGCAGTGCTGCTTTATTACAGGCTATGCTTC

CACCCTCCATTATTTGGTCAACTGGACCTAGCTTTTGACAAGAATAGGCCAGCGTTCATC

TATCTATCTTTCAAAAACATGCAGTTGCTGCTGCGTAAAGGATGTAACATCGGATTTGGT

GTGGAGACGGTGCTCGCTACGACTATACTGCCTTACTGTAGTGATCTGTCCCGCCAAGCG

ATGAGGGGAGCGGCCTGCCTCTCGTGAGCGTCAATCCATACGGTTGCATGTTCGTTTGTT

TCAAAGGCATTCCCAAGAAGGGTTTCCAGCTTGACAACTTTCTCATCGGTGGGTGTGAAC

TCGCCGGTTAGTGAGTCACCGCCCATTTTGGGAACTACCAGGGGGGTGCCAACGACTTCC

TCCGTCGACATCTCAGACCAATTTCAATTGCCCAACTGCACCGTTCTTTCTATGTTGAAG

TCATGGGCGACTATGAAATCTCCATCTTTGGTCGGTACTACCTCGATCTCGACCATATTG

AAAAGCCTGAAAGATTTGCAAACTGCCCCTACACTGTTTTCCGGCAAGTGTGGCCCATGG

AATCCACGGTGAACTATCAATTGGCGGTCGCTTGAAGTCTTTCGAGCGATAAGCCAATTG

GCGAGGCAGTGTTTCGCGATCGCATCTTTAAGAGGAAAGTCCGATGCAAATCTCCATGTA

GACCCAGTCGTTTCGGCCAAGGCGGCCTCTAAATCAACTTGAGCAGCAGTTGTGTTAACG

GAATCTGTTTTGACTGCCATTCTGTAAGAAATGTCAGCAAAGGAAAAGCTAGTTAGAGAA

GGAGAGCTTGCTCAATAGTGTGTCTATTCTCAAATGAACTATACTAGCGCAATGAGTTGG

TGTTGTTCGCTGATGGAAAAAAGGAAACGCATCCCCCTCTTCTACAAAATTCTGTGGGCC

CGGAGGGACTCCGTATGAAAGTGCACAGCATGTGGACGCATCACCATGCAATTAGCAAGC

TTAGCCAATGTCGTGTCTGTCAGAGTGTGCAGCATGTGAGCCAAGCGCAATTACAATAAA

AAAATAATATAAAAACTCTGCCGAGGTCCGAACCGTCTCAAATGCTTTGAGGGCCATCCA

AGCCAAGCGCACCTAACGCCTAGCACACCATTGTTGCGCCGCTCGAGCATTAATGGTTCG

GGACCTGGTGTCATCTGGAAACCTATGAAGCATGGACACGGCAAAATCAGCGCCGTGGCG

GTGTCAGACACGAGGACACGTCGGGGACACGTACAGGAC

>sa0662_2

gtcaacacggttcaatgtttgctcgcgtggcgcagcaatggtgcgctaggcgttagGTGC

GCCTGGCTTGGATGGCCCTCAAAGCATTTGAGGCAGTTCGGACCTCGGCAGAGATTTTAT

ATTATTTTTTTGTTGTAATCGCGCTTGGCCCACATGCTGCACACTCCGATAGACACGGCA

TTGGCTAAACCCGCTAATTGCGTGGTGATGCGTCCACGTGCTGCGCACTTTCATGCGGAG

TCCCCCCGAGTCCATAGAATAAGAGGGGGACGCATTTCCTTTTTCCCATCAGCAAACAAC

ACCAACTCACTGTGCTAGTATAGTTCATCTGAGAACAGACACACTGCTGAGCAAGCTCTC

CTTCTCTAACTAGCTTTTTCTTTGCTGACATTTCCTACAGAATGGCAGCAAAACAGATTC

CGTTAACACAACTACTGCTCAGGTTGATTTAGAGGCCGCCTTGGCCGAAGCGATCGGGTC

TACATGGAGATTTGCATCAGACTTTCCTCTTAAAAATGCAATCGCGAAACACTGCCTCGC

CAATTGGCTTATCGCTCGAAAGACTTCAAGCAACCACCAATTGATAGTTCACCGTGAATT

CCATGGGCCACACCTGCCGGAAAACAGCGTAGGGGCAGTCTGCAAATCTCTCAGACTTTT

TAATATGGTCGAGATCAACGTAGTACCGACCAAAGATAAAGATTTCATAGTCGTCCACGA

CTTCAACACAGAAAGAATGACGCAGTTAGGCAATTGAAATTGGTTTGATATGTCGACGAA

AGAGGCTGTTGGCACCCCCCTGGTAGTTCCCATAATGGGCGGTGACTCCCTAACCAGCGA

GTTCACACCCACCGATGAGAAAGTTGTCAAGCAAGAAACCCTTCTCAAGAATGCCTTTGA

AACAAACAAACAGGCAACCATACGGATTGATGCTCACGAGAGGCAGGCCGCTCCCCTCGT

CGCTTGGCTGAGCCATAGGCCGGCCTATCACGACAAGACAATCGTTATCTTTCATACATA

TGGATACAACAATGGACACGAGTTCGCCTTGGCGGTCGATCAAGCCGGCGCTACAAAAAA

TTGGCGCGAAACTGTAGCGCTAATGCCAAACATTCTGCCGAATGAACTCTCTAGGCTCGC

GAAGCAATATCCACCAAATTGCACGTCGCATTTTTACGACCTCGTTGAGGCCAGCAAGAA

TTGGTTCGCATCTGTCTTCTCCCAGAATATGATGGTGGTCGTCACTTTCCTCAAGATTGC

GGGCACTGGCGAAGGACAGACTAACAAAGTCGTTGATCCAAATGGAAATGAGGTTCTGCA

TCCTAAAGAGAAGGAGGAATATCTCCGCGATTCAGCTTCAATCGAAATCGCGAAATGAAT

CTGCGCCACCCATCCCGGAATCGAGATTGGAGGCGTCACACGAACTTATGCTTATAAGAG

TCGAGGACATCGCTACAAAATCAATCTTTGGACGGGTCAACCGAAGCGGTGGCCGACCCA

CGCGACGAAGTACATCTGGGAAGATTTCGCCAAGCCTGGAAATGCGCTCAGATGGGGTCA

AACGATTATCTCTGATCGCATTGGTGACGACCTTGCAGCTTGTGTGCTTCAAAGCCGTGG

AGAAAATTTTGTCAGCAGTCATGAATATCCTGCTGTTCATGTCAATTAGATGGGCGATCT

GTGTCTTAGTCTAAGCTTCCGCTTAACGCATCTTTGCTACTTTAATGTATTTTTTTAATG

ATATTGTTGGAGTTTTGAGCACATCTTAATCGCAAGTAGATATTTTTTTTGAATCGGATT

CGACTTCAAGCAGATGTACTTCTTTGTCTCTCGATAAATCTAATGACCCTGCGAGCCGGC

CTCTCGCCTTAAACTAATTTCTGAGAGTTAGGTCGGCGCCTCTCGTGGTTTCCATGTTCT

ATGGAAACGGCCGCTCGGGACTTGCCTTCCCGCAACTCATCGAGCTTGCGTGGCCCGCTC

GGGTGGTGGCACAAGCCATCGTTAAATCGTTCGTAATCCTCTACTCTCGCATAAATGAGA

GGGGAATCCAAAGATGATCACTTTTCAGCTAATTGGAATCTGCAATCCCAAAAGAGGCAA

GCACTATGTGGGGTCCACAACTTATAGAATTTTGGCCTAACCATTCTGCATAATGGATCA

TGACATGGGCCATTTATGGCCACAAAAACTGGCACAAATTCCAACCTATTCAGCACAATG

AACAAGTTCTTCCAATTGGAATAAAGGAGGTTAAAATTATTCGAGTAGACATGACGTGCT

GGCACGCCACTAGCCTCGCATTGATGCCACTCTTTCTTGCGCACACTTATATAATGCTGA

GCTAATAATAGAGATAAAAATAGTCAACTTGTAAACTGGACCTTTATTACTGCCCCTAAT

CTCAAGATCAAAGCGACATATACCTCTTCTCTTTCAAGTCTTTTTGCAACTTGTTCCAAA

GCAAGCGTTTGTGAATCTGCCAGGCAGCCTTCACGAAAATGGCTGATCAACTGTGTGAGG

GGCGTTGGTCCGAAGCCAATCTATCATTCGTTGGGATATCCATCACCCCTGAATTCATCG

ATGAGGCCCTCAGAGGCTTTTGGGAGTCTTTTGCCAAAAGAGCCGTTGGCTACAAAGAGG

TTTACGTTCAGGTTGATCGAGATCCTAAAGTTGCCTGGGAAGGAGCGCTCAATGGTTACC

TCGGCAAATATTCTGGCGTAAAAAATGTTCATTTTGCGCCGAACAACGCCACGATTGGAA

AGCCCCCACGCCATATCGACATTTTGCTTCGGAAAAAATTTAGCGGCGAACTTCTTGCGA

CGCTCAAAGGTGTGGCAATTTCAAACGGTGCAACTGGTTGGCTTGCGCTCGCATGTGTAA

ACCTTCTCTTGAAGGCAAGACAAGATATTGATGTCTTCTTGTTTGGGGCAGGAAAAGTCA

CCGAGGCTGTGATTCTTGCCCTCAATCATGGTGCGGCTGCAAAAATCAAAACCATGGCAG

TGCTCAGCCAAGCTCCAATTGAAGGTGGGGTTCCAGCTCGATGCCGTCATTGATCGGAAG

GTTATACCAaaagccaaactcgtcataacagcaacaaattccgaggagctcggtactcga

ggccgaCGAAATTGCGCCAAATGCAGTAACCATATCCCTCGGAAAAGATGAATTGCCCGC

CGCCTACTTCGATCGCCTTTTGAACGCAGAGGGTCTGATTATTGGCGACGATCTGGATGC

GATCGAATCGCGCAATGTCAATTCCCTGGTGCTTTACTACTCAAAACGTGATTTGAAGCT

GACAGAACATGGAAGGGATCATTGGATAAAGAACTACGCCAATGTCCTTGCTGATCCAGC

TCTCATGGAGGAACTTAAGACATGAGAGGGCCAGCCAACTTTTCATCGGTTGGCCTTGCC

AGCCTAGACTTGGCGATGGCCGGCCGACTCTACGAAACTCTTACTGCGAAACTCTCCCAC

CCCCAGTAGACAAAACCCTCTTAGGATTGCGCTCTTCAGAAGGTGCGCCTCCCCAGTCAC

AACCGGAAAGTCACTCAACTTGGCTCAAGTACCCTACTTAGTTTGTCTCTCTGTTGTGTT

TAGTATCAGCAACAACAAATATTGTAAATAACATATATTTATATGGTAATTATATGGCAT

GTAAGCATGACAACGATTATGTTTTCCATAGTTTGATAAACTCAACACCCGAAAAAGCCG

GTGACCAAAGGGTTGGGACCTCTTTGCACGAACTCCTATGGCTGCCAGACTATCTACAGA

GCTTTGACGCAGCATCTGATCGCCACACAATCCTCGCGGACGTGGATGGCGTGGCTACAT

GCAAGCAGTTTGACTGAGAGTTTTCGAGTGACTATTTTAAAAGGACAATATTTCGTCCCA

AGCGATAAAACTCGCTCACATGACTAAGTCGTTGCTTTAATTCAACTGCGTGAAAAGATT

TAAAGGGCAGAACTGTTTGTGTTTCGTATCCAGTTCACCGGCAATTATTGGCTAAGCATG

AAGCTCATTATCGTCGATTCTTTACCCAAACACTGATGCCATTCCAAATGAGTTGGGCCA

AAGATGTACTCATGTCGGACAGGCGCAATTTGGACTTCGTGATCCTCAAGTCTATCGCAG

TGCTGCTTTATTACAGGCTATGCTTCCACCCTCCATTATTTGGTCAACTGGACCTAGCTT

TTGACAAGAATAGGCCAGCGTTCATCTATCTATCTTTCAAAAACATGCAGTTGCTGCTGC

GTAAAGGATGTAACATCGGATTTGGTGTGGAGACGGTGCTCGCTACGACTATACTGCCTT

ACTGTAGTGATCTGTCCCGCCAAGCGATGAGGGGAGCGGCCTGCCTCTCGTGAGCGTCAA

TCCATACGGTTGCATGATCGTTTGTTTCAAAGGCATTCCCAAGAAGGGTTTCCAGCTTGA

CAACTTTCTCATCGGTGGGTGTGAACTCGCCGGTTAGTGAGTCACCGCCCATTTTGGGAA

CTACCAGGGGGGTGCCAACGACCTCCTCCGTCGACATCTCAGACCAATTTCAATTGCCCA

ACTGCACCGTTCTTTCTATGTTGAAGTCATGGGCGACTATGAAATCTCCATCTTTGGTCG

GTACTACCTCGATCTCGACCATATTGAAAAGCCTGAAAGATTTGCAAACTGCCCCTACAC

TGTTTTTCGGCAAGTGTGGCCCATGGAATCCACGGTGAACTATCAATTGGCGGTCGCTTG

AAGTCTTTCGAGCGATAAGCCAATTGGCGAGGCAGTGTTTCGCGATCGCATCTTTAAGAG

GAAAGTTCGATGCAAATCTCCATGTAGACCTAGTCGTTTCGGCCAAGGCGGCCTCTAAAT

CAACTTGAGCAGCAGTTGTGTTAACGGAATCTGTTTTGACTGCCATTCTGTAAGAAATGT

CAGCAAAGGAAAAGCTAGTTAGAGAAGGAGAGCTTGCTCAATAGTGTGTCTATTCTCAAA

TGAACTATACTAGCGCAATGAGCTGGTGTTGTTCGCTGATGGGAAAAAGGAAACGCATCC

CCCTCTTCTACAAAATTCTGTGGGCCCGGAGGGACTCCGTATGAAAGTGCACAGCATGTG

GACGCATCACCATGCAATTAGCAAGCTTAGCCAATGTCGTGTCTGTCAGAGTGTGCAGCA

TGTGAGCCAAGTGCAATTACTATAAAAAAATAATATAAAAACTCTGCCGAGGTCCGAACC

GTCTCAAATGCTTTGAGGGCCATCCAAGCCAAGCGCACCTAACGCCTAGCACACCATTGT

TGCGCCGCTCGAGCATTAATGGTTCGGGACCTGGTGTCATCTGGAAACCTATGAAGCATG

GACACGGCAAAATCAGCGCCGTGGCggtgtcagacacgaggacacgtcg

>sa0662_1

ttggatggccctcaaagcatttgaggcagttcggacctcggcagagattttatattATTT

TTTTGTTGTAATCGCGCTTGGCCCACATGCTGCACACTCCGATAGACACGGCATTGGCTA

AACCCGCTAATTGCGTGGTGATGCGTCCACGTGCTGCGCACTTTCATGCGGAGTCCCCCC

GAGTCCATAGAATAAGAGGGGGACGCGTTTCCTTTTTCCCATCAGCAAACAACACCAACT

CACTGTGCTAGTATAGTTCATCTGAGAACAGACACACTGCTGAGCAAGCTCTCCTTATCC

AACTAGCTTTTTCTTTGCTGACATTTCCTACAGAATGGCAGCAAAACAGATTCCGTTAAC

ACAACTACTGCTCAGGTTGATTTAGAGGCCGCCTTGGCCGAAGCGATCGAGTCTACATGG

AGATTTGCATCAGACTTTCCTCTTAAAAATGCAATCGCGAAACACTGCCTCGCCAATTGG

CTTATCGCTCGAAAGACTTCAAGCAACCACCAATTGATAGTTCACCGTGAATTCCATGGG

CCACACCTGCCGGAAAACAGCGTAGGGGCAGTCTGCAAATCTCTCAGACTTTTTAATAtg

gtcgaGATCAACGTAGTACCGACCAAAGATAAAGATTTCATAGTCGTCCACGACTTCAAC

ACAGAAAGAACGACGCAGTTAGGCAATTGAAATTGGTTTGATATGTCGACGAAAGAGGCT

ATTGGCACCCCCCTAGTAGTTCCCATAATGGGCGGTGACTCCCTAACCAGCGAGTTCACA

CCCACCGACAAGAAAATTGTCAAGCAAGAAACCCTTCTCAAGAATGCCTTTGAAACAAAC

AAACAGGCAACCATACGGATTGACGCTCACGAGAGGCAGGCCGCTCCCCTCGTCGCTTGG

CTGAACCATAGGCCGGCCTATCACGACAAGACAATCGTTATCTTTCATACATATGGATAC

AAAAATGGACACGAGTTCGCCTTGGCGGTCAATCAAGCCGGCGCTACAAAAAATTGGCGC

GAAACTGTAGGCTCTGGTAGTTCCCAAACATTCTGCCGAATGAACTCTCTAGGCTCGCGA

AGCAATATCCACCAAATTGCACGTCGCATTTTTACGACCTCGTTGAGGCCAGCAAGAATT

GGTTCGCATCTGTCTTCTCCCAGAATATGATGGTGGTCGTCACTTTCCTCAAGATTGCGG

GCACTGGCGAAGGACAGACTAACAAAGTCGTTGATCCAAATGGAAATGAGGTTTTGCATC

CTAAAGAGAAGGAGGAATATCTCCGCGATTCAGCTTCAATCGAAATCGCGAAATGAATCT

GCGCCACCCATCCCGGAATCGAGATTGGAGGCGTCACACGAACTTATGCTTATAAGAGTC

GAGGACATCGCTACAAAATCAATCTTTGGACGGGTCAACCGAAGCGGTGGCCGACCCACG

CGACGAAGTACATCTAGGAAGATTTCGCCAAGCCTGGAAATGCGCTCAGATGGGGTCAAA

CGATTATCTCTGATCGCATTGGTGACGACCTTGCAACTTGTGTGCTTCAAAGCCGTGGAG

AAAATTTTGTCAGCAGTCATGAATATCCTGCTGTTCATGTCGATTAGATGGGCGATCTGT

GTCTTAGTCTAAGCTTCCGCTTAACACATCTTTGCTACTTTAATGTATTTTTTTAATGAT

ATTGTTGGAGTTTTGAGCACATCTTAATCGCAAGTAGATATTTTTTTTGAATCGGATTCG

ACTTCAAGCAGATGTACTTCTTTGTCTCTCGATAAATCTAATGACCCTGCGAGCCGGCCT

CTCACCTTAAACTAATTTCTGAGAGTTAGGTCGGCGCCTCTCGTGGTTTCCATGTTCTAT

GGAAACGGCCGCTCGGGACTTGCCTTCCCGCAACTCATCGAGCTTGCCTGGCCCGCTCGG

GTGGTGGCACAAGCCATCGTTAAATCGTTCGTAATCCTCTACTCTCGCATAAATGAGAGG

GGAATCCAAAGATGATCACTTTTCAGCTAATTGGAATCTGCAATCCCAAAAGAGGCAAGC

ACTATGTGGGGTCCACAACTTATAGAATTTTGGCCTAACCATTCTGCATAATGGATCATG

AGATGGGCCATTTATGGCCGCAAAAACTGGCACAAATTCCAACCTATTCAGCACAATGAA

CAAGTTCTTCCAATTGGTATAAAGGAGGTTAAAATTATTCGAGTAGACATGACGTGCTGG

CACGCCACTAGCCTCGCATTGATGCCACTCTTTCTTGCGCACACTTATATAATGCTGAGC

TAATAATAGAGATAAAAATAGTCAGCTTGTAAACTGGACCTTTATTACTGCCCCTAATCT

CAAGATCAAAGCGACATATACCTCTTCTCTTTCAAGTCTTTTTGCAACTTGTTCCAAAGC

AAGCGTTTGTGAATCTGCCAGGCAGCCTTCACGAAAATGGCTGATCAACTGTGTGAGGGG

CGTTGGTCCAAAGCCAATCTATCATTCGTTGGGATATCCATCACCCCTGAATTCATTGAT

GAGGCCCTCAAAGGCTTTTGGGAGTCTTTTGCCAAAAGAGCCGTTGGCTACAAAGAGGTT

TACGTTCAGGTTGATCGAGATCCTAAAGTTGCCTGGGAAggagcgctcaATGGTTACCTC

GGCAAATATTCTGGTGTAAAAAATGTTCATTTTGCGCCGAACAACACCACGATTGGAAAG

CCCCCACGCCATATCGACATTTTGCTTCGGAAAAAATTTAGCGGCGAACTTCTTGCGACG

CTCGAAGGTGTGGCAATTTCAAACGGTGCAACTGGTTGGCTTGCGCTCGCATGTGTAAAC

CTTCTCTTGAAGGCAAGACAAGATATTGATGTCTTCTTGTTTGGGGCAGGAAAAGTCGCC

GAGGCTGTGATTCTTGCCCTCAATCATGGTGCGGCTGCAAAAATCAAAACCATGGCAGTG

CTCAGCCAAGCTCCAATTGAAGGTGGGGTTCTAGCTCGATGCCGTCATTGATTGGAAGGT

TATACCAAAAGCCAAACTCGTCATAACAGCAACAAATTCCGAGGAGCTCGGTACTCGAGG

CCGACGAAATTGCGCCAAATGCAGTAACCATATCCCTCGGAAAAGATGAATTGCCCGCCG

CCTACTTCGATCGCCTTTTGAACGCAGAGGGTCTGATTATTGGCGACGATCTGGATGCGA

CCGAATCGCGCAATGTCAATTCCCTGGTGCTTTACTACTCGAAACGTGATTTGAAGCTGA

CAGAACATGGAAGGGATCATTAGATAAAGAACTACGCCAATGTCCTTGCTGATCCAGCTC

TCATGGAGGAACTTAAGACATGAGAGGGGCCAGCCAACTTTTCATCGGTTGGCCTTGCCA

GCCTAGACTTGGCGATGGCCGGCCGACTCTACGAAACTCTTACTGCGAAACTCTCCCACC

CCCAGTAGACAAAACCCTCTTAGGATTGCGCTCTTCAGAAGGTGCGCCTCCCCAGTCACA

ACCGGAAAGTCACTCAACTTGGCTCAAGTACCCTACTTAGTTTGTCTCTCTGTCGTGTTT

AGTATCAGCAACAATAACAAATATTGTAAATAACATATATTTATATAGTAATTATATGGC

ATGTAAGCGTGACAACGATTATGTTTTCCATAGTTTGATAAACTTAACACCCGAAAAAAG

CCGGTGACCAAAGGGTTGGGACCTCTTTGCACGAACTCCTATGGCTGCCAGACTATCTAC

AGAGCTTTTTCGGAAGCTGACGCAGCATCTGATCGCCACACAATCCTCGCGGACGTGGAT

GGCGTGGCTACATGCAAGCAGTTTGATTGAGAGTTTTCGAGTGACTATTTTAAAAGGACA

ATATTTCGTCCCAAGCGATAAAACTCGCTCACATGACTAAGTCATTGCTTTAATTCAACT

GCGTGAAAAGATTTAACGGGCAGAACTGTTTGTGTTTCGTATCCAGTTCACCGGCAATTA

TTGGCTACGCATGAAGCTCATTATCGTCGATTCTTTACCCAAACACTGATGCCATTCCAA

ATGAGTTGGGCCAAAGATGTACTCATGTCGGACAGGCGCGATTTGGACTTCGTGATCCTC

AAGTCTATCGCAGTGCTGCTTTATTACAAGCTATGCTTCCACCCTCCATTATTTGGTCAA

CTGGACCTAGCTTTTGACAAGAATAGGCCAGCGTTCATCTATCTATCTTTCAAAAACATG

CAGTTGCTGCTGTGTAAAGGATGTAACATCGGATTTGGTGTGGAGACGGTGCTCGCTACG

ACTATACTGCCTTACTATAGTGATCTGTCCCGCCAAGCGATGAGGGGAGCGGCCTGCCTC

TCATGAGCGTCAATCCATACGGTTGCATGTTCGTTTGTTTCAAAGGCATTCCCAAGAAGG

GTTTCCAGCTTGACAACTTTCTCATCGGTGGGTGTGAACTCGCCGGTTAGTGAGTCACCG

CCCATTTTGGGAACTACCAGGGGGGTGCCAACGACCTCCTCCGTCGACATCTCAGACCAA

TTTCAATTGCCCAACTGCACCGTTCTTTCTATGTTGAAGTCATGGGTGACTATGAAATCT

CCATCTTTGGTCGGTACTATCTCGATCTCGACCATATTGAAAAGCCTGAAAGATTTGCAA

ACTGCCCCTACACTGTTTTCCGGCAAGTGTGGCCCATGGAATCCACGGTGAACTATCAAT

TGGCGGTCGCTTGAAGTCTTTCGAGCGATAAGCCAATTGGCGAGGCAGTGTTTCGCGATC

GCATCTTTAAGAGGAAAGTCCGATGCAAATCTCCATGTAGACCCAGTCGTTTCGGCCAAG

GCGGCCTCTAAATCAACTTGAGCAGCAGTTGTGTTAACGGAATCTGTTTTGACTGCCATT

CTGTAAGAAATGTCAGCAAAGGAAAAGCTAGTTAGAGAAGGAGAGCTTGCTCAATAGTGT

GTCTATTCTCAAATGAACTATACTAGCGCAATGAGTTGGTGTTGTTCGCTGATGGGAAAA

AGGAAATGCATCCCCCTCTTCTACAAAATTCTGTGGGCCCGGAGGGACTCCGTATGAAAG

TGCACAGCATGTGGACGCATCACCATGCAATTAGCAAGCTTAGCCAATGTCATGTCTGTC

AGAGTGTGCAGCATGTGAGCCAAGCGCAATTACAATAAAAAAATAATATAAAAACTCTGC

CGAGGTCCGAACCGTCTCAAATGCTTTGAAGGCCATCCAAGCCAAGCGCACCTAACGCCT

AGCACACCATTGTTGCGCCGCTCGAGCATTAATGGTTCGGGACCTGGTGTCATCTGGAAA

CCTATGAAGCATGGACACGGCAAAATCAGCGTCGTGGCGGTGTCAGACACGAGGACACgt

cggggac

>ss0624_Un

CTCTCCAAATTTCCCTCACCTTGAAGTATCAATTTGATCTAGAAGCCAATCCAAACTCAT

TGAAAGGCCTGTCATCAACCGGTCAACACGGTTCAATGTTTGCTTGCGTGGCGCAGCAAT

GGTGTGCTAGGCGTTAGGTGCGCCTGGCTTGGATGGCCCTCAAAGCATTTGAGGCAGTTC

GGACCTCGGCAGAGATTTTATATTATTTTTTTGTTGTAATCGCGCTTGGCCCACATGCTG

CACACTCCGATAGACACGGCATTGGCTAAACCCGCTAATTGCGTGGTGATGCGTCCACGT

GCTGCGCACTTTCATGCGGAGTCCCCCCGAGTCCATAGAATAAGAGGGGGACGCGTTTCC

TTTTTCCCATCAGCAAACAACACCAACTCACTGTGCTAGTATAGTTCATCTGAGAACAGA

CACACTGCTGAGCAAGCTCTCCTTCTCCAACTAGCTTTTTCTTTGCTGACATTTCCTACA

GAATGGCAGCAAAACAGATTCCGTTAACACAACTACTGCTCAGGTTGATTTAGAGACCGC

CTTGGCCGAAGCGATCGAGTCTACATGGAGATTTGCATCAGACTTTCCTCTTAAAAATGC

AATCGCGAAACACTGCCTCGCCAATTGGCTTATCGCTCGAAAGACTTCAAGCAACCACCA

ATTGATAGTTTACCGTGAATTCCATGGGCCACACCTGCCGGAAAACAGCGTAGGGGCAGT

CTGCAAATCTCTCAGACTTTTTAATATGGTCGAGATCAACGTAGTACCGACCAAAGATAA

AGATTTCATAGTCGTCCACGACTTCAACACAGAAAGAACGACGCAGTTAGGCAATTGAAA

TTGGTTTGATATGTCGACGAAAGAGGCTGTTGGCACCCCCTTGGTAGTTCCCATAATGGG

CGGTGACTCCCTAACCAGCGAGTTCACACCCACCGATGAGAAAGTTGTCAAGCAAGAAAC

CCTTCTCAAGAATGCCTTTGAAACAAACAAACAGGCAACCATACGGATTGACGCTCACGA

GAGGCAGGCCGCTCCCCTCGTCGCTTGGCTGAGCCATAGGCCGGCCTATCACGACAAGAC

AATCGTTATCTTTCATACATATGGATACAAAAATGGACACGAGTTCGCCTTGGCGGTCAA

TCAAGCCGGCGCTACAAAAAATTGGCGCGAAACTGTAGCACTAATGCCAAACATTCTGCC

GAATGAACTCTCTAGGCTCGCGAAGCAATATCCACCAAATTGCACGTCGCATTTTTACGA

CCTCGTTGAGGCCAGCAAGAATTGGTTCGCATCTGTCTTCTCCCAGAATATGATGGTGGT

CGTCACTTTCCTCAAGATTGCGGGCACTGGCGAAGGACAGACTAACAAAGTCGTTGATCC

AAATGGAAATGAGGTTCTGCATCCTAAAGAGAAGGAGGAATATCTCCGCGATTCAGCTTC

AATCGAAATCGCGAAATGAATCTGCGCCACCCATCCCGGAATCGAGATTGGAGGCGTCAC

ACGAACTTATGCTTATAAGAGTCGAGGACATCGCTACAAAATCAATCTTTGGACGGGTCA

ACCGAAGCGGTGGCCGACCCACGCGACGAAGTACATCTGGGAAGATTTCGCCAAGCCTGG

AAATGCGCTCAGATGGGATCAAACGATTATCTCTGATCGCATTGGTGACGACCTTGCAGC

TTGTGTGCTTCAAAGCCGTGGAGAAAATTTTGTCAGCAGTCATGAATATCCTGCTGTTCA

TGTCGATTAGATGGGCGATCTGTGTCTTAGTCTAAGCTTCCGCTTAACGCATCTTTGCTA

CTTTAATGTATTTTTTTAATGATATTGTTGGAGTTTTGAGCACATCTTAATCGCAAGTAG

ATATTTTTTTTGAATCGGATTCGACTTCAAGCAGATGTACTTCTTTGTCTCTCGATAAAT

CTAATGACCCTGCGAGCCGGCCTCTCACCTTAAACTAATTTCTGAGAGTTAGGTCGGCGC

CTCTCGTGGTTTCCATGTTCTATGGAAACGGCCGCTCGGGACTTGCCTTCCCGCAACTCA

TCGAGCTTGCCTGGCCCGCTCGGGTGGTGGCACAAGCCATCGTTAAATCGTTCGTAATCC

TCTACTCTCGCATAAATGAGAGGGGAATCCAAAGATGATCACTTTTCAGCTAATTGGAAT

CTGCAATCCCAAAAGAGGCAAGCACTATGTGGGGTCCACAACTTATAGAATTTTGGCCTA

ACCATTCTGCATAATGGATCATGACATGGGCCATTTATGGCCACAAAAACTGGCACAAAT

TCCAACCTATTCAGCACAATGAACAAGTTCTTCCAATTGGAATAAAGGAGGTTAAAATTA

TTCGAGTAGACATGACGTGCTGGCACGCCACTAGCCTCGCATTGATGCCACTCTTTCTTG

CGCACACTTATATAATGCTGAGCTAATAATAGAGATAAAAATAGTCAGCTTGTAAACTGG

ACCTTTATTACTGCCCCTAATCTCAAGATCAAAGCGACATATACCTCTTCTCTTTCAAGT

CTTTTTGCAACTTGTTCCAAAGCAAGCGTTTGTGAATCTGCCAGGCAGCCTTCACGAAAA

TGGCTGATCAACTGTGTGAGGGGCGTTGGTCCGAAGCCAATCTATCATTCGTTGGGATAT

CCATCACCCCTGAATTCATCGATGAGGCCCTCAGAGGTTTTTGGGAGTCTTTTGCCAAAA

GAGCCGTTGGCTACAAAGAGGTTTACGTTCAGGTTGATCGAGATCCTAAAGTTGCCTGGG

AAGGAGCGCTCAATGGTTACCTCGGCAAATATTCTGGCGTAAAAAATGTTCATTTTGCGC

CGAACAACGCCACGATTGGAAAGCCCCCACGCCATATCGACATTTTGCTTCGGAAAAAAT

TTAGCGGCGAACTTCTTGCAACGCTCGAAGGTGTGGCAATTTCAAACGGTGCAACTGGTT

GGCTTGCGCTCGCATGTGTAAACCTTCTCTTGAAGGCAAGACAAGATATTGATGTCTTCT

TGTTTGGGGCAGGAAAAGTCACCGAGGCTGTGATTCTTGCCCTCAATCATGGTGCGGCTG

CAAAAATCAAAACCATGGCAGTGCTCAGCCAAGCTCCAATTGAAGGTGGGGTTCCAGCTC

GATGCCATCATTGATCGGAAGGTTATACCAAAAGCCAAACTCGTCATAATAGCAACAAAT

TCTGAGGAGCTCGGTACTCGAGGCCGACGAAATTGCGCCAAATGCAGTAACCATATCCCT

CGGAAAAGATGAATTGCCCGCCGCCTACTTCGATCGCCTTTTGAACGCAGAGGGTCTGAT

TATTGGCGACGATCTGGATGCGATCGAATCGCGCAATGTCAATTCCCTGGTGCTTTACTA

CTCGAAACGTGATTTGAAGCTGACAGAACATGGACGGGATCATTGGATAAAGAACTACGC

CAATGTCCTTGCTGATCCAGCTCTCATGGAGGAACTTAAGACATGAGAGGGGCCAGCCAA

CTTTTCATCGGTTGGCCTTGCCAGCCTAGACTTGGCGATGGCCGGCCGACTCTACGAAAC

TCTTACTGCGAAACTCTCCCACCCCCAGTAGACAAAACCCTCTTAGGATTGCGCTCTTCA

GAAGGTGCGCCTCCCCAGTCACAACCGGAAAGTCACTCAACTTGGCTCAAGTACCCTACT

TAGTTTGTCTCTCTGTTGTGTTTAGTATCAGCAACAATAACAAATATTGTAAATAACATA

TATTTATATGGTAATTATATGGCATGTAAGCGTGACAACGATTATGTTTTCCATAGTTTG

ATAAACTCAACACCCGAAAAAGCCGGTGACCAAAGGGTTGGGACCTCTTTGCACGAACTC

CTATGGCTGCCAAACTATCTACAGAGCTTTGACGCAGCATCTGATCGCCACACAATCCTC

GCGGACGTGGATGGCGTGGCTACATGCAAGCAGTTTGACTGAGAGTTTTCGAGTGACTAT

TTTAAAAGGACAATATTTCGTCCCAAGCGATAAAACTCGCTCACATGACTAAGTCGTTGC

TTTAATTCAACTGCGTGAAAAGATTTAAAGGGCAGAACTGTTTGTGTTTCGTATCCAGTT

CACCGGCAATTATTAGCTACGCATGAAGCTCATTATCGTCGATTCTTTACCCAAACACTG

ATGCCATTCCAAATGAGTTGGGCCAAAGATGTACTCATGTCGGACAGGCGCGATTTGGAC

TTCGTGATCCTCAAGTCTATCGCAGTGCTGCTTTATTACAGGCTATGCTTCCACCCTCCA

TTATTTGGTCAACTGGACCTAGCTTTTGACAAGAATAGGCCAGCGTTCATCTATCTATCT

TTCAAAAACATGCAGTTGCTGCTGCGTAAAGGATGTAACATCGGATTTGGTGTGGAGACG

GTGCTCGCTACAACTATACTGCCTTACTGTAGTGATCTGTCCCGCCAAGCGATGAGGGGA

GCGGCCTGCCTCTCGTGAGCGTCAATCCATACGGTTGCATGTTCGTTTGTTTCAAAGGCA

TTCCCAAGAAGGGTTTCCAGCTTGACAACTTTCTCATCGGTGGGTGTGAACTCGCCGGTT

AGTGAGTCACCGCCCATTTTGGGAACTACCAGGGGGGTGCCAACGACTTCCTCCGTCGAC

ATCTCAGACCAATTTCAATTGCCCAACTGCACCGTTCTTTCTATGTTGAAGTCATGGGCG

ACTATGAAATCTCCATCTTTGGTCGGTACTACCTCGATCTCGACCATATTGAAAAGCCTG

AAAGATTTGCAAACTGCCCCTACACTGTTTTCCGGCAAGTGTGGCCCATGGAATCCACGG

TGAACTATCAATTGGCGGTCGCTTGAAGTCTTTCGAGCGATAAGCCAATTGGCGAGGCAG

TGTTTCGCGATCGCATCTTTAAGAGGAAAGTCCGATGCAAATCTCCATGTAGACCCAGTC

GTTTCGGCCAAGGCGGCCTCTAAATCAACTTGAGCAGCAGTTGTGTTAACGGAATCTGTT

TTGACTGCCATTCTGTAAGAAATGTCAGCAAAGGAAAAGCTAGTTAGAGAAGGAGAGCTT

GCTCAATAGTGTGTCTATTCTCAAATGAACTATACTAGCGCAATGAGTTGGTGTTGTTCG

CTGATGGAAAAAAGGAAACGCATCCCCCTCTTCTACAAAATTCTGTGGGCCCGGAGGGAC

TCCGTATGAAAGTGCACAGCATGTGGACGCATCACCATGCAATTAGCAAGCTTAGCCAAT

GTCGTGTCTGTCAGAGTGTGCAGCATGTGAGCCAAGCGCAATTACAATAAAAAAATAATA

TAAAAACTCTGCCGAGGTCCGAACCGTCTCAAATGCTTTGAGGGCCATCCAAGCCAAGCG

CACCTAACGCCTAGCACACCATTGTTGCGCCGCTCGAGCATTAATGGTTCGGGACCTGGT

GTCATCTGGAAACCTATGAAGCATGGACACGGCAAAATCAGCGTCGTGGCGGTGTCAGAC

ACGAGGGACACGTCGGGGASMCGWMCAGGAC

>ss2440_1

AAGCCTCTCCAAATTTCCCTCACCTTGAAGTATCAATTTGATCTAGAAGCCAATCCAAAC

TCATTGAAAGGCCTGTCATCAACCTGTCAACACGGTTCAATGTTTGCTCGAGTGGCGCAG

CAATGGTGTGCTAGGCGTTAGGTGCGCCTGGCTGGGATGGCCCTCAAAGCATTTGAGACA

GTTCGGACCTCGGCAGAGATTTTATATTATTTTTTTGTTGTAATCGCGTTTGGCCCACAT

GCTGCACACTCCGACAGACACGGCATTGGCTAAACCCGCTAATTGCGTGGTGATGCGTCC

ACGTGCTGCGCACTTTCATGCGGAGTCCCCCCGAGCCCGTAGAATTTTGTAGAAGAGGGG

GACGCGTTTCCTTTTTCCCATCAGCAAACAACACCAACTCACTGTGCTAGTATAGTTCAT

CTGAGAACAGACACACTGCTGAGCAAGCTCTCCTTCTCCAACTAGCTTTTTCTTTGCTGA

CATTTCCTACAAAATGGCAGCAAAACAGATTCCGTTAACACAACTGCTGCTCAGGTTGAT

TTAGAGGCCGCCTTGGCCGAAGCGACCGGGTCTACATGGAGATTTGCATCAAACTTTCCT

CTTAAAGATGCGATCGCAAAACACTGCCTCGCCAATTGGCTTATCGCTCGAAAGACTTCA

AGCAACCACCAATTGATAGTTCACCGTGAATTCCATGGGCCACACCTGCCGGAAAACAGC

GTAGGGGCAGTCTGCAAATCTCTCAGACTTTTTAATATGGTCGAGATCAACGTAGTACCG

ACCAAAGATAGAGATTTCATAGTCGTCCACGACTTCAACACAAAAAGAACGACGCAGTTA

GGCAATTGAAATTGGTCTGATATGTCGACGAAAGAGGCCGTTGGCACCCCCCTAGTAGTT

CCCATAATGGGCGGTGACTCCCTAACCAGCGAGTTCACACCCACCGATGAGAAAGTTCTC

AAGCAAGAAACCCTTCTCAGGAATGCCTTTGAAACAAACAAACAGGCAACCATACGGATT

GACGCTCACGAGAGGCAGGCCGCTCCCCTCGTCGCTTGGCTGAGCCATAGGCCGGCCTAT

CACGACAAGACAATCGTTATCTTTCATACATATGGATACAACAATGGACACAAGTTCGCC

TTGGCGGTCGATCAAGCCGACGCTACAGAAAATTGGCGCGAAACTGTAGCGCTAATGCCA

AACATTCTGCCAAATGAACTCTCTAGGCTCGCGAAGCAATATCCACCAAATATCACGTCG

CATTTTTACGACCTCGTTGAGGCCGGCAAGAATTGGTTCGCGTCTGTCTTCTCCCAGAAT

ATGATGGTGGACGTCACTTTCCTCAAGATTGCGGGCACTGGCGAAGGACAGACTAACAAA

GTCGTTGATCCAAATGGAAATGAGGTTCTGCATCCTAAAGAGAAGAAGGAATATCTCCGC

GATTCAGCTTCAATTGAAATCGCGAAATGAATCTGCGCCACCCATCCCGGAATCGAGATT

GGAGGCGTCACACGAACTTATGCTTATAAGAGTCGAGGACATCGCCACAAAATCGATCTT

TGGACGGGTCAACCGAAGCGGTGGCCGACCCACGCGACGAAGTATATCTGGGAAGATTTC

GCCAAGCCTGGAAATGCGCTCAGATGGGGTCAAACGATTATCTCTGATCGCATTGGCGAC

GACCTTGCAGCTTGTGTGCTTCAAAGCCGTGGAGAAAATTTTGTCAGCAGTCATGAATAT

CCCGCTGTTCATGTCGATTAGATGGGCGATCTGTGTCTTAGTCTAAGCTTCCGCTTAACG

CATCTTTGCTACTTTAATGTATTTTTTTAATGATATTATTTCAGCACATCTTAATCGCAA

GTAGATATTGTTTTTGAATCGGATTCGACTTCAAGCAGATGTACTTCTTTGTCTCTCGAT

AAATCTAATGACCCTGCGAGCCGGCCTTTCGCCTTAAACTAATTTCTGAGAGTTAGGTCG

GCGCCTCTCGTGGTTTCCATGTTCTATGGAAACGGCCTCTCGGGACTTGCCTTCCCGCAA

CTCATCGAGCTTGCCTGGCCCGCTCGGGTGGTGGCGCAAGCCATCGTTAAATCGTTCGTA

ATCCTCTACTCTCGCATAAATGAGAGGGGAATCCAAAGATGATCACTTTTCAGCTAATTG

GAATCTGCAATCCTAAAAGAGGCAAGCACTATGTGGGGTCCACAACTTATAGAATTTTGG

CCTAACCATTCTGCATAATGGATCATGACATGGGCCATTTATGGCCGCAAAAACTGGCAC

AAATTCCAACCTGTTCAGCACAATGAACAAGTTCTTCCAATTCAAATAAAGGAGGTTAAA

ATTATTCGAGTAGACATGATGTGCCGGCACGCCACTAGCCTCGCATTGATGCCACCCTTT

CTTGCGCACACTTATATAATGCTGAGCTAATAATAGAGATAAAAATAGTCAGCTTGTAAA

CTGGACCTTTACTACTGCCCCTAATCTCAAGATCAAAGCGACATATACCTCCTCTCTTTC

AAGTCTTTTTGCAACTTGTTCCAAAGCAAGCGTTCGTGAATCTGCCAGGCAGCCTTCACG

AAAATGGCTGATCAACTATGTGAGGGGCGTTGGTCCGAAGCCAATCTATCATTCGTTGGG

ATATCCATCACCCCTGAATTCATCGATGAGGCCCTCAGAGGCTTTTGCCAAAAGAGCCGT

TGGCTACAAAGAGGTTTACGTTCGGGTTGATCGAGATCCTAAAGTTGCCTGGGAAGGAGC

GCTCAATGGTTACCTCGGCAAATATTCTGGCGTCAAAAATGTTCATTTTGCGCCGAACAA

CGCCGCGATTGGAAAGCCCCCATGCCATATCGACATTTTGCTTCGGAAAAAATTTAGCGG

CGAATTTCTTGTGACGCTCGAAGGTGTGGCAATTTCAAACGGTGCAACTGGTTGGCTTGC

GCTCGCGTGCGTAAACCTTCTCTTGAAGGCAAGACAAGATATTGATGTCTTCTTGTTTGG

GGCGGGAAAAGTCGCCGAGGCTGTGATTCTTGCCCTCAATCACGGTGCGGCTGCAAAAAT

CAAAACCATGGCAGTGCTCAGCCAAGCTCCAATCGAAGGTGGGGTTCCAGCTCGATGTCG

TCATTGATCGGAAGGTTATACCAAAAGCCAAACTCGTCATAACAGCAACAAATTCCGAGG

AGCTCGCACTCGAGGCCGACGAAATTGCGCCAAATGCAGTAACCATATCCCTCGGAAAAG

ATGAATTGCTCGCCACCTACTTCGATCGCCTTTTGAACGCAGAGGGTTTGATTATCGGCG

ACGATCTGGATGCGATCGAATCGCGCAATGTCAATTCCCTGGTGCTTTACTACTCGAAAC

GTGATTTGAAGCTGACAGAACATGGAAGGGATCATTGGATAAAAAACTACGCCAATGTCC

TTGTTGATCCAGCTCTCATGGAGGAACTTAAGACATGGGAGGGGCCAGCCAACTTTTCAT

CGGTTGGCCTTGCCAGCCTAGACTTGGCGATGGCCGGCCGACTCTACGAAACTCTTACTG

CGAAACTCTCCCACCCCCAGTAGACAAGACCCTCTTAGGATTGCGCTCTTCAGAAGGTGC

GCCTCCCCAGTCACAACCGGAAAGTCACTCAACTTGGCTCAAGTACCCTACTTAGTTTGT

CTCTCTGTCGTGTTTAGTATCAACAACAATAACAAATATTGTAAACAACATATATTTATA

TGGTAATTATATGGCATGTAAGCGTGACAACGATTATGTTTTCCATGGTTTGATAAACTC

AACACCCGAAAAAGCCGGTGACCAAAGGGCTGGGACCTCTTTGCACGAACTCCTATGGCT

GCCAGACTATCTACAGAGCTTTTTCGAAAGCTGACGCAGCATCTGATCGCTACACAATCC

TCGCGGACGTGGATGGCGTGGCTACACGCAAGCAGTTTGACTGAGAGTTTTCGAGTGACT

ATTTTAAAAGGACGATATTTCGTCCCAAGCAATAAAACTCGCTCACATGACTAAGTCGTT

GCTTTAATTCAGCTGCGTGAAAAGATTTAAAGGGCAGAACTGTTTGTGTTTCGTATCCAG

TTCACCGGCAATTATTGGCTACGCATGAAGCTCATTATCGTCGATTCTTTACCCAAACAC

TGATGTCATTCCAAATGAGTTGGGCCAAAGATGTACTCATGTCGGACAGGCGCGATTTGG

ACTTCGTGATCCTCAAGTCTATCGCAGTGCTGCTTTATTACAGGCTATGCTTCCACCCTC

CATTATTTGGTCAACTGGACCTGGCTTTTGACAAGAATAGGCCAGCGTTCATCTATCTAT

CTTTCAAAAACATGCAGTTGCTGTTGCGTAAAGGATGTAACATCGGATTTGGTGTGGAGA

CGGTGCTTGCTACGACTATACTGCCTTACTGTAGTGATCTGTCCTGCCAAGCGACGAGGG

GAGCAGCCTGCCTCTCGTGAGCGTCAATCCATACAGTTGCATGTTCGTTTGTTTCAAAGG

CATTCCCAAGAAGGGTTTCCAGCTTGACAACTTTCTCATCGGTGGGTGTGAACTAGCCGG

TTAGTGAGTCACCGCCCATTTTGGGAACTACCAGGGGGTGCCAACGACCTCCTCCGTCGA

CATCTCAGACCAATTTCAATTGCTCAACTGCGCCGTTCTTTCTATGTTGAAGTCATGGGC

GACTATGAAATCTCCATCTTTGGTCGGTACTACCTCGATCTCGACCATATTGAAAAGCCT

GAGAGATTTGCAAATTGCCCCTACACTGTTTTCCGGCAAGTGTGGCCCATGGAATCCACG

GTGAACTATCAATTGGCGGTCGCTTGAAGTCTTTCGAGCGATAAGCCAATTGGCGAGGCA

GTGTTTCGCGATCGCATCTTTAAGAGGAAAGTCCGATGCAAATCTCCATGTAGACCCGGT

TGCTTCGGCCAAGGCGGCCTCTAAACCAACTTGAGCAGCAGTTGTGTTAACGGAATCTAT

TTTGACTACCATTTTGTAAGAAATGTCAGCAAAGGAAAAGCTAGTTAGAGAAGGAGAGCT

TGCTCAATAGTGTGTCTATTCTCATATGAACTATACTAGCGCAATGAGTTAGTGTTGTTC

GCTGATGGGAAAAAGGAAATGCATCCCCCTCTTCTACAAAATTCTGTGGGCCCGGAGGGA

CTCCGTATGAAAGTGCACAGCATGTGGACGCATCACCATGCAATTAGCGAGCTTAGCCAA

TGTCGTGTCTGTCAGAGTGTGCAGCATGTGGGCCAAGCGCAATTACAATAAAAAAATAAT

ATAAAAACTCTGCCGAGGTCTGAACCGTCTCAAATGCTTTGAGGGCCATCCAAGCCAAGC

GCACCTAACGCCTAGCACACCATTGTTGCGCCGCTCGAGCATTAATGGTCCGGGACCTGG

TGTCATCTGGAAACCTATGAAGCATGGACACGGCAAAATCAGCGCCGTGGCGGTGTCAGA

CACGGAGACACGTCGGGGACACGTACggg

>ss2440_2

caaatttccctcaccttgaagtatcaATTTGATCTAGAAGCCAATCCAAACTCATTGAAA

GGCCTGTCATCAACCGGTCAACACGGTTCAATGTTTGCTCGCGTGGCACAGCAATGGTGT

GCTAGGCGTTAGGTGCGCCTGGCTTGGATGGCCCTCAAAGCATTTGAGGCAGTTCGGACC

TCGGCAGAGATTTTATATTATTTTTTTGTTGTAATCGCGCTTGGCCCACATGCTGCACAC

TCCGATAGACACGGCATTGGCTAAACCCGCTAATTGCGTGGTGATGCGTCCACGTGCTGC

GCACTTTCAGGCGGAGTCCCCCCGATTCCATAGAATAAGAGGGGGACGCGTTTCCTTTTT

CCCATCAGCAAACAACACCAACTCACTGTGCTAGTATAGTTCATCTGAGAACAGACACAC

TGCTGAGCAAGATCTCCTTCTCCAACTAGCTTTTTCTTTGCTGACATTTCCTACAGAATG

GCAGCAAAACAGATTCCGTTAACACAACTACTGCTCAGGTTGATTTAGAGGCCGCCTTGG

CCGAAGCGATCGGGTCTACATGGAGATTTGCATCAGACTTTCCTCTTAAAAATGCAATCG

CGAAACACTGCCTCGCCAATTGGCTTATCGCTCGAAAGACTTCAAGCAACCACCAATTGA

TAGTTCACCGTGAATTCCATGGGCCACACCTGCCGGAAAACAGCGTAGGGGCAGTCTGCA

AATCTCTCAGACTTTTTAATATGGTCGAGATCAACGTAGTACCGACCAAAGATAAAGATT

TCATAGTCGTCCACGACTTCAACACAGAAAGAACGACGCAGTTAGGCAATTGAAATTGGT

TTGATATGTCGACGAAAGAGGCTGTTGGCACCCCCCTGGTAGTTCCCATAATGGGCGGTG

ACTCCCTAACCAGCGAGTTCACACCCACCGATGAGAAAGTTGTCAAGCAAGAAACCCTTC

TCAAGAATGCCTTTGAAACAAACAAACAGGCAACCATACGGATTGACGCTCACGAGAGGC

AGGCCGCTCCCCTCGTCGCTTGGCTGAGCCATAGGCCGGCCTATCATGACAAGACAATCG

TTATCTTTCATACATATGGATACAACAATGGACACGAGTTCGCCTTGGCGGTCAATCAAG

CCGGCGCTACAAAAAATTGGCGCGAAACTGTAGCGCTAATGCCAAACATTCTGCCGAATG

AACTCTCTAGGCTCGCGAAGCAATATCCACCAAATTGCACGTCGCATTTTTACGACCTCG

TTGAGGCCAGCAAGAATTGGTTCGCATCTGTCTTCTCCCAGAATATGATGGTGGTCGTCA

CTTTCCTCAAGATTGCGGGCAATGGCGAAGGACAGACTAACAAAGTCGTTGATCCAAATG

GAAATGAGGTTCTGCATCCTAAAGAGAAGGAGGAATATCTCCGCGATTCAGCTTCAATCG

AAATCGCGAAATGAATCTGCGCCACCCATCCCGGAATCGAGATTGGAGGCGTCACACGAA

CTTATGCTTATAAGAGTCGAGGACATCGCTACAAAATCAATCTTTGGACGGGTCAACCGA

AGCGGTGGCCGACCCACGCGACAAAGTACATCTGGGAAGATTTCGCCAAGCCTGGAAATG

CGCTCAGATGGGGTCAAACGATTATCTCTGATCGCATTGGTGACGACCTTGCAGCTTGTG

TGCTTCAAAGCTGTGGAGAAAATTTTGTCAGCAGTCATGAATATCTTGCTGTTCATGTCG

ATTAGATGGGCGATCTGTGTCTTAGTCTAAGCTTCCGCTTAACGCATCTTTGCTACTTTA

ATGTATTTTTTTAATGATATTGTTGGAGTTTTGAGCACATCTTAATCGCAAGTAGATATT

TTTTTTGAATCGGATTTGACTTCAAGCAGATGTACTTCTTTGTCTCTCGATAAATCTAAT

GACCCTGCGACCGGCCTCTCGCCTTAAACTAATTTCTGAGAGTTAGGTCGCCGCCTCTCG

TGGTTTCCATGTTCTATGGAAACGGCCGCTCGGGACTTGCCTTCCCGCAACTCATCGAGC

TTGCCTGGCCCGCTCGGGTGGTGGCACAAGCCATCGTTAAATCGTTCGTAATCCTCTACT

CTCGCATAAATGAGAGGGGAATCCAAAGATGATCACTTTTCAGCTAATTGGAATCTGCAA

TCCCAAAAGAGGCAAGCACTATGTGGGGTCCACAACTTATAGAATTTTGGCCTAACCATT

CTGCATAATGGATCATGACATGGGCCATTTATGGCCGTAAAAACTGGCACAAATTCCAAC

CTATTCAGCACAATGAACAAGTTCTTCCAATTGGAATAAAGGAGGTTAAAATTATTCGAG

TAGACATGACGTGCTGGCACGCCACTAGCCTCGCATTGATGCCACTCTTTCTTGCGCACA

CTTATATAATGCTGAGCTAATAATAGAGATAAAAATAGTCAGCTTGTAAACTGGACCTTT

ATTACTGCCCCTAATCTCAAGATCAAAGCGACATATACCTCTTCTCTTTCAAGTCTTTTT

GCAACTTGTTCCAAAGCAAGCGTTTGTGAATCTGCCAGGCAGCCTTCACGAAAATGGCTG

ATCAACTGTGTGAGGGGCGTTGCTCCGAAGCCAATCTATCATTCGTTGGGATATCCATCA

CCCCTGAATTCATCGATGAGGCCCTCAGAGGCTTTTGGGAGTCTTTTGCCAAAAGAGCCG

TTGGCTACAAAGAGGTTTACGTTCAGGTTGATCGAGATCCTAAAGTTGCCTGGGAAGGAG

CGCTCAATGGTTACCTCGGCAAATATTCTGGCGTAAAAAATGTTCATTTTGCGCCGAACA

ACGCCACGATTGGAAAGCCCCCACGCCATATCGACATTTTGCTTCGGAAAAAATTTAGCG

GCGAACGTCTTGCGACGCTCGAAGGTGTGGCAATTTCAAACGGTGCAACTGGTTGGCTTG

CGCTCGCATGTGTAAACCTTCTCTTGAAGGCAAGACAAGATATTGATGTCTTCTTGTTTG

GGGCAGGAAAAGTTGCCGAGGCTGTGATTCTTGCCCTCAATCATGGTGCGGCTGCAAAAA

TCAAAACCATGGCAGTGCTCAGCCAAGCTCCAATTGAAGGTGGGGTTCCAGCTCGATGCC

ATCATTGATCGGAAGGTTATACCAAAAGCCAAACTCGTCATAACAGCAACAAATTCCGAG

GAGCTCGGTACTCGAGGCCGACGAAATTGCGCCAAATGCAGTAACCATATCCCTCGGAAA

AGATGAATTGCCCGCCGCCTACTTCGATCGCCTTTTGAACGCAGAGGGTCTGATTATTGG

CGACGATCTGGATGCGATCGAATCGCACAATGTCAATTCCCTGGTGCTTTACTACTCGAA

ACGTGATTTGAAGCTGACAGAACATGGAAGGGATCATTGGATAAAGAACTACGCCAATGT

CCTTGCTGATCCAGCTCTCATGGAGGAACTTAAGACATGAGAGGGGCCAGCCAACTTTTC

ATCGGTTGGCCTTGCCAGCCTAGACTTGGCGATGGCCGGCCGACTCTACGAAACTCTTAC

TGCGAAACTCTCCCACCCCCAGTAGACAAAACCCTCTTAGGATTGCGCTCTTCAGAAGGT

GCGCCTCCCCAGTCACAACCGGAAAGTCACTCAACTTGGCTCAAGTACCCTACTTAGTTT

GTCTCTCTGTCGTGTTTAGTATCAGCAACAATAACAAATATTGTAAATAACATATATTTA

TATGGTAATTATATGGCATGTAAGCGTGACAACGATTATGTTTTCCATAGTTTGATAAAC

TCAACACCCGAAAAAGCCGGTGACCAAAGGGTTGGGACCTCTTTGCACGAACTCCTATGG

CTGCCAGACTATCTACAGAGCTTTTTCGGAAGCTGACGCAGCATCTGATCGCCACACAAT

CCTCGCGGACGTGGATGGCGTGGCTACATGCAAGCAGTTTGACTGAGAGTTTTCGAGTGA

CTGTTTTAAAAGGACAATATTTCGTCCCAAGCGATAAAACTCGCTCACATGACTAAGTCG

TTGCTTTAATTCAACTGCGTGAAAAGATTTAAAGGGCAGAACTGTTTGTGTTTCGTATCC

AGTTCACCGGCAATTATTGGCTACGCATAAAGCTCATTATCGTCGATTCTTTACCCAAAC

ACTGATGCCATTCCAAATGCGTTGGGCCAAAGATGTACTCATGTCGGACAGGCGCAATTT

GGACTTCGTGATCCTCAAGTCTATCGCAGTGCTGCTTTATTACAGGCTATGCTTCCACCC

TCCATTATTTGGTCAACTGGACCTAGCTTTTGACAAGAATAGGCCAGCGTTCATCTATCT

ATCTTTCAAAAACATGCAGTTGCTGCTGCGTAAAGGATGTAACATCGGATTTGGTGTGGA

GACGGTGCTCGCTACGACTATACTGCCTTACTGTAGTGATCTGTCCCGCCAAGCGATGAG

GGGAGCGGCCTGCCTCTCGTGAGCGTCAATCCATACGGTTGCATGTTCGTTTGTTTCAAA

AGCATTCCCAAGAAGGGTTTCCAGCTTGACAACTTTCTCATCGGTGGGTGTGAACTCGCC

GGTTAGTGAGTCACCGCCCATTTTGGGAACTACCAGGGGGGTGCCAACGACCTCCTCCGT

CGACATCTCAGACCAATTTCAATTGCCCAACTGCACCGTTCTTTCTATGTTGAAGTCATG

GGCGACTATGAAATCTCCATCTTTGGTCGGTACTACCTCGATCTCGACCATATTGAAAAG

CCTGAAAGATTTGCAAACTGCCCCTACACTGTTTTCCGGCAAGTGTGGCCCATGGAATCC

ACGGTGAACTATCAATTGGCGGTCGCTTGTAGTCTTTCGAGCGATAAGCCAATTGGCGAG

GCAGTGTTTCGCGATCGCATCTTTAAGAGGAAAGTCCGATGCAAATCTCCATGTAGACCC

AGTCGTTTCGGCCAAGGCGGCCTCTAAATCAACTTGAGCAGCAGTTGTGTTAACGGAATC

TGTTTTGACTGCCATTCTGTAAGAAATGTCAGCAAAGGAAAAGCTAGTTAGAGAAGGAGA

GCTTGCTCAATAGTGTGTCTATTCTCAAATGAACTATACTAGCGCAATGAGTTGGTGTTG

TTCGCTGATGGGAAAAAGGAAACGCATCCCCCTCTTCTACAAAATTCTGTGGGCCCGGAG

GGACTCCGTATGAAAGTGCACAGCATGTGGACGCATCACCATGCAATTAGCAAGCTTAGC

CAATGTCGTGTCTGTCAGAGTGTGCAGCATGTGAGCCAAGCGCAATTACAATAAAAAAAT

AATATAAAAACTCTGCCGAGGTCCGAACCGTCTCAAATGCTTTGAGGGCCATCCAAGCCA

AGCGCACCTAACGCCTAGCACACCATTGTTGCGCCGCTCGAGCATTAATGGTTCGGGACC

TGGTGTCATCTGGAAACCTATGAAGCATAGACACGGCAAAATCAGCGCCGTGGCGGTGTC

AGACAcgga

>sa7883_un

AATTTCCCTCACCTTGAAGTATCAATTTGATCTAGAAGCCAATCCAAACTCATTGAAAGG

CCTGTCATCAACCTGTCAACAYGGTTCAATGTTTGCTCGAGTGGCGCAGCAATGGTGTGC

TAGGCGTTAGGTGCGCCTGGCTTGGATGGCCCTCAAAGCATTTGAGACAGTTCGGACCTC

GGCAGAGATTTTATATTATTTTTTTGTTGTAATCGCGCTTGGCCCACATGCTGCACACTC

CGACAGACACGGCATTGGCWAAACCCGCTAATTGCGTGGTGATGCGTCCACGTGCTGCGC

ACTTTCATGCGGAGTCCCCCCGAGCCCGTAGAATTTTGTAGAAGAGGGGGACGCGTTTCC

TTTTTCCCATCAGCAAGCAACACCAACTCACTGTGYTAGTATAGTTCATCTGAGAACAGA

CGCACTGCTGAGCAAGCTCTCCTTCTCCAACTAGCTTTTTCTTTGCTGACATTTCCTACA

GAATGGCAGCCAAAACAGATTCCGTTAACACAACTGCTGCTGAGGTTGATTTAGAGGCCG

CCTTGGCCGAAGCGACCGGGTCTACATGGAAATTTGCATCAGACTTTCCTCTTAAAGATG

CGATTGCGAAACACTGCCTCGCCAACTGGCTTATCGCTCGAAAGACTTCAAGCAACCACC

AATTGATAGTTCACCGTGAATTCCATGGGACACACCTGTCGGAAAACAGCGTAGGGGCAG

TCTACAAATCTCTTAGACTTTTTAATATGGTCGAGATCAACGTAGTACCGACCAAAGATA

GAGATTTCATAGTCGTCCACGACTTCAACACAGAAAGAACGACGTAGTTAGGCAATTGAA

ATTGGTCTGATATGTTGACGAAAGAGGCCGTTGGCACCCCCCTGGTAGTTCCCATAATGG

GCGGTGACTCCCTAACCAGCGAGTTCACACCCACCGATGAGAAAGTTGTCAAGCAAGAAA

CCCTTCTCAGGAATGCCTTTGAAACAAACAAACAGGCAACCATACGGATTGAYGCTCACA

AGAGGCAGGCCGCTCCCCTCGTCGCTTGGCTGAGCCATAGGCCGGCCTATCACGACAAGA

CAATCGTTATCTTTCATACATATGGATACAACAATGGACACGAGTTTGCCTTGGCGGTCG

ATCAAGCCGGCGCTACAGAAAATTGGCGCGAAATTGTAGCGCTAATGCCAAACATTCTGC

CGAATGAACTCTCTAGGCTCGCGAAGCAATATCCACCAAATCGCACGTCGCATTTTTACG

ACCTCGTTGAGGCCGGCAAGAATTGGTTCGTGTCTGTCTTCTCCCAGAATATGATGGTGG

TCGTCACTTTCCTCAAGATTGCGGGCACTGGCGAAGGACAGACTAACAAAGTCGTTGATC

CAAATGGAAATGAGGTTCTGCATCCTAAAGAGAAGGAGGAATATCTCCGCGATTCAGCTT

CAATTGAAATCACGAAATGAATCTGCGCCACCCATCCCGGAATCGAGATTGGAGGCGTCA

CACGAACTTATGCTTATAAGAGTTGAAGACATCGCTACAAAATCGATCTTTGGACGGGTC

AACCGAAGCAGTGGCCGACCCACGCGACGAAGTATATCTGGGAAGATTTCGCCAAGCCTG

GAAATGCGCTCAGATGGGATCAAACGATTATCTCTGATTGCATTGGCGACAACCTTGCAG

CTTGTGTGCTTCAAAGCCGTGGAGAAAATTTTGTCAGCAGTCATGAATATCCCGCTGTTC

ATGTCGATTAGATGGGCAATCTTTGTCTTAGTCTAAGCTTCCGCTTAACGCATCTTTGCT

ACTTTAATGTATTTTTTTAATGATATTGTTGGAGTTTTCAGCACATCTTAATTGCAAGTA

GATTTTTTTTTTTTGAATCGGATTCGACTTCAAGCAGATGTACTTCTTTGTCTCTCGATA

AATCTAATGACCCTGCGAGCCGGCCTCTCGCCTTAAACTAATTTCTGAGAGTTAGGTCGG

CGCCTCTCGTGGTTTCCATGTTCTATGGAAAYGGCCTCTCGGGACTTGCCTTCCCRCAAC

TCATCGAGCTTTCTTGGCCCGCTCGGGTGGTGGCGCAAGCCATCGTTAAATCGTTCGTAA

TCCTCTACTCTCGCATAAATGAGAGGGGAATCCAAAGATGATCACTTTTCAACTAATTGG

AATCTGCAATCCCAAAAGAGGCAAGCACTATGTGGGGTCCACAACTTATAGAATTTTGGC

CTAACCATTCTGCATAATGGATCATGACATRGGCCATTTATGGCCGCAAAAACTGGCACA

AATTCCAACCTGTTCAGCACAATGAACAAGTTCTTCCAATTGGAATAAAGGAGGTTAAAA

TTATTCGAGTAGACATGACGTGCCGGCACGCCACTAGCCTCGCATTGATGCCACCCTTTC

TTGCGTACACTTATATAATGCTGAGCTAATAAYAGAGATAAAAATAGTCAGCCTGTAAAC

TGGACCTTTACTACTGCCCCTAATCTTAAGATCAAAGCGACATATACCTCCTCTCTTTCA

AGTTTTTTTGCAACTTGTTCCACAGCAAGCGTTCGTGAATCTGCCAGGCAGCCTTCACGA

AAATGGCTGATCAACTGTGTGAGGGGCGTTGGTCCGAAGCCGATCTATCATTCGTTGGGA

TATCYATCACCCCTGAATTCAGGGCTGTGCAACGGATCGCCTGACCCACCAAATCCGCCC

GACCCGACCCGACACCTAATGAATGGATGCGTGACCCCTAGCGGGCGGGTGCGGGCCTCG

TTTTTCAATTTTCTCGGGTTCGGGTCGGGTGGCGGGTGATCCGCCATTTTACCCAGCAGA

CCCGACCCACCCGTGCCTTCTTTTTTTTTTTTTTTCCTTACGCCTCCCTGCCTCAGGCCC

CTNNNATATATGTTGAAAATTTGTAAAATTTCAAACCGCCAAACCGATTCGTATCCATCC

GCCAATCCGCCAAATCCGCATCCGCCGGATTGGCGGACGGATGCGGATTGATTTTCTCCA

ATCCGCCAATCTGGCGGACAGATAGCGGATTAGAGGTTTCGTGGCCGCAACCGATCCGCA

CTCTACCTAAATTCATCGATGAGGCCCTCAGAGGATTTTGGGAGTCTTTTGCCAAAAGAG

CCGTTGGCTACAAAGAGGTTTACGTTCAGGTTGATCGAGATCCTAAAGTTGCCTGGGAAG

GAGCGCTCAATGGTTACCTCGGCAAATATTCTGGCGTCAAAAATGTTCATTTCGCGCCGA

ACAACGCCGCGATTGGAAAGCCCCCACGCCATATTGACATTTTGCTACGAAAAAAATTTA

GCGGTGAACTTCTTGYGACGCTCGAAGGTGTGGCAATTTCAAACGGTGCAACTGGTTGGC

TTGCGCTCGCATGCGTAAACCTTCTCTTGAAGGCAAGACAAGATATTGATGTCTTCTTGT

TTGGGGCGGGAAAAGTCGCCGAGGCTGTGATTCTTGCCCTCAATCATGGTGCGGCTGCAA

AAATCAAAACCATGGCAGTGCTCAGCCAAGCTCCAATCGAAGGTGGGGTTCCAGCTCGAT

GCCGTCATTGATCGGAAGGTTATACCAAAAGCCAAACTCGTCATAACAGCAACAAATTCC

GAGGAGCTCGTACTCAAGGCCGACGAAATTGCGCCAAATGCAATAACCATATCCCTCGGA

AAAGATGAATTGCCCGCCGCCTACTTCGATCTCCTTTTGAACGCAGAGGGTCTGATTATC

GGTGACGATATGGATGCGATCGAATCACGCAATGTCAATTCCCTGGTGCTTTACTACTCG

AAACGTGATTTGAAGCTGACTAAACATGGAATGGATCATTGGATAAAGAACTACGCCAAT

GTCCTTGCCGATCCAGCTCTCATGGAGGAACTTAAGACATGGGAGGGGCCAGCCAACTTT

TCATCGGTTGGCCTTGCCAGCCTAGACTTGGCGATGGCTGGCCGACTCTACGAAACTCTT

ACTGCGAAACTCTCCCACCCCCAGTAGACAACCGGAAAGTCACTCAACTTGGCTCAAGTA

CCCTACTTAGTTTGTCTCTCTGTCGTGTTTAGTATCAGCAACAATAACAAATATTGTAAA

TAACATATATTTATATGGTAATTATATGGCATGTAAGCGTGACAACGATTATGTTTTCCA

TAGTTTGATAAACTCAACACCCGAAAAAGCCGGTGACCAAAGGGTTGGGACCTCTTTGCA

CGAACTCCTATGGCTCCCAGACTATCTACAGAGCTTTTTCGGAAGCTGACGCAGCATCTG

ATCGCCACACAATCCTCGCGGACGTGGATGGTGTGGCTACACGCAAGCAGTTTGACTGAG

AGTTTTCGAGTGACTATTTTAAAAGGACAATATTTCGTCCCAAGCAATAAAACTCGCTCA

CATGACTAAGTCGTTGCTTTAATTCAGCTGTGTGAAAAGATTTAAAGGGCAGAACTGTTT

GTGTTTCGTATCCAGTTCACCGACAATTATTGGCTACGCATGAAGCTCATTATCGTCGAT

TCTTTACCCAAACACTGATGCCATTCCAAATGAGTTGGGCCAAAGATGTACTCATGTCGG

ACAGGCGCGATTTGGACTTCATGATCCTCAAGTCTATCGCAGTGCTGCTTTATTACAGGC

TATGCTTCCACCCTCCATTATTTGGTCAACTGGACCTGGCTTTTGACAAGAATAGGCCAG

CGTTCATCTATCTATCTTTCAAAAACATGCAGTTGCTGCTGCGTGAAGGATGTAACACGG

ATTTGGTGTGGAGATGATGCTCGCTACGACTATACTGCCTTACTGTAGTGATCTGTCCCA

CCAAGCGACGAGGGGAGCGGCCTGCCTCTCGTGAGCGTCAATCCATACGGTTGCATGTTC

GTTTGTTTCAAAGGCATTCCCAAGAAGGGTTTCCAGCTTGACAACTTTCTCATCGGTGGG

TGTGAACTCACCGGTTAGTGAGTCACCGCCCATTTTGAGAACTACCAGGGGGGGTGCCAA

CGACCTCCTCCGTCGACATCTCAGACCAATTTCAATTGCCCAACTGCGCCGTTCTTTCTA

TGTTGAAGTCATGGGCGACTATGAAATCTCCATCTTTGGTCGGTACTACCTCGATCTCGA

CCATATTGAAAAGCCTGAGAGATTTGCAAACTGCCCCTACACTGTTTTCCGGCAAGTGTG

GCCCATGGAATCCACGGTGAACTATCAATTGGCGGTCGCTTGAAGTCTTTCGAGCAATAA

GCCAATTGGCGAGGCAGTGTTTTGCGATCGCATCTTTAAGARGAAAGTCCGATGCAAATC

TCCATGTAGACCCGGTCGCTTCGGCCAAGGCGGCCTCTAAATCAACTTGAGCAGCAGTTG

TGTTAACGGAATCTGTTTTGACTACCTTTCTGTAGGAAATGTCAGCAAAGGAAAAGCTAG

TTAGAGAAGGAGAGCTTGCTCAATAGTGTGTCTATTCTCAGATGAACTATACTAGCGCAA

TGAGTTGGTGTTGTTCGCTGATGGGAAAAAAGAAACGCATCCCCCTCTTCTACAAAATTC

TGTGGGCCTGGAGGGACTCCGTATGAAAGTGCACAGCATGTGGACGCATCGCCATGCAAT

TAGCGAGCTTAGCCAATGCCGTGTCTGTCAGAGTGTGCAGCATGTGGGCCAAGCGCAATT

ACAATAAAAAAATAATATAAAAACTCTGCCGAGGTCCGAACCGTCTCAAATGCTTTGAGG

ACCATCCAAGCCAAGCGCACCTAACGCCTAGCACACCATTGTTGCGCCGCTCCAGCATTA

ATGGTCCAGGACCTGGTGTCATCTGGAAACCTATGAAGCATGGACACGGCAAAATCAGCA

CCGTGGCAATGTCAGACACGGGGACACGTCGGGGACATGTATGTCAGAGTT

>ssFDDB_Un

CATCAACCGGTCAASACGGTTCAATGTTTGCTCGCGTGGCGCAGCAATGGTGTGCTAGGC

GTTAGGTGCGCCTGGCTTGGATGGCCCTCAAAGCATTTGAGGCAGTTCGGACCTCAGCAG

AGATTTTATATTATTTTTTTGTTGTAATCGCGCTTGGCCCACATGCTGCACACTCCGATA

GACACGGCATTGGCTAAACCCGCTAATTGCGTGGTGATGCGTCCACGTGCTGCGCACTTT

CATGCGGAGTCCCCCCGAGTCCATAGAATAAGAGGGGGACGCGTTTCCTTTTTCCCATCA

GCAAACAACACCAACTCACTGTGCTAGTATAGTTCATCTGAGAACAGACACACTGCTGAG

CAAGCTCTCCTTCTCCAACTAGCTTTTTCTTTGCTGACATTTCCTACAGAATGGCAGCAA

AACAGATTCCGTTAACACAACTACTGCTCAGGTTGATTTAGAGGCCGCCTTGGCCGAAGC

GATCGGGTCTACATGGAGATTTGCATCAGACTTTCCTCTTAAAAATGCAATCGCGAAACA

CTGCCTCGCCAATTGGCTTATCGCTCGAAAGACTTCAAGCAACCACCAATTGATAGTTCA

CCGTGAATTCCATGGGCCACACCTGCCGGAAAACAGCGTAGGGGCAGTCTGCAAATCTCT

CAAACTTTTTAATATGGTCGAGATCAACGTAGTACCGACCAAAGATAAAGATTTCATAGT

CGTCCACGACTTCAACACAGAAAGAACGACGCAGTTAGGCAATTGAAATTGGTTTGATAT

GTCGACGAAAGAGGCTGTTGGCACCCCCCTGGTAGTTCCCATAATGGGCGGTGACTCCCT

AACCAGCGAGTTCACACCCACCGATGAGAAAGTTGTCAAGCAAGAAACCCTTCTCAAGAA

TGCCTTTGAAACAAACAAACAGGCAACCATACGGATTGACGCTCACGAGAGGCAGGCCGC

TCCCCTCGTCGCTTGGCTGAGCCATAGGCCGGCCTATCACGACAAGACAATCGTTATCTT

TCATACATATGGATACAACAATGGACACGAGTTCGCCTTGGCGGTCAATCAAGCCGGCGC

TACAAAAAATTGGCGCGAAACTGTAGCGCTAATGCCAAACATTCTACCGAATGAACTCTC

TAGGCTCGCGAAGCAATATCCACCAAATTGCACGTCGCATTTTTACGACCTCGTTGAGGC

CAGCAAGAATTGGTTCGCATCTGTCTTCTCCCAGAATATGATGGTGGTCGTCACTTTCCT

CAAGATTGCGGGCACTGGCGAAGGATAGACTAACAAAGTCGTTGATCCAAATGGAAATGA

GGTTCTGCATCCTAAAGAGAAGGAGGAATATCTCCGCGATTCAGCTTCAATCGAAATCGC

GAAATGAATCTGCGCCACCCATCCCGGAATCGAGATTGGAGGCGTCACACAAACTTATGC

TTATAAGAGTCGAGGACATCGCTACAAAATCAATCTTTGGACGGGTCAACCGAAGCGGTG

GCCGACCCACACGACGAAGTACATCTGGGAAGATTTCGCCAAGCCTGGAAATGTGCTTAG

ATGGGGTCAAACGATTATCTCTGATCGCATTGGTGACGACCTTGCAGCTTGTGTGCTTCA

AAGCCGTGGAGAAAATTTTGTCAGCAGTCATGAATATCCTGCTGTTCATGTCGATTAGAT

GGGCGATCTGTGTCTTAGTCTAAGCTTCCGCTTAACGCATCTTTGTTACTTTAATGTATT

TTTTTAATGATATTGTTGGAGTTTTGAGCACATCTTAATCGCAAGTAGATATTTTTTTTG

AATCGGATTCGACTTCAAGCAGATGTACTTCTTTGTCTCTCGACAAATCTAATGACCCTG

CGAGCCGGCCTCTCGCCTTAAACTAATTTCTGAGAGTTAGGTCGGCGCCTCTCGTGGTTT

CCATGTTCTATGGAAACGGCCGCTCGGGACTTGCCTTCCCGCAACTCATCGAGCTTGCCT

GGCCCGCTCGGGTGGTGGCACAAGCCATCGTTAAATCGTTCGTAATCCTCTACTCTCACA

TAAATGAGAGGGGAATCCAAAGATGATCACTTTTTAGCTAATTGGAATCTGCAATCCCAA

AAGAGGCAAGCACTATGTGGGGTCCACAACTTATAGAATTTTGGCCTAACCATTCTGCAT

AATGGATCATGACATGGGCCATTTATGGCCGCAAAAACTGGCACAAATTCCAACCTATTC

AGCACAATGAACAAGTTCTTCCAATTGGAATAAAGGAGGTTAAAATTATTCGAGTAGACA

TGACGTGCTGGCACGCCACTAGCCTCGCATTGATGCCACTCTTTCTTGCGCACACTTATA

TAATGCTGAGCTAATAATAGAGATAAAAATAGTCAGCTTGTAAACTGGACCTTTATTACT

GCCCCTAATCTCAAGATCAAAGCGACATATACCTCTTCTCTTTCAAGTCTTTTTGCAACT

TGTTCCAAAGCAAGCGTTTGTGAATCTGCCAGGCAGCCTTCACGAAAATGGCTGATCAAC

TGTGTGAGGGGCGTTGGTCCGAAGCCAATCTATCATTCGTTGGGATATCCATCACCCCTG

AATTCATCGATGAGGCCCTCAGAGGCTTTTGGGAGTCTTTTGCCAAAAGAGCCGTTGGCT

ACAAAGAGGTTTACGTTCAGGTTGATCGAGATCCTAAAGTTGCCTGGGAAGGAGCGCTCA

ATGGTTACCTCGGCAAATATTCTGGCGTAAAAAATGTTCATTTTGCGCCGAACAACGCCA

CGATTGGAAAGCCCCCACGCCATATCGACATTTTGCTTCGGAAAAAATTTAGCGGCGAAC

TTCTTGCGACGCTCGAAGGTGTGGCAATTTCAAACGGTGCAACTGGTTGGCTTGCGCTCG

CATGTGTAAACCTTCTCTTGAAGGCAAGACAAGATATTGATGTCTTCTTGTTTGGGGCAG

GAAAAGTCGCCGAGGCTGTGATTCTTGCCCTCAATCATGGTGCGGCTGCAAAAATCAAAA

CCATGGCAGTGCTCAATCAAGCTCCAATTGAAGGTGGGGTTCCAGCTCGATGCCGTCATT

GATCGGAAGGTTATACCAAAAGCCAAACTCGTCATAACAGCAACAAATTCCGAGGAGCTC

GGTACTCGAGGCCGACGAAATTACGCCAAATGCAGTAACCATATCCCTCGGAAAAGATGA

ATTGCCCGCCGCCTACTTCGATCGCCTTTTGAACGCAGAGGGTCTGATTATTGGCGACGA

TCTGGATGCGATCGAATCGCGCAATGTCAATTCCCTGGTGCTTTACTACTCGAAACGTGA

TTTGAAGCTGACAGAACATGGAAGGGATCATTGGATAAAGAACTACGCCAATGTCCTTGC

TGATCCAGCTCTCATGGAGGAACTTAAGACATGAGAGGGGCCAGCCAACTTTTCATCGGT

TGGCCTTGCCAGCCTAGACTTGGCGATGGCCGGCCGACTCTACGAAACTCTTACTGCGAA

ACTCTCCCACCCCCAGTAGACAAAACCCTCTTAGGATTGCGCTCTTCAGAAGGTGCGCCT

CCCCAGTCACAACCGGAAAGTCACTCAACTTGGCTCAAGTACCCTACTTAGTTTGTCTCT

CTGTCGTGTTTAGTATCAGCAACAATAACAAATATTGTAAATAACATATATTTATATGGT

AATTATATGGCATGTAAGCGTGACAACGATTATGTTTTCCATAGTTTGATAAACTCAACA

CCCGAAAAAGCCGGTGACCAAAGGGTTGGGACCTCTTTGCACGAACTCCTATGGCTGCCA

GACTATCTACAGAGCTTTTTCGGAAGCTGACGCAGCATCTGATCGCCACACAATCCTCGC

GGACGTGGATGGCGTGGCTACATGCAAGCAGTTTGACTGAGAGTTTTCGAGTGACTATTT

TAAAAGGACAATATTTCGTCCCAAGTGATAAAACTCGCTCACATGACTAAGTCGTTGCTT

TAATTCAACTGCGTGAAAAGATTTAAAGGGCAGAACTGTTTGTGTTTCGTATCCAGTTCA

CCGGCAATTATTGGCTACGCATGAAGCTCATTATCGTCGATTCTTTACCCAAACACTGAT

GCCATTCCAAATGAGTTGGGCCAAAGATGTACTCATGTCGGACAGGCGCGATTTGGACTT

CGTGATCCTCAAGTCTATCGCAGTGCTGCTTTATTACAGGCTATGCTTCCACCCTCCATT

ATTTGGTCAACTGGACCTAGCTTTTGACAAGAATAGGCCAGCGTTCATCTATCTATCTTT

CAAAAACATGCAGTTGCTGCTGCGTAAAGGATGTAACATCGGATTTGGTGTGGAGACGGT

GCTCGCTACGACTATACTGCCTTACTGTAGTGATCTGTCCCGCCAAGCGATGAGGGGAGC

GGCCTGCCTCTCGTGAGCGTCAATCCATACGGTTGCATGTTCGTTTGTTTCAAAGGCATT

CCCAAGAAGGGTTTCCAGCTTGACAACTTTCTCATCGGTGGGTGTGAACTCCCCGGTTAG

TGAGTCACCGCCCATTTTGGGAACTACCAGGGGGTGCCAACGACCTCCTCCGTCGACATC

TCAGACCAATTTCAATTGCCCAATTGCACCGTTCTTTCTATGTTGAAGTCATGGGCGACT

ATGAAATCTCCATCTTTGGTCGGTACTACCTCGATCTCGACCATATTGAAAAGCCTGAAA

GATTTGCAAACTGCCCCTACACTGTTTTCCGGCAAGTGTGGCCCATGGAATCCACGGTGA

ACTATCAATTGGCGGTCGCTTGAAGTCTTTCGAGCGATAAGCCAATTGGCGAGGCAGTGT

TTCGCGATCGCATCTTTAAGAGGAAAGTCCGATGCAAATCTCCATGTAGACCCAGTCGTT

TCGGCCAAGGCGGCCTCTAAATCAACTTGAGCAGTAGTTGTGTTAACGGAATCTGTTTTG

ACTGCCATTCTGTAAGAAATGTCAGCAAAGGAAAAGCTAGTTAGAGAAGGAGAGCTTGCT

CAATAGTGTGTCTATTCTCAAATGAACTATACTAGCGCAATGAGTTGATGTTGTTCGCTG

ATGGAAAAAAGGAAACGCATCCCCCTCTTCTACAAAATTCTGTGGGCCCGGAGGGACTCC

GTATGAAAGTGCACAGCATGTGGACGCATCACCATGCAATTAGCAAGCTTAGCCAATGTC

GTGTCTGTCAGAGTGTGCAGCATGTGTAGGAATGGCAACGGGGCGGGGCGGGGCCGGGGA

TCACCCTCCCCGTCCCCGATCCCCGATGGCAGAACTTCCCCCCGTCCCCGTCCCCATCCC

CGACGGGGAACCCACTANNNGTCTTCTCCCCGTCGGGAGTATTTTTCGGGAAAAAACCGT

TTAAATCGGGGTCGGGATCGGGGATCCCCGTCGGGCCGGTTTCAAATTGCCATCCCTAAG

CATGTGAGCCAAGCGCAATTACAATAAAAAAATAATATAAAAACTCTGCCGAGGTCCGAA

CCGTCTCAAATGCTTTGAGGGCCATCCAAGCCAAGCGCACCTAACGCCTAGCACACCATT

GTTGCGCCACTCGAGCATTAATGGTTCGGGACCTGGTGTCATCTGGAAACCTATGAAGCA

TGGACACGG

>sa7884_Un

TCTCCACATTTCCCTCACCTTGAAGTMTCAATTTGATCTAGAAGCCAATCCAAACTCATT

GAAAGGCCTGTCATCAACCGGTCAACACGGTTCAATGTTTGCTCGCGTGGCGCAGCAATG

GTGCGCTAGGCGTTAGGTGCGCCTGGCTTGGATGGCCCTCAAAGCATTTGAGGCAGTTCG

GACCTCGGCAGAGATTTTATATTATTTTTTTGTTGTAATCGCGCTTGGCCCACATGCTGC

ACACTCCGATAGACACGGCATTGGCTAAACCCGCTAATTGCGTGGTGATGCGTCCACGTG

CTGCGCACTTTCATGCGGAGTCCCCCCGAGTCCATAGAATAAGAGGGGGACGCATTTCCT

TTTTCCCATCAGCAAACAACACCAACTCACTGTGCTAGTATAGTTCATCTGAGAACAGAC

ACACTGCTGAGCAAGCTCTCCTTCTCTAACTAGCTTTTTCTTTGCTGACATTTCCTACAG

AATGGCAGCAAAACAGATTCCGTTAACACAACTACTGCTCAGGTTGATTTAGAGGCCGCC

TTGGCCGAAGCGATCGGGTCTACATGGAGATTTGCATCAGACTTTCCTCTTAAAAATGCA

ATCGCGAAACACTGCCTCGCCAATTGGCTTATCGCTCGAAAGACTTCAAGCAACCACCAA

TTGATAGTTCACCGTGAATTCCATGGGCCACACCTGCCGGAAAACAGCGTAGGGGCAGTC

TGCAAATCTCTCAGACTTTTTGATATGGTCGAGATCAACGTAGTACCGACCAAAGATAAA

GATTTCATAGTCGTCCACGACTTCAACACAGAAAGAATGACGCAGTTAAGCAATTGAAAT

TGGTTTGATATGTCGACGAAAGAGGCTGTTGGCACCCCCCTGGTAGTTCCCATAATGGGC

GGTGACTCCCTAACCAGCGAGTTCACACCCACCGATGAGAAAGTTGTCAAGCAAGAAACC

CTTCTCAAGAATGCCTTTGAAACAAACAAACAGGCAACCATACGGATTGATGCTCACGAG

AGGCAGGCCGCTCCCCTCGTCGCTTGGCTGAGCCATAGGCCGGCCTATCACGACAAGACA

ATCGTTATCTTTCATACATATGGATACAACAATGGACACGAGTTCGCCTTGGCGGTCGAT

CAAGCCGGCGCTACAAAAAATTGGCGCGAAACTGTAGCGCTAATGCCAAACATTCTGCCG

AATGAACTCTCTAGGCTCGCGAAGCAATATCCACCAAATTGCACGTCGCATTTTTACGAC

CTCGTTGAGGCCAGCAAGAATTGGTTCGCATCTGTCTTCTCCCAGAATATGATGGTGGTC

GTCACTTTCCTCAAGATTGCGGGCACTGGCGAAGGACAGACTAACAAAGTCGTTGATCCA

AATGGAAATGAGGTTTTGCATCCTAAAGAGAAGGAGGAATATCTCCGCGATTCAGCTTCA

ATCGAAATCGCGAAATGAATCTGCGCCACCCATCCCGGAATCGAGATTGGAGGCGTCACA

CGAACTTATGCTTATAAGAGTCGAGGACATCGCTACAAAATCAATCTTTGGACGGGTCAA

CCGAAGCGGTGGCCGACCCACGCGACGAAGTACATCTAGGAAGATTTCGCCAAGCCTGGA

AATGCGCTCAGATGGGGTCAAACGATTATCTCTGATCGCATTGGTGACGACCTTGCAACT

TGTGTGCTTCAAAGCCGTGGAGAAAATTTTGTCAGCAGTCATGAATATCCTGCTGTTCAT

GTCGATTAGATGGGCGATCTGTGTCTTAGTCTAAGCTTCCGCTTAACACATCTTTGCTAC

TTTAATGTATTTTTTTAATGATATTGTTGGAGTTTTGAGCACATCTTAATCGCAAGTAGA

TATTTTTTTTGAATCGGATTCGACTTCAAGCAGATGTACTTCTTTGTCTCTCGATAAATC

TAATGACCCTGCGAGCCGGCCTCTCACCTTAAACTAATTTCTGAGAGTTAGGTCGGCGCC

TCTCGTGGTTTCCATGTTCTATGGAAACGGCCGCTCGGGACTTGCCTTCCCGCAACTCAT

CGAGCTTGCCTGGCCCGCTCGGGTGGTGGCACAAGCCATCGTTAAATCGTTCGTAATCCT

CTACTCTCGCATAAATGAGAGGGGAATCCAAAGATGATCACTTTTCAGCTAATTGGAATC

TGCAATCCCAAAAGAGGCAAGCACTATGTGGGGTCCACAACTTATAGAATTTTGGCCTAA

CCATTCTGCATAATGGATCATGAGATGGGCCATTTATGGCCGCAAAAACTGGCACAAATT

CCAACCTATTCAGCACAATGAACAAGTTCTTCCAATTGGTATAAAGGAGGTTAAAATTAT

TCGAGTAGACATGACGTGCTGGCACGCCACTAGCCTCGCATTGATGCCACTCTTTCTTGC

GCACACTTATATAATGCTGAGCTAATAATAGAGATAAAAATAGTCAGCTTGTAAACTGGA

CCTTTATTACTGCCCCTAATCTCAAGATCAAAGCGACATATACCTCTTCTCTTTCAAGTC

TTTTTGCAACTTGTTCCAAAGCAAGCGTTTGTGAATCTGCCAGGCAGCCTTCACGAAAAT

GGCTGATCAACTGTGTGAGGGGCGTTGGTCCAAAGCCAATCTATCATTCGTTGGGATATC

CATCACCCCTGAATTCATTGATGAGGCCCTCAAAGGCTTTTGGGAGTCTTTTGCCAAAAG

AGCCGTTGGCTACAAAGAGGTTTACGTTCAGGTTGATCGAGATCCTAAAGTTGCCTGGGA

AGGAGCGCTCAATGGTTACCTCGGCAAATATTCTGGTGTAAAAAATGTTCATTTTGCGCC

GAACAACGCCACGATTGGAAAGCCCCCACGCCATATCGACATTTTGCTTCGGAAAAAATT

TAGCGGCGAACTTCTTGCGACGCTCGAAGGTGTGGCAATTTCAAACGGTGCAACTGGTTG

GCTTGCGCTCGCATGTGTAAACCTTCTCTTGAAGGCAAGACAAGATATTGATGTCTTCTT

GTTTGGGGCAGGAAAAGTCGCCGAGGCTGTGATTCTTGCCCTCAATCATGGTGCGGCTGC

AAAAATCAAAACCATGGCAGTGCTCAGCCAAGCTCCAATTGAAGGTGGGGTTCTAGCTCG

ATGCCGTCATTGATTGGAAGGTTATACCAAAAGCCAAACTCGTCATAACAGCAACAAATT

CCGAGGAGCTCGGTACTCGAGGCCGACGAAATTGCGCCAAATGCAGTAACCATATCCCTC

GGAAAAGATGAATTGCCCGCCGCCTACTTCGATCGCCTTTTGAACGCAGAGGGTCTGATT

ATTGGCGACGATCTGGATGCGACCGAATCGCGCAATGTCAATTCCCTGGTGCTTTACTAC

TCGAAACGTGATTTGAAGCTGACAGAACATGGAAGGGATCATTAGATAAAGAACTACGCC

AATGTCCTTGCTGATCCAGCTCTCATGGAGGAACTTAAGACATGAGAGGGGCCAGCCAAC

TTTTCATCGGTTGGCCTTGCCAGCCTAGACTTGGCGATGGCCGGCCGACTCTACGAAACT

CTTACTGCGAAACTCTCCCACCCCCAGTAGACAAAACCCTCTTAGGATTGCGCTCTTCAG

AAGGTGCGCCTCCCCAGTCACAACCGGAAAGTCACTCAACTTGGCTCAAGTACCCTACTT

AGTTTGTCTCTCTGTCGTGTTTAGTATCAGCAACAATAACAAATATTGTAAATAACATAT

ATTTATATAGTAATTATATGGCATGTAAGCGTGACAACGATTATGTTTTCCATAGTTTGA

TAAACTTAACACCCGAAAAAAGCCGGTGACCAAAGGGTTGGGACCTCTTTGCACGAACTC

CTATGGCTGCCAGACTATCTACAGAGCTTTTTCGGAAGCTGACGCAGCATCTGATCGCCA

CACAATCCTCGCGGACGTGGATGGCGTGGCTACATGCAAGCAGTTTGATTGAGAGTTTTC

GAGTGACTATTTTAAAAGGACAATATTTCGTCCCAAGCGATAAAACTCGCTCACATGACT

AAGTCATTGCTTTAATTCAACTGCGTGAAAAGATTTAACGGGCAGAACTGTTTGTGTTTC

GTATCCAGTTCACCGGCAATTATTGGCTACGCATGAAGCTCATTATCGTCGATTCTTTAC

CCAAACACTGATGCCATTCCAAATGAGTTGGGCCAAAGATGTACTCATGTCGGACAGGCG

CGATTTGGACTTCGTGATCCTCAAGTCTATCGCAGTGCTGCTTTATTACAAGCTATGCTT

CCACCCTATATTATTTGGTCAACTGGACCTAGCTTTTGACAAGAATAGGCCAGCGTTCAT

CTATCTATCTTTCAAAAACATGCAGTTGCTGCTGTGTAAAGGATGTAACATCGGATTTGG

TGTGGAGACGGTGCTCGCTACGACTATACTGCCTTACTGTAGTGATCTGTCCCGCCAAGC

GATGAGGGGAGCGGCCTGCCTCTCATGAGCGTCAATCCATACGGTTGCATGTTCGTTTGT

TTCAAAGGCATTCCCAAGAAGGGTTTCCAGCTTGACAACTTTCTCATCGGTGGGTGTGAA

CTCGCCGGTTAGTGAGTCACCGCCCATTTTGGGAACTACCAGGGGGGTGCCAACGACCTC

CTCCGTCGACATCTCAGACCAATTTCAATTGCCCAACTGCACCGTTCTTTCTATGTTGAA

GTCATGGGTGACTATGAAATCTCCATCTTTGGTCGGTACTATCTCGATCTCGACCATATT

GAAAAGCCTGAAAGATTTGCAAACTGCCCCTACACTGTTTTCCGGCAAGTGTGGCCCATG

GAATCCACGGTGAACTATCAATTGGCGGTCGCTTGAAGTCTTTCGAGCGATAAGCCAATT

GGCGAGGCAGTGTTTCGCGATCGCATCTTTAAGAGGAAAGTCCGATGCAAATCTCCATGT

AGACCCAGTCGTTTCGGCCAAGGCGGCCTCTAAATCAACTTGAGCAGCAGTTGTGTTAAC

GGAATCTGTTTTGACTGCCATTCTGTAAGAAATGTCAGCAAAGGAAAAGCTAGTTAGAGA

AGGAGAGCTTGCTCAATAGTGTGTCTATTCTCAAATGAACTATACTAGCGCAATGAGTTG

GTGTTGTTCGCTGATGGGAAAAAGGAAATGCATCCCCCTCTTCTACAAAATTCTGTGGGC

CCGGAGGGACTCCGTATGAAAGTGCACAGCATGTGGACGCATCACCATGCAATTAGCAAG

CTTAGCCAATGTCGTGTCTGTCAGAGTGTGCAGCATGTGAGCCAAGCGCAATTACAATAA

AAAAATAATATAAAAACTCTGCCGAGGTCCGAACCGTCTCAAATGCTTTGAAGGCCATCC

AAGCCAAGCGCACCTAACGCCTAGCACACCATTGTTGCGCCGCTCGAGCATTAATGGTTC

GGGACCTGGTGTCATCTGGAAACCTATGAAGCATGGACACGGCAAAATCAGCGCSGTGGC

GGTGTCAGACACGAGGACACGTCGGGGACACGTAC

>sa7886_1

aaatttccctcaccttgaagtaTCAATTTGATCTAGAAGCCAATCCAAACTCATTGAAAG

GCCTGTCATCAACCGGTCAACACGGTTCAATGTTTGCTCGCGTGGCACAGCAATGGTGTG

CTAGGCGTTAGGTGCGCCTGGCTTGGATGGCCCTCAAAGCATTTGAGGCAGTTCGGACCT

CGGCAGAGATTTTATATTATTTTTTTGTTGTAATCACGCTTGGCCCACATGCTGCACACT

CCGATAGACACGGCATTGGCTAAACCCGCTAATTGCGTGGTGATGCGTCCACGTGCTGCG

CACTTTCAGGCGGAGTCCCCCcGATTCCATAGAATAAGAGGGGGACGCGTTTCCTTTTTC

CCATCAGCAAACAACACCAACTCACTGTGCTAGTATAGTTCATCTGAGAACAGACACACT

GCTGAGCAAGCTCTCCTTCTCCAACTAGCTTTTTCTTTGCTGACATTTCCTACAGAATGG

CAGCAAAACAGATTCCGTTAACACAACTACTGCTCAGGTTGATTTAGAGGCCGCCTTGGC

CGAAGCGATCGGGTCTACATGGAGATTTGCATCAGACTTTCCTCTTAAAAATGCAATCGC

GAAACACTGCCTCGCCAATTGGCTTATCGCTCGAAAGACTTCAAGCAACCACCAATTGAT

AGTTCACCGTGAATTCCATGGGCCACACCTGCCGGAAAACAGCGTAGGGGCAGTCTGCAA

ATCTCTCAAACTTTTTAATATGGTCGAGATCAACGTAGTACCGACCAAAGATAAAGATTT

CATAGTCGTCCACGACTTCAACACAGAAAGAACGACGCAGTTAGGCAATTGAAATTGGTT

TGATATGTCGACGAAAGAGGCTGTTGGCACCCCCCTGGTAGTTCCCATAATGGGCGGTGA

CTCCCTAACCAGCGAGTTCACACCCACCGATGAGAAAGTTGTCAAGCAAGAAACCCTTCT

CAAGAATGCCTTTGAAACAAACAAACAGGCAACCATACGGATTGACGCTCACGAGAGGCA

GGCCGCTCCCCTCGTCGCTTGGCTGAGCCATAGGCCGGCCTATCACGACAAGACAATCGT

TATCTTTCATACATATGGATACAACAATGGACACGAGTTCGCCTTGGCGGTCGATCAAGC

CGGCGCTACAAAAAATTGGCGCGAAACTGTAGCGCTAATGCCAAACATTCTGCCGAATGA

ACTCTCTAGGCTCGCGAAGCAATATCCACCAAATTGCACGTCGCATTTTTACGACCTCGT

TGAGGCCAGCAAGAATTGGTTCGCATCTGTCTTCTCCCAGAATATGATGGTGGTCGTCAC

TTTCCTCAAGATTGcgggcactggcgaaggacagactaacaaagtcgttgatCCAAATGG

AAATGAGGTTCTGCATCCTAAAGAGAAGGAGGAATATCTCCGCGATTCAGCTTCAATCGA

AATCGCGAAATGAATCTGCGCCACCCATCCCGGAATCGAGATTGGAGGCGTCACACGAAC

TTATGCTTATAAGAGTCGAGGACATCGCTACAAAATCAATCTTTGGACGGGTCAACCGAA

GCGGTGGCCGACCCACGCGACGAAGTACATCTGGGAAGATTTCGCCAAGCCTGGAAATGC

GCTCAGATGGGGTCAAACGATTATCTCTGATCGCATTGGTGACGACCTTGCAGCTTGTGT

GCTTCAAAGCTGTGGAGAAAATTTTGTCAGCAGTCATGAATATCCTGCTGTTCATGTCGA

TTAGATGGGCGATCTGTGTCTTAGTCTAAGCTTCCGCTTAACGCATCTTTGCTACTTTAA

TGTATTTTTTTAATGATATTGTTGGAGTTTTGAGCACATCTTAATCGCAAGTAGATATTT

TTTTTGAATCGGATTCGACTTCAAGCAGATGTACTTCTTTGTCTCTCGATAAATCTAATG

ACCCTGCGACCGGCCTCTCGCCTTAAACTAATTTCTGAGAGTTAGGTCGGCGCCTCTCGT

GGTTTCCATGTTCTATGGAAACGGCCGCTCGGGACTTGCCTTCCCGCAACTCATCGAGCT

TGCCTGGCCCGCTCGGGTGGTGGCACAAGCCATCGTTAAATCGTTCGTAATCCTCTACTC

TCGCATAAATGAGAGGGGAATCCAAAGATGATCACTTTTCAGCTAATTGGAATCTGCAAT

CCCAAAAGAGGCAAGCACTATGTGGGGTCCACAACTTATAGAATTTTGGCCTAACCATTC

TGCATAATGGATCATGACATGGGCCATTTATGGCCGTAAAAACTGGCACAAATTCCAACC

TATTCAGCACAATGAACAAGTTCTTCCAATTGGAATAAAGGAGGTTAAAATTATTCGAGT

AGACATGACGTGCTGGCATGCCACTAGCCTCGCATTGATGCCACTCTTTCTTGCGCACAC

TTATATAATGCTGAGCTAATAATAGAGATAAAAATAGTCAGCTTGTAAACTGGACCTTTA

TTACTGCCCCTAATCTCAAGATCAAAGCGACATATACCTCTTCTCTTTCAAGTCTTTTTG

CAACTTGTTCCAAAGCAAGCGTTTGTGAATCTGCCAGGCAGCCTTCACGAAAATGGCTGA

TCAACTGTGTGAGGGGCGTTGGTCCGAAGCCAATCTATCATTCGTTGGGATATCCATCAC

CCCTGAATTCATCGATGAGGCCCTCAGAGGCTTTTGGGAGTCTTTTGCCAAAAGAGCCGT

TGGCTACAAAGAGGTTTACGTTCAGGTTGATCGAGATCCTAAAGTTGCCTGGGAAGGAGC

GCTCAATGGTTACCTCGGCAAATATTCTGGCGTAAAAAATGTTCATTTTGCGCCGAACAA

CGCCACGATTGGAAAGCCCCCACGCCATATCGACATTTTGCTTCGGAAAAAATTTAGCGG

CGAACTTCTTGCGACGCTCGAAGGTGTGGCAATTTCAAACGGTGCAACTGGTTGGCTTGC

GCTCGCATGTGTAAACCTTCTCTTGAAGGCAAGACAAGATATTGATGTCTTCTTGTTTGG

GGCAGGAAAAGTTGCCGAGGCTGTGATTCTTGCCCTCAATCATGGTGCGGCTGCAAAAAT

CAAAACCATGGCAGTGCTCAGCCAAGCTCCAATTGAAGGTGGGGTTCCAGCTTGATGCCG

TCATTGATCGGAAGGTTATACCAAAAGCCAAACTCGTCATAACAGCAACAAATTCCGAGG

AGCTCGGTACTCGAGGCCGACGAAATTGCGCCAAATGCAGTAACCATATCCCTCGGAAAA

GATGAATTGCCCGCCGCCTACTTCGATCGCCTTTTGAACGCAGAGGGTCTGATTATTGGC

GACGATCTGGATGCGATCGAATCGCACAATGTCAATTCCCTGGTGCTTTACTACTCGAAA

CGTGATTTGAAGCTGACAGAACATGGAAGGGATCATTGGATAAAGAACTACGCCAATGTC

CTTGCTGATCCAGCTCTCATGGAGGAACTTAAGACATGAGAGGGGCCAGCCAACTTTTCA

TCGGTTGGCCTTGCCAGCCTAGACTTGGCGATGGCCGGCCGACTCTACGAAACTCTTACT

GCGaaactctcccAcCCCCAGTAgACAAAACCCTcttaggattgcgctcttCAGAaGGTG

CGCCTCCCCAGTCACAACCGGAAAGTCHCTCAACTTGGCTCAAGTACCCTACTTAGTTTG

TCTCTCTGTCGTGTTTAGTATCAGCAACAATAACAAATTTTGTAAATaACATATATTTAT

AtgGTAaTtATATGGCATGTaagCGtGACAACGATTATGTTTTCCATAGTTTGATAAACT

CAACACCCGAAAAAGCCGGTGACCAAAGGGTTGGGACCTCTTTGCACGAACTCCTATGGC

TGCCAGACTATCTACAGAGCTTTTTCGGAAGCTGACGCAGCATCTGATCGCCACACAATC

CTCGCGGACGTGGATGGCGTGGCTACATGCAAGCAGTTTGACTGAGAGTTTTCGAGTGAC

TATTTTAAAAGGACAATATTTCGTCCCAAGTGATAAAACTCGCTCACATGACTAAGTCGT

TGCTTTAATTCAACTGCGTGAAAAGATTTAAAGGGCAGAACTGTTTGTGTTTCGTATCCA

GTTCACCGGCAATTATTGGCTACGCATGAAGCTCATTATCGTCGATTCTTTACCCAAACA

CTGATGCCATTCCAAATGAGTTGGGCCAAAGATGTACTCATGTCGGACAGGCGCGATTTG

GACTTCGTGATCCTCAAGTCTATCGCAGTGCTGCTTTATTACAGGCTATGCTTCCACCCT

CCATTATTTGGTCAACTGGACCTAGCTTTTGACAAGAATAGGCCAGCGTTCATCTATCTA

TCTTTCAAAAACATGCAGTTGCTGCTGCGTAAAGGATGTAACATCGGATTTGGTGTGGAG

ACGGTGCTCGCTACGACTATACTGCCTTACTGTAGTGATCTGTCCCGCCAAGCGATGAGG

GGAGCGGCCTGCCTCTCGTGAGCGTCAATCCATACGGTTGCATGTTCGTTTGTTTCAAAG

GCATTCCCAAGAAGGGTTTCCAGCTTGACAACTTTCTCATCGGTGGGTGTGAACTCCCCG

GTTAGTGAGTCACCGCCCATTTTGGGAACTACCAGGGGGTGCCAACGACCTCCTCCGTCG

ACATCTCAGACCAATTTCAATTGCCCAATTGCACCGTTCTTTCTATGTTGAAGTCATGGG

CGACTATGAAATCTCCATCTTTGGTCGGTACTACCTCGATCTCGACCATATTGAAAAGCC

TGAAAGATTTGCAAACTGCCCCTACACTGTTTTCCGGCAAGTGTGGCCCATGGAATCCAC

GGTGAACTATCAATTGGCGGTCGCTTGAAGTCTTTCGAGCGATAAGCCAATTGGCGAGGC

AGTGTTTCGCGATCGCATCTTTAAGAGGAAAGTCCGATGCAAATCTCCATGTAGACCCAG

TCGTTTCGGCCAAGGCGGCCTCTAAATCAACTTGAGCAGTAGTTGTGTTAACGGAATCTG

TTTTGACTGCCATTCTGTAAGAAATGTCAGCAAAGGAAAAGCTAGTTAGAGAAGGAGAGC

TTGCTCAATAGTGTGTCTATTCTCAAATGAACTATACTAGCGCAATGAGTTGATGTTGTT

CGCTGATGGAAAAAAGGAAACGCATCCCCCTCTTCTACAAAATTCTGTGGGCCCGGAGGG

ACTCCGTATGAAAGTGCACAGCATGTGGACGCATCACCATGCAATTAGCAAGCTTAGCCA

ATGTCGTGTCTGTcaAATTGCCATCCCTAAGCATGTGAGCCAAGCGCAATTACAATAAAA

AAATAATATAAAAACTCTGCCGAGGTCCGAACCGTCTCAAATGCTTTGAGGGCCATCCAA

GCCAAGCGCACCTAACGCCTAGCACACCATTGTTGCGCCACTCGAGCATTAATGGTTCGG

GACCTGGTGTCATCTGGAAACCTATGAAGCATGGACACggcaaaatcagcgccgtggcgg

tgtcagacacgaggacacgtcggggacacgtacaggacacg

>sa7886_2

tccAAACTCATTGAAAGGCCTGTCATCAACCGGTCAACACGGTTCAATGTTTGCTCGCGT

GGCGCAGCAATGGTGTGCTAGGCGTTAGGTGCGCCTGGCTTGGATGGCCCTCAAAGCATT

TGAGGCAGTTCGGACCTCRGCAGAGATTTTATATTATTTTTTTGTTGTAATCGCGCTTGG

CCCACATGCTGCACACTCCGATAGACACGGCATTGGCTAAACCCGCTAATTGCGTGGTGA

TGCGTCCACGTGCTGCGCACTTTCATGCGGAGTCCCCCCGAGTCCATAGAATAAGAGGGG

GACGCGTTTCCTTTTTCCCATCAGCAAACAACACCAACTCACTGTGCTAGTATAGTTCAT

CTGAGAACAGACACACTGCTGAGCAAGCTCTCCTTCTCTAACTAGCTTTTTCTTTGCTGA

CATTTCCTACAGAATGGCAGCAAAACAGATTCCGTTAACACAACTACTGCTCAGGTTGAT

TTAGAGGCCGCCTTGGCCGAAGCGATCGGGTCTACATGGAGATTTGCATCAGACTTTCCT

CTTAAAAATGCAATCGCGAAACACTGCCTCGCCAATTGGCTTATCGCTCGAAAGACTTCA

AGCAAccaccaattgatagttcaccgtgaattccatgggccacacctgccGGAAAACAGC

GTAGGGGCAGTCTGCAAATCTCTCAGACTTTTTAATATGGTCGAGATCAACGTAGTACCG

ACCAAAGATAAAGATTTCATAGTCGTCCACGACTTCAACACAGAAAGAACGACGCAATTA

GGCAATTGAAATTGGTTTGATATGTCGACGAAAGAGGCTGTTGGCACCCCCCTGGTAGTT

CCCATAATGGGCGGTGACTCCCTAACCAGCGAGTTCACACCCACCGATGAGAAAGTTGTC

AAGCAAGAAACCCTTCTCAAGAATGCCTTTGAAACAAACAAACAGGCAACCATACGGATT

GACGCTCACGAGAGGCAGGCCGCTCCCCTCGTCGCTTGGCTGAGCCATAGGCCGGCCTAT

CACGACAAGACAATCGTTATCTTTCATACATATGGATACAACAATGGACACGAGTTCGCC

TTGGCGGTCAATCAAGCCGGCGCTACAAAAAATTGGCGCGAAACTGTAGCGCTAATGCCA

AACATTCTACCGAATGAACTCTCTAGGCTCGCGAAGCAATATCCACCAAATTGCACGTCG

CATTTTTACGACCTCGTTGAGGCCAGCAAGAATTGGTTCGCATCTGTCTTCTCCCAGAAT

ATGATGGTGGTCGTCACTTTCCTCAAGATTGCGGGCACTGGCGAAGGATAGACTAACAAA

GTCGTTGATCCAAATGGAAATGAGGTTCTGCATCCTAAAGAGAAGGAGGAATATCTCCGC

GATTCAGCTTCAATCGAAATCGCGAAATGAATCTGCGCCACCCATCCCGGAATCGAGATT

GGAGGCGTCACACAAACTTATGCTTATAAGAGTCGAGGACATCGCTACAAAATCAATCTT

TGGACGGGTCAACCGAAGCGGTGGCCGACCCACACGACGAAGTACATCTGGGAAGATTTC

GCCAAGCCTGGAAATGTGCTTAGATGGGGTCAAACGATTATCTCTGATCGCATTGGTGAC

GACCTTGCAGCTTGTGTGCTTCAAAGCCGTGGAGAAAATTTTGTCAGCAGTCATGAATAT

CCTGCTGTTCATGTCGATTAGATGGGCGATCTGTGTCTTAGTCTAAGCTTCCGCTTAACG

CATCTTTGTTACTTTAATGTATTTTTTTAATGATATTGTTGGAGTTTTGAGCACATCTTA

ATCGCAAGTAGATATTTTTTTTGAATCGGATTCGACTTCAAGCAGATGTACTTCTTTGTC

TCTCGACAAATCTAATGACCCTGCGAGCCGGCCTCTCGCCTTAAACTAATTTCTGAGAGT

TAGGTCGGCGCCTCTCGTGGTTTCCATGTTCTATGGAAACGGCCGCTCGGGACTTGCCTT

CCCGCAACTCATCGAGCTTGCCTGGCCCGCTCGGGTGGTGGCACAAGCCATCGTTAAATC

GTTCGTAATCCTCTACTCTCACATAAATGAGAGGGGAATCCAAAGATGATCACTTTTTAG

CTAATTGGAATCTGCAATCCCAAAAGAGGCAAGCACTATGTGGGGTCCACAACTTATAGA

ATTTTGGCCTAACCATTCTGCATAATGGATCATGACATGGGCCATTTATGGCCGCAAAAA

CTGGCACAAATTCCAACCTATTCAGCACAATGAACAAGTTCTTCCAATTGGWATAAAGGA

GGTTAAAATTATTCGAGTAGACATGACGTGCTGGCACGCCACTAGCCTCGCATTGATGCC

ACTCTTTCTTGCGCACACTTATATAATGCTGAGCTAATAATAGAGATAAAAATAGTCAGC

TTGTAAACTGGACCTTTATTACTGCCCCTAATCTCAAGATCAAAGCGACATATACCTCTT

CTCTTTCAAGTCTTTTTGCAACTTGTTCCAAAGCAAGcgtttGTgaatctgccaggcagc

cttcacgaaaatggcTGATCAACTgTGTGAGGGGCGTTGGTCCGAAGCCAATCTATCATT

CGTTGGGATATCCATCACCCCTGAATTCATTGATGAGGCCCTCAAAGGCTTTTGGGAGTC

TTTTGCCAAAAGagccgttggcgacaaagaggtttacgttcaggttgatcgagatcctaa

agttgcctgggaaggagcgctcaatggttacctcggcaaatattctggcgtaaaaaatgt

tcattttgcgccGAACAACGCCACGATTGGAAAGCCCCCACGCCATATCGACATTTTGCT

TCGGAAAAAATTTAGCGGCGAACTTCTTGCGACGCTCGAAGGTGTGGCAATTTCAAACGG

TGCAACTGGTTGGCTTGCGCTCGCATGTGTAAACCTTCTCTTGAAGGCAAGACAAGATAT

TgatgtcttcttgtttggggcaggaaaagtcgccgAGGCTGTGATTCTTGCCCTCAATCA

TGGTGCGGCTGCAAAAATCAAAACCATGGCAGTGCTCAATCAAGCTCCAATTGAAGGTGG

GGTTCCAGCTCGATGCCGTCATTGATCGGAAGGTTATACCAAAAGCCAAACTCGTCATAA

CAGCAACAAATTCCGAGGAGCTCGGTACTCGAGGCCGACGAAATTACGCCAAATGCAGTA

ACCATATCCCTCGGAAAAGATGAATTGCCCGCCGCCTACTTCGATCGCCTTTTGAACGCA

GAGGGTCTGATTATTGGCGACGATCTGGATGCGATCGAATCGCGCAATGTCAATTCCCTG

GTGCTTTACTACTCGAAACGTGATTTGAAGCTGACAGAACATGGAAGGGATCATTGGATA

AAGAACTACGCCAATGTCCTTGCTGATCCAGCTCTCATGGAGGAACTTAAGACATGAGAG

GGGCCAGCCAACTTTTCATCGGTTGGCCTTGCCAGCCTAGACTTGGCGATGGCCGGCCGA

CTCTACGAAACTCTTACTGCGAAACTCTCCCACCCCCAGTAGACAAAACCCTCTTAGGAT

TGCGCTCTTCAGAAGGTGCGCCTCCCCAGTCACAACCGGAAAGTCACTCAACTTGGCTCA

AGTACCCTACTTAGTTTGTCTCTCTGTCGTGTTTAGTATCAGCAACAATAACAAATATTG

TAAATAACATATATTTATATGGTAATTATATGGCATGTAAGCGTGACAACGATTATGTTT

TCCATAGTTTGATAAACTCAACACCCGAAAAAGCCGGTGACCAAAGGGTTGGGACCTCTT

TGCACGAACTCCTATGGCTGCCAGACTATCTACAGAGCTTTTTCGGAAGCTGACGCAGCA

TCTGATCGCCACACAATCCTCGCGGACGTGGATGGCGTGGCTACATGCAAGCAGTTTGAC

TGAGAGTTTTCGAGTGACTGTTTTAAAAGGACAATATTTCGTCCCAAGCGATAAAACTCG

CTCACATGACTAAGTCGTTGCTTTAATTCAACTGCGTGAAAAGATTTAAAGGGCAGAACT

GTTTGTGTTTCGTATCCAGTTCACCGGCAATTATTGGCTACGCATAAAGCTCATTATCGT

CGATTCTTTACCCAAACACTGATGCCATTCCAAATGCGTTGGGCCAAAGATGTACTCATG

TCGGACAGGCGCAATTTGGACTTCGTGATCCTCAAGTCTATCGCAGTGCTGCTTTATTAC

AGGCTATGCTTCCACCCTCCATTATTTGGTCAACTGGACCTAGCTTTTGACAAGAATAGG

CCAGCGTTCATCTATCTATCTTTCAAAAACATGCAGTTGCTGCTGCGTAAAGGATGTAAC

ATCGGATTTGGTGTGGAGACGGTGCTCGCTACGACTATACTGCCTTACTGTAGTGATCTG

TCCCGCCAAGCGATGAGGGAAGCGGCCTGCCTCTCGTGAGCGTCAATCCATACGGTTGCA

TGTTCGTTTGTTTCAAAAGCATTCCCAAGAAGGGTTTCCAGCTTGACAACTTTCTCATCG

GTGGGTGTGAACTCGCCGGTTAGTGAGTCACCGCCCATTTTGGGAACTACCAGGGGGGTG

CCAACGACCTCCTCCGTCGACATCTCAGACCAATTTCAATTGCCCAACTGCACCGTTCTT

TCTATGTTGAAGTCATGGGCGACTATGAAATCTCCATCTTTGGTCGGTACTACCTCGATC

TCGACCATATTGAAAAGCCTAAAAGATTTGCAAACTGCCCCTACACTGTTTTCCGGCAAG

TGTGGCCCATGGAATCCACGGTGAACTATCAATTGGCGGTCGCTTGAAGTCTTTCGAGCG

ATAAGCCAATTGGCGAGGCAGTGTTTCGCGATCGCATCTTTAAGAGGAAAGTCCGATGCA

AATCTCCATGTAGACCCAGTCGTTTCGGCCAAGGCGGCCTCTAAATCAACTTGAGCAGCA

GTTGTGTTAACGGAATCTGTTTTGACTGCCATTCTGTAAGAAATGTCAGCAAAGGAAAAG

CTAGTTAGAGAAGGAGAGCTTGCTCAATAGTGTGTCTATTCTCAAATGAACTATACTAGC

GCAATGAGTTGGTGTTGTTCGCTGATGGGAAAAAGGAAACGCATCCCCCTCTTCTACAAA

ATTCTGTGGGCCCGGAGGGACTCCGTATGAAAGTGCACAGCATGTGGACGCATCACCATG

CAATTAGCAAGCTTAGCCAATGTCGTGTCTGTCAGAGTGTGCAGCATGTGAGCCAAGCGC

AATTACAATAAAAAAATAATATAAAAACTCTGCCGAGGTCCGAACCGTCTCAAATGCTTT

GAGGGCCATCCAAGCCAAGCGCACCTAACGCCTAGCACACCATTGTTGCGCCGCTCGAGC

ATTAATGGTTCGGGACCTGGTGTCATCTGGAAACCTATGAAGCATAGACacggcaaaatc

agcgccgtggcggtgtcagacacgaggacacgtcggggacac

>tr3960_Un

AATTTGATCTAGAAGCCAATCCAAACTCATTGAAAGGCCTGTCATCAACCTGTCAACACG

GTTCAATGTTTGCTCGAGTGGCGCAGCAATGGTGTGCTAGGCGTTAGGTGCGCCTGGCTG

GGATGGCCCTCAAAGCATTTGAGACAGTTCGGACCTCGGCAGAGATTTTATATTATTTTT

TTGTTGTAATCGCGTTTGGCCCACATGCTGCACACTCCGACAGACACGGCATTGGCTAAA

CCCGCTAATTGCGTGGTGATGCGTCCACGTGCTGCGCACTTTCATGCGGAGTCCCCCCGA

GCCCGTAGAATTTTGTAGAAGAGGGGGACGCGTTTCCTTTTTCCCATCAGCAAACAACAC

CAACTCACTGTGCTAGTATAGTTCATCTGAGAACAGACACACTGCTGAGCAAGCTCTCCT

TCTCCAACTAGCTTTTTCTTTGCTGACATTTCCTACAAAATGGCAGCAAAACAGATTCCG

TTAACACAACTGCTGCTCAGGTTGATTTAGAGGCCGCCTTGGCCGAAGCGACCGGGTCTA

CATGGAGATTTGCATCAAACTTTCCTCTTAAAGATGCGATCGCAAAACACTGCCTCGCCA

ATTGGCTTATCGCTCGAAAGACTTCAAGCAACCACCAATTGATAGTTCACCGTGAATTCC

ATGGGCCACACCTGCCGGAAAACAGCGTAGGGGCAGTCTGCAAATCTCTCAGACTTTTTA

ATATGGTCGAGATCAACGTAGTACCGACCAAAGATAGAGATTTCATAGTCGTCCACGACT

TCAACACAAAAAGAACGACGCAGTTAGGCAATTGAAATTGGTCTGATATGTCGACGAAAG

AGGCCGTTGGCACCCCCCTAGTAGTTCCCATAATGGGCGGTGACTCCCTAACCAGCGAGT

TCACACCCACCGATGAGAAAGTTCTCAAGCAAGAAACCCTTCTCAGGAATGCCTTTGAAA

CAAACAAACAGGCAACCATACGGATTGACGCTCACGAGAGGCAGGCCACTCCCCTCGTCG

CTTGGCTGAGCCATAGGCCGGCCTATCACGACAAGACAATCGTTATCTTTCATACATATG

GATACAACAATGGACACAAGTTCGCCTTGGCGGTCGATCAAGCCGACGCTACAGAAAATT

GGCGCGAAACTGTAGCGCTAATGCCAAACATTCTGCCAAATGAACTCTCTAGGCTCGCGA

AGCAATATCCACCAAATATCACGTCGCATTTTTACGACCTCGTTGAGGCCGGCAAGAATT

GGTTCGCGTCTGTCTTCTCCCAGAATATGATGGTGGACGTCACTTTCCTCAAGATTGCGG

GCACTGGCGAAGGACAGACTAACAAAGTCGTTGATCCAAATGGAAATGAGGTTCTGCATC

CTAAAGAGAAGAAGGAATATCTCCGCGATTCAGCTTCAATTGAAATCGCGAAATGAATCT

GCGCCACCCATCCCGGAATCGAGATTGGAGGCGTCACACGAACTTATGCTTATAAGAGTC

GAGGACATCGCCACAAAATCGATCTTTGGACGGGTCAACCGAAGCGGTGGCCGACCCACG

CGACGAAGTATATCTGGGAAGATTTCGCCAAGCCTGGAAATGCGCTCAGATGGGGTCAAA

CGATTATCTCTGATCGCATTGGCGACGACCTTGCAGCTTGTGTGCTTCAAAGCCGTGGAG

AAAATTTTGTCAGCAGTCATGAATATCCCGCTGTTCATGTCGATTAGATGGGCGATCTGT

GTCTTAGTCTAAGCTTCCGCTTAACGCATCTTTGCTACTTTAATGTATTTTTTTAATGAT

ATTATTTCAGCACATCTTAATCGCAAGTAGATATTTTTTTTGAATCGGATTCGACTTCAA

GCAAATGTACTTCTTTGTCTCTCGATAAATCTAATGACCCTGCGAGCCGGCCTTTCGCCT

TAAACTAATTTCTGAGAGTTAGGTCGGCGCCTCTTGTGGTTTCCATGTTCTATGGAAACG

GCCTCTCGGGACTTGCCTTCCCGCAACTCATCGAGCTTGCCTGGCCCGCTCGGGTGGTGG

CGCAAGCCATCGTTAAATCGTTCGTAATCCTCTACTCTCGCATAAATGAGAGGGGAATCC

AAAGATGATCACTTTTCAGCTAATTGGAATCTGCAATCCTAAAAGAGGCAAGCACTATGT

GGGGTCCACAACTTATAGAATTTTGGCCTAACCATTCTGCATAATGGATCATGACATGGG

CCATTTATGGCCGCAAAAACTGGCACAAATTCCAACCTGTTCAGCACAATGAACAAGTTC

TTCCAATTCGAATAAAGGAGGTTAAAATTATTCGAGTAGACATGATGTGCCGGCACGCCA

CTAGCCTCGCATTGATGCCACCCTTTCTTGCGCACACTTATATAATGCTGAGCTAATAAT

AGAGATAAAAATAGTCAGCTTGTAAACTGGACCTTTACTACTGCCCCTAATCTCAAGATC

AAAGCGACATATACCTCCTCTCTTTCAAGTCTTTTTGCAACTTGTTCCAAAGCAAGCGTT

CGTGAATCTGCCAGGCAGCCTTCACGAAAATGGCTGATCAACTATGTGAGGGGCGTTGGT

CCGAAGCCAATCTATCATTCGTTGGGATATCCATCACCCCTGAATTCATCGATGAGGCCC

TCAGAGGCTTTTGCCAAAAGAGCCGTTGGCTACAAAGAGGTTTACGTTCGGGTTGATCGA

GATCCTAAAGTTGCCTGGGAAGGAGCGCTCAATGGTTACCTCGGCAAATATTCTGGCGTC

AAAAATGTTCATTTTGCGCCGAACAACGCCGCGATTGGAAAGCCCCCATGCCATATCGAC

ATTTTGCTTCGGAAAAAATTTAGCGGCGAATTTCTTGTGACGCTCGAAGGTGTGGCAATT

TCAAACGGTGCAACTGGTTGGCTTGCGCTCGCGTGCGTAAACCTTCTCTTGAAGGCAAGA

CAAGATATTGATGTCTTCTTGTTTGGGGCGGGAAAAGTCGCCGAGGCTGTGATTCTTGCC

CTCAATCACGGTGCGGCTGCAAAAATCAAAACCATGGCAGTGCTCAGCCAAGCTCCAATC

GAAGGTGGGGTTCCAGCTCGATGTCGTCATTGATCGGAAGGTTATACCAAAAGCCAAACT

CGTCATAACAGCAACAAATTCCGAGGAGCTCGCACTCGAGGCCGACGAAATTGCGCCAAA

TGCAGTAACCATATCCCTCGGAAAAGATGAATTGCTCGCCACCTACTTCGATCGCCTTTT

GAACGCAGAGGGTCTGATTATCGGCGACGATCTGGATGCGATCGAATCGCGCAATGTCAA

TTCCCTGGTGCTTTACTACTCGAAACGTGATTTGAAGCTGACAGAACATGGAAGGGATCA

TTGGATAAAAAACTACGCCAATGTCCTTGTTGATCCAGCTCTCATGGAGGAACTTAAGAC

ATGGGAGGGGCCAGCCAACTTTTCATCGGTTGGCCTTGCCAGCCTAGACTTGGCGATGGC

CGGCCGACTCTACGAAACTCTTACTGCGAAACTCTCCCACCCCCAGTAGACAAGACCCTC

TTAGGATTGCGCTCTTCAGAAGGTGCGCCTTCCCAGTCACAACCGGAAAGTCACTCAACT

TGGCTCAAGTACCCTACTTAGTTTGTCTCTCTGTCGTGTTTAGTATCAACAACAATAACA

AATATTGTAAACAACATATATTTATATGGTAATTATATGGCATGTAAGCGTGACAACGAT

TATGTTTTCCATGGTTTGATAAACTCAACACCCGAAAAAGCCGGTGACCAAAGGGCTGGG

ACCTCTTTGCACGAACTCCTATGGCTGCCAGACTATCTACAGAGCTTTTTCGAAAGCTGA

CGCAGCATCTGATCGCTACACAATCCTCGCGGACGTGGATGGCGTGGCTACACACAAGCA

GTTTGACTGAGAGTTTTCGAGTGACTATTTTAAAAGGACGATATTTCGTCCCAAGCAATA

AAACTCGCTCACATGACTAAGTCGTTGCTTTAATTCAGCTGCGTGAAAAGATTTAAAGGG

CAGAACTGTTTGTGTTTCGTATCCAGTTCACCGGCAATTATTGGCTACGCATGAAGCTCA

TTATCGTCGATTCTTTACCCAAACACTGATGTCATTCCAAATGAGTTGGGCCAAAGATGT

ACTCATGTCGGACAGGCGCGATTTGGACTTCGTGATCCTCAAGTCTATCGCAGTGCTGCT

TTATTACAGGCTATGCTTCCACCCTCCATTATTTGGTCAACTGGACCTGGCTTTTGACAA

GAATAGGCCAGCGTTCATCTATCTATCTTTCAAAAACATGCAGTTGCTGTTGCGTAAAGG

ATGTAACATCGGATTTGGTGTGGAGACGGTGCTTGCTACGACTATACTGCCTTACTGTAG

TGATCTGTCCTGCCAAGCGACGAGGGGAGCAGCCTGCCTCTCGTGAGCGTCAATCCATAC

AGTTGCATGTTCGTTTGTTTCAAAGGCATTCCCAAGAAGGGTTTCCAGCTTGACAACTTT

CTCATCGGTGGGTGTGAACTAGCCGGTTAGTGAGTCACCGCCCATTTTGGGAACTACCAG

GGGGTGCCAACGACCTCCTCCGTCGACATCTCAGACCAATTTCAATTGCTCAACTGCGCC

GTTCTTTCTATGTTGAAGTCATGGGCGACTATGAAATCTCCATCTTTGGTCGGTACTACC

TCGATCTCGACCATATTGAAAAGCCTGAGAGATTTGCAAATTGCCCCTACACTGTTTTCC

GGCAAGTGTGGCCCATGGAATCCACGGTGAACTATCAATTGGCGGTCGCTTGAAGTCTTT

CGAGCGATAAGCCAATTGGCGAGGCAGTGTTTCGCGATCGCATCTTTAAGAGGAAAGTCC

GATGCAAATCTCCATGTAGACCCGGTTGCTTCGGCCAAGGCGGCCTCTAAACCAACTTGA

GCAGCAGTTGTGTTAACGGAATCTATTTTGACTACCATTTTGTAAGAAATGTCAGCAAAG

GAAAAGCTAGTTAGAGAAGGAGAGCTTGCTCAATAGTGTGTCTATTCTCATATGAACTAT

ACTAGCGCAATGAGTTAGTGTTGTTCGCTGATGGGAAAAAGGAAATGCATCCCCCTCTTC

TACAAAATTCTGTGGGCCCGGAGGGACTCCGTATGAAAGTGCACAGCATGTGGACGCATC

ACCATGCAATTAGCGAGCTTAGCCAATGCCGTGTCTGTCAGAGTGTGCAGCATGTGGGCC

AAGCGCAATTACAATAAAAAAATAATATAAAAACTCTGCCGAGGTCTGAACCGTCTCAAA

TGCTTTGAGGGCCATCCAAGCCAAGCGCACCTAACGCCTAGCACACCATTGTTGCGCCGC

TCGAGCATTAATGGTCCGGGACCTGGTGTCATCTGGAAACCTATGAAGCATGGACACGGC

AAAATCAGCGCCGTGGCGGTGTCAGACACGGGGGACACGTCGGGGACACGTA

>tr3972_Un

TTTCCCTCACCTTGAAGTATCAATTTGATCTAGAAGCCAATCCAAACTCATTGAAAGGCC

TGTCATCAACCGGTCAACACGGTTCAATGTTTGCTTGCGTGGCGCAGCAATGGTGTGCTA

GGCGTTAGGTGCGCCTGGCTTGGATGGCCCTCAAAGCATTTGAGGCAGTTCGGACCTCGG

CAGAGATTTTATATTATTTTTTTGTTGTAATCGCGCTTGGCCCACATGCTGCACACTCCG

ATAGACACGGCATTGGCTAAACCCGCTAATTGCGTGGTGATGCGTCCACGTGCTGCGCAC

TTTCATGCGGAGTCCCCCCGAGTCCATAGAATAAGAGGGGGACGCGTTTCCTTTTTCCCA

TCAGCAAACAACACCAACTCACTGTGCTAGTATAGTTCATCTGAGAACAGACACACTGCT

GAGCAAGCTCTCCTTCTCCAACTAGCTTTTTCTTTGCTGACATTTCCTACAGAATGGCAG

CAAAACAGATTCCGTTAACACAACTACTGCTCAGGTTGATTTAGAGGCCGCCTTGGCCGA

AGCGATCGAGTCTACATGGAGATTTGCATCAGACTTTCCTCTTAAAAATGCAATCGCGAA

ACACTGCCTCGCCAATTGGCTTATCGCTCGAAAGACTTCAAGCAACCACCAATTGATAGT

TCACCGTGAATTCCATGGGCCACACCTGCCGGAAAACAGCGTAGGGGCAGTCTGCAAATC

TCTCAGACTTTTTAATATGGTCGAGATCAACGTAGTACCGACCAAAGATAAAGATTTCAT

AGTCGTCCACGACTTCAACACAGAAAGAACGACGCAGTTAGGCAATTGAAATTGGTTTGA

TATGTCGACGAAAGAGGCTGTTGGCACCCCCTTGGTAGTTCCCATAATGGGCGGTGACTC

CCTAACCAGCGAGTTCACACCCACCGATGAGAAAGTTGTCAAGCAAGAAACCCTTCTCAA

GAATGCCTTTGAAACAAACAAACAGGCAACCATACGGATTGACGCTCACGAGAGGCAGGC

CGCTCCCCTCGTCGCTTGGCTGAGCCATAGGCCGGCCTATCACGACAAGACAATCGTTAT

CTTTCATACATATGGATACAAAAATGGACACGAGTTCGCCTTGGCGGTCAATCAAGCCGG

CGCTACAAAAAATTGGCGCGAAACTGTAGCGCTAATGCCAAACATTCTGCCGAATGAACT

CTCTAGGCTCGCGAAGCAATATCCACCAAATTGCACGTCGCATTTTTACGACCTCGTTGA

GGCCAGCAAGAATTGGTTCGCATCTGTCTTCTCCCAGAATATGATGGTGGTCGTCACTTT

CCTCAAGATTGCGGGCACTGGCGAAGGACAGACTAACAAAGTCGTTGATCCAAATGGAAA

TGAGGTTCTGCATCCTAAAGAGGAGGAGGAATATCTCCGCGATTCAGCTTCAATCGAAAT

CGCGAAATGAATCTGCGCCACCCATCCCGGAATCGAGATTGGAGGCGTCACACGAACTTA

TGCTTATAAGAGTCGAGGACATCGCTACAAAATCAATCTTTGGACGGGTCAACCGAAGCG

GTGGCCGACCCACGCGACGAAGTACATCTGGGAAGATTTCGCCAAGCCTGGAAATGCGCT

CAGATGGGGTCAAACGATTATCTCTGATCGCATTGGTGACGACCTTGCAGCTTGTGTGCT

TCAAAGCCGTGGAGAAAATTTTGTCAGCAGTCATGAATATCCTGCTGTTCATGTCGATTA

GATGGGCGATCTGTGTCTTAGTCTAAGCTTCCGCTTAACGCATCTTTGCTACTTTAATGT

ATTTTTTTAATGATATTGTTGGAGTTTTGAGCACATCTTAATCGCAAGTAGATATTTTTT

TTGAATCGGATTCGACTTCAAGCAGATGTACTTCTTTGTCTCTCGATAAATCTAATGACC

CTGCGAGCCGGCCTCTCACCTTAAACTAATTTCTGAGAGTTAGGTCGGCGCCTCTCGTGG

TTTCCATGTTCTATGGAAACGGCCGCTCGGGACTTGCCTTCCCGCAACTCATCGAGCTTG

CCTGGCCCGCTCGGGTGGTGGCACAAGCCATCGTTAAATCGTTCGTAATCCTCTACTCTC

GCATAAATGAGAGGGGAATCCAAAGATGATCACTTTTCAGCTAATTGGAATCTGCAATCC

CAAAAGAGGCAAGCACTATGTGGGGTCCACAACTTATAGAATTTTGGCCTAACCATTCTG

CATAATGGATCATGACATGGGCCATTTATGGCCACAAAAACTGGCACAAATTCCAACCTA

TTCAGCACAATGAACAAGTTCTTCCAATTGGAATAAAGGAGGTTAAAATTATTCGAGTAG

ACATGACGTGCTGGCACGCCACTAGCCTCGCATTGATGCCACTCTTTCTTGCGCACACTT

ATATAATGCTGAGCTAATAATAGAGATAAAAATAGTCAGCTTGTAAACTGGACCTTTATT

ACTGCCCCTAATCTCAAGATCAAAGCGACATATACCTCTTCTCTTTCAAGTCTTTTTGCA

ACTTGTTCCAAAGCAAGCGTTTGTGAATCTGCCAGGCAGCCTTCACGAAAATGGCTGATC

AACTGTGTGAGGGGCGTTGGTCCGAAGCCAATCTATCATTCGTTGGGATATCCATCACCC

CTGAATTCATCGATGAGGCCCTCAGAGGTTTTTGGGAGTCTTTTGCCAAAAGAGCCGTTG

GCTACAAAGAGGTTTACGTTCAGGTTGATCGAGATCCTAAAGTTGCCTGGGAAGGAGCGC

TCAATGGTTACCTCGGCAAATATTCTGGCGTAAAAAATGTTCATTTTGCGCCGAACAACG

CCACGATTGGAAAGCCCCCACGCCATATCGACATTTTGCTTCGGAAAAAATTTAGCGGCG

AACTTCTTGCAACGCTCGAAGGTGTGGCAATTTCAAACGGTGCAACTGGTTGGCTTGCGC

TCGCATGTGTAAACCTTCTCTTGAAGGCAAGACAAGATATTGATGTCTTCTTGTTTGGGG

CAGGAAAAGTCACCGAGGCTGTGATTCTTGCCCTCAATCATGGTGCGGCTGCAAAAATCA

AAACCATGGCAGTGCTCAGCCAAGCTCCAATTGAAGGTGGGGTTCCAGCTCGATGCCATC

ATTGATCGGAAGGTTATACCAAAAGCCAAACTCGTCATAATAGCAACAAATTCTGAGGAG

CTCGGTACTCGAGGCCGACGAAATTGCGCCAAATGCAGTAACCATATCCCTCGGAAAAGA

TGAATTGCCCGCCGCCTACTTCGATCGCCTTTTGAACGCAGAGGGTCTGATTATTGGCGA

CGATCTGGATGCGATCGAATCGCGCAATGTCAATTCCCTGGTGCTTTACTACTCGAAACG

TGATTTGAAGCTGACAGAACATGGAACGGATCATTGGATAAAGAACTACGCCAATGTCCT

TGCTGATCCAGCTCTCATGGAGGAACTTAAGACATGAGAGGGGCCAGCCAACTTTTCATC

GGTTGGCCTTGCCAGCCTAGACTTGGCGATGGCCGGCCGACTCTACGAAACTCTTACTGC

GAAACTCTCCCACCCCCAGTAGACAAAACCCTCTTAGGATTGCGCTCTTCAGAAGGTGCG

CCTCCCCAGTCACAACCGGAAAGTCACTCAACTTGGCTCAAGTACCCTACTTAGTTTGTC

TCTCTGTTGTGTTTAGTATCAGCAACAATAACAAATATTGTAAATAACATATATTTATAT

GGTAATTATATGGCATGTAAGCGTGACAACGATTATGTTTTCCATAGTTTGATAAACTCA

ACACCCGAAAAAGCCGGTGACCAAAGGGTTGGGACCTCTTTGCACGAACTCCTATGGCTG

CCAAACTATCTACAGAGCTTTGACGCAGCATCTGATCGCCACACAATCATCGCGGACGTG

GATGGCGTGGCTACATGCAAGCAGTTTGACTGAGAGTTTTCGAGTGACTATTTTAAAAGG

ACAATATTTCGTCCCAAGCGATAAAACTCGCTCACATGACTAAGTCGTTGCTTTAATTCA

ACTGCGTGAAAAGATTTAAAGGGCAGAACTGTTTGTGTTTCGTATCCAGTTCACCGGCAA

TTATTGGCTACGCATGAAGCTCATTATCGTCGATTCTTTACCCAAACACTGATGCCATTC

CAAATGAGTTGGGCCAAAGATGTACTCATGTCGGACAGGCGCGATTTGGACTTCGTGATC

CTCAAGTCTATCGCAGTGCTGCTTTATTACAGGCTATGCTTCCACCCTCCATTATTTGGT

CAACTGGACCTAGCTTTTGACAAGAATAGGCCAGCGTTCATCTATCTATCTTTCAAAAAC

ATGCAGTTGCTGCGTAAAGGATGTAACATCGGATTTGGTGTGGAGACGGTGCTCGCTACA

ACTATACTGCCTTACTGTAGTGATCTGTCCCGCCAAGCGATGAGGGGAGCGGCCTGCCTC

TCGTGAGCGTCAATCCATACGGTTGCATGTTCGTTTGTTTCAAAGGCATTCCCAAGAAGG

GTTTCCAGCTTGACAACTTTCTCATCGGTGGGTGTGAACTCGCCGGTTAGTGAGTCACCG

CCCATTTTGGGAACTACCAGGGGGGTGCCAACGACTTCCTCCGTCGACATCTCAGACCAA

TTTCAATTGCCCAACTGCACCGTTCTTTCTATGTTGAAGYCATGGGCGACTATGAAATCT

CCATCTTTGGTCGGTACTACCTCGATCTCGACCATATTGAAAAGCCTGAAAGATTTGCAA

ACTGCCCCTACACTGTTTTCCGGCAAGTGTGGCCCATGGAATCCACGGTGAACTATCAAT

TGGCGGTCGCTTGAAGTCTTTCGAGCGATAAGCCAATTGGCGAGGCAGTGTTTCGCGATC

GCATCTTTAAGAGGAAAGTCCGATGCAAATCTCCATGTAGACCCAGTCGTTTCGGCCAAG

GCGGCCTCTAAATCAACTTGAGCAGCAGTTGTGTTAACGGAATCTGTTTTGACTGCCATT

CTGTAAGAAATGTCAGCAAAGGAAAAGCTAGTTAGAGAAGGAGAGCTTGCACAATAGTGT

GTCTATTCTCAAATGAACTATACTAGCGCAATGAGTTGGTGTTGTTCGCTGATGGAAAAA

AGGAAACGCATCCCCCTCTTCTACAAAATTATGTGGGCCCGGAGGGACTCCGTATGAAAG

TGCACAGCATGTGGACGCATCACCATGCAATTAGCAAGCTTAGCCAATGTCGTGTCTGTC

AGAGTGTGCAGCATGTGAGCCAAGCGCAATTACAATAAAAAAATAATATAAAAACTCTGC

CGAGGTCCGAACCGTCTCAAATGCTTTGAGGGCCATCCAAGCCAAGCGCACCTAACGCCT

AGCACACCATTATTGCGCCGCTCGAGCATTAATGGTTTGGGWCTTGGTGTCATCTGGAAA

CCTAWGAAGCATGGACACGGCAAAATCAGCGTTGTGGCGG

>tr3958_Un

ATTTCCCTCACCTTGAAGTATCAATTTGATCTAGAAGCCAATCCAAACTCATTGAAAGGC

CTGTCATCAACCTGTCAACACGGTTCAATGTTTGCTCGAGTGGCGCAGCAATGGTGTGCT

AGGCGTTAGGTGCGCCTGGCTGGGATGGCCCTCAAAGCATTTGAGACAGTTCGGACCTCG

GCAGAGATTTYATATTATTTTTTTGTTGTAATCGCGTTTGGCCCACATGCTGCACACTCC

GACAGACACGGCATTGGCTAAACCCGCTAATTGCGTGGTGATGCGTCCACGTGCTGCGCA

CTTTCATGCGGAGTCCCCCCGAGCCCGTAGAATTTTGTAGAAGAGGGGGACGCGTTTCCT

TTTTCCCATCAGCAAACAACACCAACTCACTATGCTAGTATAGTTCATCTGAGAACAGAC

ACACTGCTGAGCAAGCTCTCCTTCTCCAACTAGCTTTTTCTTTGCTGACATTTCCTACAA

AATGGCAGCAAAACAGATTCCGTTAACACAACTGCTGCTCAGGTTGATTTAGAGGCCRCC

TTGGCCGAAGCGACCGGGTCTACATGGAGATTTGCATCAAACTTTCCTCTTWAAGATGCG

ATCGCAAAACACTGCCTCGCCAATTGGCTTATCGCTCGAAAGACTTCAAGCAACCACYAA

TTGATAGTTCACCGTGAATTCCATGGGCCACACCTGCCGGAAAACAGYGTAGGGGCAGTC

TGCAAATCTCTCAGACTTTTTAATATGGTCGAGATCAACGTAGTACCGACCAAAGATAGA

GATTTCATAGTCGTCCACGACTTCAACACAGAAAGAACGACGCAGTTAGGCAATTGAAAT

TGGTCTGATATGTCGACGAAAGAGGCCGTTGGCACCCCCCTAGTAGTTCCCATAATGGGC

GGTGACTCCCTAACCAGCGAGTTCACACCCACCGATGAGAAAGTTGTCAAGCAAGAAACC

CTTCTCAGGAATGCCTTTGAAACAAACAAACAGGCAACCATACGGATTGACGCTCACGAG

AGGCAGGCCGCTCCCCTCGTCGCTTGGCTGAGCCATAGGCCGGCCTATCACGACAAGACA

ATCGTTATCTTTCATACATATGGRTACAACAATGGACACAAGTTCGCCTTGGCGATCGAT

CAAGCCGACGCTACAGAAAATTGGCGCGAAACTGTAGCGCTAATGCCAAACATTCTGCCA

AATGAACTCTCTAGGCTCGCGAAGCAATATCCACCAAATATCACGTCGCATTTTTACGAC

CTCGTTGAGGCCGGCAAGAATTGGTTCRCRTCTGTCTTCTCCCAGAATATGATGGTGGAC

GTCACTTTCCTCAAGATTGCGGGCACTGGCGAAGGACAGACTAACAAAGTCGTTGATCYA

AATGGAAATGAGGTTCTGCATCCTAAAGAGAAGGAGGAATATCTCCGCGATTCAGCTTCA

ATTGAAATCGCGAAATGAATCTGCGCCACCCATCCCGGAATCGAGATTGGAGGCGTCACA

CGAACTTATGCTTATAAGAGTCGAGGACATCGCCACAAAATCGATCTTTGGACGGGTCAA

CCGAAGCGGTGGCCGACCCACGCGACGAAGTATATCTGGGAAGATTTCGCCAAGCCTGGA

AATGCGCTCAGATGGGGTCAAACGATTATCTCTGATCGCATTGGCGACGACCTTGCAGCT

TGTGTGCTTCAAAGCCGTGGAGAAAATTTTGTCAGCAGTCATGAATATCCCGCTGTTCAT

GTCGATTAGATGGGCGATCTGTGTCTTAGTCTAAGCTTCCGCTTAACGCATCTTTGCTAC

TTTAATGTATTTTTTTAATGATATTGTTGGAGTTTTCAGCACATCTTAATCGCAAGTAGA

TATTTTTTTTGAATCGGATTCGACTTCAAGCAGATGTACTTCTTTGTCTCTCGATAAATC

TAATGACCCTGCGAGCCGGCCTTTCGCCTTAAACTAATTTCTGAGAGTTAGGTCGGCGCC

TCTCGTGGTTTCCATGTTCTATGGAAACGGCCTCTCGGGACTTGCCTTCCCGCAACTCAT

CGAGCTTGCCTGGCCCGCTCGGGTGGTGGCGCAAGCCATCGTTAAATCGTTCGTAATCCT

CTACTCTCGCATAAATGAGAGGGGAATCCAAAGATGATCACTTTTCAGCTAATTGGAATC

TGCAATCCTAAAAGAGGCAAGCACTATGTGGGGTCCACAACTTATAGAATTTTGGCCTAA

CCATTCTGCATAATGGATCATGACATGGGCCATTTATGGCCGCAAAAACTGGCACAAATT

CCAACCTGTTCAGCACAATGAACAAGTTCTTCCAATTGGAATAAAGGAGGTTAAAATTAT

TCGAGTAGACATGATGTGCCGGCACGCCACTAGCCTCGCATTGATGCCACCCTTTCTTGC

GCACACTTATATAATGCTGAGCTAATAATAGAGATAAAAATAGTCAGCTTGTAAACTGGA

CCTTTACTACTGCCCCTAATCTCAAGATCAAAGCGACATATACCTCCTCTCTTTCAAGTC

TWTTTGCAACTTGTTCCAAAGCAAGCGTTCGTGARTCTGCCAGGCAGCCTTCACGAAAAT

GGCTGATCAACTATGTGAGGGGCGTTGGTCCGAAGCCAATCTATCATTCGTTGGGATATC

CATCACCCTTGAATTCATCGATGAGGCCCTCARAGGCTTTTGCCAAAAGAGCCGTTGGCT

ACAAAGAGGTTTACGTTCGGGTTGATCGAGATCCTAAAGWTGCCTGGGRAGGAGCGCTCA

ATGGTTACCTCGGCAAATATTCTGGCGTCAAAAATGTTCATTTTGCGCCGAACAACGCCG

CGATTGGAAAGCCCCCATGCCATATCGACATTTTGCTTCGGAAAAAATTTAGCGGCGAAT

TTCTTGTGACGCTCGAAGGTGTGGCAATTTCAAACGGTGCAACTGGTTGGCTTGCGCTCG

CGTGCGTAAACCTTCTCTTGAAGGCAAGACAAGATATTGATGTCTTCTTGTTTGGGGCGG

GAAAAGTCGYCGAGGCTGTGRTTCTTGCCCTCAATCACGGTGCGGCTGCAAAAATCAAAC

CATGGCAGTGCTCAGCCAAGCTCCAATCGAAGGTGGGGTTCCAGCTCGATGTCGTCATTG

ATCGGAAGGTTATACCAAAAGCCAAACTCGTCATAACAGCAACAAATTCCGAGGAGCTCG

CACTCGAGGCCGACGAAATTGCGCCAAATGCAGTAACCATATCCCTCGGAAAAGATGAAT

TGCTTGCCGCCTACTTCGATCGCCTTTTGAACGCAGAGGGTCTGATTATCGGCGACGATC

TGGATGCGATCGAATCGCGCAATGTCAATTCCCTGGTGCTTTACTACTCGAAACGTGATT

TGAAGCTGACAGAACATGGAAGGGATCATTGGATAAAAAACTACGCCAATGTCCTTGTTG

ATCCAGCTCTCATGGAGGAACTTAAGACATGGGAGGGGCCAGCCAACTTTTCATCGGTTG

GCCTTGCCAGCCTAGACTTGGCGATGGCCGGCCGACTCTACGAAACTCTTACTGCGAAAC

TCTCCCACCCCCAGTAGACAAGACCCTCTTAGGATTGCGCTCTTCAGAAGGTGCGCCTCC

CCAGTCACAACCGGAAAGTCACTCAACTTGGCTCAAGTACCCTACTTAGTTTGTCTCTCT

GTCGTGTTTAGTATCAACAACAATAACAAATATTGTAAACAACATATATTTATATGGTAA

TTATATGGCATGTAAGCGTGACAACGATTATGTTTTCCATAGTTTGATAAACTCAACACC

CGAAAAAGCTGGTGACCAAAGGGTTGGGACCTCTTTGCACGAACTCCTATGGCTGCCAGA

CTATCTACAGAGCTTTTTCGAAAGCTGACGCAGCATCTGATCGCTACACAATCCTCGCGG

ACGTGGATGGCGTGGCTACACGCAAGCAGTTTGACTGAGAGTTTTCGAGTGACTATTTTA

AAAGGACGATATTTCGTCCCAAGCAATAAAACTCGCTCACATGACTAAGTCGTTGCTTTA

ATTCAGCTGCGTGAAAAGATTTAAAGGGCAGAACTGTTTGTGTTTCGTATCCAGTTCACC

GGCAATTATTGGCTACGCATGAAGCTCATTATCGTCGATTCTTTACCCAAACACTGATGT

CATTCCAAATGAGTTGGGCCAAAGATGTACTCATGTCGGACAGGCGCGATTTGGACTTCG

TGWTCCTCRAGTCTATCGCAGTGCTGCTTTATTACAGGCTATGCTTCCACCCTCCATTAT

TTGGTCAACTGGACCTGGCTTTYGACAAGAATAGGCCAGCGTTCATCTATCTATCTTTCA

AAAACATGCAGTTGCTGCTGCGTAAAGGATGTAACATCGGATTTGGTGTGGAGACGGTGC

TTGCTACGACTATACTGCCTTACTRTAGTGATCTGTCCTGCCAAGCGACGAGGGGAGCAG

CCTGCCTCTCGTGAGCGTCAATCCATACAGTTGCATGTTCGTTTGTTTCAAAGGCATTCC

CAAGAAGGGTTTCCAGCTTGACAACTTTCTCATCGGTGGGTGTGAACTAGCCGGTTAGTG

AGTCACCGCCCATTTTGGGAACTACCAGGGGGGTGCCAACGAYCTCCTCCGTYGACATCT

CAGACCAATTTCAATTGCTCAACTGCGCCGTTCTTTCTATGTTGAAGTCATGGGCGACTA

TGAAATCTCCATCTTTGGTCGGTACTACCTCGATCTCGACCATATTGAAAAGCCTGAGAG

ATTTGCAAATTGCCCCTACACTGTTTTCCGGCAAGTGTGGCCCATGGAATCCACGGKGWA

CTATCAATTGGCGGTCGCTKGAAGTCTTTCGRGCGATAAGCCAATTGGCGAGGCAGTGTT

TCGCGATCGCATCTTTAAGAGGAAAGTCCGATGCAAATCTCCATGTAGACCCGGTTGCTT

CGGCCAAGGCGGCCTCTAAACCAACTTGAGCAGCAGTTGTGTTAACGGAATCTATTTTGA

CTACCATTTTGTAAGAAATGTCAGCAAAGGAAAAGCTAGTTAGAGAAGGAGAGCTTGCTC

AATAGTGTGTCTATTCTCATATGAACTATACTAGCGYAATGAGTTAGTGTTGTTCGCTGA

TGGGAAAAAGGAAATGCATCCCCCTCTTCTACAAAATTCTGTGGGCCCGGAGGGACTCCG

TATGAAAGTGCACAGCATGTGGACGCATCACCATGCAATTAGCGAGCTTAGCCAATGCCG

TGTCTGTCAGAGTGTGCAGCATGTGGGCCAAGCGCAATTACAATAAAAAAATAATATAAA

AACTCTGCCGAGGTCTGAACCGTCTCAAATGCTTTGAGGGCCATCCAAGCCAAGCGCACC

TAACGCCTAGCACACCATTGTTGCGCCGCTCGAGCATTAATGGTCCGGGACCTGGTGTCA

TCTGGAAACCTATGAAGCATGGACACGGCAAAATCAGCGCCGTGGCGGTGTCARACACGG

GGACACGTMSGGGACACGTMMRKSACACGTCATCTACCGT

>tr3966_Un

CAATTTGATCTAGAAGCCAATCCAAACTCATTGAAAGGCCTGTCATCAACCTGTCAACAC

GGTTCAATGTTTGCTCGAGTGGCGCAGCAATGGTGTGCTAGGCGTTAGGTGCGCCTGGCT

GGGATGGCCCTCAAAGCATTTGAGACAGTTCGGACCTCGGCAGAGATTTTATATTATTTT

TTTGTTGTAATCGCGTTTGGCCCACATGCTGCACACTCCGACAGACACGGCATTGGCTAA

ACCCGCTAATTGCGTGGTGATGCGTCCACGTGCTGCGCACTTTCATGCGGAGTCCCCCCG

AGCCCGTAGAATTTTGTAGAAGAGGGGGACGCRTTTCCTTTTTCCCATCAGCAAACAACA

MCAACTCACTATGCTAGTATAGTTCATCTGAGAACAGACACACTGCTGAGCAAGCTCTCC

TTCTCCAACTAGCTTTTTCTTTGCTGACATTTCCTACAAAATGGCAGCAAAACAGATTCC

GTTAACACAACTGCTGCTCAGGTTGATTTAGAGGCCGCCTTGGCCGAAGCGACCGGGTCT

ACATGGAGATTTGCATCAAACTTTCCTCTTAAAGATGCGATCGCAARACACTGYCTCGCC

AATTGGCTTATCGCTCGAAAGACTTCAAGCAACCACCAATTGATAGTTCACCGTGAATTC

CATGGGCCACACCTACCGGAAAACAGCGTAGGGGCAGTCTGCAAATCTCTCAGACTTTTT

AATATGGTCGAGATCAACGTAGTACCGACCAAAGATAGAGATTTCATAGTCGTCCACGAC

TTCAACACAGAAAGAACGACGCAGTTAGGCAATTGAAATTGGTCTGATATGTCGACRAAA

GAGGCCGTTGGCACCCCCCTAGTAGTTCCCATAATGGGCGGTGACTCCCTAACCAGCGAG

TTCACACCCACCGATGAGAAAGTTGTCAAGCAAGAAACCCTTCTCAGGAATGCCTTTGAA

ACAAACAAACAGGCAACCATACGGATTGACGCTCACGAGAGGCAGGCCGCTCCCCTCGTC

GCTTGGCTGAGCCATAGGCCGGCCTATCACGACAAGACAATCGTTATCTTTCATACATAT

GGATACAACAATGGACACAAGTTCGCCTTGGCGATCGATCAAGCCGACGCTACAGAAAAT

TGGCGCGAAACTGTAGCGCTAATGCCAAACATTCTGCCAAATGAACTCTCTAGGCTCGCG

AAGCAATATCCACCAAATATCACGTCGCATTTTTACGACCTCGTTGAGGCCGGCAAGAAT

TGGTTCGCGTCTGTCTTCTCCCAGAATATGATGGTGGACGTCACTTTCCTCAAGATTGCG

GGCACTGGCGAAGGACAGACTAACAAAGTCGTTGATCCAAATGGAAATGAGGTTCTGCAT

CCTAAAGAGAAGGAGGAATATCTCCGCGATTCAGCTTCAATTGAAATCGCGAAATGAATC

TGCGCCACCCATCCCGGAATCGAGATTGGAGGCGTCACACGAACTTATGCTTATAAGAGT

YGAGGACATCGCCACAAAATCGATCTTTGGACGGGTCAACCGAAGCGGTGGCCGACCCAC

GCGACGAAGTATATCTGGGAAGATTTCGCCAAGCCTGGAAATGCGCTCAGATGGGGTCAA

ACGATTATCTCTGATCGCATTGGCGACGACCTTGCAGCTTGTGTGCTTCAAAGCCGTGGA

GAAAATTTTGTCAGCAGTCATGAATATCCCGCTGTTCATGTCGATTAGATGGGCGATCTG

TGTCTTAGTCTAAGCTTCCGCTTAACGCATCTTTGCTACTTTAATGTATTTTTTTAATGA

TATTGTTGGAGTTTTCAGCACATCTTAATCGCAAGTAGATATTTTTTTTGAATCGGATTC

GACTTCAAGCAGATGTACTTCTTTGTCTCTCGATAAATCTAATGACCCTGCGAGCCGGCC

TTTCGCCTTAAACTAATTTCTGAGAGTTAGGTCGGCGCCTCTCGTGGTTTCCATGTTCTA

TGGAAACGGCCTCTCGGGACTTGCCTTCCCGCAACTCATCGAGCTTGCCTGGCCCGCTCG

GGTGGTGGCGCAAGCCATCGTTAAATCGTTCGTAATCCTCTACTCTCGCATAAATGAGAG

GGGAATCCAAAGATGATCACTTTTCAGCTAATTGGAATCTGCAATCCTAAAAGAGGCAAG

CACTATGTGGGGTCCACAACTTATAGAATTTTGGCCTAACCATTCTGCATAATGGATCAT

GACATGGGCCATTTATGGCCGCAAAAACTGGCACAAATTCCAACCTGTTCAGCACAATGA

ACAAGTTCTTCCAATTGGAATAAAGGAGGTTAAAATTATTCGAGTAGACATGATGTGCCG

GCACGCCACTAGCCTCGCATTGATGCCACCCTTTCTTGCGCACACTTATATAATGCTGAG

CTAATAATAGAGATAAAAATAGTCAGCTTGTAAACTGGACCTTTACTACTGCCCCTAATC

TCAAGATCAAAGCGACATATACCTCCTCTCTTTCAAGTCTTTTTGCAACTTGTTCCAAAG

CAAGCGTTCGTGAATCTGCCAGGCAGCCTTCACGAAAATGGCTGATCAACTATGTGAGGG

GCGTTGGTCCGAAGCCAATCTATCATTCGTTGGGATATCCATCACCCCTGAATTCATCGA

TGAGGCCCTCAGAGGCTTTTGCCAAAAGAGCCGTTGGCTACAAAGAGGTTTACGTTCGGG

TTGATCGAGATCCTAAAGTTGCYTGGGAAGGAGCGCTCAATGGTTACCTCGGCAAATATT

CTGGCGTCAAAAATGTTCATTTTGCGCCGAACAACGCCGCGATTGGAAAGCCCCCATGCC

ATATCGACATTTTGCTTCGGAAAAAATTTAGCGGCGAATTTCTTGTGACGCTCGAAGGTG

TGGCAATTTCAAACGGTGCAACTGGKTGGCTTGCGCTCGCGTGCGTAAACCTTCTCTTGA

AGGCAAGACAAGATATTGATGTCTTCTTGTTTGGGGCGGGAAAAGTCGCCGAGGCTGTGA

TTCTTGCCCTCAATCACGGTGCGGCTGCAAAAATCAAACCATGGCAGTGCTCAGCCAAGC

TCCAATCGAAGGTGGGGTTCCAGCTCGATGTCGTCATTGATCGGAAGGTTATACCAAAAG

CCAAACTCGTCATAACAGCAACAAWTTCCGAGGAGCTCGCACTCGAGGCCGACGAAATTG

CGCCAAATGCAGTAACCATATCCCTCGGAAAAGATGAATTGCTTGCCGCCTACTTCGATC

GCCTTTTGAACGCAGAGGGTCTGATTATCGGCGACGATCTGGATGCGATCGAATCGCGCA

ATGTCAATTCCCTGGTGCTTTACTACTCGAAACGTGATTTGAAGCTGACAGAACATGGAA

GGGATCATTGGATAAAAAACTACGCCAATGTCCTTGTTGATCCAGCTCTCATGGAGGAAC

TTAAGACATGGGAGGGGCCAGCCAACTTTTCATCGGTTGGCCTTGCCAGCCTAGACTTGG

CGATGGCCGGCCGACTCTACGAAACTCTTACTGCGAAACTCTCCCACCCCCAGTAGACAA

GACCCTCTTAGGATTGCGCTCTTCAGAAGGTGCGCCTCCCCAGTCACAACCGGAAAGTCA

CTCAACTTGGCTCAAGTACCCTACTTAGTTTGTCTCTCTGTCGTGTTTAGTATCAACAAC

AATAACAAATATTGTAAACAACATATATTTATATGGTAATTATATGGCATGTAAGCGTGA

CAACGATTATGTTTTCCATAGTTTGATAAACTCAACACCCGAAAAAGCTGGTGACCAAAG

GGTTGGGACCTCTTTGCACGAACTCCTATGGCTGCCAGACTATCTACAGAGCTTTTTCGA

AAGCTGACGCAGCATCTGATCGCTACACAATCCTCGCGGACGTGGATGGCGTGGCTACAC

GCAAGCAGTTTGACTGAGAGTTTTCGAGTGACTATTTTAAAAGGACGATATTTCGTCCCA

AGCAATAAAACTCGCTCACATGACTAAGTCGTTGCTTTAATTCAGCTGCGTGAAAAGATT

TAAAGGGCAGAACTGTTTGTGTTTCGTATCCAGTTCACCGGCAATTATTGGCTACGCATG

AAGCTCATTATCGTCGATTCTTTACCCAAACACTGATGTCATTCCAAATGAGTTGGGCCA

AAGATGTACTCATGTCGGACAGGCGCGATTTGGACTTCGTGATCCTCAAGTCTATCGCAG

TGCTRCTTTATTACAGGCTATGCTTCCACCCTCCATTATTTGGTCAACTGGACCTGGCTT

TTGACAAGAATAGGCCAGCGTTCATCTATCTATCTTTCAAAAACATGCAGTTGCTGCTGC

GTAAAGGATGTAACATCGGATTTGGTGTGGAGACGGTGCTTGCTACGACTATACTGCCTT

ACTGTAGTGATCTGTCCTGCCAAGCGACGAGGGGAGCAGCCTGCCTCTCGTGAGCGTCAA

TCCATACAGTTGCATGTTCGTTTGTTTCAAAGGCATTCCCAAGAAGGGTTTCCAGCTTGA

CAACTTTCTCATCGGTGGGTGTGAACTAGCCGGTTAGTGAGTCACCGCCCATTTTGGGAA

CTACCAGGGGGGTGCCAACGACCTCCTCCGTCGACATCTCAGACCAATTTCAATTGCTCA

ACTGCGCCGTTCTTTCTATGTTGAAGTCATGGGCGACTATGAAATCTCCATCTTTGGTCG

GTACTACCTCGATCTCGACCATATTGAAAAGCCTGAGAGATTTGCAAATTGCCCCTACAC

TGTTTTCCGGCAAGTGTGGCCCATGGAATYCACGGTGAACTATCAATTGGCGGTCGCTTG

AAGTCTTTCGAGCGATAAGCCAATTGGCGAGGCAGTGTTTCGCGATCGCATCTTTAAGAG

GAAAGTCCGATGCAAATCTCCATGTAGACCCGGTTGCTTCGGCCAAGGCGGCCTCTAAAC

CAACTTGAGCAGCAGTTGTGTTAACGGAATCTATTTTGACTACCATTTTGTAAGAAATGT

CAGCAAAGGAAAAGCTAGTTAGAGAAGGAGAGCTTGCTCAATAGTGTGTCTATTCTCATA

TGAACTATACTAGCGCAATGAGTTAGTGTTGTTCGCTGATGGGAAAAAGGAAATGCATCC

CCCTCTTCTACAAAATTCTGTGGGCCCGGAGGGACTCCGTATGAAAGTGCACAGCATGTG

GACGCATCACCATGCAATTAGCGAGCTTAGCCAATGCCGTGTCTGTCAGAGTGTGCAGCA

TGTGGGCCAAGCGCAATTACAATAAAAAAATAATATAAAAACTCTGCCGAGGTCTGAACC

GTCTCAAATGCTTTGAGGGCCATCCAAGCCAAGCGCACCTAACGCCTAGCACACCATTGT

TGCGCCGCTCGAGCATTAATGGTCCGGGACCTGGTGTCATCTGGAAACCTATGAAGCATG

GACACGGCAAAATCAGCGCCGTGGCGGTGTCAGACACGGGGWCACGTACGGGACACG

>tr3970_2

aGCCTCTCCAAATTTCCCTCACCTTGAAGTATCAATTTGATCCAGAAGCCAATCCAAACT

CATTGAAAGGCCTGTCATCAACCTGTCAACACGGTTCAATGTTGCTCGAGCGGCGCAGCA

ATGGTGTGCTAGGCGTTAGGTGCGCCTGGCTTGGATGGCCCTCAAAGCATTTGAGACAGT

TCGGACCTCGGCAGAGATTTTATATTATTTTTTTGTTGTAATCGCGCTTGGCCCACATGC

TGCACACTCCGACAGACACGGCATTGGCTAAGCCCGCTAATTGCGTGATGATGCGTTCAC

GTGCTGCGCACTTTCATGYGGAGTCCCCCCGAGCCCGTAGAATTTgagggggacgccttt

cctttttcccatcagcaaacaacaccaactcactgtgctagtatagttcatctGAGAACA

GACACACTGCTGAGCAAGCTCTCCTTCTCCAACTAGCTTTTTCTTTGCTGACATTTCCTA

CAGAATGGCAACCAAAACAGATTCCGTTAACACAACTGCTGCTCAGGTTGATTTGGAGGC

CGCCTTGGCCGAAGTGACCGGGTCTACATGAAGATTTGCATCAGACTTTCCTCTTAAAGA

TGCGATCGCAAAACACTGCCTCGCCAACTGGCTTATCGCTCGAAAGACTTCAAGCAACCA

CCAATTGATAGTTCACCGTGAATTCCATGGACCACACCTGCCGGAAAACAGCGTAGGGGC

AGTCTGCAAATCTCTCAGACTTTTTAATATGGTCGAGATCGACGTAGCACCAACCAAAGA

TAGAGATTTCATAGTCGTCCACGACTTCAACACAGAAAGAATGACGCAGTTAGGCAATCG

AAATTGGTCTGATATGTCGACGAAGGAGGCCGTTGACACCCCCCTGGAAGTTCCCAAAAT

GGGCGGTGACTCCCTAACCAGCGAGTTCACATCCACCGATGAGAAAGTTGTCAAGCAGGA

AACCCTTCTTAGGAATGCCTTTGAAACAAACTAACAGGCAACCATATGGATTGACGCTCA

CGAGAGGCAGGCCGCTCCCCTCGTCGCTTGGCTGAGCCATAGGCCAGCCTATCACGACAA

GACAATCATTATCTTTTATACATATGGATACAACAATGGACACGAGTTCGCCTTGGCGGT

CGATCAAGCCGGCACTATGGAAAATTGGCGCGAAACTATAGCGCTAATGCCAAACATTCT

GCCGGATGAACTCTCTAGGCTCGCGAAGCAATATCCACCAAATTGCATGTCGCATTTTTA

CGACCTTGTTGCGGCTGGCAAGAATTGGTTCGCGGCTGTCTTCTCCCAGAATATGATGGT

GGTCGTCACTTTCCTCAAGATTGCGGGCACTGGCGAAGGACAAACTAACAAAGTCGTTGA

TCCAAATGGAAATGAGGTTCTACATCCTAAAGAGAAGGAGGAATATCTCCGCAATTCAAC

TTCAATCGAAATCGCGAAATGAATTTGCGCCACCCATCCCAGAATCGAGATTGGAGGCGT

CACACGAACTTATGCTTATCAGAGTCGAGGACATCGTTACAAAACCGATCTTTGGACGGG

TCAGCCGAAGTGGTGGCCGACCCACGCGACGAAGTATATCTGGGAGGATTTCGCCAAGCC

TGGAAATGCGCTCAGATGGGGTCAAACGATTATCTCTGATCGCATTGGCGACGACCTTGC

AGCTTGTGTGCTTCAAAGCCGTGGAGAAAATTTTGTCAGCAGTCATAAATATCCCACTAT

TCATGTCGATTAGATGGGCGATCTGTGTCTTGGTCTAAGCTTCCGCTTAACGCAtCTTTG

CTACTTTAATGTATTTTtttaatgatattgttggagttttcagcacatcttaatcgcaag

tAGATATTTTTTTTGAATCGAATTCAATTTCAAGCAGATGTACTTCTTTGTCTCTCGATA

AATCTAATGACCCTGCGAGCCGGCCTCTCGCCTTAAACTAATTTCTGAGAGTTAGGCCGA

TGCCTCTCGTGGTTTCCATGTTCTATGGAAACGGCCTCTCGGGACTTGCCTTCCTGCAAC

TCATCGAGCTTGCCTGCTCGGGTGGTGGCGCAAGCCATCGTTAAATCATTCGTAATCCTT

TACTCTCGCATAAATGAGAGGGGAATCCAAAGATGATCACTTTTCAGCTAATTGGAATCT

GCAATCCCAAAAGTGGCAAGCACTATGTGGGGTCCACAACTTATAGAATTCTGGCCTAAC

CATTCTGCATAATGGATCATGACATGGGCCATTTATGGCCGCAAAAACTGGCACAAATTC

CAACCTGTTCAGCACAATGAACAAGTTCTTCCAATTGGAATAAGGAGGTTAAAATTATTC

GAGTAGACATGACGTGTCGGCACGCCACTAGCCTCGCATTCATGCCACCCTTTCTTGCGC

ACACTTATATAATGCTGAGCTAATAATAGAGATAAAAATAGTCAGCTTGTAAACTGGACC

TTTACTACTGCCCCTAATCTCAAGATCAAAGCGACATATACCTCCTCTCTTTCAAGTCTT

TTTGCAACTTGTTCCAAAGCAAGCGTTCGTGAATCTGCCAGGCAGCCTACACGAAAATGG

CCGATCAACTGTGTGAGGGGCGTTAGTCCGAAGCCGATCTATCATTCGTTGGGATATCCA

TCACCCCTGAATTCATCGATGAGGCCCTCAGAGGCTTTTGGGAGTCTTTTGCCAAAAGAG

CCGTTGGCTACAAAGAGGTTTACGTTCAGGTTGATCGAGATCCTAAAGTTGCCTGGGAAG

GAGTGCTCAATGGTTACCTCGACAAATATTCTGGCATCAAAAATGTTCATTTTGCGCCGA

ACAATGCCGCGATTGGAAAGCCCCCACACCATATCGACATTTTGCTTCGGAAAAAATTCG

GCGGCGAACTTCTTGCGACGCTCGAAGGTGTGGCAATTTCAAACGGTGCAACTGGTTGAC

TTGCGCTCGCATGCGTAAACCTTCTCTTGAAGGCAAGACAAGATATTGATGTCTTCTTGT

TTGGGGCGGGAAAAGTCGCCGAGGCTGTGATTCTTGCCCTCAATCATGGTGCAGCTGCAA

AAATCAAAACCATGGCAGTGCTCAGCCAAGCTCCAATCGAAGGTGGGGTTCCAGCTCGAT

GCCGTCGATGATCGGAAGGTTATACCAAAAGCCAAACTCGTCATAACAGCAACAAATTCC

AAGGAGCTCGTACTCGAGGCCGACGAAATTGCGCCAAATGCAATAACCATATCCCTCGGA

AAAGATGAATTGCCCGCCGTCTACTTCGATCGCCTTTTGAACGCAGAGGGTCTGATTATC

GGCGACGATCTGGATGCGATCGAATCGCGCAATGTCAATTCCCTGGTGCTTTACTACTCG

AAACGTGATTTGAAGCTGACAGAACATGGAAGGGATCATCGGATAAAGAACTACGCCAAT

GTCCTTGCCGATCCAGCTCTCATGGAAAAACTTAAGACATGGGAGGGGCCAGCCAACTTT

TCATCGGTTGGCCTTGCCAACCTAGACTTGGCAATGGCCGGCCGACTCTACGAAACTCTT

ACTGCGAAACTCTCCGACCCCCAGTAGACAAGACCCTCTTAGGATTGCGCTCTTCAGAAG

GTGCGCCTCCCCAGTCACAACCGGAAAGTCACTCAACTTGGCTCAAGTACCCTACTTAGT

TTGTCTCTCTTTCGTGTTTTGTATCAGCAACAATAACAAATATTGTAAATAACATATATT

TATATGGTAATTATATGGCATGTAAGCGTGACAACGATTATGTTTTCCATAGTTTGCTAA

ACTCAACACCCGAAAAAGCTGGTGACCAAAGGGTTGGCACCTCTTTGCACGAACTCCTAT

GGCTGCCAGACTATCTACAGAGCTTTTTCGAAAGCTAATGTAGCATCTGATCGCCACACA

ATCCTCGCGGACGTGGATGGCGTGGCTACACGCAAGTAGTTTGACTGAGAGTTTTCGAGT

GACTATTTTAAAAGGACAATATTTCGTCCCAAGCGATAAAACTCGCTCACATGACTAAGT

CGTTGCTTTAATTCGGCTACGTGAAAAGATTTAAAGGGCAAAACTATTTGTGTTTCGTTT

CCAGTTCACCGGCAATTATCAGCTACGCATGAAGCTCATTATCGTCGATTCTTTACCCAA

ACACTGATGCCATTCCAAATGAGTTGGGCCAAAGATGTACTCTTGTCGGACAGGCACGAT

TTGGACTTCGTGATCCTCAAGTCTATCGCAGTACTGCTTTATTACAGGCTATGCTTCCAC

CCTCCATTATTTGGTCAACTGGACCTGGCTTTTGACGAGAATAGGCCAGCGTTCATCTAT

CTATCTTTCAAAAACATGTAGTTGCTGCTGCGTAAAGGATGTAACATCGGATTTGGTGTG

GAGACGGTGCTCGCTACGACTATATTGCCTTACTGTAGTGATCTGTCCCGCCAAGCGACG

AGGGGAGCGGCCTGCCTCTCGTGAGCGTCAATCCATACGATTGCATGTTCGTTTGTTTCA

AAGGCATTCCCAAGAAGGGTTTCCAGCTTGACAACTTTCTCATTGGTGGATGTGAACTCG

CCGGTTAGGGAGTCACCGCCTATTTTGGAAACTACCAGGAGGGTGCCAACGACCTCCTCC

GTCGACATCTCAGACCAATTTCAATTGCCCAACTGCGCCGTTCTTTCTATGTTGAAGTCA

TGGGCGACTATGAAATCTCTATCTTTGTTCGGTACTACGTCGATCTCGACCATATTGAAA

AGCCTGAGAGATTTGCAAACTGCCCCTACACTGTTTTCCGACAGGTGTGACCCATGGAAT

CCATGGTGAACTATCAATTGGAGGTCGCTTGAAGTCTTTCGAGCGATAAGCCAACTGGCG

AGGCAGTGTTTCGCGATCGCAGCTTTAAGAGGAAAGTCCGATGCAAATCTCCATGTAGAC

ACGGTCGCTTCGGCCAAGGCGGCCTCTAAATCAACTTGAGCAACAGTTGTGTTAACGGAA

TCTGTTTTGGTTGCCATTCTGTAGGAAATGTCAGCAAAGGAAAAGCTAGTTAGAGAATGA

GAGCTTGCTCAACAGTGTGTTTATTCTCAGATGAACTATACTAGCGCAATGAGTTGGTGT

TGTTCGCTGATGGGAAAAAGGAAATGCATGCCCCTCTTCTACAAAATTATGTGGGCCCAG

AGGGACTCCGTATGAAAATGCACAGCATGTGGATGCATCACCATGCAATTAGCGAGCTTA

GCCAATGCCGTGTCTGTCAGAGTGTACAGCATGTGGGCCAAGCGCAATTACAATAAAAAA

ATAATATAAAATCTCTGCCGAGGTCtgaaccgtctcaaatgctttgagggccatccaagc

caagcgcacctaacgcctagcacaccattgttgcgccgctcaagcattaatggtccggga

cctggtgtca

>tr3970_1

gaagtatcaatTTGATCTAGAAGCCAATCCAAACTCATTGAAAGGCCTGTCATCAACCTG

TCAACACGGTTCAATGTTTGCTCGAGTGGCGCAGCAATGGTGTGCTAGGCGTTAGGTGCG

CCTGGCTGGGATGGCCCTCAAAGCATTTGAGACAGTTCGGACCTCGGCAGAGATTTTATA

TTATTTTTTTGTTGTAATCGCGTTTGGCCCACATGCTGCACACTCCGACAGACACGGCAT

TGGCTAAACCCGCTAATTGCGTGGTGATGCGTCCACGTGCTGCGCACTTTCATGTGGAGT

CCCCCGAGCCCGTAGAATTTTGTAGAAGAGGGGGACGCCTTTCCTTTTTCCCATCAGCAA

ACAACACCAACTCACTGTGCTAGTATAGTTCATCTGAGAACAGGCACACTGCTGAGCAAG

CTCTCCTTCTCCAACTAGCTTTTTCTTTGCTGACATTTCCTACAGAATGGCAGCAAAACA

GATTCCGTTAACACAACTGCTGCTCAAGTTGATTTAGAGGCCGCCTTGGCCGAAGCGACC

GGGTCTACATGGAGATTTGCATCAAACTTTCCTCTTAAAGATGCGATCGCAAAACACTGC

CTCGCCAATTGGCTTATCGCTCGAAAGACTTCAAGCAACCACCAATTGATAGTTCACCGT

GAATTCCATGGGCCACACCTGCCGGAAAACAGCGTAGGGGCAGTCTGCAAATCTCTCAGA

CTTTTTAATATGGTCGAGATCAACGTAGTACCGACCAAAGATAGAGATTTCATAGTCATC

CACGACTTCAACACAGAAAGAACGACGCAGTTAGACAATTGAAATTGGTCTGATATGTCA

ACGAAAGAGGCCATTGGCACCCCCCTAGTAGTTCCCATAATGGGCGGTGACTCTCTAATC

AGCAAGTTCACACCCACCGATGAGAAAGTTGTCAAGCAAGAAACCCTTCTCAGGAATGCC

TTTGAAACAAACAAACAGGCAACCATACGGATTGACGCTCACGAGAGGCAGGCCGCTCCC

CTCGTCGCTTGGCTGAGCCATAGGCCGGCCTATCACGACAAGACAATCGTAATCTTTCAT

ACATATGGATACAACAATGGACACAAGTTCGCCTTGGCAGTCGATCAAGCCGACGCTACA

GAAAATTGGCGCGAAACTGTAGCGCTAATGCCAAACATTCTGCCGAATGAACTCTCTAGG

CTCGCGAAGCAATATCCACCAAATATCACGTCGCATTTTTACGACCTCGTTGAGGCCGGC

AAGAATTGGTTCGCGTCTGTCTTCTCCCAGAATATGATGGTGGTCGTCACTTTCCTCAAG

ATTGTGGGCACTGGCGAAGGACAGACTAACAAAGTCGTTGATCCAAATGGAAATGAGGTT

CTGCATCCTAAAGAGAAGGAGGAATATCTCCGCGATTCAGCTTCAATCGAAATCGCGAAA

TGAATCTGCGCCACCCATCCCGGAATCGAGATTGGAGGCGTCACACGAACTTATGCTTAT

AAGAGTCGAGGACACCGCTACAAAATCGATCTTTGGACAGGTCAACCGAAGCGGTGGCCG

ACCCACGCGACGAAGTATATCTGGGAAGATTTCGCCAAGCCTGGAAACGCGCTCAGATGG

GGTCAAACGATTATCTCTGATCGCATTGGCGACGACCTTGCAGCTTGTGTGCTTCAAAGC

CGTGGAGAAAATTTTGTCAGCAGTCATGAATATCCCGCTGTTCATGTCGATTAGATGGGC

GATCTGTGTCTTAGTCTAAGCTTCCGCTTAACGCATCTTTGCTACTTTAATGTATTTTTT

TAATGATATTGTTGGAGTTTTCAGCACATCTTAATCGCAAGTAGATATTTTTTTTGAATC

GGATTCGACTTCAAGCAGATGTACTTCTTTGTCTCTCGATAAATCTAATGACCCTGCGAG

CCGGCCTCTCGCCTTAAACTAATTTCTGAGAGTTAGGTCGGCGCCTCTCGTGGTTTCCAT

GTTCTATGGAAACGGCCTCTCGGGACTTACCTTCCCGTAACTCATCGAGCTTGCCTGGCC

CGCTCGGGTGGTGGCGCAAGCCATCGTTAAATCGTTCGTAATCCTCTACTCTCGCATAAA

TGAGAGGGGAATCCAAATATGATCACTTTTCAGCTAATTGGAATCTGCAATCCTAAAAGA

GGCAAGCACTATGTGGGGTCCACAACTTATAGAATTTTGGCCTAACCATTCTGCATAATG

GATCATGACATGGGCCATTTATGGCCGCAAAAACTGGCACAAATTCCAACCTGTTCAGCA

CAATGAACAAGTTCTTCCAATTGGAATAAAGGAGGTTAAAATTATTCGAGTAGACATGAT

GTGCCGGCACGCCACTAGCCTCGCATTGATGCCACCCTTTCTTGCGCACACTTATATAAT

GCTGAGCTAATAATAGAGATAAAAATAGTCAGCTTGTAAACTGGACCTTTACTACTGCCC

CTAATCTCAAGATCAAAGCGACATATACCTCCTCTCTTTCAAGTCTTTTTGCAACTTGTT

CCAAAGCAAGCGTTCGTGAATCTGCTAGGCAGCCTTCACGAAAATGGCTGATCAACTATG

TGAGGGGCGTTGGTCCGAAGCCAATCTATCATTCGTTGGGATATCCATCACCCCTGAATT

CATCGATGAGGCCCTCAGAGGCTTTTGCCAAAAGAGCCGTTGGCTACAAAGAGGTTTACG

TTCGGGTTGATCGAGATCCTAAAGTTGCCTGGGAAGGAGCGCTCAATGGTTACCTCGGCA

AATATTCTGGTGTCAAAAATGTTCATTTTGCGCCGAACAACGCCGCGATTGGAAAGCCCC

CACGCCATATCGACATTTTGCTTCGGAAAAAATTTAGCGGCGAATTTCTTGCGACGCTCG

AAGGTGTGGCAATTTCAAACGGTGCAACTGGTTGGCTTGCGCTCGCATGCGTAAACCTTC

TCTTGAAGGCAAGACAAGATATTGATGTCTTCTTGTTTGGGGCGGGAAAAGTCGCCGAGG

CTGTGATTCTTGCCCTCAATCACGGTGCGGCTGCAAAAATCAAAACCATGGCAGTGCTCA

GCCAAGCTTCAATCGAAGGTGGGGTTCCAACTCGATGTCGTCATTGATCAAAAGGTTATA

CCAAAAGCCAAACTCGTCATAACAGCAACAAATTCCGAGGAGCTCGTACTCGAGGCCGAC

GAAATTGCGCCAAATGCAGTAACCATATCCCTCGGAAAAGATGAATTGCTCGCCGCCTAC

TTCGATCGCCTTTTGAACGCAGAGGGTCTGATTATCGGCGACGATCTGGATGCGATCGAA

TCGCGCAATGTCAATTCCCTGGTGCTTTACTACTTGAAACGTGATTTGAAGCTGACAGAA

CATGGAAGGGATCATTAGATAAAGAACTACGCCAATGTCCTTGTTGATCCAGCTCTCATG

GAGGAACTTAAGACATGGGAGGGGCCAGCCAACTTTTCATCGGTTGGCCTTGCCAGCCTA

GACTTGGCGATGGCCGGCCGACTCTACGAAACTCTTACTGCGAAACTCTCCCACCCCCAG

TAGACAAGACCCGCTTAGGATTGCGCTCTTCAGAAGGTGCGCCTCCCCAGTCACAACCGG

AAAGTCACTCAACTTGGCTCAAGTACCCTACTTAGTTTGTCTCTCTGTCGTGTTTAGTAT

CAACAACAATAACAAATATTGTAAACAACATATATTTATATGGTAATTATATGGCATGTA

AGCGTGACAACGATTATGTTTTCCATAGTTTGATAAACTCAACACCTGAAAAAGCCGGTG

ACCAAAGGGTTGGGACCTCTTTGCACGAACTCCTATGGCTGCCAGACTATCTACAGAGCT

TTTTCGGAAGCTGACGCAGCATCTGATCGCTACACAATCCTCGCGGACGTGGATGGCGTG

GCTACACGCAAGCAGTTTGACTGAGAGTTTTCGAGTGACTATTTTAAAAGGACGATATTT

CGTCCCDAGCAATAAAACTCGCTCACATGACTAAGTCGTTGCTTTAATTCAGCTGCGTGA

AAAGATTTAAAGGGCAGAACTGTTTGtGTTTCGTATCCAGTTCACCGGCAATTATTGGCT

ACGCATGAAGCTCATTATCGTCGATTCTTTACCCAAACACTGATGTCATTCCAAATGAGT

TGGGCCAAAGATGTACTCATGTCGGACAGGCGCGATTTGGACTTCGTGATCCTCAAGTCT

ATCGCAGTGCTGCTTTATTACAGGCTATGCTTCCACCCTCCATTATTTGGTCAACTGGAC

CTGGCTTTTGACAAGAATAGGCCAGCGTTCATCTGTCTATCTTTCAAAAACATGCAGTTG

CTGCTGCGTAAAGGATGTAACATCGGATTTGGTGTGGAGACGGTGCTTGCTACGACTATA

CTGCCTTACTGTAGTGATCTGTCCTGCCAAGCGACGAGGGGAGCAGCCTGCCTCTCGTGA

GCGTCAATCCATACGGTTGCATGTTCGTTTGTTTCAAAGGCATTCCCAAGAAGGGTTTCC

AGCTTGACAACTTTCTCATTGGTGGGTGTGAACTCGCCGGTTAGTGAGTCACCGCCCATT

TTGGGAACTACCAGGGGGGTGCCAACGACCTCCTCCGTCGACATCTCAGACCAATTTCAA

TTGCTCAACTGCGCCGTTCTTTCTATGTTGAAGTCATGGGCGACTATGAAATCTCCATCT

TTGGTCGGTACTACCTCGATCTCGACCATATTGAAAAGCCTGAGAGATTTGCAAACTGCC

CCTACACTGTTTTCCAGCAAGTGTGGCCCATGGAATCCACGGTGAACTATCAATTGGCGG

TCGCTTGAAGTCTTTCGAGCGATAAGCCAATTGGCGAGGCAGTGTTTCGCGATCGCATCT

TTAAGAGGAAAGTCCGATGCAAATCTCCATGTAGACCCGGTCGCTTCGGCCAAGGCGGCC

TCTAAACCAACTTGAGCAGCAGTTGTGTTAATGGAATCTATTTTGACTGCCATTCTGTAA

GAAATGTCAGCAAAGGAAAAGCTAGTTAGAGAAGGAGAGCTTGCTCAATAGTGTGTCTAT

TCTCATATGAACTATACTAGCGTAATGAGTTAGTGTTGTTCGCTGATGGGAAAAAGGAAA

TGCATCCCCCTCTTCTACAAAATTCTGTGGGCCCGGGGGGACTCCGTATGAAAGTGCACA

GCATGTGGACGCATCACCATGCAATTAGCGAGCTTAGCCAATGCCGTGTCTGTCAGAGTG

TGCAGCATGTGGGCCAAGCGCAATTACAATAAAAAAATAATATAAAAACTCTACCGAGGT

CTGAACCGTCTCAAATGCTTTGAGGGCCATCCAAGCCAAGCGCACCTAACGCCTAGCACA

CCATTGTTGCGCCGCTCAAGCATTAATGGTCCGGGACCTGGTGTCATCTGGAAACCTATG

AAGCATGGACACGgcaaaatcagcg

>tr3982_2

tctccaaatttccctcaccttgaagtatcaatttgatctagaagccaatccaaactcatt

gaaaggcctgtcatcaacctgtcaacacggttcaatgtttgctcgagtggcgcagCAATG

GTGTGCTAGGCGTTAGGTGCGCCTGGCTGGGATGGCCCTCAAAGCATTTGAGACAGTTCG

GACCTCGGCAGAGATTTTATATTATTTTTTTGTTGTAATCGCGTTTGGCCCACATGCTGC

ACACTCCGACAGACACGGCATTGGCTAAACCCGCTAATTGCGTGGTAATGCGTCCACGTG

CTGCGCACTTTCATGCGGAGTCCCCCCGAGCCCGTAGAATTTTGTAGAAGAGGGGGACGC

GTTTCCTTTTTCCCATCAGCAAACAACACCAACTCACTGTGCTAGTATAGTTCATCTGAG

AACAGACACACTGCTGAGCAAGCTCTCCTTCTCCAACTAGCTTTTTCTTTGCTGACATTT

CCTACAGAATGGCAGCAAAACAGATTCCGTTAACACAATTGCTGCTCAGGTTGATTTAGA

GGCCGCCTTGGCCGAAGCGACCGGGTCTACATGGAGATTTGCATCAAACTTTCCTCTTAA

AGATGCGATCGCAAAACACTGCCTCGCCAATTGGCTTATCGCTCGAAAGACTTCAAGCAA

CCACCAATTGATAGTTCACCGTGAATTCCATGGGCCACACCTGCCGGAAAACAGCGTAGG

GGCAGTCTGCAAATCTCTCAGACTTTTTAATATGGTCGAGATCAACGTAGTACCGACCAA

AGATAGAGATTTCATAGTCATCCACGACTTCAACACAGAAAGAACGACGCAGTTAGGCAA

TTGAAATTGGTCTGATATGTCGACGAAAGAGGCCGTTGGCACCCCCCTAGTAGTTCCCAT

AATGGGCGGTGACTCCCTAACCAGCGAGTTCACACCCACCGATGAGAAAGTTGTCAAGCA

AGAAACCCTTCTCAGGAATGCCTTTGAAACAAACAAACAAGCAACCATACGGATTGACGC

TCACGAGAGGCAGGCCACTCCCCTCGTCGCTTGGCTGAGCCATAGGCCGGCCTATCACGA

CAAGACAATCGTTATCTTTCATACATATGGATACAACAATGGACACAAGTTCGCCTTGGC

GGTCGATCAAGCCGACGCTACAGAAAATTGGTGCGAAACTGTAGCGCTAATGCCAAACAT

TCTGCCGAATGAACTCTCTAGGCTCGCGAAGCAATATCCACCAAATATCACGTCGCATTT

TTACGACCTCGTTGAGGCTGGCAAGAATTGGTTCGCGTCTGTCTTCTCCCAGAATATGAT

GGTGGTCATCACTTTCCTCAAGATTGCGGGCACTGGCGAAGGACAGACTAACAAAGTCGT

TGATCCAAATGGAAATGAGGTTCTGCATCCTAAAGAGAAGGAGGAATATCTCCGCGATTC

AGCTTCAATCGAAATCGCGAAATGAATCTGCGCCACCCATCCCGGAATCGAGATTGGAGG

CGTCACACGAACTTATGCTTATAAGAGTCGAGGACATCGCTACAAAATCGATCTTTGGAC

GGGTCAACCGAAGCGGTGGCCGACCCACGCGACGAAGTATATCTGGGAAGATTTCGCCAA

GCCTGGAAATGCGCTCAGATGGGGTCAAACGATTATCTCTGATCGCATTGGCGACGACCT

TGTAGCTTGTGTGCTTCAAAGCCGTGGAGAAAATTTTGTCAGCAGTCATGAATATCCCGC

TGTTCATGTCGATTAGATGGGCGATCTGTGTCTTAGTCTAAGCTTCCGCTTAATGCATCT

TTGCTACTTTAATGTATTTTTTTAATGATATTGTTAGAGTTTTCAGCACATCTTAATCGC

AAGTAGATATTTTTTTTGAATCGGATTCGACTTCAAGCAGATGTACTTCTTTGTCTCTCG

ATAAATCTAATGACCCTGCGAGCCggcctctcgccttaaactaatttctgagagttaggt

cggcgcctctcgtggtttccatgttctatggaaacggcctctcgggtcttgccttcccgc

aacTCATCGAGcTTGCCTGGCCCGCcCgGgtggtggcGCAAGCCATCGTTAAATCGTTCG

TAATCCTCTACTCTCGCATAAATGAGAGGGGAATCCAAAGATGATCACTTTTCAGCTAAT

TGGAATCTGCAATCCTAAAAGAGGCAAGCACTATGTGGGGTCCACAACTTATAGAATTTT

GGCCTAACCATTCTACATAATGGATCATGACATGGGCCATTTATGGCCGCAAAAACTGGC

ACAAATTCCAACCTGTTCAGCACAATGAACAAGGTCTTCCAATTGGAATAAAGGAGGTTA

AAATTATTCGAGTAGACATGATGTGCCGGCACGCCACTAGCCTCGCATTGATGCCACCCT

TTCTTGCACACACTTATATAATGCTGAGCTAATAATAGAGATAAAAATAGTCAGCTTGTA

AACTGGACCTTTACTACTGCCCCTAATCTCAAGATCAAAGCGACATATACCTCCTCTCTT

TCAAGTCTTTTTGCAACTTGTTCCAAAGCAAGCGTTCGTGAATCTGCCAGGCAGCCTTCA

CGAAAATGGCTGATCAACTATGTGAGGGGCGTTGGTCCGAAGCCAATCTATCATTCGTTG

GGATATCCATCACCCCTGAATTCATCGATGAGGCCCTCAGAGGCTTTTGCCAAAAGAGCC

GTTGGCTACAAAGAGGTTTACGTTCGGGTTGATCGAGATCCTAAAGTTGCCTGGGAAGGA

GCACTCAATGGTTACCTCGGCAAATATTCTGGCGTCAAAAATGTTCATTTTGCGCCGAAC

AACGCCGCGATGGGAAAGCCCCCACGCCATATCGACATTTTGCTTCGGAAAAAATTTAGC

GGCGAATTTCTTGCAACGCTCGAAGGTGTGGCAATTTCAAACGGTGCAACTGGTTGGCTT

GCGCTCGGATGCGTAAACCTTCTCTTGAAGGCAAGACAAGATATTGATGTCTTCTTGTTT

GGGGCGGGAAAAGTCGCCGAGGCTGTGATTCTTGCCCTCAATCACGGTGCGGCTGCAAAA

ATCAAAACCATGGCAGTGCTCAGCCAAGCTCCAATCGAAGATGGGGTTCCAGCTCGATGT

CGTCATTGATCGGAAGGTTATACCAAAAGCCAAACTCGTCATAACAACAACAAATTCCGA

GGAGCTCGTACTCGAGGCCGACGAAATTGCGCCAAATGCAGTAACCATATCCCTCGGAAA

AGATGAATTGCTCACTGCCTACTTCGATTGCCTTTTGAACGTAGAGGGTCTGATTATCGG

CGACGATCTGGATGCGATCGAATCGCGCAATGTCAATTCCCTGGTGCTTTACTACTCGAA

ACGTGATTTGAAGCTAACAGAACATGGAAGGGATCATTAGATAAAGAACTACGCCAATGT

CCTTGTTGATCCAGCTCTCATGGAGGAACTTAAGACATGGGAGGGGCCAGCCAACTTTTC

ATCGGTTGGCCTTGCCAGCCTAGACTTGGCGATGGCCGGCCGACTCTATGAAACTCTTAC

TGCGAAACTCTCCCACCCCCAGTAGACAAGACCCTCTTAGGATTGCGCTCTTCAGAAGGT

GCGCCTCCCCAGTCACAACCGGAAAGTCACTCAACTTGGCTCAAGTACCCTACTTAGTTT

GTCTCTCTGTCGTGTTTAGTATCAACAACAATAACAAATATTGTAAACAACATATATTTA

TATGGTAATTATATGGCATGTAAGCGTGACAACGATTATGCTTTCCATAGTTTGATAAAC

TCAACACCCGAAAAAGCCAGTGACCAAAGGGTTGGGACCTCTTTGCACGAACTCCTATGG

CTGCCAGACTATCTACAGAGCTTTTTCGGAAGCTGACGCAGCATCTGATCGCTACACAAT

CCTCGCGGACGTGGATGGCATGGCTACACGCAAGCAGTTTGACTGAGAGTTTTCGAGTGA

CTATTTTAAAAGGACGATATTTCGTCCCAAGCAATAAAACTCGCTCACATGACTAAGTCG

TTGCTTTAATTCAGCTGCGTGAAAAGATTTAAAGGGCAGAACTGTTTGTGTTTCGTATCC

AGTTCACCGGTAATTATTGGCTACGCATGAAGCTCATTATCGTCGATTCTTTACCCAAAC

ACTGATGTCATTCCAAATGAGTTGGGCCAAAGATGTACTCATGTCGGACAGGCGCGATTT

GGACTTCGTGATCCTCAAGTCTATCACAGTGCTGCTTTATTACAGGCTATGCTTCCACCC

TCCATTATTTGGTCAACTGGACCTGGCTTTTGACAAGAATAGGCCAGCGTTTATCTATCT

ATCTTTCAAAAACATGCAATTGCTGCTGCGTAAAGGATGTAACATCGGATTTGGTGTGGA

GACGGTGCTTGCTACGACTATACTGCCTTACTGTAGTGATCTGTCCCGCCAAGCGACGAG

GGGAGCAGCCTGCCTCTCGTGAGCGTCAATCCATACGGTTGCATGTTCGTTTGTTTCAAA

GGCATTCCCAAGAAGGGTTTCCAGCTTGACAACTTTCTCATCGGTGGATGTGAACTCGCC

GGTTAGTGAGTCACCGCCCATTTTGGGAACTACCAGGGGGGTGCCAACGACCTCCTCCGT

CGACATCTCAGACCAATTTCAATTGCTCAACTGCGCCTTTCTTTCTATGTTGAAGTCATG

GGCGACTATGAAATCTCCATCTTTGGTCGGTACTACCTCGATCTCGACCATATTGAAAAG

CCTGAGAGATTTGCAAACTGCCCCTACACTGTTTTCCGGCAAGTGTGGCCCATGGAATCC

ACGGTGAATTATCAATTGGCGGTCGCTTTAAGTCTTTCGAGCGATAAGCCAATTGGCGAG

GCAGTGTTTCGTGATCGCATCTTTAAGAGGAAAGTCTGATGCAAATCTCCATGTAGACCC

GGTCGCTTCGGCCAAGGTGGCCTCTAAACCAACTTGAGCAGCAGTTGTGTTAACGAAATC

TATTTTGACTGCCATTCTATAAGAAATGTCAGCAAAGGAAAAGCTAGTTAGAGAAGGAGA

GCTTGCTCAATAGTGTGTCTATTCTCATATGAACTATACTAGCGCAATGAGTTAGTGTTG

TTCGCTGATGGGAAAAAGGAAATGCATCCCCCTCTTCTACAAAATTCTGTGGGCCCGGAG

GGACTCCGTATGAAAATGCACAGCATGTGGACGCATCACCATGCAATTAGCGAGCTTAGC

CAATGTCGTGTCTATCAGAGTGTGCAGCATGTGGGCCAAGCGCAATTACAATAAAAAAAT

AATATAAAAACTCTGCCGAGGTCTGAACCGTCTCAAATGCtttgagggccatccaAGCCA

AGCGCACCTAACGCCTAGCACACCATTGTTGCGCCGCTCGAGCATTAATGGTCCGGGACC

TGGTGTCATCTGGAAACCTATGAAGCATGGACACGGCAAAATCAGCGCCGTGGCGGTGTT

AGACACGGGGACACGTACGGGACACgtmmk

>tr3982_1

tggccctcaaagcatttgagacagttcGGACCTCGGCAGAGATTTTATATTATTTTTTTG

TTGTAATCGCGTTTGGCCCACATGCTGCACACTCCGACAGACACGGCATTGGCTAAACCC

GCTAATTGCGTGGTGATGCGTCCACGTGCTGCGCACTTTCATGCGGAGTCCCCCCGAGCC

CGTAGAATTTTGTAGAAGAGGGGGACGCGTTTCCTTTTTCCCATCAGCAAACAACACCAA

CTCACTGTGCTAGTATAGTTCATCTGAGAACAGACACACTGCTGAGCAAGCTCTCCTTCT

CCAACTAGCTTTTTCTTTGCTGACATTTCCTACAAAATGGCAGCAAAACAGATTCCGTTA

ACACAACTGCTGCTCAGGTTGATTTAGAGGCCGCCTTGGCCGAAGCGACCGGGTCTACAT

GGAGATTTGCATCAAGCTTTCCTCTTAAAGATGCGATCGCAAAACACTGCCTCGCCAATT

GGCTTATCGCTCGAAAGACTTCAAGCAACCACCAATTGATAGTTCACCGTGAATTCCATG

GGCCACACCTGCCGGAAAACAGCGTAGGGGCAGTCTGCAAATCTCTCAGACTTTTTAATA

TGGTCGAGATCAACGTAGTACCGACCAAAGATAGAGATTTCATAGTCGTCCACGACTTCA

ACACAGAAAGAACGACGCAGTTAGGCAATTGAAATTGGTCTGATATGTCGACGAAAGAGG

CCGTTGGCACCCCCCTAGTAGTTCCCATAATGGGCGGTGACTCCCTAACCAGCGAGTTCA

CACCCACCGATGAGAAAGTTGTCAAGCAAGAAACCCTTCTCAGGAATGCCTTTGAAACAA

ACAAACAGGCAACCATACGGATTGACGCTCACGAGAGGCAGGCCGCTCCCCTCGTCGCTT

GGCTGAGCCATAGGCCGGCCTATCACGACAAGACAATCGTTATCTTTCATACATATGGAT

ACAACAATGGACACAAGTTCGCCTTGGCGGTCGATCAAGCCGATGCTACAAAAAATTGGC

GCGAAACTGTAGCGCTAATGCCAAACATTCTGCCAAATGAACTCTCTAGGCTCGCGAAGC

AATATCCACCAAATATCACGTCGCATTTTTACGACCTCGTTGAGGCCGGCAAGAATTGGT

TCGCGTCTGTCTTCTCCCAGAATATGATAGTGGACGTCACTTTCCTCAAGATTGCGGGCA

CTGGCGAAGGACAGACTAACAAAGTCGTTGATCCAAATGGAAATGAGGTTCTGCATCCTA

AAGAGAAGGAGGAATATCTCCGCGATTCAGCTTCAATTGAAATCGCGAAATGAATCTGCG

CCACCCATCCCGGAATCGAGATTGGAGGCGTCACACGAACTTATGCTTATAAGAGTCGAG

GACATCGCCACAAAATCGATCTTTGGACGGGTCAACCGAAGCGGTGGCCGACACACGCGA

CGAAGTATATCTGGGAAGATTTCGCCAAGCCTGGAAATGCGCTCAGATGGGGTCAAACGA

TTATCTCTGATCGCATTGGCGACGACCTTGCAGCTTGTGTGCTTCAAAGCCGTggagaaa

attttgtcagcagtcatgaatatcccgctgttcatgtcgaTTAGATGGGCGATCTGTGTC

TTAGTCTAAGCTTCCGCTTAACGCATCTTTGCTACTTTAATGTATTTTTTTAATGATATT

GTTGGAGTTTTCAGCACATCTTAATCGCAAGTAGATATTTTTTTTGAATCGGATTCGACT

TCAAGCAGATGTACTTCTTTGTCTCTCGATAAATCTAATGACCCTGCGAGCCGGCCTTTC

GCCTTAAACTAATTTCTGAGAGTTAGGTCGGCGCCTCTCGTGGTTTCCATGTTCTATGGA

AACGGCCTCTCGGGACTTGCCTTCCCGCAACTCATCGAGCTTGCCTGGCCCGCTCGGGTG

GTGGCGCAAGCCATCGTTAAATCGTTCGTAATCCTCTACTCTCGCATAAATGAGAGGGGA

ATCCAAAGATGATCACTTTTCAGCTAATTGGAATCTGCAATCCTAAAAGAGGCAAGCACT

ATGTGGGGTCCACAACTTATAGAATTTTGGCCTAACCATTCTGCATAATGGATCATGACA

TGGGCCATTTATGGCCGCAAAAACTGGCACAAATTCCAACCTGTTCAGCACAATGAACAA

GTTCTTCCAATTGGAATAAAGGAGGTTAAAATTATTCGAGTAGACATGATGTGCCGGCAC

GCCACTAGCCTCGCATTGATGCCACCCTTTCTTGCGCACACTTATATAATGCTGAGCTAA

TAATAGAGATAAAAATAGTCAGCTTGTAAACTGGACCATTACTACTGCCCCTAATCTCAA

AGATCAAAGCGACATATACCTCCTCTCTTTCAAGTCTTTTTGCAACTTGTTCCAAAGCAA

GCGTTCGTGAATCTGCCAGGCAGCCTTAACGAAAATGGCTGATCAACTATGTGAGGGGCG

TTGGTCCGAAGCCAATCTATCATTCGTTGGGATATCCATCACCCTGAATTCATCGATGAG

GCCCTCAGAGGCTTTTGCCAAAAGAGCCGTTGGCTACAAAGAGGTTTACGTTCGGGTTGA

TCGAGATCCTAAAGTTGCCTGGGAAGGAGCGCTCAATGGTTACCTCGGCAAATATTCTGG

CGTCAAAAATGTTCATTTTGCGCCGAACAACGCCGCGATTGGAAAGCCCCCATGCCATAT

CGACATTTTGCTTCGGAAAAAATTTAGCGGCGAATTTCTTGTGACGCTCGAAGGTGTGGC

AATTTCAAACGGTGCAACTGGTTGGCTTGCGCTCGCGTGCGTAAACCTTCTCTTGAAGGC

AAGACAAGATATTGATGTCTTCTTGTtTGGGGCGGGAAAAGTCGCCGAGGCTGTGATTCT

TGCCCTCAATCACGGTGCGGCTGCAAAAATCAAAACCATGGCAGTGCTCAGCCAAGCTCC

AATCGAAGGTGGGGTTCCAGCTCGATGTCGTCATTGATCGGAAGGTTATACCAAAAGCCA

AACTCGTCATAACAGCAACAAATTCCCAGGAGCTCGCACTCGAGGCCGACGAAATTGCGC

CAAATGCAGTAACCATATCCCTCGGAAAAGATGAATTGCTCGCCGCCTACTTCGATCGCC

TTTTGAACGCAGAGGGTCTGATTATCGGCGACGATCTGGATGCGATCGAATCGCGCAATG

TCAATTCCCTGGTGCTTTACTACTCGAAACGTGATTTGAAGCTGACAGAACATGGAAGGG

ATCATTGGATAAAAAACTACGCCAATGTCCTTGTTGATCCAGCTCTCATGGAGGAACTTA

AGACATGGGAGGGGCCAGCCAACTTTTCATCGGTTGGCCTTGCCAGCCTAGACTTGGCGA

TGGCCGGCCGACTCTACGAAACTCTTACTGCGAAACTCTCCCACCCCCAATAGACAAGAC

CCTCTTAGGATTGCGCTCTTCAGAAGGTGCGCCTCCCCAGTCACAACCGGAAAGTCACTC

AACTTGGCTCAAGTACCCTACTTAGTTTGTCTCTCTGTCGTGTTTAGTATCAACAACAAT

AACAAATATTGTAAACAACATATATTTATATGGTAATTATATGGCATGTAAGCGTGACAA

CGATTATGTTTTCCATAGTTTGATAAACTCAACACCCGAAAAAGCCGGTGACCAAAGGGT

TGGGACCTCTTTGCACGAACTCCTATGGCTGCCAGACTATCTACAGAGCTTTTTCGAAAG

CTGACGCAGCATCTGATCGCTACACAATCCTCGCGGACGTGGATGGCGTGGCTACACGCA

AGCAGTTTGACTGAGAGTTTTCGAGTGACTATTTTAAAAGGACGATATTTcgtcccaagc

aataaaactcgctcacatgactaagtcgttgcTTTAATTCAGCTGCGTGAAAAgatttaa

agggcagaactgtttgtgtttcgtatccagttcACCGGCAATTATTGGCTACGCATGAAG

CTCATTATCGTCGATTCTTTACCCAAACACTGATGTCATTCCAAATGAGTTGGGCCAAAG

ATGTACTCATGTCGGACAGGCGCGATTTGGACTTCGTGATCCTCAAGTCTATCGCAGTGC

TGCTTTATTACAGGCTATGCTTCCACCCTCCATTATTTGGTCAACTGGACCTGGCTTTTG

ACAAGAATAGGCCAGCGTTCATCTATCTATCTTTCAAAAACATGCAGTTGCTGCTGCGTA

AAGGATGTAACATCGGATTTGGTGTGGAGACGGTGCTTGCTACGACTATACTGCCTTACT

GTAGTGATCTGTCCTGCCAAGCGACGAGGGGAGCAGCCTGCCTCTCGTGAGCGTCAATCC

ATACAGTTGCATGTTCGTTTGTTTCAAAGGCATTCCCAAGAAGGGTTTCCAGCTTGACAA

CTTTCTCATCGGTGGGTGTGAACTAGCCGGTTAGTGAGTCACCGCCCATTTTGGGAACTA

CCAGGGGGGTGCCAACGACCTCCTCCGTCGACATCTCAGACCAATTTCAATTGCTCAACT

GCGCCGTTCTTTCTATGTTGAAGTCATGGGCGACTATGAAATCTCCATCTTTGGTCGGTA

CTACCTCGATCTCGACCATATTGAAAAGCCTGAGAGATTTGCAAATTGCCCCTACACTGT

TTTCCGGCAAGTGTGGCCCATGGAATCCACGGTGAACTATCAATTGGCGGTCGCTTGAAG

TCTTTCGAGCGATAAGCCAATTGGCGAGGCAGTGTTTCGCGATCGCATCTTTAAGAGGAA

AGTCCGATGCAAATCTCCATGTAGACCCGGTTGCTTCGGCCAAGGCGGCCTCTAAACCAA

CTTGAGCAGCAGTTGTGTTAACGGAATCTATTTTGACTACCATTTTGTAAGAAATGTCAG

CAAAGGAAAAGCTAGTTAGAGAAGGAGAGCTTGCTCAATAGTGTGTCTATTCTCATATGA

ACTATACTAGCGCAATGAGTTAGTGTTGTTCGCTGATGGGAAAAAGGAAATGCATCCCCC

TCTTCTACAAAATTCTGTGGGCCCGGAGGGACTCCGTATGAAAGTGCACAGCATGTGGAC

GCATCACCATGCAATTAGCGAGCTTAGCCAATGCCGTGTCTGTCAGAGTGTGCAGCATGT

GGGCCAAGCGCAATTACAATAAAAAAATAATATAAAAACTCTGCCGAGGTCTGAACCGTC

TCAAATGCTTTGAGGGCCATCCAAGCCAAGCGCACCTAACGCCTAGCACACCATTGTTGC

GCCGCTCGAGCATTAATGGTCCGGGACCTGGTGTCATCTGGAAACCTATGAAGCATGGAC

ACGGCAAAATCAGCGTCGTGGCGGTGTCAgacacggggacacgtcgg

>tr3981_2

ctctccaaatttccctcaccttgaagtatcaaTTTGATCTAGAAGCCAATCCAAACTCAT

TGAAAGGCCTGTCATCAACCGGTCAACACGGTTCAATGTTTGCTCGCGTGGCGCAGCAAT

GGTGTGCTAGGCATTAGGTGCGCCTGGCTTGGATGGCCCTCAAAGCATTTGAGGCAGTTC

GGACCTCGGCAGAGATTTTATATTATTTtTTTGTTGTAATCGCGCTTGGCCCACATGCTG

CACACTCCGATAGACACGGCATTGGCTAAACCCGCTAATTGCGTGGTGATGCGTCCACGT

GCTGCGCACTTTCATGCGGAGTCCCCCCGAGTCCATAGAATAAGAGGGGGACGCGTTTCC

TTTTTCCCATCAGCAAACAACACCAACTCACTGTGCTAGTATAGTTCATCTGAGAACAGA

CACACTGCTGAGCAAGCTCTCCTTCTCCAACTAGCTTTTTCTTTGCTGACATTTCCTACA

GAATGGCAGCAAAACAGATTCCGTTAACACAACTACTGCTCAGGTTGATTTAGAGGCCGC

CTTGGCCGAAGCGATCGGGTCTACATGGAGATTTGCATCAGACTTTCCTCTTAAAAATGC

AATCGCGAAACACTGCCTCGCCAATTGGCTTATCGCTCGAAAGACTTCAAGCAACCACCA

ATTGATAGTTCACCGTGAATTCCATGGGCCACACCTGCCCGAAAACAGCGTAGGGGCAGT

CTGCAAATCTCTCAGACTTTTTAATATGGTCGAGATCAACGTAGTACCGACCAAAGATAA

AGATTTCATAGTCGTCCACGACTTCAACACAGAAAGAACGACGCAGTTAGGCAATTGAAA

TTGGTTTGATATGTCGACGAAAGAGGCTGTTGGCACCCCCCTGGTAGTTCCCATAATGGG

CGGTGACTCCCTAACCAGCGAGTTCACACCCACCGATGAGAAAGTTGTCAAGCAAGAAAC

CCTTCTCAAGAATGCCTTTGAAACAAACAAACAGGCAACCATACGGATTGACGCTCACGA

GAGGCAGGCCGCTCCCCTCGTCGCTTGGCTGAGCCATAGGCCGGCCTATCACGACAAGAC

AATCGTTATCTTTCATACATATGGATACAACAATGGACACGAGTTCGCCTTGGCGGTCAA

TCAAGCCGGCGCTACAAAAAATTGGCGCGAAACTGTAGCGCTAATGCCAAACATTCTGCC

GAATGAACTCTCTAGGCTCGCGAAGCAATATCCACCAAATTGCACGTCGCATTTTTACGA

CCTCGTTGAGGCCAGCAAGAATTGGTTCGCATCTGTCTTCTCCCAGAATATGATGGTGGT

CGTCACTTTCCTCAAGATTGCGGGCACTAGCGAAGGACAGACTAACAAAGTCGTTGATCC

AAATGGAAATGAGGTTCTGCATCCTAAAGAGAAGGAGGAATATCTCCGCGATTCCGCTTC

AATCGAAATCGCGAAATGAATCTGCGCCACCCATCCCGGAATCGAGATTGGAGGCGTCAC

ACGAACTTATGCTTATAAGAGTCGAGGACATCGCTACAAAATCAATCTTTGGACGGGTCA

ACCGAAGCGGTGGCCGACCCACGCGACGAAGTACATCTGGGAAGATTTCGCCAAGCCTGG

AAATGCGCTCAGATGGGGTCAAACGATTATCTCTGATCGCATTGGTGACGACCTTGCAGC

TTGTGTGCTTCAAAGCCGTGGAGAAAATTTTGTCAGCAGTCATGAATATCCTGCTGTTCA

TGTCGATTAGATGGGCGATCTGTGTCTTAGTCTAAGCTTCTGCTTAACGCATCTTTGCTA

CTTTAATGTATTTTTTTAATGATATTGTTGGAGTTTTGAGCACATCTTAATCGCAAGTAG

ATATTTTTTTTGAATCGGATTCGACTTCAAGCAGATGTACTTCTTTGTCTCTCGATAAAT

CTAATGACCCTGCGAGCCGGCCTCTCACCTTAAACTAATTTCTGAGAGTTAGGTCGGCGC

CTCTCGTGGTTTCCATGTTTTATGGAAACGGCCGCTCGggacttgccttccCgCAACTcA

TCGAGCTTGCCTGGCCCGCTCGGGTGGTGGCACAAGCCATCGTTAAATCGTTCGTAATCC

TCTACTCTCGCATAAATGAGAGGGGAATCCAAAGATGATCACTTTTCAGCTAATTGGAAT

CTGCAATCCCAAAAGAGGCAAGCACTATGTGGGGTCCACAACTTATAGAATTTTGGCCTA

ACCATTCTGCATAATGGATCATGACATGGGCCATTTATGGCCGCAAAAACTGGCACAAAT

TCCAACCTATTCAGCACAATGAACAAGTTCTTCCAATTGGAATAAAGGAGGTTAAAATTA

TTCGAGTAGACATGACGTGCTGGCACGCCACTAGCCTCGCATTGATGCCACTCTTTCTTG

CGCACACTTATATAATGCTGAGCTAATAATAGAGATAAAAATAGTCCGCTTGTAAACTGG

ACCTTTATTACTGCCCCTAATCTCAAGATCAAAGCGACATATACCTCTTCTCTTTCAAGT

CTTTTTGCAACTTGTTCCAAAGCAAGCGTTTGTGAATCTGCCAGGCAGCCTTCACGAAAA

TGGCTGATCAACTGTGTGAGGGGCGTTGGTCCGAAGCCAATCTATCATTCGTTGGGATAT

CTATCACCCCTGAATTCATCGATGAGGCCCTCAGAGGCTTTTGGGAGTCTTTTGCCAAAA

GAGCCGTTGGCTACAAAGAGGTTTACGTTCAGGTTGATCGAGATCCTAAAGTTGCCTGGG

AAGGAGCGCTCAATGGTTACCTCGGCAAATATTCTGGCGTAAAAAATTTTCATTTTGCGC

CGAACAACGCCACGATTGGAAAGCCCCCACGCCATATCGACATTTTGCTTCGGAAAAAAT

TTAGCGGCGAACTTCTTGCGACGCTCGAAGGTGTGGCAATTTCAAACGGTGCAACTAGTT

GGCTTGCGCTCGCATGTGTAAACCTTCTCTTGAAGGCAAGACAAGAAATTGATGTCTTCT

TGTTTGGGGCAGGAAAAGTCGCGGAGGCTGTGATTCTTGCCCTTAATCATGGTGCGGCTG

CAAAAATCAAAACCATGGCAGTGCTCAGCCAAGCTCCAATTGAAGGTGGGGTTCCAGCTC

GATGCCGTCATTGATCGGAAGGTTATACCAAAAGCCAAACTCGTCATAACAGCAACAAAT

TCCGAGGAGCTCGGTACTCGAGGCCGACGAAATTGCGCCAAATGCAGTAACCATATCCCT

CGGAAAAGATGAATTGCCCGCCGCCTACTTCGATCGCCTTTTGAACGCAGAGGGTCTGAT

TATTGGCGACGATCTGGATGCGATCGAATCGCGCAATGTCAATTCCCTGGTGCTTTACTA

CTCGAAACGTGATTTGAAGCTGACAGAACATGGAAGGGATCATTGGATAAAGAACTACGC

CAATGTCCTTGCTGATCCAGCTCTCATGGAGGAACTTAAGACATGAGAGGGGCCAGCCAA

CTTTTCATCGGTTGGCCTTGCCAGCCTAGACTTGGCAATGGCCGGCCGACTCTACGAAAC

TCTTACTGCGAAACTCTCCCACCCCCAGTAGACAAAACCCTCTTAGGATTGCGCTCTTCA

GAAGGTGCGCCTCCCCAGTCACAACCGAAAAGTCACTCAACTTGGCTCAAGTACCCTACT

TAGTTTGTCTCTCTGTCGTGTTTAGTATCAGCAACAATAACAAATATTGTAAATAACATA

TATTTATATGGTGATTATATGGCATGAAAGCGTGACAACGAATATGTTTTCCATAGTTTG

ATAAACTCAACACCCGAAAAAGCCGGTGACCAAAGGGTTGGGACCTCTTTGCACGAACTC

CTATGGCTGCCAGACTATCTACAGAGCTTTTTCGGAAGCTGACGCAGCATCTGATCGCCA

CACAATCCTCGCGGACGTGGATGGCGTGGCTACATGCAAGCAGTTTGACTGAGAGTTTTC

GAGTGACTATTTTAAAAGGACAATATTTCGTCCCAAGCGATAAAGCTCGCTCACATGACT

AAGTCGTTGCTTTAATTCAACTGCGTGAAAAGATTTAAAGGGCAGAACTGTTTGTGTTTC

GTATCCAGTTCACCAGCAATTATTGGCTACGCATAAAGCTCATTATCGTCGATTCTTTAC

CCAAACACTGATGCCATTCCAAATGAGTTAGGCCAAAGATGTACTCATGTCGGACAGGCG

CGATTTGGACTTCGTGATCCTCAAGTCTATCGCAGTGCTGCTTTATTACAAGCTATGCTT

CCACCCTCCATTATTTGGTCAACTGGACCTAGCTTTTGACAAGAATAGGCCAGCGTTCAT

CTATCTATCTTTCAAAAACATGCAGTTGCTGCTGCGTAAAGGATGTAACATCGGATTTGG

TGTGGAGACGGTGCTCGCTACGACTATACTGCCTTACTGTAGTGATCTGTCCCGCCAAGC

GATGAGGGGAGCGGCCTGCCTCTCGTGAGCGTCAATCCATACGGTTGCATGTTCGTTTGT

TTCAAAGGCATTCCCAAGAAGGGTTTCCAGCTTGACAACTTTCTCATCAGTGGGTGTGAA

CTCGCCGGTTAGTGAGTCACCGCCCATTTTGGGAACTACCAGGGGGGTGCCAACGACCTC

CTCCGTCGACATCTCAGACCAATTTCAATTGCCCAACTGCACCGTTCTTTCTATGTTGAA

GTCATGGGCGACTATGAAATCTCCatctttggtcggtactacctcgatctcgaccatatt

gaaaagcctgagagatttgcaaattgcccctacactgttttccggcaagtgtggcccatg

GAATCCACGGTGAGCTATCAATTGGCGGTCGCTTGAAGTCTTTCGAGCGATAAGCCAATT

GGCGAGGCAGTGTTTCGCGATCGCATCTTTAAGAGGAAAGTCCGATGCAAATCTCCATGT

AGACCCAGTCGTTTCGGCCAAGGCGGCCTCTAAATCAACTTGAGTAGCAGTTGTGTTAAC

GGAATCTGTTTTGACTGCCATTCTGTAAGAAATGTCAGCAAAGGAAAAGCTAGTTAGAGA

AGGAGAGCTTGCTCAATAGTGTGTCTATTCTCAAATGAACTATACTAGCGCAATGAGTTG

GTGTTGTTCGCTGATGGGAAAAAGGAAACGCATCCCCCTCTTCTACAAAATTCTGTGGGC

CCGGAGGGACTCCGTATGAAAGTGCACAGCATGTGGACGCATCACCATGCAATTAGCAAG

CTTAGCCAATGTCGTGTCTGTCAGAGTGTGCAGCATGTGAGCCAAGCGCAATTACAATAA

AAAAATAATATAAAAACTCTGCCGAGGTCCGAACCGTCTCAAATGCTTTGAGGGCCATCC

AAGCCAAGCGCACCTAACGCCTAGCACACCATTGTTGCGCCGCTCGAGCATTAATGGTTC

GGGACCTGGTGTCATCTGGAAACCTATGAAGCATGGACACGGCAAAATCAGCGCCGTGGC

TGTGTCAGACACAAGGACACGTCGGGGACACGTACaggacacgt

>ta3981_1

ctccaaatttccctcaccttgaagtatcaatttgatctAGAAGCCAATCCAAACtCATTG

AAAGGCCTGTCATCAACCTGTCAACACGGTTCAATGTTTGCTCGAGTGGCGCAGCAATGG

TGTGCTAGGCGTTAGGTGCGCCTGGCTGGGATGGCCCTCAAAGCATTTGAGACAGTTCGG

ACCTCGGCAGAGATTTTATATTATTTTTTTGTTGTAATCGCGTTTGGCCCACATGCTGCA

CACTCCGACAGACACGGCATTGGCTAAACCCGCTAATTGCGTGGTGATGCGTCCACGTGC

TGCGCACTTTCATGCGGAGTCCCCCCGAGCCCGTAGAATTTTGTAGAAGAGGGGGACGCG

TTTCCTTTTTCCCATCAGCAAACAACACCAACTCACTATGCTAGTATAGTTCATCTGAGA

ACAGACACACTGCTGAGCAAGCTCTCCTTCTCCAACTAGCTTTTTCTTTGCTGACATTTC

CTACAAAATGGCAGCAAAACAGATTCCGTTAACACAACTGCTGCTCAGGTTGATTTAGAG

GCCGCCTTGGCCGAAGCGACCGGGTCTACATGGAGATTTGCATCAAACTTTCCTCTTAAA

GATGCGATCGCAAAACACTGCCTCGCCAATTGGCTTATCGCTCGAAAGACTTCAAGCAAC

CACCAATTGATAGTTCACCGTGAATTCCATGGGCCACACCTGCCGGAAAACAGCGTAGGG

GCAGTCTGCAAATCTCTCAGACTTTTTAATATGGTCGAGATCAACGTAGTACCGACCAAA

GATAGAGATTTCATAGTCGTCCACGACTTCAACACAGAAAGAACGACGCAGTTAGGCAAT

TGAAATTGGTCTGATATGTCGACGAAAGAGGCCGTTGGCACCCCCCTAGTAGTTCCCATA

ATGGGCGGTGACTCCCTAACCAGCGAGTTCACACCCACCGATGAGAAAGTTGTCAAGCAA

GAAACCCTTCTCAGGAATGCCTTTGAAACAAACAAACAGGCAACCATACGGATTGACGCT

GACGAGAGGCAGGCCGCTCCCCTCGTCGCTTGGCTGAGCCATAGGCCGGCCTATCACGAC

AAGACAATCGTTATCTTTCATACATATGGATACAACAATGGACACAAGTTCGCCTTGGTG

ATCGATCAAGCCGACGCTACAGAAAATTGGCGCGAAACTGTAGCGCTAATGCCAAACATT

CTGCCAAATGAACTCTCTAGGCTCGCGAAGCAATATCCACCAAATATCACGTCGCATTTT

TACGACCTCGTTGAGGCCGGCAAGAATTGGTTCGCGTCTGTCTTCTCCCAGAATATGATG

GTGGACGTCACTTTCCTCAAGATTGCGGGCACTGGCGAAGGACAGACTAACAAAGTCGTT

GATCCAAATGGAAATGAGGTTCTGCATCCTAAAGAGAAGGAGGAATATCTCCGCGATTCA

GCTTCAATTGAAATCGCGAAATGAATCTGCGCCACCCATCCCGGAATCGAGATTGGAGGC

GTCACACGAACTTATGCTTATAAGAGTCGAGGACATCGCCACAAAATCGATCTTTGGACG

GGTCAACCGAAGCGGTGGCCGACCCACGCGACGAAGTATATCTGGGAAGATTTCGCCAAG

CCTGGAAATGCGCTCAGATGGGGTCAAACGATTATCTCTGATCGCATTGGCGACGACCTT

GCAGCTTGTGTGCTTCAAAGCCGTGGAGAAAATTTTGTCAGCAGTCATGAATATCCCGCT

GTTCATGTCGATTAGATGGGCGATCTGTGTCTTAGTCTAAGCTTCCGCTTAACGCATCTT

TGCTACTTTAATGTATTTTTTTAATGATATTGTTGGAGTTTTCAGCACATCTTAATCGCA

AGTAGATATTTTTTTTGAATCGGATTCGACTTCAAGCAGATGTACTTCTTTGTCTCTCGA

TAAATCTAATAACCCTGCGAGCCGGCCTTTCGCCTTAAACTAATTTCTGAGAGTTAGGTC

GGCGCCTCTCGTGGTTTCCATGTTCTATGGAAACGGCCTCTCGGGACTTGCCTTCCCGCA

ACTCATCGAGCTTGCCTGGCCCGCTCGGGTGGTGGCGCAAGCCATCGTTAAATCGTTCGT

AATCCTCTACTCTCGCATAAATGAGAGGGGAATCCAAAGATGATCACTTTTCAGCTAATT

GGAATCTGCAATCCTAAAAGAGGCAAGCACTATGTGGGGTCCACAACTTATAGAAATTTG

GCCTAACCATTCTGCATAATGGATCATGACATGGGCCATTTATGGCCGCAAAAACTGGCA

CAAATTCCAACCTGTTCAGCACAATGAACAAGTTCTTCCAATTGGAATAAAGGAGGTTAA

AATTATTCGAGTAGACATGATGTGCCGGCACGCCACTAGCCTCGCATTGATGCCACCCTT

TCTTGCGCACACTTATATAATGCTGAGCTAATAATAGAGATAAAAATAGTCAGCTTGTAA

ACTGGACCTTTACTACTGCCCCTAATCTCAAGATCAAAGCGACATATACCTCCTCTCTTT

CAAGTCTTTTTGCAACTTGTTCCAAAGCAAGCGTTCGTGAATCTGCCAGGCAGCCTTCAC

GAAAATGGCTGATCAACTATGTGAGGGGCGTTGGTCCGAAGCCAATCTATCATTCGTTGG

GATATCCATCACCCCTGAATTCATCGATGAGGCCCTCAGAGGCTTTTGCCAAAAGAGCCG

TTGGCTACAAAGAGGTTTACGTTCGGGTTGATCGAGATCCTAAAGTTGCCTGGGAAGGAG

CGCTCAATGGTTACCTCGGCAAATATTCTGGCGTCAAAAATGTTCATTTTGCGCCGAACA

ACGCCGCGATTGGAAAGCCCCCATGCCATATCGACATTTTGCTTCGGAAAAAATTTAGCG

GCGAATTTCTTGTGACGCTCGAAGGTGTGGCAATTTCAAACGGTGCAACTGGTTGGCTTG

CGCTCGCGTGCGTAAACCTTCTCTTGAAGGCAAGACAAGATATTGATGTCTTCTTGTTTG

GGGCGGGAAAAGTCGCCGAGGCTGTGATTCTTGCCCTCAATCACGGTGCGGCTGCAAAAA

TCAAACCATGGCAGTGCTCAGCCAAGCTCCAATCGAAGGTGGGGTTCCAGCTCGATGTCG

TCATTGATCGGAAGGTTATACCAAAAGCCAAACTCGTCATAACAGCAACAAATTCCGAGG

AGCTCGCACTCGAGGCCGACGAAATTGCGCCAAATGCAGTAACCATATCCCTCGGAAAAG

ATGAATTGCTTGCCGCCTACTTCGATCGCCTTTTGAACGCAGAGGGTCTGATTATCGGCG

ACGATCTGGATGCGATCGAATCGCGCAATGTCAATTCCCTGGTGCTTTACTACTCGAAAC

GTGATTTGAAGCTGACAGAACATGGAAGGGATCATTGGATAAAAAACTACGCCAATGTCC

TTGTTGATCCAGCTCTCATGGAGGAACTTAAGACAtGGGAGGGgccagccaacttttcat

cggttggcctTGCCAGCCTAGACTTGGCGATGGCCGGCCGACTCTACGAAACTCTTACTG

CGAAACTCTCCCACCCCCAGTAGACAAGACCCTCTTAGGATTGCGCTCTTCAGAAGGTGC

GCCTCCCCAGTCACAACCGGAAAGTCACTCAACTTGGCTCAAGTACCCTACTTAGTTTGT

CTCTCTGTCGTGTTTAGTATCAACAACAATAACAAATATTGTAAACAACATATATTTATA

TGGTAATTATATGGCATGTAAGCGTGACAACGATTATGTTTTCCATAGTTTGATAAACTC

AACACCCGAAAAAGCTGGTGACCAAAGGGTTGGGACCTCTTTGCACGAACTCCTATGGCT

GCCAGACTATCTACAGAGCTTTTTCGAAAGCTGACGCAGCATCTGATCGCTACACAATCC

TCGCGGACGTGGATGGCGTGGCTACACGCAAGCAGTTTGACTGAGAGTTTTCGAGTGACT

ATTTTAAAAGGACGATATTTCGTCCCAAGCAATAAAACTCGCTCACATGACTAAGTCGTT

GCTTTAATTCAGCTGCGTGAAAAGATTTAAAGGGCAGAACTGTTTGTGtTTCGTATCCAG

TTCACCGGCAAttattggctacgcataaagctcattATCGTCGATTCTTTACCCAAACAC

TGATGTCATTCCAAATGAGTTGGGCCAAAGATGTACTCATGTCGGACAGGCGCGATTTGG

ACTTCGTGATCCTCAAGTCTATCGCAGTGCTGCTTTATTACAGGCTATGCTTCCACCCTC

CATTATTTGGTCAACTGGACCTGGCTTTTGACAAGAATAGGCCAGCGTTCATCTATCTAT

CTTTCAAAAACATGCAGTTGCTGCTGCGTAAAGGATGTAACATCGGATTTGGTGTGGAGA

CGGTGCTTGCTACGACTATACTGCCTTACTGTAGTGATCTGTCCTGCCAAGCGACGAGGG

GAGCAGCCTGCCTCTCGTGAGCGTCAATCCATACAGTTGCATGTTCGTTTGTTTCAAAGG

CATTCCCAAGAAGGGTTTCCAGCTTGACAACTTTCTCATCGGTGGGTGTGAACTAGCCGG

TTAGTGAGTCACCGCCCATTTTGGGAACTACCAGGGGGGTGCCAACGACCTCCTCCGTCG

ACATCTCAGACCAATTTCAATTGCTCAACTGCGCCGTTCTTTCTATGTTGAAGTCATGGG

CGACTATGAAATCTCCATCTTTGGTCGGTACTACCTCGATCTCGACCATATTGAAAAGCC

TGAGAGATTTGCAAATTGCCCCTACACTGTTTTCCGGCAAGTGTGGCCCATGGAATCCAC

GGTGAACTATCAATTGGCGGTCGCTTGAAGTCTTTCGAGCGATAAGCCAATTGGCGAGGC

AGTGTTTTGCGATCGCATCTTTAAGAGGAAAGTCCGATGCAAATCTCCATGTAGACCCGG

TTGCTTCGGCCAAGGCGGCCTCTAAACCAACTTGAGCAGCAGTTGTGTTAACGGAATCTA

TTTTGACTACCATTTTGTAAGAAATGTCAGCAAAGGAAAAGCTAGTTAGAGAAGGAGAGC

TTGCTCAATAGTGTGTCTATTCTCATATGAACTATACTAGCGCAATGAGTTAGTGTTGTT

CGCTGATGGGAAAAAGGAAATGCATCCCCCTCTTCTACAAAATTCTGTGGGCCCGGAGGG

ACTCCGTATGAAAGTGCACAGCATGTGGACGCATCACCATGCAATTAGCGAGCTTAGCCA

ATGCCGTGTCTGTCAGAGTGTGCAGCATGTGGGCCAAGCGCAATTACAATAAAAAAATAA

TATAAAAACTCTGCCGAGGTCTGAACCGTCTCAAATGCTTTGAGGGCCATCCAAGCCAAG

CGCACCTAACGCCTAGCACACCATTGTTGCGCCGCTCGAGCATTAATGGTCgGGGcctgg

tgtcatctggaaacctatgaagcatggacacggcaaaatcagcg

>ta3976_1

tcACCTTGAAGTATCAATTTGATCTAGAAGCCAATCCAAACTCATTGAAAGGCCTGTCAT

CAACCTGTCAACACGGTTCAATGTTTGCTCGAGTGGCGCAGCAATGGTGTGCTAGGCGTT

AGGTGCGCCTGGCTGGGATGGCCCTCAAAGCATTTGAGACAGTTCGGACCTCGGCAGAGA

TTTTATATTATTTTTTTGTTGTAATCGCGTTTGGCCCACATGCTGCACACTCCGACAGAC

ACGGCATTGGCTAAACCCGCTAATTGCGTGGTGATGCGTCCACGTGCTGCGCACTTTCAT

GCGGAGTCCCCCCGAGCCCGTAGAATTTTGTAGAAGAGGGGGACGCGTTTCCTTTTTCCC

ATCAGCAAACAACACCAACTCACTATGCTAGTATAGTTCATCTGAGAACAGACACACTGC

TGAGCAAGCTCTCCTTCTCCAACTAGCTTTTTCTTTGCTGACATTTCCTACAAAATGGCA

GCAAAACAGATTCCGTTAACACAACTGCTGCTCAGGTTGATTTAGAGGCCGCCTTGGCCG

AAGCGACCGGGTCTACATGGAGATTTGCATCAAACTTTCCTCTTAAAGATGCGATCGCAA

AACACTGCCTCGCCAATTGGCTTATCGCTCGAAAGACTTCAAGCAACCACCAATTGATAG

TTCACCGTGAATTCCATGGGCCACACCTGCCGGAAAACAGCGTAGGGGCAGTCTGCAAAT

CTCTCAGACTTTTTAATATGGTCGAGATCAACGTAGTACCGACCAAAGATAGAGATTTCA

TAGTCGTCCACGACTTCAACACAGAAAGAACGACGCAGTTAGGCAATTGAAATTGGTCTG

ATATGTCGACGAAAGAGGCCGTTGGCACCCCCCTAGTAGTTCCCATAATGGGCGGTGACT

CCCTAACCAGCGAGTTCACACCCACCGATGAGAAAGTTGTCAAGCAAGAAACCCTTCTCA

GGAATGCCTTTGAAACAAACAAACAGGCAACCATACGGATTGACGCTGACGAGAGGCAGG

CCGCTCCCCTCGTCGCTTGGCTGAGCCATAGGCCGGCCTATCACGACAAGACAATCGTTA

TCTTTCATACATATGGATACAACAATGGACACAAGTTCGCCTTGGTGATCGATCAAGCCG

ACGCTACAGAAAATTGGCGCGAAACTGTAGCGCTAATGCCAAACATTCTGCCAAATGAAC

TCTCTAGGCTCGCGAAGCAATATCCACCAAATATCACGTCGCATTTTTACGACCTCGTTG

AGGCCGGCAAGAATTGGTTCGCGTCTGTCTTCTCCCAGAATATGATGGTGGACGTCACtt

tcctcaagattgcgggcactaGCGAAGGACAGACTAACAAAGTCGTTGATCCAAATGGAA

ATGAGGTTCTGCATCCTAAAGAGAAGGAGGAATATCTCCGCGATTCAGCTTCAATTGAAA

TCGCGAAATGAATCTGCGCCACCCATCCCGGAATCGAGATTGGAGGCGTCACACGAACTT

ATGCTTATAAGAGTCGAGGACATCGCCACAAAATCGATCTTTGGACGGGTCAACCGAAGC

GGTGGCCGACCCACGCGACGAAGTATATCTGGGAAGATTTCGCCAAGCCTGGAAATGCGC

TCAGATGGGGTCAAACGATTATCTCTGATCGCATTGGCGACGACCTTGCAGCTTGTGTGC

TTCAAAGCCGTGGAGAAAATTTTGTCAGCAGTCATGAATATCCCGCTGTTCATGTCGATT

AGATGGGCGATCTGTGTCTTAGTCTAAGCTTCCGCTTAACGCATCTTTGCTACTTTAATG

TATTTTTTTAATGATATTGTTGGAGTTTTCAGCACATCTTAATCGCAAGTAGATATTTTT

TTTGAATCGGATTCGACTTCAAGCAGATGTACTTCTTTGTCTCTCGATAAATCTAATAAC

CCTGCGAGCCGGCCTTTCGCCTTAAACTAATTTCTGAGAGTTAGGTCGGCGCCTCTCGTG

GTTTCCATGTTCTATGGAAACGGCCTCTCGGGACTTGCCTTCCCGCAACTCATCGAGCTT

GCCTGGCCCGCTCGGGTGGTGGCGCAAGCCATCGTTAAATCGTTCGTAATCCTCTACTCT

CGCATAAATGAGAGGGGAATCCAAAGATGATCACTTTTCAGCTAATTGGAATCTGCAATC

CTAAAAGAGGCAAGCACTATGTGGGGTCCACAACTTATAGAAATTTGGCCTAACCATTCT

GCATAATGGATCATGACATGGGCCATTTATGGCCGCAAAAACTGGCACAAATTCCAACCT

GTTCAGCACAATGAACAAGTTCTTCCAATTGGAATAAAGGAGGTTAAAATTATTCGAGTA

GACATGATGTGCCGGCACGCCACTAGCCTCGCATTGATGCCACCCTTTCTTGCGCACACT

TATATAATGCTGAGCTAATAATAGAGATAAAAATAGTCAGCTTGTAAACTGGACCTTTAC

TACTGCCCCTAATCTCAAGATCAAAGCGACATATACCTCCTCTCTTTCAAGTCTTTTTGC

AACTTGTTCCAAAGCAAGCGTTCGTGAATCTGCCAGGCAGCCTTCACGAAAATGGCTGAT

CAACTATGTGAGGGGCGTTGGTCCGAAGCCAATCTATCATTCGTTGGGATATCCATCACC

CCTGAATTCATCGATGAGGCCCTCAGAGGCTTTTGCCAAAAGAGCCGTTGGCTACAAAGA

GGTTTACGTTCGGGTTGATCGAGATCCTAAAGTTGCCTGGGAAGGAGCGCTCAATGGTTA

CCTCGGCAAATATTCTGGCGTCAAAAATGTTCATTTTGCGCCGAACAACGCCGCGATTGG

AAAGCCCCCATGCCATATCGACATTTTGCTTCGGAAAAAATTTAGCGGCGAATTTCTTGT

GACGCTCGAAGGTGTGGCAATTTCAAACGGTGCAACTGGTTGGCTTGCGCTCGCGTGCGT

AAACCTTCTCTTGAAGGCAAGACAAGATATTGATGTCTTCTTGTTTGGGGCGGGAAAAGT

CGCCGAGGCTGTGATTCTTGCCCTCAATCACGGTGCGGCTGCAAAAATCAAACCATGGCA

GTGCTCAGCCAAGCTCCAATCGAAGGTGGGGTTCCAGCTCGATGTCGTCATTGATCGGAA

GGTTATACCAAAAGCCAAACTCGTCATAACAGCAACAAATTCCGAGGAGCTCGCACTCGA

GGCCGACGAAATTGCGCCAAATGCAGTAACCATATCCCTCGGAAAAGATGAATTGCTTGC

CGCCTACTTCGATCGCCTTTTGAACGCAGAGGGTCTGATTATCGGCGACGATCTGGATGC

GATCGAATCGCGCAATGTCAATTCCCTGGTGCTTTACTACTCGAAACGTGATTTGAAGCT

GACAGAACATGGAAGGGATCATTGGATAAAAAACTACGCCAATGTCCTTGTTGATCCAGC

TCTCATGGAGGAACTTAAGACATGGGAGGGGCCAGCCAACTTTTCATCGGTTGGCCTTGC

CAGCCTAGACTTGGCGATGGCCGGCCGACTCTACGAAACTCTTACTGCGAAACTCTCCCA

CCCCCAGTAGACAAGACCCTCTTAGGATTGCGCTCTTCAGAAGGTGCGCCTCCCCAGTCA

CAACCGGAAAGTCACTCAACTTGGCTCAAGTACCCTACTTAGTTTGTCTCTCTGTCGTGT

TTAGTATCAACAACAATAACAAATATTGTAAACAACATATATTTATATGGTAATTATATG

GCATGTAAGCGTGACAACGATTATGTTTTCCATAGTTTGATAAACTCAACACCCGAAAAA

GCTGGTGACCAAAGGGTTGGGACCTCTTTGCACGAACTCCTATGGCTGCCAGACTATCTA

CAGAGCTTTTTCGAAAGCTGACGCAGCATCTGATCGCTACACAATCCTCGCGGACGTGGA

TGGCGTGGCTACACGCAAGCAGTTTGACTGAGAGTTTTCGAGTGACTATTTTAAAAGGAC

GATATTTCGTCCCAAGCAATAAAACTCGCTCACATGACTAAGTCGTTGCTTTAATTCAGC

TGCGTGAAAAGATTTAAAGGGCAGAACTGTTTGTGTTTCGTATCCAGTTCACCGGCAATT

ATTGGCTACGCATGAAGCTCATTATCGTCGATTCTTTACCCAAACACTGATGTCATTCCA

AATGAGTTGGGCCAAAGATGTACTCATGTCGGACAGGCGCGATTTGGACTTCGTGATCCT

CAAGTCTATCGCAGTGCTGCTTTATTACAGGCTATGCTTCCACCCTCCATTATTTGGTCA

ACTGGACCTGGCTTTTGACAAGAATAGGCCAGCGTTCATCTATCTATCTTTCAAAAACAT

GCAGTTGCTGCTGCGTAAAGGATGTAACATCGGATTTGGTGTGGAGACGGTGCTTGCTAC

GACTATACTGCCTTACTGTAGTGATCTGTCCTGCCAAGCGACGAGGGGAGCAGCCTGCCT

CTCGTGAGCGTCAATCCATACAGTTGCATGTTCGTTTGTTTCAAAGGCATTCCCAAGAAG

GGTTTCCAGCTTGACAACTTTCTCATCGGTGGGTGTGAACTAGCCGGTTAGTGAGTCACC

GCCCATTTTGGGAACTACCAGGGGGGTGCCAACGACCTCCTCCGTCGACATCTCAGACCA

ATTTCAATTGCTCAACTGCGCCGTTCTTTCTATGTTGAAGTCATGGGCGACTATGAAATC

TCCATCTTTGGTCGGTACTACCTCGATCTCGACCATATTGAAAAGCCTGAGAGATTTGCA

AATTGCCCCTACACTGTTTTCCGGCAAGTGTGGCCCATGGAATCCACGGTGAACTATCAA

TTGGCGGTCGCTTGAAGTCTTTCGAGCGATAAGCCAATTGGCGAGGCAGTGTTTTGCGAT

CGCATCTTTAAGAGGAAAGTCCGATGCAAATCTCCATGTAGACCCGGTTGCTTCGGCCAA

GGCGGCCTCTAAACCAACTTGAGCAGCAGTTGTGTTAACGGAATCTATTTTGACTACCAT

TTTGTAAGAAATGTCAGCAAAGGAAAAGCTAGTTAGAGAAGGAGAGCTTGCHCAATAGTG

TGTCTATTCTCATATGAACTATACTAGCGCAATGAGTTAGTGTTGTTCGCTGATGGGAAA

AAGGAAATGCATCCCCCTCTTCTACAAAATTCTGTGGGCCCGGAGGGACTCCGTATGAAA

GTGCACAGCATGTGGACGCATCACCATGCAATTAGCGAGCTTAGCCAATGCCGTGTCTGT

CAGAGTGTGCAGCATGTGGGCCAAGCGCAATTACAATAAAAAAATAATATAAAAACTCTG

CCGAGGTCTGAACCGTCTCAAATGCTTTGAGGGCCATCCAAGCCAAGCGCACCTAACGCC

TAGCACACCATTGTTGCGCCGCTCGAGCATTAATGGTCCGGGACCTGGTGTCATCTGGAA

ACCTATGAAGCATGGACACGGCAAAATCAGCGCCGTGGCt

>ta3976_2

accttgaagtatcaatttgatctagaagccaATCCAAACTCATTGAAAGGCCTGTCATCA

ACCGGTCAACACGGTTCAATGTTTGCTCGCGTGGCGCAGCAATGGTGTGCTAGGCATTAG

GTGCGCCTGGCTTGGATGGCCCTCAAAGCATTTGAGGCAGTTCGGACCTCGGCAGAGATT

TTATATTATTTTTTTGTTGTAATCGCGCTTGGCCCACATGCTGCACACTCCGATAGACAC

GGCATTGGCTAAACCCGCTAATTGCGTGGTGATGCGTCCACGTGCTGCGCACTTTCATGC

GGAGTCCCCCCGAGTCCATAGAATAAGAGGGGGACGCGTTTCCTTTTTCCCATCAGCAAA

CAACACCAACTCACTGTGCTAGTATAGTTCATCTGAGAACAGACACACTGCTGAGCAAGC

TCTCCTTCTCCAACTAGCTTTTTCTTTGCTGACATTTCCTACAGAATGGCAGCAAAACAG

ATTCCGTTAACACAACTACTGCTCAGGTTGATTTAGAGGCCGCCTTGGCCGAAGCGATCG

GGTCTACATGGAGATTTGCATCAGACTTTCCTCTTAAAAATGCAATCGCGAAACACTGCC

TCGCCAATTGGCTTATCGCTCGAAAGACTTCAAGCAACCACCAATTGATAGTTCACCGTG

AATTCCATGGGCCACACCTGCCCGAAAACAGCGTAGGGGCagtctgcaaatctctcagac

tttttaatatggtcgagatCAACGTAGTACCGACCAAAGATAAAGATTTCATAGTCGTCC

ACGACTTCAACACAGAAAGAACGACGCAGTTAGGCAATTGAAATTGGTTTGATATGTCGA

CGAAAGAGGCTGTTGGCACCCCCCTGGTAGTTCCCATAATGGGCGGTGACTCCCTAACCA

GCGAGTTCACACCCACCGATGAGAAAGTTGTCAAGCAAGAAACCCTTCTCAAGAATGCCT

TTGAAACAAACAAACAGGCAACCATACGGATTGACGCTCACGAGAGGCAGGCCGCTCCCC

TCGTCGCTTGGCTGAGCCATAGGCCGGCCTATCACGACAAGACAATCGTTATCTTTCATA

CATATGGATACAACAATGGACACGAGTTCGCCTTGGCGGTCAATCAAGCCGGCGCTACAA

AAAATTGGCGCGAAACTGTAGCGCTAATGCCAAACATTCTGCCGAATGAACTCTCTAGGC

TCGCGAAGCAATATCCACCAAATTGCACGTCGCATTTTTACGACCTCGTTGAGGCCAGCA

AGAATTGGTTCGCATCTGTCTTCTCCCAGAATATGATGGTGGTCGTCACTTTCCTCAAGA

TTGCGGGCACTAGCGAAGGACAGACTAACAAAGTCGTTGATCCAAATGGAAATGAGGTTC

TGCATCCTAAAGAGAAGGAGGAATATCTCCGCGATTCCGCTTCAATCGAAATCGCGAAAT

GAATCTGCGCCACCCATCCCGGAATCGAGATTGGAGGCGTCACACGAACTTATGCTTATA

AGAGTCGAGGACATCGCTACAAAATCAATCTTTGGACGGGTCAACCGAAGCGGTGGCCGA

CCCACGCGACGAAGTACATCTGGGAAGATTTCGCCAAGCCTGGAAATGCGCTCAGATGGG

GTCAAACGATTATCTCTGATCGCATTGGTGACGACCTTGCAGCTTGTGTGCTTCAAAGCC

GTGGAGAAAATTTTGTCAGCAGTCATGAATATCCTGCTGTTCATGTCGATTAGATGGGCG

ATCTGTGTCTTAGTCTAAGCTTCTGCTTAACGCATCTTTGCTACTTTAATGTATTTTTTT

AATGATATTGTTGGAGTTTTGAGCACATCTTAATCGCAAGTAGATAttttttttgaatcg

gattcgacttcaagcagatgtacttctttgtctctcgataAaTCTAATGACCCTGCGAGC

CGGCCTCTCACCTTAAACTAATTTCTGAGAGTTAGGTCGGCGCCTCTCGTGGTTTCCATG

TTTTATGGAAACGGCCGCTCGGGACTTGCCTTCCCGCAACTCATCGAGCTTGCCTGGCCC

GCTCGGGTGGTGGCACAAGCCATCGTTAAATCGTTCGTAATCCTCTACTCTCGCATAAAT

GAGAGGGGAATCCAAAGATGATCACTTTTCAGCTAATTGGAATCTGCAATCCCAAAAGAG

GCAAGCACTATGTGGGGTCCACAACTTATAGAATTTTGGCCTAACCATTCTGCATAATGG

ATCATGACATGGGCCATTTATGGCCGCAAAAACTGGCACAAATTCCAACCTATTCAGCAC

AATGAACAAGTTCTTCCAATTGGAATAAAGGAGGTTAAAATTATTCGAGTAGACATGACG

TGCTGGCACGCCACTAGCCTCGCATTGATGCCACTCTTTCTTGCGCACACTTATATAATG

CTGAGCTAATAATAGAGATAAAAATAGTCCGCTTGTAAACTGGACCTTTATTACTGCCCC

TAATCTCAAGATCAAAGCGACATATACCTCTTCTCTTTCAAGTCTTTTTGCAACTTGTTC

CAAAGCAAGCGTTTGTGAATCTGCCAGGCAGCCTTCACGAAAATGGCTGATCAACTGTGT

GAGGGGCGTTGGTCCGAAGCCAATCTATCATTCGTTGGGATATCTATCACCCCTGAATTC

ATCGATGAGGCCCTCAGAGGCTTTTGGGAGTCTTTTGCCAAAAGAGCCGTTGGCTACAAA

GAGGTTTACGTTCAGGTTGATCGAGATCCTAAAGTTGCCTGGGAAGGAGCGCTCAATGGT

TACCTCGGCAAATATTCTGGCGTAAAAAATTTTCATTTTGCGCCGAACAACGCCACGATT

GGAAAGCCcccatgccatatcgACATTTTgcTtCGGAAAAAATTTAGCGGCGAACTTCTT

GCGACGCTCGAAGGTGTGGCAATTTCAAACGGTGCAACTAGTTGGCTTGCGCTCGCATGT

GTAAACCTTCTCTTGAAGGCAAGACAAGAAATTGATGTCTTCTTGTTTGGGGCAGGAAAA

GTCGCGGAGGCTGTGATTCTTGCCCTTAATCATGGTGCGGCTGCAAAAATCAAAACCATG

GCAGTGCTCAGCCAAGCTCCAATTGAAGGTGGGGTTCCAGCTCGATGCCGTCATTGATCG

GAAGGTTATACCAAAAGCCAAACTCGTCATAACAGCAACAAATTCCGAGGAGCTCGGTAC

TCGAGGCCGACGAAATTGCGCCAAATGCAGTAACCATATCCCTCGGAAAAGATGAATTGC

CCGCCGCCTACTTCGATCGCCTTTTGAACGCAGAGGGTCTGATTATTGGCGACGATCTGG

ATGCGATCGAATCGCGCAATGTCAATTCCCTGGTGCTTTACTACTCGAAACGTGATTTGA

AGCTGACAGAACATGGAAGGGATCATTGGATAAAGAACTACGCCAATGTCCTTGCTGATC

CAGCTCTCATGGAGGAACTTAAGACATGAGAGGGGCCAGCCAACTTTTCATCGGTTGGCC

TTGCCAGCCTAGACTTGGCAATGGCCGGCCGACTCTACGAAACTCTTACTGCGAAACTCT

CCCACCCCCAGTAGACAAAACCCTCTTAGGATTGCGCTCTTCAGAAGGTGCGCCTCCCCA

GTCACAACCGAAAAGTCACTCAACTTGGCTCAAGTACCCTACTTAGTTTGTCTCTCTGTC

GTGTTTAGTATCAGCAACAATAACAAATATTGTAAATAACATATATTTATATGGTGATTA

TATGGCATGAAAGCGTGACAACGAATATGTTTTCCATAGTTTGATAAACTCAACACCCGA

AAAAGCCGGTGACCAAAGGGTTGGGACCTCTTTGCACGAACTCCTATGGCTGCCAGACTA

TCTACAGAGCTTTTTCGGAAGCTGACGCAGCATCTGATCGCCACACAATCCTCGCGGACG

TGGATGGCGTGGCTACATGCAAGCAGTTTGACTGAGAGTTTTCGAGTGACTATTTTAAAA

GGACAATATTTCGTCCCAAGCGATAAAGCTCGCTCACATGACTAAGTCGTTGCTTTAATT

CAACTGCGTGAAAAGATTTAAAGGGCAGAACTGTTTGTGTTTCGTATCCAGTTCACCAGC

AATTATTGGCTACGCATAAAGCTCATTATCGTCGATTCTTTACCCAAACACTGATGCCAT

TCCAAATGAGTTAGGCCAAAGATGTACTCATGTCGGACAGGCGCGATTTGGACTTCGTGA

TCCTCAAGTCTATCGCAGTGCTGCTTTATTACAAGCTATGCTTCCACCCTCCATTATTTG

GTCAACTGGACCTAGCTTTTGACAAGAATAGGCCAGCGTTCATCTATCTATCTTTCAAAA

ACATGCAGTTGCTGCTGCGTAAAGGATGTAACATCGGATTTGGTGTGGAGACGGTGCTCG

CTACGACTATACTGCCTTACTGTAGTGATCTGTCCCGCCAAGCGATGAGGGGAGCGGCCT

GCCTCTCGTGAGCGTCAATCCATACGGTTGCATGTTCGTTTGTTTCAAAGGCATTCCCAA

GAAGGGTTTCCAGCTTGACAACTTTCTCATCAGTGGGTGTGAACTCGCCGGTTAGTGAGT

CACCGCCCATTTTGGGAACTACCAGGGGGGTGCCAACGACCTCCTCCGTCGACATCTCAG

ACCAATTTCAATTGCCCAACTGCACCGTTCTTTCTATGTTGAAGTCATGGGCGACTATGA

AATCTCCATCTTTGGTCGGTACTACCTCGATCTCGACCATATTGAAAAGCCTGAAAGATT

TGCAAACTGCCCCTACACTGTTTTCTGGCAAGTGTGGCCCATGGAATCCACGGTGAGCTA

TCAATTGGCGGTCGCTTGAAGTCTTTCGAGCGATAAGCCAATTGGCGAGGCAGTGTTTCG

CGATCGCATCTTTAAGAGGAAAGTCCGATGCAAATCTCCATGTAGACCCAGTCGTTTCGG

CCAAGGCGGCCTCTAAATCAACTTGAGTAGCAGTTGTGTTAACGGAATCTGTTTTGACTG

CCATTCTGTAAGAAATGTCAGCAAAGGAAAAGCTAGTTAGAGAAGGAGAGCTTGCTCAAT

AGTGTGTCTATTCTCAAATGAACTATACTAGCGCAATGAGTTGGTGTTGTTCGCTGATGG

GAAAAAGGAAACGCATCCCCCTCTTCTACAAAATTCTGTGGGCCCGGAGGGACTCCGTAT

GAAAGTGCACAGCATGTGGACGCATCACCATGCAATTAGCAAGCTTAGCCAATGTCGTGT

CTGTCAGAGTGTGCAGCATGTGAGCCAAGCGCAATTACAATAAAAAAATAATATAAAAAC

TCTGCCGAGGTCCGAACCGTCTCAAATGCTTTGAGGGCCATCCAAGCCAAGCGCACCTAA

CGCCTAGCACACCATTGTTGCGCCGCTCGAGCATTAATGGTTCGGGACCTGGTGTCATCT

GGAAACCTATGAAGCATGGACACGGCAAAATCAGCGCCGTGGCTGTGTCAGACACAAGGA

CACGTCGGGGrCacgtacaggacac

>ta3975_1

gacctcggcagagattttatattatttttttgttGTAATCGCGTTTGGCCCACATGCTGC

ACACTCCGACAGACACGGCATTGGCTAAACCCGCTAATTGCGTGGTGATGCGTCCACGTG

CTGCGCACTTTCATGCGGAGTCCCCCCGAGCCCGTAGAATTTTGTAGAAGAGGGGGACGC

GTTTCCTTTTTCCCATCAGCAAACAAcaccaactcactgtgctagtataGTTCATCTGAG

AACAGACACACTGCTGAGCAAGCTCTCCTTCTCCAACTAGCTTTTTCTTTGCTGACATTT

CCTACAAAATGGCAGCAAAACAGATTCCGTTAACACAACTGCTGCTCAGGTTGATTTAGA

GGCCGCCTTGGCCGAAGCGACCGGGTCTACATGGAGATTTGCATCAAGCTTTCCTCTTAA

AGATGCGATCGCAAAACACTGCCTCGCCAATTGGCTTATCGCTCGAAAGACTTCAAGCAA

CCACCAATTGATAGTTCACCGTGAATTCCATGGGCCACACCTGCCGGAAAACAGCGTAGG

GGCAGTCTGCAAATCTCTCAGACTTTTTAATATGgtcgagatcaacgtagtaccgaccaa

agataaagatttcatagtcgtccacgacttcaacacagaaagaacgacgcagttaggcaa

ttgaaattggtttgatatgtcgacgaaagaggctgttggcacccccctggtagttcccat

aatggGCGGTGACTCCCTAACCAGCGAGTTCACACCCACCGATGAGAAAGTTGTCAAGCA

AGAAACCCTTCTCAGGAATGCCTTTGAAACAAACAAACAGGCAACCATACGGATTGACGC

TCACGAGAGGCAGGCCGCTCCCCTCGTCGCTTGGCTGAGCCATAGGCCGGCCTATCacga

caagACAATCGTTATCTTTCATACATATGGATACAACAATGGACACAAGTTCGCCTTGGC

GGTCGATCAAGCCGATGCTACAAAAAATTGGCGCGAAACTGTAGCGCTAATGCCAAACAT

TCTGCCAAATGAACTCTCTAGGCTCGCGAAGCAATATCCACCAAATATCACGTCGCATTT

TTACGACCTCGTTGAGGCCGGCAAGAATTGGTTCGCGTCTGTCTTCTCCCAGAATATGAT

AGTGGACGTCACTTTCCTCAAGATTGCGGGCACTGGCGAAGGACAGACTAACAAAGTCGT

TGATCCAAATGGAAATGAGGTTCTGCATCCTAAAGAGAAGGAGGAATATCTCCGCGATTC

AGCTTCAATTGAAATCGCGAAATGAATCTGCGCCACCCATCCCGGAATCGAGATTGGAGG

CGTCACACGAACTTATGCTTATAAGAGTCGAGGACATCGCCACAAAATCGATCTTTGGAC

GGGTCAACCGAAGCGGTGGCCGACACACGCGACGAAGTATATCTGGGAAGATTTCGCCAA

GCCTGGAAATGCGCTCAGATGGGGTCAAACGATTATCTCTGATCGCATTGGCGACGACCT

TGCAGCTTGTGTGCTTCAAAGCCGTGGAGAAAATTTTGTCAGCAGTCATGAATATCCCGC

TGTTCATGTCGATTAGATGGGCGATCTGTGTCTTAGTCTAAGCTTCCGCTTAACGCATCT

TTGCTACTTTAATGTATTTTTTTAATGATATTGTTGGAGTTTTCAGCACATCTTAATCGC

AAGTAGATATTTTTTTTGAATCGGATTCGACTTCAAGCAGATGTACTTCTTTGTCTCTCG

ATAAATCTAATGACCCTGCGAGCCGGCCTTTCGCCTTAAACTAATTTCTGAGAGTTAGGT

CGGCGCCTCTCGTGGTTTCCATGTTCTATGGAAACGGCCTCTCGGGACTTGCCTTCCCGC

AACTCATCGAGCTTGCCTGGCCCGCTCGGGTGGTGGCGCAAGCCATCGTTAAATCGTTCG

TAATCCTCTACTCTCGCATAAATGAGAGGGGAATCCAAAGATGATCACTTTTCAGCTAAT

TGGAATCTGCAATCCTAAAAGAGGCAAGCACTATGTGGGGTCCACAACTTATAGAATTTT

GGCCTAACCATTCTGCATAATGGATCATGACATGGGCCATTTATGGCCGCAAAAACTGGC

ACAAATTCCAACCTGTTCAGCACAATGAACAAGTTCTTCCAATTGGAATAAAGGAGGTTA

AAATTATTCGAGTAGACATGATGTGCCGGCACGCCACTAGCCTCGCATTGATGCCACCCT

TTCTTGCGCACACTTATATAATGCTGAGCTAATAATAGAGATAAAAATAGTCAGCTTGTA

AACTGGACCATTACTACTGCCCCTAATCTCAAAGATCAAAGCGACATATACCTCCTCTCT

TTCAAGTCTTTTTGCAACTTGTTCCAAAGCAAGCGTTCGTGAATCTGCCAGGCAGCCTTA

ACGAAAATGGCTGATCAACTATGTGAGGGGCGTTGGTCCGAAGCCAATCTATCATTCGTT

GGGATATCCATCACCCTGAATTCATCGATGAGGCCCTCAGAGGCTTTTGCCAAAAGAGCC

GTTGGCTACAAAGAGGTTTACGTTCGGGTTGATCGAGATCCTAAAGTTGCCTGGGAAGGA

GCGCTCAATGGTTACCTCGGCAAATATTCTGGCGTCAAAAATGTTCATTTTGCGCCGAAC

AACGCCGCGATTGGAAAGCCCCCATGCCATATCGACATTTTGCTTCGGAAAAAATTTAGC

GGCGAATTTCTTGTGACGCTCGAAGGTGTGGCAATTTCAAACGGTGCAACTGGTTGGCTT

GCGCTCGCGTGCGTAAACCTTCTCTTGAAGGCAAGACAAGATATTGATGTCTTCTTGTTT

GGGGCGGGAAAAGTCGCCGAGGCTGTGATTCTTGCCCTCAATCACGGTGCGGCTGCAAAA

ATCAAAACCATGGCAGTGCTCAGCCAAGCTCCAATCGAAGGTGGGGTTCCAGCTCGATGT

CGTCATTGATCGGAAGGTTATACCAAAAGCCAAACTCGTCATAACAGCAACAAATTCCCA

GGAGCTCGCACTCGAGGCCGACGAAATtgcgccaaatGCAGTAACCATATCCCTCGGAAA

AGATGAATTGCTCGCCGCCTACTTCGATCGCCTTTTGAACGCAGAGGGTCTGATTATCGG

CGACGATCTGGATGCGATCGAATCGCGCAATGTCAATTCCCTGGTGCTTTACTACTCGAA

ACGTGATttgaaactgacagaacatggaagggatCATTGGATAAAAAACTACGCCAATGT

CCTTGTTGATCCAGCTCTCATGGAGGAACTTAAGACATGGGAGGGGCCAGCCAACTTTTC

ATCGGTTGGCCTTGCCAGCCTAGACTTGGCGATGGCCGGCCGACTCTACGAAACTCTTAC

TGCGAAACTCTCCCACCCCCAATAGACAAGACCCTCTTAGGATTGCGCTCTTCAGAAGGT

GCGCCTCCCCAGTCACAACCGGAAAGTCACTCAACTTGGCTCAAGTACCCTACTTAGTTT

GTCTCTCTGTCGTGTTTAGTATCAACAACAATAACAAATATTGTAAACAACATATATTTA

TATGGTAATTATATGGCATGTAAGCGTGACAACGATTATGTTTTCCATAGTTTGATAAAC

TCAACACCCGAAAAAGCCGGTGACCAAAGGGTTGGGACCTCTTTGCACGAACTCCTATGG

CTGCCAGACTATCTACAGAGCTTTTTCGAAAGCTGACGCAGCATCTGATCGCTACACAAT

CCTCGCGGACGTGGATGGCGTGGCTACACGCAAGCAGTTTGACTGAGAGTTTTCGAGTGA

CTATTTTAAAAGGACGATATTTCGTCCCAAGCAATAAAACTCGCTCACATGACTAAGTCG

TTGCTTTAATTCAGCTGCGTGAAAAGATTTAAAGGGCAGAACTGTTTGTGTTTCGTATCC

AGTTCACCGGCAATTATTGGCTACGCATGAAGCTCATTATCGTCGATTCTTTACCCAAAC

ACTGATGTCATTCCAAATGAGTTGGGCCAAAGATGTACTCATGTCGGACAGGCGCGATTT

GGACTTCGTGATCCTCAAGTCTATCGCAGTGCTGCTTTATTACAGGCTATGCTTCCACCC

TCCATTATTTGGTCAACTGGACCTGGCTTTTGACAAGAATAGGCCAGCGTTCATCTATCT

ATCTTTCAAAAACATGCAGTTGCTGCTGCGTAAAGGATGTAACATCGGATTTGGTGTGGA

GACGGTGCTTGCTACGACTATACTGCCTTACTGTAGTGATCTGTCCTGCCAAGCGACGAG

GGGAGCAGCCTGCCTCTCGTGAGCGTCAATCCATACAGTTGCATGTTCGTTTGTTTCAAA

GGCATTCCCAAGAAGGGTTTCCAGCTTGACAACTTTCTCATCGGTGGGTGTGAACTAGCC

GGTTAGTGAGTCACCGCCCATTTTGGGAACTACCAGGGGGGTGCCAACGACCTCCTCCGT

CGACATCTCAGACCAATTTCAATTGCTCAACTGCGCCGTTCTTTCTATGTTGAAGTCATG

GGCGACTATGAAATCTCCATCTTTGGTCGGTACTACCTCGATCTCGACCATATTGAAAAG

CCTGAGAGATTTGCAAATTGCCCCTACACTGTTTTCCGGCAAGTGTGGCCCATGGAATCC

ACGGTGAACTATCAATTGGCGGTCGCTTGAAGTCTTTCGAGCGATAAGCCAATTGGCGAG

GCAGTGTTTCGCGATCGCATCTTTAAGAGGAAAGTCCGATGCAAATCTCCATGTAGACCC

GGTTGCTTCGGCCAAGGCGGCCTCTAAACCAACTTGAGCAGCAGTTGTGTTAACGGAATC

TATTTTGACTACCATTTTGTAAGAAATGTCAGCAAAGGAAAAGCTAGTTAGAGAAGGAGA

GCTTGCTCAATAGTGTGTCTATTCTCATATGAACTATACTAGCGCAATGAGTTAGTGTTG

TTCGCTGATGGGAAAAAGGAAATGCATCCCCCTCTTCTACAAAATTCTGTGGGCCCGGAG

GGACTCCGTATGAAAGTGCACAGCATGTGGACGCATCACCATGCAATTAGCGAGCTTAGC

CAATGCCGTGTCTGTCAGAGTGTGCAGCATGTGGGCCAAGCGCAATTACAATAAAAAAAT

AATATAAAAACTCTgctgaggtccgaaccgtctcaaatgctttgagggccAtccaagcca

agcgcacctaacgcctagcacaccattgttgcgccgt

>ta3975_2

ctcgcgtggcgcagcaatggtgtgctaggcgttaggtgcgcctggcttggatgGCCCTCA

AAGCATTTGAGGCAGTTCGGACCTCGGCAGAGATTTTATATTATTTTTTTGTTGTAATCG

CGCTTGGCCCACATGCTGCACACTCCGATAGACACGGCATTGGCTAAACCCGCTAATTGC

GTGGTGATGCGTCCACGTGCTGCGCACTTTCATGCGGAGTCCCCCCGAGTCCATAGAATA

AGAGGGGGACGCGTTTCCTTTTTTCCATCAGCAAACAACACCAACTCACTGTGCTAGTAT

AGTTCATCTGAGAACAGACACACTGCTGAGCAAGCTCTCCTTCTCCAACTAGCTTTTTCT

TTGCTGACATTTCCTACAGAATGGCAGCAAAACAGATTCCGTTAACACAACTACTGCTCA

GGTTGATTTAGAGGCCGCCTTGGCCGAAGCGATCGGGTCTACATGGAGATTTGCATCAGA

CTTTCCTCTTAAAAATGCAATCGCGAAACACTACCTTGCCAATTGGCTTATCGCTCGAAA

GACTTCAAGCAACCACCAATTGATAGTTCACCGTGAATTCCATGGGCCACACCTGCCGGA

AAACAGCGTAGGGGCAGTCTGCAAATCTCTCAGACTTTTTAATATGGTCGAGATCAACGT

AGTACCGACCAAAGATAAAGATTTCATAGTCGTCCACGACTTCAACACAGAAAGAACGAC

GCAGTTAGGCAATTGAAATTGGTTTGATATGTCGACGAAAGAGGCTGTTGGCACCCCCCT

GGTAGTTCCCATAATGGGCGGTGACTCCCTAAAGAGCGAGTTCACACCCACCGATGAGAA

AGTTGTCAAGCAAGAAACCCTTCTCAAGAATGCCTTTGAAACAAACAAACAGGCAACCAT

ACGGATTGACGCTCACGAGAGGCAGGCCGCTCCCCTCGTCGCTTGGCTGAGCCATAGGCC

GGCCTATCACGACAAGACAAHCGTTATCTTTCATACATATGGATACAACAATGGACACGA

GTTCGCCTTGGCGGTCGATCAAGCCGGCGCTACAAAAAATTGGCGCGAAACTGTAGCGCT

AATGCCAAACATTCTGCCGAATGAACTCTCTAGGCTCGCGAAGCAATATCCACCAAATTG

CACGTCGCATTTTTACGACCTCGTTGAGGCCAGCAAGAATTGGTTCGCATCTGTCTTCTC

CCAGAATATGATGGTGGTCGTCACTTTCCTCAAGATTGCGGGCACTGGCAAAGGACAGAC

TAACAAAGTCGTTGATCCAAATGGAAATGAGGTTCTGCATCCTAAAGAGAAGGAGGAATA

TCTCCGCGATTCAGCTTCAATCGAAATCGCGAAATGAATCTGCGCCACCCATCCCGGAAT

CGAGATTGGAGGCGTCACACGAACTTATGCTTATAAGAGTCGAGGACATCGCTACAAAAT

CAATCTTTGGACGGGTCAACCGAAGCGGTGGCCGACCCACGCGACGAAGTACATCTGGGA

AGATTTCGCCAAGCCTGGAAATGCGCTCAGATGGGGTCAAACGATTATCTCTGATCGCAT

TGGTGACGACCTTGCAGCTTGTGTGCTTCAAAGCCGTGGAGAAAATTTTGTCAGCAGTCA

TGAATATCCTGCTGTTCATGTCGATTAGATGGGCGATCTGTGTCTTAGTCTAAGCTTCCG

CTTAACGCATCTTTGCTACTTTAATGTATTTTTTTAATGATATTGTTGGAGTTTTGAGCA

CATCTTAATCGCAAGTAGATATTTTTTTTGAATCGGATTCGACTTCAAGCagatGTACTT

CTTTGTCTCTCGATAAATCTAATGACCCTGCgagccggCCTCTCGCCTTAAACTAATTTC

TGAGAGTTAGGTCGGCGCCTCTCGTGGTTTCCATGTTCTATGGAAACGGCCGCTCGGGAC

TTGCCTTCCCGCAACTCATCGAGCTTGCCTGGCCCGCTCAGGTGGTGGCACAAGCCATCG

TTAAATCGTTCGTAATCCTCTACTCTCGCATAAATGAGAAGGGAATCCAAAGATGATCAC

TTTTCAGCTAATTGGAATCTGCAATCCCAAAAGAGGCAAGCACTATGTGGGGTCCACAAC

TTATAGAATTTTGGCCTAACCATTCTGCATAATGGATCATGACATAGGCCATTTATGGCC

GCAAAAACTGGCACAAATTCCAACCTATTCAGCACAATGAACAAGTTCTTCCAATTGGAA

TAAAGGAGGTTAAAATTATTCAAGTAGACATGACGTGCTGGCACGCCACTAGCCTCGCAT

TGATGCCACTCTTTCTTGCGCACACTTATATAATGCTGAGCTAATAATAGAGATAAAAAT

AGTCAGCTTGTAAACTGGACCTTTATTACTGCCCCTAATCTCAAGATCAAAGCAACATAT

ACCTCTTCTCTTTCAAGTCTTTTTGCAACTTGTTCTAAAGCAAGCGTTTGTGAATCTGCC

AAGCAGCCTTCACGAAAATGGCTGATCAACGGTGTGAGGGGCGTTGGTCCGAAGCCAATC

TACCATTCGTTGGGATATCCATCACCCCTGAATTCATCGATGAGGCCCTCAGAGGCTTTT

GGGAGTCTTTTGCCAAAAGAGCCGTTGGCTACAAAGAGGTTTACGTTCAGGTTGATCGAG

ATCCTAAAGTTGCCTGGGAAGGAGCGCTCAATGGTTACCTCGGCAAATATTCTGGCGTAA

AAAATGTTCATTTTGCGCCGAACAACGCCACGATTGGAAAGCCCCCACGCCATATTGACA

TTTTGCTTCGGAAAAAATTTAGCGGCGAACTTCTTGCGATGCTCGAAGGTGTGGCAATTT

CAAACGGTGCAACTGGTTGGCTTGCGCTCGCATGTGTAAACCTTCTCTTGAAGGCAAGAC

AAGATATTGATGTCTTCTTGTTTGGGGCAGGAAAAGTCGCCGAGGCTGTGATTCTTGCCC

TCAATCATGGTGCGGCTGCAAAAATCAAAACCATGGCAGTGCTCAGCCAAGCTCCAATTG

AAGGTGGGGTTCCAGCTCGATGCCGTCATTGATCGGAAGGTTATACCAAAAGCCAAACTC

GTCATAACAGCAACAAATTCCGAGGAGCTCGGTACTCGAGGCCGACGAAATTGCGCCAAA

TGCAGTAACCACATCCCTCGGAAAAGATGAATTGCCCGCCGCCTACTTCGATCGCCTTTT

GAACGCAGAGGGTCTGATTATTGGCGACGATCTGGATGTGATCGAATCGCGCAATGTCAA

TTCCCTGGTGCTTTACTACTCGAAACGTGATTTGAAACTGACAGAACATGGAAGGGATCA

TTGGATAAAGAACTACGCCAATGTCCTTGCTGATCCAGCTCTCATGGAGGAACTTAAGAC

ATGAGAGGGGCCAGCCAACTTTTCATCGGTTGGCCTTGCCAGCCTAGACTTGGCGATGGC

CGGCCGACTCTACGAAACTCTTACTGCGAAACTCTCCCACCCCCAGTAGACAAAACCCTC

TTAGGATTGCGCTCTTCAGAAGGTGCGCCTCCCCAGTCACAACCGGAAAGTCACTCAACT

TGGCTCAAGTACCCTACTTAGTTTGTCTCTCTGTCGTGTTTAGTATCAGCAACAATAACA

AATATTGTAAATAACATATATTTATATGGTAATTATATGGCATGTAAGCGTGACAACGAT

TATGTTTTCCATAGTTTGATAAACTCAACACCCGAAAAAGCCGGTGACCAAAGGGTTGGG

ACCTCTTTGCACGAACTCCTATGGCTGCCAGACTATCTACAGAGCTTTTTCGGAAGCTGA

CGCAACATCTGATCGCCACACAATCCTCGCGGACGTGGATGGCGTGGCTACATGCAAGCA

GTTTGACTGAGAGTTTTCGAGTGACTATTTTAAAAGGACAATATTTCGTCCCAAGCGATA

AAACTCGCTCACATGACTAAGTCGTTGCTTTAATTCAACTGCGTGAAAAGATTTAAAGGG

CAGAACTGTTTGTGTTTCGTATCCAGTTCACCGGCAATTATTGGCTACGCATGAAGCTCA

TTATCGTCGATTCTTTACCCAAACACTGATGCCATTCCAAATGAGTTGGGCCAAAGATGT

ACTCATGTCGGACAGGCACGATTTGGACTTCGTGATCCTCAAGTCTATCGCAGTGCTGCT

TTATTACAGGCTATGCTTCCACCCTCCATTATTTGGTCAACTGGACCTAGCTTTTGACAA

GAATAGGCCAGCGTTCATCTATCTATCTTTCAAAAACATGCAGTTGCTGCTGCGTAAAGG

ATGTAACATCGGATTTGGTGTGGAGACGGTGCTCGCTACGACTATACTGCCTTACTGTAG

TGATCTGTCCCGCCAAGCGATGAGGGGAGCGGCCTGCCTCTCGTGAGCGTCAATCCATAC

GGTTGCATGTTCGTTTGTTTCAAAGGCATTCCCAAGAAGGGTTTCcagcTTGACAACTTT

CTCATCGGTGGGTGTGAACTCGCCGGTTAGTGAGTCACCGCCCATTTTGGGAACTACCAG

GGGGGTGCCAACGACCTCCTCCGTCGACATCTCAGACCAATTTCAATTGCCCAACTGCAC

CGTTCTTTCTATGTTGAAGTCATGGGCGACTATGAAATCTCCATCTTTGGTCGGTACTAC

CCCGATCTCGACCATATTGAAAAGCCTGAAAGATTTGCAAACTGCCCCTACACTGTTTTC

CGGCAAGTGTGGCCCATGGAATCCACGGTGAACTATCAATTGGCGGTCGCTTGAAGTCTT

TCGAGCGATAAGCCAATTGGCGAGGCAGTGTTTCGCGATCGCATCTTTAAGAGGAAAGTC

CGATGCAAATCTCCATGTAAACCCAGTCGTTTCGGCCAAGGCGGCCTCTAAATCAACTTG

AGCAGCAGTTGTGTTAACGGAATCTGTTTTGACTGCCATTCTGTAAGAAATGTCAGCAAA

GGAAAAGCTAGTTAGAGAAGGAGAGCTTGCTCAATAGTGTGTCTATTCTCAAATGAACTA

TACTAGCGCAATGAGTTGGTGTTGTTCGCTGATGGGAAAAAGGAAACGCATCCCCCTCTT

CTACAAAATTCTGTGGGCCCGGAGGGACTCCGTATGAAAGTGCACAGCATGTGGACGCAT

CACCATGCAATTAGCAAGCTTAGCCAATGTCGTGTCTGTCAGAGTGTGCAGCATGTGAGC

CAAGCGCAATTACAATAAAAAAATAATATAAAAACTCTGCTGAGGTCCGAACCGTCTCAA

ATGCTTTGAGGGCCATCCAAGCCAAGCGCACCTAACGCCTAGCACACCATTGTTGCGCCG

TTCGAGCATTAAtgrkymkkra

>tr3957_1

AACTCATTGAAAGGCCTGTCATCAACCTGTCAACACGGTTCAATGTTTGCTCGAGTGGCG

CAGCAATGGTGTGCTAGGCGTTAGGTGCGCCTGGCTGGGATGGCCCTCAAAGCATTTGAG

ACAGTTCGGACCTCGGCAGAGATTTTATATTATTTTTTTGTTGTAATCGCGTTTGGCCCA

CATGCTGCACACTCCGACAGACACGGCATTGGCTAAACCCGCTAATTGCGTGGTGATGCG

TCCACGTGCTGCGCACTTTCATGCGGAGTCCCCCCGAGCCCGTAGAATTTTGTAGAAGAG

GGGGACGCGTTTCCTTTTTCCCATCAGCAAACAACACCAACTCACTATGCTAGTATAGTT

CATCTGAGAACAGACACACTGCTGAGCAAGCTCTCCTTCTCCAACTAGCTTTTTCTTTGC

TGACATTTCCTACAAAATGGCAGCAAAACAGATTCCGTTAACACAACTGCTGCTCAGGTT

GATTTAGAGGCCGCCTTGGCCGAAGCGACCGGGTCTACATGGAGATTTGCATCAAACTTT

CCTCTTAAAGATGCGATCGCAAAACACTGCCTCGCCAATTGGCTTATCGCTCGAAAGACT

TCAAGCAACCACCAATTGATAGTTCACCGTGAATTCCATGGGCCACACCTGCCGGAAAAC

AGCGTAGGGGCAGTCTGCAAATCTCTCAGACTTTTTAATATGGTCGAGATCAACGTAGTA

CCGACCAAAGATAGAGATTTCATAGTCGTCCACGACTTCAACACAGAAAGAACGACGCAG

TTAGGCAATTGAAATTGGTCTGATATGTCGACGAAAGAGGCCGTTGGCACCCCCCTAGTA

GTTCCCATAATGGGCGGTGACTCCCTAACCAGCGAGTTCACACCCACCGATGAGAAAGTT

GTCAAGCAAGAAACCCTTCTCAGGAATGCCTTTGAAACAAACAAACAGGCAACCATACGG

ATTGACGCTCACGAGAGGCAGGCCGCTCCCCTCGTCGCTTGGCTGAGCCATAGGCCGGCC

TATCACGACAAGACAATCGTTATCTTTCATACATATGGATACAACAATGGACACAAGTTC

GCCTTGGCGATCGATCAAGCCGACGCTACAGAAAATTGGCGCGAAACTGTAGCGCTAATG

CCAAACATTCTGCCAAATGAACTCTCTAGGCTCGCGAAGCAATATCCACCAAATATCACG

TCGCATTTTTACGACCTCGTTGAGGCCGGCAAGAATTGGTTCGCGTCTGTCTTCTCCCAG

AATATGATGGTGGACGTCACTTTCCTCAAGATTGCGGGCACTGGCGAAGGACAGACTAAC

AAAGTCGTTGATCCAAATGGAAATGAGGTTCTGCATCCTAAAGAGAAGGAGGAATATCTC

CGCGATTCAGCTTCAATTGAAATCGCGAAATGAATCTGCGCCACCCATCCCGGAATCGAG

ATTGGAGGCGTCACACGAACTTATGCTTATAAGAGTCGAGGACATCGCCACAAAATCGAT

CTTTGGACGGGTCAACCGAAGCGGTGGCCGACCCACGCGACGAAGTATATCTGGGAAGAT

TTCGCCAAGCCTGGAAATGCGCTCAGATGGGGTCAAACGATTATCTCTGATCGCATTGGC

GACGACCTTGCAGCTTGTGTGCTTCAAAGCCGTGGAGAAAATTTTGTCAGCAGTCATGAA

TATCCCGCTGTTCATGTCGATTAGATGGGCGATCTGTGTCTTAGTCTAAGCTTCCGCTTA

ACGCATCTTTGCTACTTTAATGTATTTTTTTAATGATATTGTTGGAGTTTTCAGCACATC

TTAATCGCAAGTAGATATTTTTTTTGAATCGGATTCGACTTCAAGCAGATGTACTTCTTT

GTCTCTCGATAAATCTAATGACCCTGCGAGCCGGCCTTTCGCCTTAAACTAATTTCTGAG

AGTTAGGTCGGCGCCTCTCGTGGTTTCCATGTTCTATGGAAACGGCCTCTCGGGACTTGC

CTTCCCGCAACTCATCGAGCTTGCCTGGCCCGCTCGGGTGGTGGCGCAAGCCATCGTTAA

ATCGTTCGTAATCCTCTACTCTCGCATAAATGAGAGGGGAATCCAAAGATGATCACTTTT

CAGCTAATTGGAATCTGCAATCCTAAAAGAGGCAAGCACTATGTGGGGTCCACAACTTAT

AGAATTTTGGCCTAACCATTCTGCATAATGGATCATGACATGGGCCATTTATGGCCGCAA

AAACTGGCACAAATTCCAACCTGTTCAGCACAATGAACAAGTTCTTCCAATTGGAATAAA

GGAGGTTAAAATTATTCGAGTAGACATGATGTGCCGGCACGCCACTAGCCTCGCATTGAT

GCCACCCTTTCTTGCGCACACTTATATAAAGCTGAGCTAATAATAGAGATAAAAATAGTC

AGCTTGTAAACTGGACCTTTACTACTGCCCCTAATCTCAAGATCAAAGCGACATATACCT

CCTCTCTTTCAAGTCTTTTTGCAACTTGTTCCAAAGCAAGCGTTCGTGAATCTGCCAGGC

AGCCTTCACGAAAATGGCTGATCAACTATGTGAGGGGCGTTGGTCCGAAGCCAATCTATC

ATTCGTTGGGATATCCATCACCCCTGAATTCATCGATGAGGCCCTCAGAGGCTTTTGCCA

AAAGAGCCGTTGGCTACAAAGAGGTTTACGTTCGGGTTGATCGAGATCCTAAAGTTGCCT

GGGAAGGAGCGCTCAATGGTTACCTCGGCAAATATTCTGGCGTCAAAAATGTTCATTTTG

CGCCGAACAACGCCGCGATTGGAAAGCCCCCATGCCATATCGACATTTTGCTTCGGAAAA

AATTTAGCGGCGAATTTCTTGTGACGCTCGAAGGTGTGGCAATTTCAAACGGTGCAACTG

GTTGGCTTGCGCTCGCGTGCGTAAACCTTCTCTTGAAGGCAAGACAAGATATTGATGTCT

TCTTGTTTGGGGCGGGAAAAGTCGCCGAGGCTGTGATTCTTGCCCTCAATCACGGTGCGG

CTGCAAAAATCAAACCATGGCAGTGCTCAGCCAAGCTCCAATCGAAGGTGGGGTTCCAGC

TCGATGTCGTCATTGATCGGAAGGTTATACCAAAAGCCAAACTCGTCATAACAGCAACAA

ATTCCGAGGAGCTCGCACTCGAGGCCGACGAAATTGCGCCAAATGCAGTAACCATATCCC

TCGGAAAAGATGAATTGCTTGCCGCCTACTTCGATCGCCTTTTGAACGCAGAGGGTCTGA

TTATCGGCGACGATCTGGATGCGATCGAATCGCGCAATGTCAATTCCCTGGTGCTTTACT

ACTCGAAACGTGATTTGAAGCTGACAGAACATGGAAGGGATCATTGGATAAAAAACTACG

CCAATGTCCTTGTTGATCCAGCTCTCATGGAGGAACTTAAGACATGGGAGGGGCCAGCCA

ACTTTTCATCGGTTGGCCTTGCCAGCCTAGACTTGGCGATGGCCGGCTGACTCTACGAAA

CTCTTACTGCGAAACTCTCCCACCCCCAGTAGACAAGACCCTCTTAGGATTGCGCTCTTC

AGAAGGTGCGCCTCCCCAGTCACAACTGGAAAGTCACTCAACTTGTACCCTACTTAGTTT

GTCTCTCTGTCGTGTTTAGTATCAACAACAATAACAAATATTGTAAACAACATATATTTA

TATGGTAATTATATGGCATGTAAGCGTGACAACGATTATGTTTTCCATAGTTTGATAAAC

TCAACACCTGAAAAAGCCGGTGGCCAAAGGGTTGGGACCTCTTTGCACGAACTCCTATGG

CTGCCAGACTATCTACAGAGCTTTTTCGGAAGCTGACGCAGCATCTGATCGCTACACAAT

CCTCGCGGACGTGGATGGCGTGGCTACACGCAAGCAGTTTGACTGAGAGTTTTCGAGTGA

CTATTTTAAAAGGACGATATTTCGTCCCAAGCAATAAAACTCGCTCACATGACTAAGTCG

TTGCTTTAATTCAGCTGCGTGAAAAGATTTAAAGGGCAGAACTGTTTGTGTTTCGTATCC

AGTTCACCGGCAATTATTGGCTACGCATGAAGCTCATTATCGTCGATTCTTTACCCAAAC

ACTGATGTCATTCCAAATGAGTTGGGCCAAAGATGTACTCATGTCGGACAGGCGCGATTT

GGACTTCGTGATCCTCAAGTCTATCGCAGTGCTGCTTTATTACAGGCTATGCTTCCACCC

TCCATTATTTGGTCAACTGGACCTGGCTTTTGACAAGAATAGGCCAGCGTTCATCTATCT

ATCTTTCAAAAACATGCAGTTGCTGCTGCGTAAAGGATGTAACATCGGATTTGGTGTGGA

GACGGTGCTTGCTACGACTATACTGCCTTACTGTAGTGATCTGTCCTGCCAAGCGACGAG

GGGAGCAGCCTGCCTCTCGTGAGCGTCAATCCATACAGTTGCATGTTCGTTTGTTTCAAA

GGCATTCCCAAGAAGGGTTTCCAGCTTGACAACTTTCTCATCGGTGGGTGTGAACTAGCC

GGTTAGTGAGTCACCGCCCATTTTGGGAACTACCAGGGGGGTGCCAACGACCTCCTCCGT

CGACATCTCAGACCAATTTCAATTGCTCAACTGCGCCGTTCTTTCTATGTTGAAGTCATG

GGCGACTATGAAATCTCCATCTTTGGTCGGTACTACCTCGATCTCGACCATATTGAAAAG

CCTGAGAGATTTGCAAATTGCCCCTACACTGTTTTCCGGCAAGTGTGGCCCATGGAACCC

ACGGTGAACTATCAATTGGCGGTCGCTTGAAGTCTTTCGAGCGATAAGCCAATTGGCGAG

GCAGTGTTTCGCGATCGCATCTTTAAGAGGAAAGTCCGATGCAAATCTCCATGTAGACCC

GGTTGCTTCGGCCAAGGCGGCCTCTAAACCAACTTGAGCAGCAGTTGTGTTAAYGGAATC

TATTTTGTCTACCATTTTGTAAGAAATGTCAGCAAAGGAAAAGCTAGTTAGAGAAGGAGA

GCTTGCTCAATAGTGTGTCTATTCTCATATGAACTATACTAGCGCAATGAGTTAGTGTTG

TTCGCTGATGGGAAAAAGGAAATGCATCCCCCTCTTCTACAAAATTCTGTGGGCCCGGAG

GGACTCCGTATGAAAGTGCACAGCATGTGGACGCATCACCATGCAATTAGCGAGCTTAGC

CAATGCCGTGTCTGTCAGAGTGTGCAGCATGTGGGCCAAGCGCAATTACAATAAAAAAAT

AATATAAAAACTCTRCCGAGGTCTGAACCGTCTCAAATGCTTTGAGGGCCATCCAAGCCA

AGCGCACCTAACGCCTAGCACACCATTGTTGCGCCGCTCRAGCATTAATGGTCCGGGACC

TGGTGTCATCTGGAAACCTATGAAGCATGGACACGGCAAAATCAGCGCCGTGGCGGTGTC

AGACACGGGGACACGTACGGGAC

>tr3957_2

TCCAAATTTCCCTCACCTTGAAGTATCAATTTGATCTAGAAGCCAATCCAAACTCATTGA

AAGGCCTGTCATCAACCTGTCAACACGGTTCAATGTTTGCTCGAGTGGCGCAGCAATGGT

GTGCTAGGCGTTAGGTGCGCCTGGCTGGGATGGCCCTCAAAGCATTTGAGACAGTTCGGA

CCTCGGCAGAGATTTTATATTATTTTTTTGTTGTAATCGCGTTTGGCCCACATGCTGCAC

ACTCCGACAGACACGGCATTGGCTAAACCCGCTAATTGCGTGGTGATGCGTCCACGTGCT

GCGCACTTTCATGTGGAGTCCCCCGGGCCCGTAGAATTAGAGGGGGACGCCTTTCCTTTT

TCCCATCAGCAAACAACACCAACTCACTGTGCTAGTATAGTTCATCTGAGAACAGGCACA

CTGCTGAGCAAGCTCTCCTTCTCCAACTAGCTTTTTCTTTGCTGACATTTCCTACAGAAT

GGCAGCAAAACAGATTCCGTTAACACAACTGCTGCTCAAGTTGATTTAGAGGCCGCCTTG

GCCGAAGCGACCGGGTCTACATGGAGATTTGCATCAAACTTTCCTCTTAAAGATGCGATC

GCAAAACACTGCCTCGCCAATTGGCTTATCGCTCGAAAGACTTCAAGCAACCACCAATTG

ATAGTTCACCGTGAATTCCATGGGCCACACCTGCCGGAAAACAGCGTAGGGGCAGTCTGC

AAATCTCTCAGACTTTTTAATATGGTCGAGATCAACGTAGTACCGACCAAAGATAGAGAT

TTCATAGTCATCCACGACTTCAACACAGAAAGAACGACGCAGTTAGACAATTGAAATTGG

TCTGATATGTCAACGAAAGAGGCCATTGGCACCCCCCTAGTAGTTCCCATAATGGGCGGT

GACTCTCTAATCAGCAAGTTCACACCCACCGATGAGAAAGTTGTCAAGCAAGAAACCCTT

CTCAGGAATGCCTTTGAAACAAACAAACAGGCAACCATACGGATTGACGCTCACGAGAGG

CAGGCCGCTCCCCTCGTCGCTTGGCTGAGCCATAGGCCGGCCTATCACGACAAGACAATC

GTAATCTTTCATACATATGGATACAACAATGGACACAAGTTCGCCTTGGCAGTCGATCAA

GCCGACGCTACAGAAAATTGGCGCGAAACTGTAGCGCTAATGCCAAACATTCTGCCGAAT

GAACTCTCTAGGCTCGCGAAGCAATATCCACCAAATATCACGTCGCATTTTTACGACCTC

GTTGAGGCCGGCAAGAATTGGTTCGCGTCTGTCTTCTCCCAGAATATGATGGTGGTCGTC

ACTTTCCTCAAGATTGTGGGCACTGGCGAAGGACAGACTAACAAAGTCGTTGATCCAAAT

GGAAATGAGGTTCTGCATCCTAAAGAGAAGGAGGAATATCTCCGCGATTCAGCTTCAATC

GAAATCGCGAAATGAATCTGCGCCACCCATCCCGGAATCGAGATTGGAGGCGTCACACGA

ACTTATGCTTATAAGAGTCGAGGACATCGCCACAAAATCGATCTTTGGACGGGTCAACCG

AAGCGGTGGCCGACCCACGCGACGAAGTATATCTGGGAAGATTTCGCCAAGCCTGGAAAC

GCGCTCAGATGGGGTCAAACGATTATCTCTGATCGCATTGGCGACGACCTTGCAGCTTGT

GTGCTTCAAAGCCGTGGAGAAAATTTTGTCAGCAGTCATGAATATCCCGCTGTTCATGTC

GATTAGATGGGCGATCTGTGTCTTAGTCTAAGCTTCCGCTTAACGCATCTTTGCTACTTT

AATGTATTTTTTTAATGATATTGTTGGAGTTTTCAGCACATCTTAATCGCAAGTAGATAT

TTTTTTTGAATCGGATTCGACTTCAAGCAGATGTACTTCTTTGTCTCTCGATAAATCTAA

TGACCCTGCGAGCCGGCCTCTCGCCTTAAACTAATTTCTGAGAGTTAGGTCGGCGCCTCT

CGTGGTTTCCATGTTCTATGGAAACGGCCTCTCGGGACTTACCTTCCCGTAACTCATCGA

GCTTGCCTGGCCCGCACGGGTGGTGGCGCAAGCCATCGTTAAATCGTTCGTAATCCTCTA

CTCTCGCATAAATGAGAGGGGAATCCAAATATGATCACTTTTCAGCTAATTGGAATCTGC

AATCCTAAAAGAGGCAAGCACTATGTGGGGTCCACAACTTATAGAATTTTGGCCTAACCA

TTCTGCATAATGGATCATGACATGGGCCATTTATGGCCGCAAAAACTGGCACAAATTCCA

ACCTGTTCAGCACAATGAACAAGTTCTTCCAATTGGAATAAAGGAGGTTAAAATTATTCG

AGTAGACATGATGTGCCGGCACGCCACTAGCCTCGCATTGATGCCACCCTTTCTTGCGCA

CACTTATATAATGCTGAGCTAATAATAGAGATAAAAATAGTCAGCTTGTAAACTGGACCT

TTACTACTGCCCCTAATCTCAAGATCAAAGCGACATATACCTCCTCTCTTTCAAGTCTTT

TTGCAACTTGTTCCAAAGCAAGCGTTCGTGAATCTGCTAGGCAGCCTTCACGAAAATGGC

TGATCAACTATGTGAGGGGCGTTGGTCCGAAGCCAATCTATCATTCGTTGGGATATCCAT

CACCCCTGAATTCATCGATGAGGCCCTCAGAGGCTTTTGCCAAAAGAGCCGTTGGCTACA

AAGAGGTTTACGTTCGGGTTGATCGAGATCCTAAAGTTGCCTGGGAAGGAGCGCTCAATG

GTTACCTCGGCAAATATTCTGGTGTCAAAAATGTTCATTTTGCGCCGAACAACGCCGCGA

TTGGAAAGCCCCCACGCCATATCGACATTTTGCTTCGGAAAAAATTTAGCGGCGAATTTC

TTGCGACGCTCGAAGGTGTGGCAATTTCAAACGGTGCAACTGGTTGGCTTGCGCTCGCAT

GCGTAAACCTTCTCTTGAAGGCAAGACAAGATATTGATGTCTTCTTGTTTGGGGCGGGAA

AAGTCGCCGAGGCTGTGATTCTTGCCCTCAATCACGGTGCGGCTGCAAAAATCAAAACCA

TGGCAGTGCTCAGCCAAGCTTCAATCGAAGGTGGGGTTCCAACTCGATGTCGTCATTGAT

CAAAAGGTTATACCAAAAGCCAACCTCGCCATAACAGCAACAAATTCCGAGGAGCTCGTA

CTCGAGGCCGACGAAATTGCGCCAAATGCAGTAACCATATCCCTCGGAAAAGATGAATTG

CTCGCCGCCTACTTCGATCGCCTTTTGAACGCAGAGGGTCTGATTATCGGCGACGATCTG

GATGCGATCGAATCGCGCAATGTCAATTCCCTGGTGCTTTACTACTTGAAACGTGATTTG

AAGCTGACAGAACATGGAAGGGATCATTAGATAAAGAACTACGCCAATGTCCTTGTTGAT

CCAGCTCTCATGGAGGAACTTAAGACATGGGAGGGGCCAGCCAACTTTTCATCGGTTGGC

CTTGCCAGCCTAGACTTGGCGATGGCCGGCCGACTCTACGAAACTCTTACTGCGAAACTC

TCCCACCCCCAGTAGACAAGACCCGCTTAGGATTGCGCTCTTCAGAAGGTGCGCCTCCCC

AGTCACAACCGGAAAGTCACTCAACTTGGCTCAAGTACCCTACTTAGTTTGTCTCTCTGT

CGTGTTTAGTATCAACAACAATAACAAATATTGTAAACAACATATATTTATATGGTAATT

ATATGGCATGTAAGCGTGACAACGATTATGTTTTCCATAGTTTGATAAACTCAACACCCG

AAAAAGCTGGTGACCAAAGGGTTGGGACCTCTTTGCACGAACTCCTATGGCTGCCAGACT

ATCTACAGAGCTTTTTCGGAAGCTGACGCAGCATCTGATCGCTACACAATCCTCGCGGAC

GTGGATGGCGTGGCTACACGCAAGCAGTTTGACTGAGAGTTTTCGAGTGACTATTTTAAA

AGGACGATATTTCGTCCCAAGCAATAAAACTCGCTCACATGACTAAGTCGTTGCTTTAAT

TCAGCTGCGTGAAAAGATTTAAAGGGCAGAACTGTTTGTGTTTCGTATCCAGTTCACCGG

CAATTATTGGCTACGCATGAAGCTCATTATCGTCGATTCTTTACCCAAACACTGATGTCA

TTCCAAATGAGTTGGGCCAAAGATGTACTCATGTCGGACAGGCGCGATTTGGACTTCGTG

ATCCTCAAGTCTATCGCAGTGCTGCTTTATTACAGGCTATGCTTCCACCCTCCATTATTT

GGTCAACTGGACCTGGCTTTTGACAAGAATAGGCCAGCGTTCATCTGTCTATCTTTCAAA

ACCATGCAGTTGCTGCTGCGTAAAGGATGTAACATCGGATTTGGTGTGGAGACGGTGCTT

GCTACGACTATACTGCCTTACTGTAGTGATCTGTCCTGCCAAGCGACGAGGGGAGCAGCC

TGCCTCTCGTGAGCGTCAATCCATACGGTTGCATGTTCGTTTGTTTCAAAGGCATTCCCA

AGAAGGGTTTCCAGCTTGACAACTTTCTCATTGGTGGGTGTGAACTCGCCGGTTAGTGAG

TCACCGCCCATTTTGGGAACTACCAGGGGGGTGCCAACGACCTCCTCCGTCGACATCTCA

GACCAATTTCAATTGCTCAACTGCGCCGTTCTTTCTATGTTGAAGTCATGGGCGACTATG

AAATCTCCATCTTTGGTCGGTACTACCTCGATCTCGACCATATTGAAAAGCCTGAGAGAT

TTGCAAACTGCCCCTACACTGTTTTCCAGCAAGTGTGGCCCATGGAATCCACGGTGAACT

ATCAATTGGCGGTCGCTTGAAGTCTTTCGAGCGATAAGCCAATTGGCGAGGCAGTGTTTC

GCGATCGCATCTTTAAGAGGAAAGTCCGATGCAAATCTCCATGTAGACCCGGTCGCTTCG

GCCAAGGCGGCCTCTAAACCAACTTGAGCAGCAGTTGTGTTAAYGGAATCTATTTTGTCT

ACCATTTTGTAAGAAA

>tr3967_1

gtccacgtgctgcgcactttcatgcggagtccccccgagcccgtagaattttgtagaaga

gggggacgcgtttcctttttcccatcagcaaacaacaCCAACTCACTGTGCTAGTATAGT

TCATCTGAGAACAGGCACACTGCTGAGCAAGCTCTCCTTCTCCAACTAGCTTTTTCTTTG

CTGACATTTCCTACAGAATGGCAGCAAAACAGATTCCGTTAACACAACTGCTGCTCAAGT

TGATTTAGAGGCCGCCTTGGCCGAAGCGACCGGGTCTACATGGAGATTTGCATCAAACTT

TCCTCTTAAAGATGCGATCGCAAAACACTGCCTCGCCAATTGGCTTATCGCTCGAAAGAC

TTCAAGCAACCACCAATTGATAGTTCACCGTGAATTCCATGGGCCACACCTGCCGGAAAA

CAGCGTAGGGGCAGTCTGCAAATCTCTCAGACTTTTTAATATGGTCGAGATCAACGTAGT

ACCGACCAAAGATAGAGATTTCATAGTCATCCACGACTTCAACACAGAAAGAACGACGCA

GTTAGACAATTGAAATTGGTCTGATATGTCAACGAAAGAGGCCATTGGCACCCCCCTAGT

AGTTCCCATAATGGGCGGTGACTCTCTAATCAGCAAGTTCACACCCACCGATGAGAAAGT

TGTCAAGCAAGAAACCCTTCTCAGGAATGCCTTTGAAACAAACAAACAGGCAACCATACG

GATTGACGCTCACGAGAGGCAGGCCGCTCCCCTCGTCGCTTGGCTGAGCCATAGGCCGGC

CTATCACGACAAGACAATCGTAATCTTTCATACATATGGATACAACAATGGACACAAGTT

CGCCTTGGCAGTCGATCAAGCCGACGCTACAGAAAATTGGCGCGAAACTGTAGCGCTAAT

GCCAAACATTCTGCCGAATGAACTCTCTAGGCTCGCGAAGCAATATCCACCAAATATCAC

GTCGCATTTTTACGACCTCGTTGAGGCCGGCAAGAATTGGTTCGCGTCTGTCTTCTCCCA

GAATATGATGGTGGTCGTCACTTTCCTCAAGATTGTGGGCACTGGCGAAGGACAGACTAA

CAAAGTCGTTGATCCAAATGGAAATGAGGTTCTGCATCCTAAAGAGAAGGAGGAATATCT

CCGCGATTCAGCTTCAATCGAAATCGCGAAATGAATCTGCGCCACCCATCCCGGAATCGA

GATTGGAGGCGTCACACGAACTTATGCTTATAAGAGTCGAGGACACCGCTACAAAATCGA

TCTTTGGACAGGTCAACCGAAGCGGTGGCCGACCCACGCGACGAAGTATATCTGGGAAGA

TTTCGCCAAGCCTGGAAACGCGCTCAGATGGGGTCAAACGATTATCTCTGATCGCATTGG

CGACGACCTTGCAGCTTGTGTgcttcaaagccgtggagaaaattttgtcaGCAGTCATGA

ATATCCCGCTGTTCATGTCGATTAGATGGGCGATCTGTGTCTTAGTCTAAGCTTCCGCTT

AACGCATCTTTGCTACTTTAATGTATTTTTTTAATGATATTGTTGGAGTTTTCAGCACAT

CTTAATCGCAAGTAGATATTTTTTTTGAATCGGATTCGACTTCAAGCAGATGTACTTCTT

TGTCTCTCGATAAATCTAATGACCCTGCGAGCCGGCCTCTCGCCTTAAACTAATTTCTGA

GAGTTAGGTCGGCGCCTCTCGTGGTTTCCATGTTCTATGGAAACGGCCTCTCGGGACTTA

CCTTCCCGTAACTCATCGAGCTTGCCTGGCCCGCTCGGGTGGTGGCGCAAGCCATCGTTA

AATCGTTCGTAATCCTCTACTCTCGCATAAATGAGAGGGGAATCCAAATATGATCACTTT

TCAGCTAATTGGAATCTGCAATCCTAAAAGAGGCAAGCACTATGTGGGGTCCACAACTTA

TAGAATTTTGGCCTAACCATTCTGCATAATGGATCATGACATGGGCCATTTATGGCCGCA

AAAACTGGCACAAATTCCAACCTGTTCAGCACAATGAACAAGTTCTTCCAATTGGAATAA

AGGAGGTTAAAATTATTCGAGTAGACATGATGTGCcggcacgccactagcctcgcattga

tgccATCCTTTCTTGCGCACACTTATATAATGCTGAGCTAATAATAGAGATAAAAATAGT

CAGCTTgtAAACTGGACCTTTACTACTGCCCCTAATCTCAAGATCAAAGCGACATATACC

TCCTCTCTTTCAAGTCTTTTTGCAACTTGTTCCAAAGCAAGCGTTCGTGAATCTGCTAGG

CAGCCTTCACGAAAATGGCTGATCAACTATGTGAGGGGCGTTGGTCCGAAGCCAATCTAT

CATTCGTTGGGATATCCATCACCCCTGAATTCATCGATGAGGCCCTCAGAGGCTTTTGCC

AAAAGAGCCGTTGGCTACAAAGAGGTTTACGTTCGGGTTGATCGAGATCCTAAAGTTGCC

TGGGAAGGAGCGCTCAATGGTTACCTCGGCAAATATTCTGGTGTCAAAAATGTTCATTTT

GCGCCGAACAACGCCGCGATTGGAAAGCCCCCACGCCATATCGACATTTTGCTTCGGAAA

AAATTTAGCGGCGAATTTCTTGCGACGCTCGAAGGTGTGGCAATTTCAAACGGTGCAACT

GGTTGGCTTGCGCTCGCATGCGTAAACCTTCTCTTGAAGGCAAGACAAGATATTGATGTC

TTCTTGTTTGGGGCGGGAAAAGTCGCCGAGGCTGTGATTCTTGCCCTCAATCACGGTGCG

GCTGCAAAAATCAAAACCATGGCAGTGCTCAGCCAAGCTTCAATCGAAGGTGGGGTTCCA

ACTCGATGTCGTCATTGATCAAAAGGTTATACCAAAAGCCAAACTCGTCATAACAGCAAC

AAATTCCGAGGAGCTCGTACTCGAGGCCGACGAAATTGCGCCAAATGCAGTAACCATATC

CCTCGGAAAAGATGAATTGCTCGCCGCCTACTTCGATCGCCTTTTGAACGCAGAGGGTCT

GATTATCGGCGACGATCTGGATGCGATCGAATCGCGCAATGTCAATTCCCTGGTGCTTTA

CTACTTGAAACGTGATTTGAAGCTGACAGAACATGGAAGGGATCATTAGATAAAGAACTA

CGCCAATGTCCTTGTTGATCCAGCTCTCATGGAGGAACTTAAGACATGGGAGGGGCCAGC

CAACTTTTCATCGGTTGGCCTTGCCAGCCTAGACTTGGCGATGGCCGGCCGACTCTACGA

AACTCTTACTGCGAAACTCTCCCACCCCCAGTAGACAAGACCCGCTTAGGATTGCGCTCT

TCAGAAGGTGCGCCTCCCCAGTCACAACCGGAAAGTCACTCAACTTGGCTCAAGTACCCT

ACTTAGTTTGTCTCTCTGTCGTGTTTAGTATCAACAACAATAACAAATATTGTAAACAAC

ATATATTTATATGGTAATTATATGGCATGTAAGCGTGACAACGATTATGTTTTCCATAGT

TTGATAAACTCAACACCTGAAAAAGCCGGTGACCAAAGGGTTGGGACCTCTTTGCACGAA

CTCCTATGGCTGCCAGACTATCTACAGAGCTTTTTCGGAAGCTGACGCAGCATCTGATCG

CTACACAATCCTCGCGGACGTGGATGGCGTGGCTACACGCAAGCAGTTTGACTGAGAGTT

TTCGAGTGACTATTTTAAAAGGACGATATTTCGTCCCAAGCAATAAAACTCGCTCACATG

ACTAAGTCGTTGCTTTAATTCAGCTGCGTGAAAAGATTTAAAGGGCAGAACTGTTTGTGT

TTCGTATCCAGTTCACCGGCAATTATTGGCTACGCATGAAGCTCATTATCGTCGATTCTT

TACCCAAACACTGATGTCATTCCAAATGAGTTGGGCCAAAGATGTACTCATGTCGGACAG

GCGCGATTTGGACTTCGTGATCCTCAAGTCTATCGCAGTGCTGCTTTATTACAGGCTATG

CTTCCACCCTCCATTATTTGGTCAACTGGACCTGGCTTTTGACAAGAATAGGCCAGCGTT

CATCTGTCTATCTTTCAAAAACATGCAGTTGCTGCTGCGTAAAGGATGTAACATCGGATT

TGGTGTGGAGACGGTGCTTGCTACGACTATACTGCCTTACTGTAGTGATCTGTCCTGCCA

AGCGACGAGGGGAGCAGCCTGCCTCTCGTGAGCGTCAATCCATACGGTTGCATGTTCGTT

TGTTTCAAAGGCATTCCCAAGAAGGGTTTCCAGCTTGACAACTTTCTCATTGGTGGGTGT

GAACTCGCCGGTTAGTGAGTCACCGCCCATTTTGGGAACTACCAGGGGGGTGCCAACGAC

CTCCTCCGTCGACATCTCAGACCAATTTCAATTGCTCAACTGCGCCGTTCTTTCTATGTT

GAAGTCATGGGCGACTATGAAATCTCCATCTTTGGTCGGTACTACCTCGATCTCGACCAT

ATTGAAAAGCCTGAGAGATTTGCAAACTGCCCCTACACTGTTTTCCAGCAAGTGTGGCCC

ATGGAATCCACGGTGAACTATCAATTGGCGGTCGCTTGAAGTCTTTCGAGCGATAAGCCA

ATTGGCGAGGCAGTGTTTCGCGATCGCATCTTTAAGAGGAAAGTCCGATGCAAATCTCCA

TGTAGACCCGGTCGCTTCGGCCAAGGCGGCCTCTAAACCAACTTGAGCAGCAGTTGTGTT

AATGGAATCTATTTTGACTGCCATTCTGTAAGAAATGTCAGCAAAGGAAAAGCTAGTTAG

AGAAGGAGAGCTTGCTCAATAGTGTGTCTATTCTCATATGAACTATACTAGCGTAATGAG

TTAGTGTTGTTCGCTGATGGGAAAAAGGAAATGCATCCCCCTCTTCTACAAAATTCTGTG

GGCCCGGGGGGACTCCGTATGAAAGTGCACAGCATGTGGACGCATCACCATGCAATTAGC

GAGCTTAGCCAATGCCGTGTCTGTCAGAGTGTGCAGCATGTGGGCCAAGCGCAATTACAA

TAAAAAAATAATATAAAAACTCTACCGAGGTCTGAACCGTCTCAAATGCTTTGAGGGCCA

TCCAAGCCAAGCGCACCTAACGCCTAGCACACCATTGTTGCGCCGCTCAAGCATTAATGG

TCCGGGACCTGGTGTCATCtg

>tr3967_2

gcctctccaaatttccctcaccttgaagtatcaatttgatctagaagccaatccaaactc

attgaaaggcctgtcatcaacctgtcaacacggttcaatgtttgctcgagtggcgcagca

atggtgtgctaggcgttaggtgcgcctggctgggatggccctcaaagcatttgagacagt

tcggacctcggcagagattttatattatttttttgttgtaatcgcgtttggcccacatgc

tgcacactccgacagacacggcattggctaaacccgctaattgcgtggtgatgcGTCCAC

GTGCTGCGCACTTTCATGCGGaGTCCCCCCGAGCCCGTAGAATTTTGTAGAAGAGGGGGA

CGCGTTTCCTTTTTCCCATCAGCAAACAACACCAACTCACTATGCTAGTATAGTTCATCT

GAGAACAGACACACTGCTGAGCAAgctctccttctccaactaGCTTTTTCTTTGCTGACA

TTTCCTACAAAATGGCAGCAAAACAGATTCCGTTAACACAACTGCTGCTCAGGTTGATTT

AGAGgccgccttggccgaagcgaccggGTCTACATGGAGATTTGCATCAAACTTTCCTCT

TAAAGATGCGATCGCAAAACACTGCCTCGCCAATTGGCTTATCGCTCGAAAGACTTCAAG

CAACCACCAATTGATAGTTCACCGTGAATTCCATGGGCCACACCTGCCGGAAAACAGCGT

AGGGGCAGTCTGCAAATCTCTCAGACTTTTTAATATGGTCGAGATCAACGTAGTACCGAC

CAAAGATAGAGATTTCATAGTCGTCCACGACTTCAACACAGAAAGAACGACGCAGTTAGG

CAATTGAAATTGGTCTGATATGTCGACGAAAGAGGCCGTTGGCACCCCCCTAGTAGTTCC

CATAATGGGCGGTGACTCCCTAACCAGCGAGTTCACACCCACCGATGAGAAAGTTGTCAA

GCAAGAAACCCTTCTCAGGAATGCCTTTGAAACAAACAAACAGGCAACCATACGGATTGA

CGCTCACGAGAGGCAGGCCGCTCCCCTCGTCGCTTGGCTGAGCCATAGGCCGGCCTATCA

CGACAAGACAATCGTTATCTTTCATACATATGGATACAACAATGGACACAAGTTCGCCTT

GGCGATCGATCAAGCCGACGCTACAGAAAATTGGCGCGAAACTGTAGCGCTAATGCCAAA

CATTCTGCCAAATGAACTCTCTAGGCTCGCGAAGCAATATCCACCAAATATCACGTCGCA

TTTTTACGACCTCGTTGAGGCCGGCAAGAATTGGTTCGCGTCTGTCTTCTCCCAGAATAT

GATGGTGGACGTCACTTTCCTCAAGATTGCGGGCACTGGCGAAGGACAGACTAACAAAGT

CGTTGATCCAAATGGAAATGAGGTTCTGCATCCTAAAGAGAAGGAGGAATATCTCCGCGA

TTCAGCTTCAATTGAAATCGCGAAATGAATCTGCGCCACCCATCCCGGAATCGAGATTGG

AGGCGTCACACGAACTTATGCTTATAAGAGTCGAGGACATCGCCACAAAATCGATCTTTG

GACGGGTCAACCGAAGCGGTGGCCGACCCACGCGACGAAGTATATCTGGGAAGATTTCGC

CAAGCCTGGAAATGCGCTCAGATGGGGTCAAACGATTATCTCTGATCGCATTGGCGACGA

CCTTGCAGCTTGTGTGCTTCAAAGCCGTGGAGAAAATTTTGTCAGCAGTCATGAATATCC

CGCTGTTCATGTCGATTAGATGGGCGATCTGTGTCTTAGTCTAAGCTTCCGCTTAACGCA

TCTTTGCTACTTTAATGTATTTTTTTAATGATATTGTTGGAGTTTTCAGCACATCTTAAT

CGCAAGTAGATATTTTTTTTGAATCGGATTCGACTTCAAGCAGATGTACTTCTTTGTCTC

TCGATAAATCTAATGACCCTGCGAGCCGGCCTTTCGCCTTAAACTAATTTCTGAGAGTTA

GGTCGGCGCCTCTCGTGGTTTCCATGTTCTATGGAAACGGCCTCTCGGGACTTGCCTTCC

CGCAACTCATCGAGCTTGCCTGGCCCGCTCGGGTGGTGGCGCAAGCCATCGTTAAATCGT

TCGTAATCCTCTACTCTCGCATAAATGAGAGGGGAATCCAAAGATGATCACTTTTCAGCT

AATTGGAATCTGCAATCCTAAAAGAGGCAAGCACTATGTGGGGTCCACAACTTATAGAAT

TTTGGCCTAACCATTCTGCATAATGGATCATGACATGGGCCATTTATGGCCGCAAAAACT

GGCACAAATTCCAACCTGTTCAGCACAATGAACAAGTTCTTCCAATTGGAATAAAGGAGG

TTAAAATTATTCGAGTAGACATGATGTGCCGGCACGCCACTAGCCTCGCATTGATGCCAC

CCTTTCTTGCGCACACTTATATAATGCTGAGCTAATAATAGAGATAAAAATAGTCAGCTT

GTAAACTGGACCTTTACTACTGCCCCTAATCTCAAGATCAaagcgacatatacctcctct

CTTTCAAGTCTTTTTGCAACTTGTTCCAAAGCAAGCGTTCGTGAATCTGCTAGGCAGCCT

TCACGAAAATGGCTGAtcaactatgtgaggggcgttGGTCCGAAGCCAATCTATCATTCG

TTGGGATATCCAcCACCCCTGAATTCATCGATGAGGCCCTCAGAGGCTTTTGCCAAAAGA

GCCGtTGGCTACAAAGAGGTTTACGTTCGGGTTGATCGAGATCCTAAAGTTGCCtGGGAA

GGAGCGctcaatggttacctcggcaaatattCTGGCGTCAAAAATGTTCATTTTGCGCCG

AACAACGCCGCGATTGGAAAGCCCCCATGCCATATCGACATTTTGCTTCGGAAAAAATTT

AGCGGCGAATTTCTTGTGACGCTCGAAGGTGTGGCAATTTCAAACGGTGCAACTGGTTGG

CTTGCGCTCGCGTGCGTAAACCTTCTCTTGAAGGCAAGACAAGATATTGATGTCTTCTTG

TTTGGGGCGGGAAAAGTCGCCGAGGCTGTGATTCTTGCCCTCAATCACGGTGCGGCTGCA

AAAATCAAACCATGGCAGTGCTCAGCCAAGCTCCAATCGAAGGTGGGGTTCCAGCTCGAT

GTCGTCATTGATCGGAAGGTTATACCAAAAGCCAAACTCGTCATAACAGCAACAAATTCC

GAGGAGCTCGCACTCGAGGCCGACGAAATTGCGCCAAATGCAGTAACCATATCCCTCGGA

AAAGATGAATTGCTTGCCGCCTACTTCGATCGCCTTTTGAACGCAGAGGGTCTGATTATC

GGCGACGATCTGGATGCGATCGAATCGCGCAATGTCAATTCCCTGGTGCTTTACTACTCG

AAACGTGATTTGAAGCTGACAGAACATGGAAGGGATCATTGGATAAAAAACTACGCCAAT

GTCCTTGTTGATCCAGCTCTCATGGAGGAACTTAAGACATGGGAGGGGCCAGCCAACTTT

TCATCGGTTGGCCTTGCCAGCCTAGACTTGGCGATGGCCGGCCGACTCTACGAAACTCTT

ACTGCGAAACTCTCCCACCCCCAGTAGACAAGACCCTCTTAGGATTGCGCTCTTCAGAAG

GTGCGCCTCCCCAGTCACAACCGGAAAGTCACTCAACTTGGCTCAAGTACCCTACTTAGT

TTGTCTCTCTGTCGTGTTTAGTATCAACAACAATAACAAATATTGTAAACAACATATATT

TATATGGTAATTATATGGCATGTAAGCGTGACAACGATTATGTTTTCCATAGTTTGATAA

ACTCAACACCCGAAAAAGCTGGTGACCAAAGGGTTGGGACCTCTTTGCACGAACTCCTAT

GGCTGCCAGACTATCTACAGAGCTTTTTCGAAAGCTGACGCAGCATCTGATCGCTACACA

ATCCTCGCGGACGTGGATGGCGTGGCTACACGCAAGCAGTTTGACTGAGAGTTTTCGAGT

GaCTATTTTAAAAGGACgaTATTtcgtcccaagcaataaaactcgctcacatgactaagt

cgttgctttaattcagctgcgtgaaaagatttaaagggcagaactgtttgtgtttcgtat

ccaGTTCACCGGCAATTATTGGCTACGCATGAAGCTCATTATCGTCGATtctttacccaa

acactgatgtcATTCCAAATGAGTTGGGCCAAAGATGTACTCATGTCGGACAGGCGCGAT

TTGGACTTCGTGATCCTCAAGTCTATCGCAGTGCTGCTTTATTACAGGCTATGCTTCCAC

CCTCCATTATTTGGTCAACTGGACCTGGCTTTTGACAAGAATAGGCCAGCGTTCATCTAT

CTATCTTTCAAAAACATGCAGTTGCTGCTGCGTAAAGGATGTAACATCGGATTTGGTGTG

GAGACGGTGCTTGCTACGACTATACTGCCTTACTGTAGTGATCTGTCCTGCCAAGCGACG

AGGGGAGCAGCCTGCCTCTCGTGAGCGTCAATCCATACAGTTGCATGTTCGTTTGTTTCA

AAGGCATTCCCAAGAAGGGttTCCAGCTTGACAACTTTCTCATtGGTGGGTGTGAACTAG

CCGGTTAGTGAGTCACCGCCCATTTTGGGAACTACCAGGGGGGTGCCAACGACCTCCTCC

GTCGACATCTCAGACCAATTTCAATTGCTCAACTGCGCCGTTCTTTCTATGTTGAAGTCA

TGGGCGACTATGAAATCTCCATCTTTGGTCGGTACTACCTCGATCTCGACCATATTGAAA

AGCCTGAGAGATTTGCAAATTGCCCCTACACTGTTTTCCGGCAAGTGTGGCCCATGGAAT

CCACGGTGAACTATCAATTGGCGGTCGCTTGAAGTCTTTCGAGCGATAAGCCAATTGGCG

AGGCAGTGTTTCGCGATCGCATCTTTAAGAGGAAAGTCCGATGCAAATCTCCATGTAGAC

CCGGTTGCTTCGGCCAAGGCGGCCTCTAAACCAACTTGAGCAGCAGTTGTGTTAACGGAA

TCTATTTTGACTACCATTTTGTAAGAAATGTCAGCAAAGGAAAAGCTAGTTAGAGAAGGA

GAGCTTGCTCAATAGTGTGTCTATTCTCATATGAACTATACTAGCGCAATGAGTTAGTGT

TGTTCGCTGATGGGAAAAAGGAAATGCATCCCCCTCTTCTACAAAATTCTGTGGGCCCGG

AGGGACTCCGTATGAAAGTGCACAGCATGTGGACGCATCACCATGCAATTAGCGAGCTTA

GCCAATGCCGTGTCTGTCAGAGTGTGCAGCATGTGGGCCAAGCGCAATTACAATAAAAAA

ATAATATAAAAACTCTGCCGAGGTCTGAACCGTCTCAAATGCTTTGAGGGCCATCCAAGC

CAAGCGCACCTAACGCCTAGCACACCATTGTTGCGCCGCTCGAGCATTAATGGTCCGGGA

CCTGGTGTCATCTGGAAACCTATGAAGCATGGACACGGCAAAATCAGCGCCGTGGCGGTG

TCAGACACGGGGACACGTACGGGACACGTccatct

>tr3979_1

ctggcttggatggccctcaaagcaTTTGAGGCAGTTCGGACCTCGGCAGAGATTTTATAT

TATTTTTTTGTTGTAATCGCGCTTGGCCCACATGCTGCACACTCCGATAGACACGGCATT

GGCTAAACCCGCTAATTGCGTGGTGATGCGTCCACGTGCTGCGCACTTTCATGCGGAGTC

CCCCCGAGTCCATAGAATAAGAGGGGGACGCGTTTCCTTTTTTCCATCAGCAAACAACAC

CAACTCACTGTGCTAGTATAGTTCATCTGAGAACAGACACACTGCTGAGCAAGCTCTCCt

tctccaactAGCTTTTTCTTTGCTGACATTTCCTACAGAATGGCAGCAAAACAGATTCCG

TTAACACAACTACTGCTCAGGTTGATTTAGAGGCCGCCTTGGCCGAAGCGATCGGGTCTA

CATGGAGATTTGCATCAGACTTTCCTCTTAAAAATGCAATCGCGAAACACTACCTTGCCA

ATTGGCTTATCGCTCGAAAGACTTCAAGCAACCACCAATTGATAGTTCACCGTGAATTCC

ATGGGCCACACCTGCCGGAAAACAGCGTAGGGGCAGTCTGCAAATCTCTCAGACTTTTTA

ATATGGTCGAGATCAACGTAGTACCGACCAAAGATAAAGATTTCATAGTCGTCCACGACT

TCAACACAGAAAGAACGACGCAGttaGGCAATTGAAATTGGTTTGATATGTCGACGAAAG

AGGCTGTTGGCACCCCCCTGGTAGTTCCCATAATGGGCGGTGACTCCCTAAAGAGCGAGT

TCACACCCACCGATGAGAAAGTTGTCAAGCAAGAAACCCTTCTCAAGAATGCCTTTGAAA

CAAACAAACAGGCAACCATACGGATTGACGCTCACGAGAGGCAGGCCGCTCCCCTCGTCG

CTTGGCTGAGCCATAGGCCGGCCTATCACGACAAGACAATCGTTATCTTTCATACATATG

GATACAACAATGGACACGAGTTCGCCTTGGCGGTCGATCAAGCCGGCGCTACAAAAAATT

GGCGCGAAACTGTAGCGCTAATGCCAAACATTCTGCCGAATGAACTCTCTAGGCTCGCGA

AGCAATATCCACCAAATTGCACGTCGCATTTTTACGACCTCGTTGAGGCCAGCAAGAATT

GGTTCGCATCTGTCTTCTCCCAGAATATGATGGTGGTCGTCACTTTCCTCAAGATTGCGG

GCACTGGCAAAGGACAGACTAACAAAGTCGTTGATCCAAATGGAAATGAGGTTCTGCATC

CTAAAGAGAAGGAGGAATATCTCCGCGATTCAGCTTCAATCGAAATCGCGAAATGAATCT

GCGCCACCCATCCCGGAATCGAGATTGGAGGCGTCACACGAACTTATGCTTATAAGAGTC

GAGGACATCGCTACAAAATCAATCTTTGGACGGGTCAACCGAAGCGGTGGCCGACCCACG

CGACGAAGTACATCTGGGAAGATTTCGCCAAGCCTGGAAATGCGCTCAGATGGGGTCAAA

CGATTATCTCTGATCGCATTGGTGACGACCTTGCAGCTTGTGTGCTTCAAAGCCGTGGAG

AAAATTTTGTCAGCAGTCATGAATATCCTGCTGTTCATGTCGATTAGATGGGCGATCTGT

GTCTTAGTCTAAGCTTCCGCTTAACGCATCTTTGCTACTTTAATGTATTTTTTTAATGAT

ATTGTTGGAGTTTTGAGCACATCTTAATCGCAAGTAGATATTTTTTTTGAATCGGATTCG

ACTTCAAGCAGATGTACTTCTTTGTCTCTCGATAAATCTAATGACCCTGCGAGCCAGCCT

CTCGCCTTAAACTAATTTCTGAGAGTTAGGTCGGCGCCTCTCGTGGTTTCCATGtTCTAt

GGaaacggccgctcgggacttgccttcccgcaactcatCGAGCTTGCCTGGCCCGCTCAG

GTGGTGGCACAAGCCATCGTTAAATCGTTCGTAATCCTCTACTCTCGCATAAATGAGAAG

GGAATCCAAAGATGATCACTTTTCAGCTAATTGGAATCTGCAATCCCAAAAGAGGCAAGC

ACTATGTGGGGTCCACAACTTATAGAATTTTGGCCTAACCATTCTGCATAATGGATCATG

ACATAGGCCATTTATGGCCGCAAAAACTGGCACAAATTCCAACCTATTCAGCACAATGAA

CAAGTTCTTCCAATTGGAATAAAGGAGGTTAAAATTATTCAAGTAGACATGACGTGCTGG

CACGCCACTAGCCTCGCATTGATGCCACTCTTTCTTGCGCACACTTATATAATGCTGAGC

TAATAATAGAGATAAAAATAGTCAGCTTGTAAACTGGACCTTTATTACTGCCCCTAATCT

CAAGATCAAAGCAACATATACCTCTTCTCTTTCAAGTCTTTTTGCAACTTGTTCTAAAGC

AAGCGTTTGTGAATCTGCCAAGCAGCCTTCACGAAAATGGCTGATCAACGGTGTGAGGGG

CGTTGGTCCGAAGCCAATCTACCATTCGTTGGGATATCCATCACCCCTGAATTCATCGAT

GAGGCCCtCAGaGGCTTTTGGGAGTCTTTTGCcaaaagagccgttggctacaaagaggtt

tacgttcaggttgatcgagatcctaaagttgcctgggaaggaGcGCTCAATGGTTACCTC

GGCAAATATTCTGGCGTAAAAAATGTTCATTTTGCGCCGAACAACGCCACGATTGGAAAG

CCCCCACGCCATATTGACATTTTGCTTCGGAAAAAATTTAGCGGCGAACTTCTTGCGATG

CTCGAAGGTGTGGCAATttcaaacggtgcaactggttggcttgcgcTCGCATGTGTAAAC

CTTCTCTTGAAGGCAAGACAAGATATTGATGTCTTCTTGTTTGGGGCAGGAAAAGTCGCC

GAGGCTGTGATTCTTGCCCTCAATCATGGTGCGGCTGCAAAAATCAAAACCATGGCAGTG

CTCAGCCAAGCTCCAATTGAAGGTGGGGTTCCAGCTCGATGCCGTCATTGATCGGAAGGT

TATACCAAAAGCCAAACTCGTCATAACAGCAACAAATTCCGAGGAGCTCGGTACTCGAGG

CCGACGAAATTGCGCCAAATGCAGTAACCACATCCCTCGGAAAAGATGAATTGCCCGCCG

CCTACTTCGATCGCCTTTTGAACGCAGAGGGTCTGATTATTGGCGACGATCTGGATGTGA

TCGAATCGCGCAATGTCAATTCCCTGGTGCTTTACTACTCGAAACGTGATTTGAAACTGA

CAGAACATGGAAGGGATCATTGGATAAAGAACTACGCCAATGTCCTTGCTGATCCAGCTC

TCATGGAGGAACTTAAGACATGAGAGGGGCCAGCcaacttttcatcggttggccttgcca

gcctagacttggcgatggccggccgactctacgaaactcttactgcgaaactctcccacc

CCCAGTAGACAAAACCCTCTTAGGATTGCGCTCTTCAGAAGGTGCGCCTCCCCAGTCACA

ACCGGAAAGTCACTCAACTTGGCTCAAGTACCCTACTTAGTTTGTCTCTCTGTCGTGTTT

AGTATCAGCAacaataacaaatattgtaaataacatatatttatatggTAATTATATGGC

ATGTAAGCGTGACAACGATTATGTTTTCCATAGTTTGATAAACTCAACACCCGAAAAAGC

CGGTGACCAAAGGGTTGGGACCTCTTTGCACGAACTCCTATGGCTGCCAGACTATCTACA

GAGCTTTTTCGGAAGCTGACGCAACATCTGATCGCCACACAATCCTCGCGGACGTGGATG

GCGTGGCTACATGCAAGCAGTTTGACTGAGAGTTTTCGAGTgactattttaaaaGGACAA

TATTTCGTCCCAAGCGATAAAACTCGCTCACATGACTAAGTCGTTGCTTTaattcaactg

cgtgaaaagatttaaAGGGCAGaACTGTTTGTGTTTCGTATCCAGTTCACCGGCAATTAT

TGGCTACGCATGAAGCTCATTATCgtcgattctttacccaaacactgatgccattccaaa

tgagttgggccaaagatgtactCATGTCGGACAGGCACGATTTGGacttcgtgatcctca

agtctatcgcagtgctgctttattacaggctatgcttccaccctccattatttggtcaac

tGGACCTAGCTTTTGACAAGAATAGGCCAGCGTTCATCTATCTATCTTTCAAAAACATGC

AGTTGCTGCTGCGTAAAGGATGTAACATCGGATTTGGTGTGGAGACGGTGCTCGCTACGA

CTATACTGCCTTACTGTAGTGATCTGTCCCGCCaagcgatgaggggagcggcctgcctct

cgtgagcgtcaatccatacggttgcatgttcgtttgtttcaaaggcattcccaagaaggg

tttccagcttgacaactttctcatcggtgggtgtgaactccccggttagtgagtcaccgc

ccattttgggaactaccaggggggtgccaacgacctcctccgtcgacatctcagaccaat

ttcaattgcccaattgcaccgttctttctatgttgaagtcatgggcgactATGAAATCTC

CATCTTTGGTCGGTACTACCCCGATCTCGACCATATTGAAAAGCCTGAAAGATTTGCAAA

CTGCCCCTACACTGTTTTCCGGCAAGTGTGGCCCATGGAATCCACGGTGAACTATCAATT

GGCGGTCGCTTGAAGTCTTTCGAGCGATAAGCCAATTGGCGAGGCAGTGTTTCGCGATCG

CATCTTTAAGAGGAAAGTCCGATGCAAATCTCCATGTAAACCCAGTCGTTTCGGCCAAGG

CGGCCTCTAAATCAACTTGAGCAGCAGTTGTGTTAACGGAATCTGTTTTGACTGCCATTC

TGTAAGAAATGTCAGCAAAGGAAAAGCTAGTTAGAGAAGGAGAGCTTGCTCAATAGTGTG

TCTATTCTCAAATGAACTATACTAGCGCAATGAGTTGGTGTTGTTCGCTGATGGGAAAAA

GGAAACGCATCCCCCTCTTCTACAAAATTCTGTGGGCCCGGAGGGACTCCGTATGAAAGT

GCACAGCATGTGGACGCATCACCATGCAATTAGCAAGCTTAGCCAATGTCGTGTCTGTCA

GAGTGTGCAGCATGTGAGCCAAGCGCAATTACAATAAAAAAATAATATAAAAACTCTGCT

GAGGTCCGAACCGTCTCAAATGCTTTGAGGGCCATCCAAGCCAAGCGCACCTAACGCCTA

GCACACCATTGTTGCGCCGTTCGAGCATTAATGGTTCGGGACCTGGTGTCATCTGGAAAC

CTATGAAGCATGGACACGGCAAAatcagc

>tr3979_2

cCAATCCAAACTCATTGAAAGGCCTGTCATCAACCGGTCAACACGGTTCAATGTTTGCTC

GCGTGGCGCAGCAATGGTGTGCTAGGCGTTAGGTGCGCCTGGCTTGGATGGCCCTCAAAG

CATTTGAGGCAGTTCGGACCTCAGCAGAGATTTTATATTATTTTTTTGTTGTAATCGCGC

TTGGCCCACATGCTGCACACTCCGATAGACACGGCATTGGCTAAACCCGCTAATTGCGTG

GTGATGCGTCCACGTGCTGCGCACTTTCATGCGGAGTCCCCCCGAGTCCATAGAATAAGA

GGGGGACGCGTTTCCTTTTTCCCATCAGCAAACAACACCAACTCACTGTGCTAGTATAGT

TCATCTGAGAACAGACACACTGCTGAGCAAGCTCTCCTTCTCCAACTAGCTTTTTCTTTG

CTGACATTTCCTACAGAATGGCAGCAAAACAGATTCCGTTAACACAACTACTGCTCAGGT

TGATTTAGAGGCCGCCTTGGCCGAAGCGATCGGGTCTACATGGAGATTTGCATCAGACTT

TCCTCTTAAAAATGCAATCGCGAAACACTGCCTCGCCAATTGGCTTATCGCTCGAAAGAC

TTCAAGCAACCACCAATTGATAGTTCACCGTGAATTCCATGGGCCACACCTGCCGGAAAA

CAGCGTAGGGGCAGTCTGCAAATCTCTCAAACTTTTTAATATGGTCGAGATCAACGTAGT

ACCGACCAAAGATAAAGATTTCATAGTCGTCCACGACTTCAACACAGAAAGAACGACGCA

GTTAGGCAATTGAAATTGGTTTGATATGTCGACGAAAGAGGCTGTTGGCACCCCCCTGGT

AGTTCCCATAATGGGCGGTGACTCCCTAACCAGCGAGTTCACACCCACCGATGAGAAAGT

TGTCAAGCAAGAAACCCTTCTCAAGAATGCCTTTGAAACAAACAAACAGGCAACCATACG

GATTGACGCTCACGAGAGGCAGGCCGCTCCCCTCGTCGCTTGGCTGAGCCATAGGCCGGC

CTATCACGACAAGACAATCGTTATCTTTCATACATATGGATACAACAATGGACACGAGTT

CGCCTTGGCGGTCAATCAAGCCGGCGCTACAAAAAATTGGCGCGAAACTGTAGCGCTAAT

GCCAAACATTCTACCGAATGAACTCTCTAGGCTCGCGAAGCAATATCCACCAAATTGCAC

GTCGCATTTTTACGACCTCGTTGAGGCCAGCAAGAATTGGTTCGCATCTGTCTTCTCCCA

GAATATGATGGTGGTCGTCACTTTCCTCAAGATTGCGGGCACTGGCGAAGGATAGACTAA

CAAAGTCGTTGATCCAAATGGAAATGAGGTTCTGCATCCTAAAGAGAAGGAGGAATATCT

CCGCGATTCAGCTTCAATCGAAATCGCGAAATGAATCTGCGCCACCCATCCCGGAATCGA

GATTGGAGGCGTCACACAAACTTATGCTTATAAGAGTCGAGGACATCGCTACAAAATCAA

TCTTTGGACGGGTCAACCGAAGCGGTGGCCGACCCACACGACGAAGTACATCTGGGAAGA

TTTCGCCAAGCCTGGAAATGTGCTTAGATGGGGTCAAACGATTATCTCTGATCGCATTGG

TGACGACCTTGCAGCTTGTGTGCTTCAAAGCCGTGGAGAAAATTTTGTCAGCAGTCATGA

ATATCCTGCTGTTCATGTCGATTAGATGGGCGATCTGTGTCTTAGTCTAAGCTTCCGCTT

AACGCATCTTTGTTACTTTAATGTATTTTTTTAATGATATTGTTGGAGTTTTGAGCACAT

CTTAATCGCAAGTAGATATTTTTTTTGAATCGGATTCGACTTCAAGCAGATGTACTTCTT

TGTCTCTCGACAAATCTAATGACCCTGCGAGCCGGCCTCTCGCCTTAAACTAATTTCTGA

GAGTTAGGTCGGCGCCTCTCGTGGTTTCCATGTTCTATGGAAACGGCCGCTCGGGACTTG

CCTTCCCGCAACTCATCGAGCTTGCCTGGCCCGCTCGGGTGGTGGCACAAGCCATCGTTA

AATCGTTCGTAATCCTCTACTCTCACATAAATGAGAGGGGAATCCAAAGATGATCACTTT

TTAGCTAATTGGAATCTGCAATCCCAAAAGAGGCAAGCACTATGTGGGGTCCACAACTTA

TAGAATTTTGGCCTAACCATTCTGCATAATGGATCATGACATGGGCCATTTATGGCCGCA

AAAACTGGCACAAATTCCAACCTATTCAGCACAATGAACAAGTTCTTCCAATTGGAATAA

AGGAGGTTAAAATTATTcaagtagacatgacgtgCTGGCACGCCACTAGCCTCGCATTGA

TGCCACTCTTTCTTGCGCACACTTATATAATGCTGAGCTAATAATAGAGATAAAAATAGT

CAGCTTGTAAACTGGACCTTTATTACTGCCCCTAATCTCAAGATCAAAGCGACATATACC

TCTTCTCTTTCAAGTCTTTTTGCAACTTGTTCCAAAGCAAGCGTTTGTGAATCTGCCAGG

CAGCCTTCACGAAAATGGCTGATCAACTGTGTGAGGGGCGTTGGTCCGAAGCCAATCTAT

CATTCGTTGGGATATCCATCACCCCTGAATTCATCGATGAGGCCCTCAGAGGCTTTTGGG

AGTCTTTTGCCAAAAGAGCCGTTGGCTACAAAGAGGTTTACGTTCAGGTTGATCGAGATC

CTAAAGTTGCCTGGGAAGGAGCGCTCAATGGTTACCTCGGCAAATATTCTGGCGTAAAAA

ATGTTCATTTTGCGCCGAACAACGCCACGATTGGAAAGCCCCCACGCCATATCGACATTT

TGCTTCGGAAAAAATTTAGCGGCGAACTTCTTGCGACGCTCGAAGGTGTGGCAATTTCAA

ACGGTGCAACTGGTTGGCTTGCGCTCGCATGTGTAAACCTTCTCTTGAAGGCAAGACAAG

ATATTGATGTCTTCTTGTTTGGGGCAGGAAAAGTCGCCGAGGCTGTGATTCTTGCCCTCA

ATCATGGTGCGGCTGCAAAAATCAAAACCATGGCAGTGCTCAATCAAGCTCCAATTGAAG

GTGGGGTTCCAGCTCGATGCCGTCATTGATCGGAAGGTTATACCAAAAGCCAAACTCGTC

ATAACAGCAACAAATTCCGAGGAGCTCGGTACTCGAGGCCGACGAAATTACGCCAAATGC

AGTAACCATATCCCTCGGAAAAGATGAATTGCCCGCCGCCTACTTCGATCGCCTTTTGAA

CGCAGAGGGTCTGATTATTGGCGACGATCTGGATGCGATCGAATCGCGCAATGTCAATTC

CCTGGTGCTTTACTACTCGAAACGTGATTTGAAGCTGACAGAACATGGAAGGGATCATTG

GATAAAGAACTACGCCAATGTCCTTGCTGATCCAGCTCTCATGGAGGAACTTAAGACATG

AGAGGGGCCAGCCAACTTTTCATCGGTTGGCCTTGCCAGCCTAGACTTGGCGATGGCCGG

CCGACTCTACGAAACTCTTACTGCGAAACTCTCCCACCCCCAGTAGACAAAACCCTCTTA

GGATTGCGCTCTTCAGAAGGTGCGCCTCCCCAGTCACAACCGGAAAGTCACTCAACTTGG

CTCAAGTACCCTACTTAGTTTGTCTCTCTGTCGTGTTTAGTATCAGCAacaataacaaat

attgtaaataacatatatttatatggTAATTATATGGCATGTAAGCGTGACAACGATTAT

GTTTTCCATAGTTTGATAAACTCAACACCCGAAAAAGCCGGTGACCAAAGGGTTGGGACC

TCTTTGCACGAACTCCTATGGCTGCCAGACTATCTACAGAGCTTTTTCGGAAGCTGACGC

AGCATCTGATCGCCACACAATCCTCGCGGACGTGGATGGCGTGGCTACATGCAAGCAGTT

TGACTGAGAGTTTTCGAGTGACTATTTTAAAAGGACAATATTTCGTCCCAAGTGATAAAA

CTCGCTCACATGACTAAGTCGTTGCTTTAATTCAACTGCGTGAAAAGATTTAAAGGGCAG

AACTGTTTGTGTTTCGTATCCAGTTCACCGGCAATTATTGGCTACGCATGAAGCTCATTA

TCGTCGATTCTTTACCCAAACACTGATGCCATTCCAAATGAGTTGGGCCAAAGATGTACT

CATGTCGGACAGGCGCGATTTGGACTTCGTGATCCTCAAGTCTATCGCAGTGCTGCTTTA

TTACAGGCTATGCTTCCACCCTCCATTATTTGGTCAACTGGACCTAGCTTTTGACAAGAA

TAGGCCAGCGTTCATCTATCTATCTTTCAAAAACATGCAGTTGCTGCTGCGTAAAGGATG

TAACATCGgATTTGGTGTGGAGACGGTGCTCGCTACGACTATACTGCCTTACTGTAGTGA

TCTGTcccgccaagcGATGAGGGGAGCGGCCTGCCTCTCGTGAGCGTCAATCCATACGGT

TGCATGTTCGTTTGTTTCAAAGGCATTCCCAAGAAGGGTTTCCAGCTTGACAACTTTCTC

ATCGGTGGGTGTGAACTCCCCGGTTAGTGAGTCACCGCCCATTTTGGGAACTACCAGGGG

GTGCCAACGACCTCCTCCGTCGACATCTCAGACCAATTTCAATTGCCCAATTGCACCGTT

CTTTCTATGTTGAAGTCATGGGCGACTATGAAATCTCCATCTTTGGTCGGTACTACCTCG

ATCTCGACCATATTGAAAAGCCTGAAAGATTTGCAAACTGCCCCTACACTGTTTTCCGGC

AAGTGTGGCCCATGGAATCCACGGTGAACTATCAATTGGCGGTCGCTTGAAGTCTTTCGA

GCGATAAGCCAATTGGCGAGGCAGTGTTTCGCGATCGCATCTTTAAGAGGAAAGTCCGAT

GCAAATCTCCATGTAGACCCAGTCGTTTCGGCCAAGGCGGCCTCTAAATCAACTTGAGCA

GTAGTTGTGTTAACGGAATCTGTTTTGGCTGCCATTCTGTAAGAAATGTCAGCAAAGGAA

AAGCTAGTTAGAGAAGGAGAGCTTGCTCAATAGTGTGTCTATTCTCAAATGAACTATACT

AGCGCAATGAGTTGATGTTGTTCGCTGATGGAAAAAAGGAAACGCATCCCCCTCTTCTAC

AAAATTCTGTGGGCCCGGAGGGACTCCGTATGAAAGTGCACAGCatgtggacgcatcacc

atgcaattagcaagcttagccaatgtcgtgtctgtcagagtgtgcagcatgtgagccaag

cgcaattacaataaaaaaataaTATaAAAACtCTGCCGAGGTCCGAACCGTCTCAAATGC

TTTGAGGGCCATCCAAGCCAAGCGCACCTAACGCCTAGCACACCATTGTTGCGCCACTCG

AGCATTAATGGTTCGGGACCTGGTGTCATCTGGAAACCTATGAAGCATGGACACGGCAAA

ATCAGCGCCGTGGCGGTGTCAGACACGAGGACACGTCGGGGACacgtaca

>tr3983_1

caartttccctcacCTTGAAGTATCAATTTGATCTAGAAGCCAATCCAAACTCATTGAAA

GGCCTGTCATCAACCGGTCAACACGGTTCAATGTTTGCTCGCGTGGCGCAGCAATGGTGT

GCTAGGCGTTAGGTGCGCCTGGCTTGGATGGCCCTCAAAGCATTTGAGGCAGTTCGGACC

TCGGCAGAGATTTTATATTATTTTTTTGTTGTAATCGCGCTTGGCCCACATGCTGCACAC

TCCGATAGACACGGCATTGGCTAAACCCGCTAATTGCGTGGTGATGCGTCCACGTGCTGC

GCACTTTCATGCGGAGTCCCCCCGAGTCCATAGAATAAGAGGGGGACGCGTTTCCTTTTT

TCCATCAGCAAACAACACCAACTCACTGTGCTAGTATAGTTCATCTGAGAACAGACACAC

TGCTGAGCAAGCTCTCCTTCTCCAACTAGCTTTTTCTTTGCTGACATTTCCTACAGAATG

GCAGCAAAACAGATTCCGTTAACACAACTACTGCTCAGGTTGATTTAGAGGCCGCCTTGG

CCGAAGCGATCGGGTCTACATGGAGATTTGCATCAGACTTTCCTCTTAAAAATGCAATCG

CGAAACACTACCTTGCCAATTGGCTTATCGCTCGAAAGACTTCAAGCAACCACCAATTGA

TAGTTCACCGTGAATTCCATGGGCCACACCTGCCGGAAAACAGCGTAGGGGCAGTCTGCA

AATCTCTCAGACTTTTTAATATGGTCGAGATCAACGTAGTACCGACCAAAGATAAAGATT

TCATAGTCGTCCACGACTTCAACACAGAAAGAACGACGCAGTTAGGCAATTGAAATTGGT

TTGATATGTCGACGAAAGAGGCTGTTGGCACCCCCCTGGTAGTTCCCATAATGGGCGGTG

ACTCCCTAAAGAGCGAGTTCACACCCACCGATGAGAAAGTTGTCAAGCAAGAAACCCTtc

tcaagaatgcctttgaaacaaacaaacaggcaaccatACGGATTGACGCTCACGAGAGGC

AGGCCGCTCCCCTCGTCGCTTGGCTGAGCCATAGGCCGGCCTATCACGACAAGACAATCG

TTATCTTTCATACATATGGATACAACAATGGACACGAGTTCGCCTTGGCGGTCGATCAAG

CCGGCGCTACAAAAAATTGGCGCGAAACTGTAGCGCTAATGCCAAACATTCTGCCGAATG

AACTCTCTAGGCTCGCGAAGCAATATCCACCAAATTGCACGTCGCATTTTTACGACCTCG

TTGAGGCCAGCAAGAATTGGTTCGCATCTGTCTTCTCCCAGAATATGATGGTGGTCGTCA

CTTTCCTCAAGATTGCGGGCACTGGCAAAGGACAGACTAACAAAGTCGTTGATCCAAATG

GAAATGAGGTTCTGCATCCTAAAGAGAAGGAGGAATATCTCCGCGATTCAGCTTCAATCG

AAATCGCGAAATGAATCTGCGCCACCCATCCCGGAATCGAGATTGGAGGCGTCACACGAA

CTTATGCTTATAAGAGTCGAGGACATCGCTACAAAATCAATctttggacgggtcaaccga

agcggtggccgacccacgcgacgaagtacatctgggaagatttcgccaagcctggaaatg

cgctcagatggggtcaaacgattatctctgatcgcattggtgacgaccttGCAGCTTGTG

TGCTTCAAAGCCGTGGAGAAAATTTTGTCAGCAGTCATGAATATCCTGCTGTTCATGTCG

ATTAGATGGGCGATCTGTGTCTTAGTCTAAGCTTCCGCTTAACGCATCTTTGCTACTTTA

ATGTATTTTTTTAATGATATTGTTGGAGTTTTGAGCACATCTTAATCGCAAGTAGATATT

TTTTTTGAATCGGATTCGACTTCAAGCAGATGTACTTCTTTGTCTCTCGATAAATCTAAT

GACCCTGCGAGCCAGCCTCTCGCCTTAAACTAATTTCTGAGAGTTAGGTCGGCGCCTCTC

GTGGTTTCCATGTTCTATGGAAACGGCCGCTCGGGACTTGCCTTCCCGCAACTCATCGAG

CTTGCCTGGCCCGCTCAGGTGGTGGCACAAGCCATCGTTAAATCGTTCGTAATCCTCTAC

TCTCGCATAAATGAGAAGGGAATCCAAAGATGATCACTTTTCAGCTAATTGGAATCTGCA

ATCCCAAAAGAGGCAAGCACTATGTGGGGTCCACAACTTATAGAATTTTGGCCTAACCAT

TCTGCATAATGGATCATGACATAGGCCATTTATGGCCGCAAAAACTGGCACAAATTCCAA

CCTATTCAGCACAATGAACAAGTTCTTCCAATTGGAATAAAGGAGGTTAAAATTATTCAA

GTAGACATGACGTGCTGGCACGCCACTAGCCTCGCATTGATGCCACTCTTTCTTGCGCAC

ACTTATATAATGCTGAGCTAATAATAGAGATAAAAATAGTCAGCTTGTAAACTGGACCTT

TATTACTGCCCCTAATCTCAAGATCAAAGCAACATATACCTCTTCTCTTTCAAGTCTTTT

TGCAACTTGTTCTAAAGCAAGCGTTTGTGAATCTGCCAAGCAGCCTTCACGAAAATGGCT

GATCAACGGTGTGAGGGGCGTTGGTCCGAAGCCAATCTACCATTCGTTGGGATATCCATC

ACCCCTGAATTCATCGATGAGGCCCTCAGAGGCTTTTGGGAGTCTTTTGCCAAAAGAGCC

GTTGGCTACAAAGAGGTTTACGTTCAGGTTGATCGAGATCCTAAAGTTGCCTGGGAAGGA

GCGCTCAATGGTTACCTCGGCAAATATTCTGGCGTAAAAAATGTTCATTTTGCGCCGAAC

AACGCCACGATTGGAAAGCCCCCACGCCATATTGACATTTTGCTTCGGAAAAAATTTAGC

GGCGAACTTCTTGCGATGCTCGAAGGTGTGGCAATTTCAAACGGTGCAACTGGTTGGCTT

GCGCTCGCATGTGTAAACCTTCTCTTGAAGGCAAGACAAGATATTGATGTCTTCTTGTTT

GGGGCAGGAAAAGTCGCCGAGGCTGTGATTCTTGCCCTCAATCATGGTGCGGCTGCAAAA

ATCAAAACCATGGCAGTGCTCAGCCAAGCTCCAATTGAAGGTGGGGTTCCAGCTCGATGC

CGTCATTGATCGGAAGGTTATACCAAAAGCCAAACTCGTCATAACAGCAACAAATTCCGA

GGAGCTCGGTACTCGAGGCCGACGAAATTGCGCCAAATGCAGTAACCACATCCCTCGGAA

AAGATGAATTGCCCGCCGCCTACTTCGATCGCCTTTTGAACGCAGAGGGTCTGATTATTG

GCGACGATCTGGATGTGATCGAATCGCGCAATGTCAATTCCCTGGTGCTTTACTACTCGA

AACGTGATTTGAAACTGACAGAACATGGAAGGGATCATTGGATAAAGAACTACGCCAATG

TCCTTGCTGATCCAGCTCTCATGGAGGAACTTAAGACATGAGAGGGGCCAGCCAACTTTT

CATCGGTTGGCCTTGCCAGCCTAGACTTGGCGATGGCCGGCCGACTCTACGAAACTCTTA

CTGCGAAACTCTCCCACCCCCAGTAGACAAAACCCTCTTAGGATTGCGCTCTTCAGAAGG

TGCGCCTCCCCAGTCACAACCGGAAAGTCACTCAACTTGGCTCAAGTACCCTACTTAGTT

TGTCTCTCTGTCGTGTTTAGTATCAGCAACAATAACAAATATTGTAAATAACATATATTT

ATATGGTAATTATATGGCATGTAAGCGTGACAACGATTATGTTTTCCATAGTTTGATAAA

CTCAACACCCGAAAAAGCCGGTGACCAAAGGGTTGGGACCTCTTTGCACGAACTCCTATG

GCTGCCAGACTATCTACAGAGCTTTTTCGGAAGCTGACGCAACATCTGATCGCCACACAA

TCCTCGCGGACGTGGATGGCGTGGCTACATGCAAGCAGTTTGACTGAGAGTTTTCGAGTG

ACTATTTTAAAAGGACAATATTTCGTCCCAAGCGATAAAACTCGCTCACATGACTAAGTC

GTTGCTTTAATTCAACTGCGTGAAAAGATTTAAAGGGCAGAACTGTTTGTGTTTCGTATC

CAGTTCACCGGCAATTATTGGCTACGCATGAAGCTCATTATCGTCGATTCTTTACCCAAA

CACTGATGCCATTCCAAATGAGTTGGGCCAAAGATGTACTCATGTCGGACAGGCACGATT

TGGACTTCGTGATCCTCAAGTCTATCGCAGTGCTGCTTTATTACAGGCTATGCTTCCACC

CTCCATTATTTGGTCAACTGGACCTAGCTTTTGACAAGAATAGGCCAGCGTTCATCTATC

TATCTTTCAAAAACATGCAGTTGCTGCTGCGTAAAGGATGTAACATCGGATTTGGTGTGG

AGACGGTGCTCGCTACGACTATACTGCCTTACTGTAGTGATCTGTCCCGCCAAGCGATGA

GGGGAGCGGCCTGCCTCTCGTGAGCGTCAATCCATACGGTTGCATGTTCGTTTGTTTCAA

AGGCATTCCCAAGAAGGGTTTCCAGCTTGACAACTTTCTCATCGGTGGGTGTGAACTCGC

CGGTTAGTGAGTCACCGCCCATTTTGGGAACTACCAGGGGGGTGCCAACGACCTCCTCCG

TCGACATCTCAGACCAATTTCAATTGCCCAACTGCACCGTTCTTTCTATGTTGAAGTCAT

GGGCGACTATGAAATCTCCATCTTTGGTCGGTACTACCCCGATCTCGACCATATTGAAAA

GCCTGAAAGATTTGCAAACTGCCCCTACACTGTTTTCCGGCAAGTGTGGCCCATGGAATC

CACGGTGAACTATCAATTGGCGGTCGCTTGAAGTCTTTCGAGCGATAAGCCAATTGGCGA

GGCAGTGTTTCGCGATCGCATCTTTAAGAGGAAAGTCCGATGCAAATCTCCATGTAAACC

CAGTCGTTTCGGCCAAGGCGGCCTCTAAATCAACTTGAGCAGCAGTTGTGTTAACGGAAT

CTGTTTTGACTGCCATTCTGTAAGAAATGTCAGCAAAGGAAAAGCTAGTTAGAGAAGGAG

AGCTTGCTCAATAGTGTGTCTATTCTCAAATGAACTATACTAGCGCAATGAGTTGGTGTT

GTTCGCTGATGGGAAAAAGGAAACGCATCCCCCTCTTCTACAAAATTCTGTGGGCCCGGA

GGGACTCCGTATGAAAGTGCACAGCATGTGGACGCATCACCATGCAATTAGCAAGCTTAG

CCAATGTCGTGTCTGTCAGAGTGTGCAGCATGTGAGCCAAGCGCAATTACAATAAAAAAA

TAATATAAAAACtctgccgaggtccgaaccgTCTCAAATGCTTTGAGGGCCAHCCAAGCC

AAGCGCACCTAACGCCTAGCACACCATTGTTGCGCCGTTCGAGCATTAATGGTTCGGGAC

CTGGTGTCATCTGGAAACCTATGAAGCATGGACACGGCAAAATCAGCGCCGTGGCGGTGT

Cagacac

>tr3983_2

ctctccaartttccctcaccttGAAGTATCAATTTGATCTAGAAGCCAATCCAAACTCAT

TGAAAGGCCTGTCATCAACCGGTCAACACGGTTCAATGTTTGCTTGCGTGGCGCAGCAAT

GGTGTGCTAGGCGTTAGGTGCGCCTGGCTTGGATGGCCCTCAAAGCATTTGAGGCAGTTC

GGACCTCGGCAGAGATTTTATATTATTTTTTTGTTGTAATCGCGCTTGGCCCACATGCTG

CACACTCCGATAGACACGGCATTGGCTAAACCCGCTAATTGCGTGGTGATGCGTCCACGT

GCTGCGCACTTTCATGCGGAGTCCCCCCGAGTCCATAGAATAAGAGGGGGACGCGTTTCC

TTTTTCCCATCAGCAAACAACACCAACTCACTGTGCTAGTATAGTTCATCTGAGAACAGA

CACACTGCTGAGCAAGCTCTCCTTCTCCAACTAGCTTTTTCTTTGCTGACATTTCCTACA

GAATGGCAGCAAAACAGATTCCGTTAACACAACTACTGCTCAGGTTGATTTAGAGGCCGC

CTTGGCCGAAGCGATCGAGTCTACATGGAGATTTGCATCAGACTTTCCTCTTAAAAATGC

AATCGCGAAACACTGCCTCGCCAATTGGCTTATCGCTCGAAAGACTTCAAGCAACCACCA

ATTGATAGTTCACCGTGAATTCCATGGGCCACACCTGCCGGAAAACAGCGTAGGGGCAGT

CTGCAAATCTCTCAGACTTTTTAATATGGTCGAGATCAACGTAGTACCGACCAAAGATAA

AGATTTCATAGTCGTCCACGACTTCAACACAGAAAGAACGACGCAGTTAGGCAATTGAAA

TTGGTTTGATATGTCGACGAAAGAGGCTGTTGGCACCCCCTTGGTAGTTCCCATAATGGG

CGGTGACTCCCTAACCAGCGAGTTCACACCCACCGATGAGAAAGTTGTCAAGCAAGAAAC

CCTTCTCAAGAATGCCTTTGAAACAAACAAACAGGCAACCATACGGATTGACGCTCACGA

GAGGCAGGCCGCTCCCCTCGTCGCTTGGCTGAGCCATAGGCCGGCCTATCACGACAAGAC

AATCGTTATCTTTCATACATATGGATACAAAAATGGACACGAGTTCGCCTTGGCGGTCAA

TCAAGCCGGCGCTACAAAAAATTGGCGCGAAACTGTAGCGCTAATGCCAAACATTCTGCC

GAATGAACTCTCTAGGCTCGCGAAGCAATATCCACCAAATTGCACGTCGCATTTTTACGA

CCTCGTTGAGGCCAGCAAGAATTGGTTCGCATCTGTCTTCTCCCAGAATATGATGGTGGT

CGTCACTTTCCTCAAGATTGCGGGCACTGGCGAAGGACAGACTAACAAAGTCGTTGATCC

AAATGGAAATGAGGTTCTGCATCCTAAAGAGGAGGAGGAATATCTCCGCGATTCAGCTTC

AATCGAAATCGCGAAATGAATCTGCGCCACCCATCCCGGAATCGAGATTGGAGGCGTCAC

ACGAACTTATGCTTATAAGAGTCGAGGACATCGCTACAAAATCAATCTTTGGACGGGTCA

ACCGAAGCGGTGGCCGACCCACGCGACGAAGTACATCTGGGAAGATTTCGCCAAGCCTGG

AAATGCGCTCAGATGGGGTCAAACGATTATCTCTGATCGCATTGGTGACGACCttgCAGC

TTGTGTGCTTCAAAGCCGTGGAGAAAATTTTGTCAGCAGTCATGAATATCCTGCTGTTCA

TGTCGATTAGATGGGCGATCTGTGTCTTAGTCTAAGCTTCCgcttaacgcatcttTGCTA

CTTTAATGTATTTTTTTAATGATATTGTTGGAGTTTTGAGCACATCTTAATCGCAAGTAG

ATATTTTTTTTGAATCGGATTCGACTTCAAGCAGATGTACTTCTTTGTCTCTCGATAAAT

CTAATGACCCTGCGAGCCGGCCTCTCACCTTAAACTAATTTCTGAGAGTTAGGTCGGCGC

CTCTCGTGGTTTCCATGTTCTATGGAAACGGCCGCTCGGGACTTGCCTTCCCGCAACTCA

TCGAGCTTGCCTGGCCCGCTCGGGTGGTGGCACAAGCCATCGTTAAATCGTTCGTAATCC

TCTACTCTCGCATAAATGAGAGGGGAATCCAAAGATGATCACTTTTCAGCTAATTGGAAT

CTGCAATCCCAAAAGAGGCAAGCACTATGTGGGGTCCACAACTTATAGAATTTTGGCCTA

ACCATTCTGCATAATGGATCATGACATGGGCCATTTATGGCCACAAAAACTGGCACAAAT

TCCAACCTATTCAGCACAATGAACAAGTTCTTCCAATTGGAATAAAGGAGGTTAAAATTA

TTCGAGTAGACATGACGTGCTGGCACGCCACTAGCCTCGCATTGATGCCACTCTTTCTTG

CGCACACTTATATAATGCTGAGCTAATAATAGAGATAAAAATAGTCAGCTTGTAAACTGG

ACCTTTATTACTGCCCCTAATCTCAAGATCAAAGCGACATATACCTCTTCTCTTTCAAGT

CTTTTTGCAACTTGTTCCAAAGCAAGCGTTTGTGAATCTGCCAGGCAGCCTTCACGAAAA

TGGCTGATCAACTGTGTGAGGGGCGTTGGTCCGAAGCCAATCTATCATTCGTTGGGATAT

CCATCACCCCTGAATTCATCGATGAGGCCCTCAGAGgtttttgggagtcttttgccaaaa

GAGCCGTTGGCTACAAAGAGGTTTACGTTCAGGTTGATtGAGATCCTAAAGTTGCCTGGG

AAGGAGCGCTCAATGGTTACCTCGGCAAATATTCTGGCGTAAAAAATGTTCATTTTGCGC

CGAACAACGCCACGATTGGAAAGCCCCCACGCCATATCGACATTTTGCTTCGGAAAAAAT

TTAGCGGCGAACTTCTTGCAACGCTCGAAGGTGTGGCAATTTCAAACGGTGCAACTGGTT

GGCTTGCGCTCGCATGTGTAAACCTTCTCTTGAAGGCAAGACAAGATATTGATgtcttct

tgtttggggcaGGAAAAGtcgCCGAGGCTGTGATTCTTGCCCTCAATCATGGTGCGGCTG

CAAAAATCAAAACCATGGCAGTGCTCAGCCAAGCTCCAATTGAAGGTGGGGTTCCAGCTC

GATGCCATCATTGATCGGAAGGTTATACCAAAAGCCAAACTCGTCATAATAGCAACAAAT

TCTGAGGAGCTCGGTACTCGAGGCCGACGAAATTGCGCCAAATGCAGTAACCATATCCCT

CGGAAAAGATGAATTGCCCGCCGCCTACTTCGATCGCCTTTTGAACGCAGAGGGTCTGAT

TATTGGCGACGATCTGGATGCGATCGAATCGCGCAATGTCAATTCCCTGGTGCTTTACTA

CTCGAAACGTGATTTGAAGCTGACAGAACATGGAACGGATCATTGGATAAAGAACTACGC

CAATGTCCTTGCTGATCCAGCTCTCATGGAGGAACTTAAGACATGAGAGGGGCCAGCCAA

CTTTTCATCGGTTGGCCTTGCCAGCCTAGACTTGGCGATGGCCGGCCGACTCTACGAAAC

TCTTACTGCGAAACTCTCCCACCCCCAGTAGACAAAACCCTCTTAGGATTGCGCTCTTCA

GAAGGTGCGCCTCCCCAGTCACAACCGGAAAGTCACTCAACTTGGCTCAAGTACCCTACT

TAGTTTGTCTCTCTGTTGTGTTTAGTATCAGCAACAATAACAAATATTGTAAATAACATA

TATTTATATGGTAATTATATGGCATGTAAGCGTGACAACGATTATGTTTTCCATAGTTTG

ATAAACTCAACACCCGAAAAAGCCGGTGACCAAAGGGTTGGGACCTCTTTGCACGAACTC

CTATGGCTGCCAAACTATCTACAGAGCTTTGACGCAGCATCTGATCGCCACACAATCATC

GCGGACGTGGATGGCGTGGCTACATGCAAGCAGTTTGACTGAGAGTTTTCGAGTGACTAT

TTTAAAAGGACAATATTTCGTCCCAAGCGATAAAACTCGCTCACATGACTAAGTCGTTGC

TTTAATTCAACTGCGTGAAAAGATTTAAAGGGCAGAACTGTTTGTGTTTCGTATCCAGTT

CACCGGCAATTATTGGCTACGCATGAAGCTCATTATCGTCGATTCTTTACCCAAACACTG

ATGCCATTCCAAATGAGTTGGGCCAAAGATGTACTCATGTCGGACAGGCGCGATTTGGAC

TTCGTGATCCTCAAGTCTATCGCAGTGCTGCTTTATTACAGGCTATGCTTCCACCCTCCA

TTATTTGGTCAACTGGACCTAGCTTTTGACAAGAATAGGCCAGCGTTCATCTATCTATCT

TTCAAAAACATGCAGTTGCTGCGTAAAGGATGTAACATCGGATTTGGTGTGGAGACGGTG

CTCGCTACAACTATACTGCCTTACTGTAGTGATCTGTCCCGCCAAGCGATGAGGGGAGCG

GCCTGCCTCTCGTGAGCGTCAATCCATACGGTTGCATGTTCGTTTGTTTCAAAGGCATTC

CCAAGAAGGGTTTCCAGCTTGACAACTTTCTCATCGGTGGGTGTGAACTCGCCGGTTAGT

GAGTCACCGCCCATTTTGGGAACTACCAGGGGGGTGCCAACGACTTCCTCCGTCGACATC

TCAGACCAATTTCAATTGCCCAACTGCACCGTTCTTTCTATGTTGAAGTCATGGGCGACT

ATGAAATCTCCATCTTTGGTCGGTACTACCTCGATCTCGACCATATTGAAAAGCCTGAAA

GATTTGCAAACTGCCCCTACACTGTTTTCCGGCAAGTGTGGCCCATGGAATCCACGGTGA

ACTATCAATTGGCGGTCGCTTGAAGTCTTTCGAGCGATAAGCCAATTGGCGAGGCAGTGT

TTCGCGATCGCATCTTTAAGAGGAAAGTCCGATGCAAATCTCCATGTAGACCCAGTCGTT

TCGGCCAAGGCGGCCTCTAAATCAACTTAAGCAGCAGTTGTGTTAACGGAATCTGTTTTG

ACTGCCATTCTGTAAGAAATGTCAGCAAAGGAAAAGCTAGTTAGAGAAGGAGAGCTTGCA

CAATAGTGTGTCTATTCTCAAATGAACTATACTAGCGCAATGAGTTGGTGTTGTTCGCTG

ATGGAAAAAAGGAAACGCATCCCCCTCTTCTACAAAATTATGTGGGCCCGGAGGGACTCC

GTATGAAAGTGCACAGCATGTGGACGCATCACCATGCAATTAGCAAGCTTAGCCAATGTC

GTGTCTGTCAGAGTGTGCAGCATGTGAGCCAAGCGCAATTACAATAAAAAAATAATATAA

AAACTCTGCCGAGGTCCGAACCGTCTCAAATGCTTTGAGGGCCATCCAAGCCAAGCGCAC

CTAACGCCTAGCACACCATTATTGCGCCGCTCGAGCATTAATGGTTTGGGACTTGGTGTC

ATCTGGAAACCTATGAAGCATGGACACGGCAAAATCAGCGTTGTGGCGGtGTCAGACACG

AGGACACGCGGGgac

>qu0754_2

CAGCAATGGTGTGCTAGGCGTTAGGTGCGCCTGGCTGGGATGGCCCTCAAAGCATTTGAG

ACAGTTCGGACCTCGGCAGAGATTTTATATTATTTTTTTTGTTGTAATCGCGTTTGGCCC

ACATGCTGCACACTCCGACAGACACGGCATTGGCTAAACCCGCTAATTGCGTGGTGATGC

GTCCACGTGCTGCGCACTTTCATGTGGAGTCCCCCCGAGCCCGTAGAATTTTGTAGAAGA

GGGGGACGCGTTTCCTTTTTCCCATCAGCAAACAACACCAACTCACTGTGCTAGTATAGT

TCATCTGAGAACAGACACACTGCTGAGCAAGCTCTCCTTCTCCAACTAGCTTTTTCTTTG

CTGACATTTCCTACAGAATGGCAGCAAAACAGATTCCGTTAACACAACTGCTGCTCAGGT

TGATTTAGAGGCCGCCTTGGCCGAAGCGACCGGGTCTACATGGAGATTTGCATCAAACTT

TCCTCTTAAAGATGCGATCGCAAAACACTGCCTCGCCAATTGGCTTATCGCTTGAAAGAC

TTCAAGCAACCACCAATTGATAGTTCACCGTGAATTCCATGGGCCACACCTGCCGGAAAA

CAGTGTAGGGGCAGTCTGCAAATCTCTCAGACTTTTTAATATGGTCGAGATCAACGTAGT

ACCGACCAAAGATAGAGATTTCATAGTCATCCACGACTTCAACACAGAAAGAACGACGCA

GTTAGGCAATTGAAATTGGTCTGATATGTTGACGAAAGAGGCCATTGGCACCCCCCTAGT

AGTTCCCATAATGGGCGGTGACTCCCTAACCAGCGAGTTCACACCCACCGATGAGAAAGT

TGTCAAGCAAGAAACCCTTCTCAGGAATGCCTTTGAAACAAACAAACAGGCAACCATACG

GATTGACGCTCACGAGAGGCAGGCCGCTCCCCTCGTCGCTTGGCTGAGCCATAGGCCGGC

CTATCACGACAAGACAATCGTTATCTTTCATACATATGGATACAACAATGGACACAAGTT

CGCCTTGGCGGTCGATCAAGCCGACGCTACAGAAAATTGGCGCGAAACTGTAGCGCTAAT

GCCAAACATTCTGCCGAATGAACTCTCTAGGCTCGCGAAGCAATATCCACCAAATATCAC

GTCGCATTTTTACGACCTCGTTGAGGCCGGCAAGAATTGGTTCGCGTCTGTCTTCTCCCA

GAATATGATGGTGGTCGTCACTTTCCTCAAGATTGCGGGCACTGGCGAAGGACAGACTAA

CAAAGTCGTTGATCCAAATGGAAATGAGGTTCTGCATCCtaaagaGAAGGAGGAATATCT

CCGCGATTCAGCTTCAATCGAAATCGCGAAATGAATCTACGCCACCCATCCCGGAATCGA

GATTGGAGGCGTCACACGAACTTATGCTTATAAGAGTCGAGGACATCGCTACAAAATCGA

TCTTTGGACAGGTCAACCGAAGCGGTGGCCGACCTACGCGACGAAGTATATCTGGGAAGA

TTTCGCCAAGCCTGGAAATGCGCTCAGATGGGGTCAAACGATTATCTCTGATCGCATTGG

CGACGACCTTGCAGCTTGTGTGCTTCAAAGCCGTGGAGAAAATTTTGTCAGCAGTCATGA

ATATCCCGCTGTTCATGTCGATTAGATGGGCGATCTGTGTCTTAGTCTAAGCTTCCGCTT

AACGCATCTTTGCTACTTTAATGTATTTTTTTAAtgacattgttggagttttcaacAcat

cttaatcgcTGTAAGAAAAATTGATAGAGAGAGAATAAATTGTATGTATTATTATTGATT

GATATAATGTTATATTTATTACAATGATTCTTGATTCCTATATATAGAAGAGGGTGAGTG

TGTAGTAGGTAAGGAGTAGGTNNNTGGTGTAGTGGGTTAGGTTTAGTGGAGTAGGTTTAA

GTGGGTGAAGTAGGTGATAGGTTTTAACAATCGCaagTAGATATTTTTTTTGAATCGGAT

TCGACTTCAAGCAGATGTACTTCTTTGTCTCTCGATAAATCTAATGACCCTGCGAGCCGG

CCTCTCGCCTTAAACTAATTTCTGAGAGTTAGGTCGGCGCCTCTCGTGGTTTCCATGTTC

TATGGAAACGGCCTCTCGGGACTTGCCTTCCCGCAACTCATCGAGCTTGCCTGGCCCGCT

CGGGTGGTGGCGCAAGCCATCGTTAAATCGTTCGTAATCCTCTACTCTCGAATAAATGAG

AGGGGAATCCAAAGATGATCACTTTTCAGCTAATTGGAATCTGCAATCCTAAAAGAGGCA

AGCACTATGTGGGGTCCACAACTTATAGAATTTTGGCCTAACCATTCTGCATAATGGATC

ATGACATGGGCCATTTATGGCCGCAAAAACTGGCACAAATTCCAACCTGTTCAGCACAAT

GAACAAGTTCTTCCAATTGGAATAAAGGAGGTTAAAATTATTCGAGTAGACATGATGTGC

CGGCACACCACTAGCCTCGCATTGATGCCACCCTTTCTTGCGCACACTTATATAATGCTG

AGCTAATAATAGAGATAAAAATAGTCAGCTTGTAAACTGGACCTTTACTACTGCCCCTAA

TCTCAAGATCAAAGCGACATATACCTCCTCTCTTTCAAGTCTTTTTGCAACTTGTTCCAA

AGCAAGCATTCGTGAATCTGCCAGGCAACCTTCACGAAAATGGCTGATCAACTATGTGAG

GGGCGTTGGTCCGAAGCCAATCTATCATTCGTTGGGATATCCATCACCCCTGAATTCATC

GATGAGGCCCTCAGAGGCTTTTGCCAAAAGAGCCGTTGGCTACAAAGAGGTTTACGTTCA

GGTTGATCGAGATCCTAAAGTTGCCTGGGAAGGAGCGCTCAATGGTTAcctcgacaaata

ttctGGCGTCAAAAATGTTCATTTTGCGCCGAACAACGCCGCGATTGGAAAGCCCCCACG

CCATATCGACATTTTGCTTCGGAAAAAATTTAGCGGCGAATTTCTTGCAACGCTCGAAGG

TGTGGCAATTTCAAACAGTGCAACTGGTTGGCTTGCACTCGCATGCGTAAACCTTCTCTT

GAAGGCAAGACAAGATATTGATGTCTTCTTGTTTGGGGCGGGAAAAGTCGCCGAGGCTGT

GATTCTTGCCCTCAATCACGGTGCGGCTGCAAAAATCAAAACCATGGCAGTGCTCAGCCA

AGCTCCAATCGAAGGTGGGGTTCCAACTCGATGTCGTCATTGATCAGAAGGTTATACCAA

AAGCCAAACTCGTCATAACAGCAACAAATTCCGAGGAGCTCGTACTCGAGGCCGACGAAA

TTGCGCCAAATGCAGTAACCATATCCCTCGGAAAAGATGAATTGCTCGCCGCCTACTTCG

ATCGCCTTTTGAACGCAGAGGGTCTGATTATCGGCGACGATCTGGATGCGATCGAATCGC

GCAATGTCAATTCCCTGGTGCTTTACTACTCGAAACGTGATTTGAAGCTGACAGAACATG

GAAGGGATCATTGGATAAAGAACTACGCCAATGTCCTTGTTGATCCAGCTCTCATGGAGG

AACTTAAGACATGGGAGGGGCCAGCCAACTTTTCATCGGTTGGCCTTGCCAGCCTAGACT

TGGCGATGGCCGGCCGACTCTACGAAACTCTTACTGCAAAACTCTCCCACCCCCAGTAGA

CAAGACCCTCTTAGGATTGCGCTCTTCAGAAGGTGCGCCTCCCCAGTCACAACCGGAAAG

TCACTCAACTTGGCTCAAGTACCCTACTTAGTTTGTCTCTCTGTCGTGTTTAGTATCAAC

AACAATAACAAATATTGTAAACAACATATATTTATATGGTAATTATATGGCATGTAAGCG

TGACAACGATTATGTTTTCCATAGTTTGATAAACTCAACACCCGAAAAAGCCGGTGACCA

AAGGGTTGGGACCTCTTTGCACGAACTCCTATGGCTGCCAGACTATCTACAGAGCTTTTT

CGGAAGCTGACGCAGCATCTGATCGCTACACAATCCTCGCGGACGTGGATGGCGTGGCTA

CACGCAAGCAGTTTGACTGAGAGTTTTCGAGTGACTATTTTAAAAGGACGATATTTCGTC

CCAAGCAATAAAACTCGCTCACATGACTAAGTCGTTGCTTTAATTCAGCTGCGTGAAAAG

ATTTAAAGGGCAGAACTGTTTGTGTTTCGTATCCAGTTCACCGGCAATTATTGGCTACGC

ATGAAGCTCATTATCGTCGATTCTTTACCCAAACACTAATGTCATTCCAAATGAGTTGGG

CCAAAGATGTACTCATGTCGGACAGGCGCGATTTGGACTTCGTGATCCTCAAGTCTATCG

CAGTGCTGCTTTATTACAGGCTATGCTTCCACCCTCCATTATTTGGTCAACTGGACCTGG

CTTTTGACAAGAATAGGCCAGCGTTCATCTATCTATCTTTCAAAAACATGCAGTTGCTGC

TGCGTAAAGGATGTAACATCGGATTTGGTGTGGAGACGGTGCTTGCTACGACTATACTGC

CTTACTGTAGTGATCTGTCCTGCCAAGCGACGAGGGGAGCAGCCTGCCTCTCGTGAGCGT

CAATCCATACGGTTGCATGTTCGTTTGTTTCAAAGGCATTCCCAAGAAGGGTTTCCAGCT

TGACAACTTTCTCATTGGTGGGTGTGAACTCGCCGGTTAGTGAGTCACCGCCCATTTTGG

GAACTACCAGGGGGGTGCCAACGACCTCCTCCGTCGACATCTCAGACCAATTTCAATTGC

TCAACTGCGCCATTCTTTCTATGTTGAAGTCATGGGCGACTATGAAATCTCCATCTTTGG

TCGGTACTACCTCGATCTCGACCATATTGAAAAGCCTGAGAGATTTGCAAACTGCCCCTA

CACTGTTTTCCAGCAAGTGTGGCCCATGGAATCCACGGTGAACTATCAATTGGCGGTCGC

TTGAAGTCTTTCGAGCGATAAGCCAATTGGCGAGGCAGTGTTTCGCGATCGCATCTTTAA

GAGGAAAGTCCGATGCAAATCTCCATGTAGACCCGGTCGCTTCGGCCAAGGCGGCCTCTA

AACCAACTTGAGCAGCAGTTGTGTTAACGGAATCTATTTTGACTGCCATTCTGTAAGAAA

CGTCAGCAAAGGAAAAGCTAGTTAGAGAAGGAGAGCTTGCTCAATAGTGTGTCTATTCTC

ATATGAACTATACTAGCGTAATGAGTTAGTGTTGTTCGCTGATGGGAAAAAGGAAATGCA

TCCCCCTCTTCTACAAAATTCTGTGGGCCCGGAGGGACTCCGTATGAAAGTGCACAGCAT

GTGGACGCATCACCATGCAATTAGCGAGCTTAGCCAATGCCGTGTCTGTCAGAGTGTGTA

GCATGTGGGCCAAGCGCAATTACAATAAAAAAATAATATAAAAACTCTGCCGAGGTCTGA

ACCGTCTCAAATGCTTTGAGGGCCATCCAAGCCAAGCGCACCTAACGCCTAGCACACCAT

TGTTGCGCCGCTCGAGCA

>qu0754_1

CAGCAATGGTGTGCTAGGCGTTAGGACCTGGCTTGGATGGCCCTCAAAGCATTTGAGACA

GTTCGGACCTCGGCAGAGATTTTATATTATTTTTTTGTTGTAATCGCGCTTGGCCCACAT

GCTGCACACTCCGACAGACACGGCATTGGCTAAGCCCGCTAATTGCGTGATGATGBGTTC

ACGTGCTGCGCACTTTCATGCGGAGTCCCCCCGAGCCCGTAGAATTTTGTAGAAGAGGGG

GACGCGTttcctttttcccatcagcaaacaacaccaactcactgtgcTAGTATAGTTCAT

CTGAGAACAGACACACTGCTGAGCAAGCTCTCCTTCTCCAACTAGCTTTTTCTTTGCTGA

CATTTCCTACAGAATGGCAACCAAAACAGATTCCGTTAACACAACTGCTGCTCAGGTTGA

TTTGGAGGCCGCCTTGGCCGAAGTGACCGGGTCTACATGAAGATTTGCATCAGACTTTCC

TCTTAaAGATGCGATCGCAAAACACTGCCTCGCCAACTGGCTTATCGCTCGAAAGACTTC

AAGCAACCACCAATTGATAGTTCACCGTGAATTCCATGGAACACACCTGCCGGAAAACAA

CGTAGGGGCAGTCTGCAAATCTCTCAGACTTTTTAATATGGTCGAGATCGACGTAGTACC

AACCAAAGATAGAGATTTCATAGTCGTCCACGACTTCAACACAGAAAGAATGACGCAGTT

AGGCAATCGAAATTGGTCTGATATGTCGACGAAGGAGGCCGTTGACACCCCCCTGGAAGT

TCCCAAAATGGGCGGTGACTCCCTAACTAGCGAGTTCACATCCACCGATGAGAAAGTTGT

CAAGCAGGAAACCCTTCTCAGGAATGCCTTTGAAACAAACTAACAGGCAACCATATGGAT

TGACGCTCACGAGAGGCAGGCCGCTCCCCTCGTCGCTTGGCTGAGCCATAGGCCAGCCTA

TCACGACAAGACAATCATTATCTTTTATACATATGGATACAACAATGGACACGAGTTCGC

CTTGGCGGTCGATCAAGCCGGCACTACGGCAAATTGGCGCGAAACTATAGCTCTAATGCC

AAACATTCTGCCGGATGAACTCTCTAGGCTCGCGAAGCAATATCCACCAAATTGCATGTC

GCATTTTTACGACCTTATTGCGGCTGGCAAGAATTGGTTCGCGGCTGTCTTCTCCCAGAA

TATGATGGTGGTCGTCACTTTCCTCAAGATTGCGGGCACTGGCGAAGGACAAACTAACAA

AGTCGTTGATCCAAATGGAAATGAGGTTCAACATCCTAAAGAGAAGGAGAAATATCTCCG

CAATTCAACTTCAATCGAAATCGCGAAATGAATTTGCGCCACCCATCCCAGAATCGAGAT

TGGAGGCGTCACACGAACTTATGCTTATAAGAGTCGAGGACATCGTTACAAAACCGATCT

TTGGACGGGTCAGCCGAAGCGGTGGCCGACCCACGCGACGAAGTATATCTGGGAGGATTT

CGCCAAGCCTGGAAATGCGCTCAGATGGGGTCAAACGATTATCTCTGATCGCATTGGCGA

CGACCTTGCAGCTTGTGTGCTTCAAAGCCGTGGAGAAAATTTTGTCAGCAGTCATAAATA

TCCCACTGTTCATGTCGATTAGATGGGCGATCTGTGTCTTGGTCTAAGCTTCCGCTTAAC

GCATCTTTGCTACTTTAATGTATTTTTTTAATGACATTGTTGGAGTTTTCAACACATCTT

AATCGCAAGTAGATATTTTTTTTTAATCGAATTCAATTTCAAGCAGATGTACTTCTTTGT

CTCTCGATAAATCTAATGACCCTGCGAGCCGGCCTCTCGCCTTAAACTAATTTCTGAGAG

TTAGGCCGATGCCTCTCATGGTTTCCATGTTCTATGGAAACGGCCTCTCGGGACTTGCCT

TCCCGCAACTCATCGAGCTTGCCTGCTCGGGTGGTGGCGCAAGCCATCGTTAAATCATTC

GTAATCCTTTACTCTCGCATAAATGAGAGGGGAATCCAAAGATGATCACTTTTCAGCTAA

TTGGAATCTGCAATCCCAAAAGTGGCAAGCACTATGTGGGGTCCACAACTTATAGAATTC

TGGCCTAACCATTCTGCATAATGGATCATGACATGGGCCATTTATGGCCGCAAAAACTGG

CACAAATTCCAACCTGTTCAGCACAATGAACAAGTTCTTCCAATTGGAATAAGGAGGTTA

AAATTATTCGAGTAGACATGACGTGTCGGCACGCCACTAGCCTCGCATTCATGCCACCCT

TTCTTGCGCACACTTATATAATGCTGAGCTAATAATAGAGATAAAAATAGTCAGCTTGTA

AACTGGACCTTTACTACTGCCCCTAATCTCAAGATCAAAGCGACATATACCTCCTCTCTT

TCAAGTCTTTTTGCAACTTGTTCCAAAGCAAGCGTTCGTGAATCTGCCAGGCAGCCTACA

CGAAAATGGCCGATCAACTGTGTGAGGGGCATTAGTCCGAAGCCGATCTATCATTCGTTG

GGATATCCATCACCCCTGAATTCATCGATGAGGCCCTCAGAGGCTTTTGGGAGTCTTTTG

CCAAAAGAGCCGTTGGCTACAAAGAGGTTTACGTTCAGGTTGATCGAGATCCTAAAGTTG

CCTGGAAAGGAGTGCTCAATGGTTACCTCGACAAATATTCTGGCATCAAAAATGTTCATT

TTGCACCGAACAATGCCGCGATTGGAAAGCCCCCACACCATATCGACATTTTGCTTCGGA

AAAAATTCGGCGGCGAACTTCTTGCGACGCTCGAAGGTGTGGCAATTTCAAACGGTGCAA

CTGGTTGACTTGCGCTTGCATGCGTAAACCTTCTCTTGAAGGCAAGACAAGATATTGATG

TCTTGTTTGGGGCGGGAAAAGTCGCCGAGGTTGTGATTCTTGCCCTCAATCATGGTGCAG

CTGCAAAAATCAAAACCATGGCAGTGCTCAGCCAAGCTCCAATCGAAGGTGGGGTTCCAG

CTCGATGCCGTCGATGATCGGAAGGTTATACCAAAAGCCAAACTCGTCATAACAGCAACA

AATTCCAAGGAGCTCGTACTCGAGGCCGACGAAATTGCGCCAAATGCAATAACCATATCC

CTCGGAAAAGATGAATTGCCCGCCGTCTACTTCGATCGCCTTTTGAACGCAGAGGGTCTG

ATTATCGGCGACGATCTGGATGCGATCGAATCGCGCAATGTCAATTCCCTGGTGCTTTAC

TACTCGAAACGTGATTTGAAGCTGACAGAACATGGAAGGGATCATCGGATAAAGAACTAC

GCCAATGTCCTTGCCGATCCAGCTCTCATGGAAAAACTTAAGACATGGGAGGGGCCAGCC

AACTTTTCATCGGTTGGCCTTGCCAACCTAGACTTGGCAATGGCCGGCCGACTCTACGAA

ACTCTTACTGCGAAACTCTCCGACCCCCAGTAGACAAGACCCTCTTAGGATTGCGCTCTT

CAGAAGGTGCGCCTCCCCAGTCACAACCGGAAAGTCACTCAACTTGGCTCAAGTACCCTA

CTTAGTTTGTCTCTCTTTCGTGTTTTGTATCAGCAACAATAACAAATATTGTAAATAACA

TATATTTATATGGTAATTATATGGCATGTAAGCGTGACAACGATTATGTTTTCCATAGTT

TGCTAAACTCAACACCCGAAAAAGCTGGTGACCAAAGGGTTGGCACCTCTTTGCACGAAC

TCCTATGGCTGCCAGACTATCTACAGAGCTTTTTCGAAAGCTAATGTAGCATCTGATCGC

CACACAATCCTCGCGGACGTGGATGGCGTGGCTACACGCAAGCAGTTTGACTGAGAGTTT

TCGAGTGACTATTTTAAAAGGACAATATTTCGTCCCAAGCGATAAAACTCGCTCACATGA

CTAAGTCGTTGCTTTAATTCGGTTGCGTGAAAAGATTTAAAGGGCAAAACTATTTGTGTT

TCGTTTCCAGTTCACCGGCAATTATCAGCTACGCATGAAGCTCATTATCGTCGATTCTTT

ACCCAAACACTGATGCCATTCCAAATGAGTTGGGCCAAAGATGTACTCTTGTCGGACAGG

CGCGATTTGGACTTCGTGATCCTCAAGTCTATCGCAGTGCTGCTTTATTACAGGCTATGC

TTCCACCCTCCATTATTTGGTCAACTGGACCTGGCTTTTGACGAGAATAGGCCAGCGTTC

ATCTATCTATCTTTCAAAAACATGTAGTTGCTGCTGCGTAAAGGATATAACATCGGATTT

GGTGTGGAGACGGTGCTCGCTACGACTATATTGCCTTATTGTAGTGATCTGTCCCGCCAA

GCGACGAGGGGAGCGGTCTGCCTCTCGTGAGCGCCAATCCATACGATTGCATGTTCGTTT

GTTTCAAAGGCATTCCCAAGAAGGGTTTCCAGCTTGACAACTTTCTCATCGGTGGATGTG

AACTCGCCGGTTAGGGAGTCACCGCCCATTTTGGAAACTACCAGGAGGGTGCCAACGACC

TCCTCCGTCGACATCTCAGACCAATTTCAATTGCCCAACTGCGCCGTTCTTTCTATGTTG

AAGTCATGGGCGACTATGAAATCTCTATCTTTGTTCAGTACTACGTCGATCTCGACCATA

TTGAAAAGCCTGAGAGATTTGCAAACTGCCCTTACACTGTTTTCCGACAGGTGTGACCCA

TGGAATCCACGGTGAACTATCAATTGGAGGTCGCTTGAAGTCTTTCGAGCGATAAGCCAA

TTGGCGAGGCAGTGTTTCGCGATCGCATCTTTAAGAGGAAAGTCCGATGCAAATCTCCAT

GTAGACACGGTCGCTTCGGCCAAGGCGGCCTCTAAATCAACTTGAGCAACAGTTGTGTTA

ACGGAATCTGTTTTGGTTGCCATTCTGTAGGAAATGTCACCAAAGGAAAAGCTAGTTAGA

GAATGAGAGCTTGCTCAACAGTGTGTTTATTCTCAGATGAACTATACTAGCGCAATGAGT

TGGTGTTGTTCGCTGATGGGAAAAAGGAAATGCATCCCCCTCTTCTACAAAATTATGTGG

GCCCAGAGGGACTCCGTATGAAAATGCACAGCATGTGGATGCATCACCATGCAATTAGCG

AGCTTAGCCAATGCCGTGTCTGTCAGAGTGTACAGCATGTGGGCCAAGCGCAATTACAAT

AAAAAAATAATATAAAATCTCTGCCGAGGTCCGAACCGTCTCAAATGCTTTGAGGGCCAT

CCAAGCCAAGCACACCTAACGCCTAGCACACCATTGTTGCGCCGCTCGAGCA

>qu0755_1

tcaccttgaagtATCAATTTGATCCAGAAGCCAATCCAAACTCATTGAAAGGCCTGTCAT

CAACCTGTCAACACGGTTCAATGTTGCTCGAGCGGCGCAGCAATGGTGTGCTAGGCGTTA

GGACCTGGCTTGGATGGCCCTCAAAGCATTTGAGACAGTTCGGACCTCGGCAGAGATTTT

ATATTATTTTTTTTGTTGTAATCGCGTTTGGCCCACATGCTGCACACTCCGACAGACACG

GCATTGGCTAAACCCGCTAATTGCGTGGTGATGCGTCCACGTGCTGCGCACTTTCATGTG

GAGTCCCCCCGAGCCCGTAGAATTTTGTAGAAGAGGGGGACGCGTTTCCTTTTTCCCATC

AGCAAACAACACCAACTCACTGTGCTAGTATAGTTCATCTGAGAACAGACACACTGCTGA

GCAAGCTCTCCTTCTCCAACTAGCTTTTTCTTTGCTGACATTTCCTACAGAATGGCAGCA

AAACAGATTCCGTTAACACAACTGCTGCTCAGGTTGATTTAGAGGCCGCCTTGGCCGAAG

CGACCGGGTCTACATGGAGATTTGCATCAAACTTTCCTCTTAAAGATGCGATCGCAAAAC

ACTGCCTCGCCAATTGGCTTATCGCTTGAAAGACTTCAAGCAACCACCAATTGATAGTTC

ACCGTGAATTCCATGGGCCACACCTGCCGGAAAACAGTGTAGGGGCAGTCTGCAAATCTC

TCAGACTTTTTAATATGGTCGAGATCAACGTAGTACCGACCAAAGATAGAGATTTCATAG

TCATCCACGACTTCAACACAGAAAGAACGACGCAGTTAGGCAATTGAAATTGGTCTGATA

TGTTGACGAAAGAGGCCATTGGCACCCCCCTAGTAGTTCCCATAATGGGCGGTGACTCCC

TAACCAGCGAGTTCACACCCACCGATGAGAAAGTTGTCAAGCAAGAAACCCTTCTCAGGA

ATGCCTTTGAAACAAACAAACAGGCAACCATACGGATTGACGCTCACGAGAGGCAGGCCG

CTCCCCTCGTCGCTTGGCTGAGCCATAGGCCGGCCTATCACGACAAGACAATCGTTATCT

TTCATACATATGGATACAACAATGGACACAAGTTCGCCTTGGCGGTCGATCAAGCCGACG

CTACAGAAAATTGGCGCGAAACTGTAGCGCTAATGCCAAACATTCTGCCGAATGAACTCT

CTAGGCTCGCGAAGCAATATCCACCAAATATCACGTCGCATTTTTACGACCTCGTTGAGG

CCGGCAAGAATTGGTTCGCGTCTGTCTTCTCCCAGAATATGATGGTGGTCGTCACTTTCC

TCAAGATTGCGGGCACTGGCGAAGGACAGACTAACAAAGTCGTTGATCCAAATGGAAATG

AGGTTCTGCATCCTAAAGAGAAGGAGGAATATCTCCGCGATTCAGCTTCAATCGAAATCG

CGAAATGAATCTACGCCACCCATCCCGGAATCGAGATTGGAGGCGTCACACGAACTTATG

CTTATAAGAGTCGAGGACATCGCTACAAAATCGATCTTTGGACAGGTCAACCGAAGCGGT

GGCCGACCTACGCGACGAAGTATATCTGGGAAGATTTCGCCAAGCCTGGAAATGCGCTCA

GATGGGGTCAAACGATTATCTCTGATCGCATTGGCGACGACCTTGCAGCTTGTGTGCTTC

AAAGCCGTGGAGAAAATTTTGTCAGCAGTCATGAATATCCCGCTGTTCATGTCGATTAGA

TGGGCGATCTGTGTCTTAGTCTAAGCTTCCGCTTAACGCATCTTTGCTACTTTAATGTAT

TTTTTTAATGATATTGTTGGAGTTTTCAACACATCTTAATCGCTGTAAGAAAAATTGATA

GAGAGAGAATAAATTGTATGTATTATTATTGATTGATATAATGTTATATTTATTACAATG

TTCTTGATTCCTATATATAGAAGAGGGTGAGTGTGTAGTAGGTAAGGAGTNNNTTTAGTG

GAGTAGGTGAGGTGGTAGTGGTGTAGTGGGTTAGGTTTAGTGGAGTAGGTTAAGTGGGTG

AAGTAGGTGATAGGTTTTAACAATCGCAAGTAGATATTTTTTTTGAATCGGATTCGACTT

CAAGCAGATGTACTTCTTTGTCTCTCGATAAATCTAATGACCCTGCGAGCCGGCCTCTCG

CCTTAAACTAATTTCTGAGAGTTAGGTCGGCGCCTCTCGTGGTTTCCATGTTCTATGGAA

ACGGCCTCTCGGGACTTGCCTTCCCGCAACTCATCGAGCTTGCCTGGCCCGCTCGGGTGG

TGGCGCAAGCCATCGTTAAATCGTTCGTAATCCTCTACTCTCGAATAAATGAGAGGGGAA

TCCAAAGATGATCACTTTTCAGCTAATTGGAATCTGCAATCCTAAAAGAGGCAAGCACTA

TGTGGGGTCCACAACTTATAGAATTTTGGCCTAACCATTCTGCATAATGGATCATGACAT

GGGCCATTTATGGCCGCAAAAACTGGCACAAATTCCAACCTGTTCAGCACAATGAACAAG

TTCTTCCAATTGGAATAAAGGAGGTTAAAATTATTCGAGTAGACATGATGTGCCGGCACA

CCACTAGCCTCGCATTGATGCCACCCTTTCTTGCGCACACTTATATAATGCTGAGCTAAT

AATAGAGATAAAAATAGTCAGCTTGTAAACTGGACCTTTACTACTGCCCCTAATCTCAAG

ATCAAAGCGACATATACCTCCTCTCTTTCAAGTCTTTTTGCAACTTGTTCCAAAGCAAGC

ATTCGTGAATCTGCCAGGCAACCTTCACGAAAATGGCTGATCAACTATGTGAGGGGCGTT

GGTCCGAAGCCAATCTATCATTCGTTGGGATATCCATCACCCCTGAATTCATCGATGAGG

CCCTCAGAGGCTTTTGCCAAAAGAGCCGTTGGCTACAAAGAGGTTTACGTTCAGGTTGAT

CGAGATCCTAAAGTTGCCTGGGAAGGAGCGCTCAATGGTTACCTCGGCAAATATTCTGGC

GTCAAAAATGTTCATTTTGCGCCGAACAACGCCGCGATTGGAAAGCCCCCACGCCATATC

GACATTTTGCTTCGGAAAAAATTTAGCGGCGAATTTCTTGCAACGCTCGAAGGTGTGGCA

ATTTCAAACAGTGCAACTGGTTGGCTTGCACTCGCATGCGTAAACCTTCTCTTGAAGGCA

AGACAAGATATTGATGTCTTCTTGTTTGGGGCGGGAAAAGTCGCCGAGGCTGTGATTCTT

GCCCTCAATCACGGTGCGGCTGCAAAAATCAAAACCATGGCAGTGCTCAGCCAAGCTCCA

ATCGAAGGTGGGGTTCCAACTCGATGTCGTCATTGATCAGAAGGTTATACCAAAAGCCAA

ACTCGTCATAACAGCAACAAATTCCGAGGAGCTCGTACWCGAGGCCGACGAAATTGCGCC

AAATGCAGTAACCATATCCCTCGGAAAAGATGAATTGCTCGCCGCCTACTTCGATCGCCT

TTTGAACGCAGAGGGTCTGATTATCGGCGACGATCTGGATGCGATCGAATCGCGCAATGT

CAATTCCCTGGTGCTTTACTACTCGAAACGTGATTTGAAGCTGACAGAACATGGAAGGGA

TCATTGGATAAAGAACTACGCCAATGTCCTTGTTGATCCAGCTCTCATGGAGGAACTTAA

GACATGGGAGGGGCCAGCCAACTTTTCATCGGTTGGCCTTGCCAGCCTAGACTTGGCGAT

GGCCGGCCGACTCTACGAAACTCTTACTGCAAAACTCTCCCACCCCCAGTAGACAAGACC

CTCTTAGGATTGCGCTCTTCAGAAGGTGCGCCTCCCCAGTCACAACCGGAAAGTCACTCA

ACTTGGCTCAAGTACCCTACTTAGTTTGTCTCTCTGTCGTGTTTAGTATCAACAACAATA

ACAAATATTGTAAACAACATATATTTATATGGTAATTATATGGCATGTAAGCGTGACAAC

GATTATGTTTTCCATAGTTTGATAAACTCAACACCCGAAAAAGCCGGTGACCAAAGGGTT

GGGACCTCTTTGCACGAACTCCTATGGCTGCCAGACTATCTACAGAGCTTTTTCGGAAGC

TGACGCAGCATCTGATCGCTACACAATCCTCGCGGACGTGGATGGCGTGGCTACACGCAA

GCAGTTTGACTGAGAGTTTTCGAGTGACTATTTTAAAAGGACGATATTTCGTCCCAAGCA

ATAAAACTCGCTCACATGACTAAGTCGTTGCTTTAATTCAGCTGCGTGAAAAGATTTAAA

GGGCAGAACTGTTTGTGTTTCGTATCCAGTTCACCGGCAATTATTGGCTACGCATGAAGC

TCATTATCGTCGATTCTTTACCCAAACACTAATGTCATTCCAAATGAGTTGGGCCAAAGA

TGTACTCATGTCGGACAGGCGCGATTTGGACTTCGTGATCCTCAAGTCTATCGCAGTGCT

GCTTTATTACAGGCTATGCTTCCACCCTCCATTATTTGGTCAACTGGACCTGGCTTTTGA

CAAGAATAGGCCAGCGTTCATCTATCTATCTTTCAAAAACATGCAGTTGCTGCTGCGTAA

AGGATGTAACATCGGATTTGGTGTGGAGACGGTGCTTGCTACGACTATACTGCCTTACTG

TAGTGATCTGTCCTGCCAAGCGACGAGGGGAGCAGCCTGCCTCTCGTGAGCGTCAATCCA

TACGGTTGCATGTTCGTTTGTTTCAAAGGCATTCCCAAGAAGGGTTTCCAGCTTGACAAC

TTTCTCATTGGTGGGTGTGAACTCGCCGGTTAGTGAGTCACCGCCCATTTTGGGAACTAC

CAGGGGGGTGCCAACGACCTCCTCCGTCGACATCTCAGACCAATTTCAATTGCTCAACTG

CGCCATTCTTTCTATGTTGAAGTCATGGGCGACTATGAAATCTCCATCTTTGGTCGGTAC

TACCTCGATCTCGACCATATTGAAAAGCCTGAGAGATTTGCAAACTGCCCCTACACTGTT

TTCCAGCAAGTGTGGCCCATGGAATCCACGGTGAACTATCAATTGGCGGTCGCTTGAAGT

CTTTCGAGCGATAAGCCAATTGGCGAGGCAGTGTTTCGCGATCGCATCTTTAAGAGGAAA

GTCCGATGCAAATCTCCATGTAGACCCGGTCGCTTCGGCCAAGGCGGCCTCTAAACCAAC
[truncated: 852,936 more chars]
