# Supplementary material for: Conservation of an Agrobacterium cT-DNA insert in Camellia section Thea reveals the ancient origin of tea plants from a genetically modified ancestor
Source: Front Plant Sci. 2022 Dec 6;13:997762. doi: 10.3389/fpls.2022.997762 (PMC9763466; doi:10.3389/fpls.2022.997762)
Supplement: Supplementary Table 3 — Details for indels. [file Table_3.docx]

| position | type | sequence change | direct repeat | x |
| --- | --- | --- | --- | --- |
| 239 | del | TGCG=A |  | 10 |
| **255** | **ins** | **Plant repeat** | **GCCTT** | **1** |
| 258 | del | CTC |  | 3 |
| 438 | del | AATTTTG |  | 1 |
| 440 | del | TTTTG |  | 1 |
| 441 | del | TTTGTAG (also in G240) |  | 134 |
| 442 | del | TTGTAGA |  | 3 |
| 444 | del | GTAGAA |  | 1 |
| 446 | ins | AGA |  | 1 |
| 449a | ins | AGCCCGTAGAATTTTGTAGA |  | 1 |
| 449b | ins | AGCCCGTAGAATTTTGTAGAAGCCCGTAGAATTTTGTAGA |  | 1 |
| 449c | Ins | AGAGGG |  | 3 |
| **626** | **ins** | **Plant repeat** | **GATTTAG** | **1** |
| 640 | del | CCTTGGCCG |  | 1 |
| 1012 | del | AAGAGCGAGTTCAC |  | 1 |
| 1277 | ins | CGCTAATGCC=GCTCTGGTAGTTCCC |  | 2 |
| 1508 | del | AGA |  | 7 |
| 1712 | del | AGCCTG |  | 1 |
| 1790 | del | AAAGCCGTGG=CGATCT |  | 1 |
| 1928 | del | GGAGTTT |  | 2 |
| **1946** | **ins** | **Plant repeat** | **AATCGC** | **17** |
| 2001 | del | CTT (=TCT) |  | 1 |
| 2146a | del | TGCCTGGCCCG |  | 2 |
| 2146b | del | TGCCTGGCC |  | 1 |
| 2152 | del | GGCCC |  | 15 |
| 2337 | dupl | ATCATGACATCC/GG |  | 3 |
| 2553 | del | AAACTGGACCTTTATTACTGTC |  | 1 |
| 2644 | del | AGCGTTCGTGAATCTGCCAGGC |  | 1 |
| 2744 | ins | CACC |  | 1 |
| **2748** | **ins** | **Plant repeat** | **CCTGAATTCA** | **8** |
| 2767 | del | TCAGAGGCTTT |  | 1 |
| 2768 | del | AGAGGCTTTTG |  | 1 |
| 2769a | del | GAGGCTTT |  | 1 |
| 2769b | del | GAGGCTTTTGG/GCTTTTGGGAG |  | 3 |
| 2770 | ins | AAGGCTTTTGG |  | 1 |
| 2772 | del | GCTTTTGGGAG |  | 3 |
| 2774 | del | TTTTGGGAGT |  | 1 |
| 2779a | del | GGAGTCTTTTG/C(T)TTTTGGGAGT (also in G240) |  | 55 |
| 2779b | del | GGAGT |  | 1 |
| 2781a | del | AGTCTTTTTGC |  | 2 |
| 2781b | del | AGTCTTTTTGCC |  | 1 |
| **2824** | **ins** | **Plant repeat** | **GATCGAGATC** | **1** |
| 3085 | del | TCT/CTT |  | 3 |
| 3182 | ins | GCGGTGGAAAGTCTAATCACGAGTTGGTCGAG |  | 2 |
| 3383 | ins | TCT |  | 1 |
| 3471 | ins | TTC |  | 1 |
| 3525 | ins | AGATTG |  | 1 |
| 3574 | del | TTT |  | 1 |
| 3664 | del | CAAAACCCTCTTAGGATTGCGCTCTTCAGAAGGTGCGCCTCCCCAGTCA |  | 1 |
| 3687 | del | CTTCAGAAGGTGCGCC |  | 3 |
| 3736 | del | GCTCAAG |  | 1 |
| 3789 | del | TAA/AAT |  | 2 |
| 3905 | ins | GATCGG |  | 1 |
| 3962a | del | TTCGGAAGC |  | 40 |
| 3962b | del | TCGGAAGT |  | 5 |
| 3962c | del | TTCGGAA=GC |  | 1 |
| 3962d | ins | TTCGGAAGC=GACGCAGGAGCTT |  | 1 |
| 3962e | ins | TTCGGAAGC=GACGCA |  | 1 |
| 3962f | ins | TTCGGAAGC=GACGCAGCA |  | 1 |
| 3969 | del | AGC |  | 1 |
| 4183 | del | TCACCGGC |  | 4 |
| 4440 | del | TGC/TAC |  | 14 |
| 5238 | del | CTACAAA |  | 1 |
| **5347** | **ins** | **Plant repeat** | **AGCATGTG** | **1** |
